# Supplementary material for: Predictive Potential of RNA Polymerase B (II) Subunit 1 (RPB1) Cytoplasmic Aggregation for Neoadjuvant Chemotherapy Failure
Source: Int J Mol Sci. 2023 Nov 1;24(21):15869. doi: 10.3390/ijms242115869 (PMC10650411; doi:10.3390/ijms242115869)
Supplement: Supplementary file 1 [file ijms-24-15869-s001.zip › Figure S1.pdf]

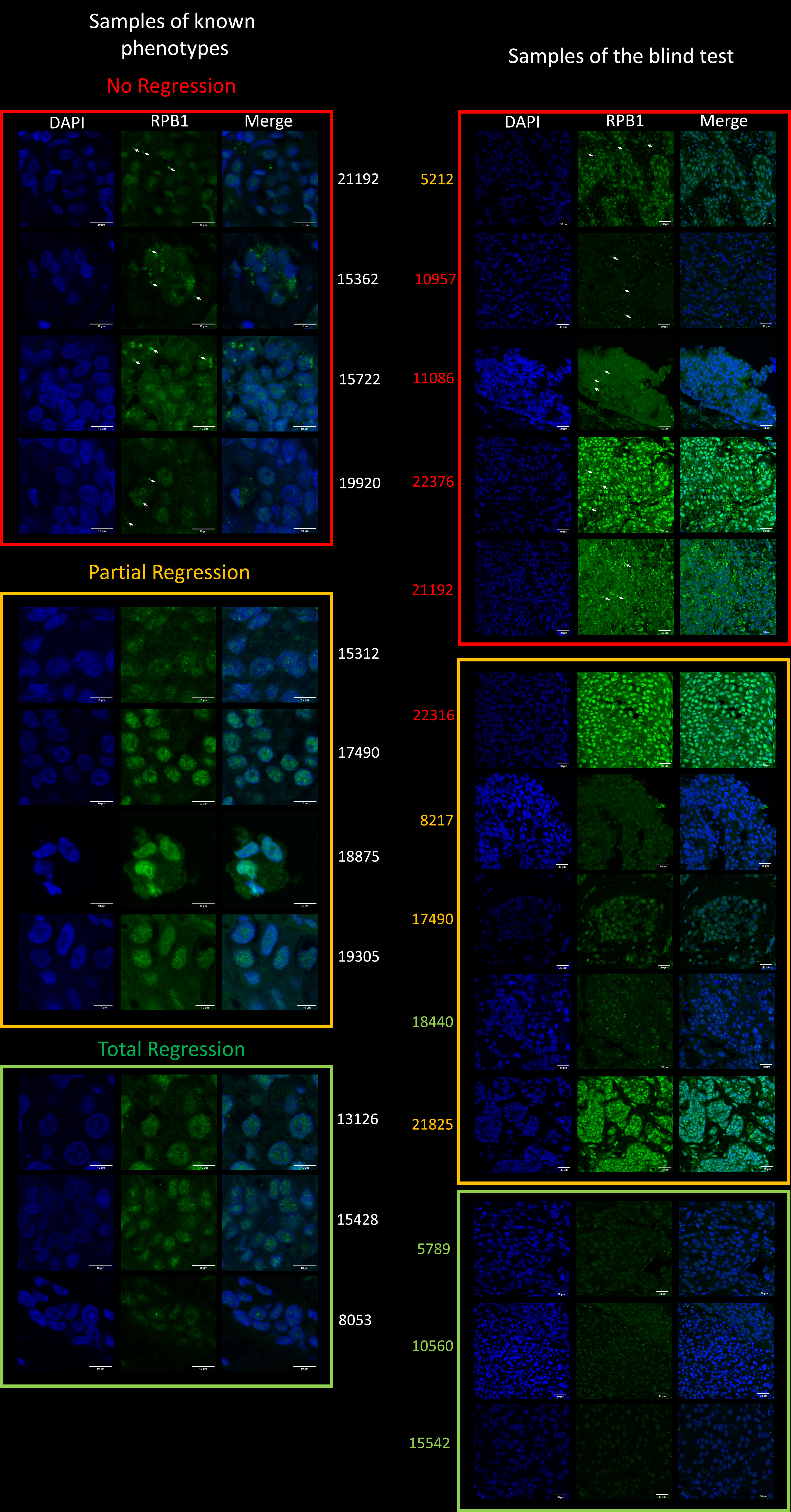
Samples of known phenotypes
No Regression
DAPI
RPB1
Merge
21192
15362
15722
19920
Partial Regression
15312
17490
18875
19305
Total Regression
13126
15428
8053
Samples of the blind test
DAPI
RPB1
Merge
5212
10957
11086
22376
21192
22316
8217
17490
18440
21825
5789
10560
15542
