# Supplementary material for: Predictive Potential of RNA Polymerase B (II) Subunit 1 (RPB1) Cytoplasmic Aggregation for Neoadjuvant Chemotherapy Failure
Source: Int J Mol Sci. 2023 Nov 1;24(21):15869. doi: 10.3390/ijms242115869 (PMC10650411; doi:10.3390/ijms242115869)
Supplement: Supplementary file 1 [file ijms-24-15869-s001.zip › ijms-2597436-supplementary caption.pdf]

Supplementary Figure S1.

One representative fluorescent image from each of the studied invasive carcinoma of NST biopsies is listed and categorized according to response to neoadjuvant treatment. "No regression", "partial regression" and "total regression" cases are inside red, yellow and green boxes, respectively. Case numbers for the internal record are shown in the middle. Eleven cases with known phenotypes are on the left, and additional 13 cases who participated in the blind test are on the right. Case numbers with different colors as the bracket indicate the cases that have been categorized falsely (three cases), and the color of the number represents the falsely assumed category, respectively. Arrows indicate three examples for RPB1 foci in each of the images taken from tumors belonging to the real (left) or assumed (right) "no regression" phenotypic category. Scale bars for images on the left: 10 μm and right: 30 μm.

Supplementary Figure S2.

Antibodies recognizing nucleus engaged RPB1 phosphorylated at the fifth serine of its heptapeptide C-terminal repeats (P5-RPB1-CTD) reveals cytoplasmic staining in none of the three studied phenotypic categories as indicated on the left. Nuclei are revealed using DAPI shown in cyan. RPB1 is shown in red, and the merged image of the two staining is highlighted. Scale bar: 10 μm.

Supplementary Figure S3.

4-8 fields of 46,509 μm2 of each sample in the blind experiment is shown stained with DAPI (blue), Rpb1 (green) and Hsp90 (red) respectively. Combinations of the pairwise merged channels and all the three are showed along with a phase contrast image taken from all specimens.

Supplementary Table S1.

Both carcinoma of NST and clear cell renal cell carcinoma samples from patients are listed for demographic details and other specific attributes respectively. These include for every patient for carcinoma NST: gender, age, ER Allred: Estrogen receptor score, PR Allred: Progesterone receptor score, HER2 occurrence, regression score as: TR1 (Total regression), TR2 (Partial regression), TR3 (No regression). For Clear cell renal cell carcinoma: gender, age, tumour size, stadium, metastasis and grade. In case of clear cell renal cell carcinoma samples RPB1 has appeared or not in cytoplasmic foci as indicated. The sample numbers are as described in [13].

The Allred score is a summation of the positive tumor cell proportion and intensity, ranging from 0 to 8, with 12 possible values. The distribution of positive tumor cell proportion is as follows: 0% (0), 1% (1), 1-10% (2), 11-33% (3), 34-66% (4), and >66% (5). The intensity scale is negative (0), weak (1), moderate (2), and strong (3). The Allred score is determined by adding the positive tumor cell proportion and intensity, where values from 0 to 2 are considered negative, while values from 3 to 8 are considered positive.

Supplementary Table S2.

Confusion matrixes of the blind test. Accurate and erroneous predictions are highlighted and are used to calculate the accuracy (ACC) and error rate (ERR) of classification of the cases to no regression, partial regression and total regression categories according to [15]. Matthews Correlation Coefficient (MCC) is highlighted as calculated according to [16]. The complete confusion matrix and the confusion matrixes for each regression categories are highlighted on separate pages.
