# Supplementary material for: Predictive Potential of RNA Polymerase B (II) Subunit 1 (RPB1) Cytoplasmic Aggregation for Neoadjuvant Chemotherapy Failure
Source: Int J Mol Sci. 2023 Nov 1;24(21):15869. doi: 10.3390/ijms242115869 (PMC10650411; doi:10.3390/ijms242115869)
Supplement: Supplementary file 1 [file ijms-24-15869-s001.zip › Table S2.pdf]

Complete Confusion Matrix

		True (Observed) Class		
Predicted Class		Total Regression	Partial Regression	No Regression
	Total Regression	3	0	0
	Partial Regression	1	3	1
	No Regression	0	1	4

No Regression Classification

	True Class	
Predicted Class		Positive	Negative
	Positive	4	1
	Negative	1	7

ERR = 2 / 13 = 15.385%

ACC = 11/13 = 84.615%

False Positives: 1
False Negatives: 1

MCC = 0.675

Partial Regression Classification

	True Class		
Predicted Class		Positive	Negative
	Positive	3	2
	Negative	1	7

ERR = 3 / 13 = 23.077%

ACC = 10 / 13 = 76.923%

False Positives: 2
False Negatives: 1

MCC = 0.5007

Total Regression Classification

	True Class	
Predicted Class	Positive	Negative
Positive	3	0
Negative	1	9

ERR = 1 / 13 = 7.692%

ACC = 12 / 13 = 92.308%

False Positives: 0
False Negatives: 1

MCC = 0.8216
