# Supplementary figures and images for: Predictive Potential of RNA Polymerase B (II) Subunit 1 (RPB1) Cytoplasmic Aggregation for Neoadjuvant Chemotherapy Failure
Source: Int J Mol Sci. 2023 Nov 1;24(21):15869. doi: 10.3390/ijms242115869 (PMC10650411; doi:10.3390/ijms242115869)

DAPI

P5-RPB1-CTD

Merge

No regression

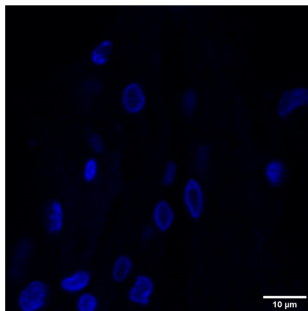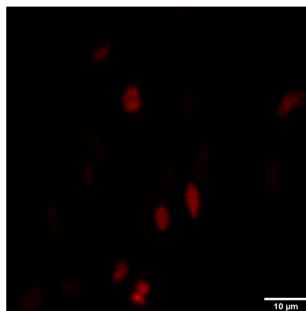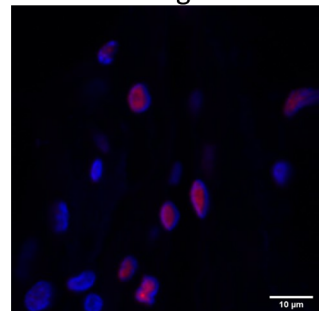

Partial regression

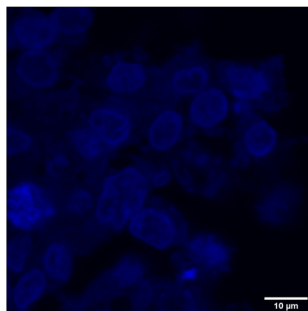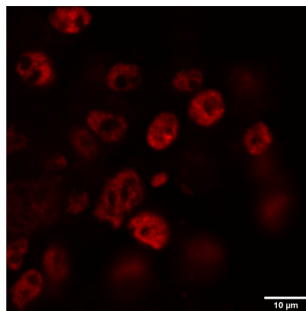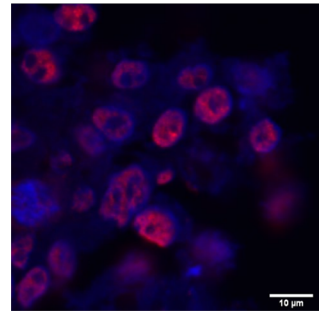

Total regression

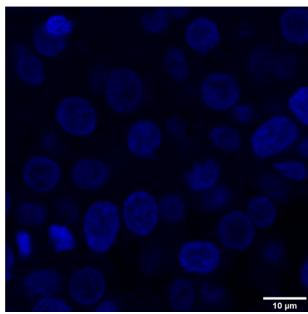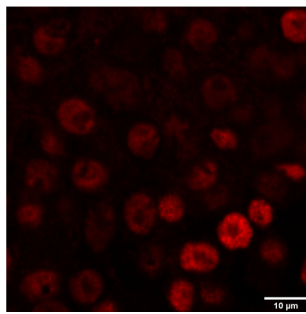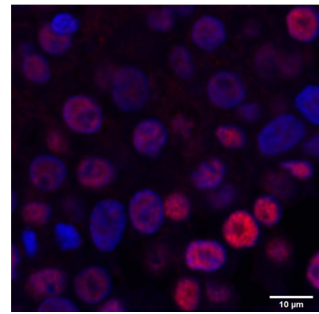

Supplement: Supplementary file 1 [file ijms-24-15869-s001.zip › Figure S2.pdf]

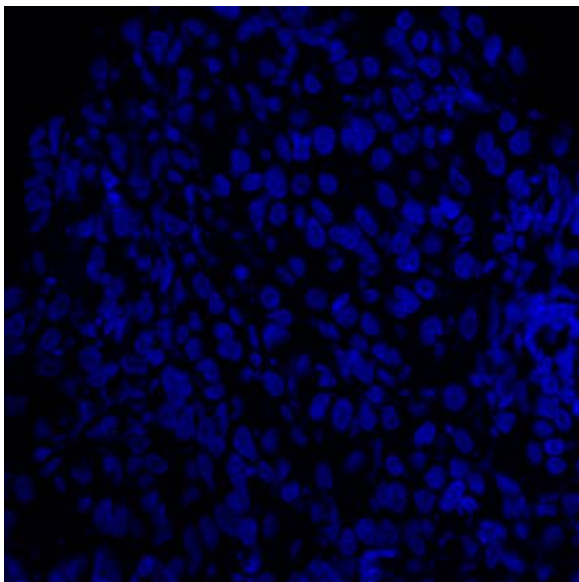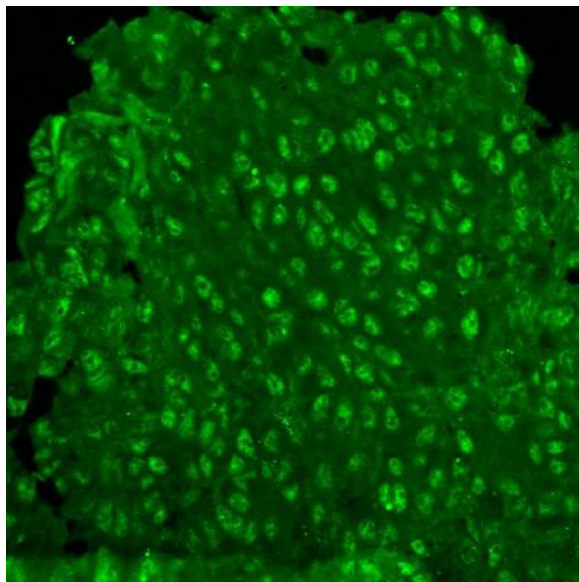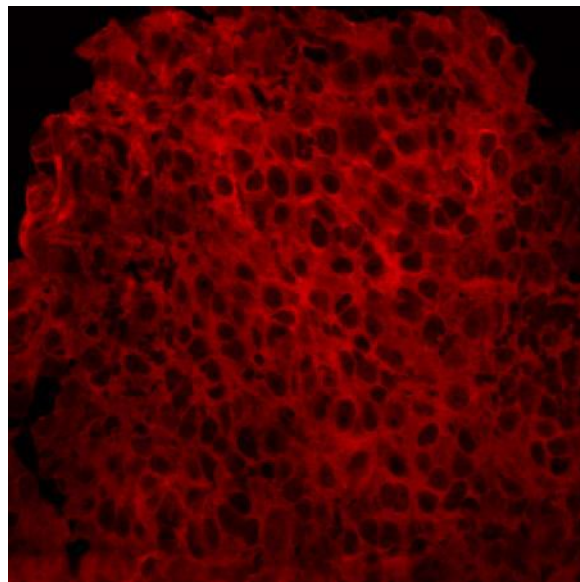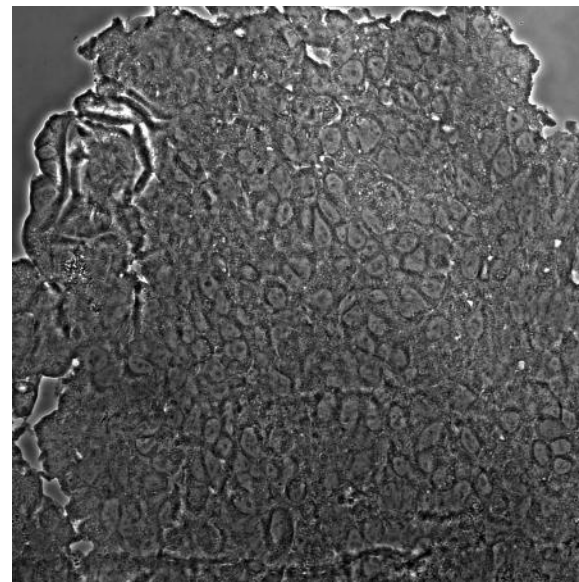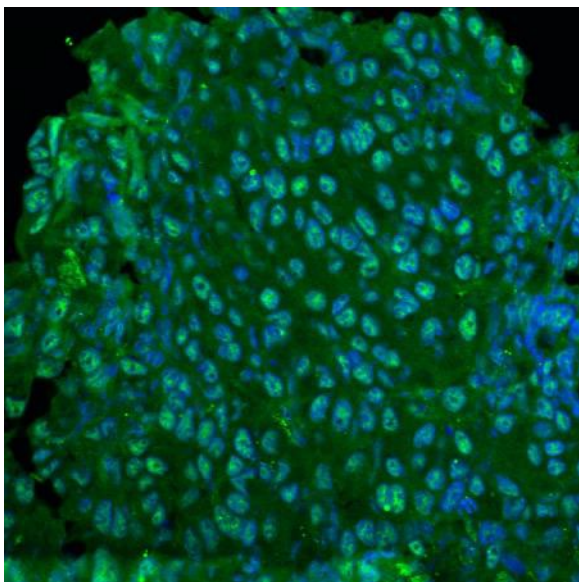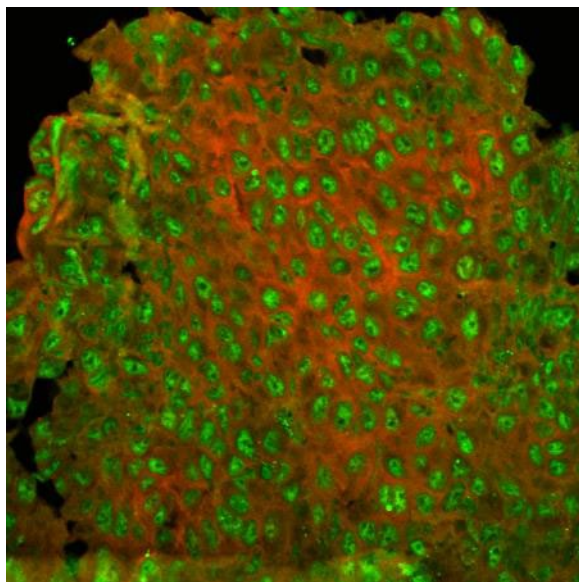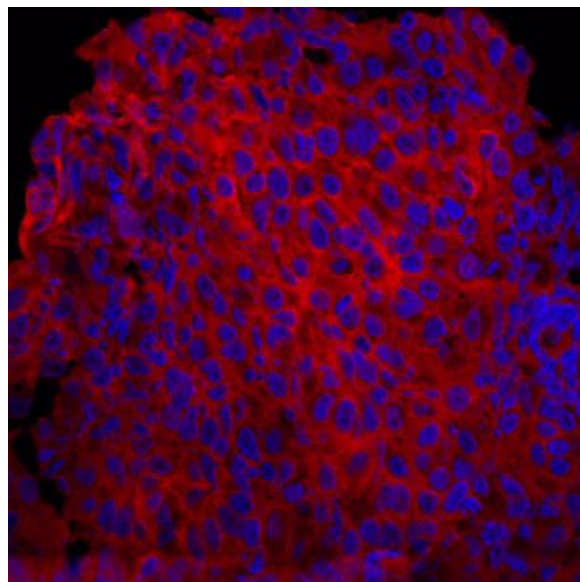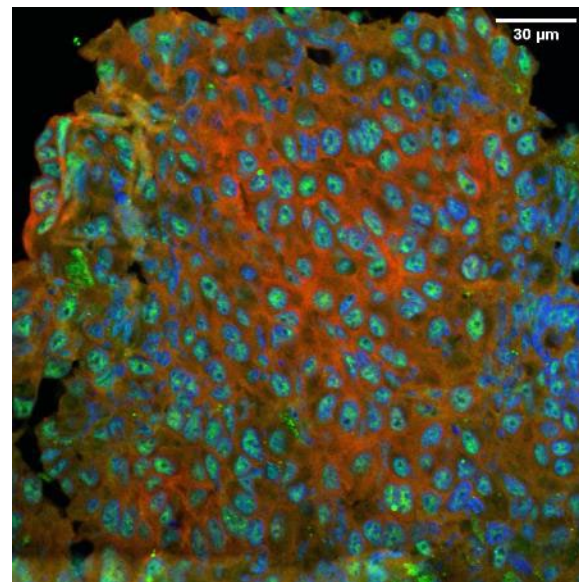

5212\_00

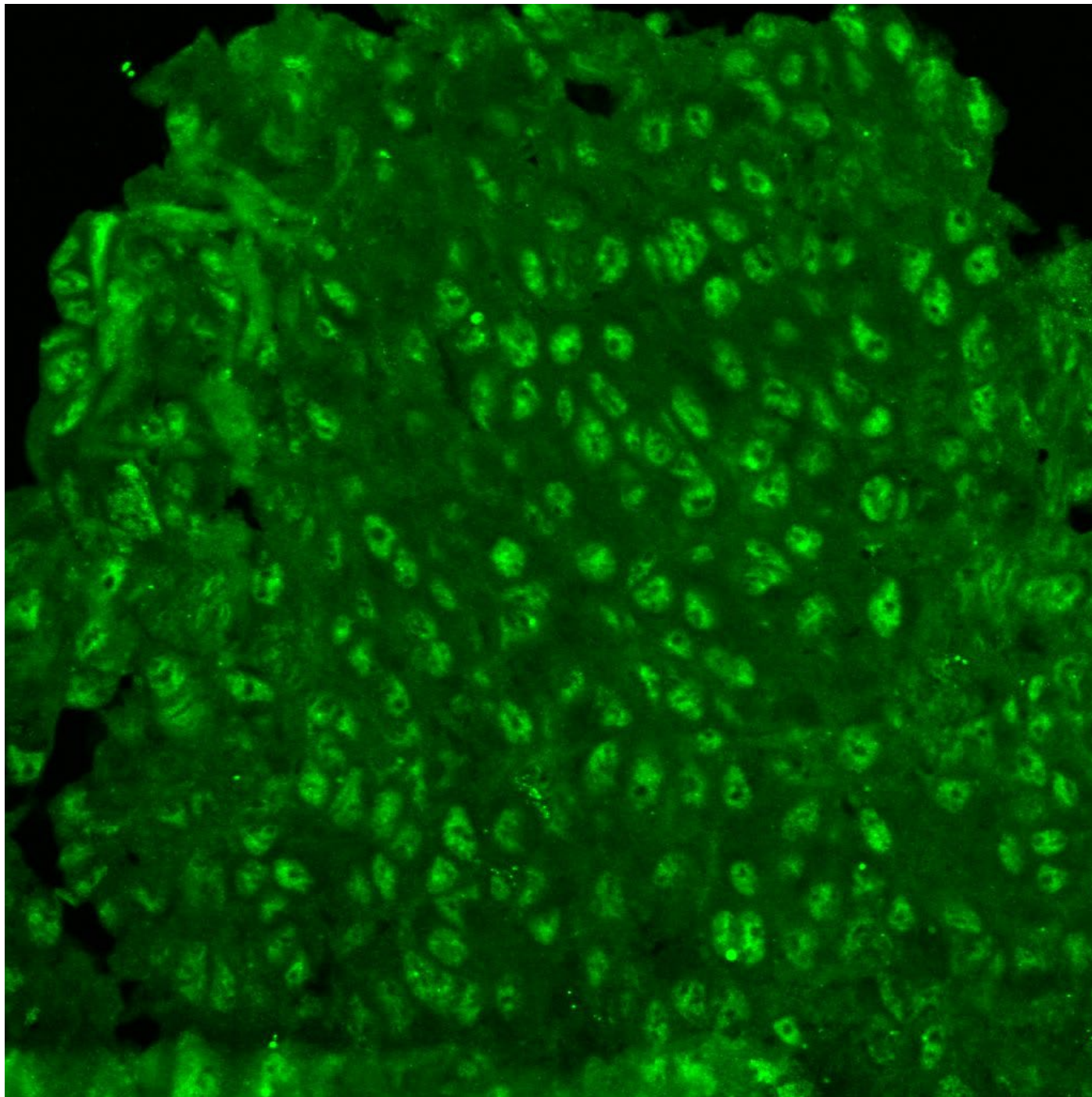

5212\_00

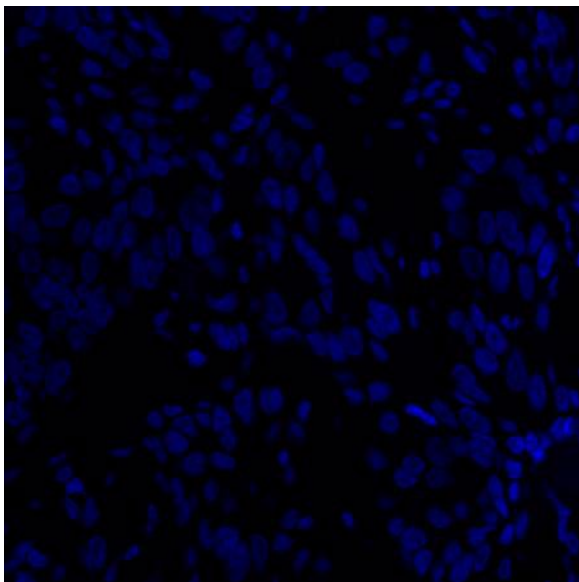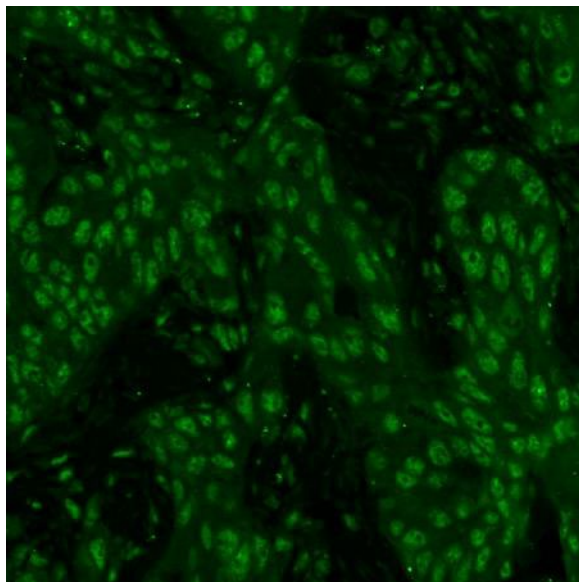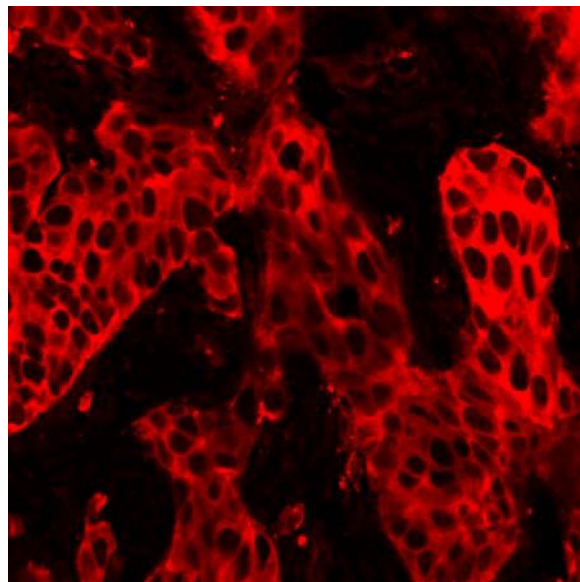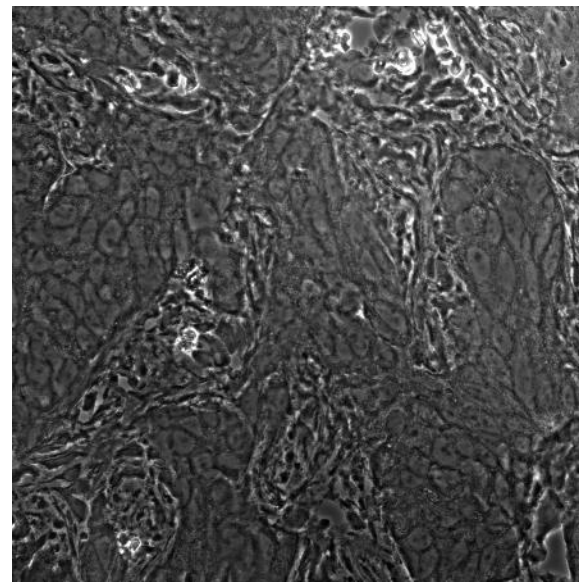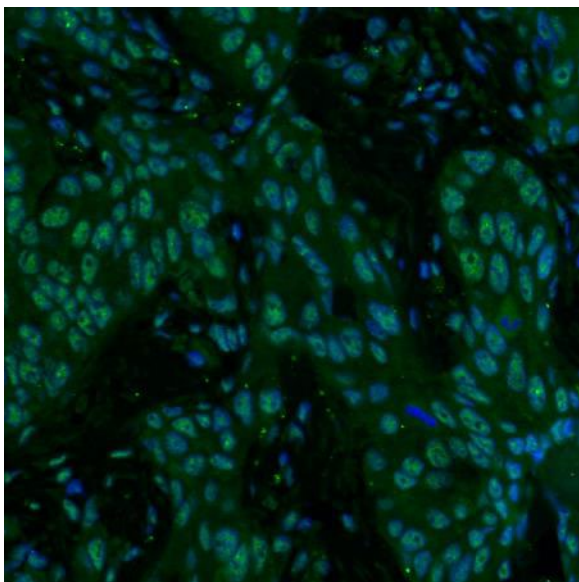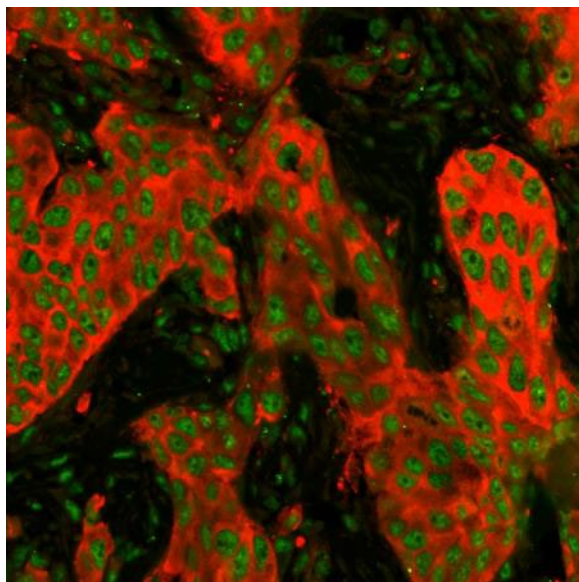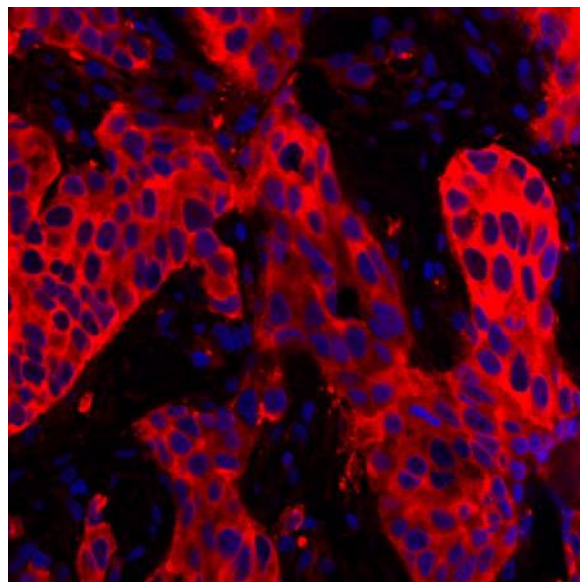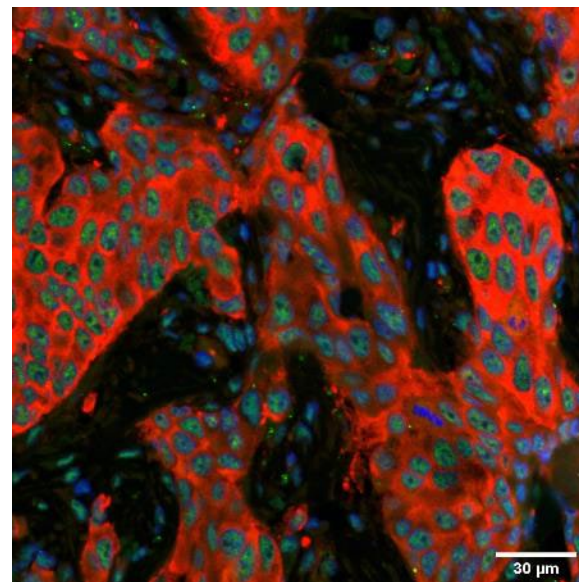

5212\_01

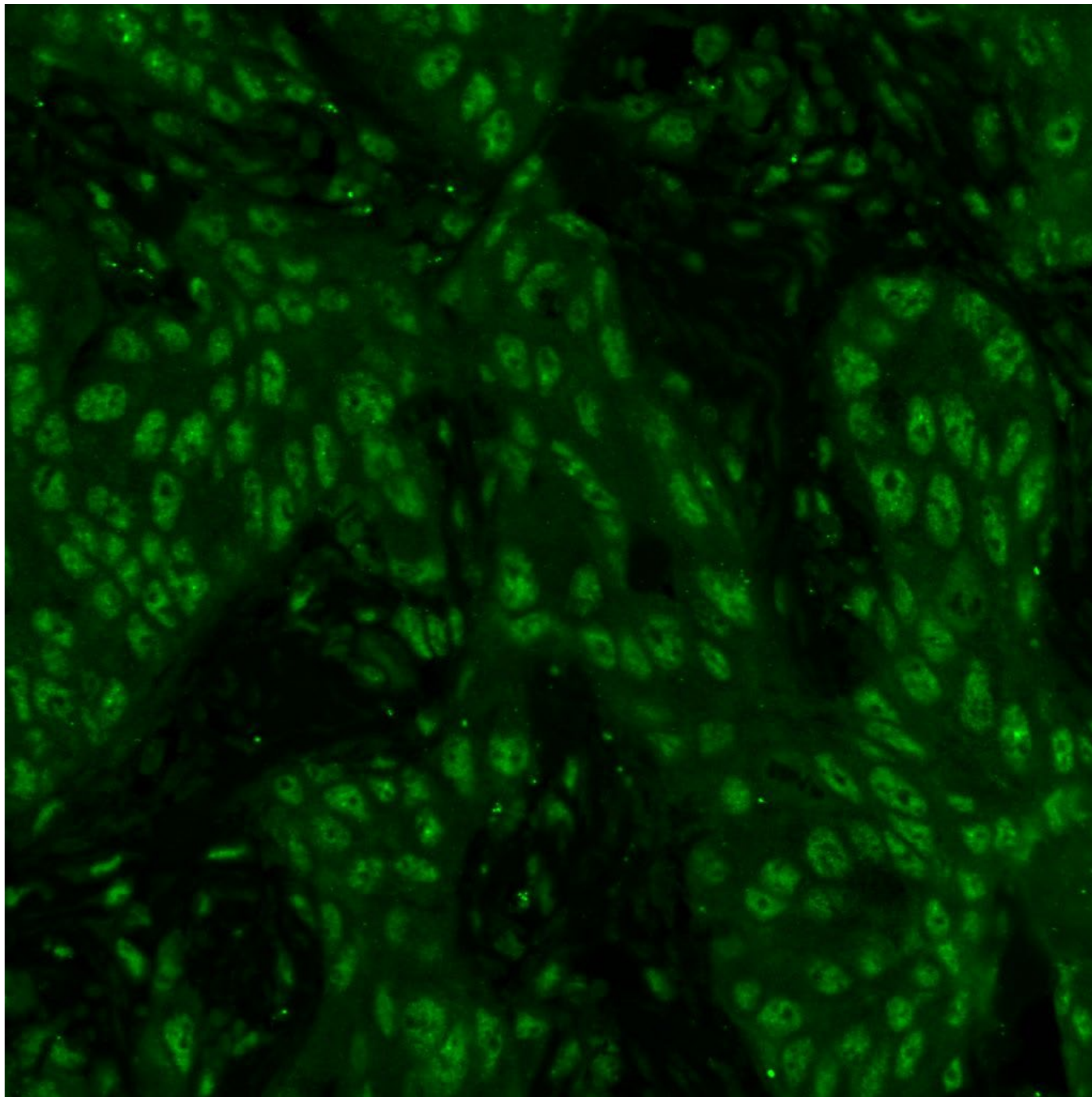

5212\_01

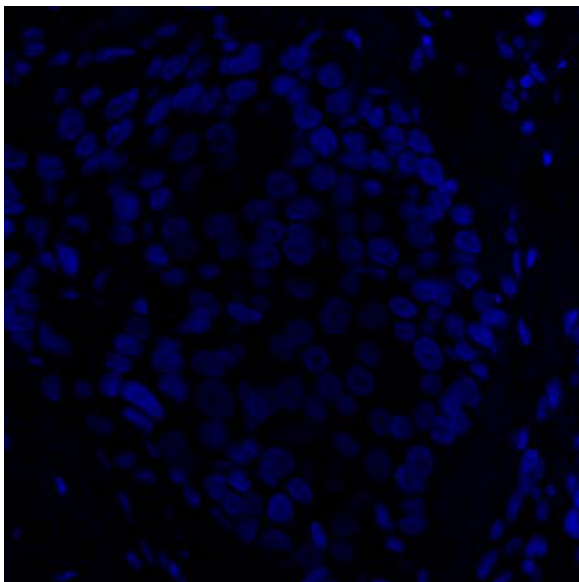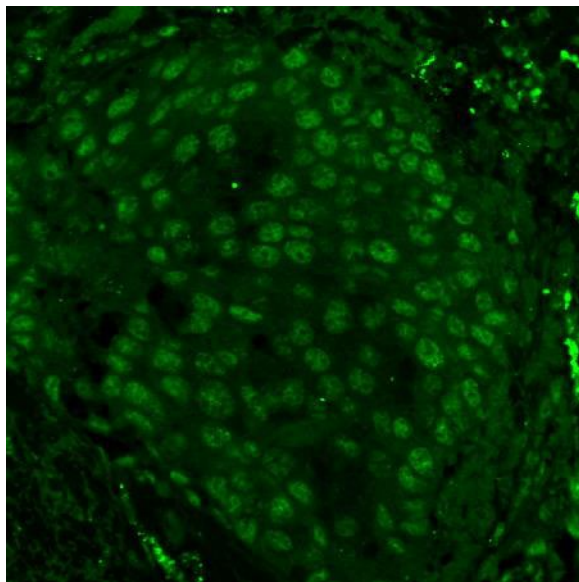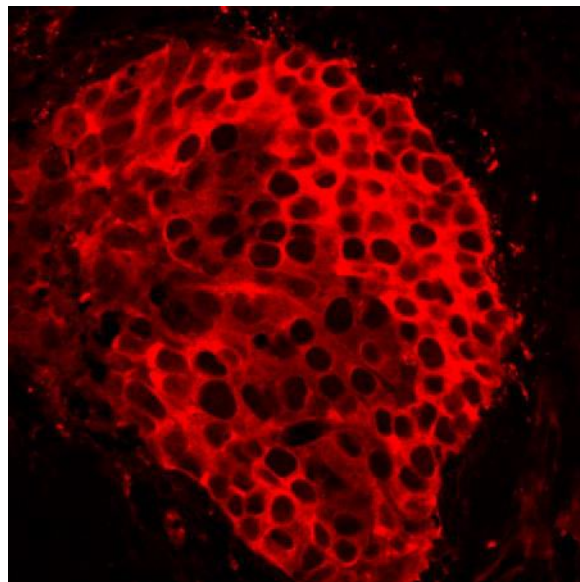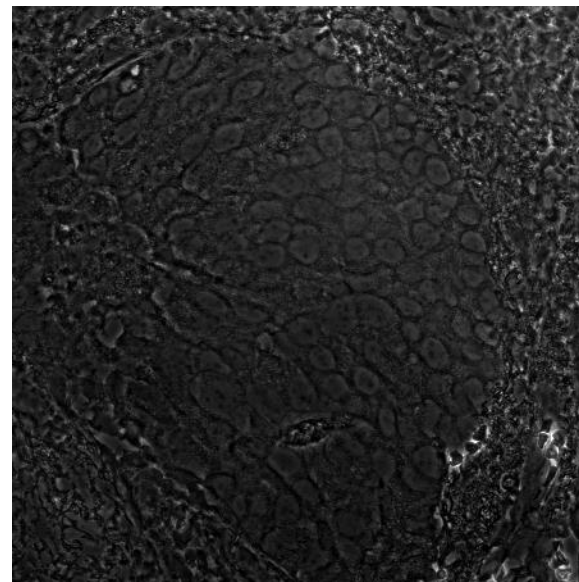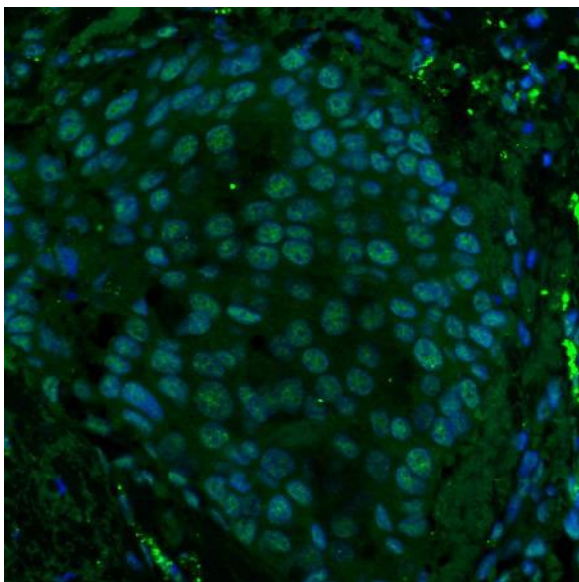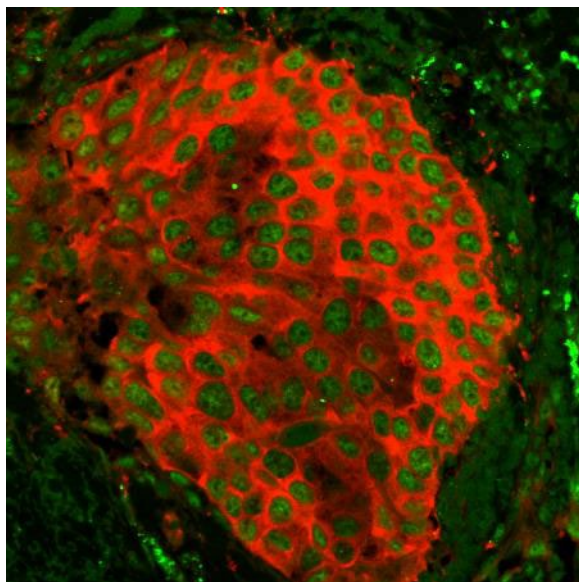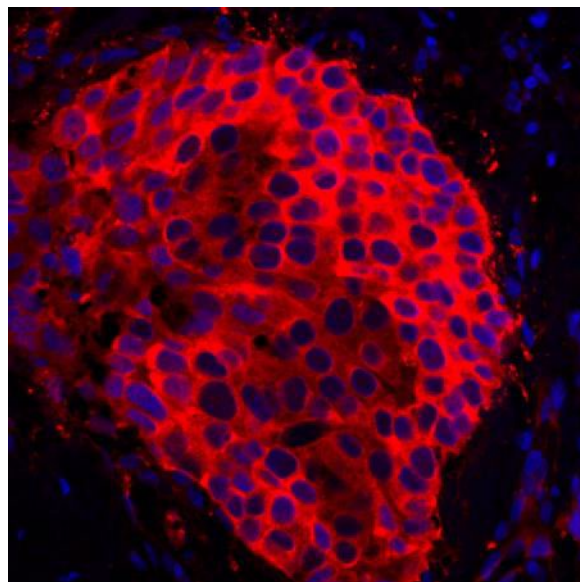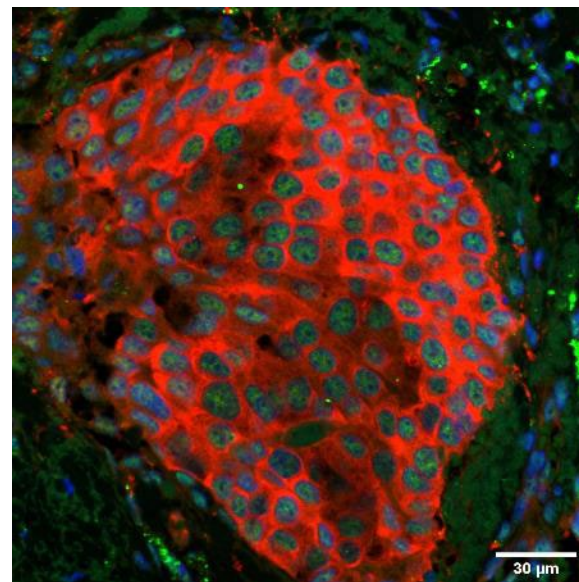

5212\_02

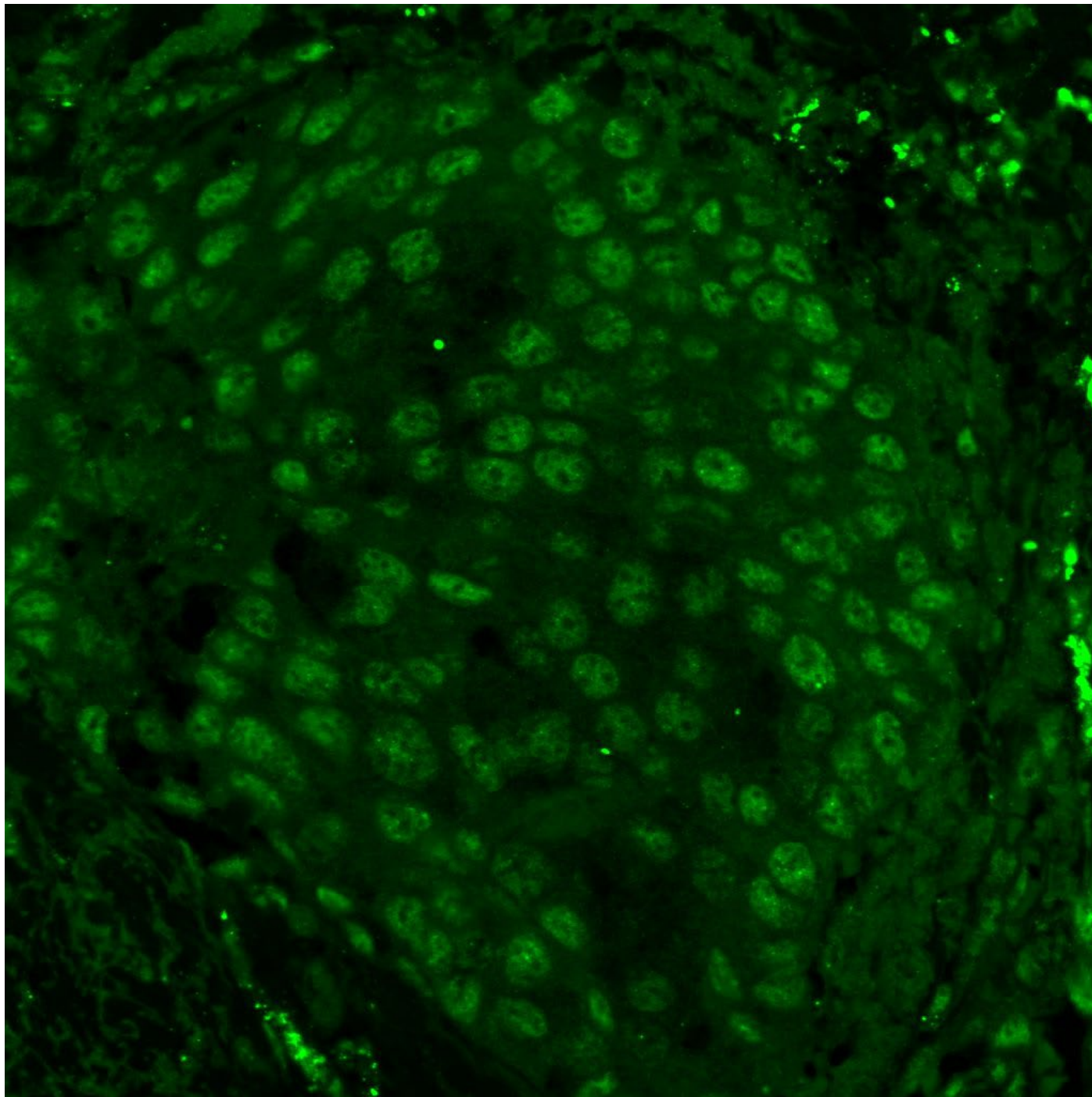

5212\_02

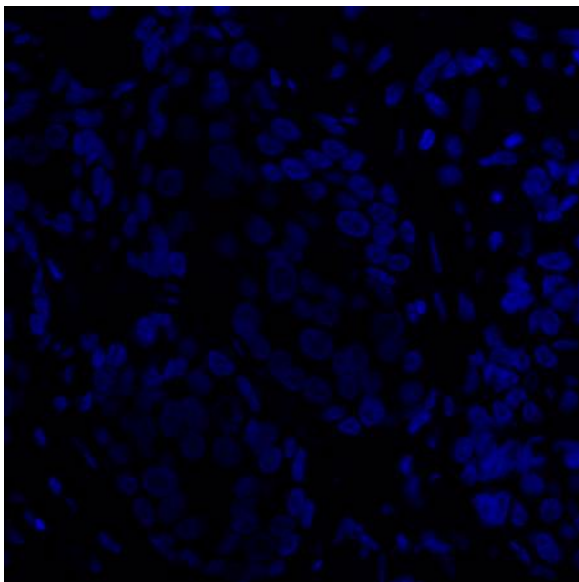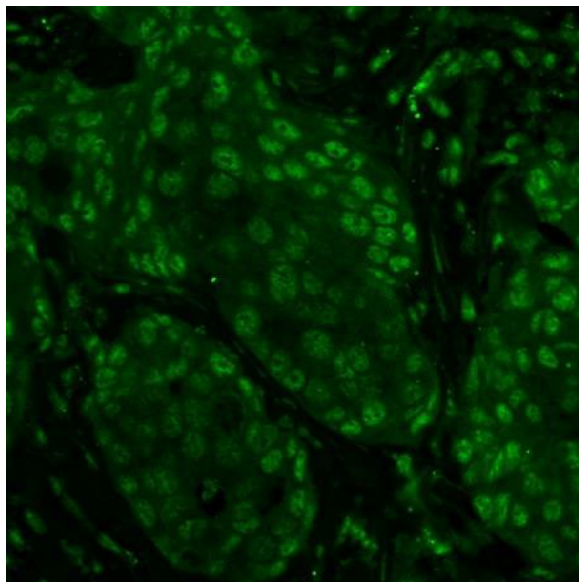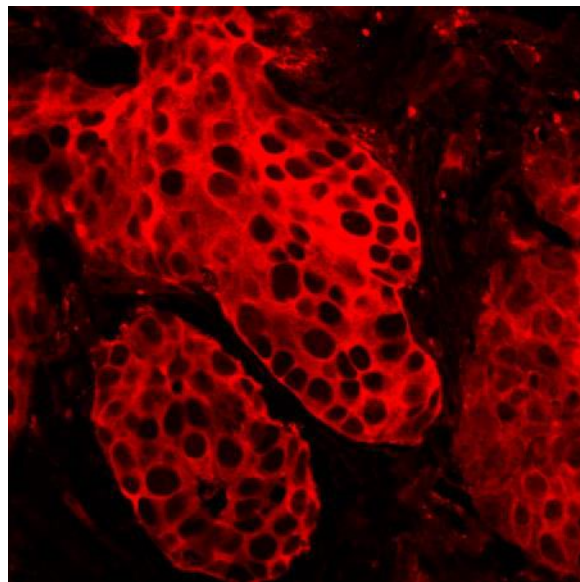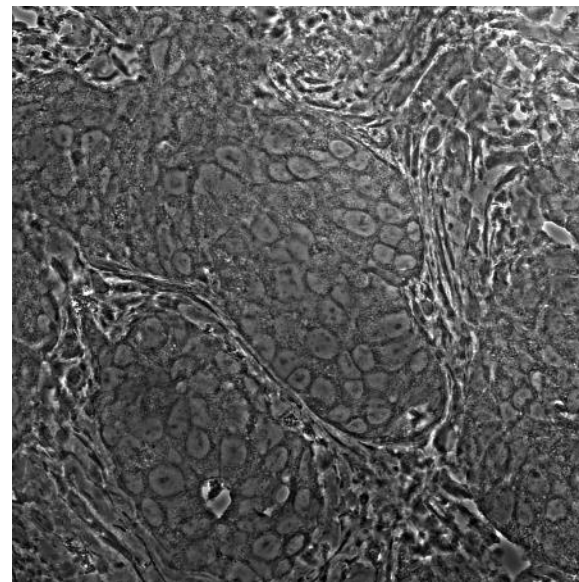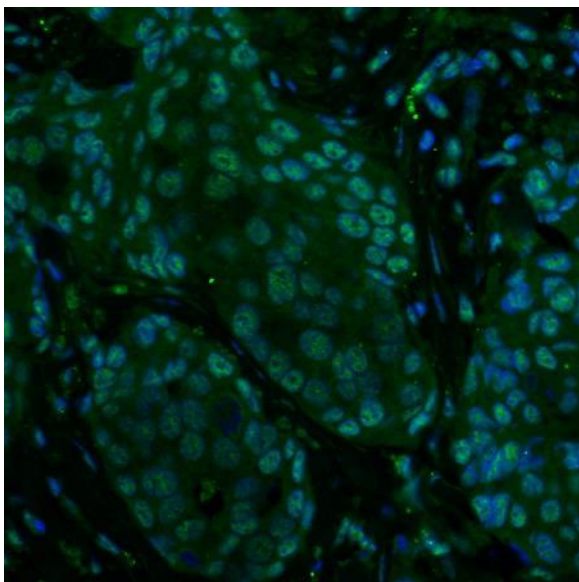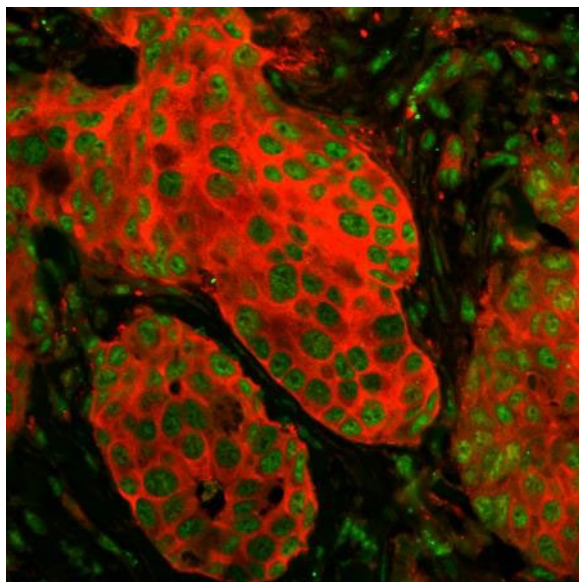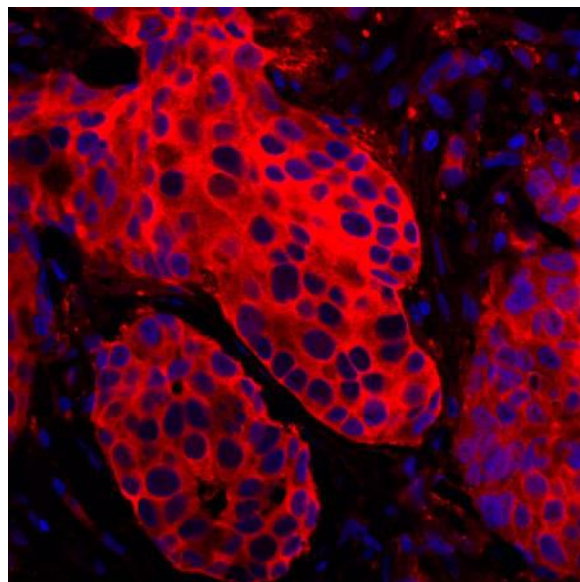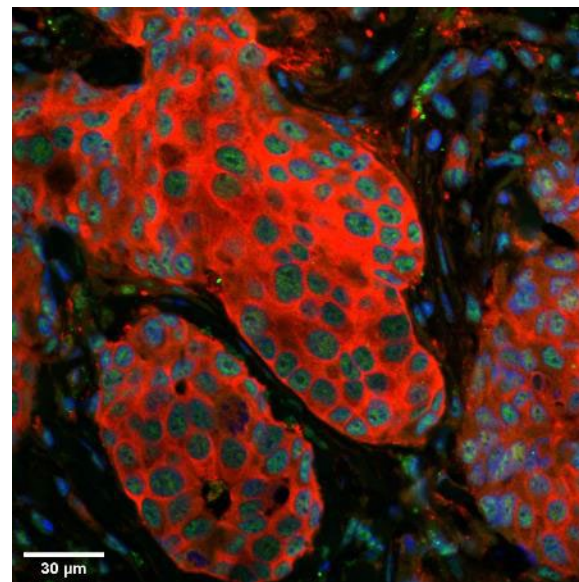

5212\_03

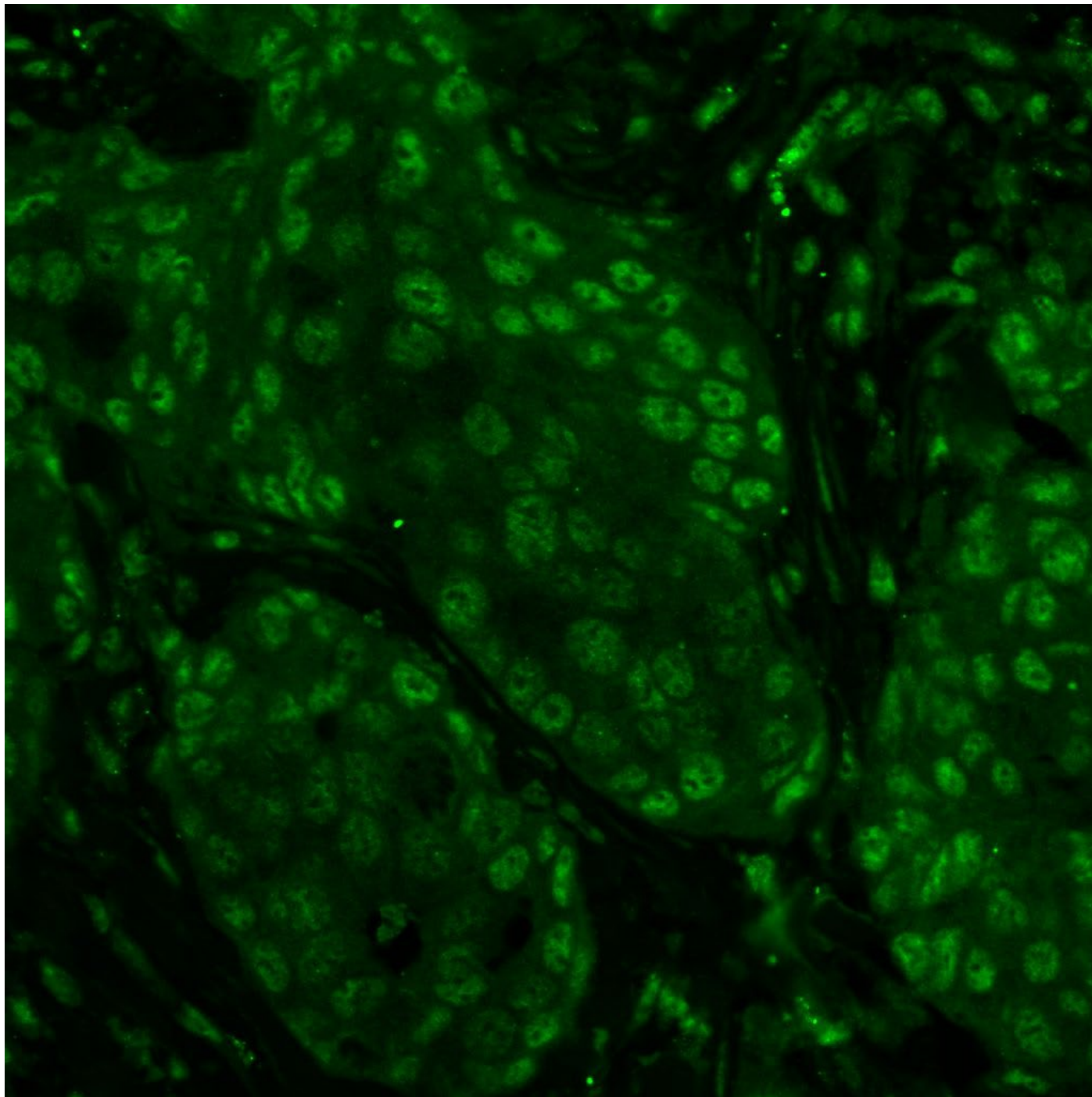

5212\_3

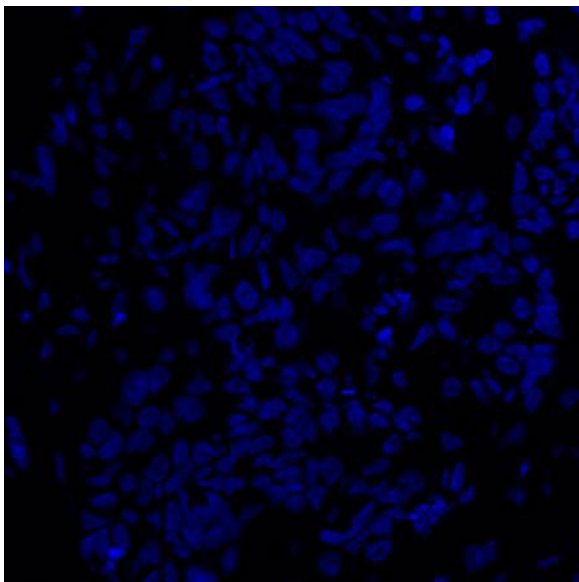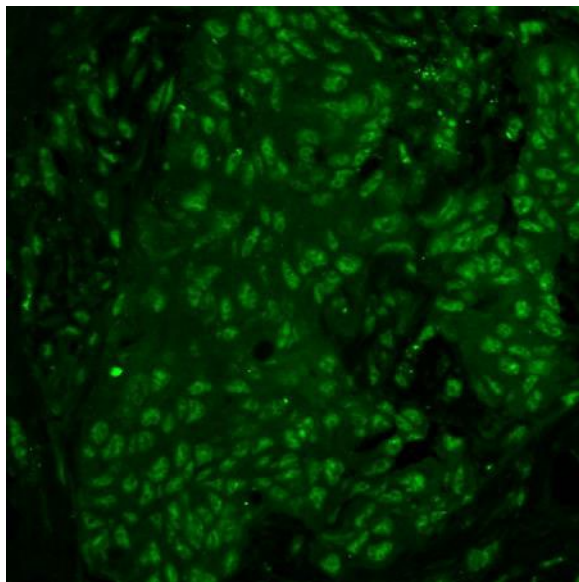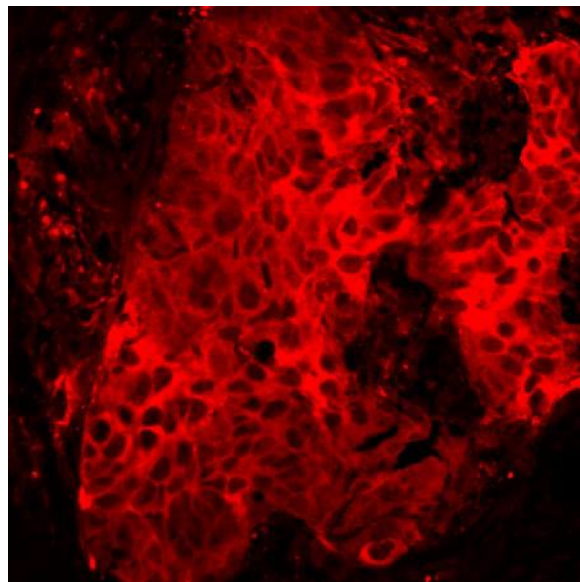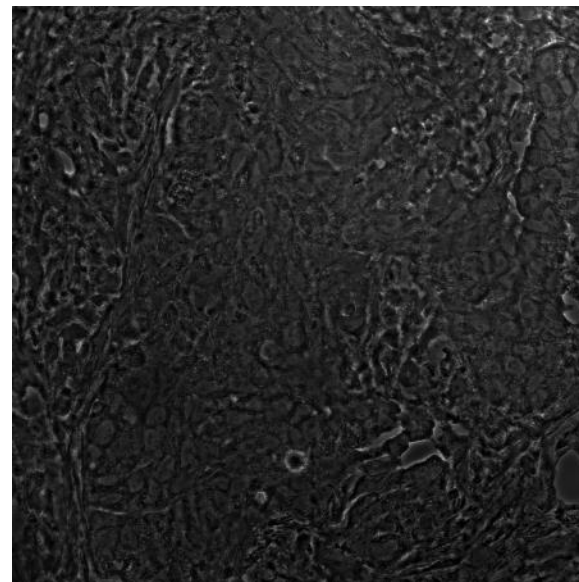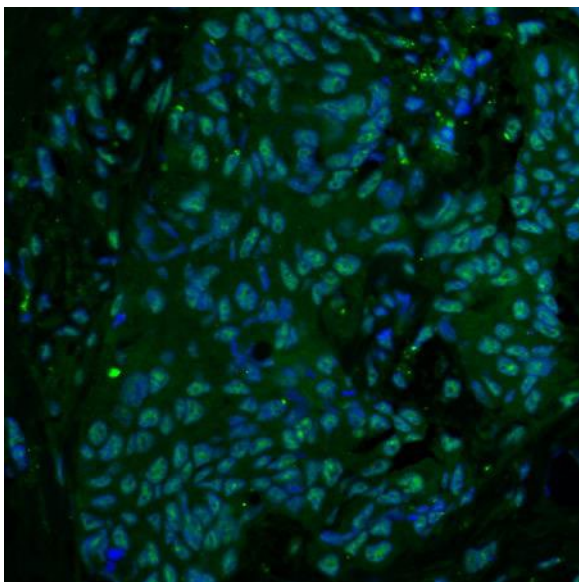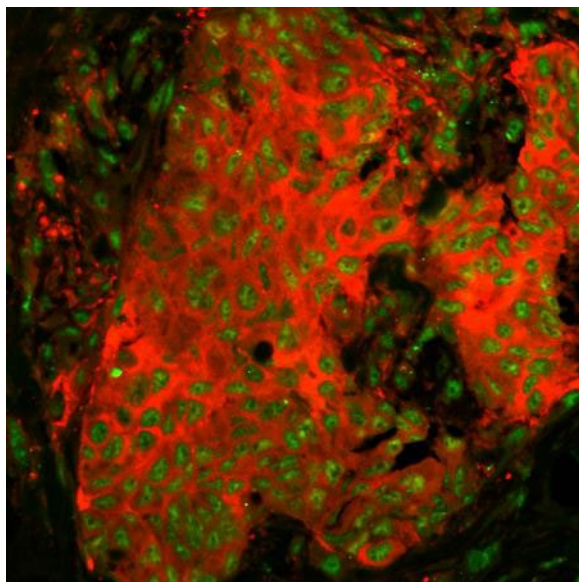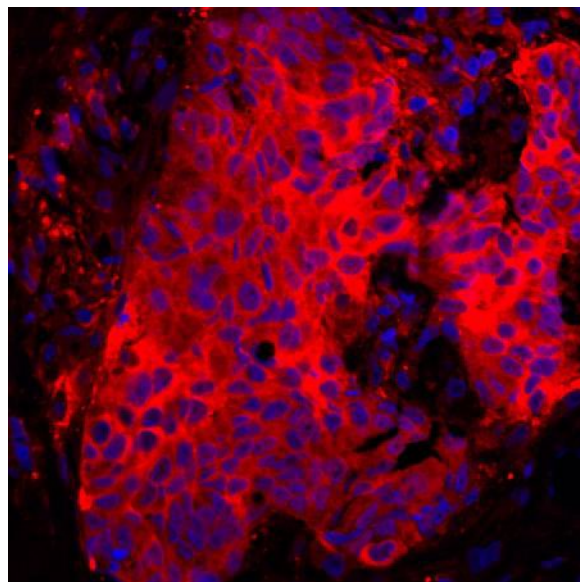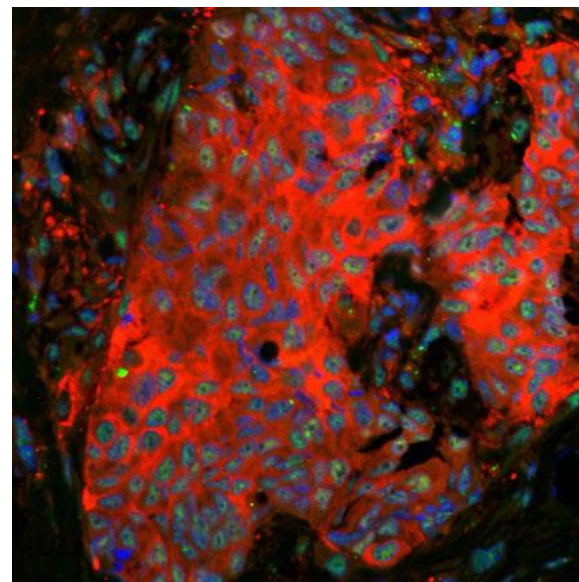

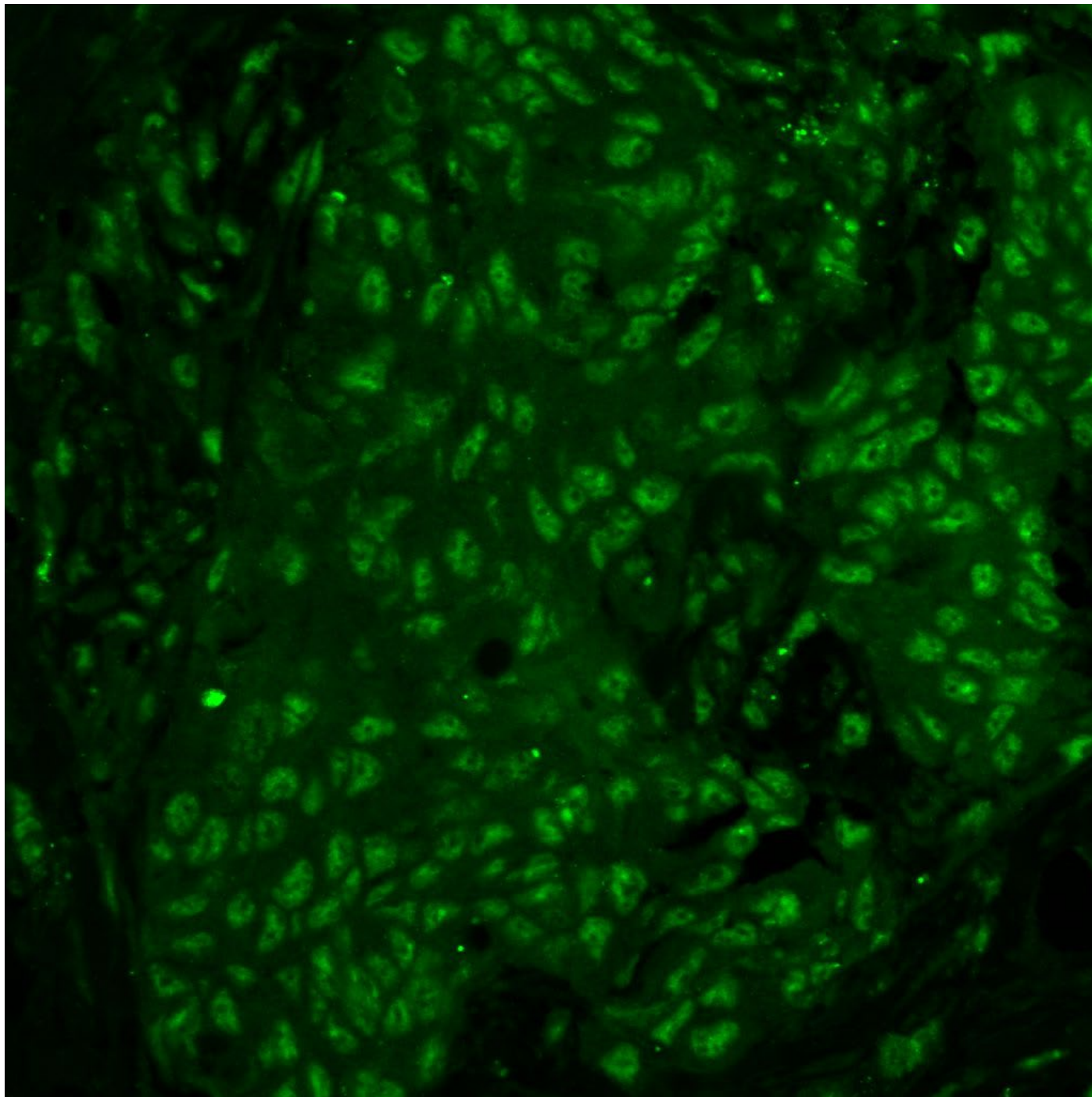

5212\_3

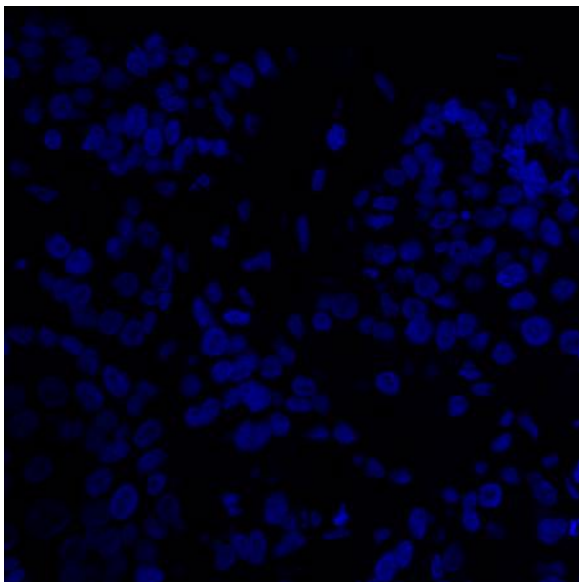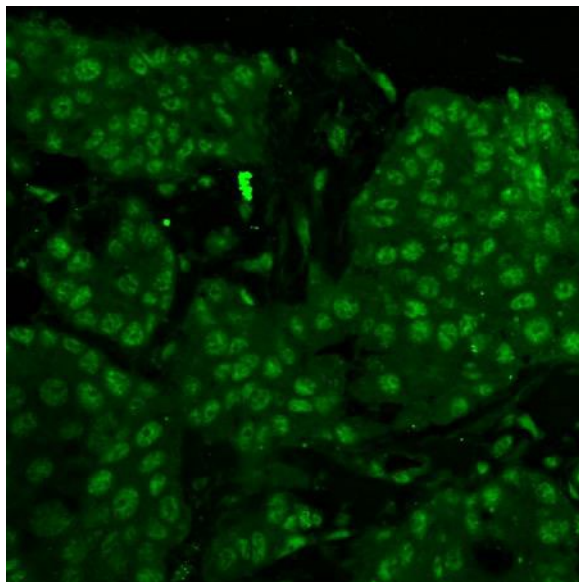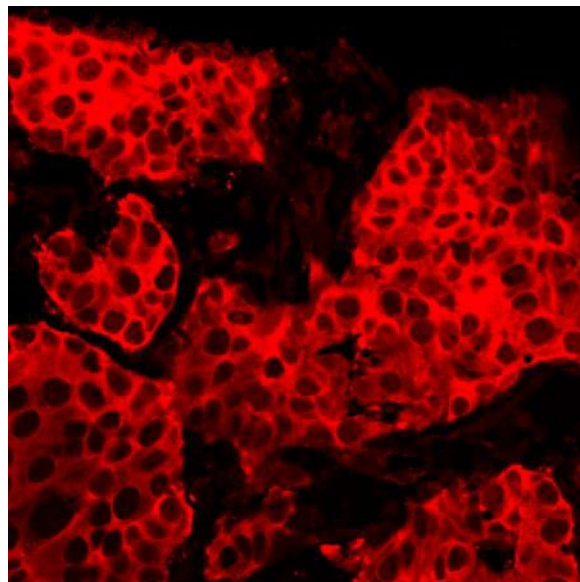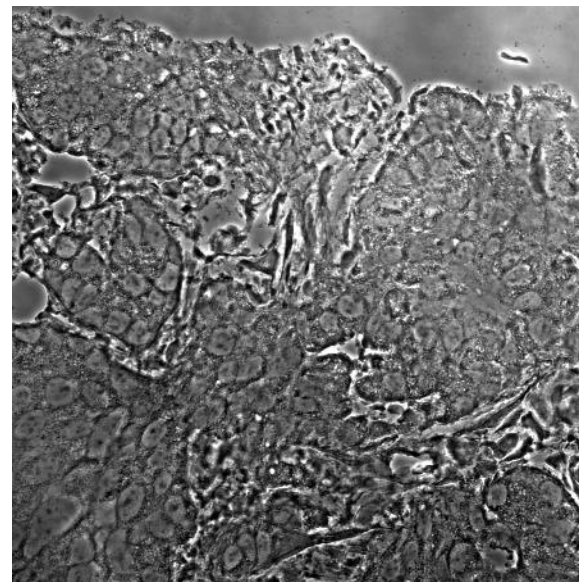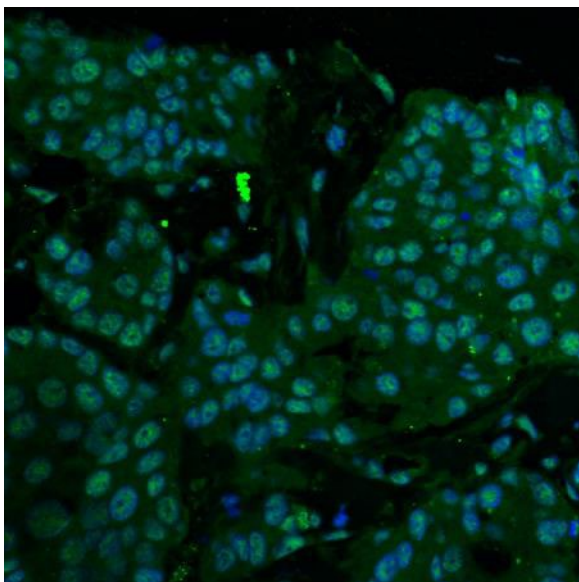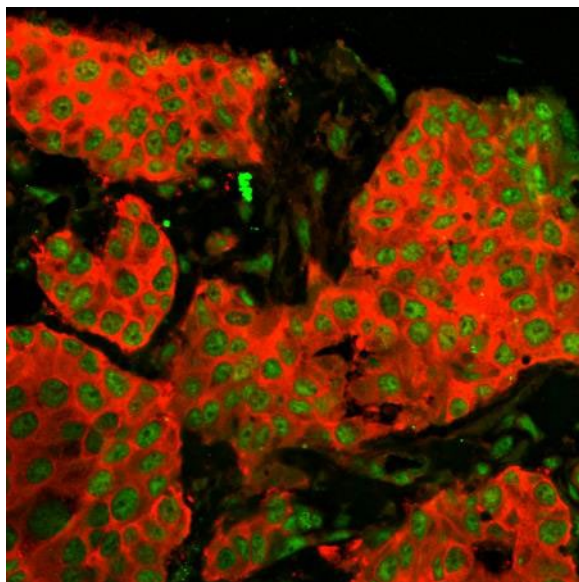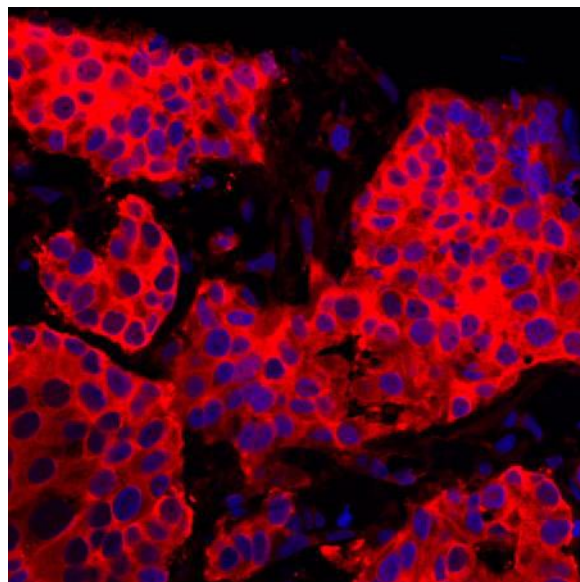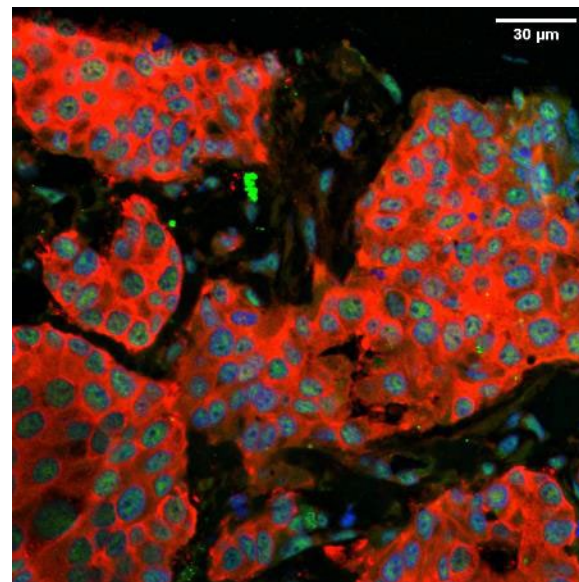

5212\_05

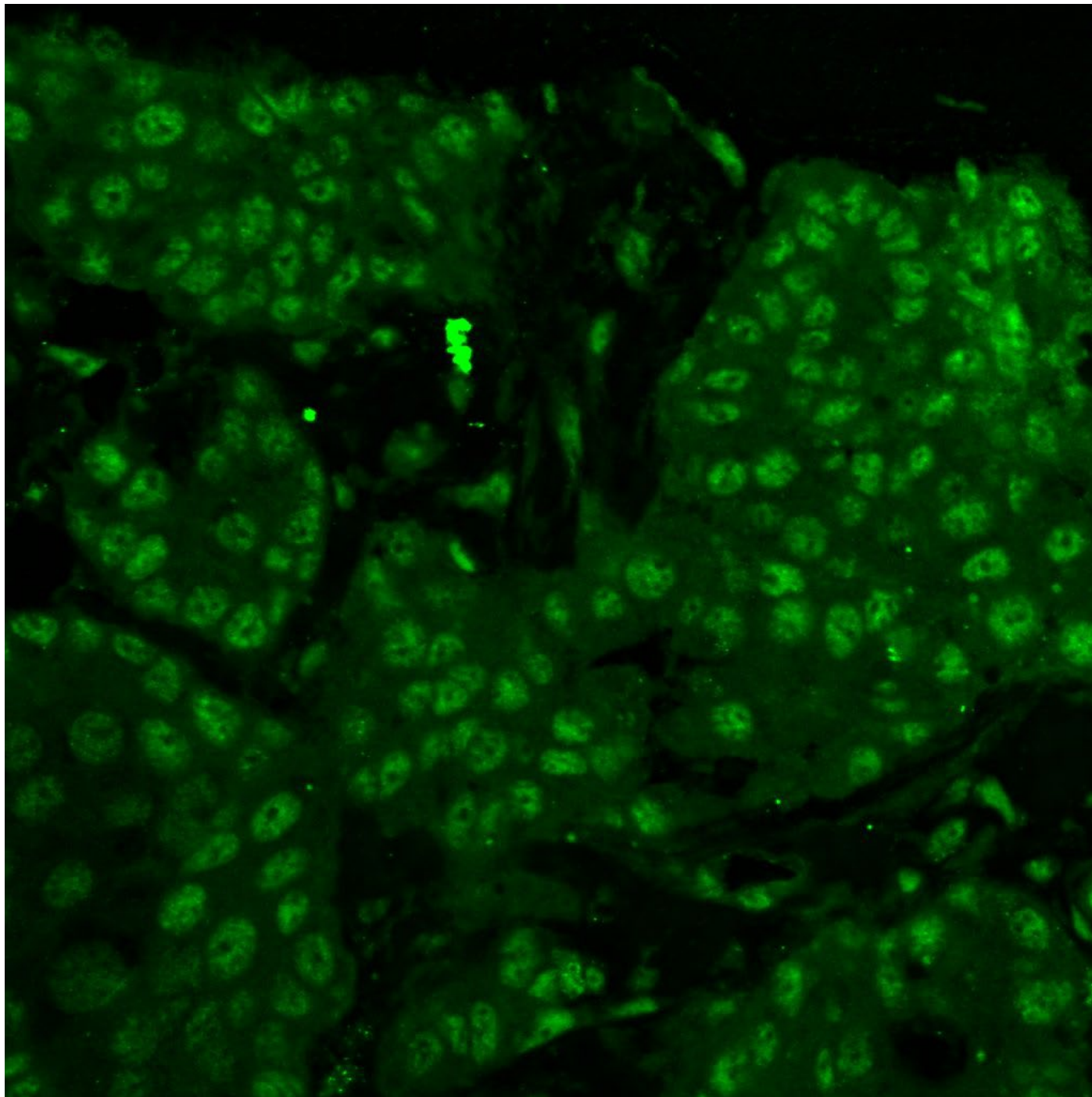

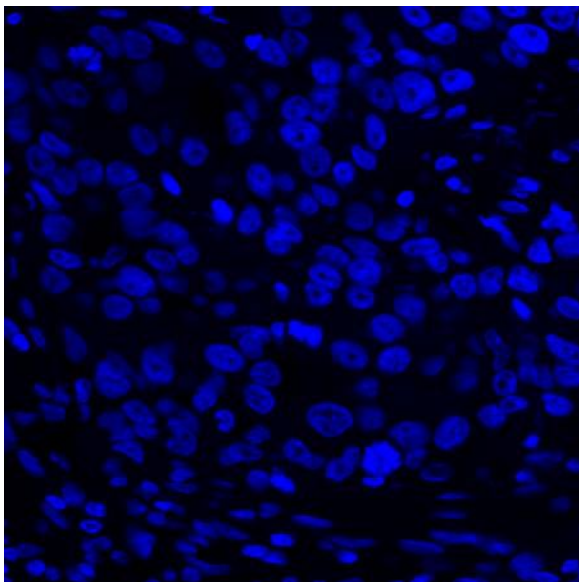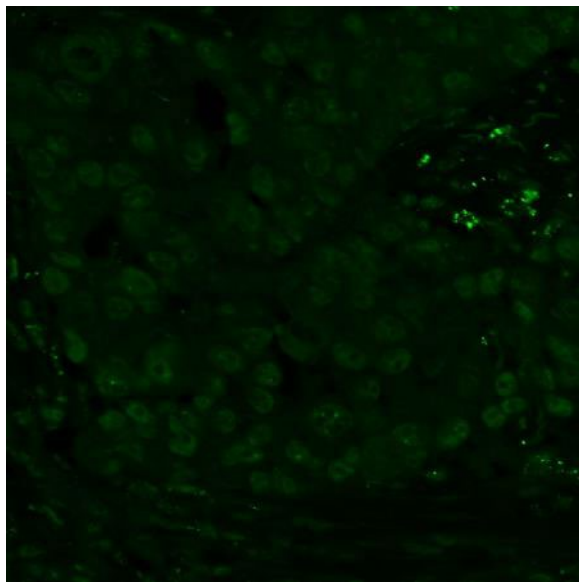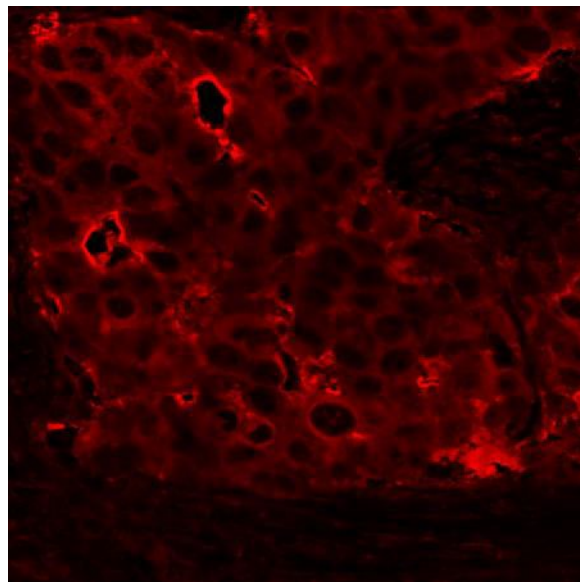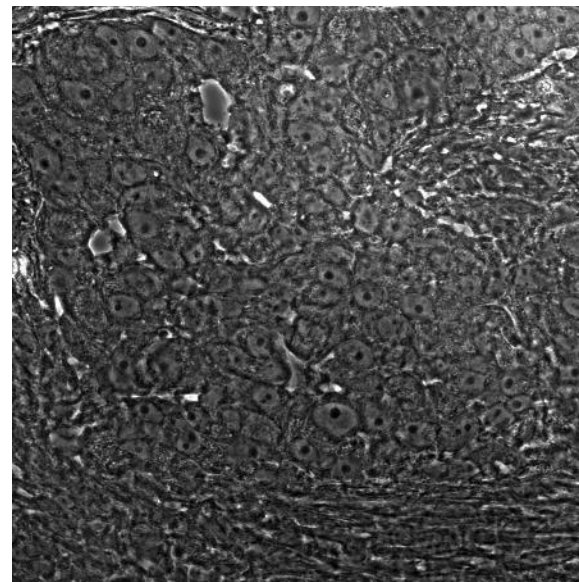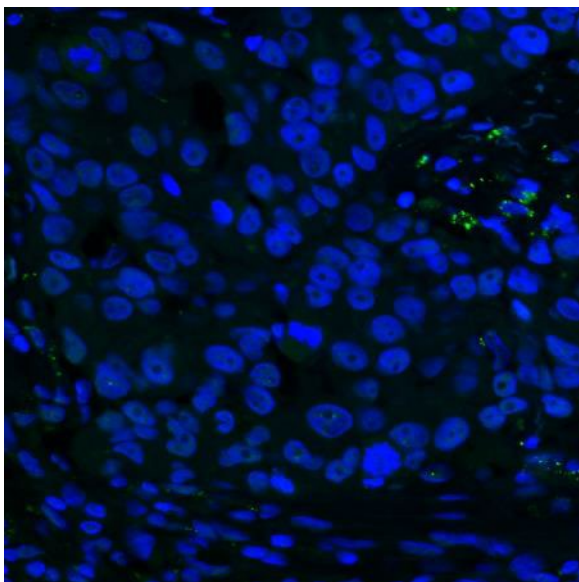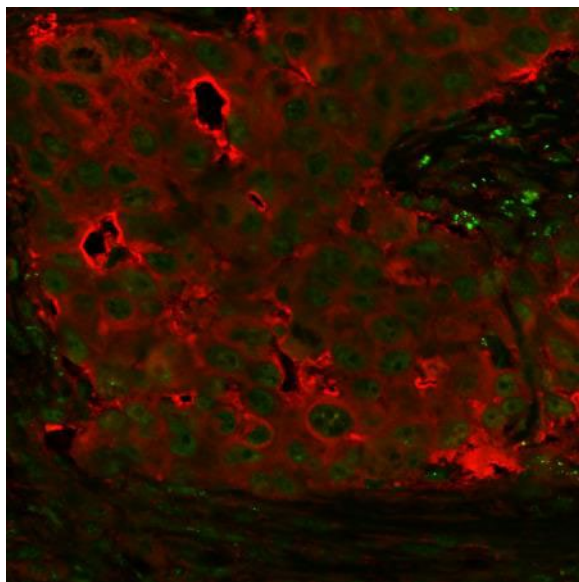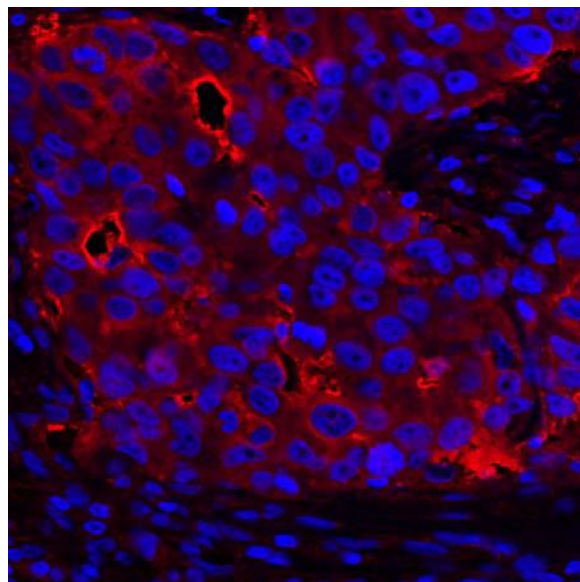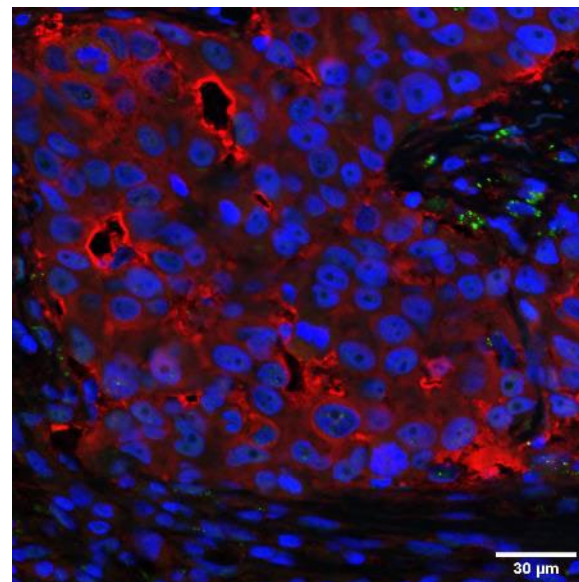

5789\_00

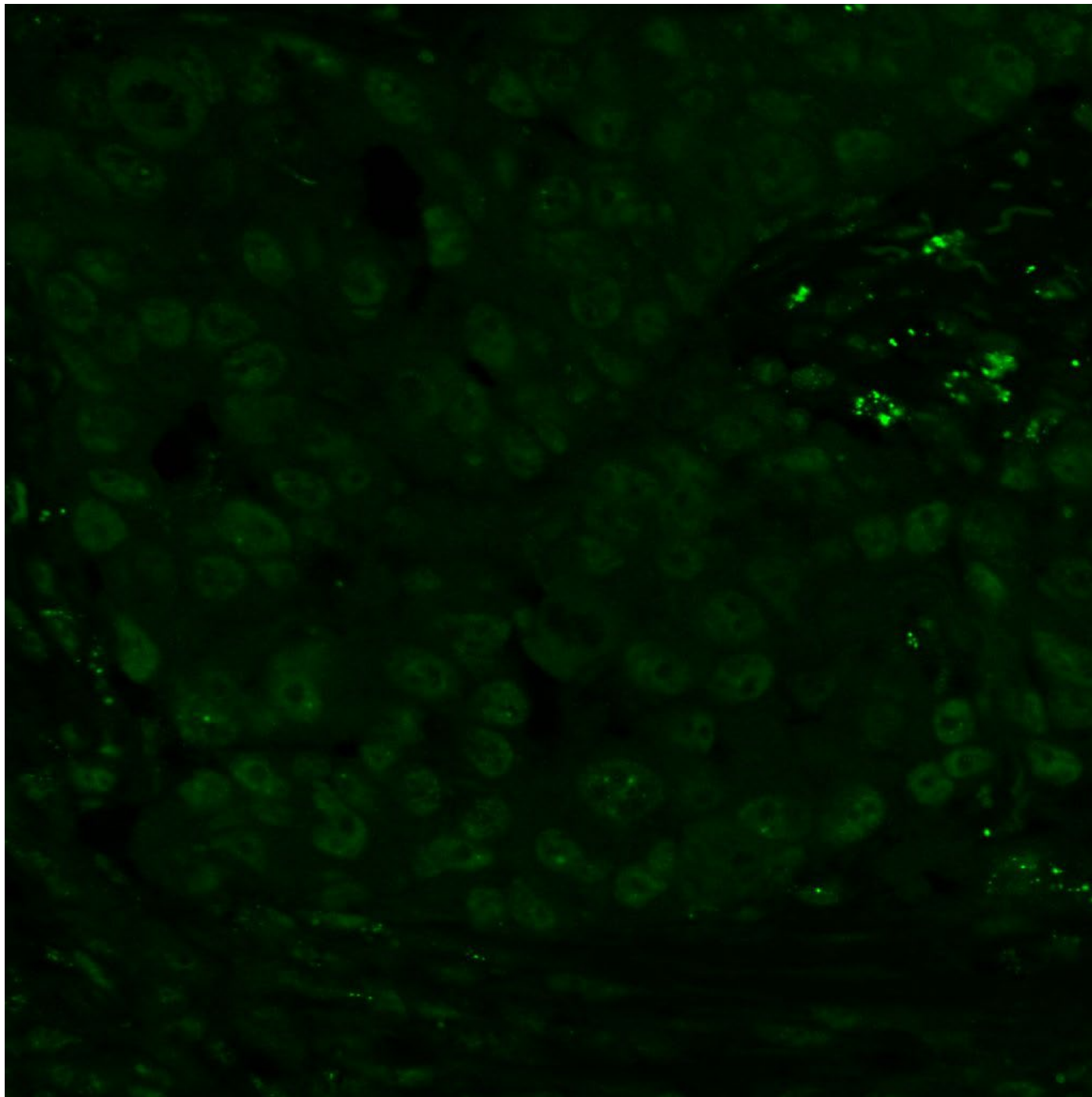

5789\_00

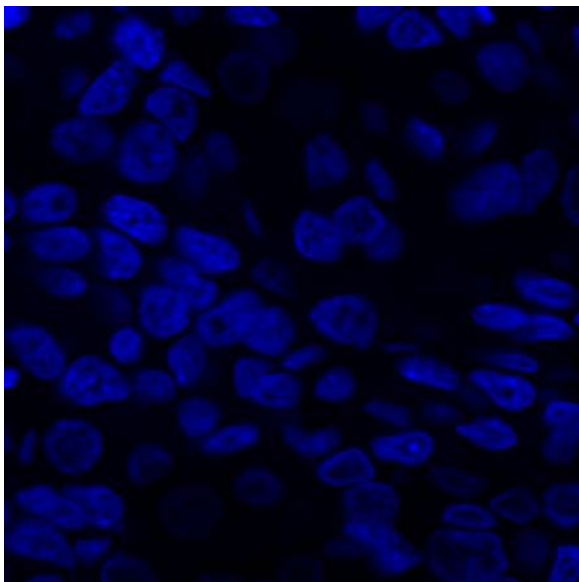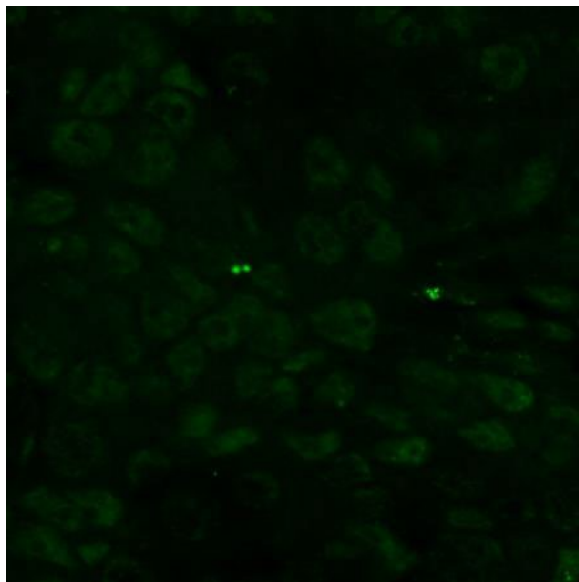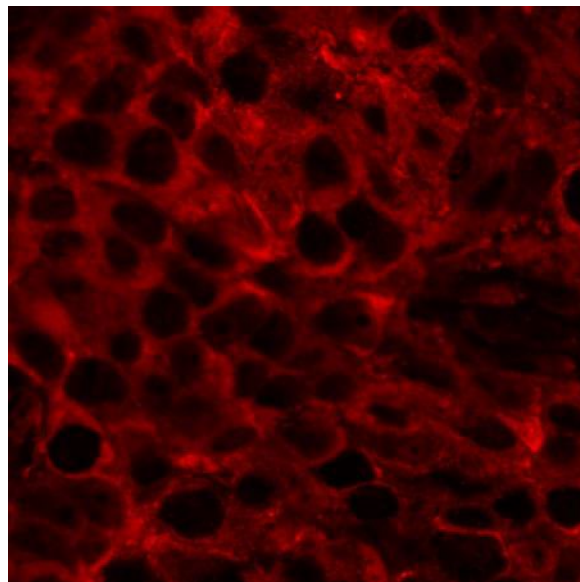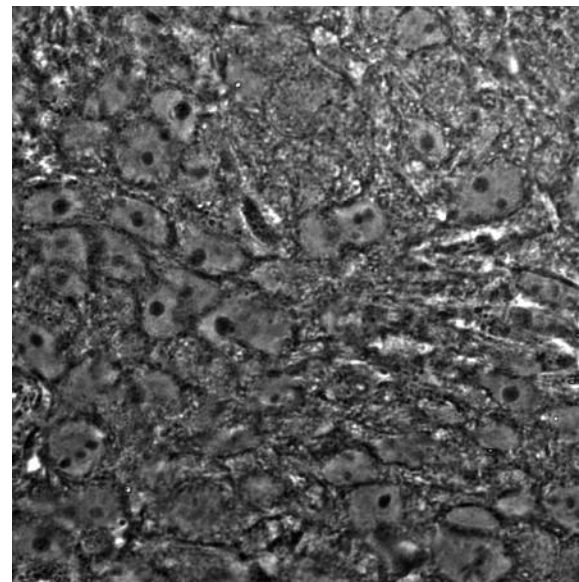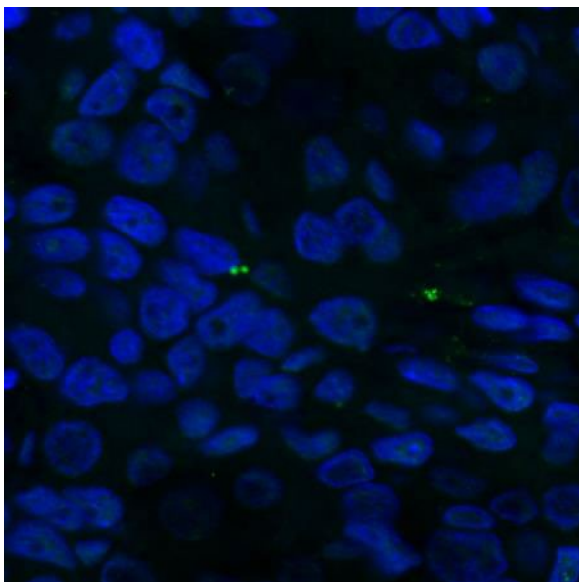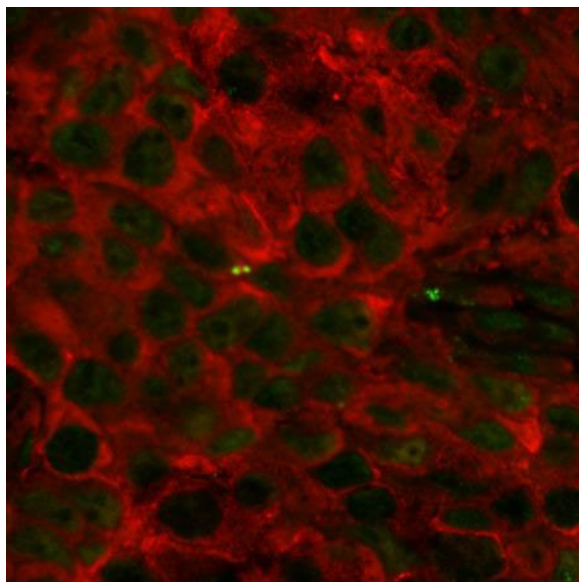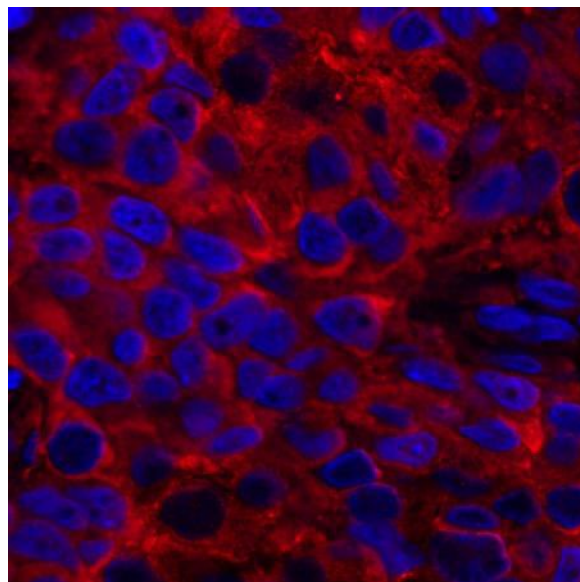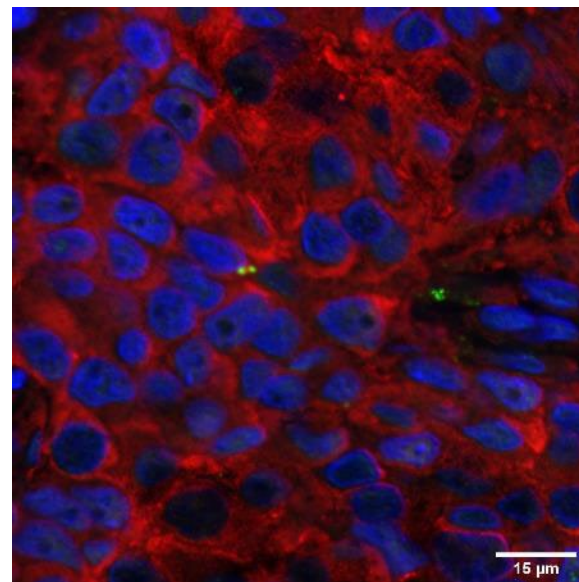

5789\_01

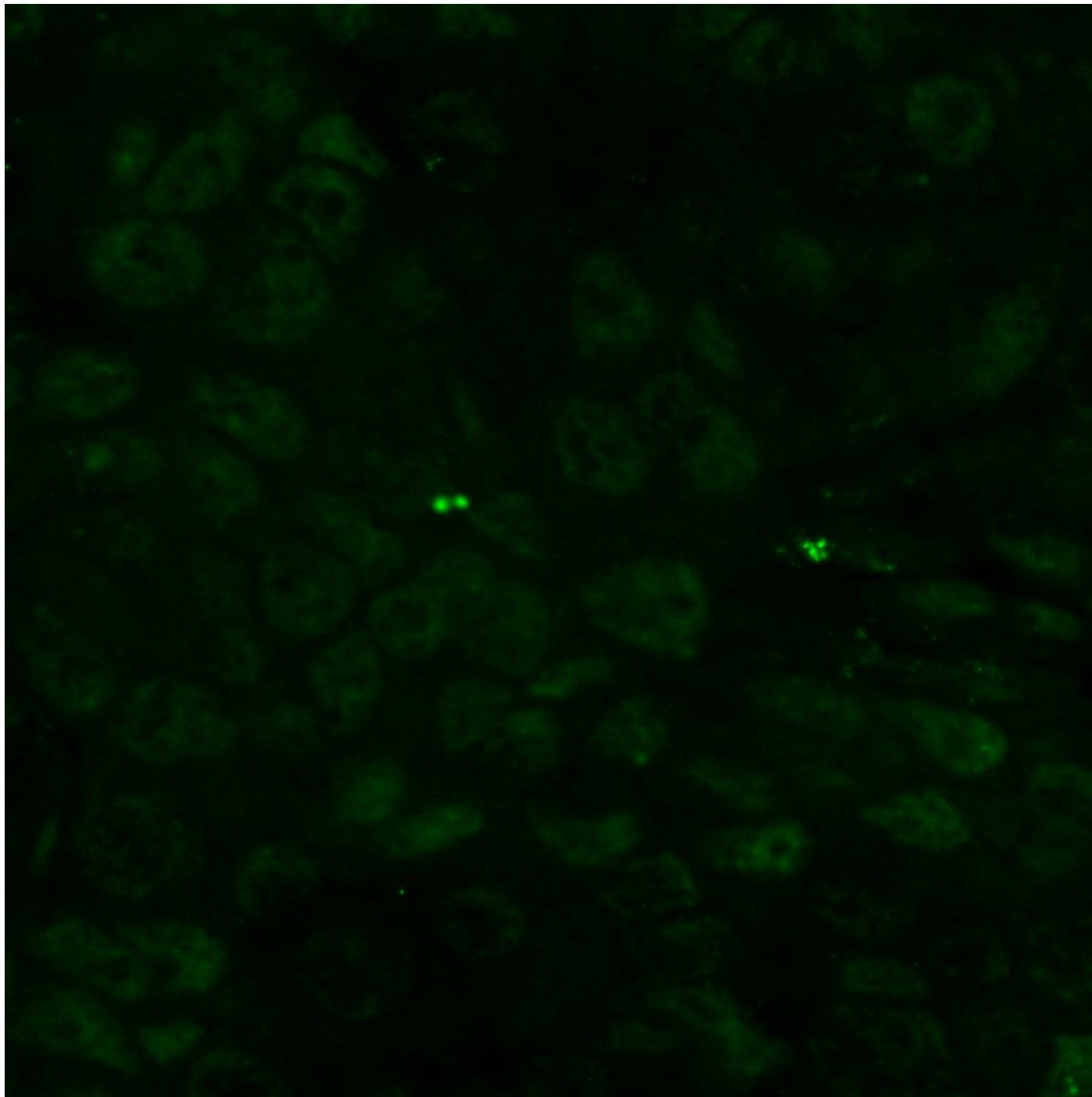

5789\_01

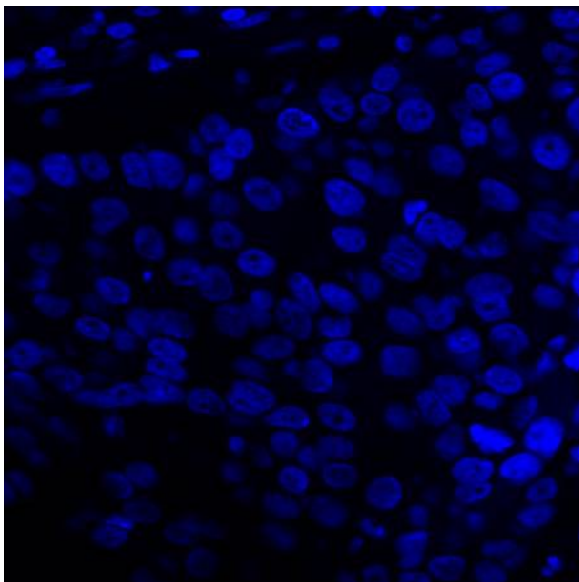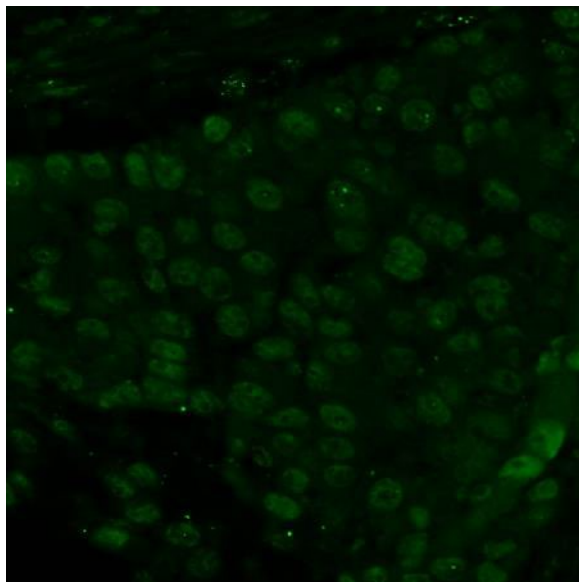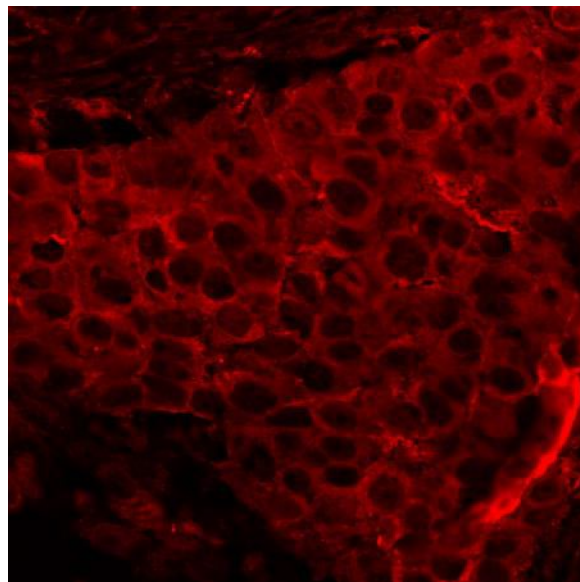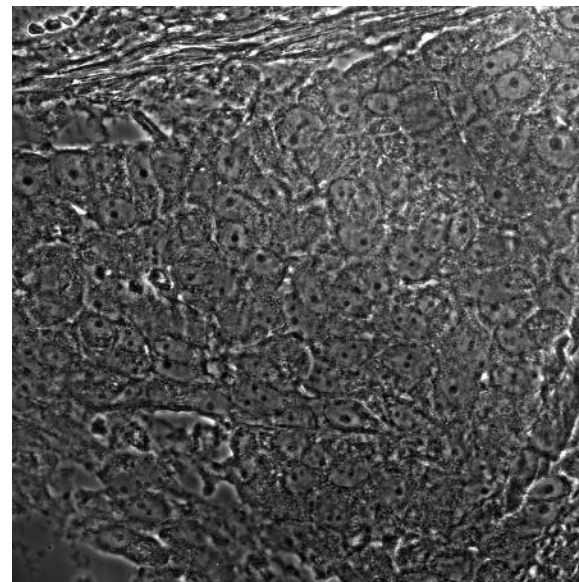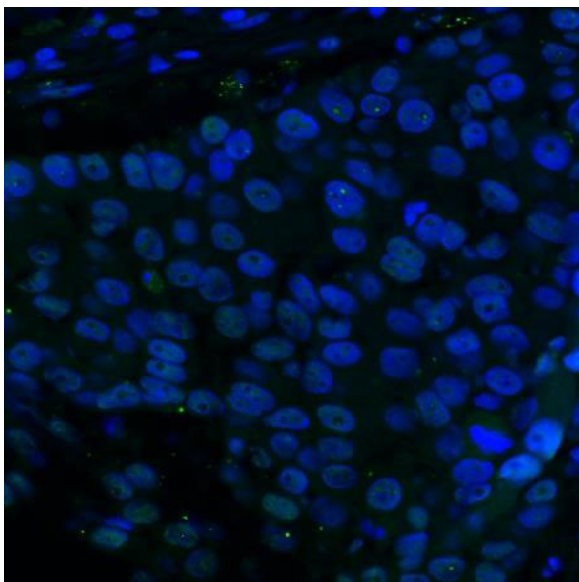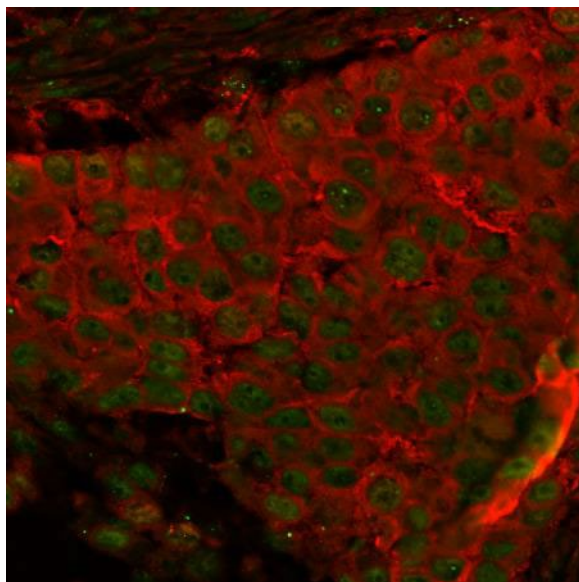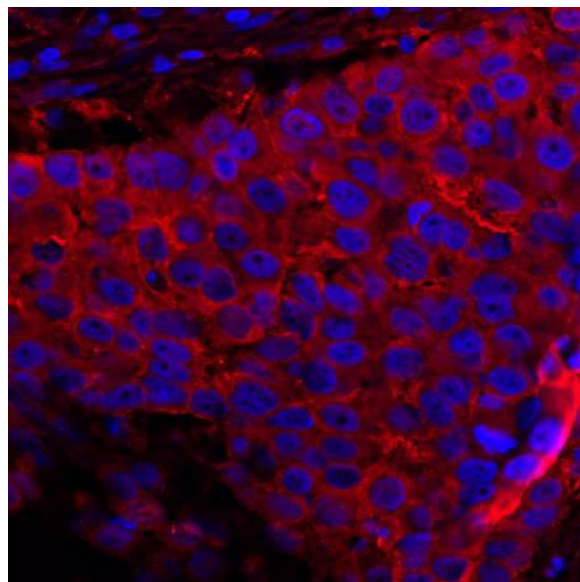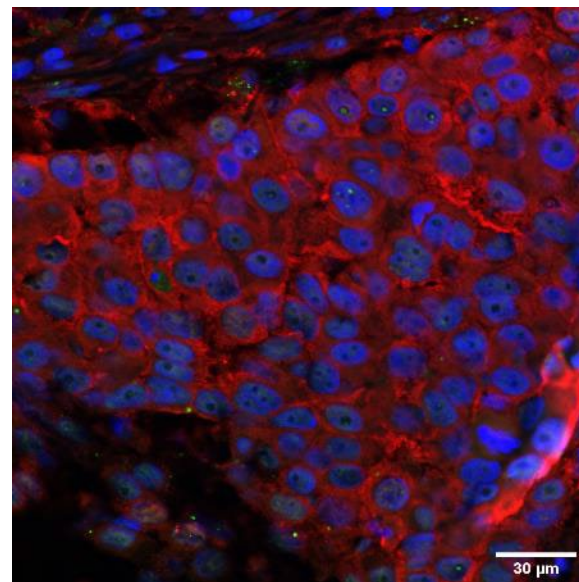

5789\_02

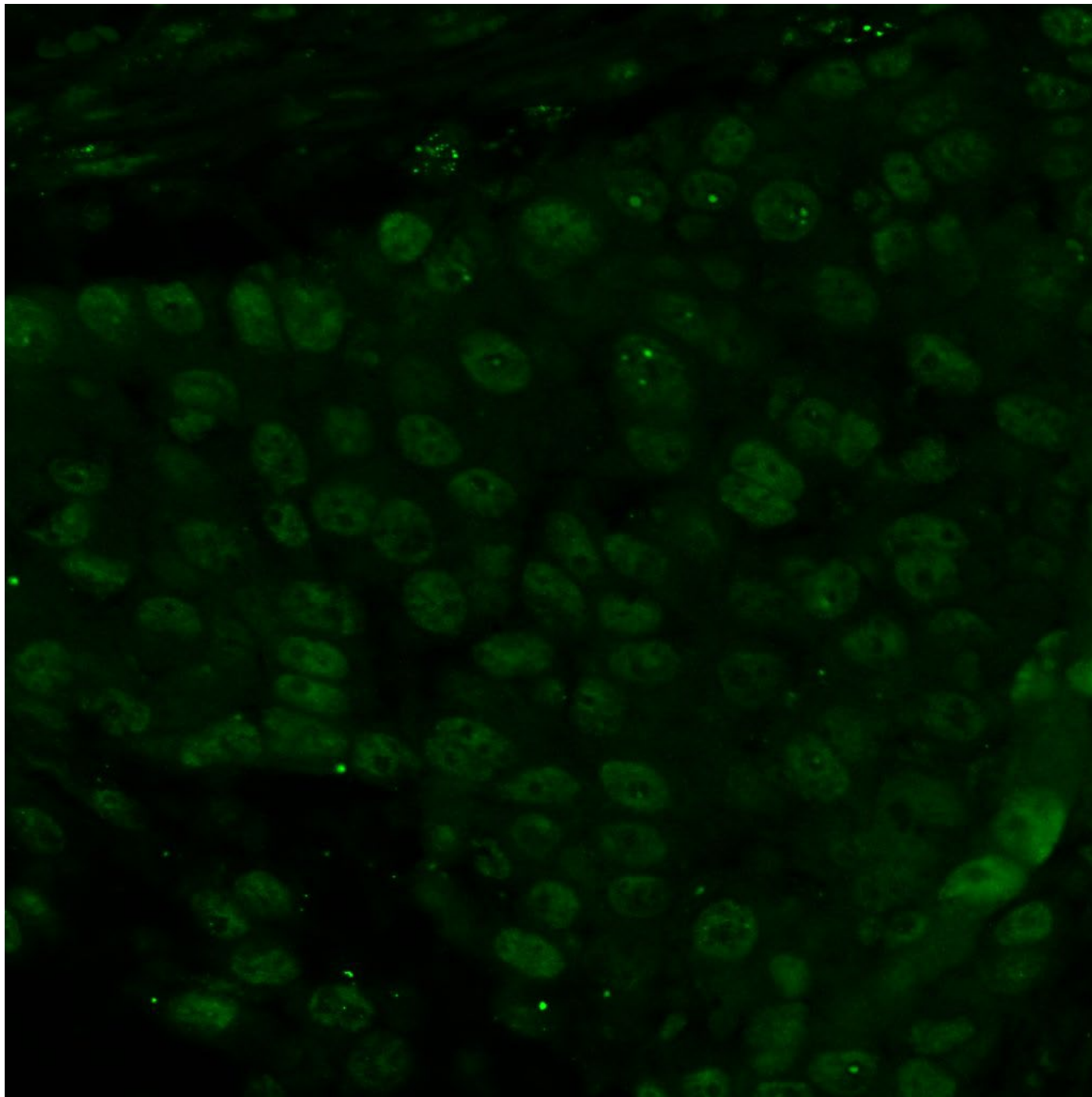

5789\_02

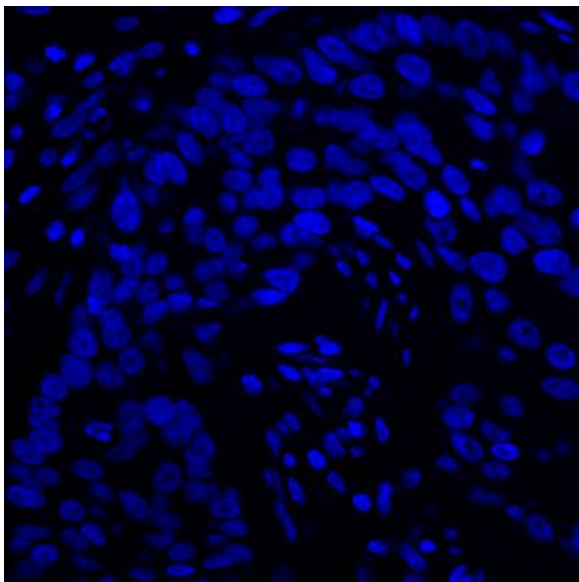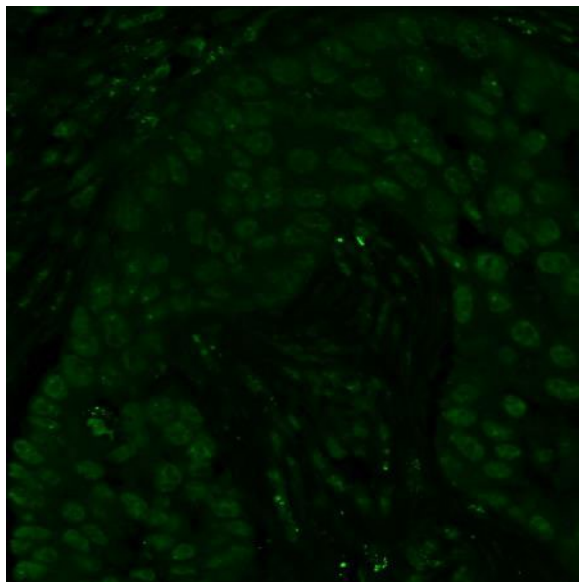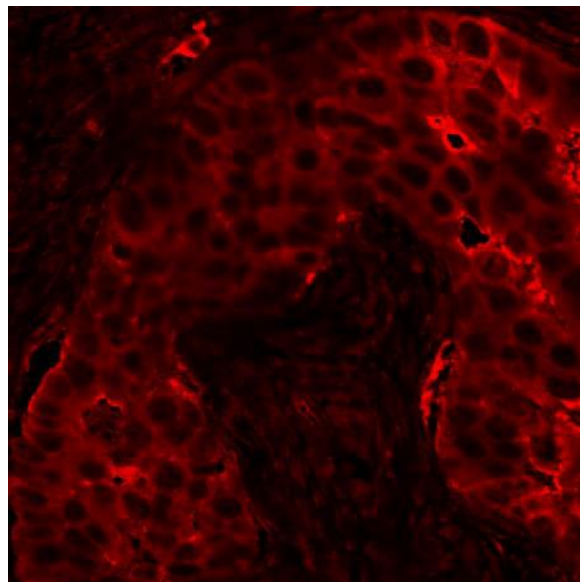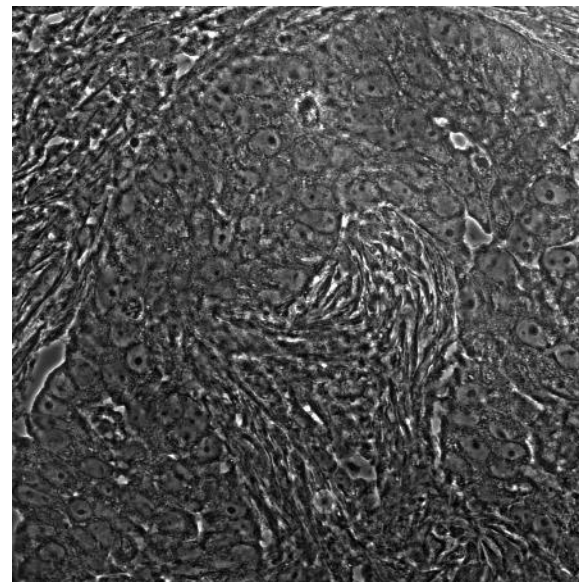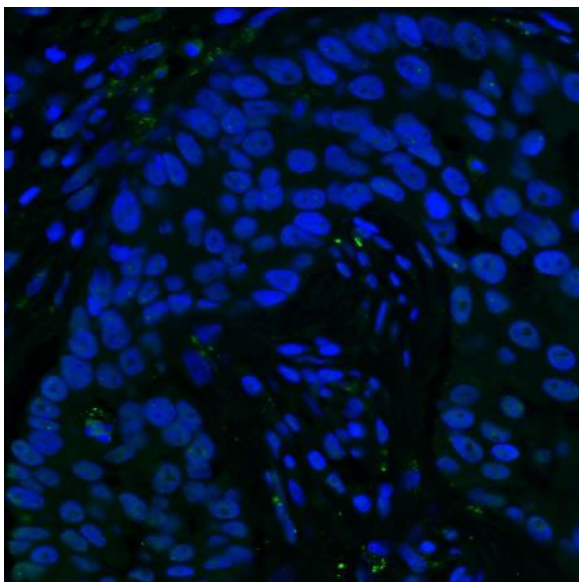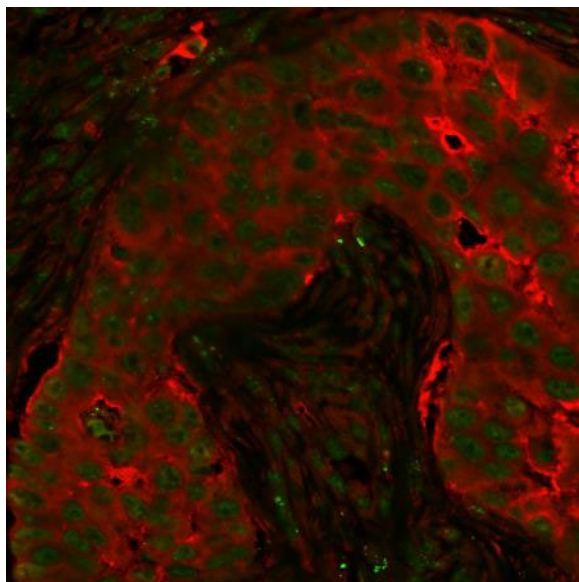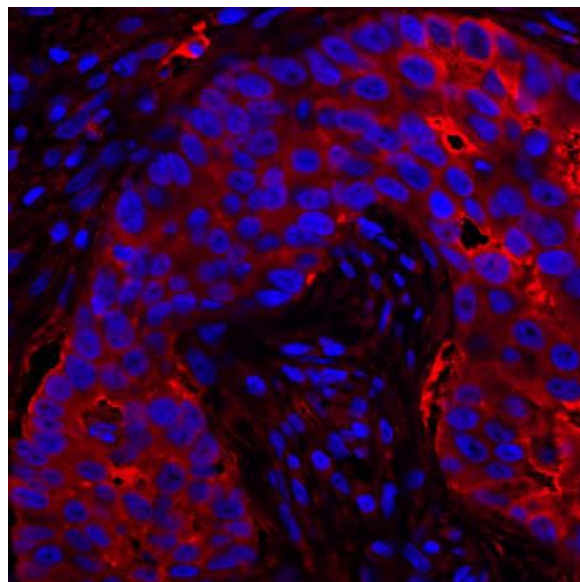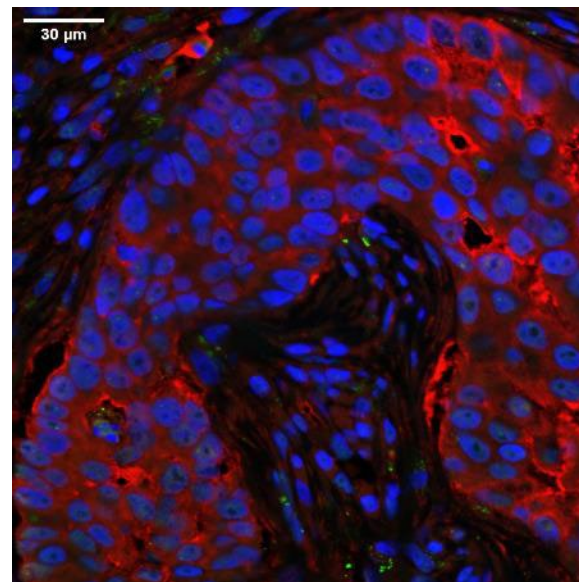

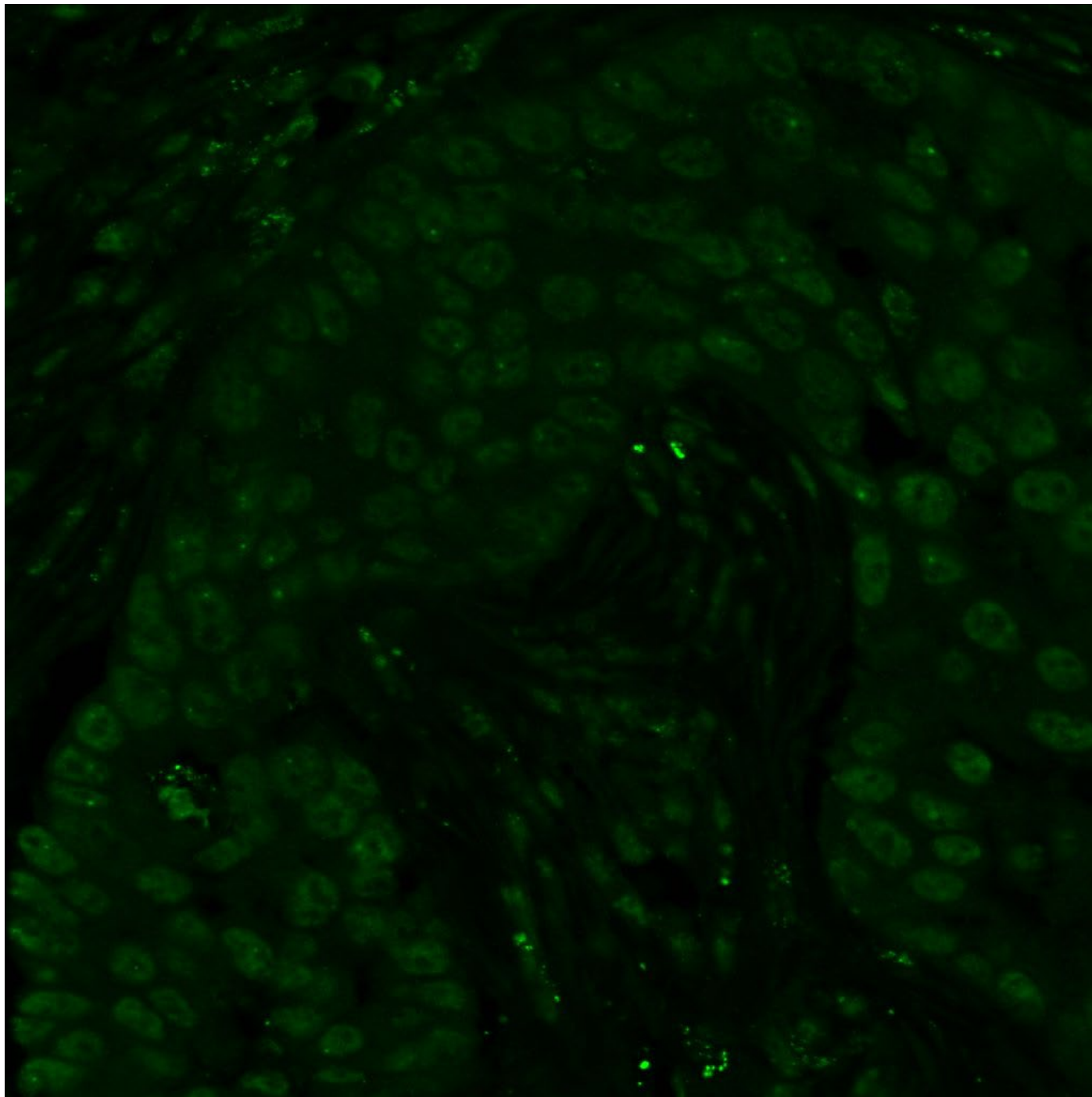

5789\_03

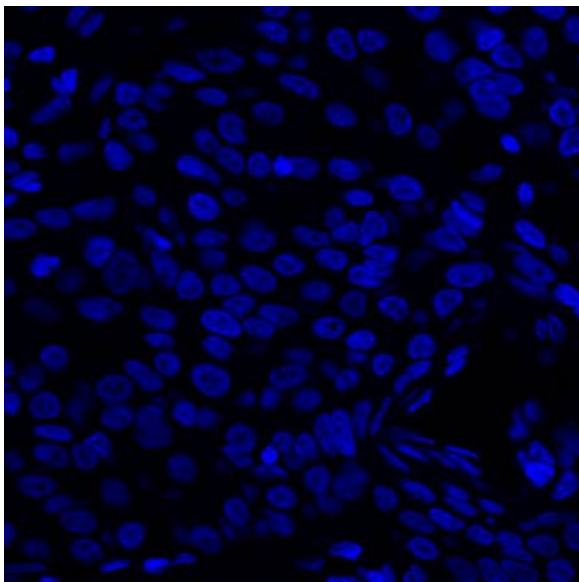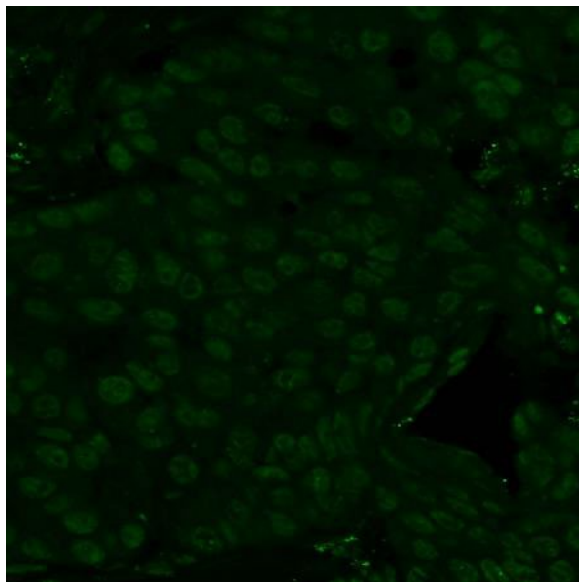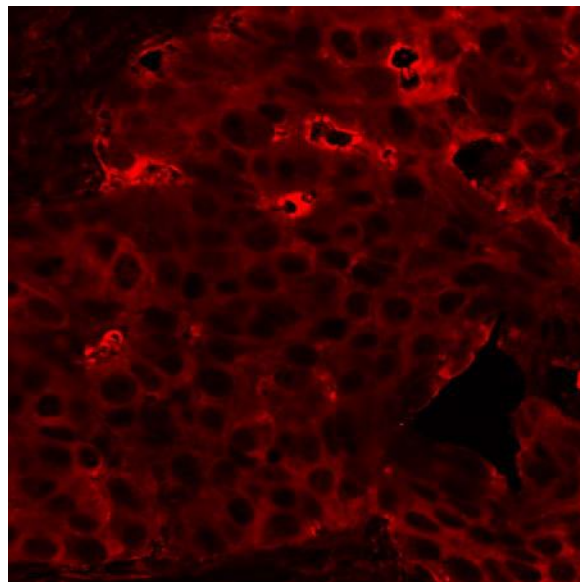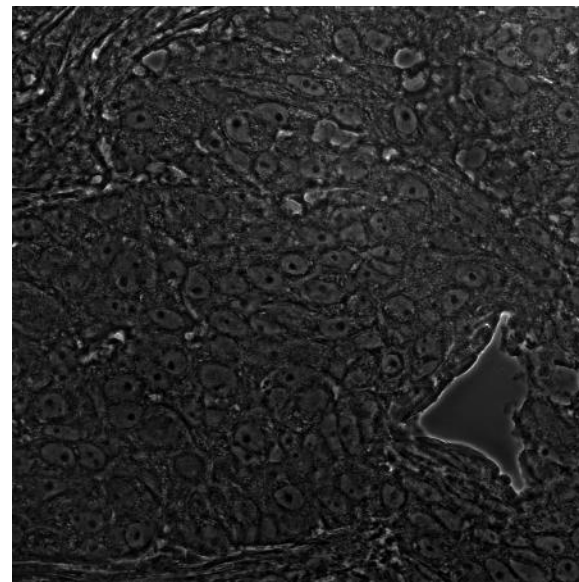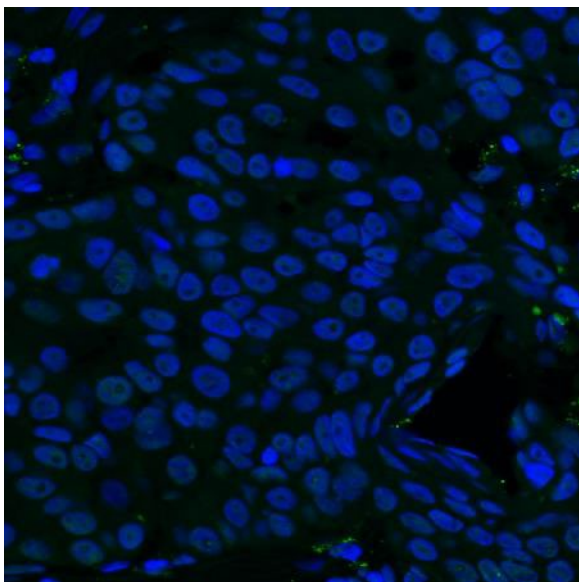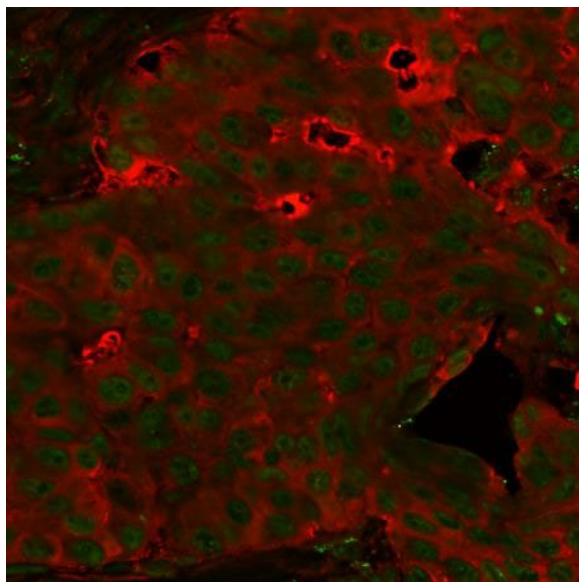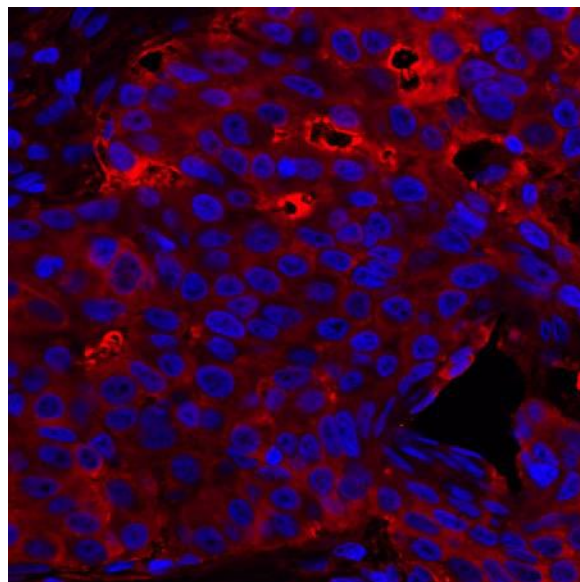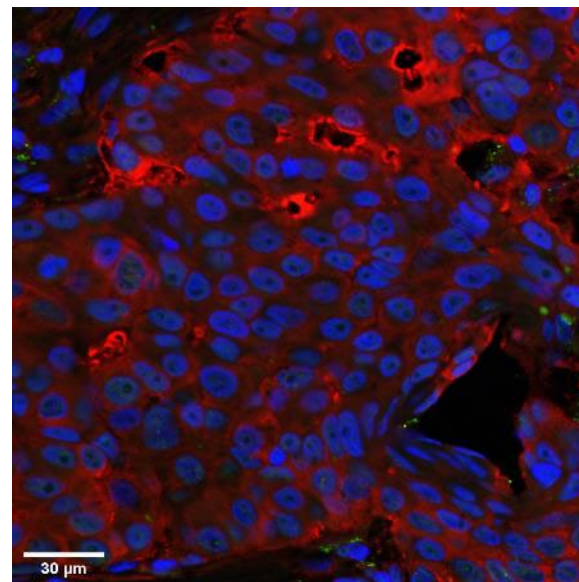

5789\_04

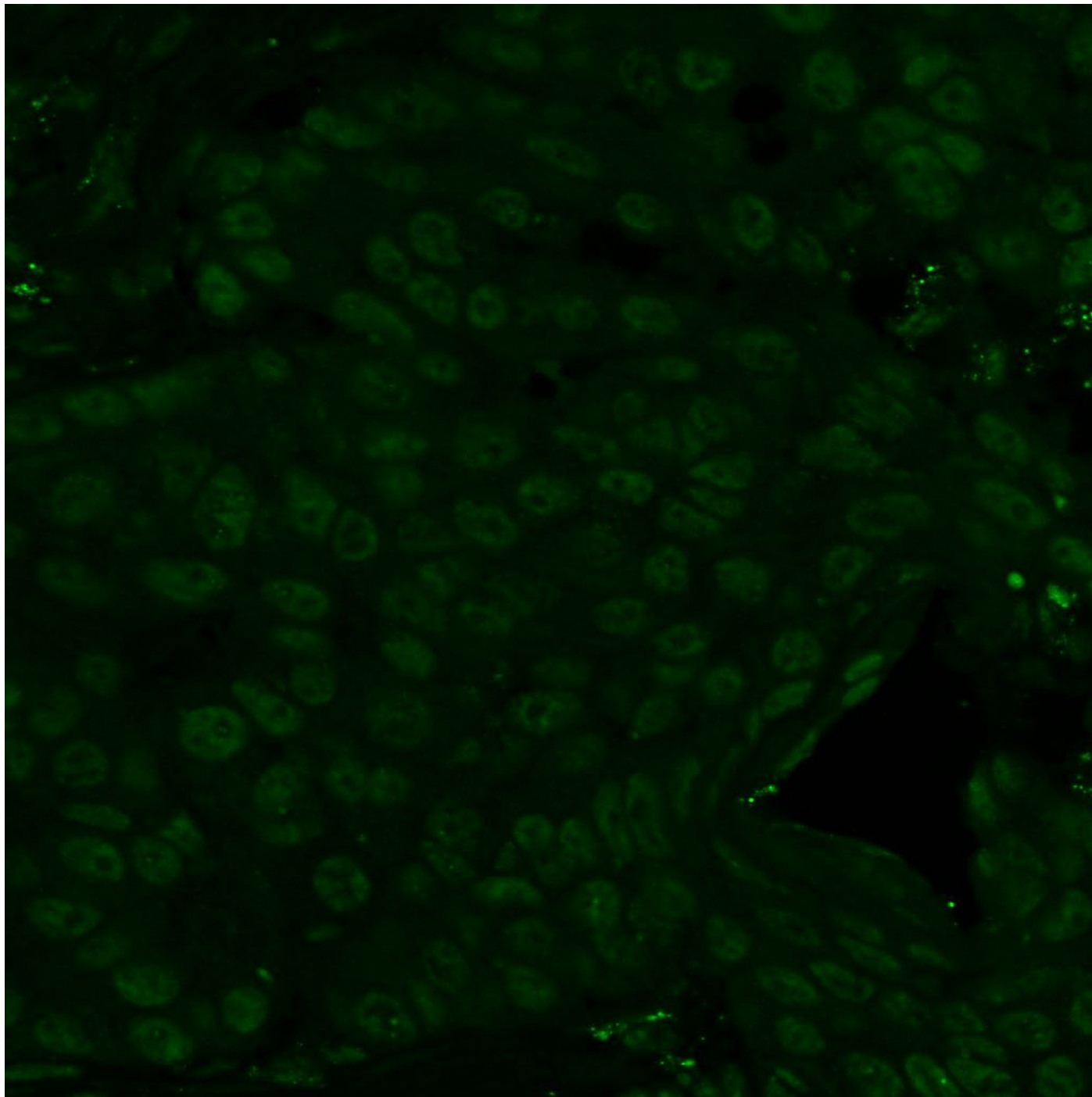

5789\_04

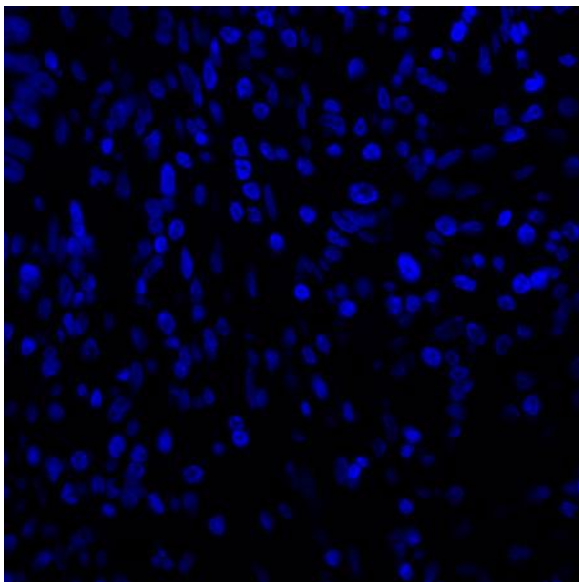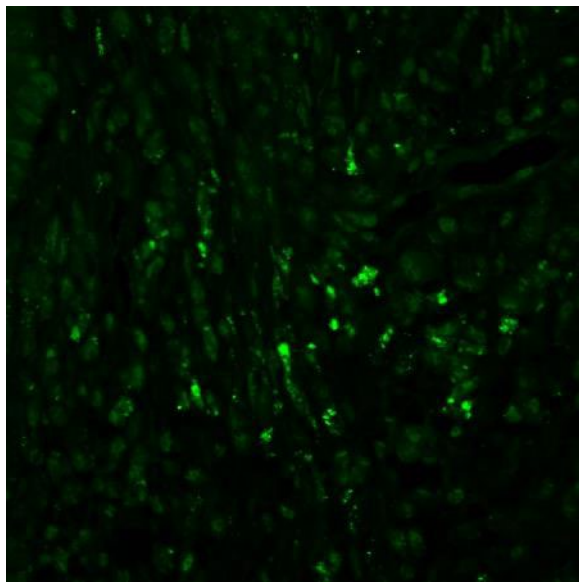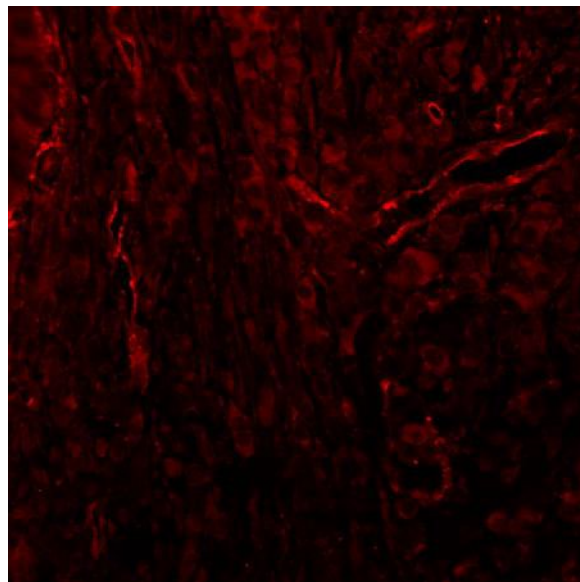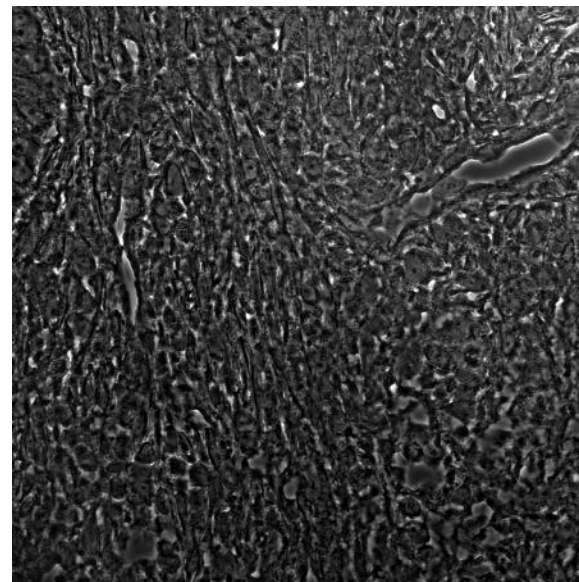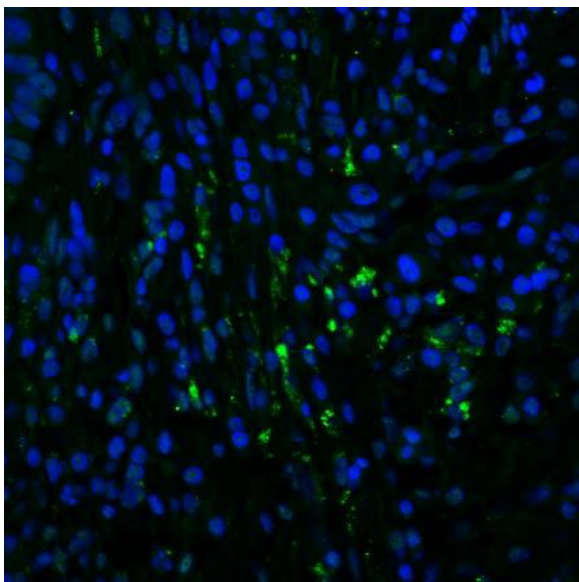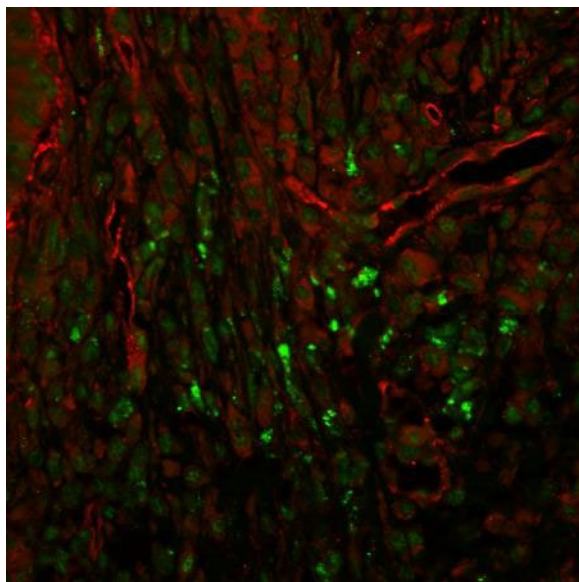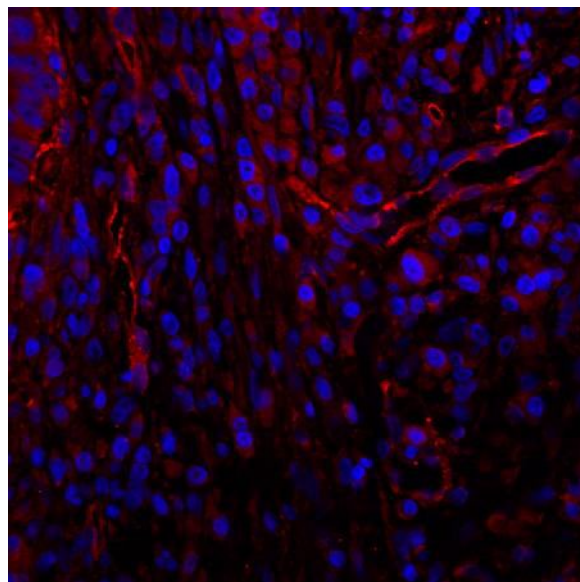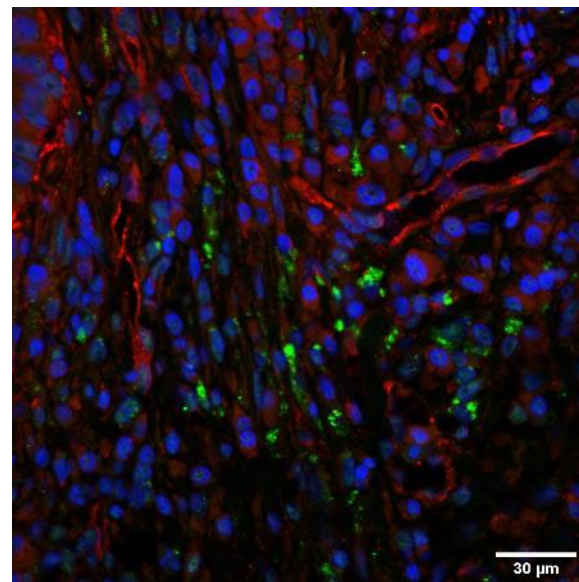

5789\_05

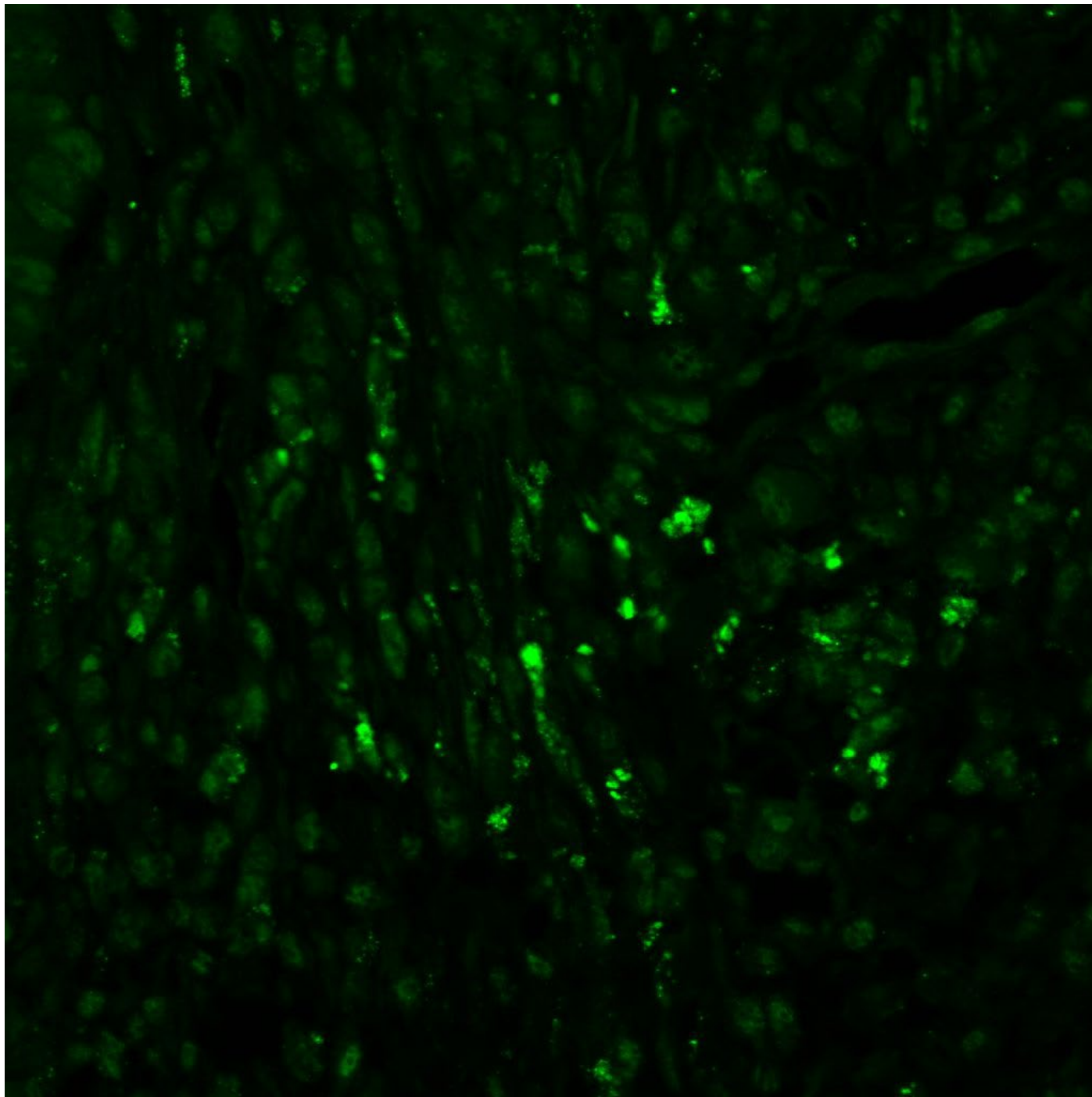

5789\_05

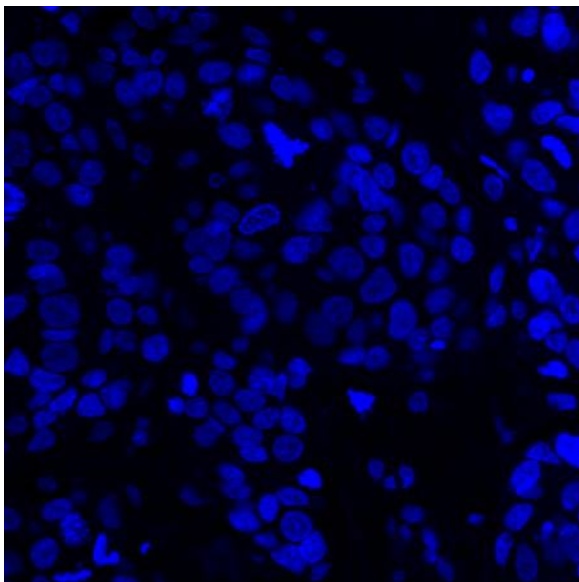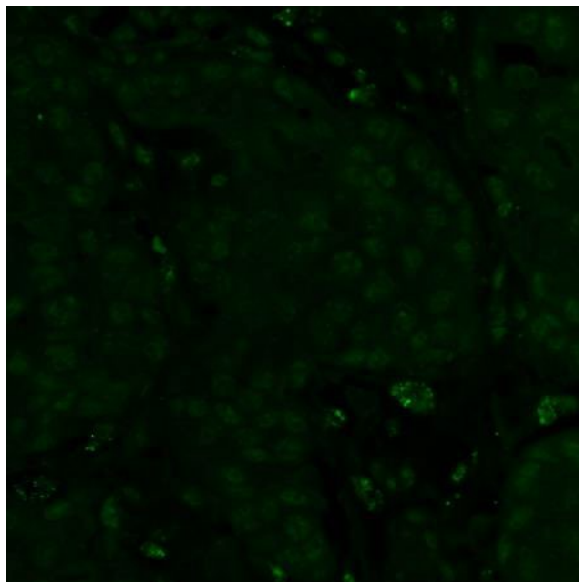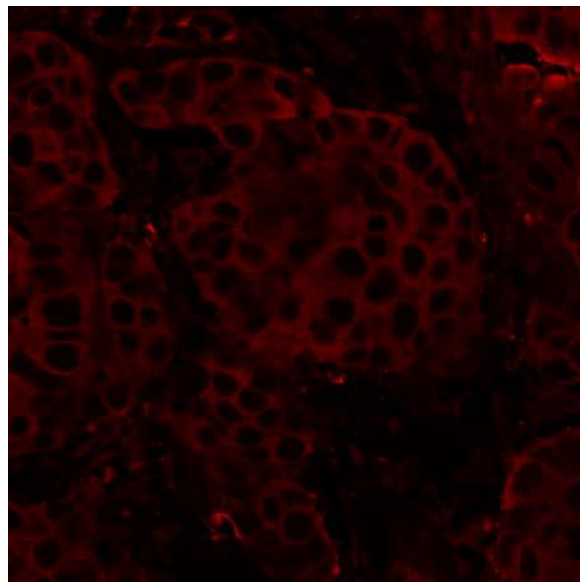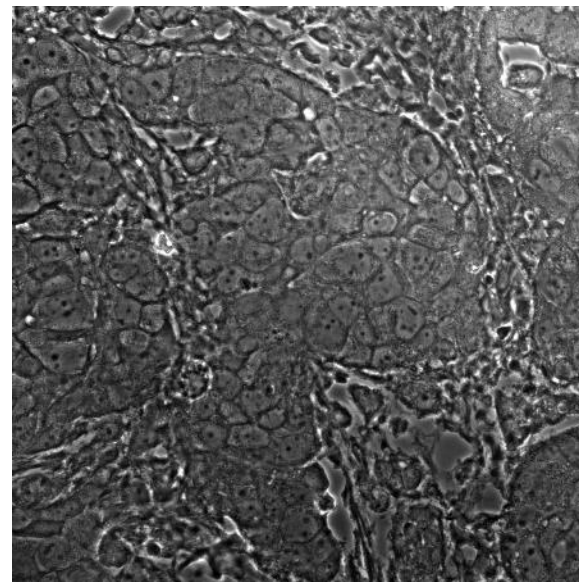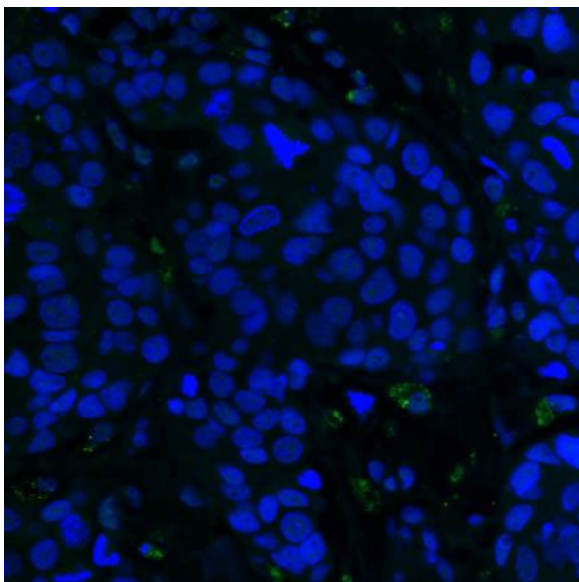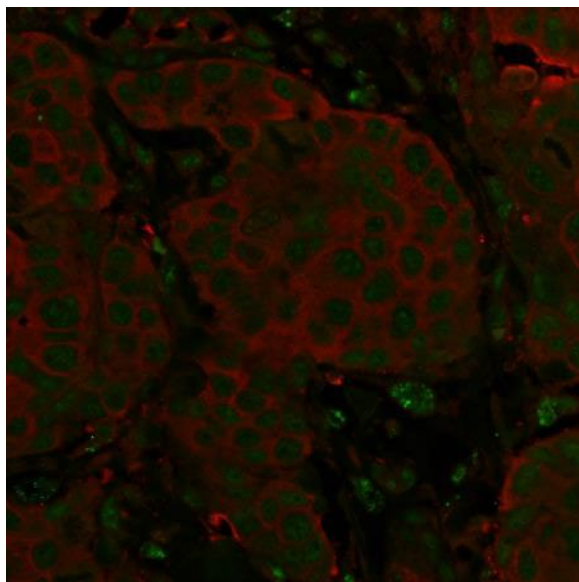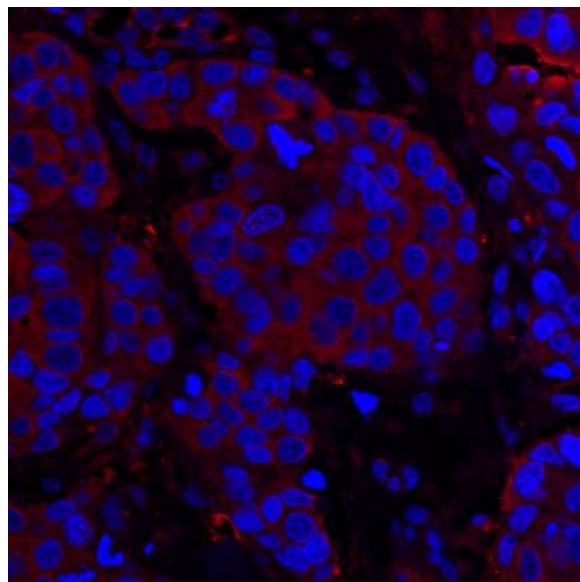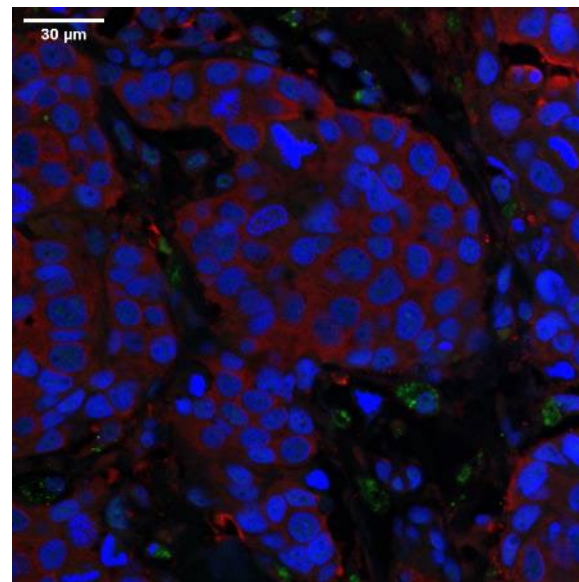

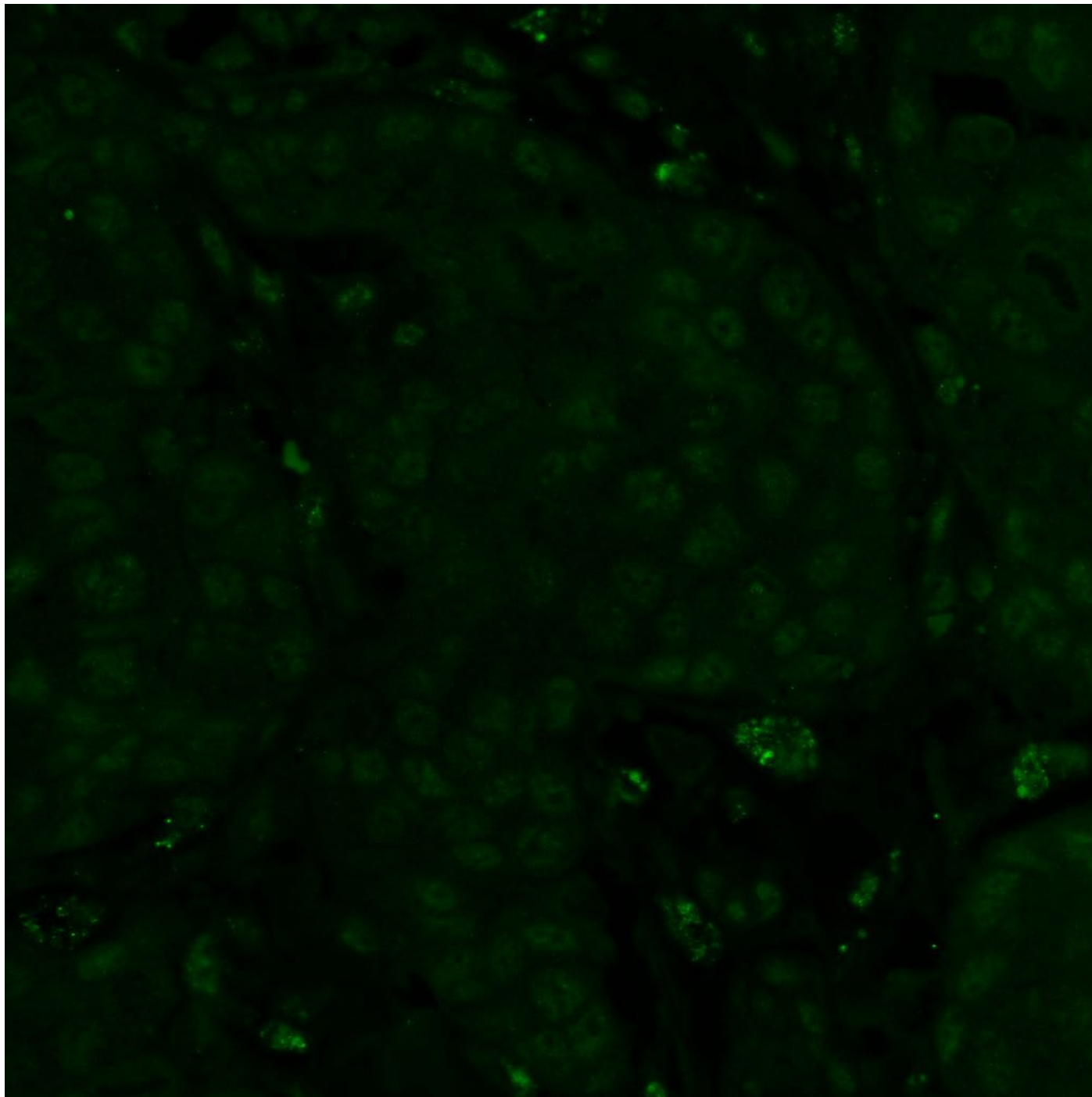

8217\_00

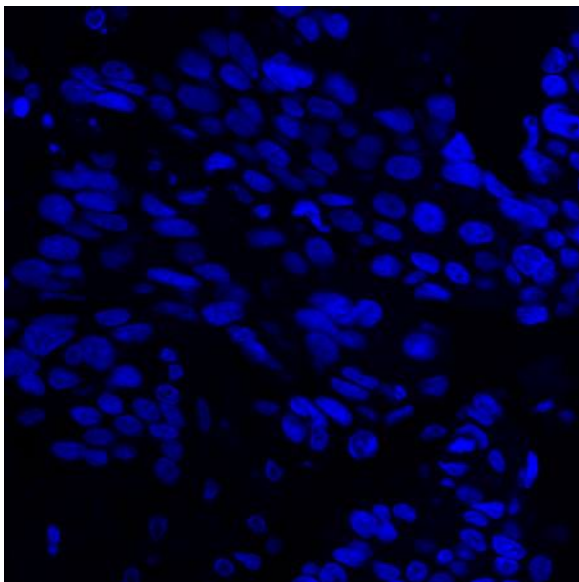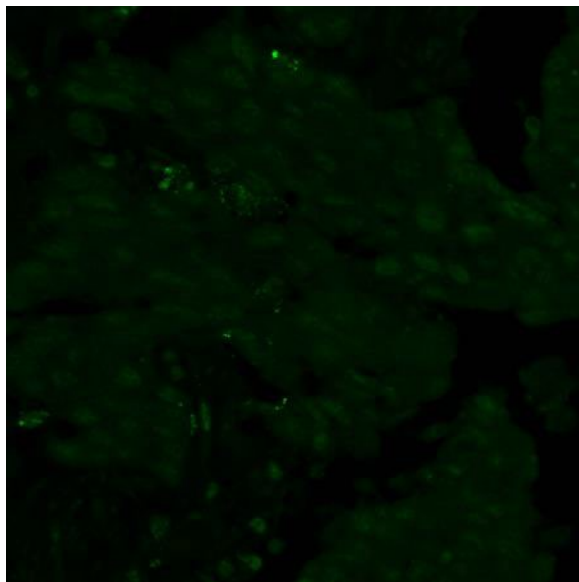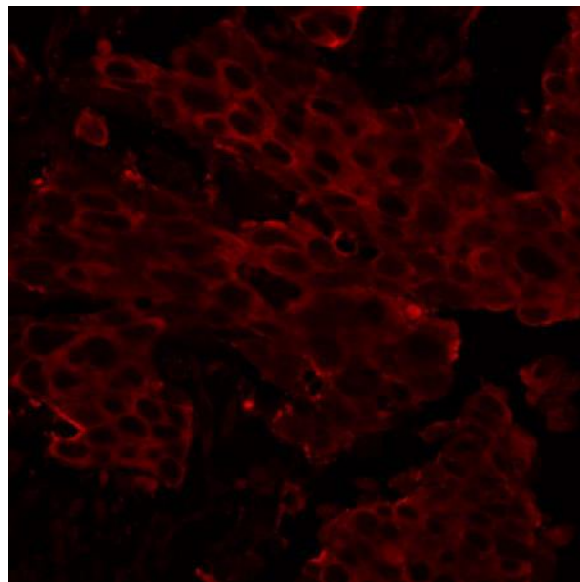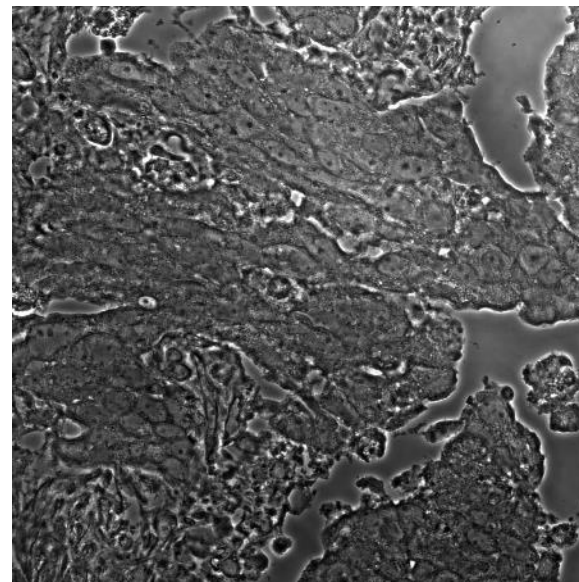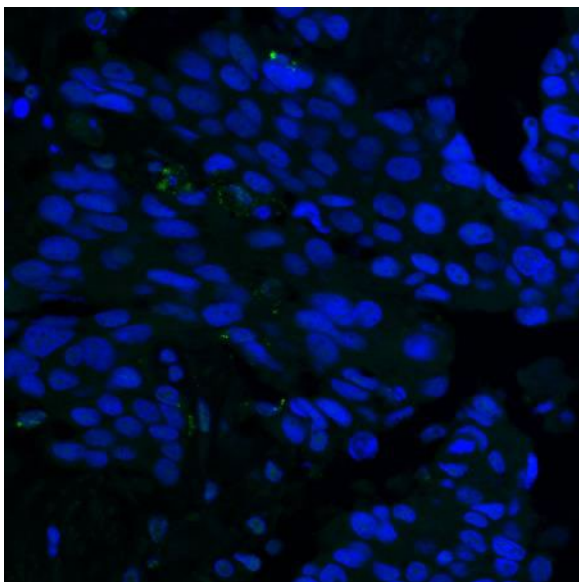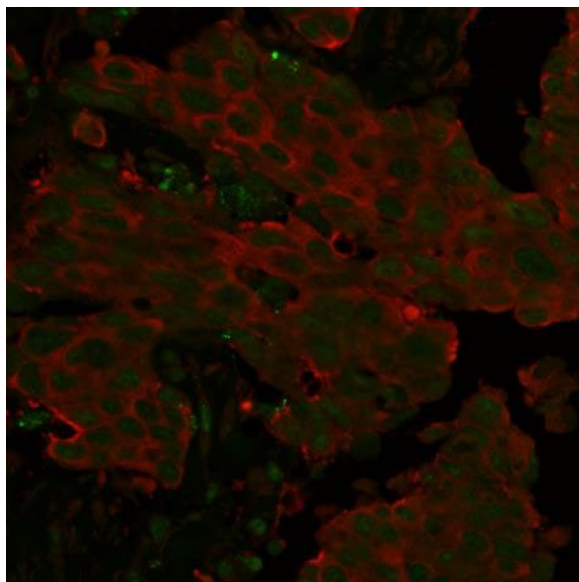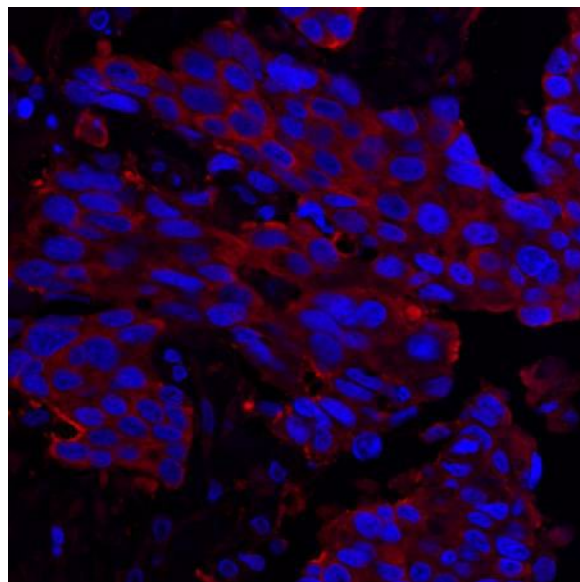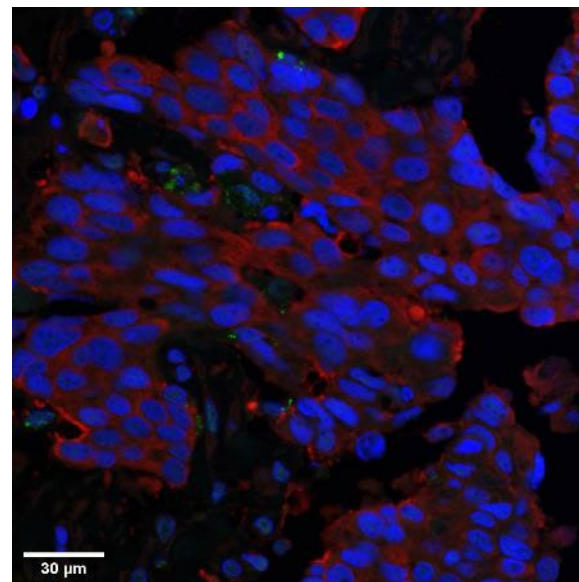

8217\_01

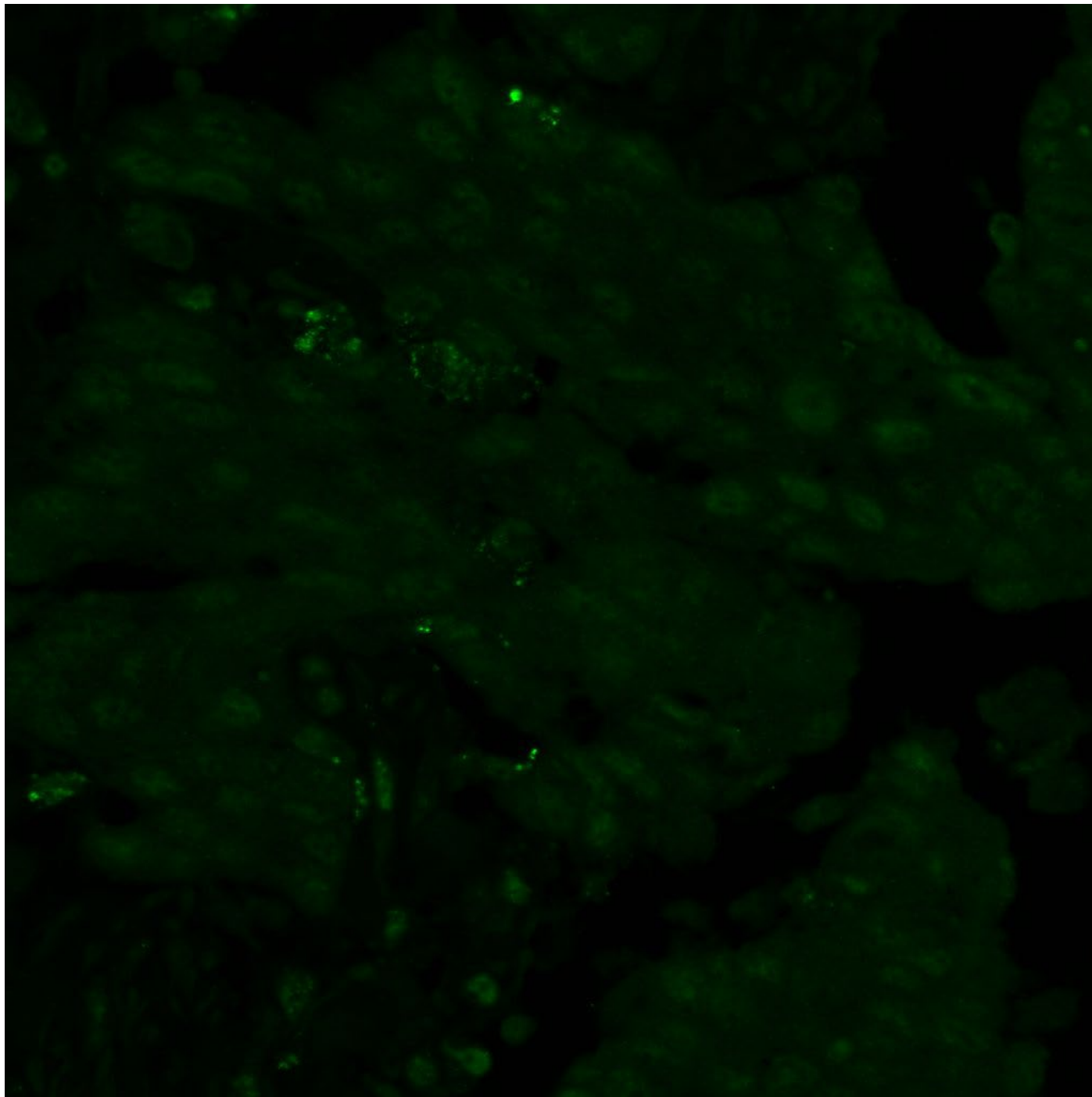

8217\_01

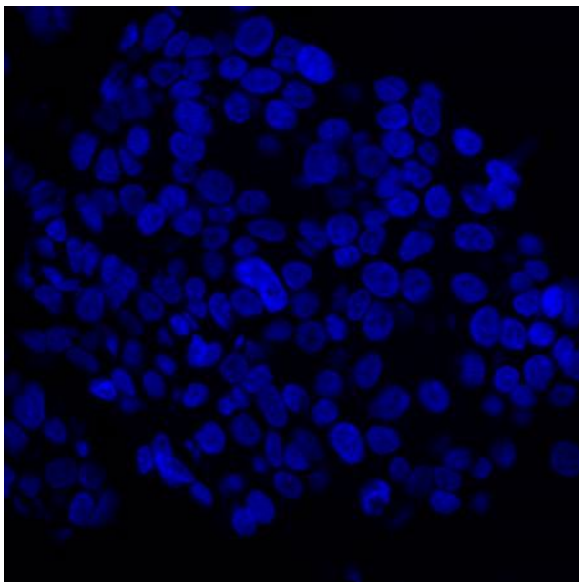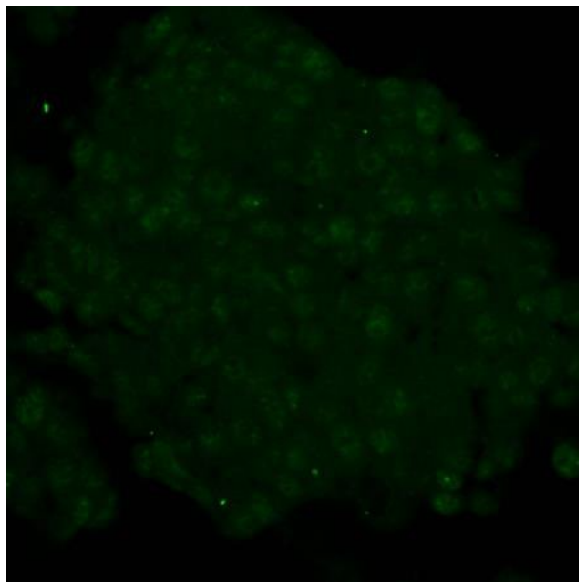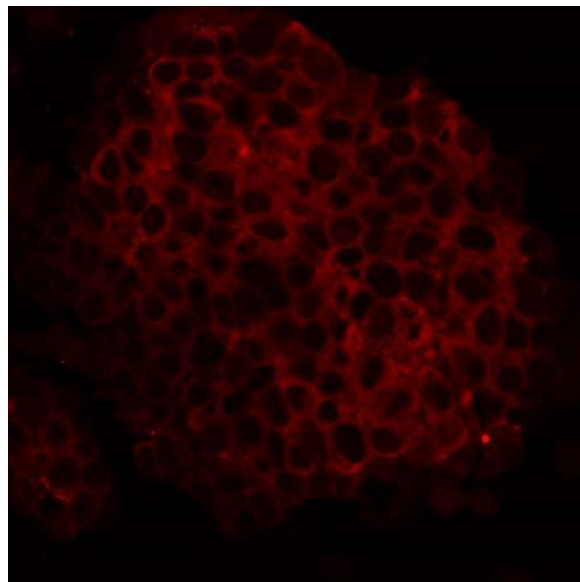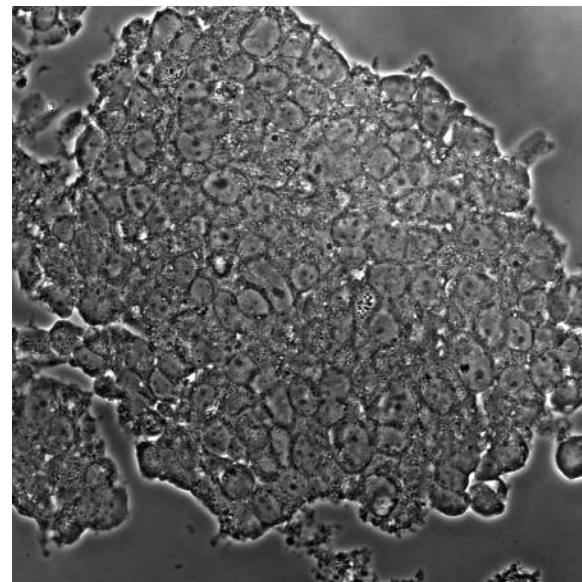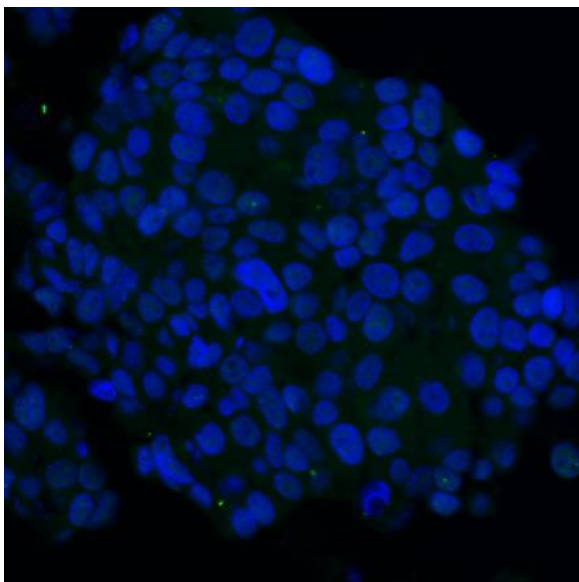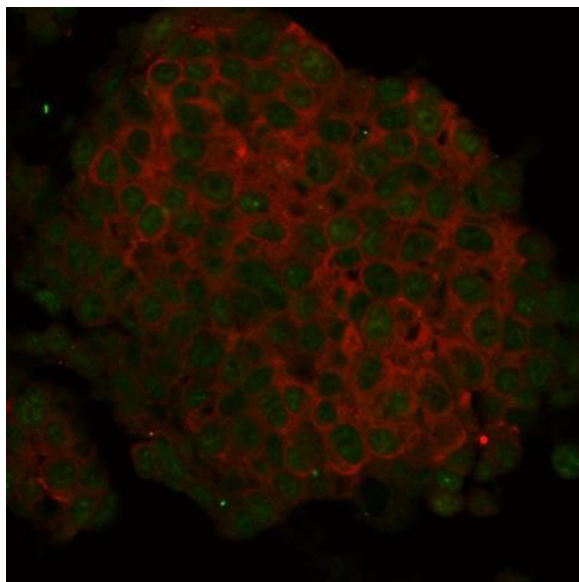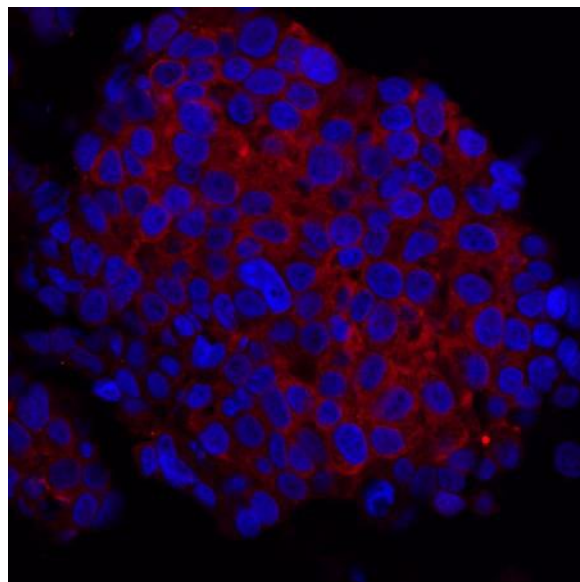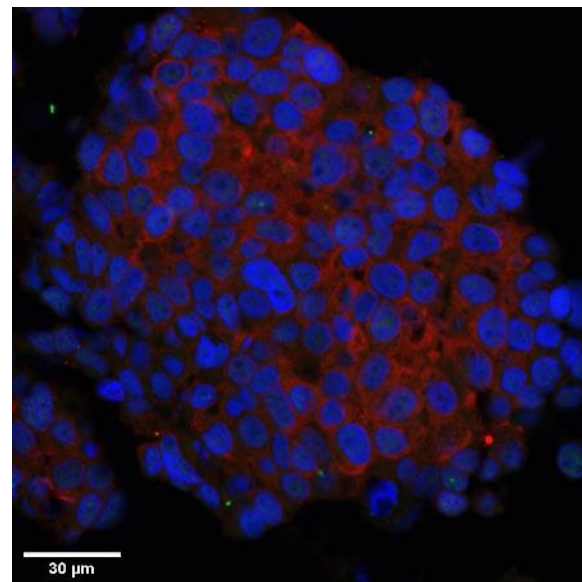

8217\_02

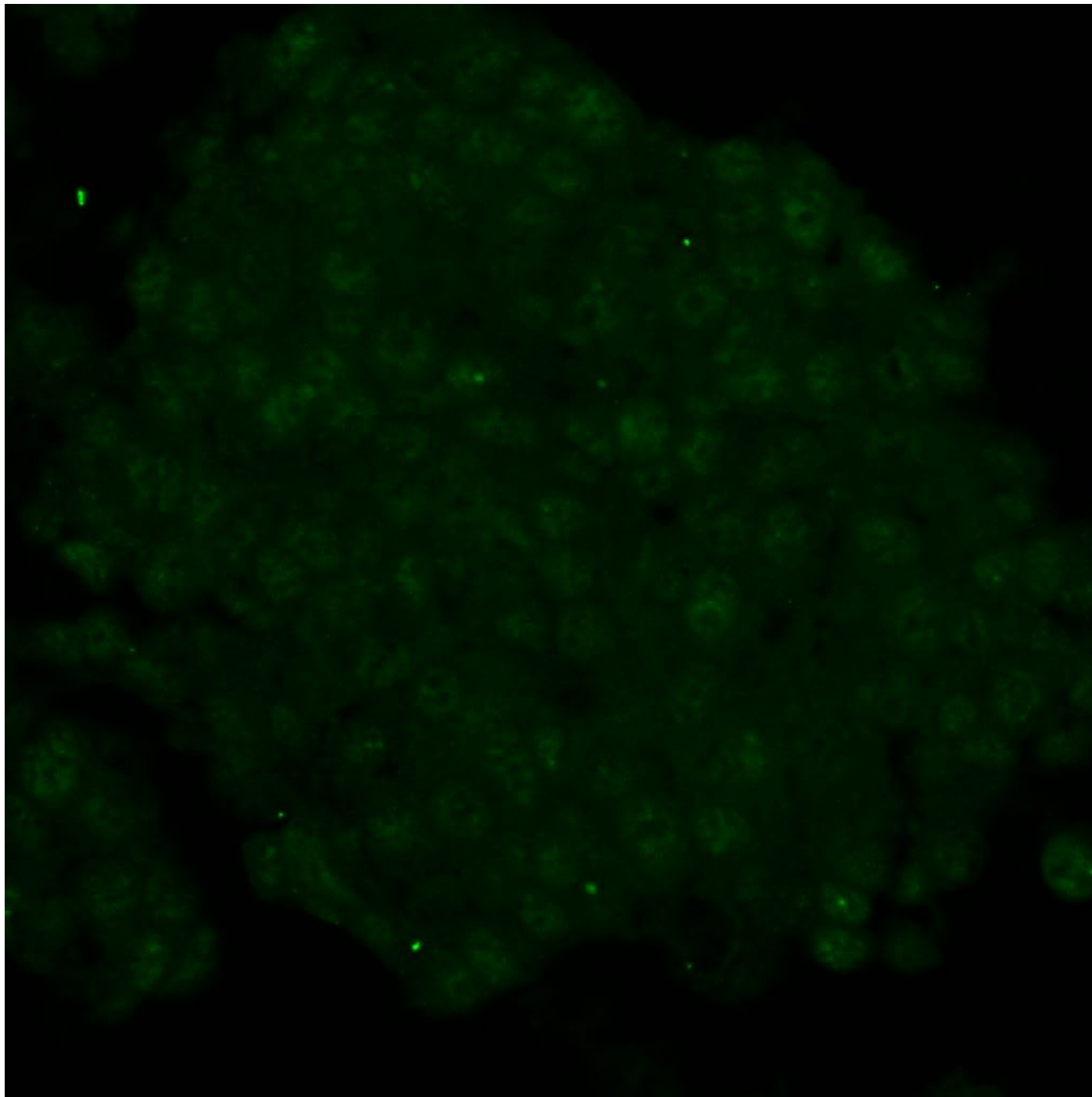

8217\_02

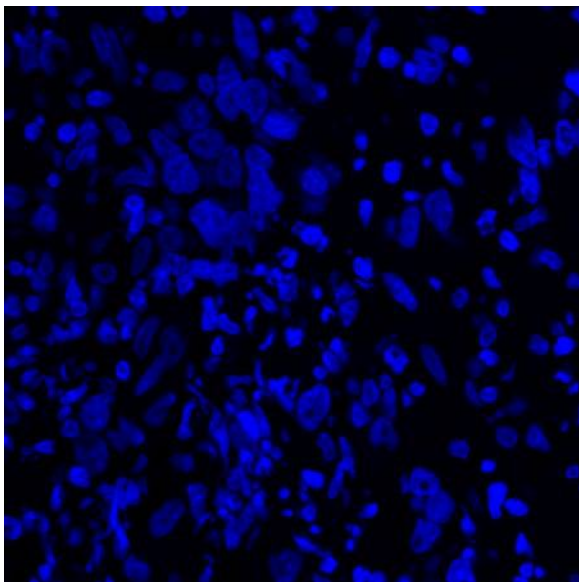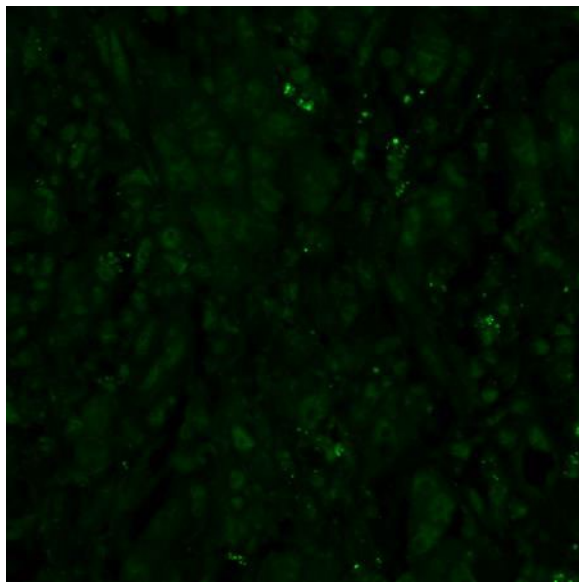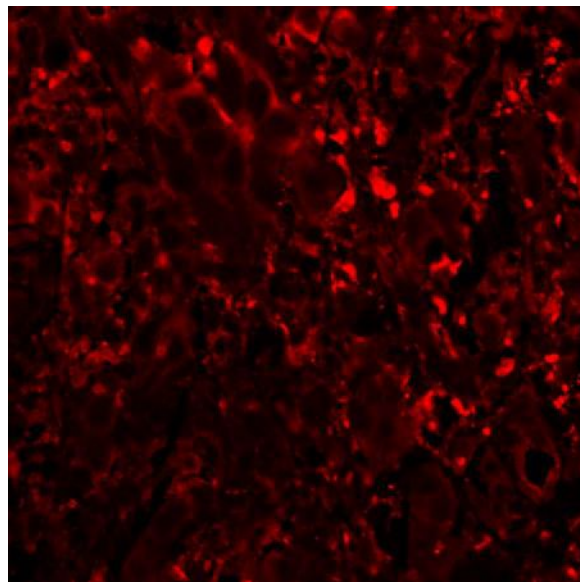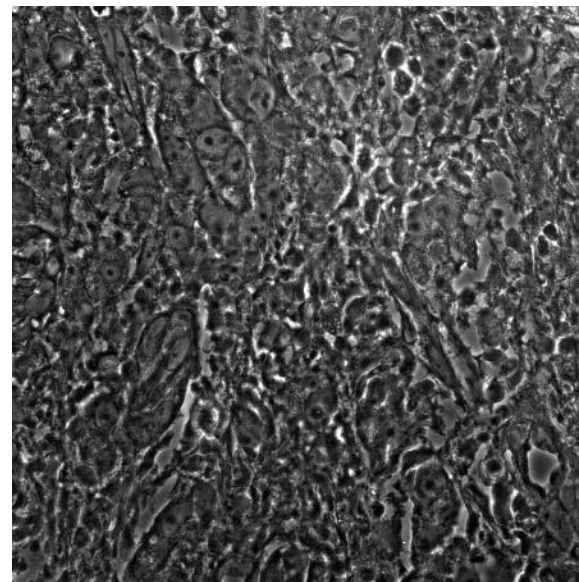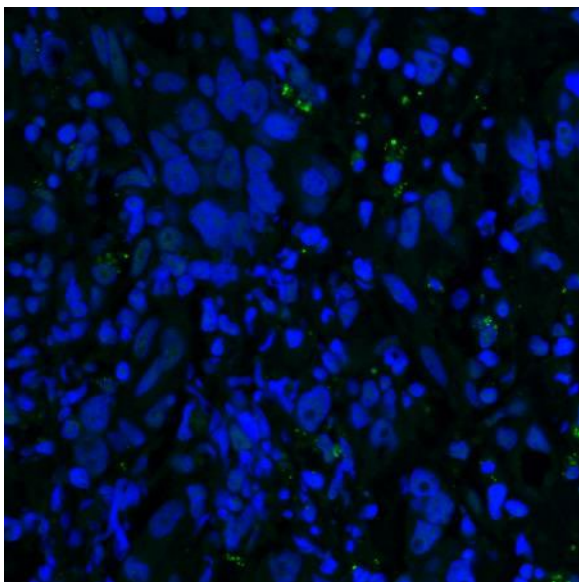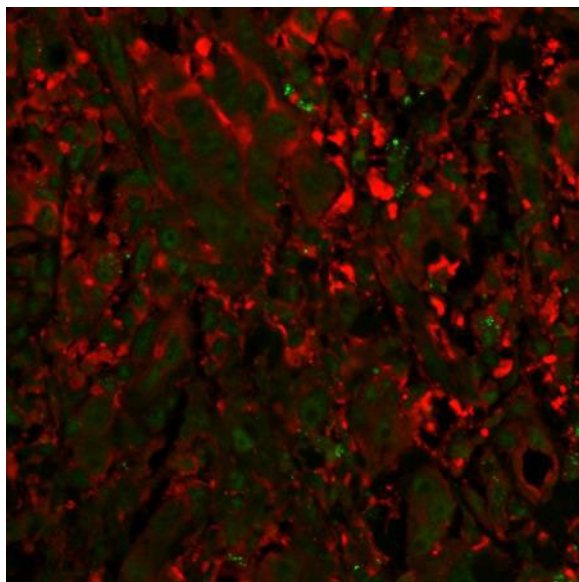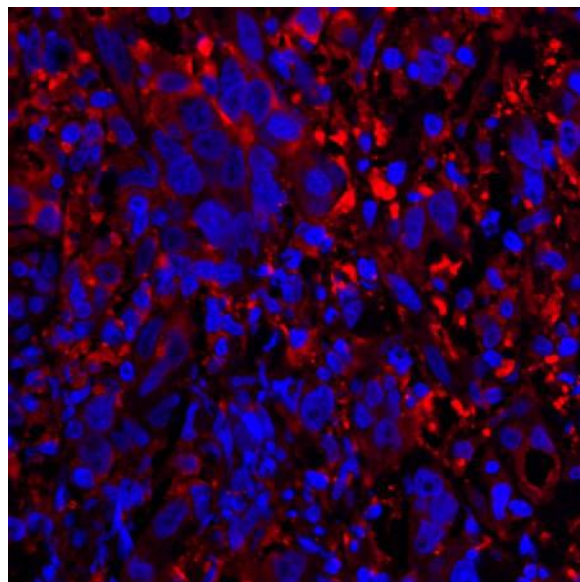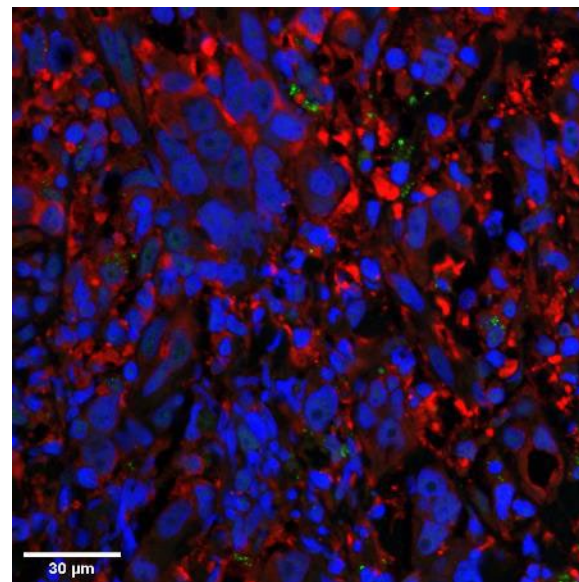

8217\_03

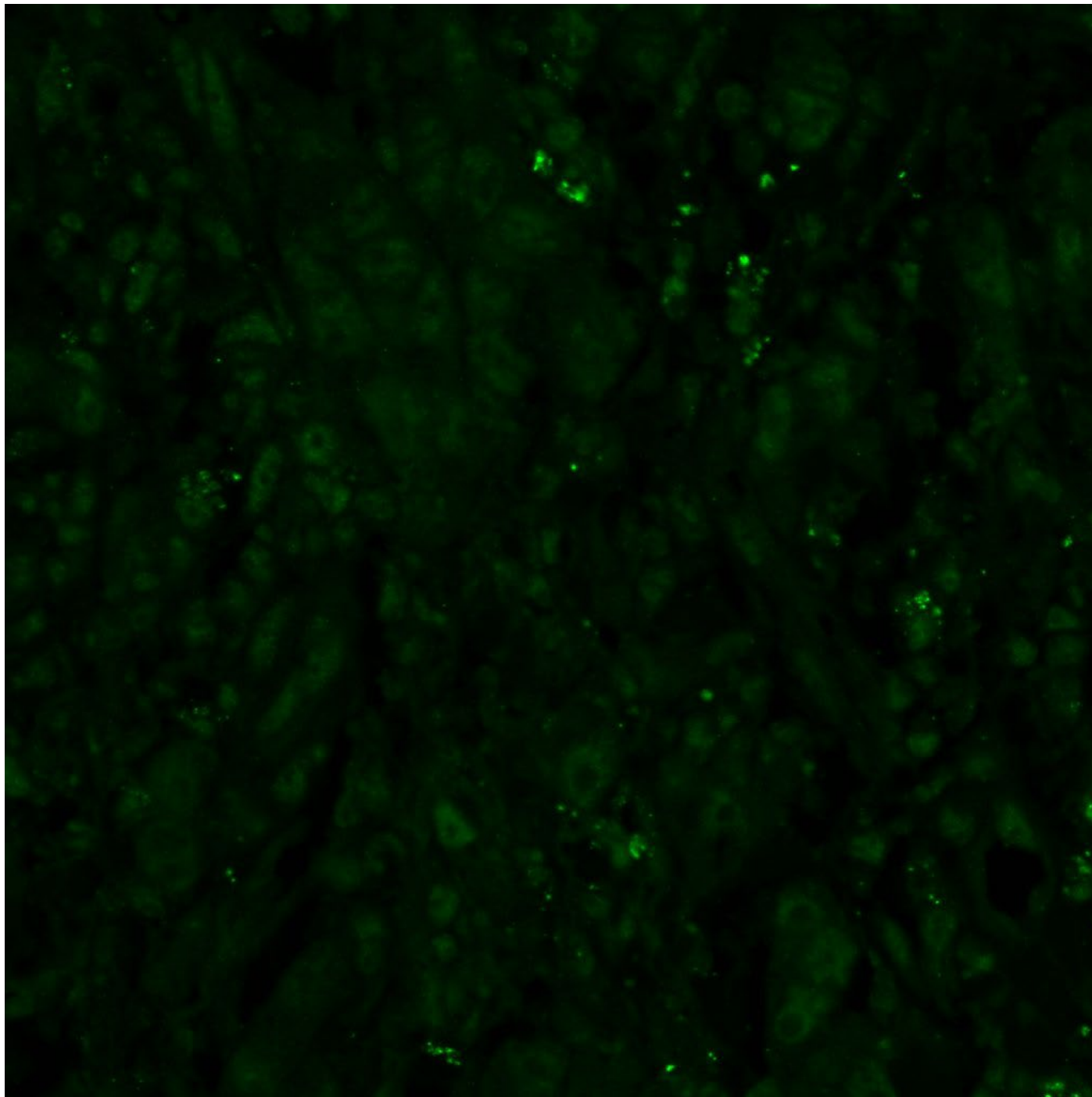

8217\_03

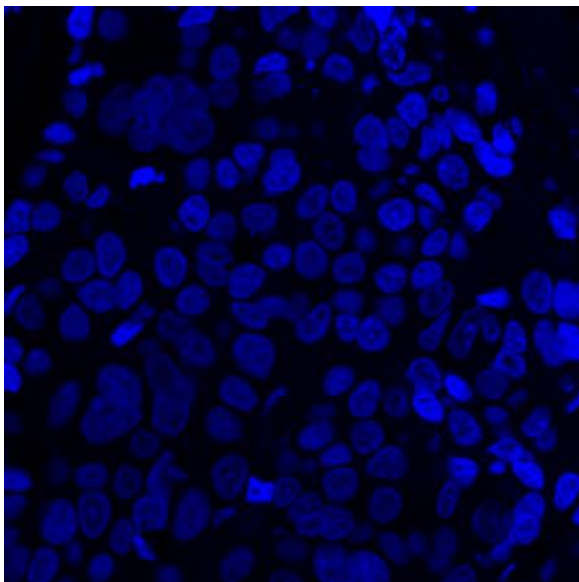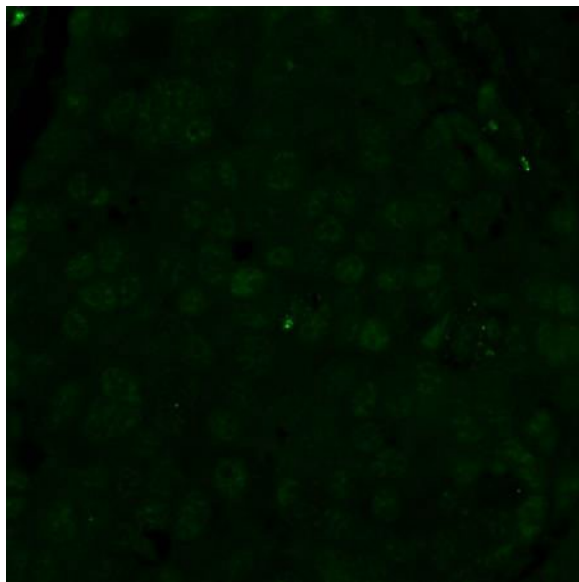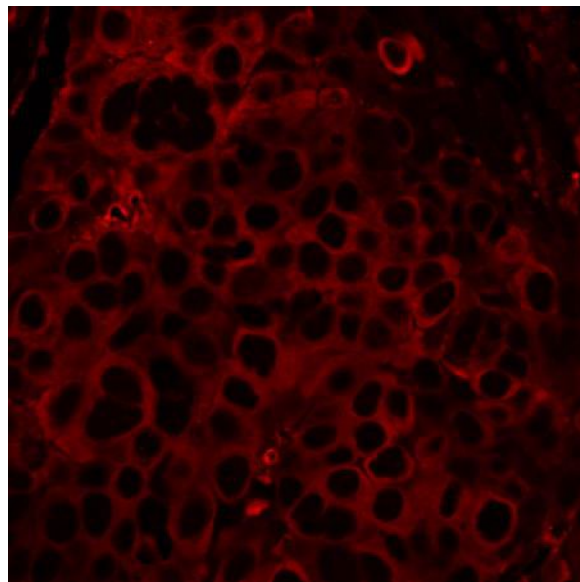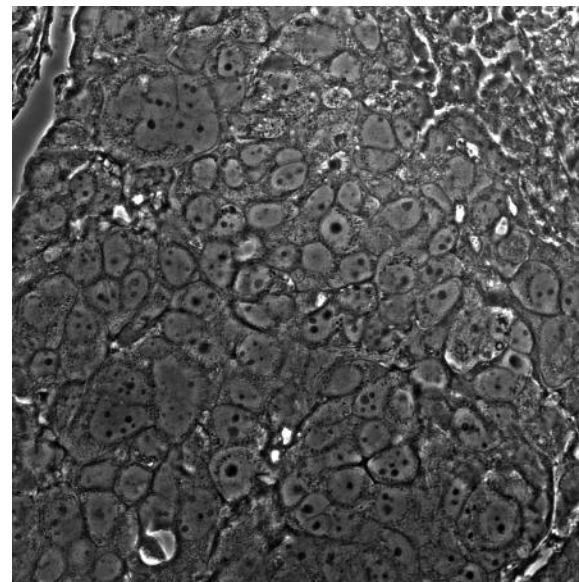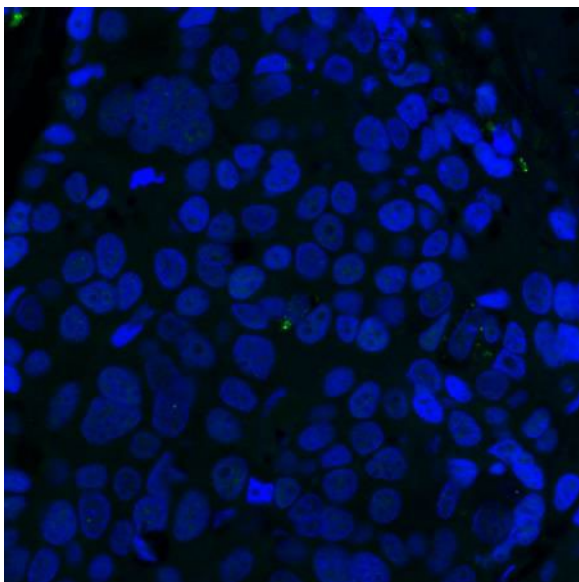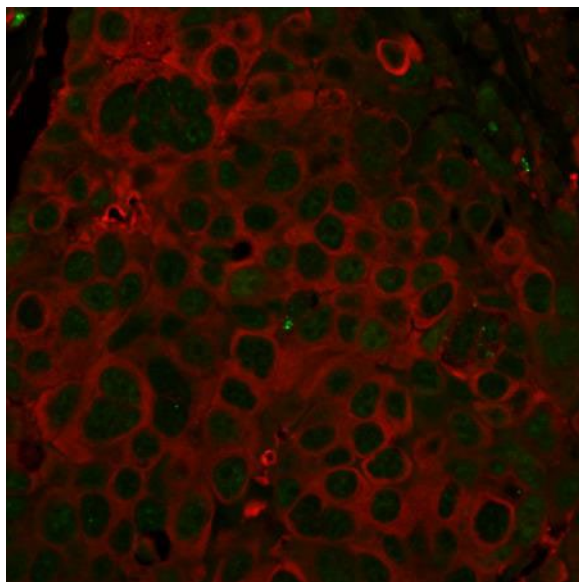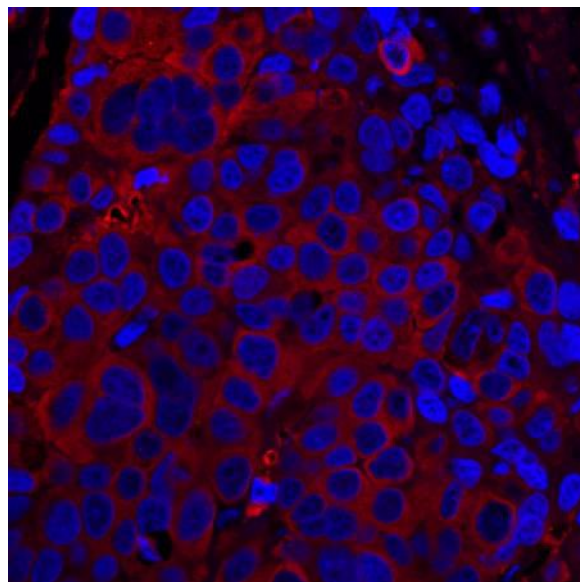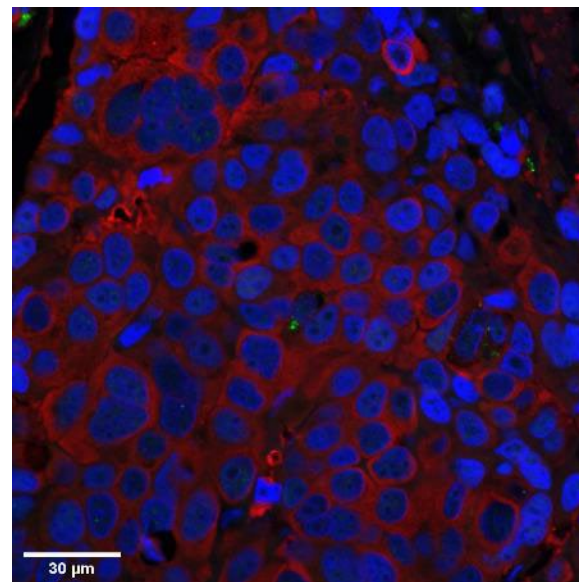

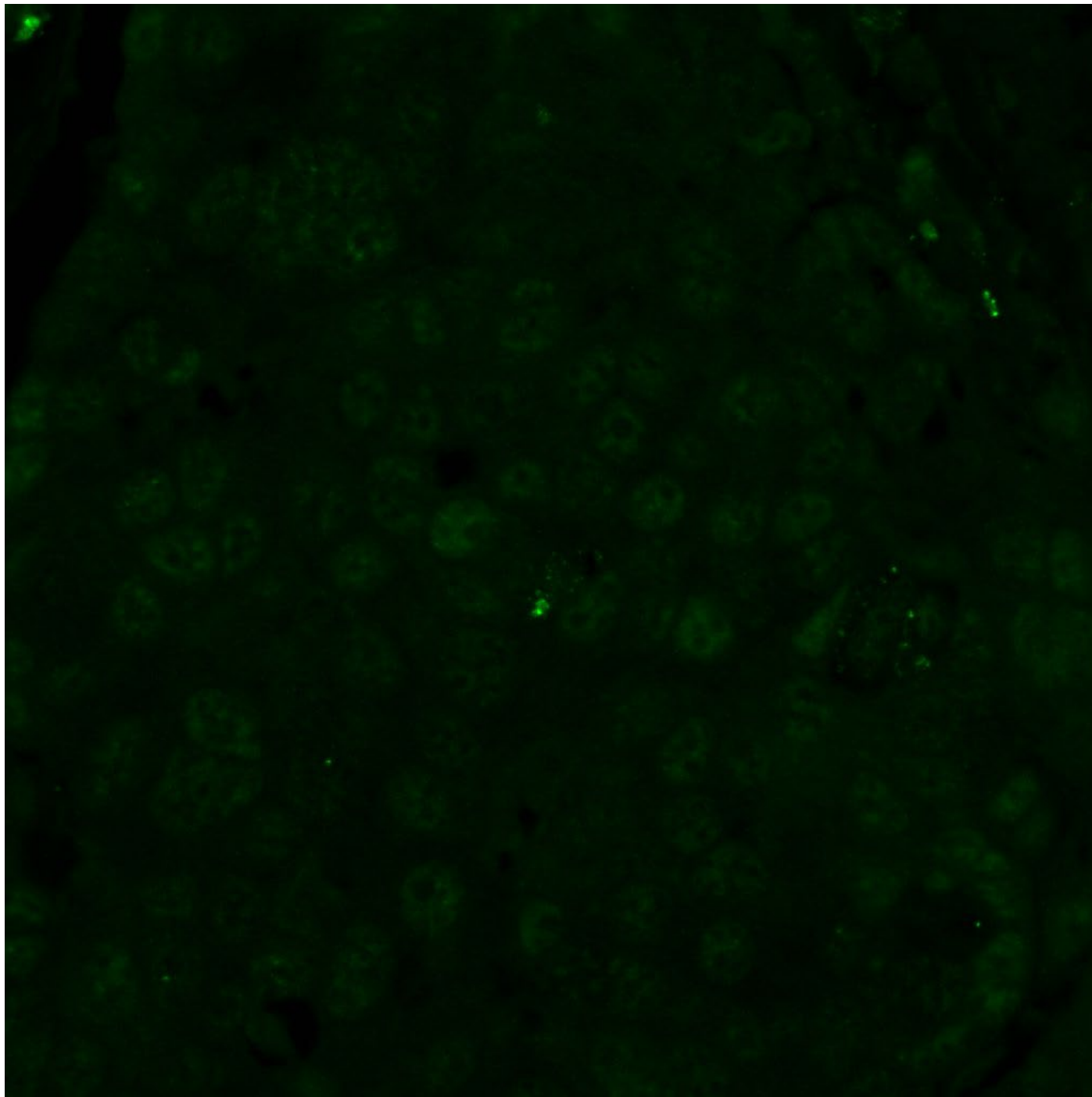

8217\_04

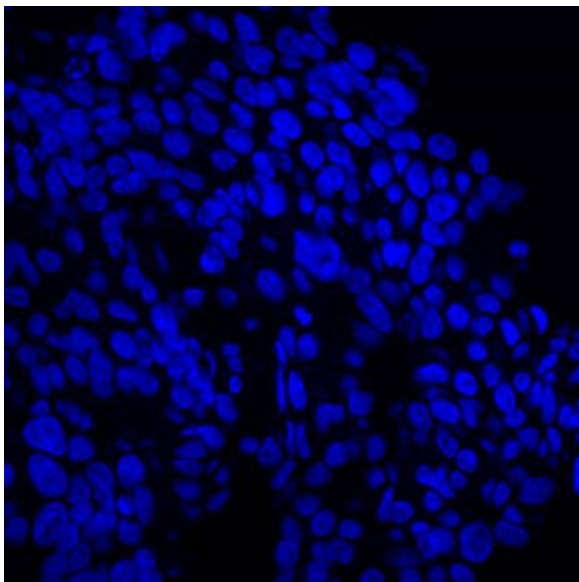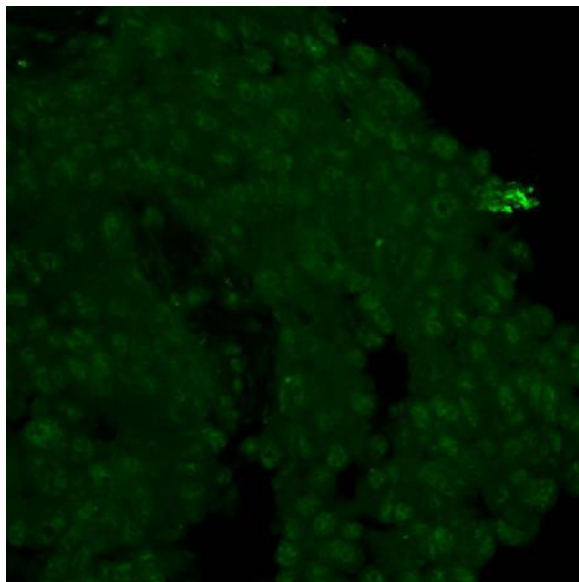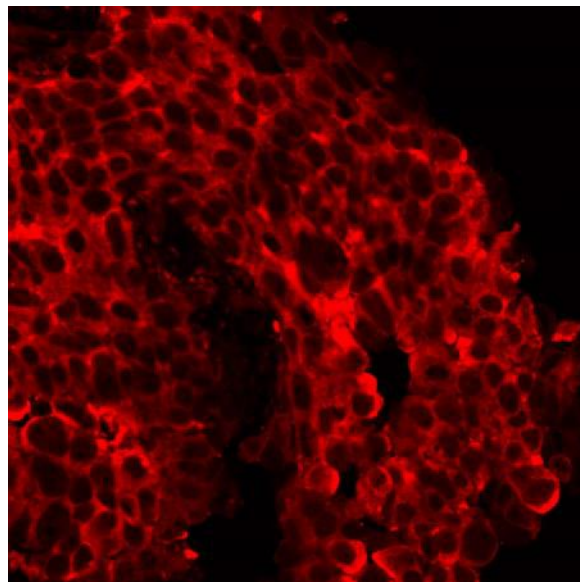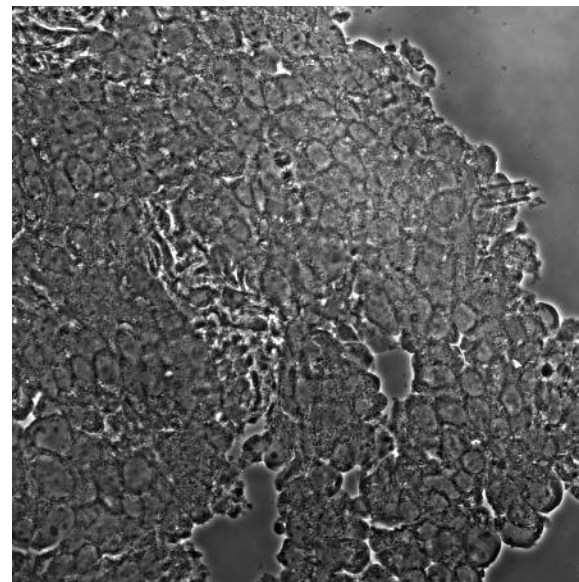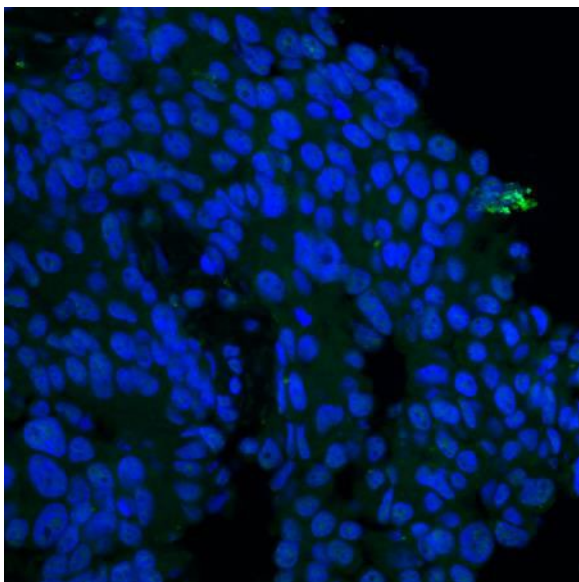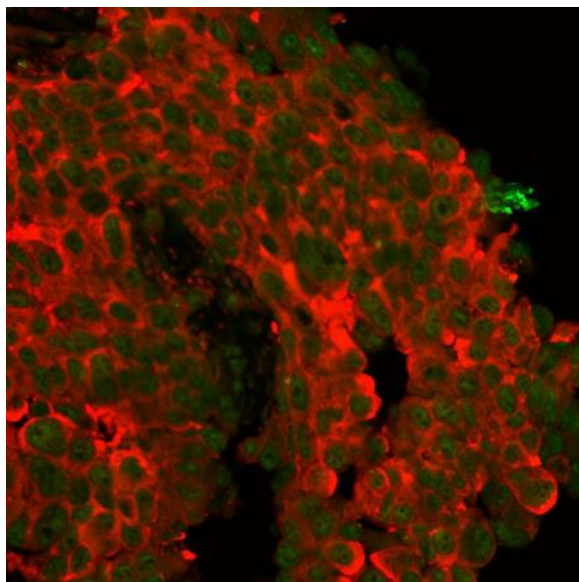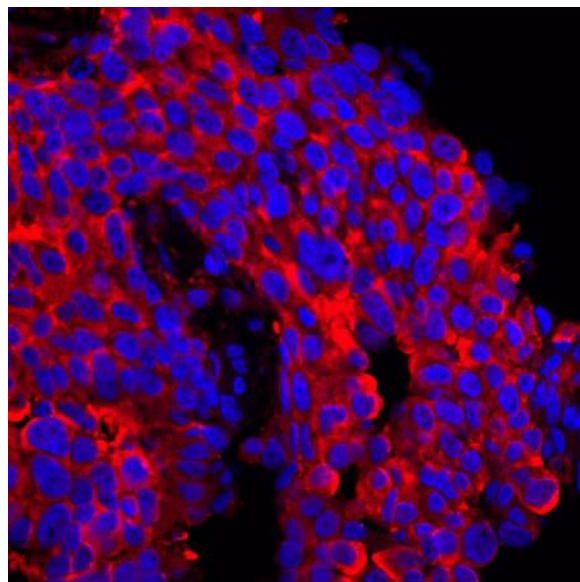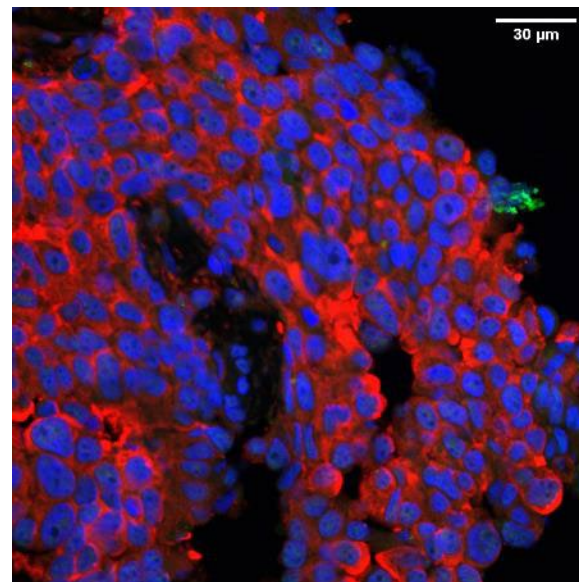

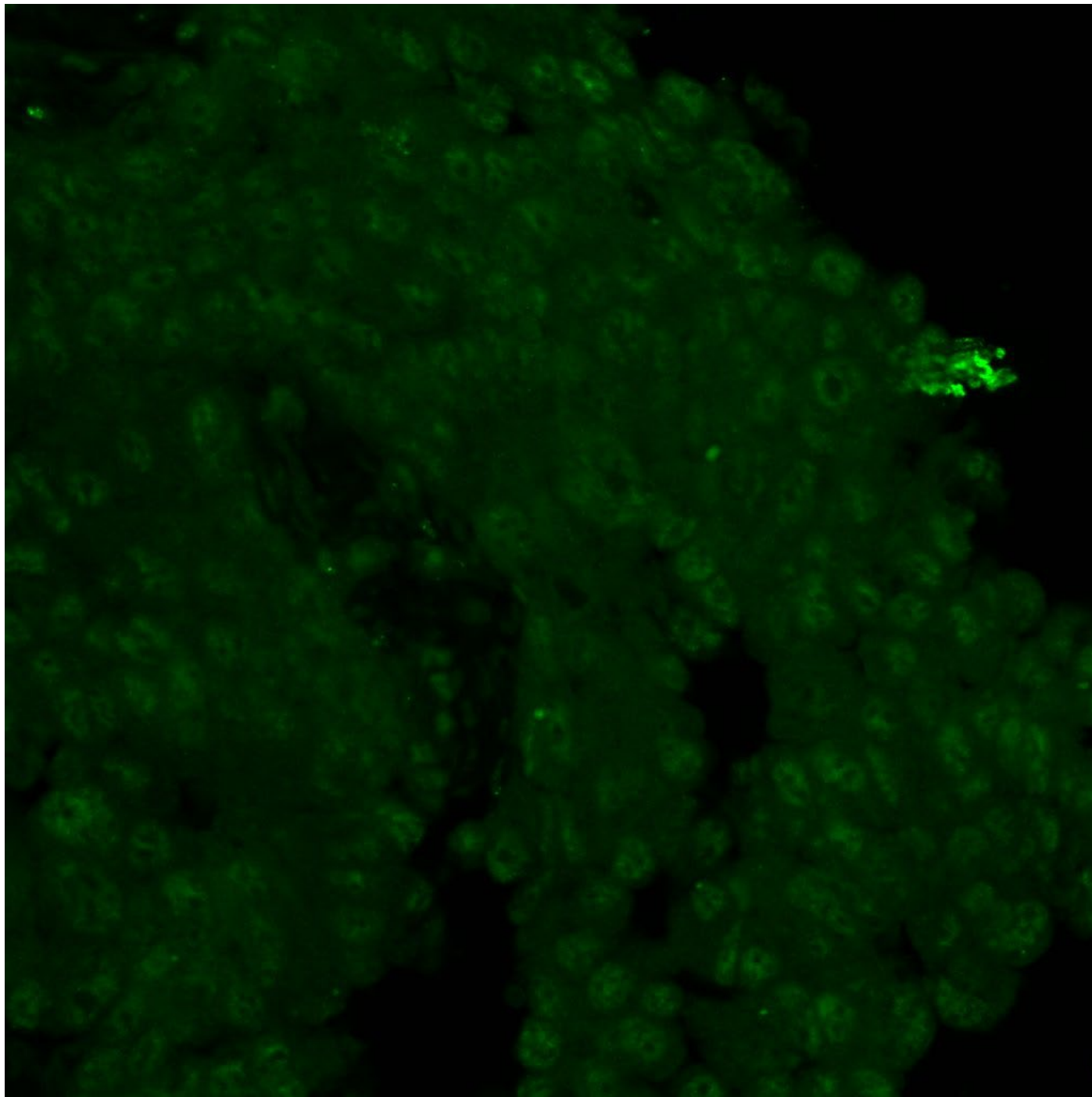

8217\_05

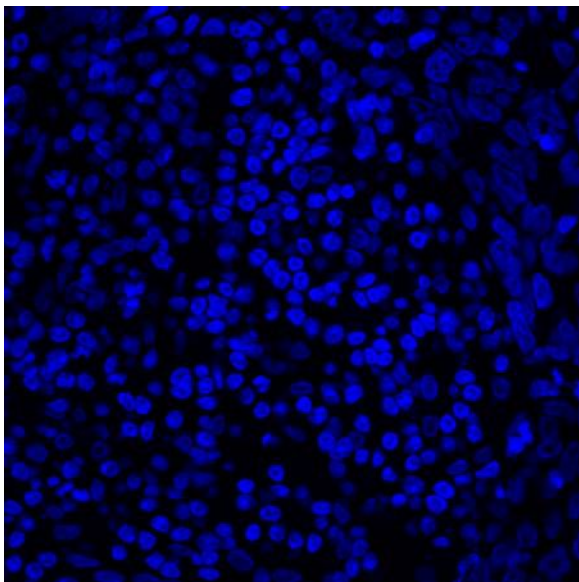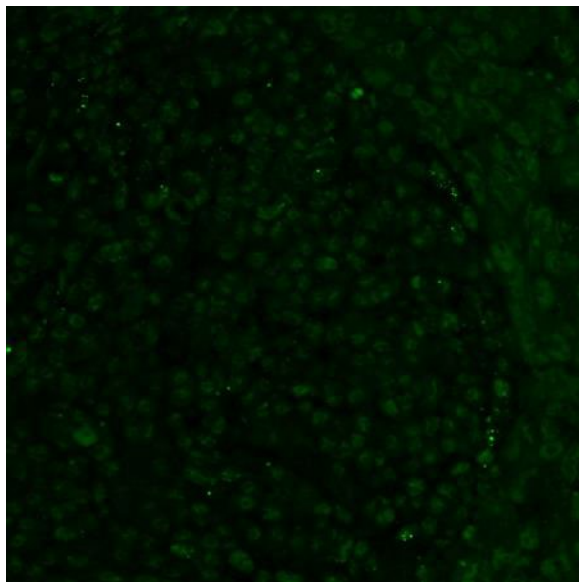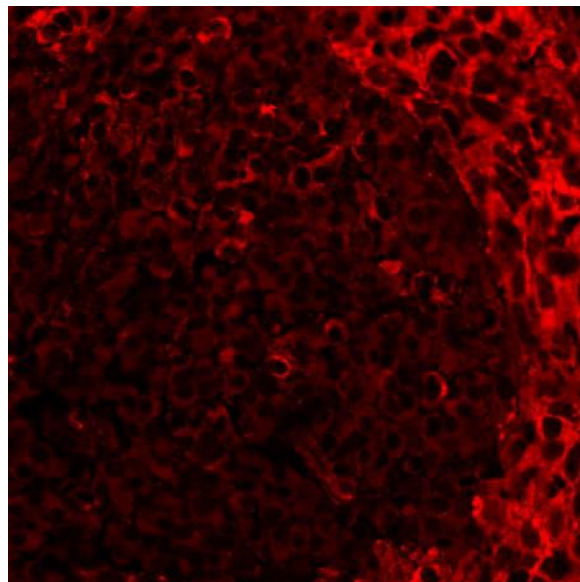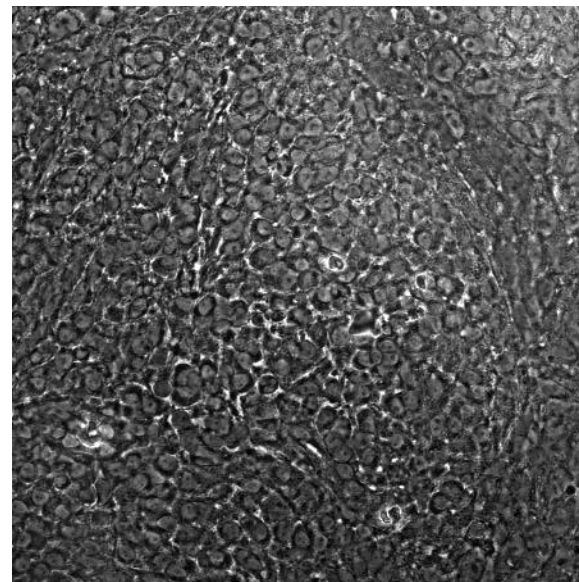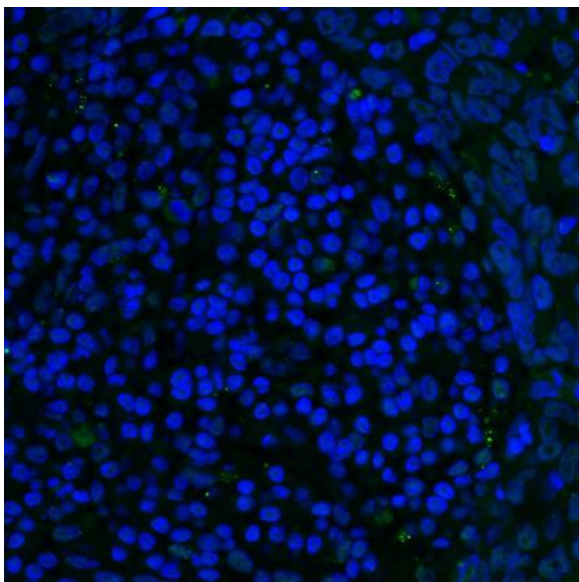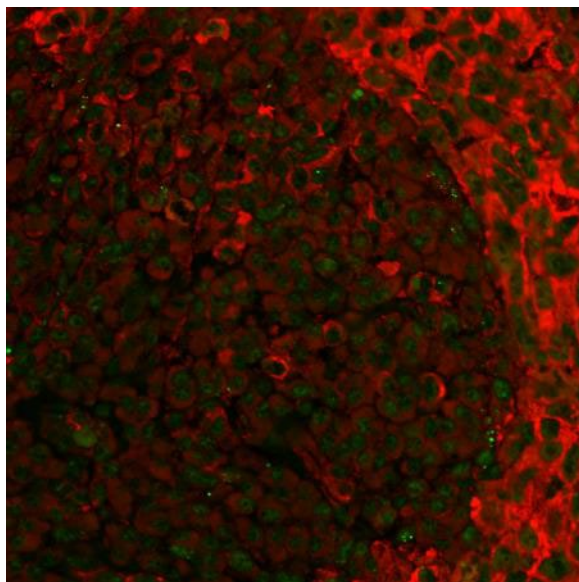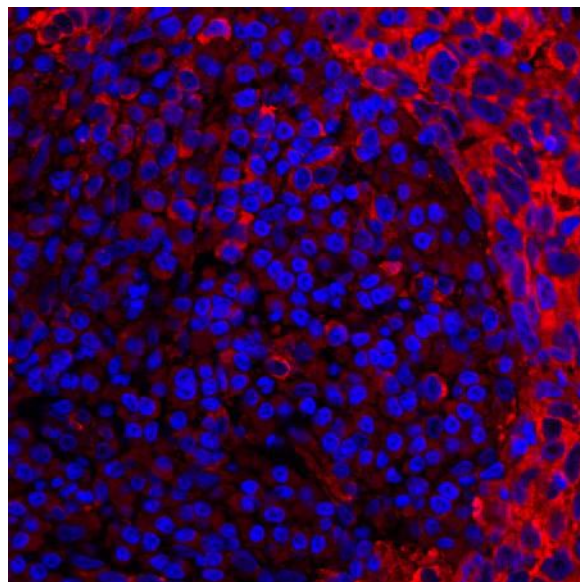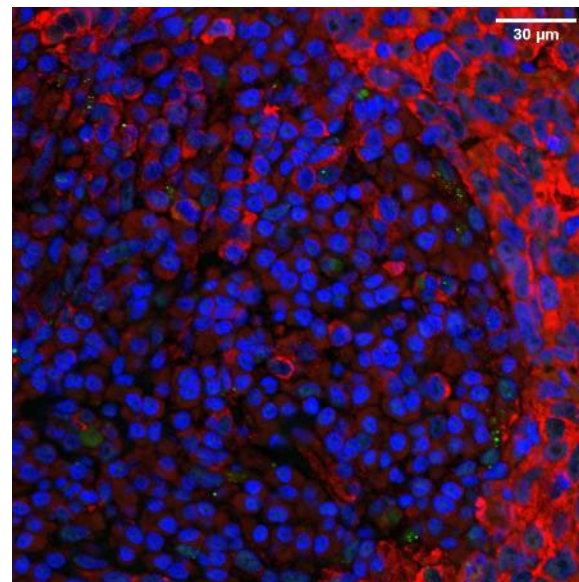

10560\_00

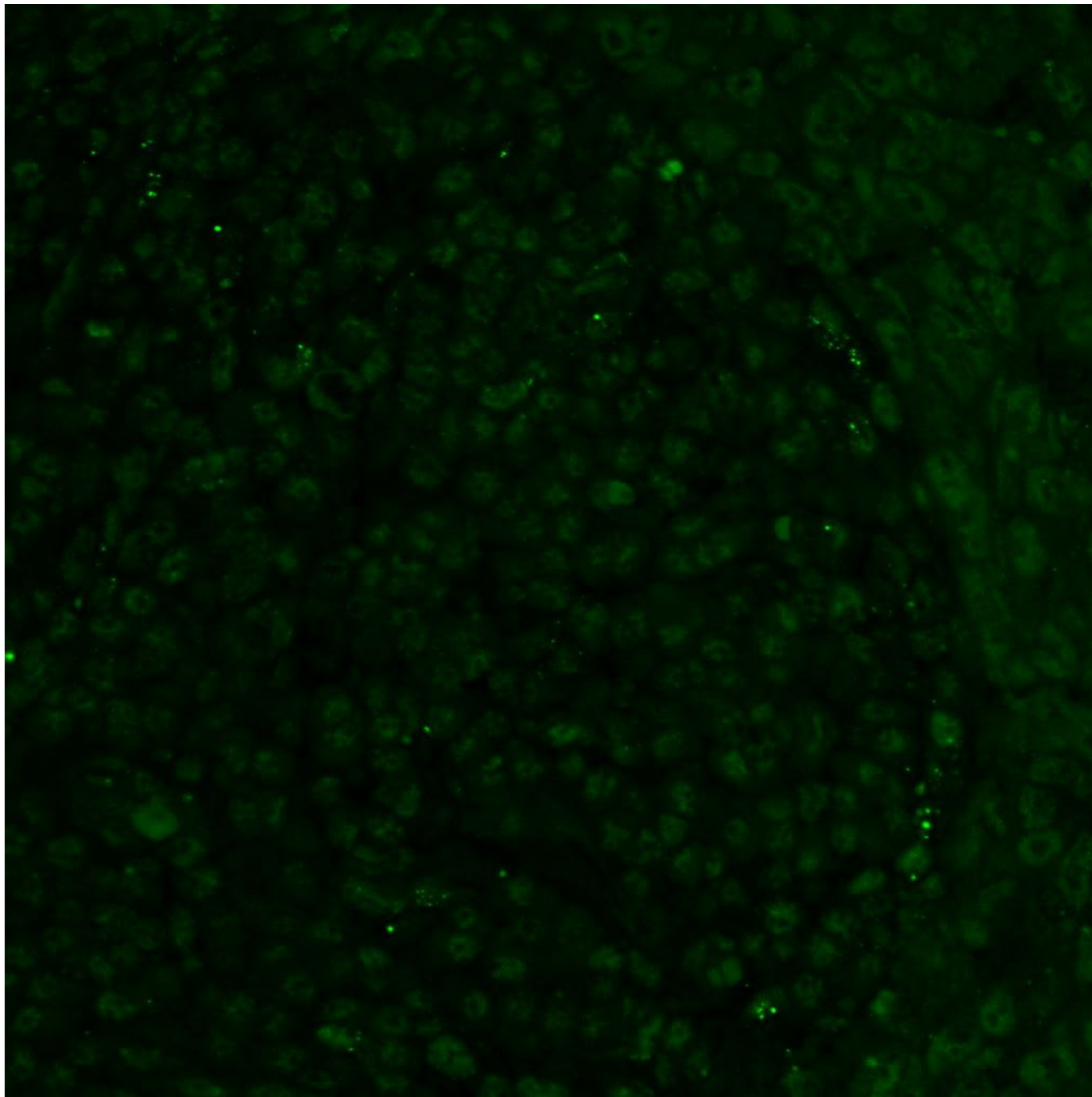

10560\_00

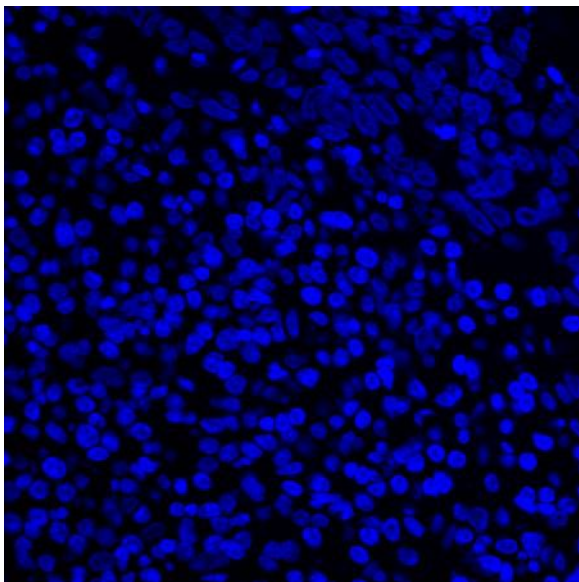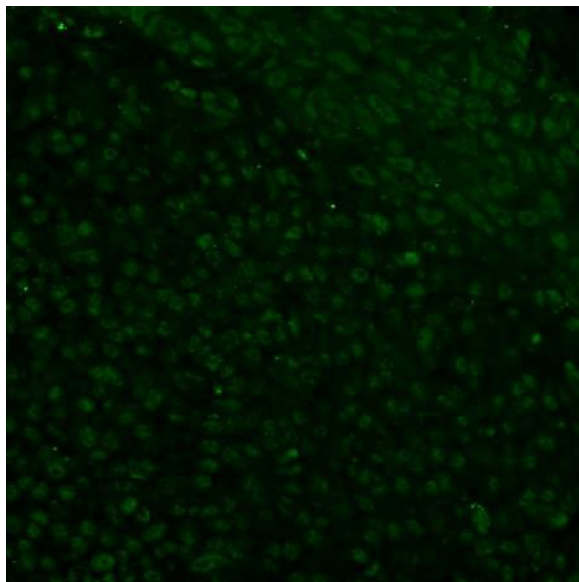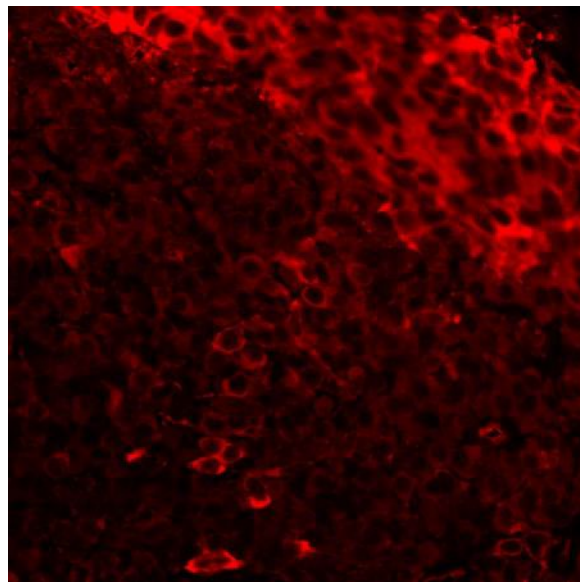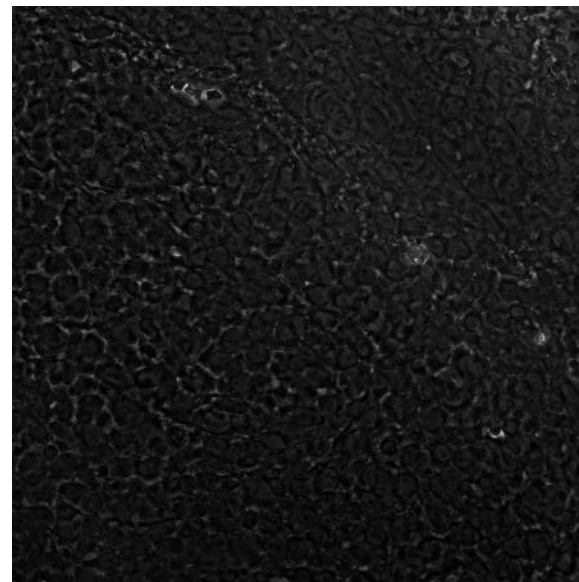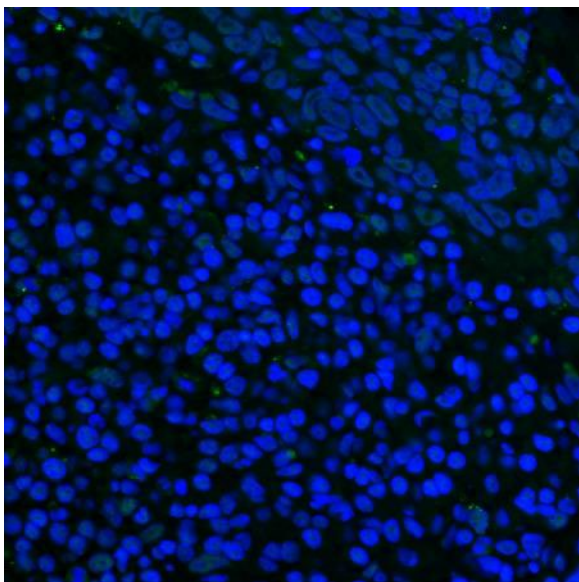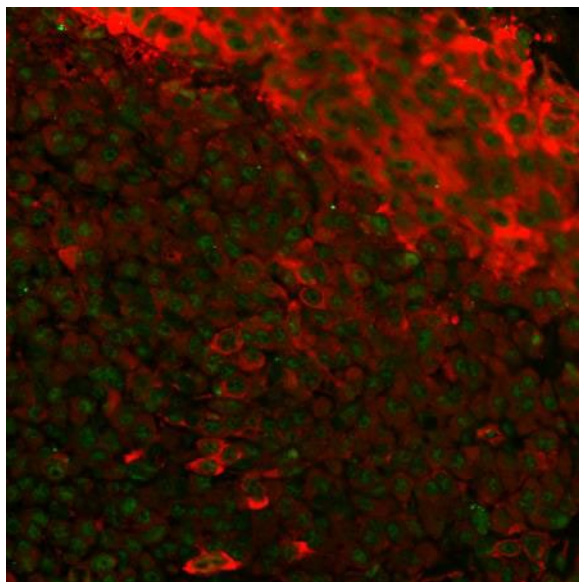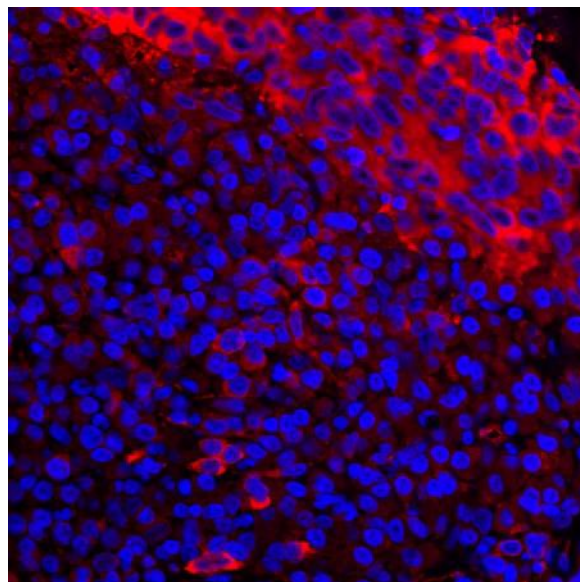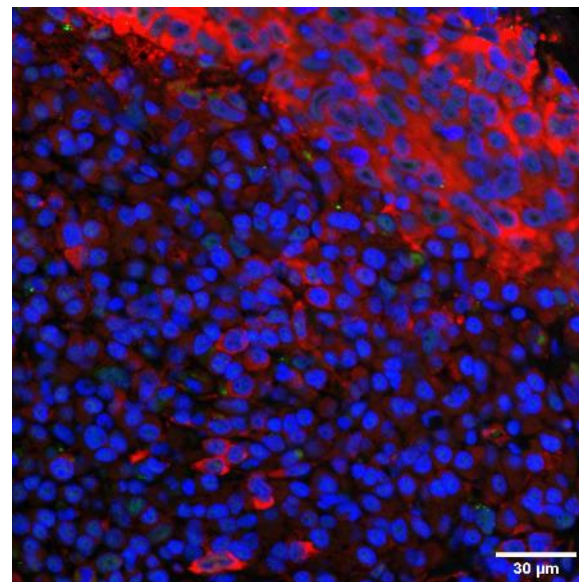

10560\_01

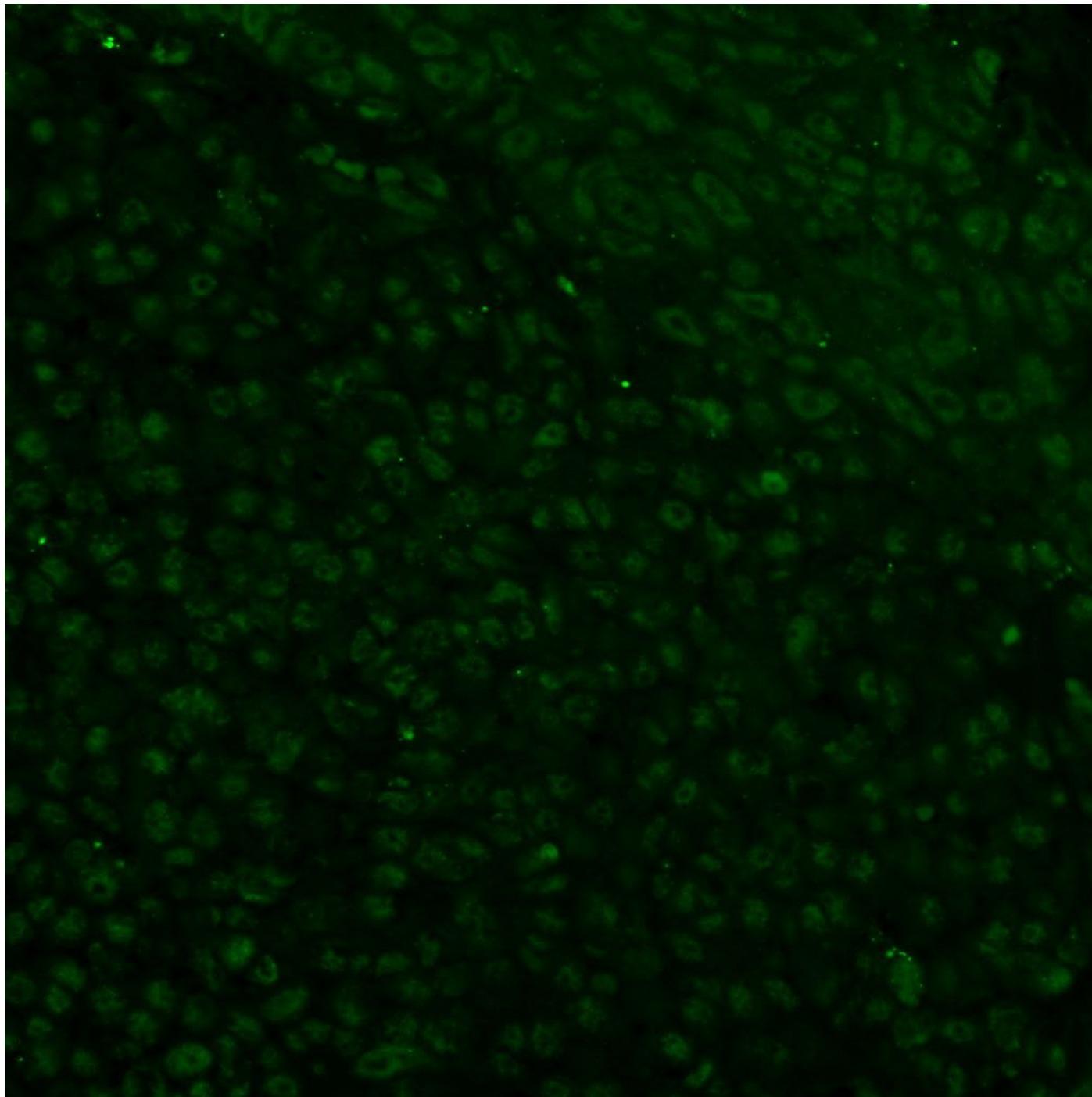

10560\_01

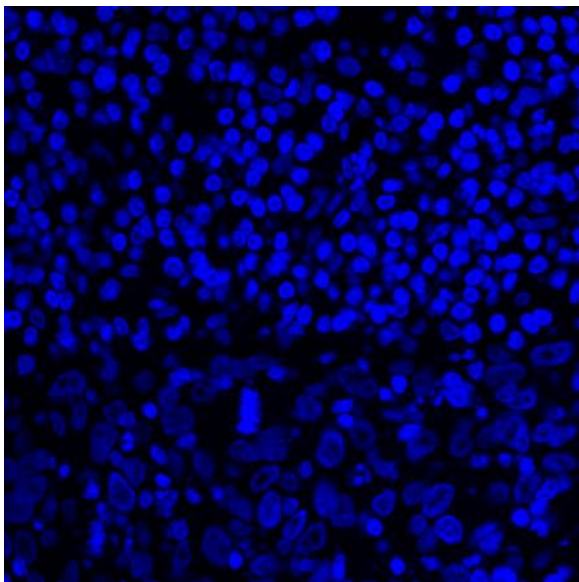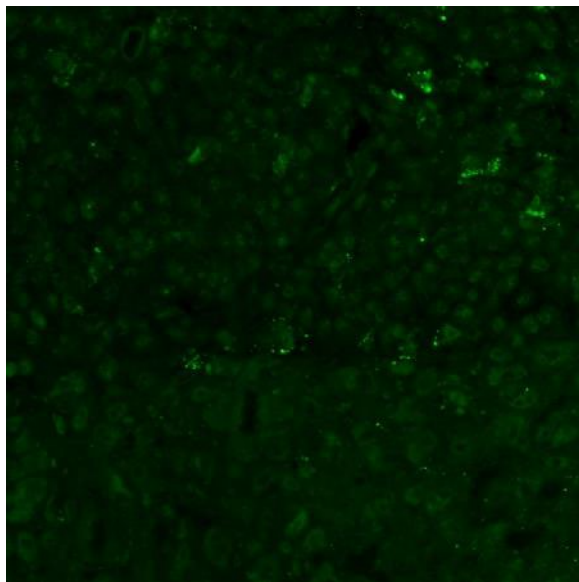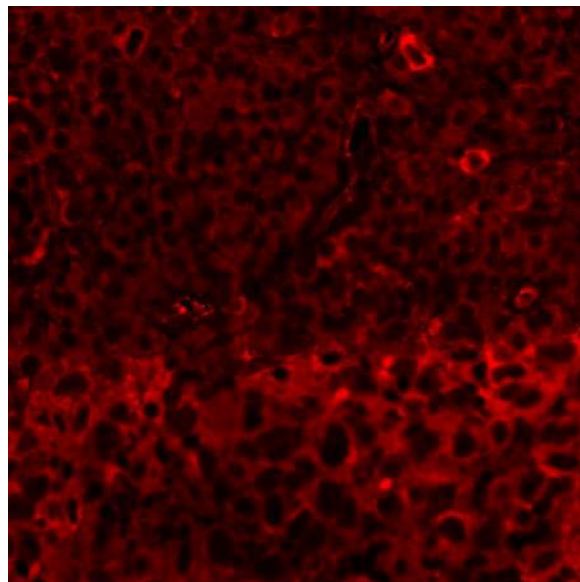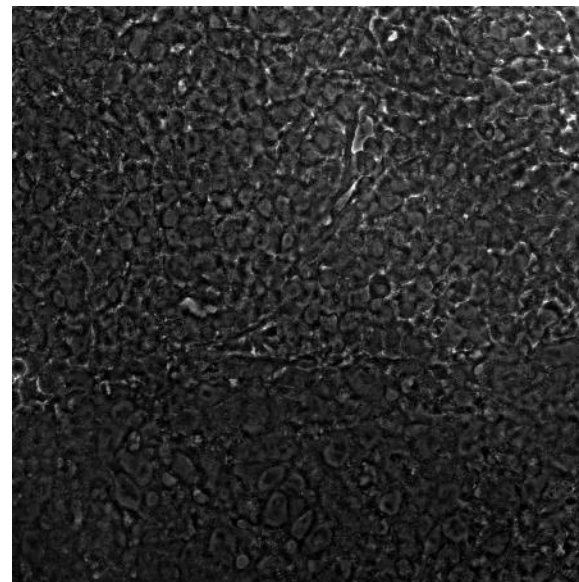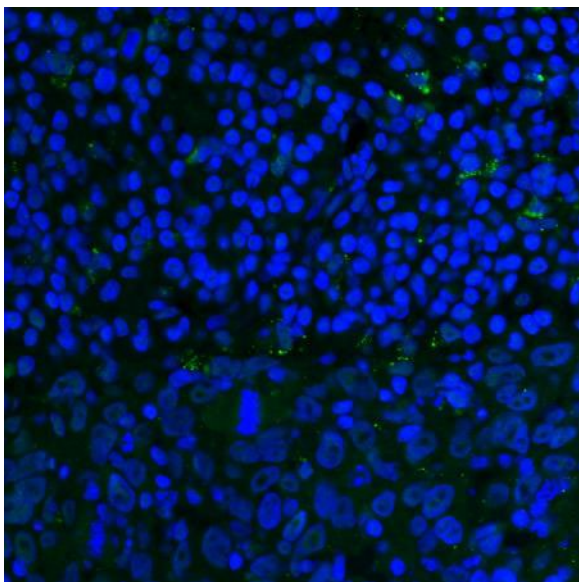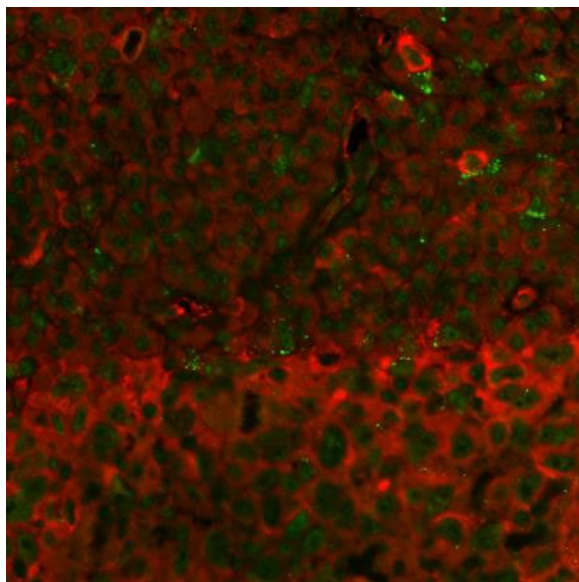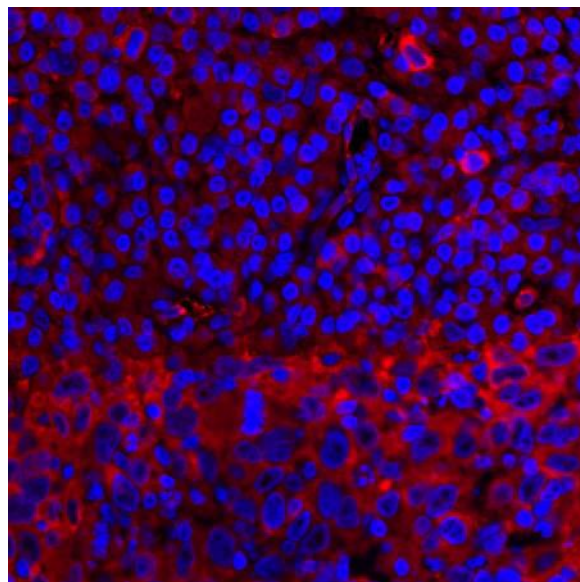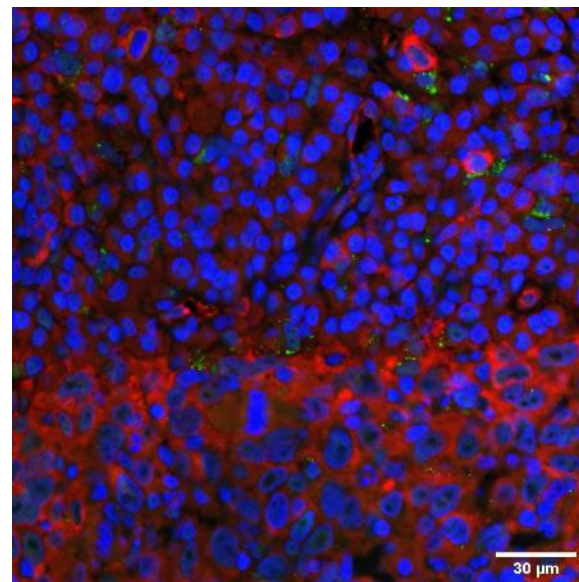

10560\_02

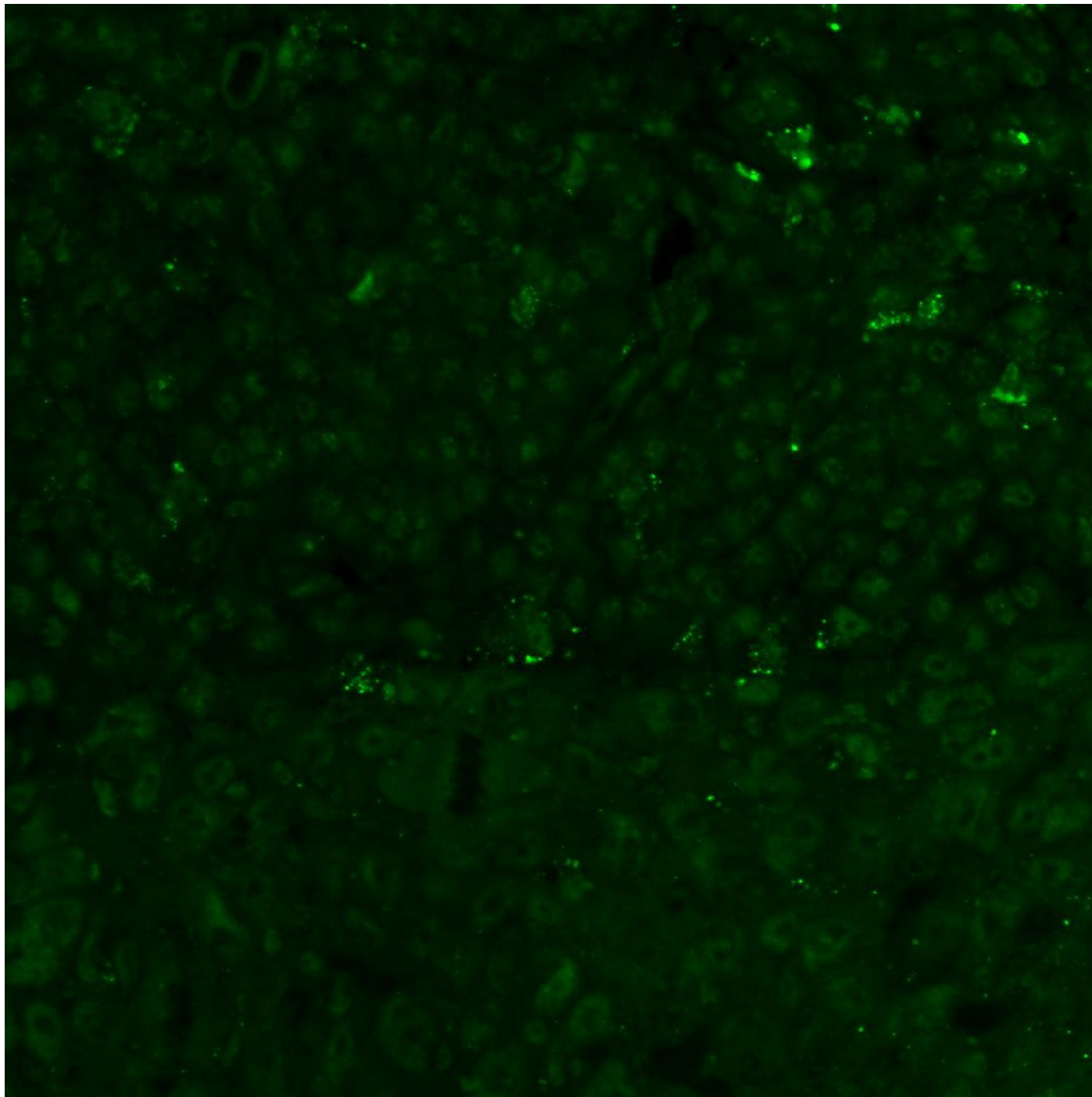

10560\_02

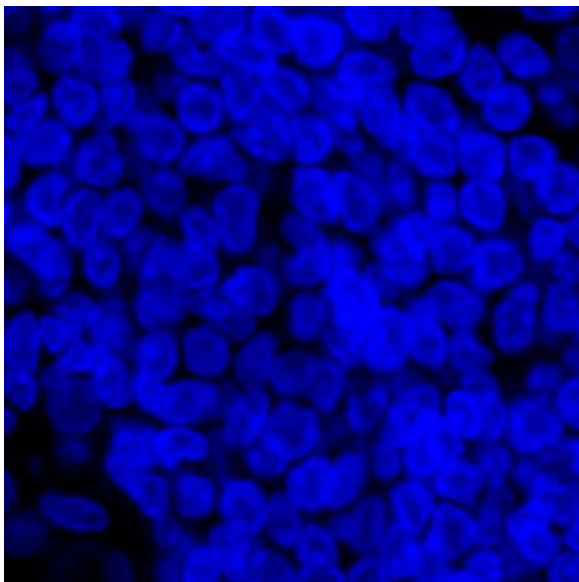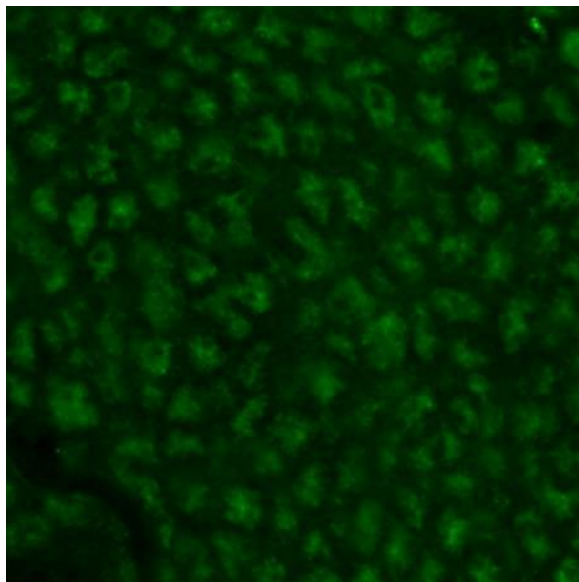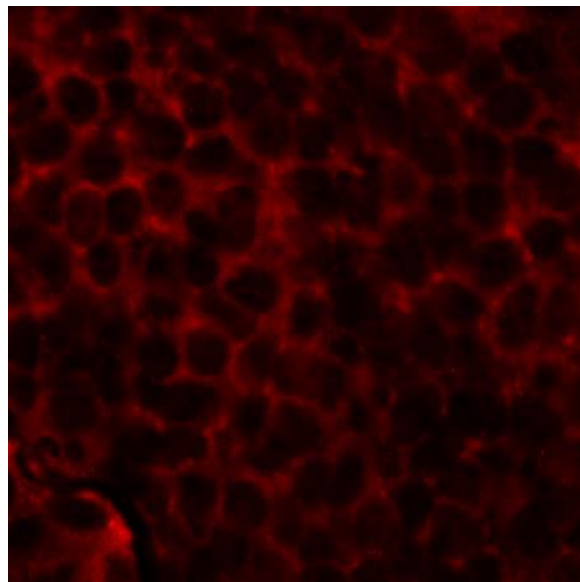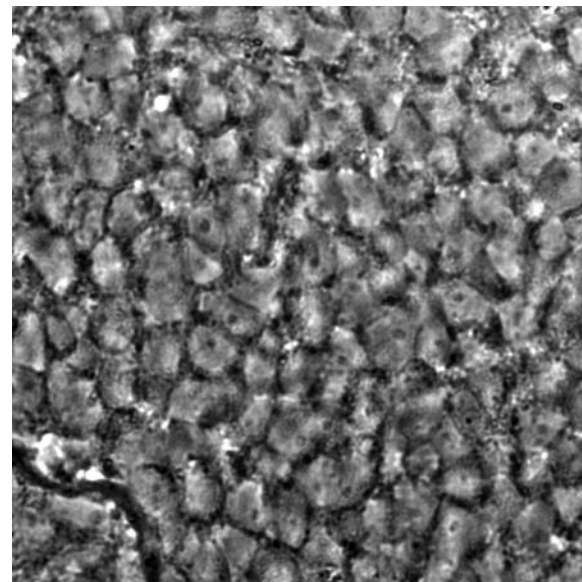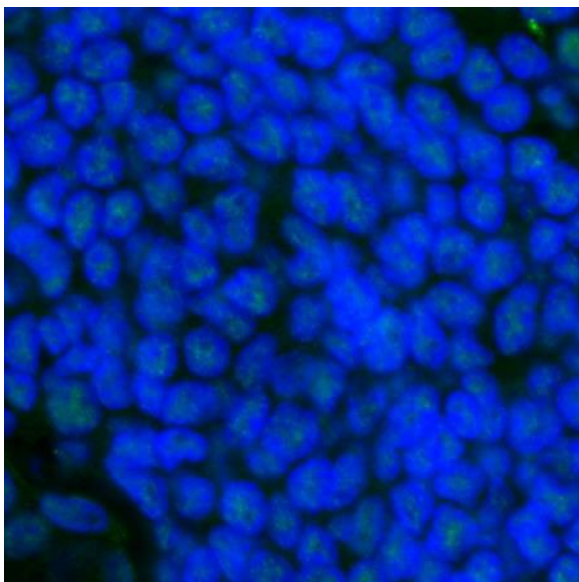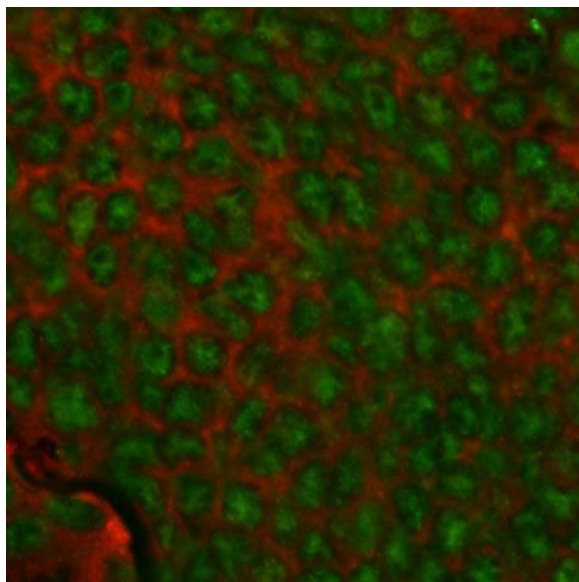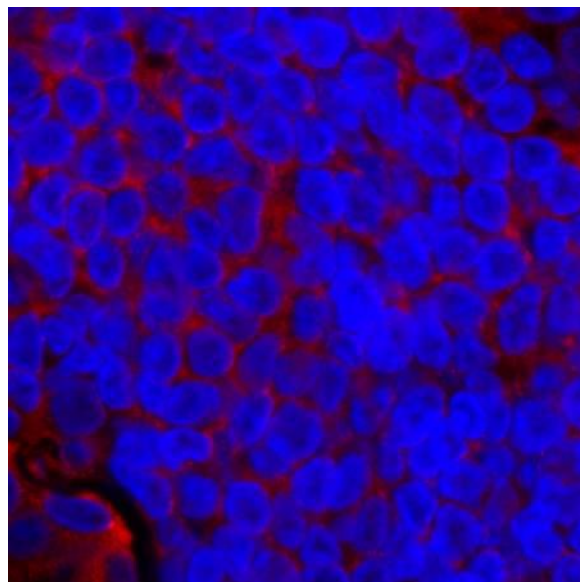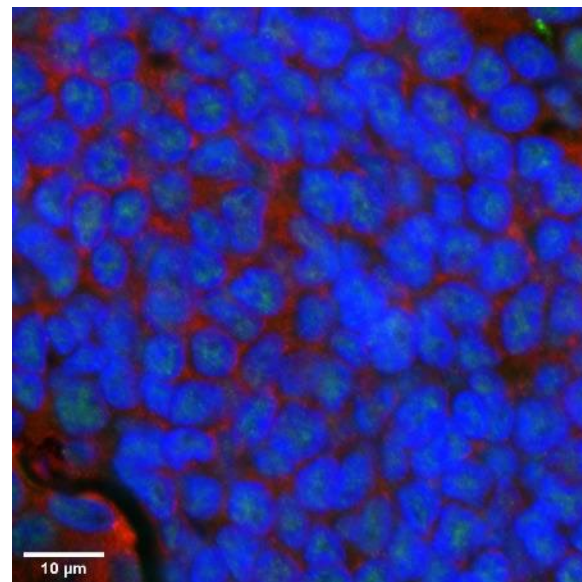

10560\_03

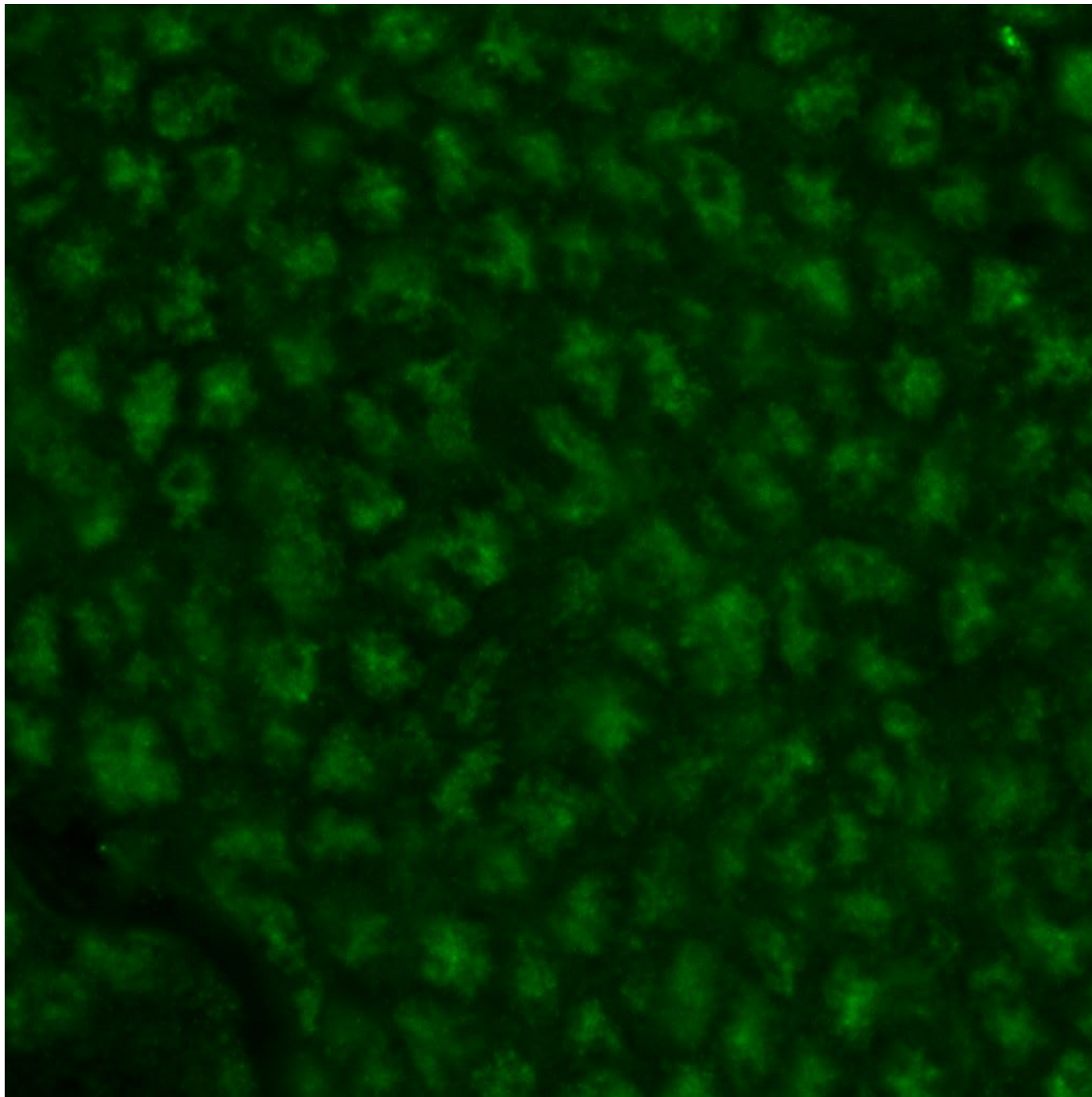

10560\_03

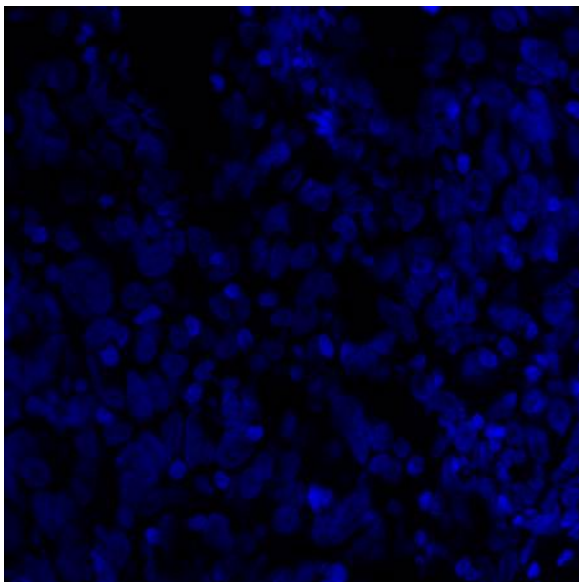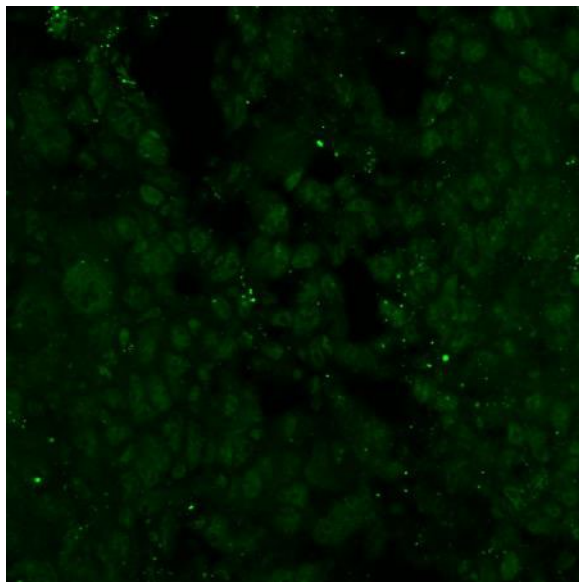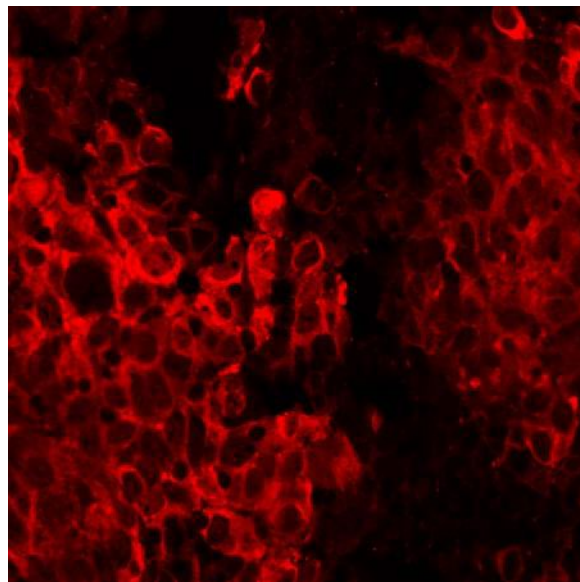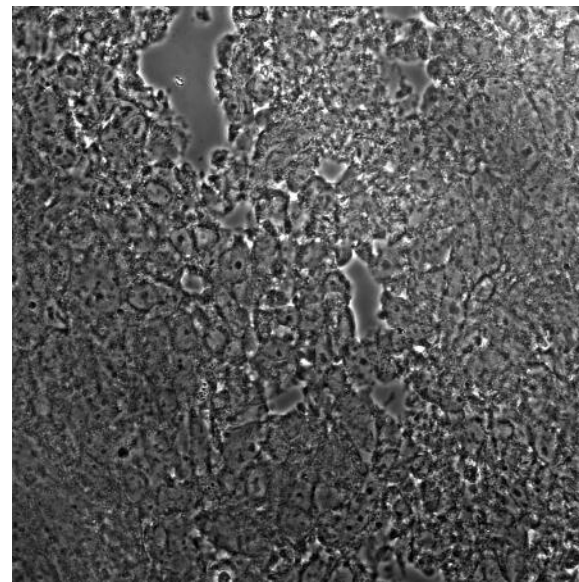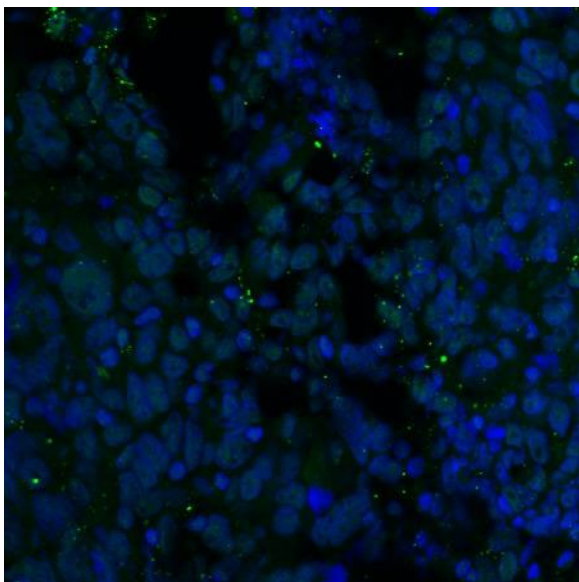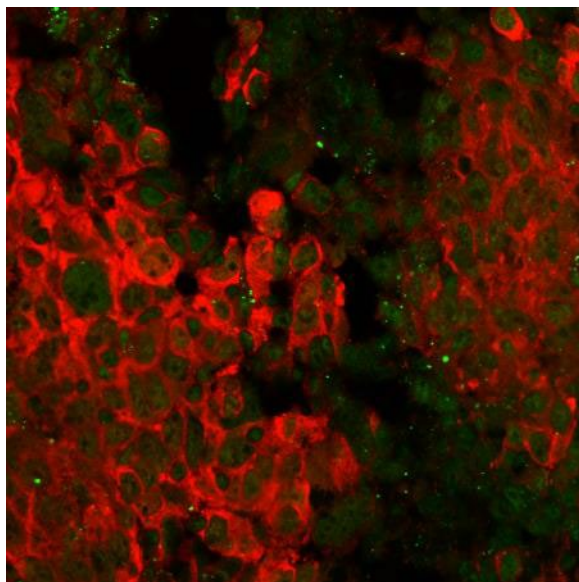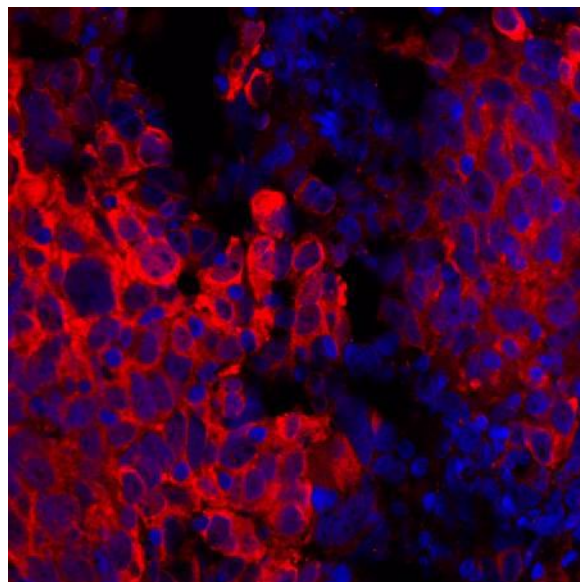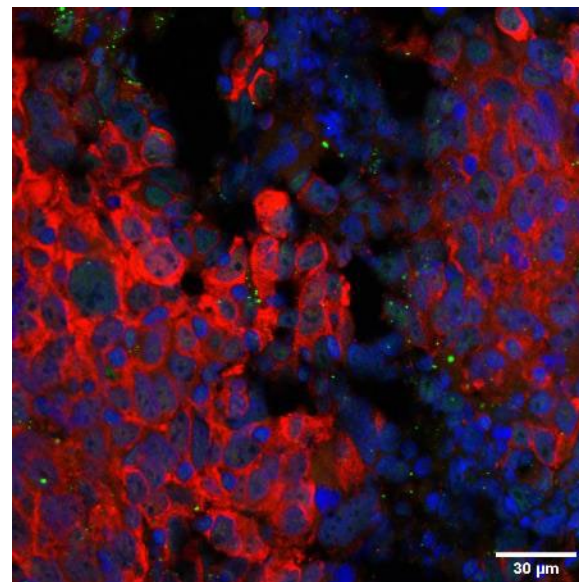

10560\_04

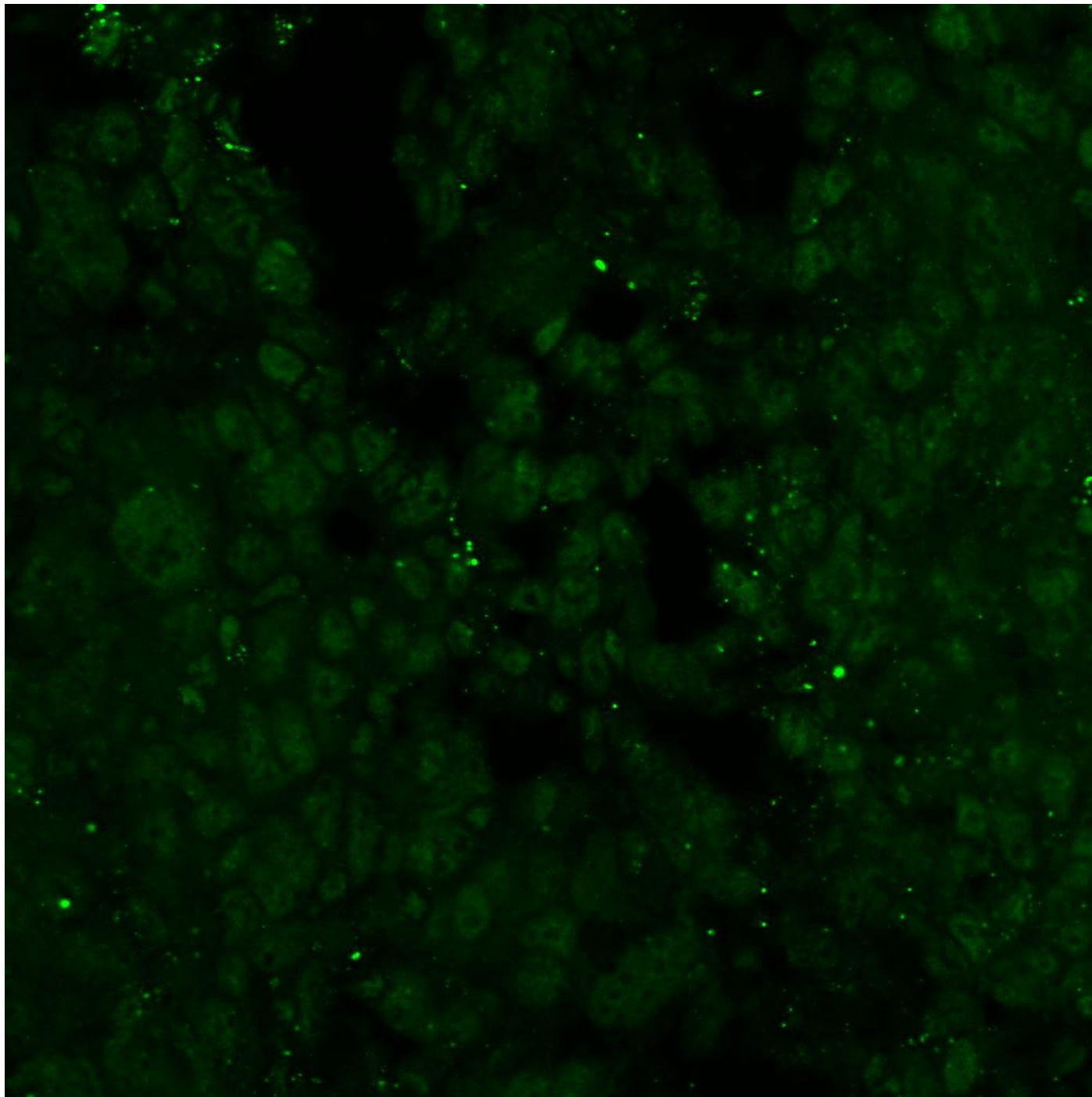

10560\_04

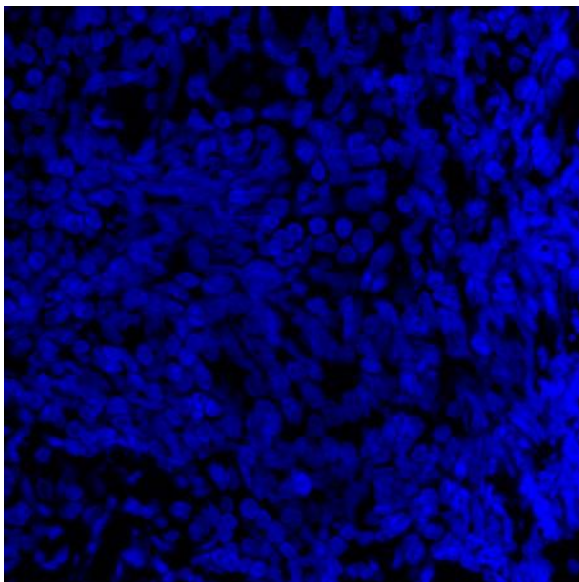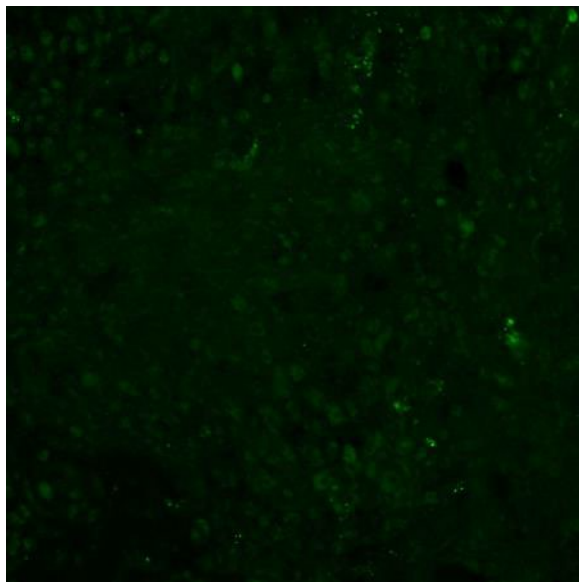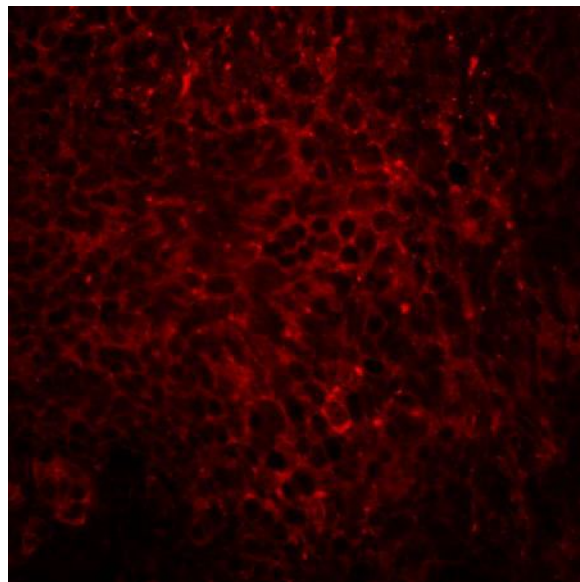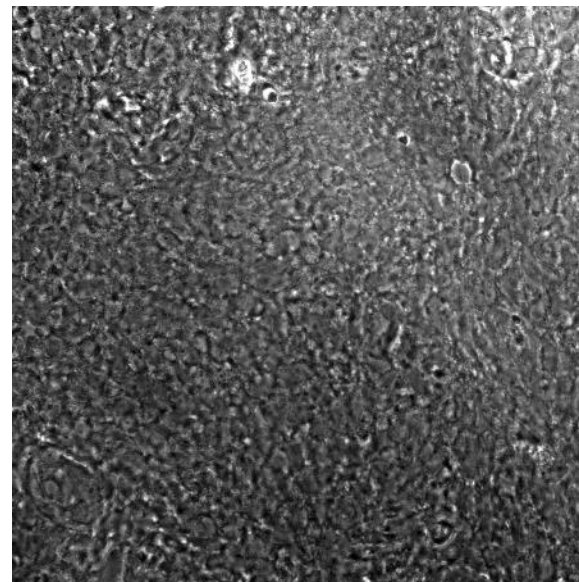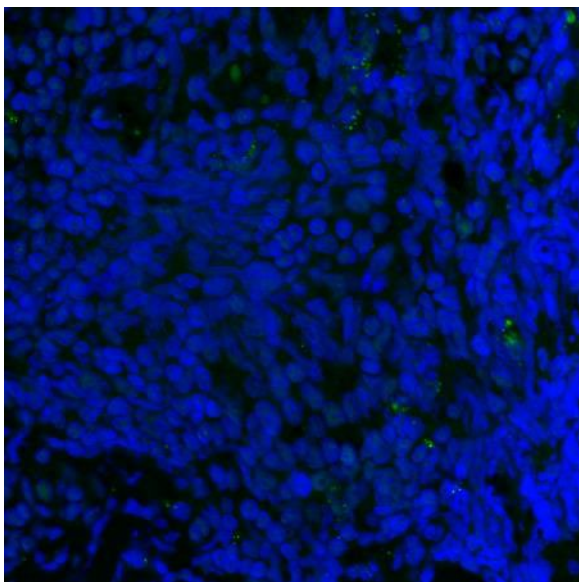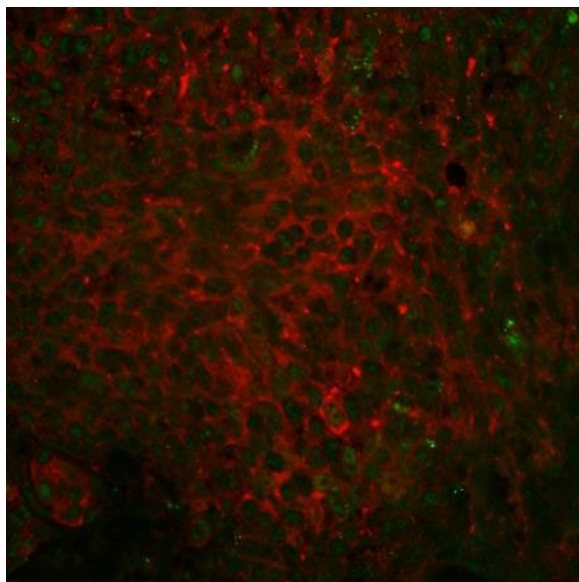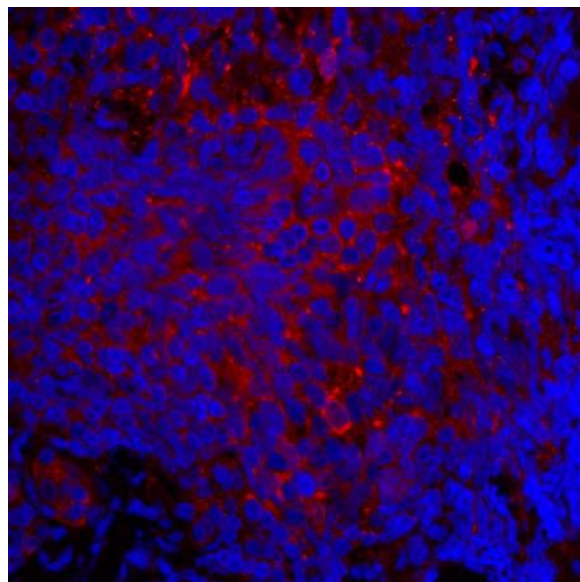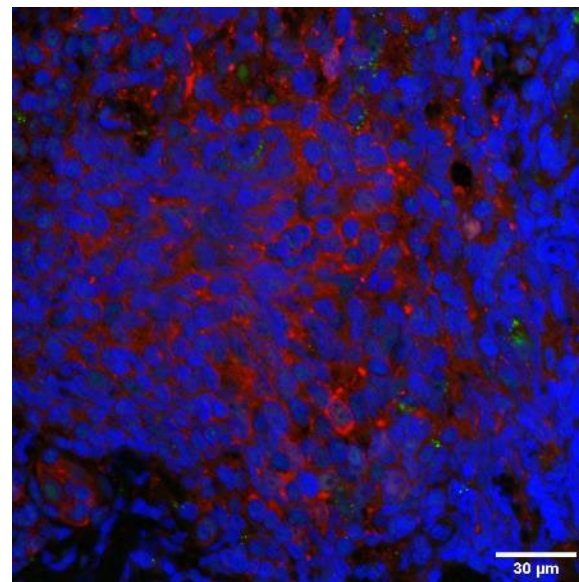

10560\_05

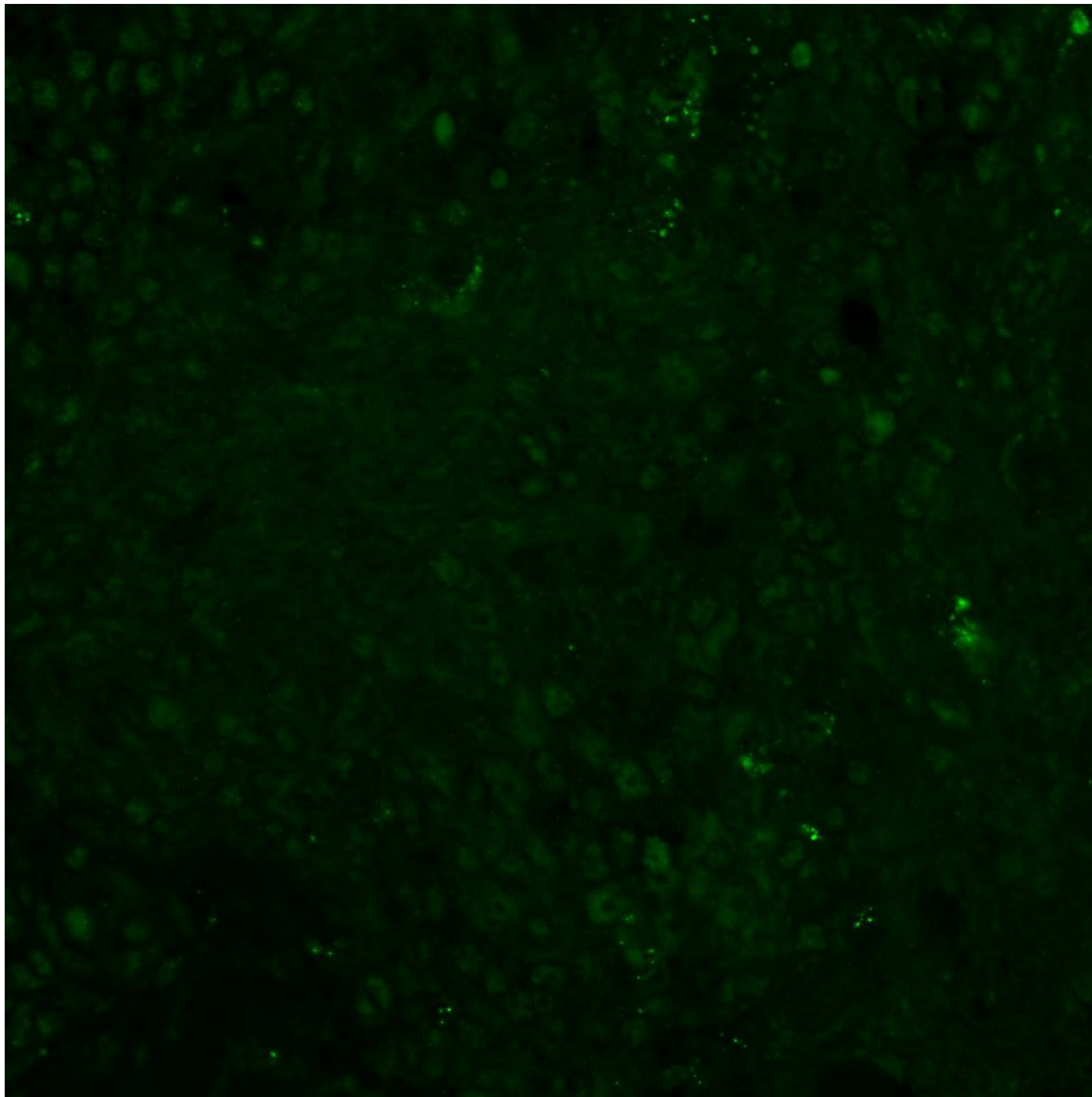

10560\_05

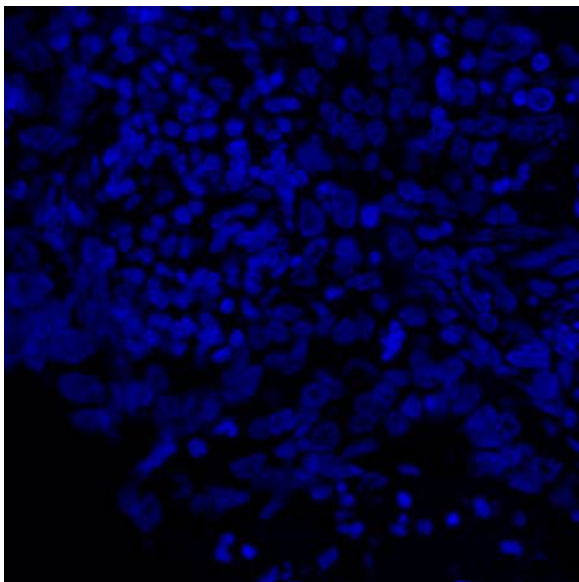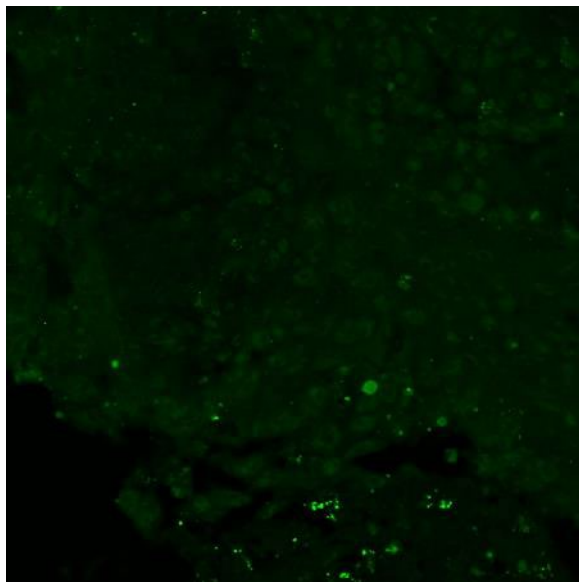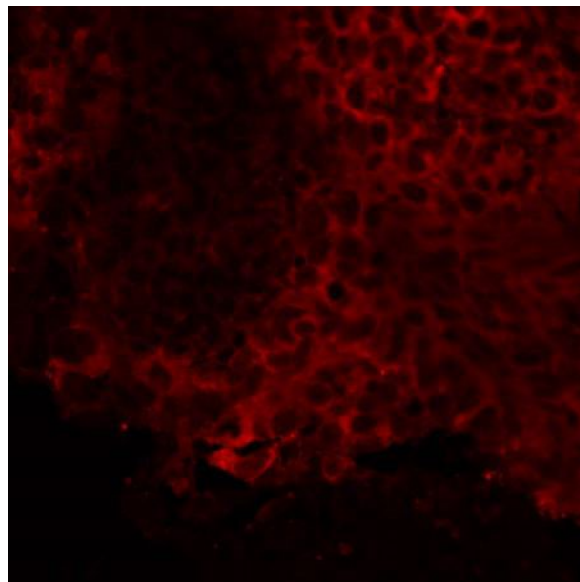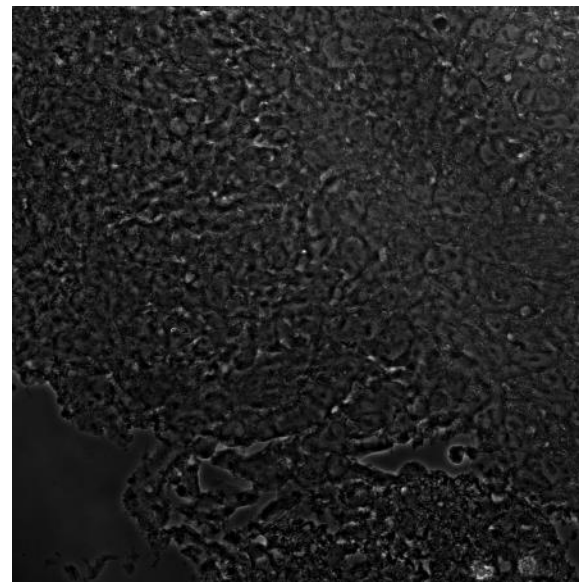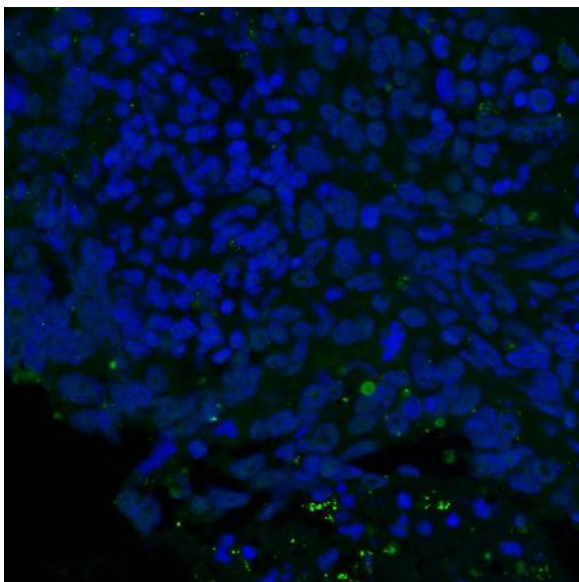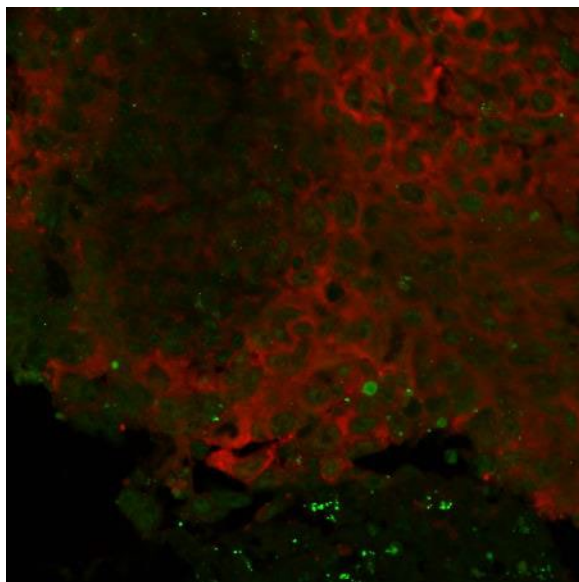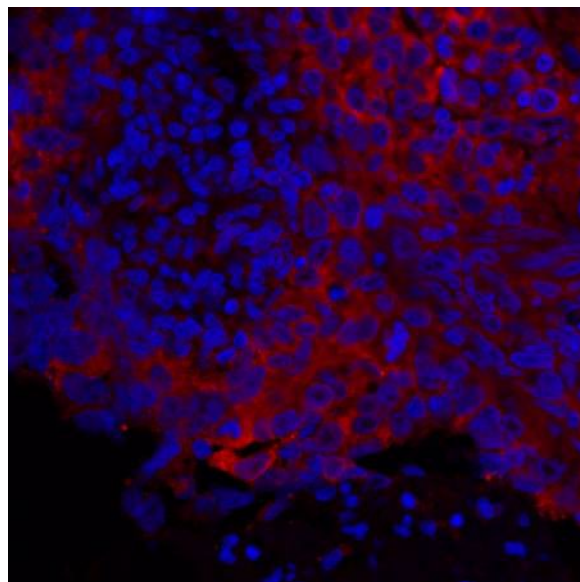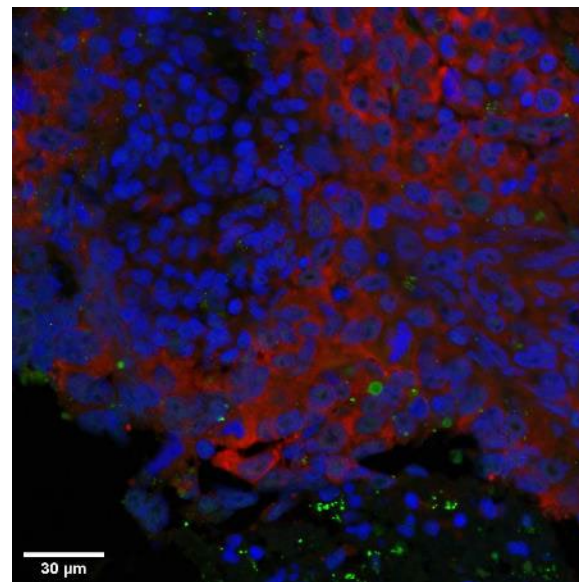

10560\_06

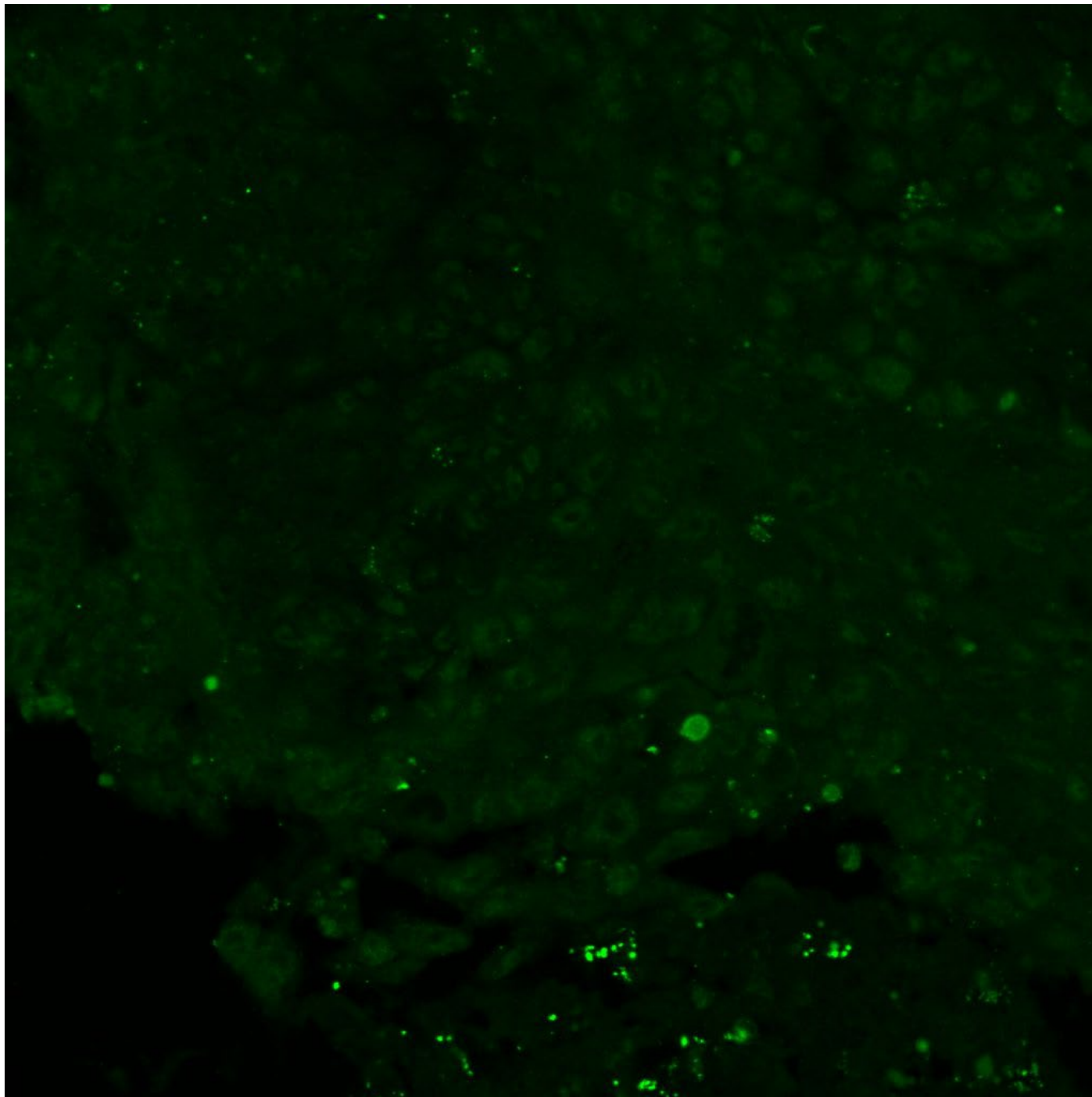

10560\_06

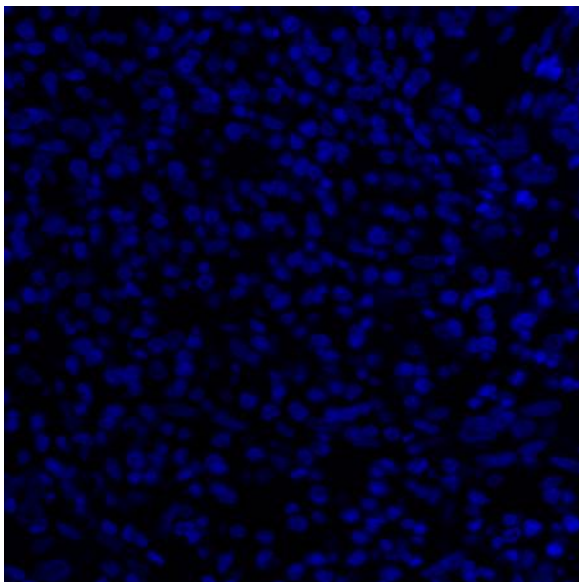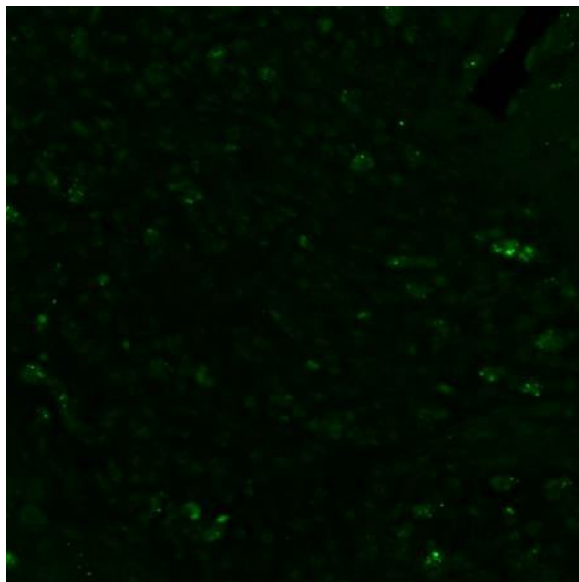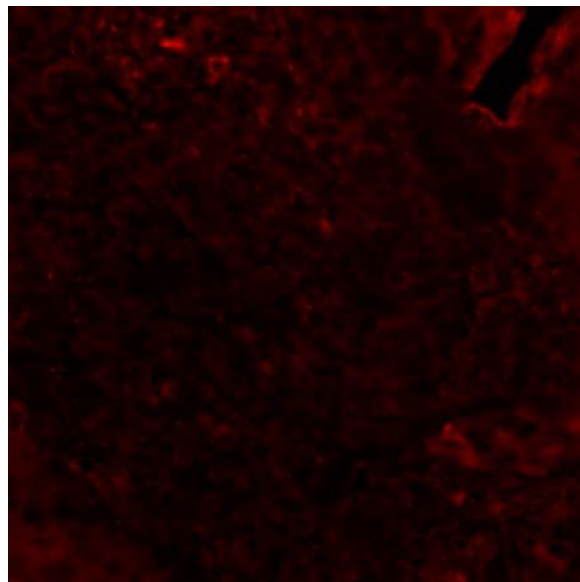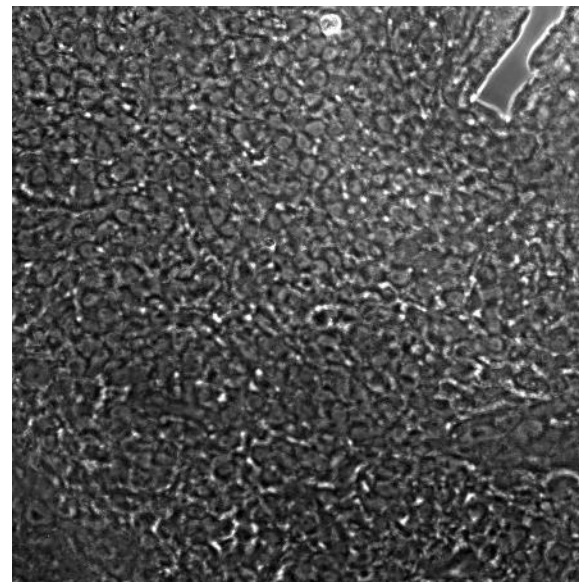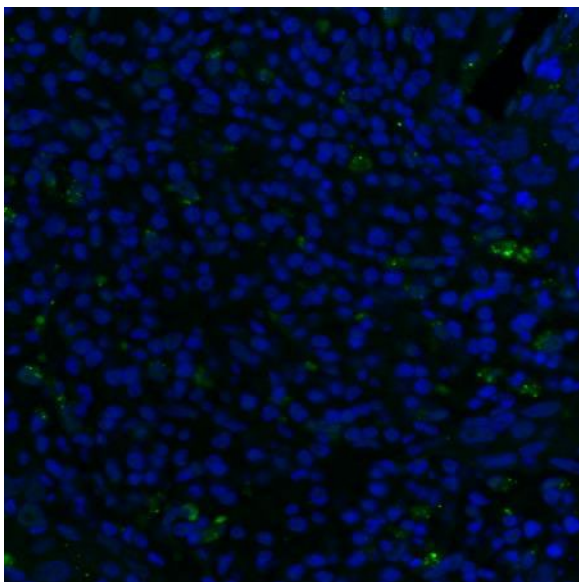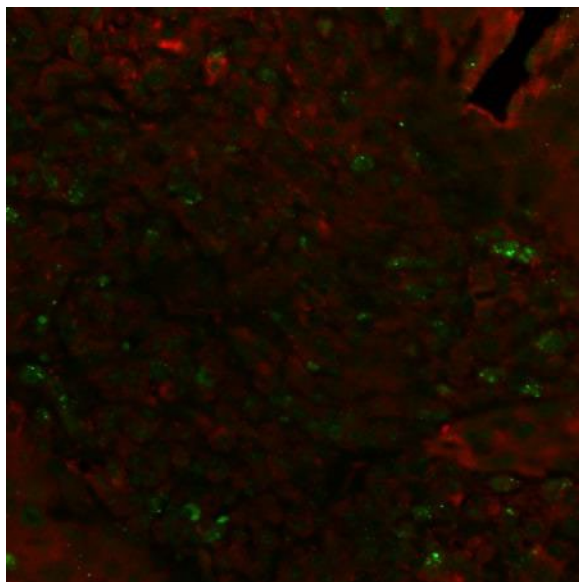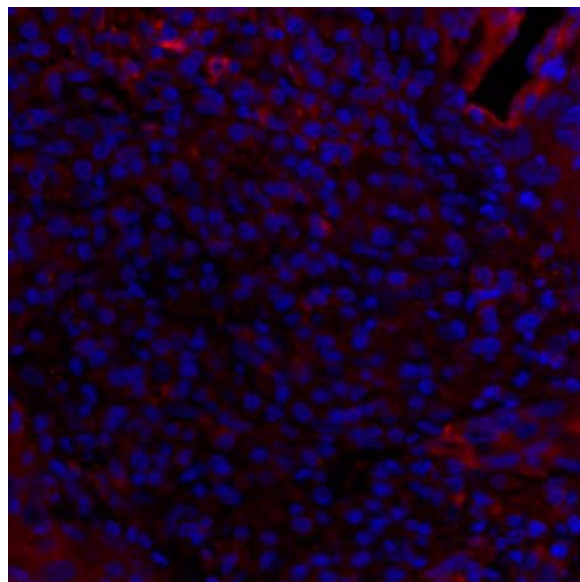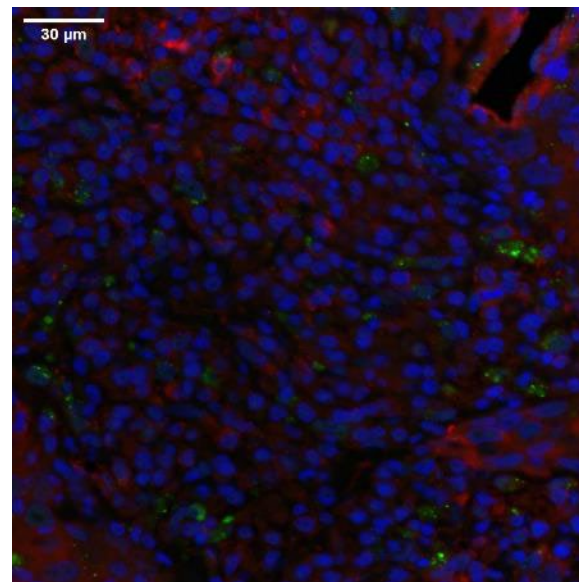

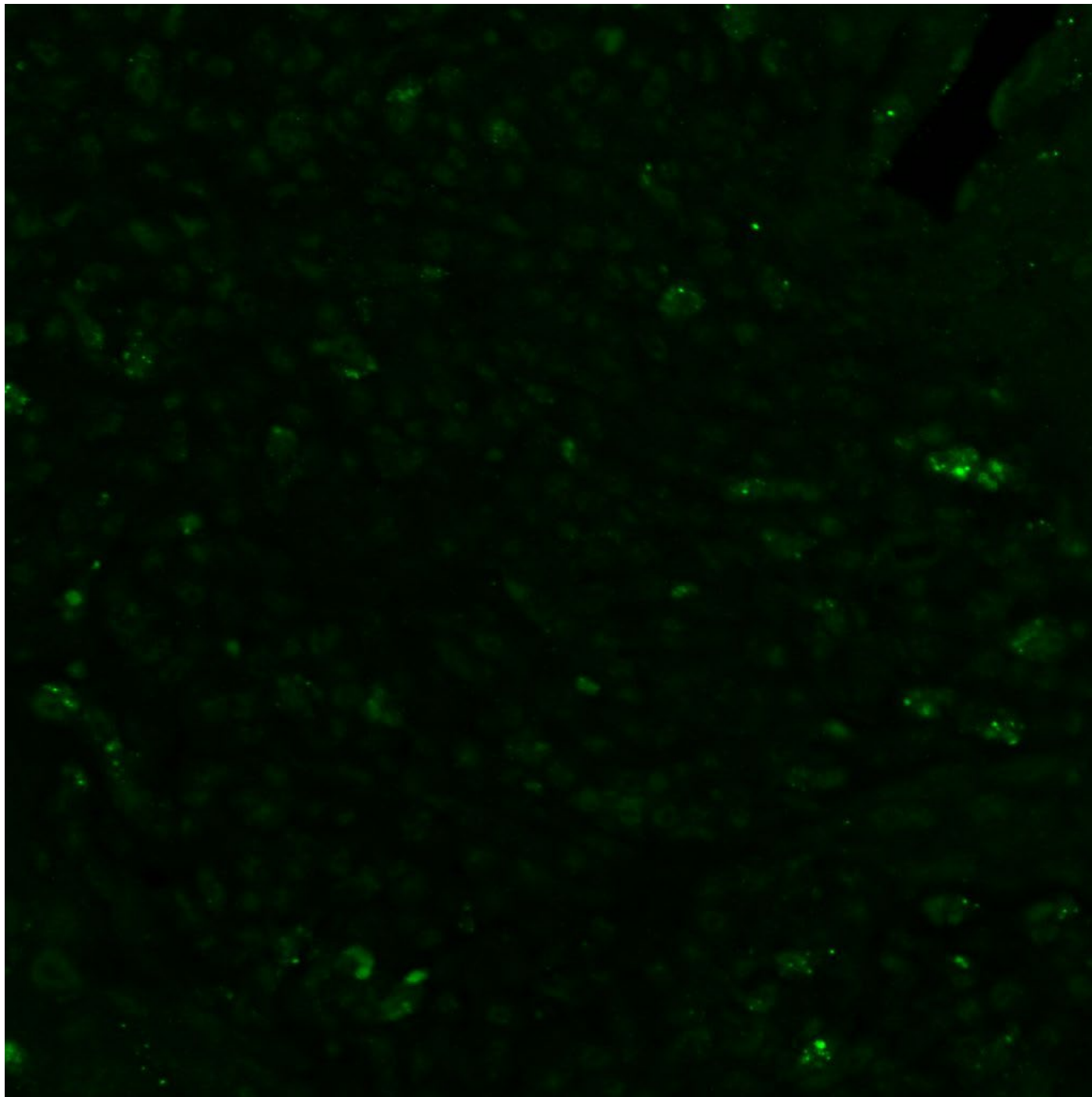

10560\_07

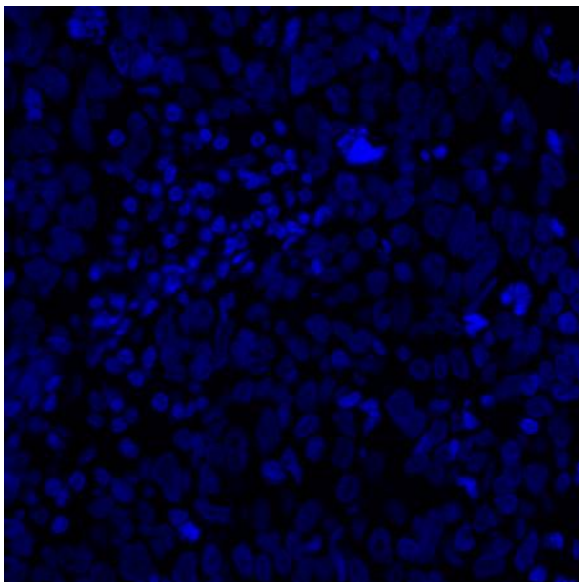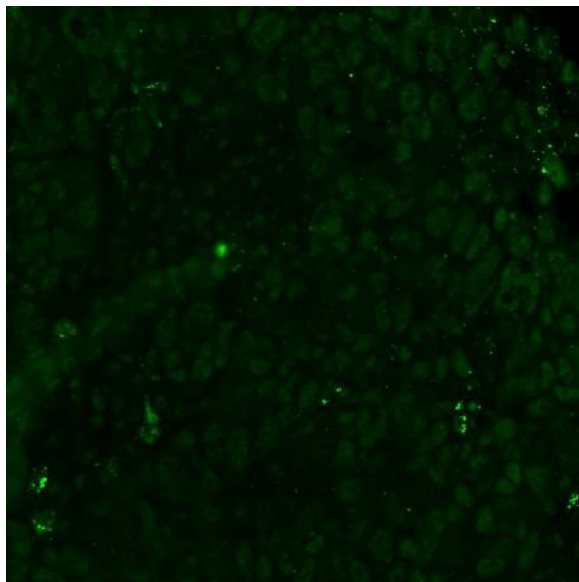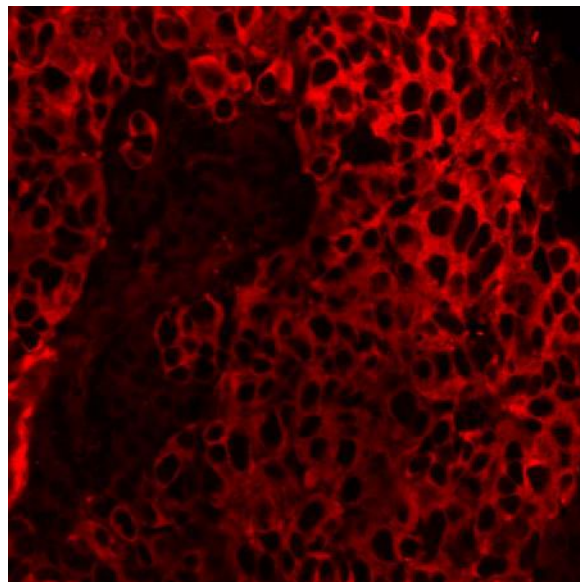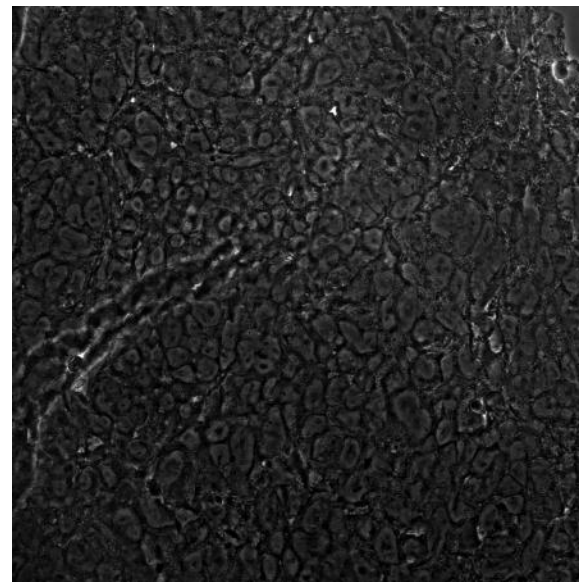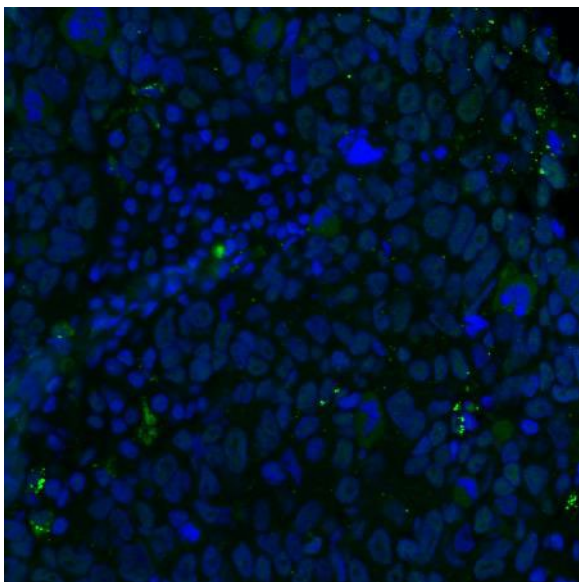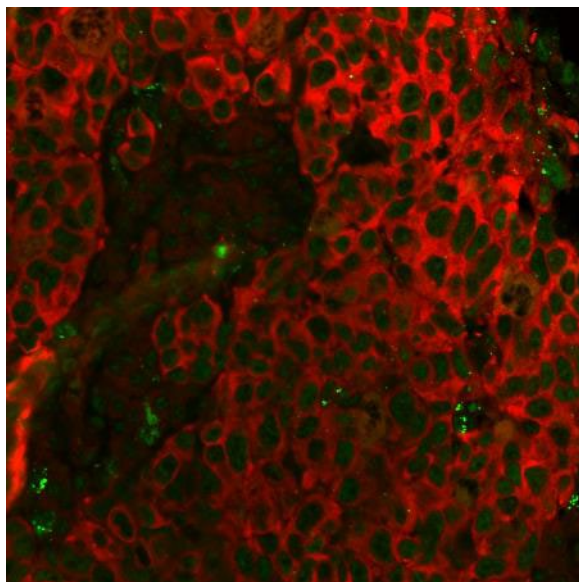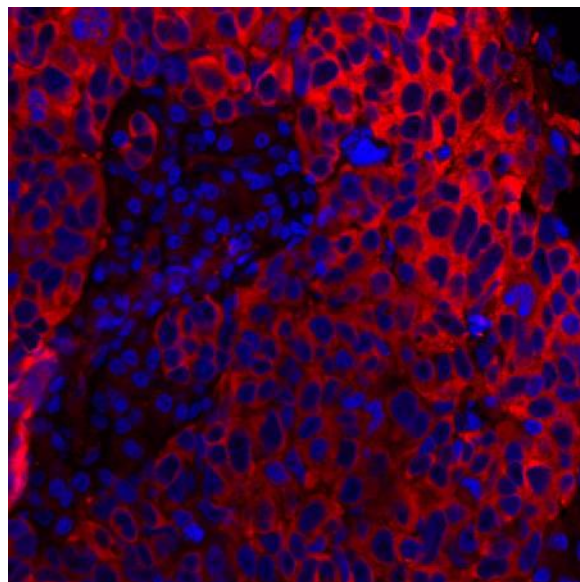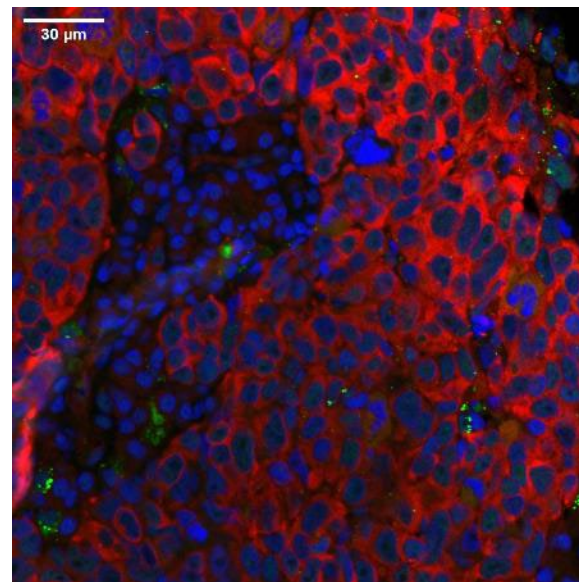

10560\_08

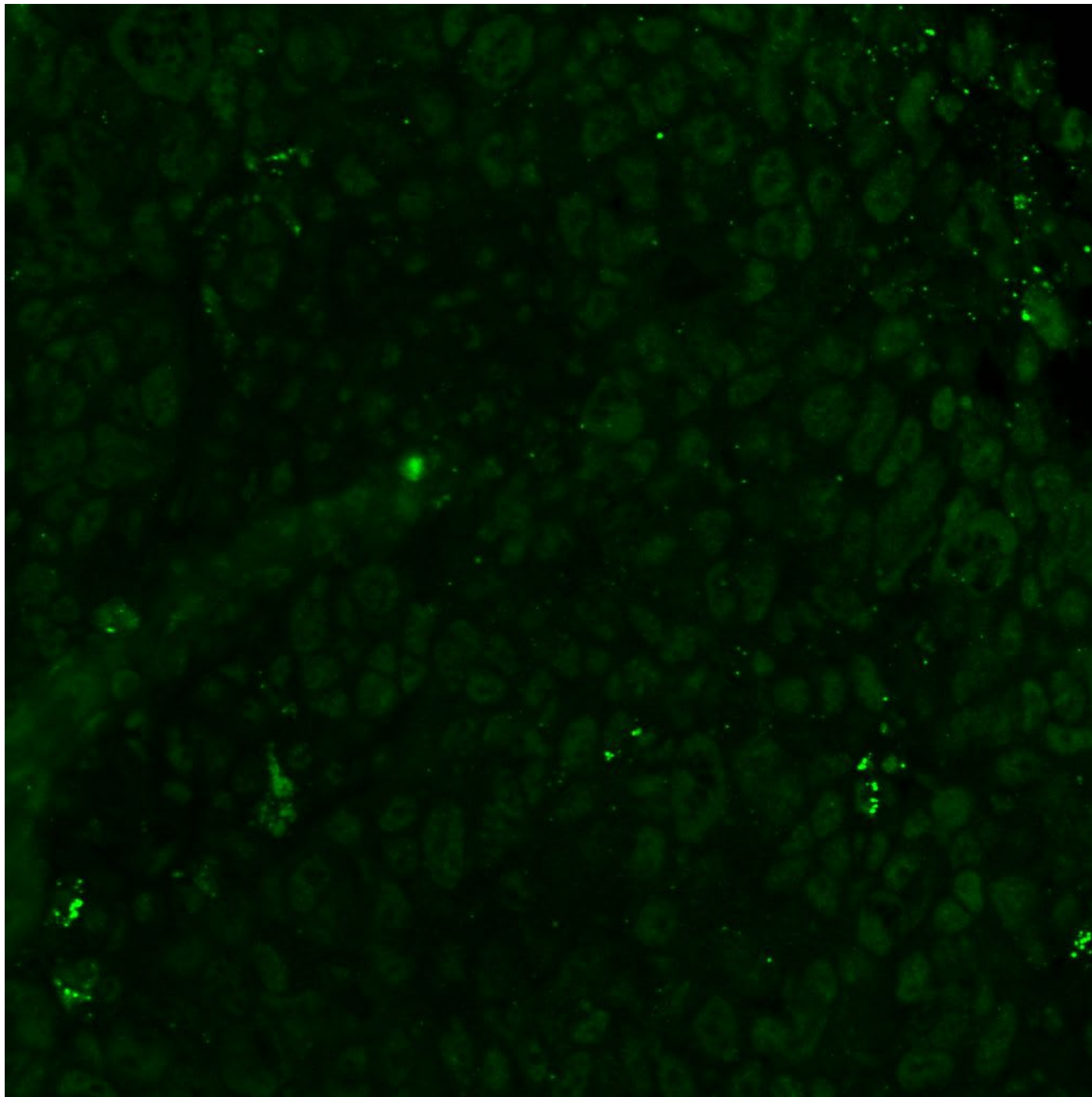

10560\_08

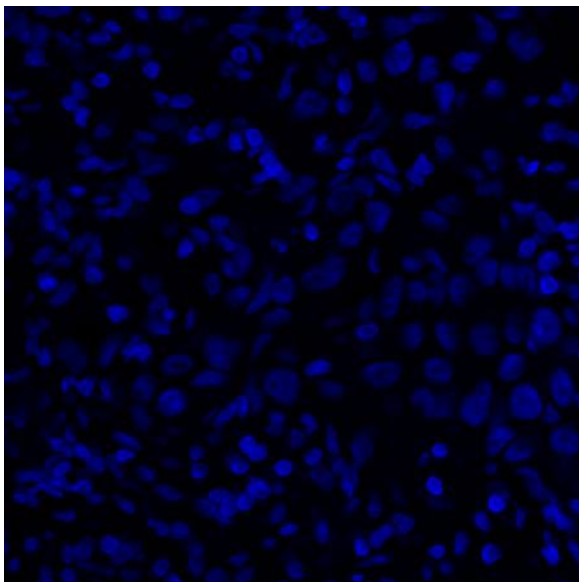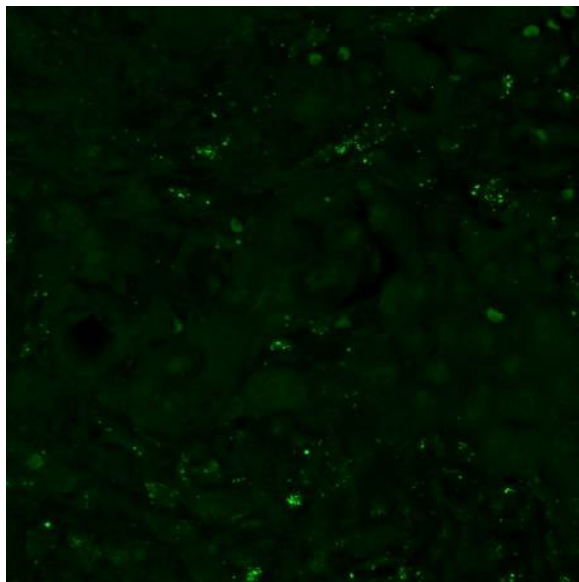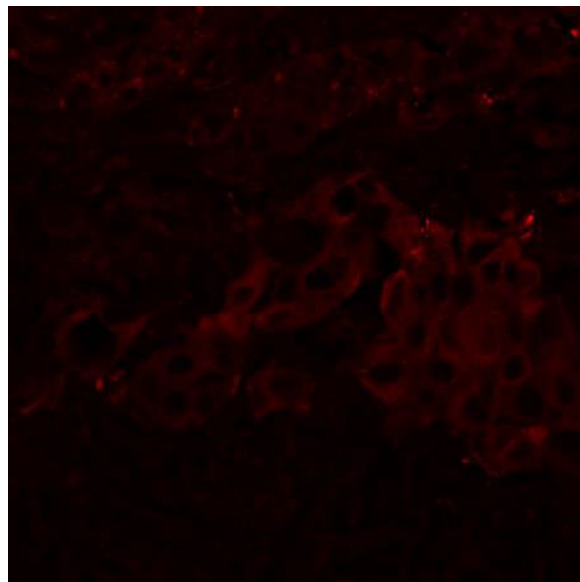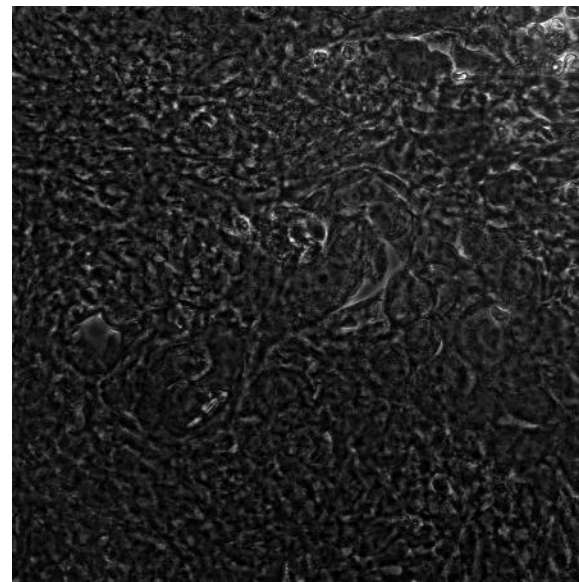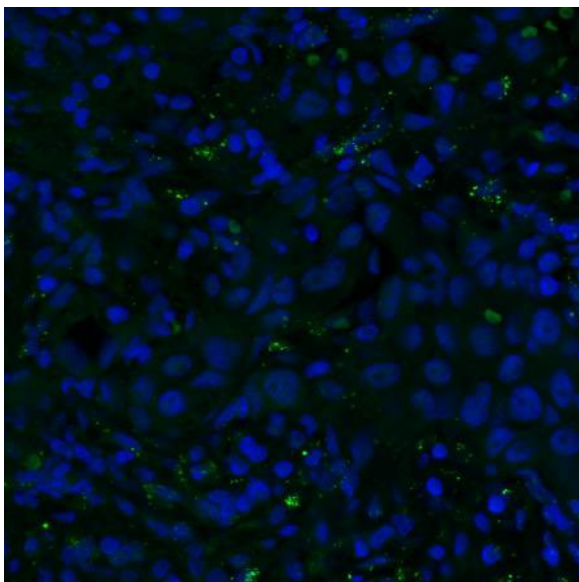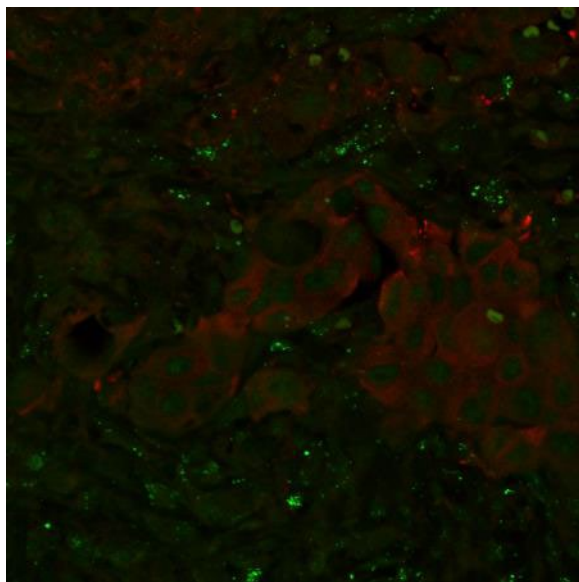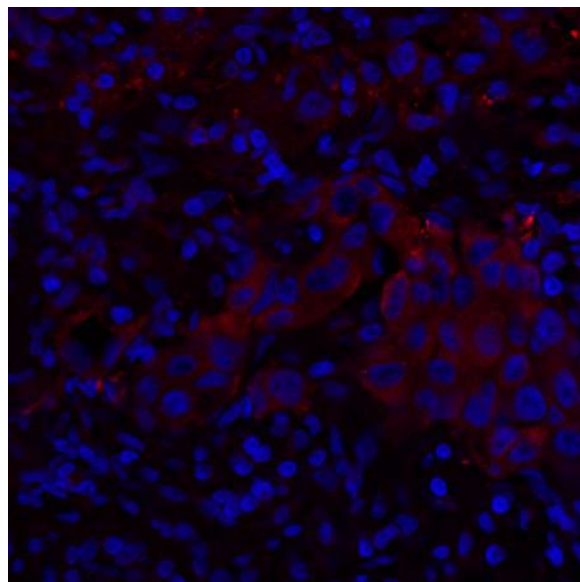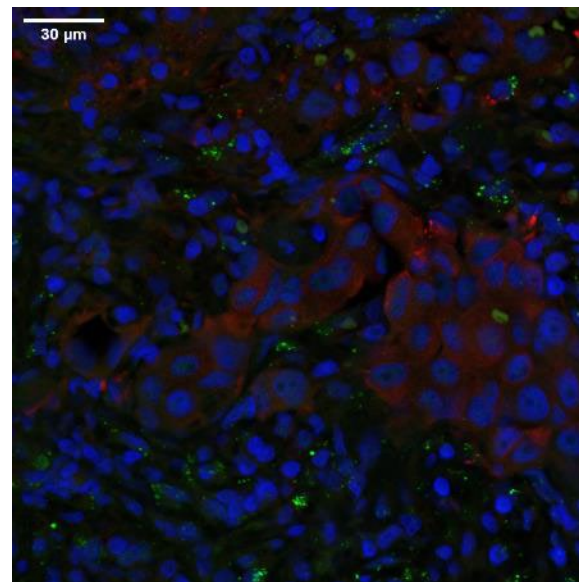

10957\_00

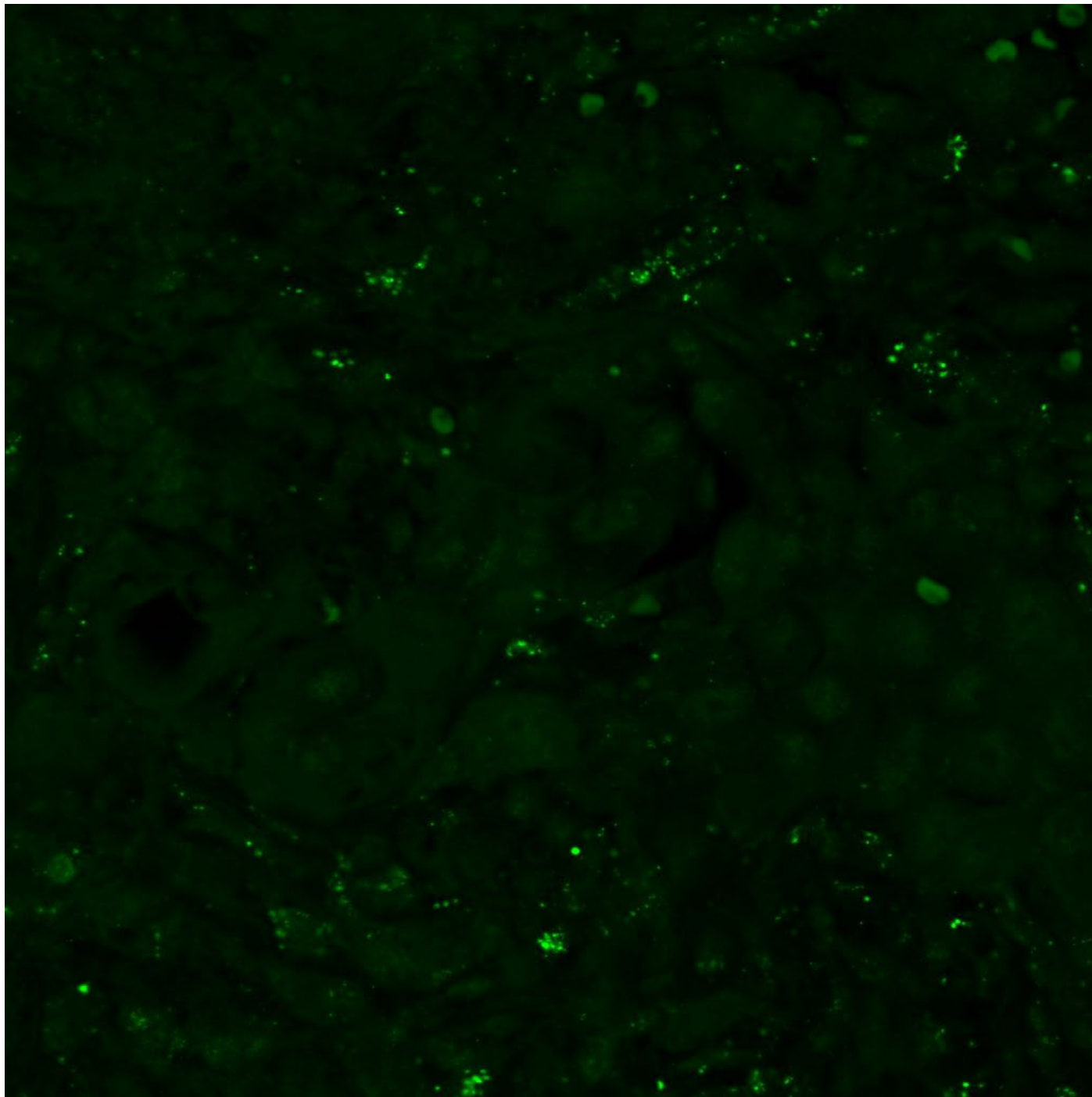

10957\_00

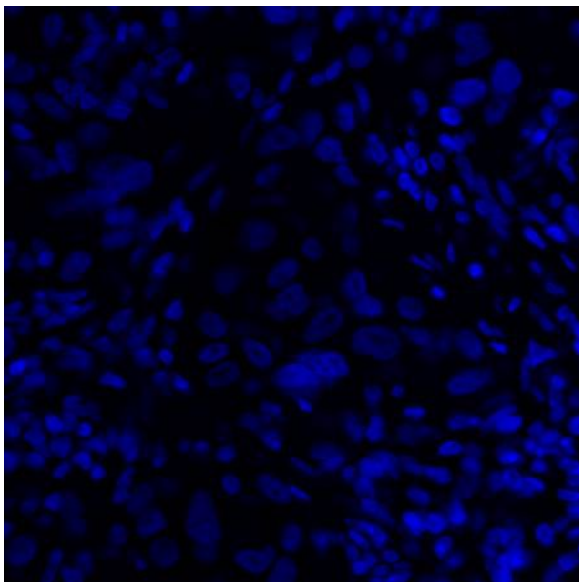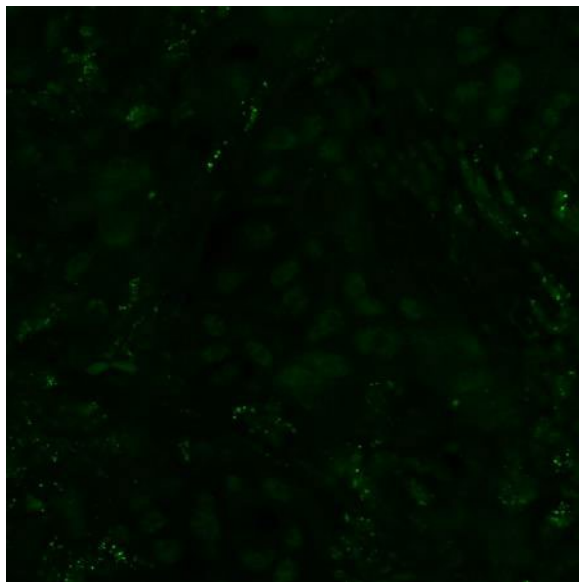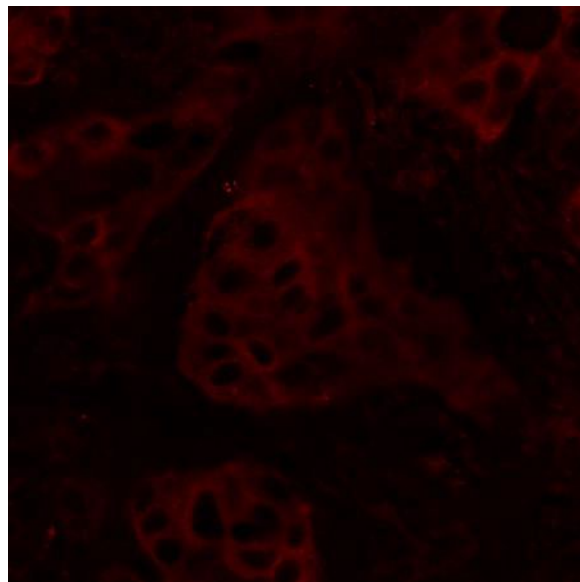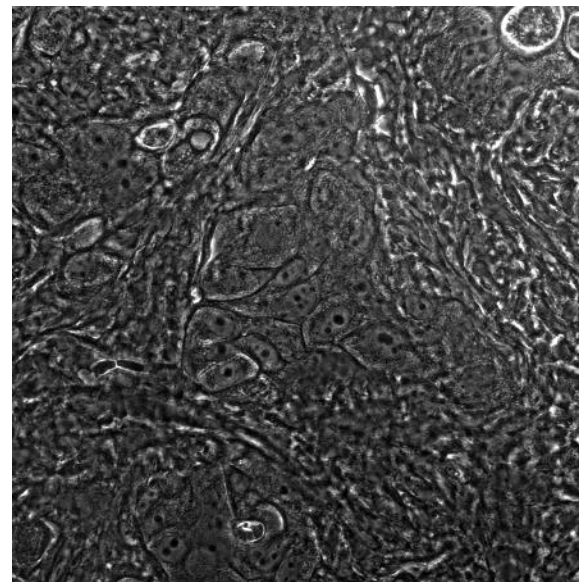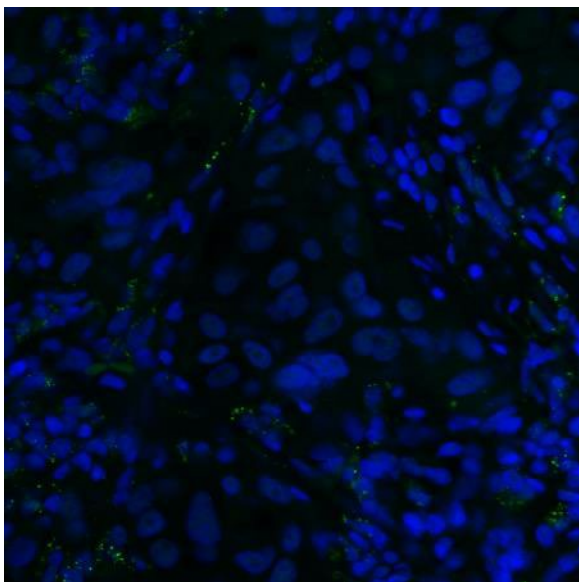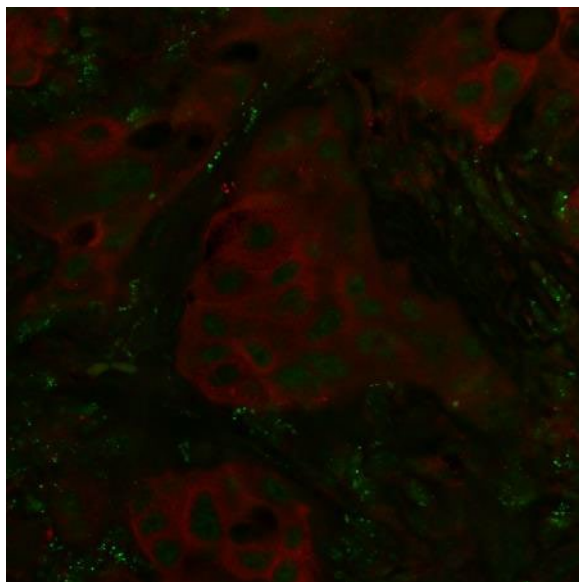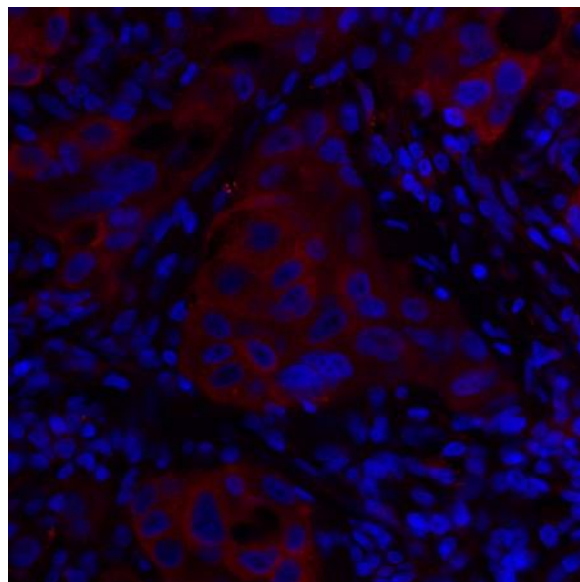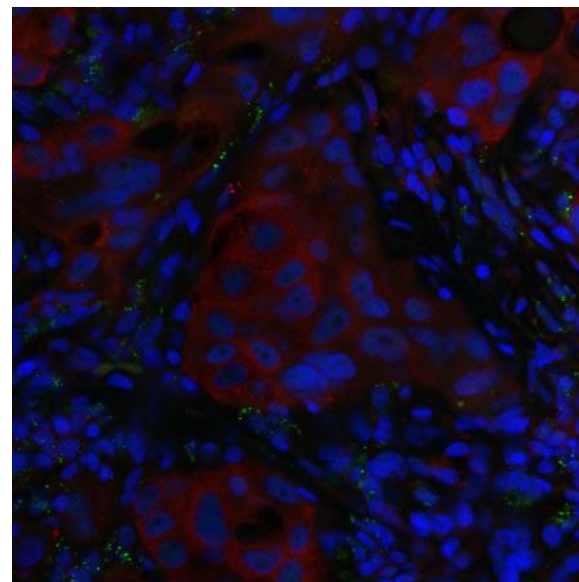

10957\_01

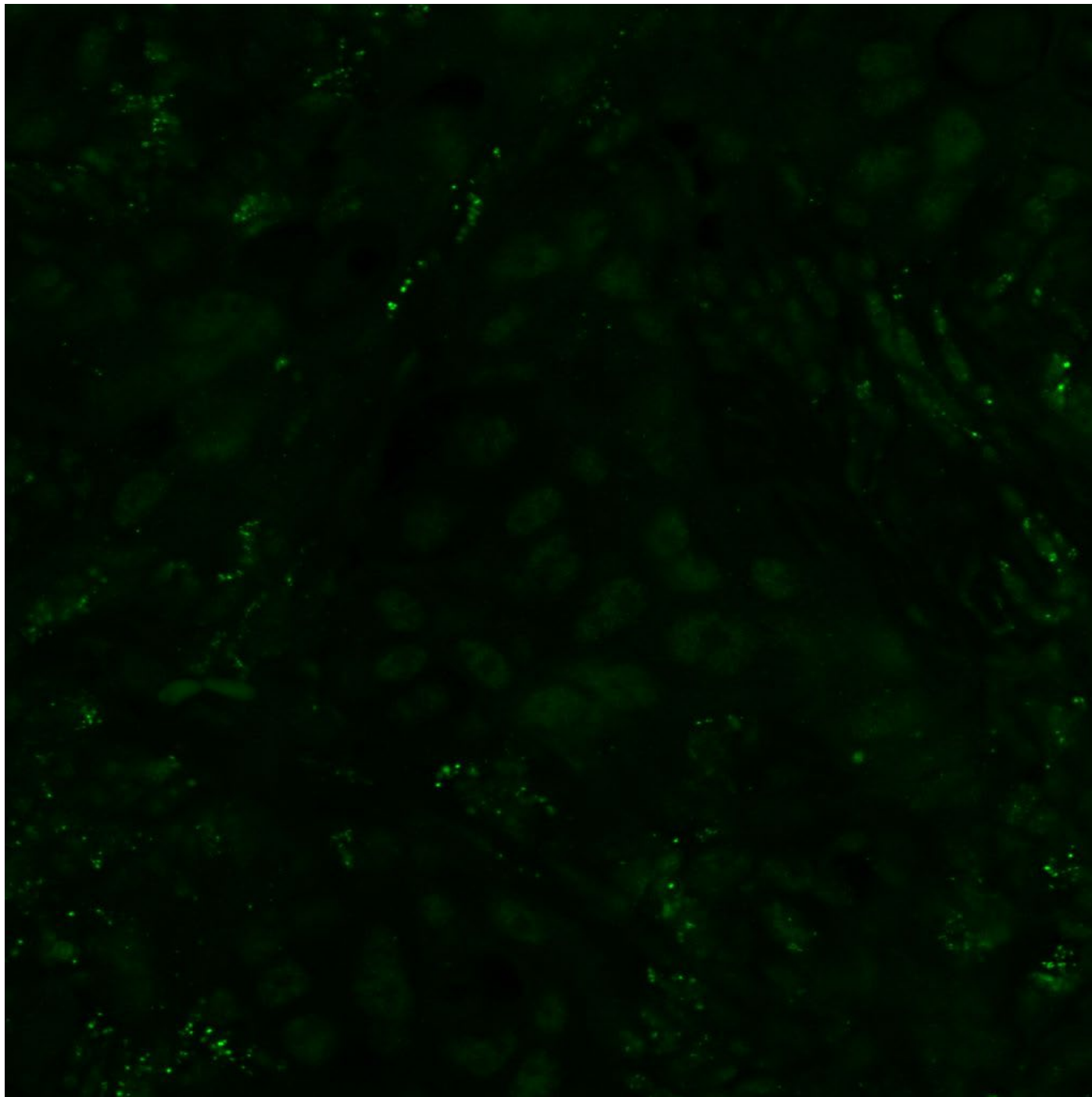

10957\_01

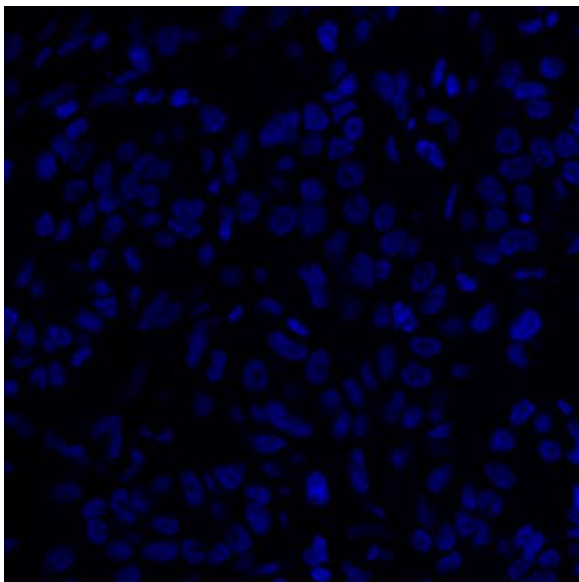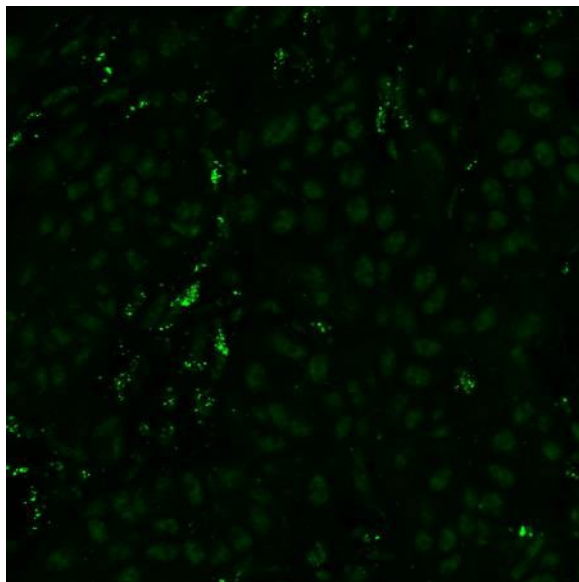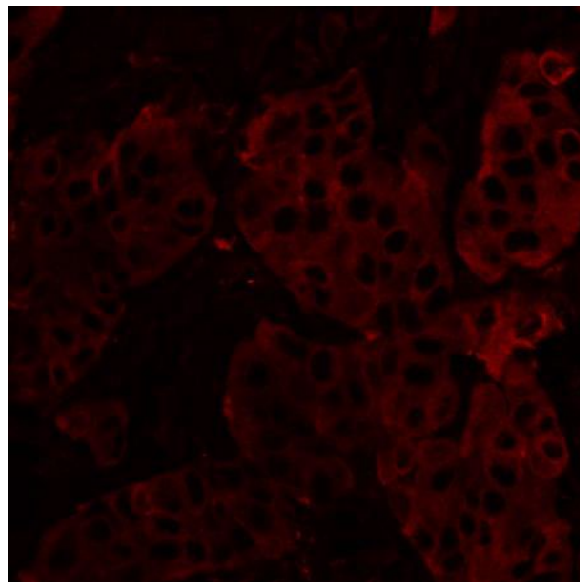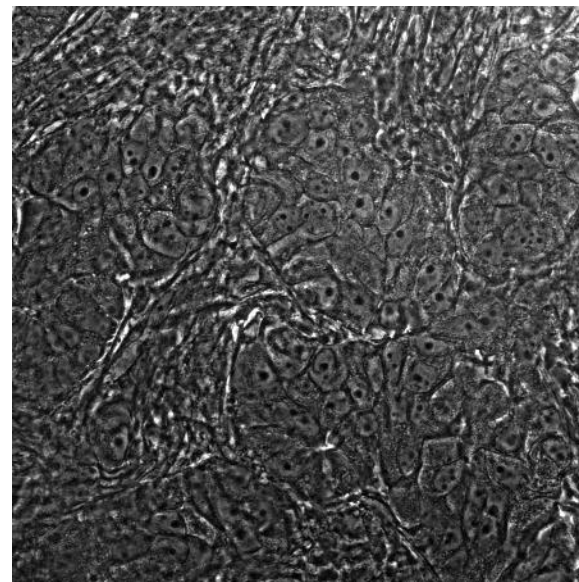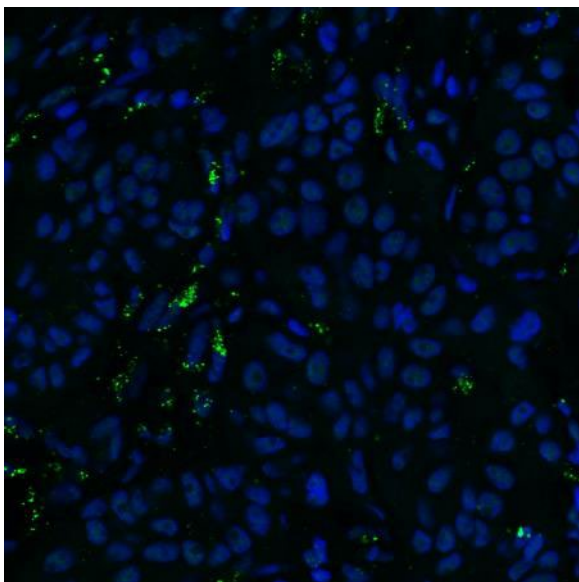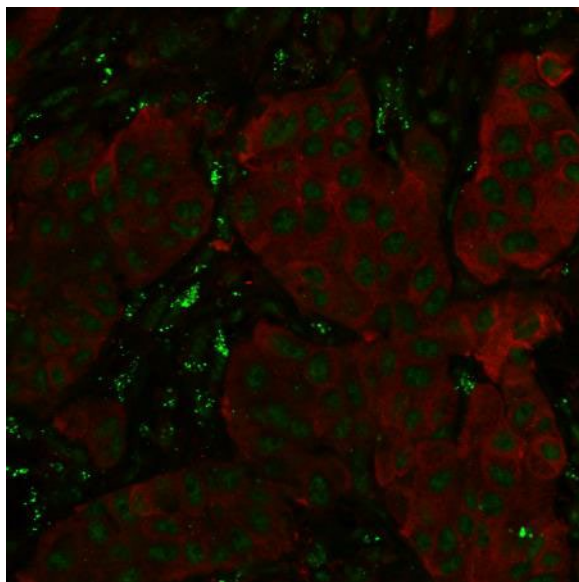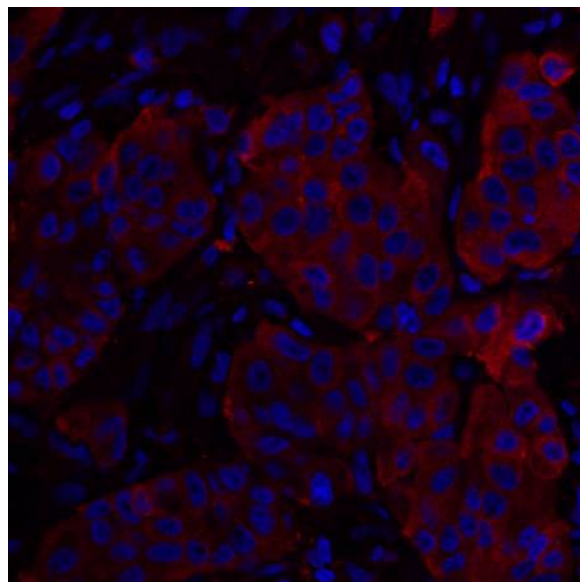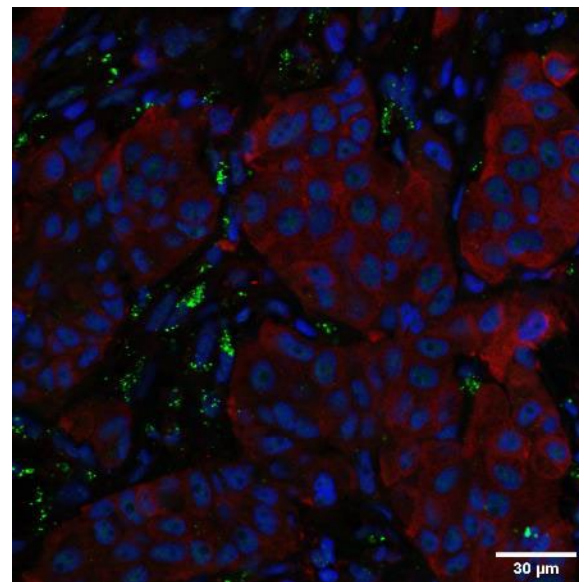

10957\_02

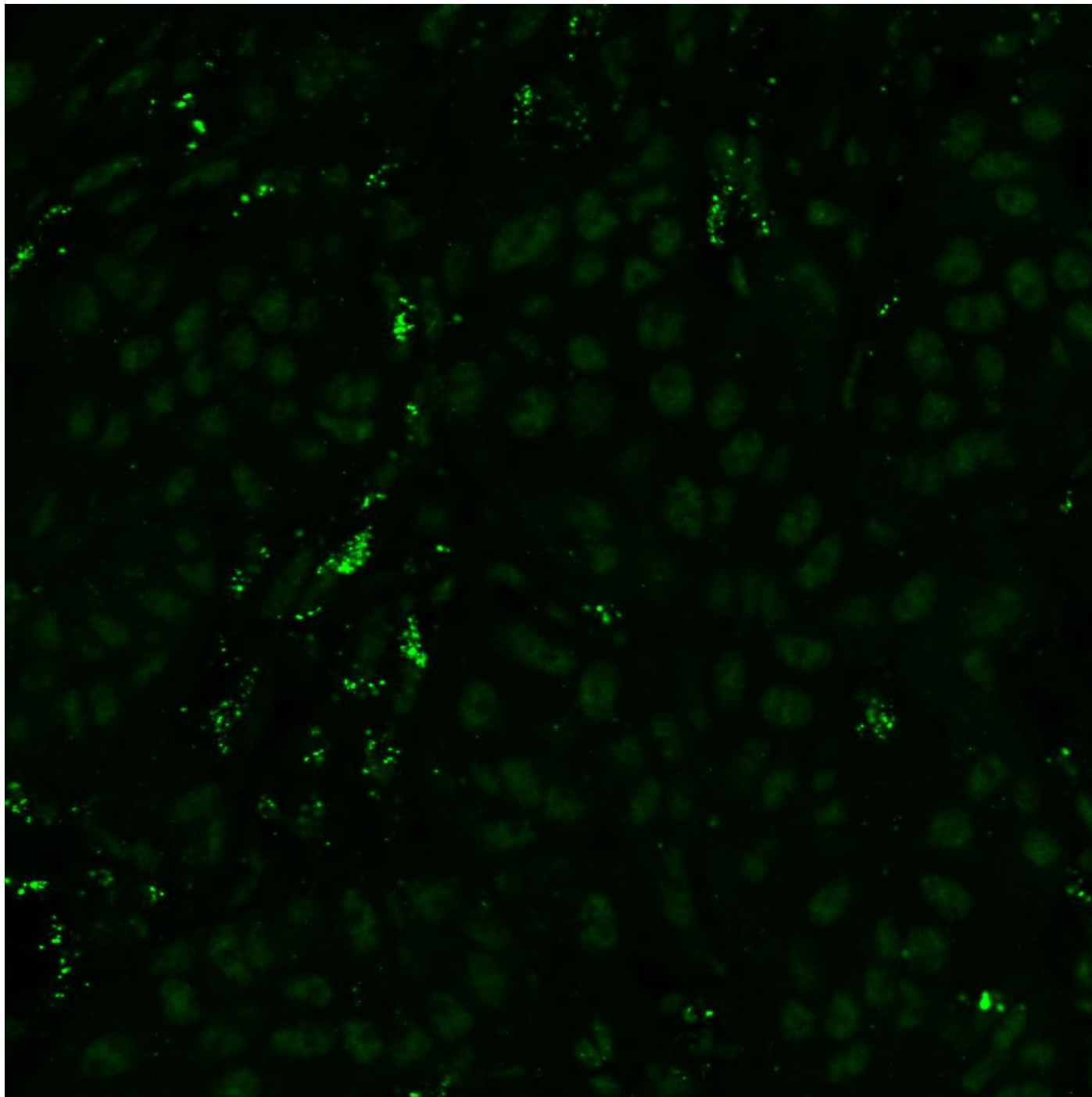

10957\_02

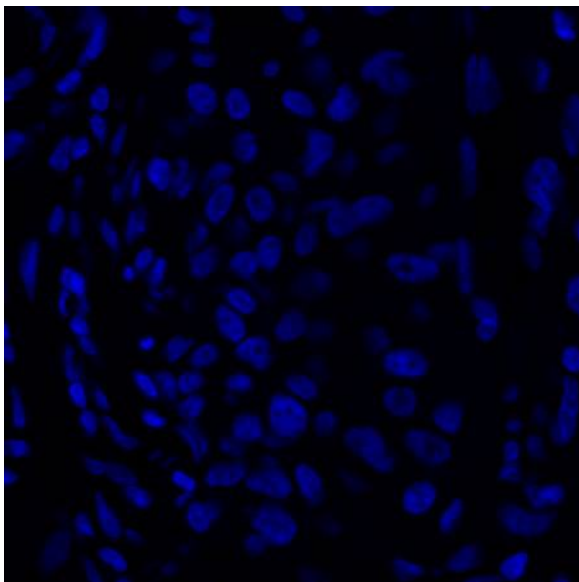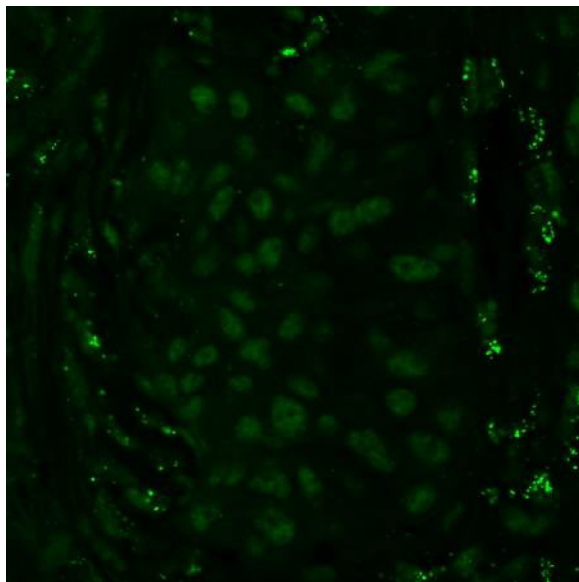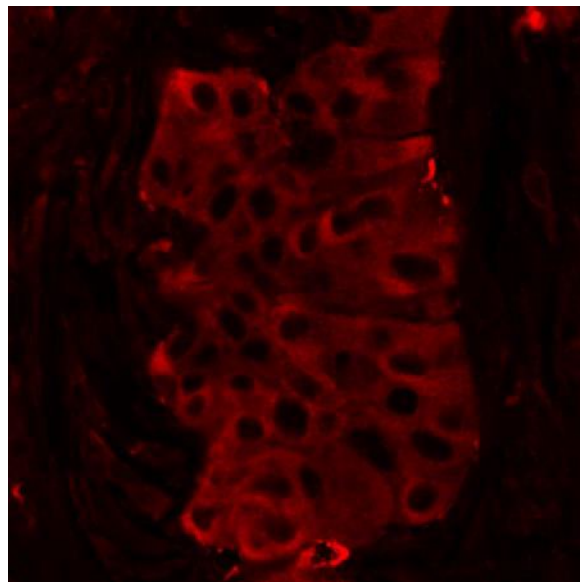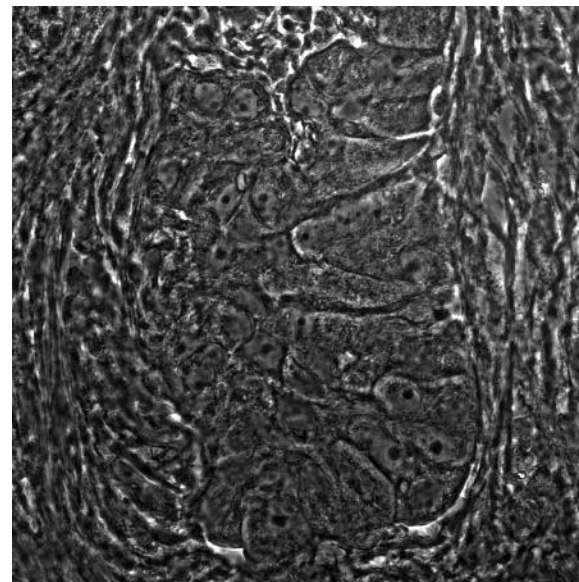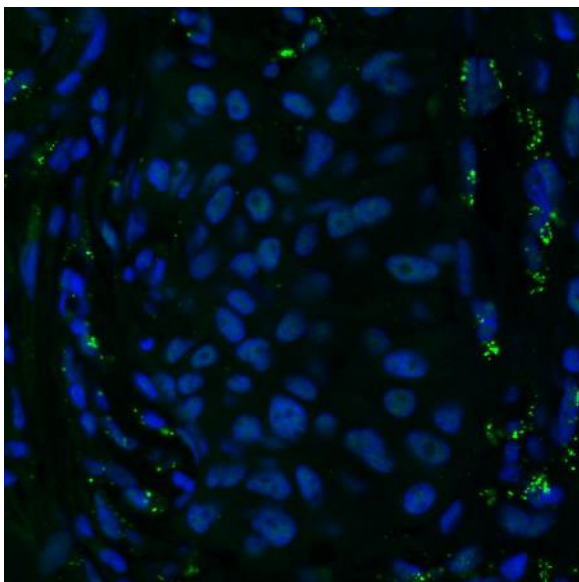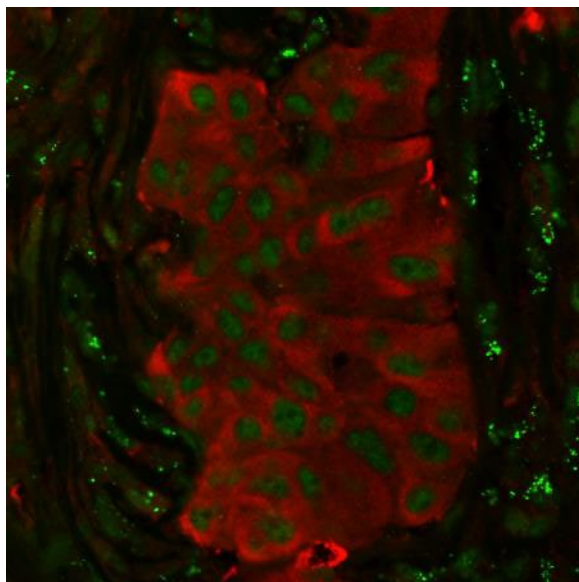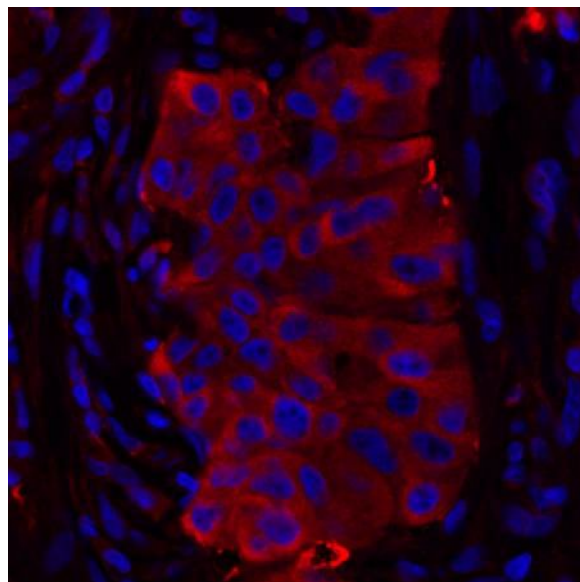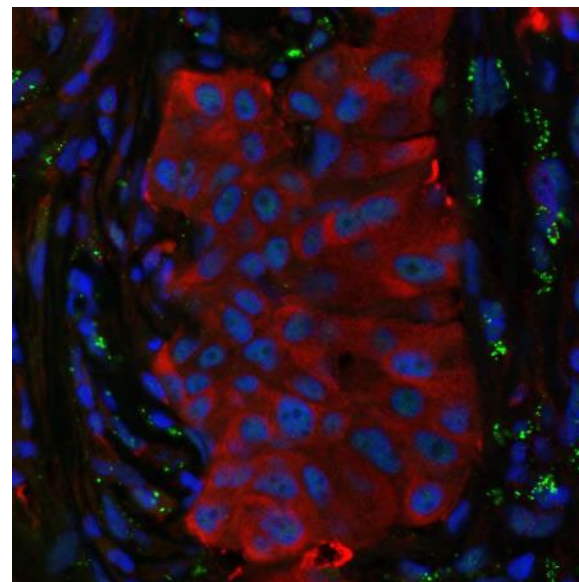

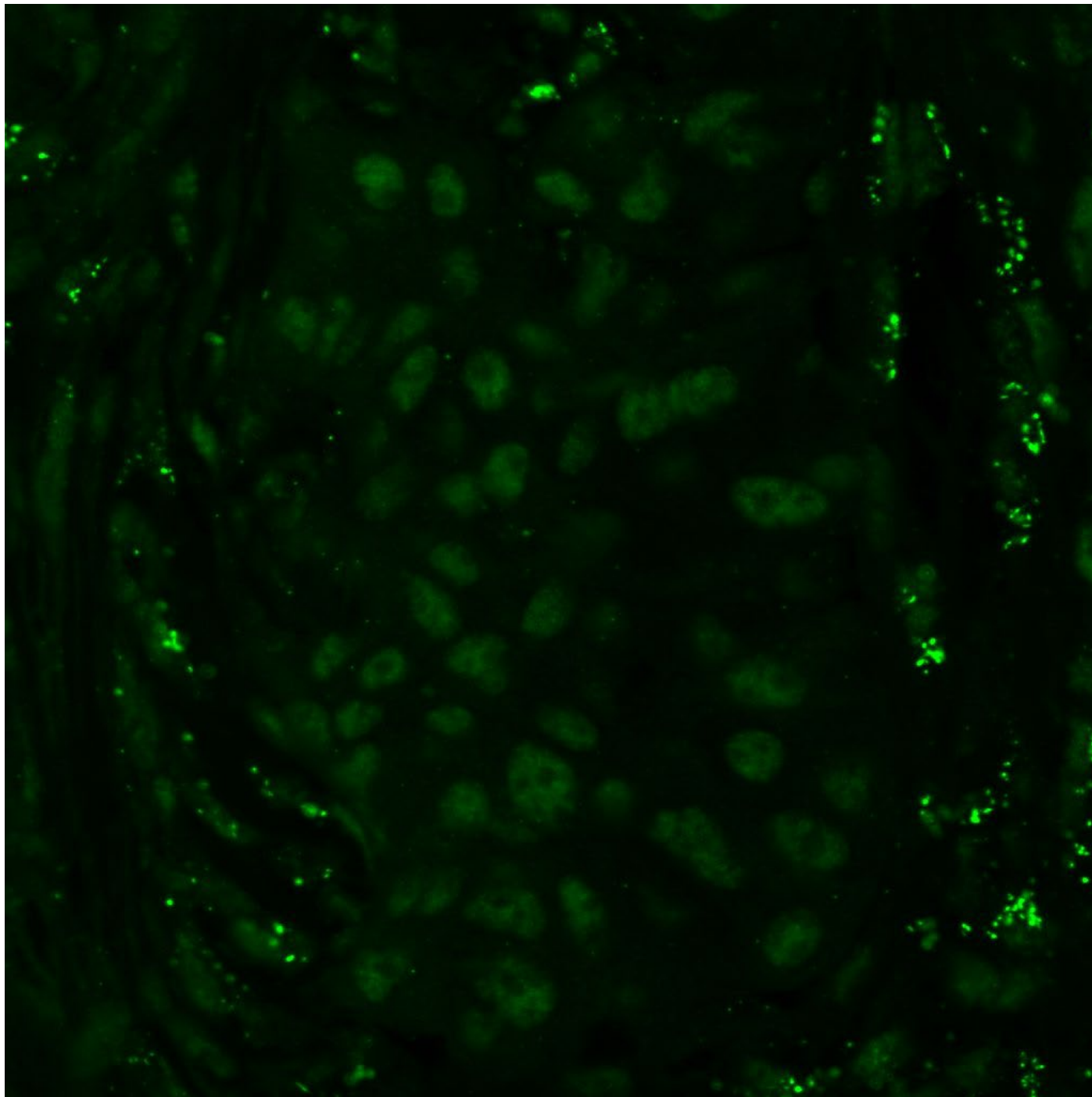

10957\_03

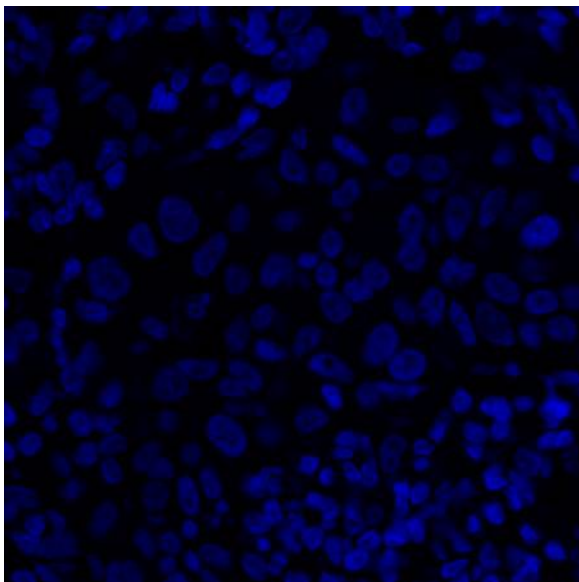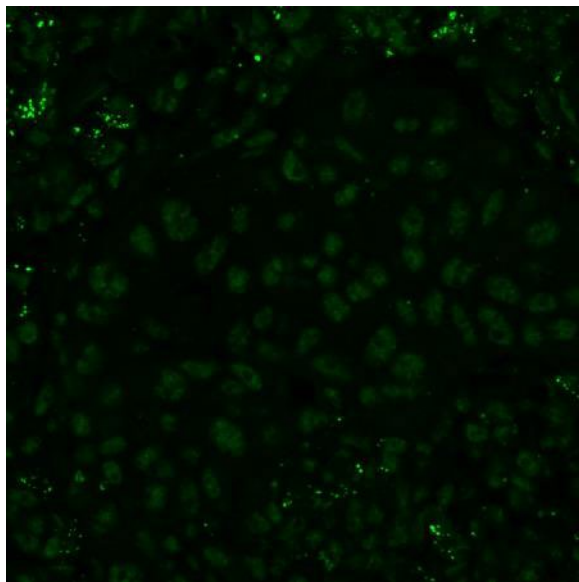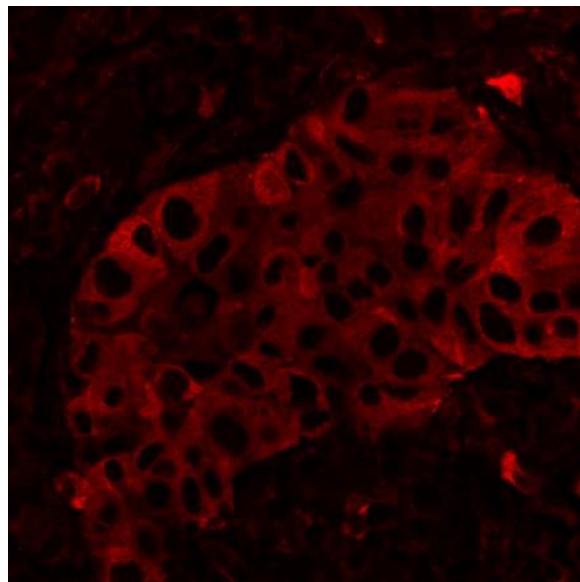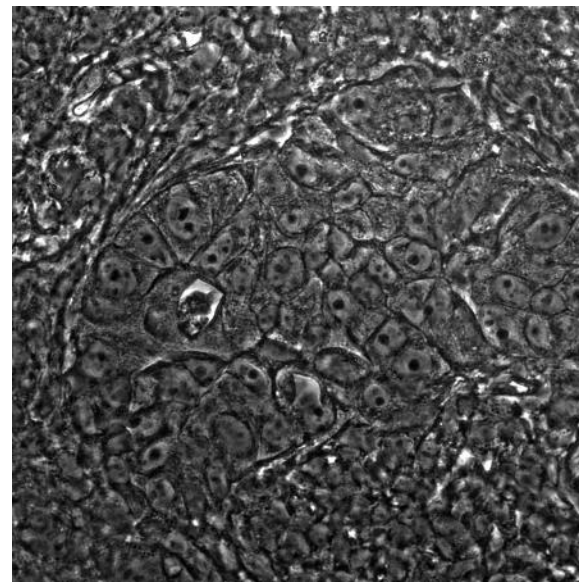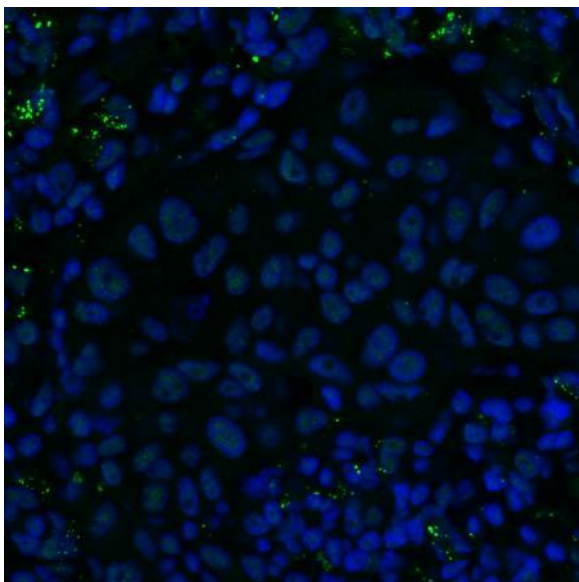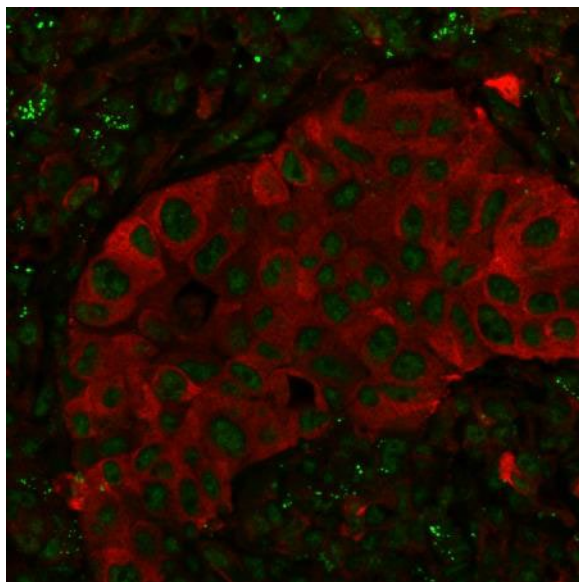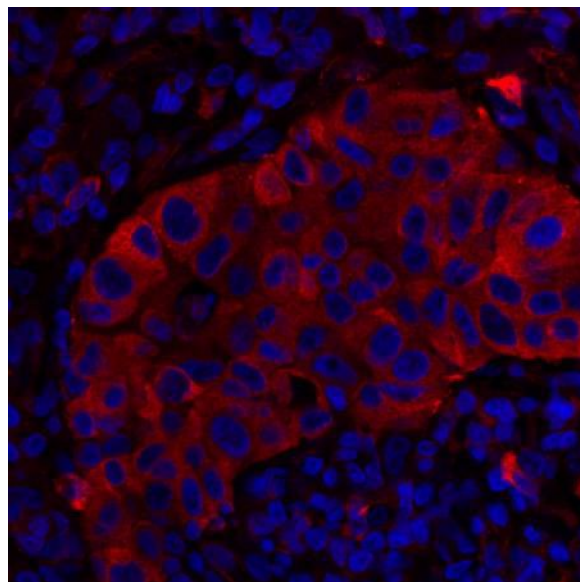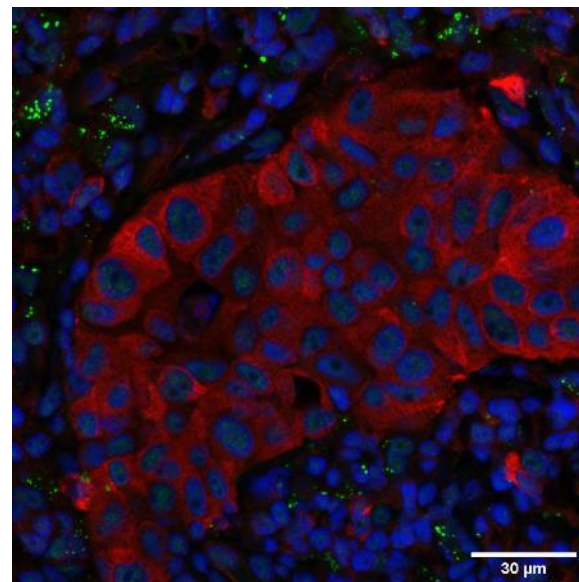

10957\_04

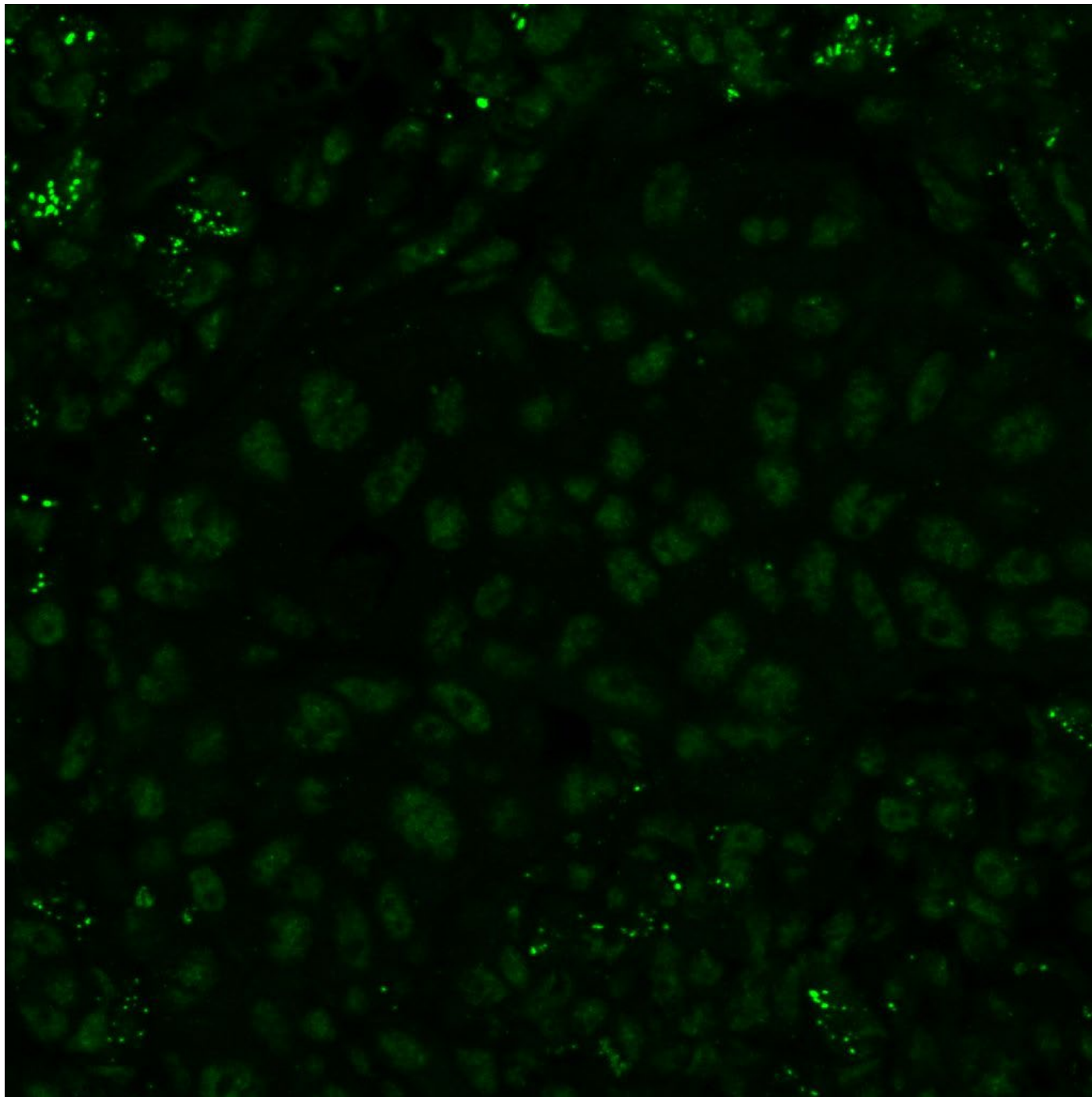

10957\_04

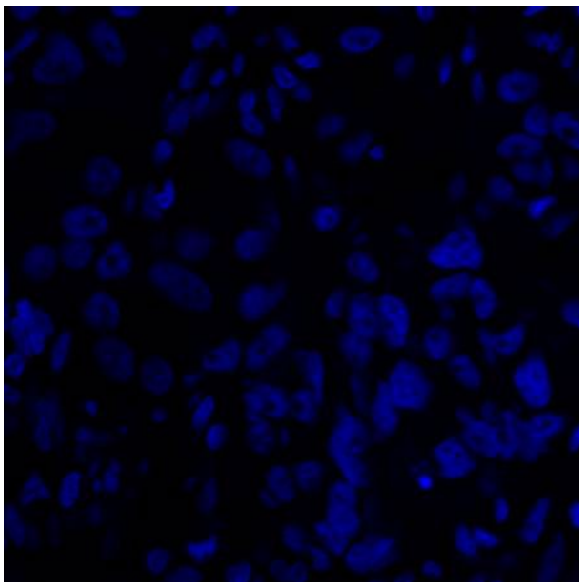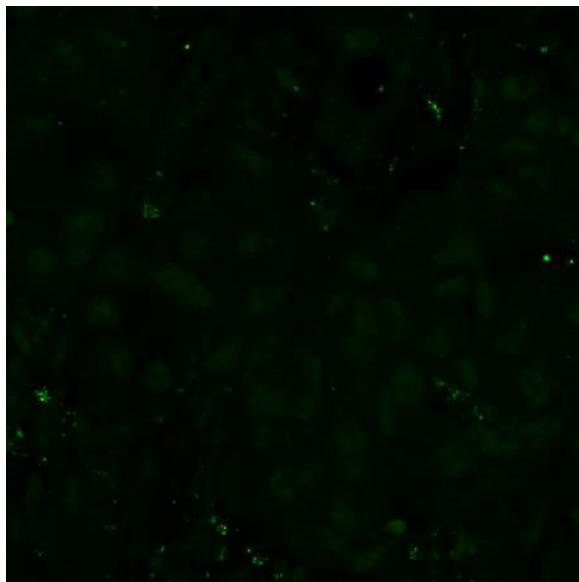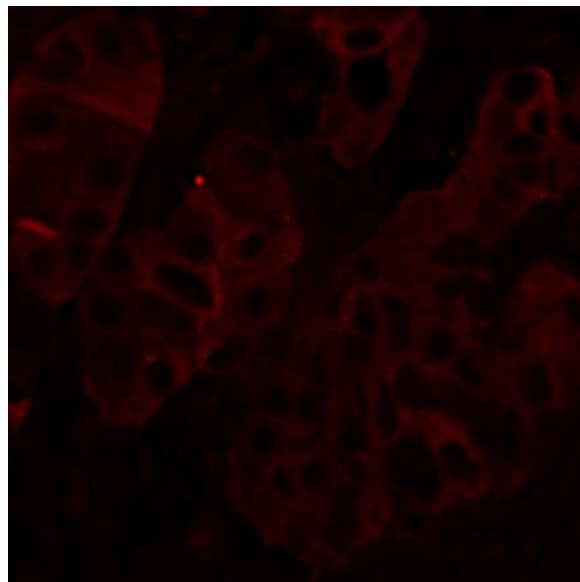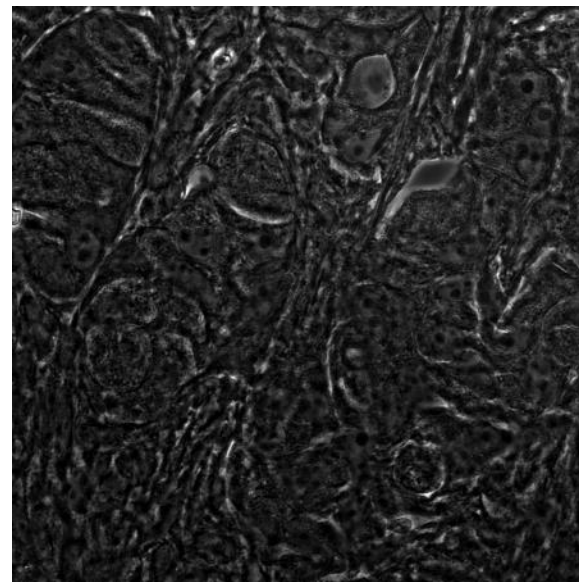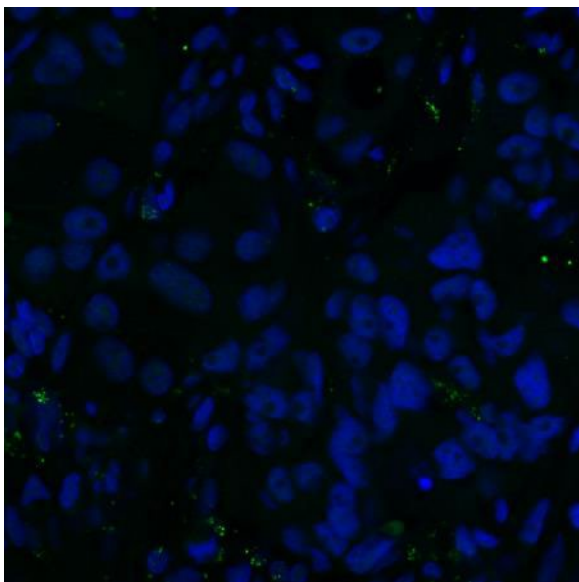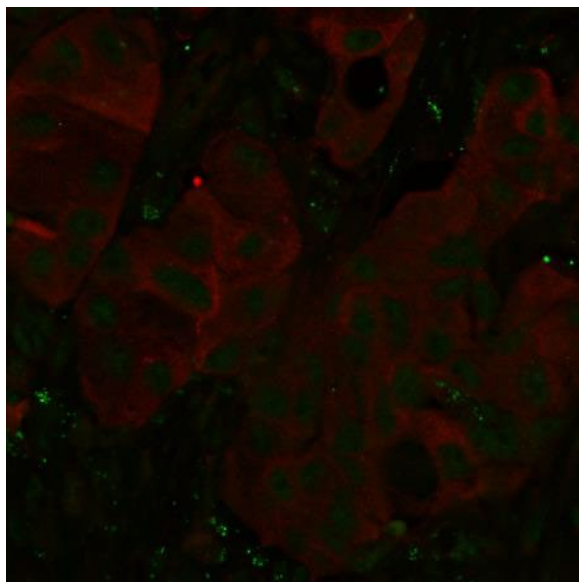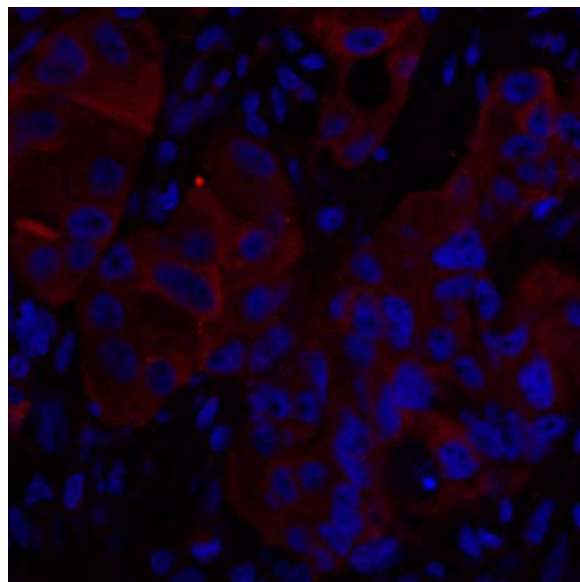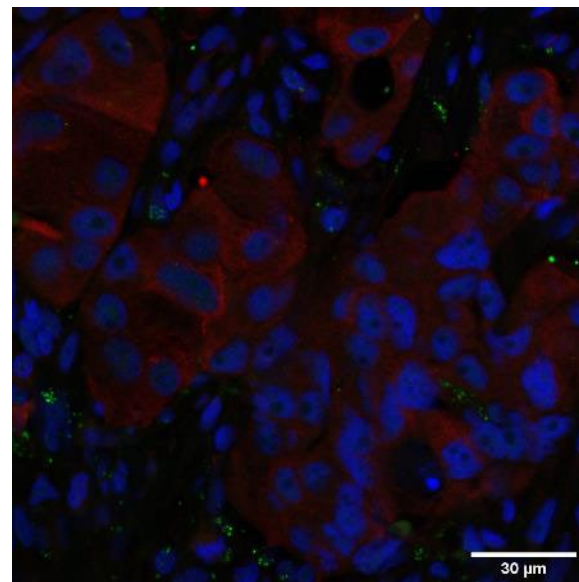

10957\_05

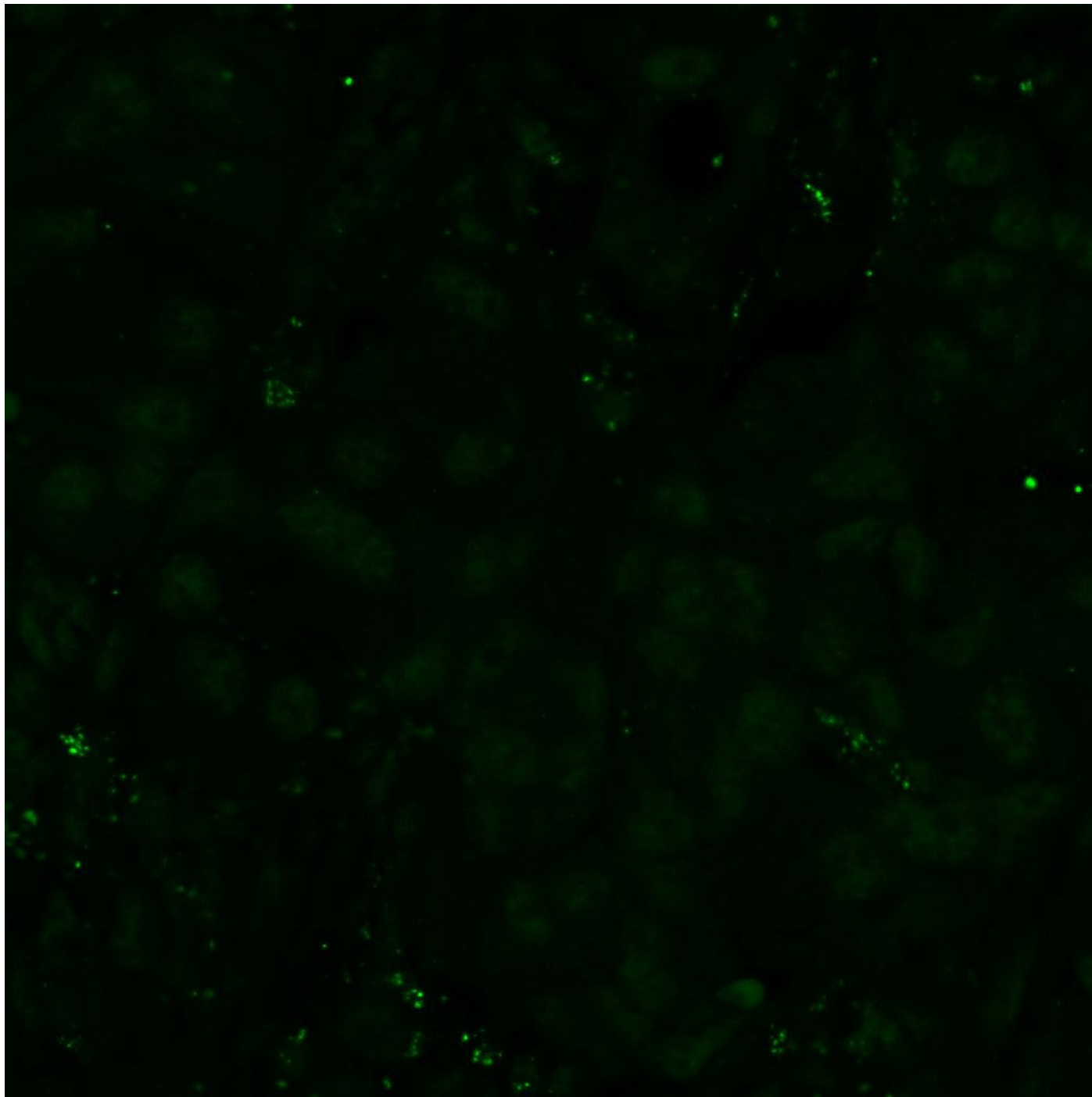

10957\_05

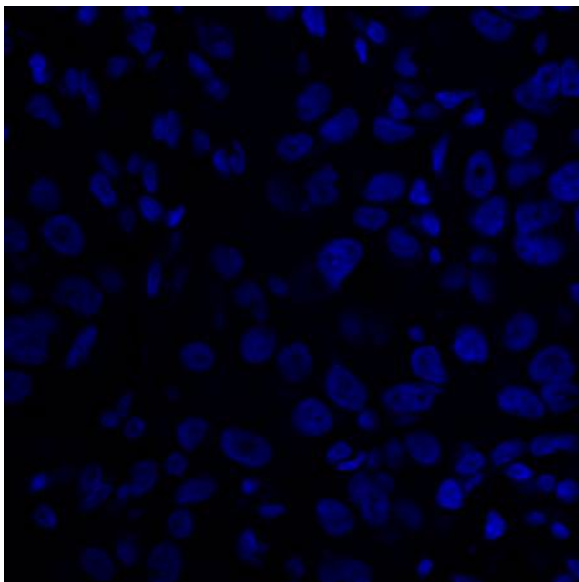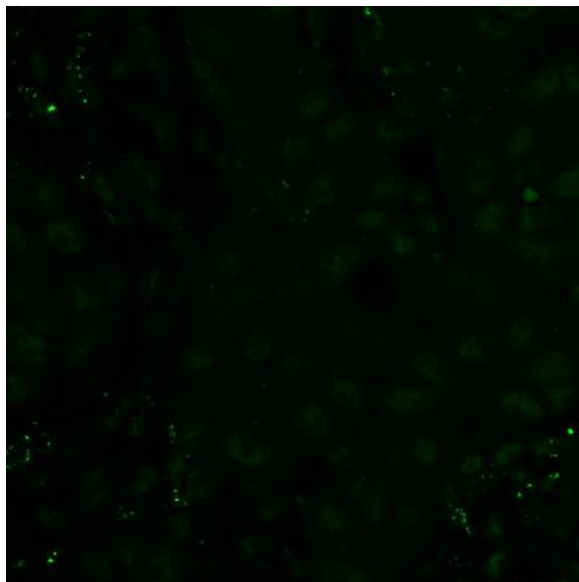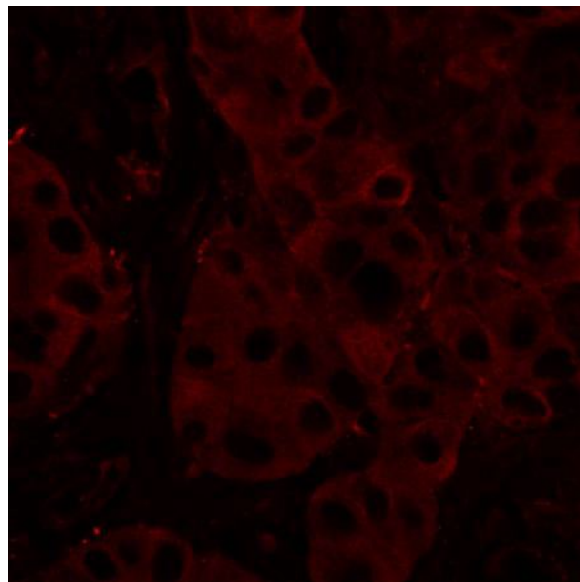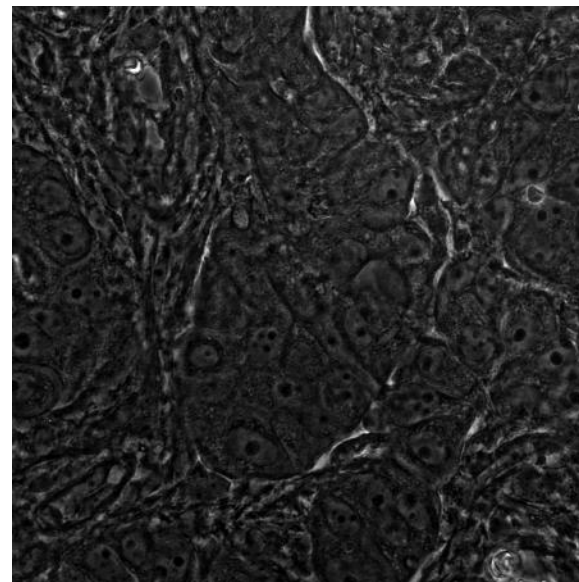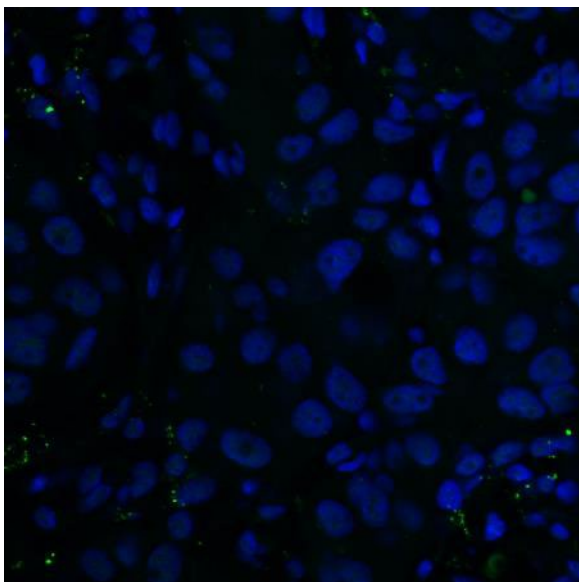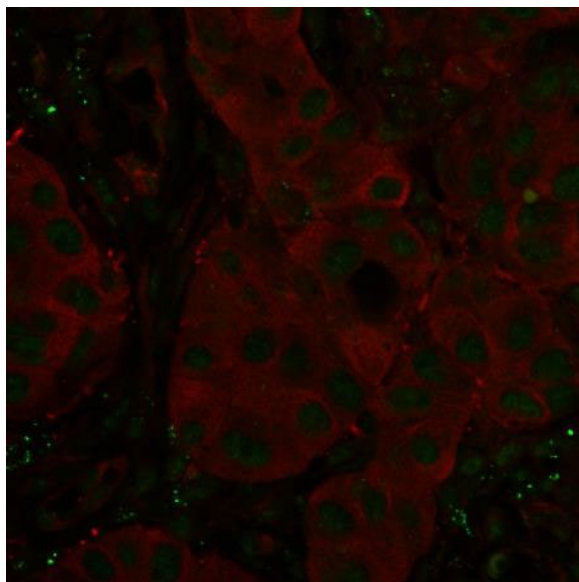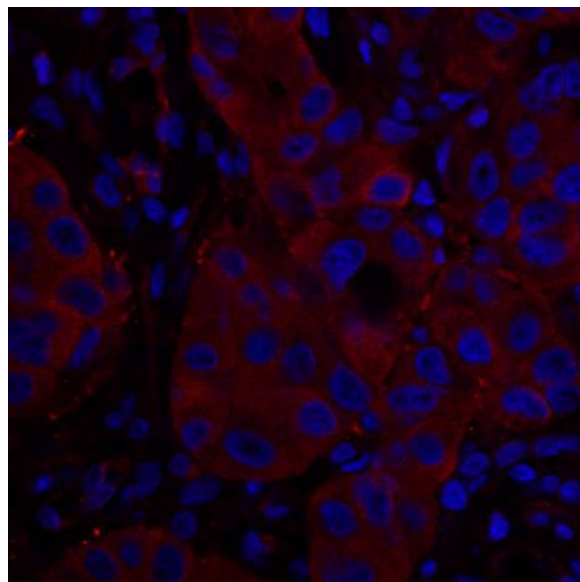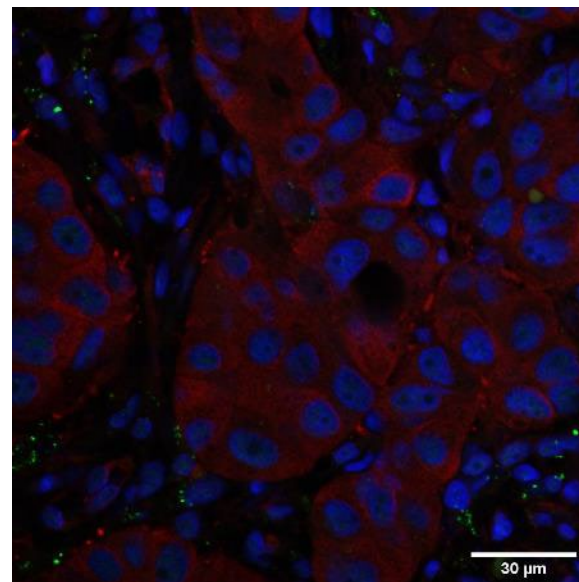

10957\_06

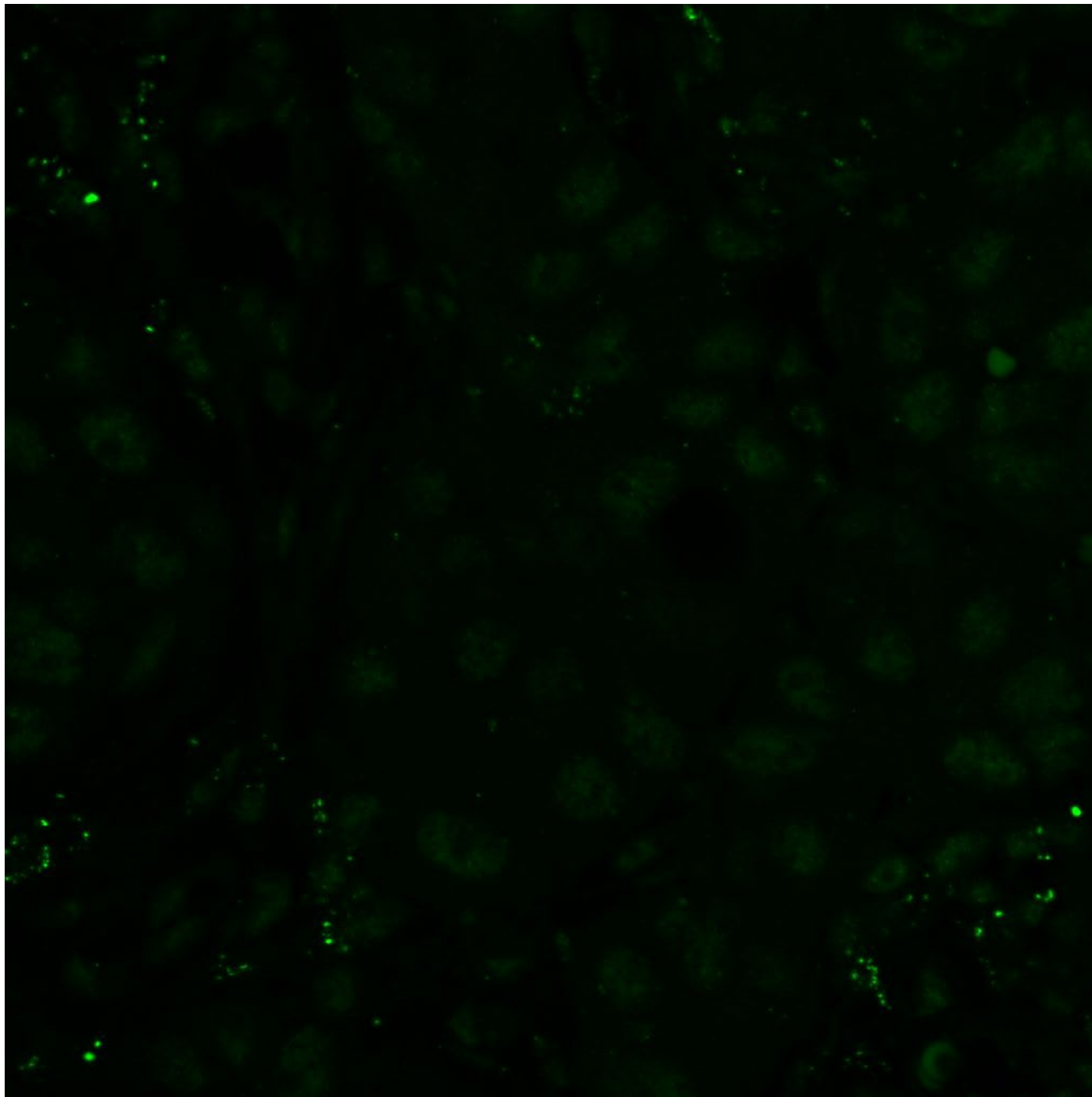

10957\_06

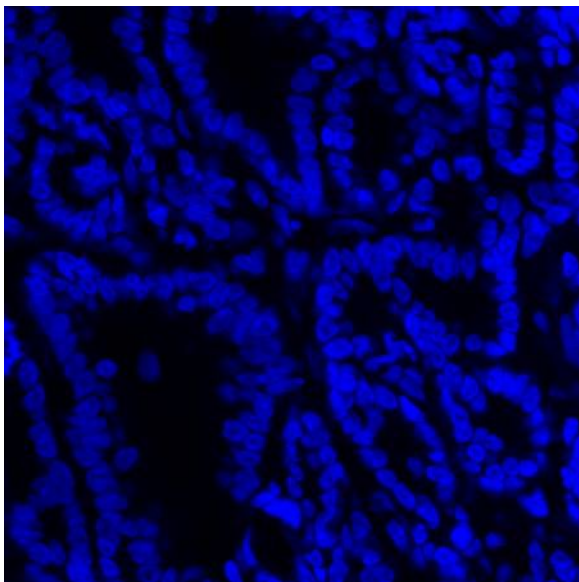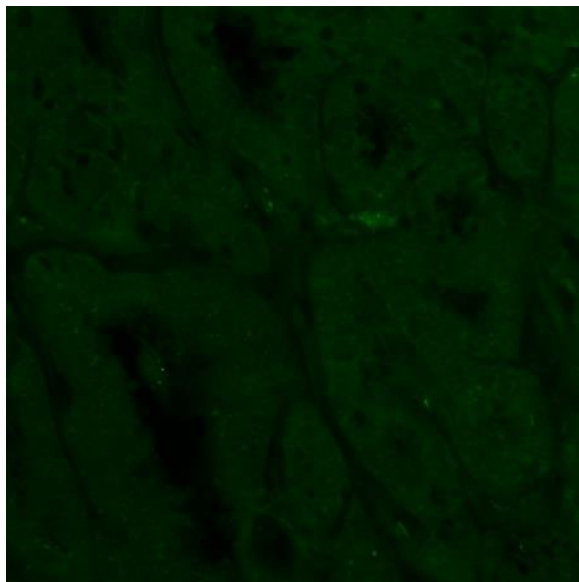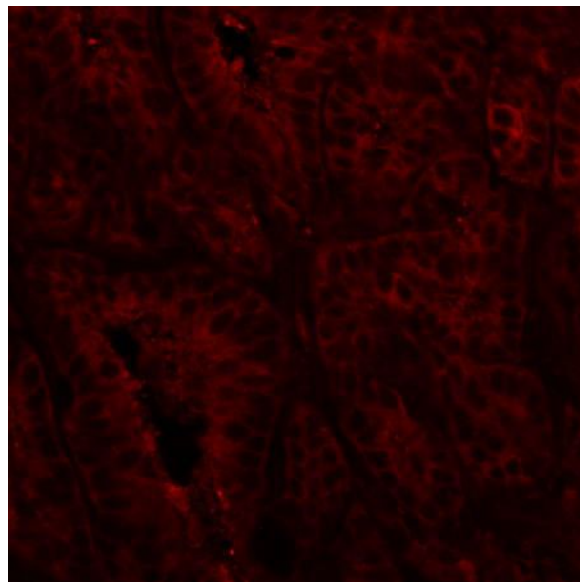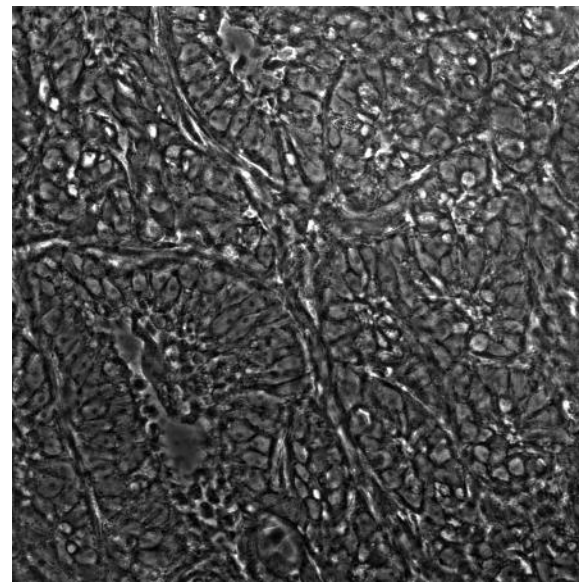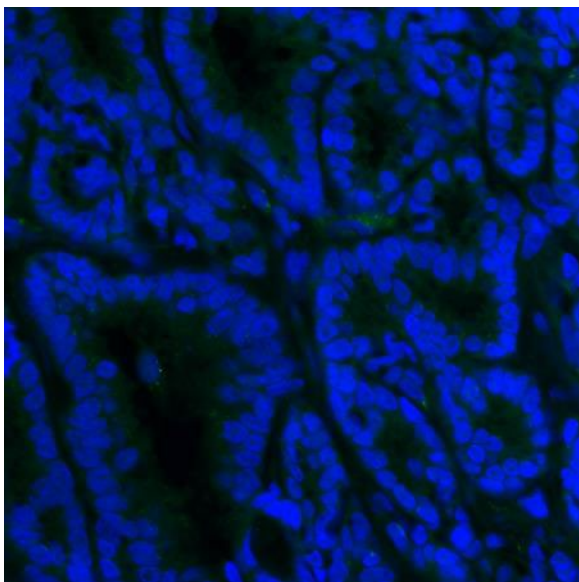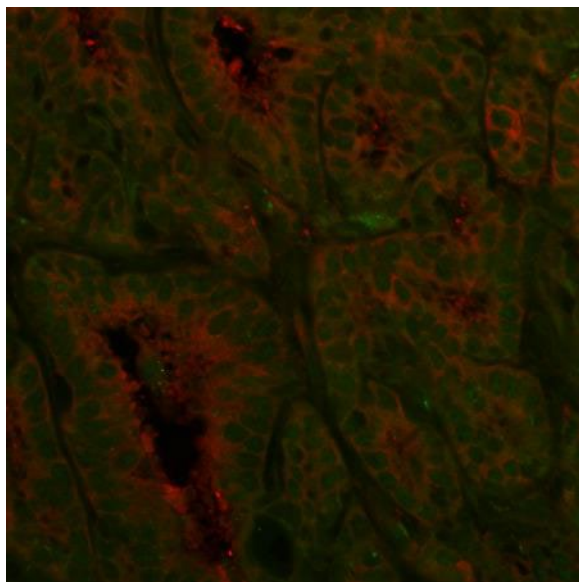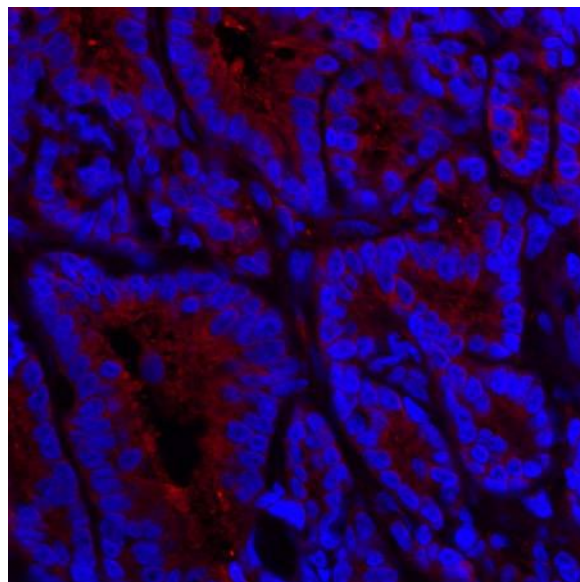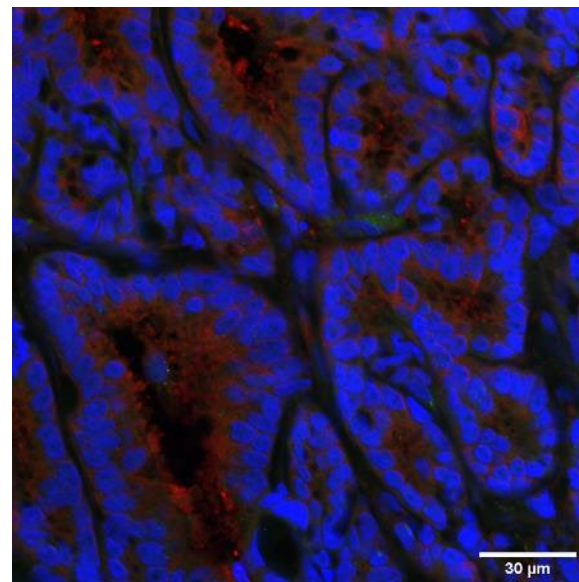

11086\_00

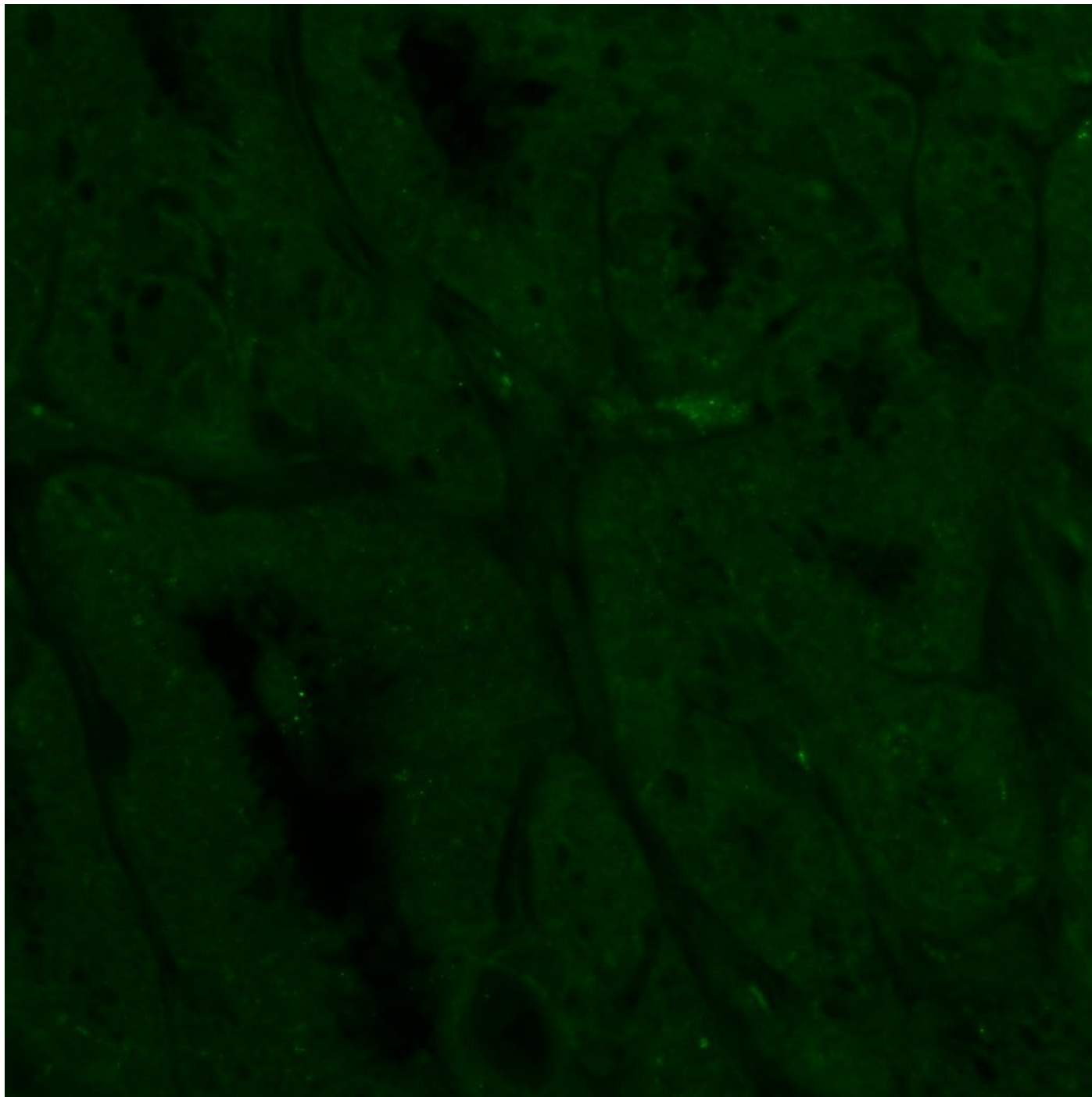

11086\_0

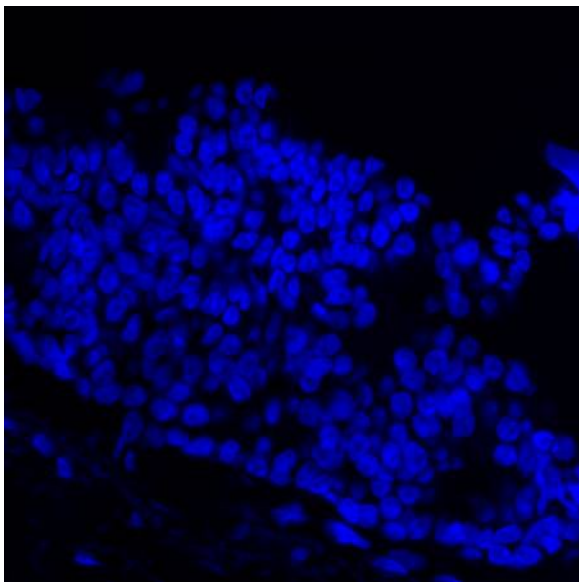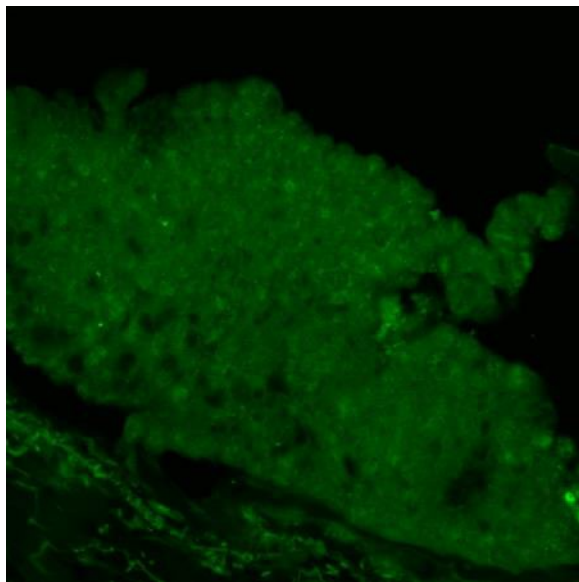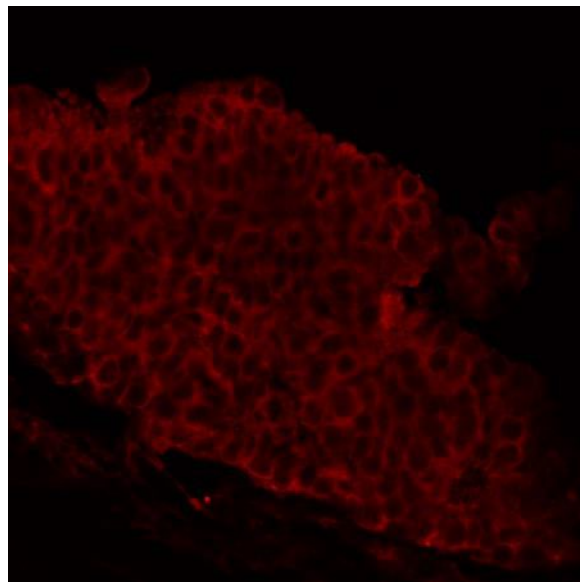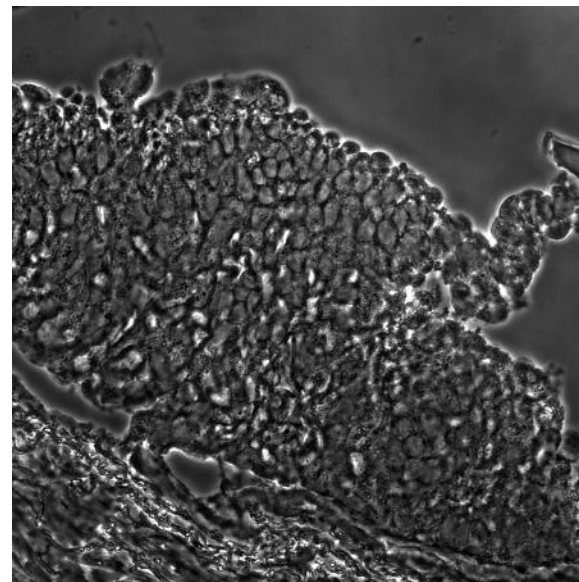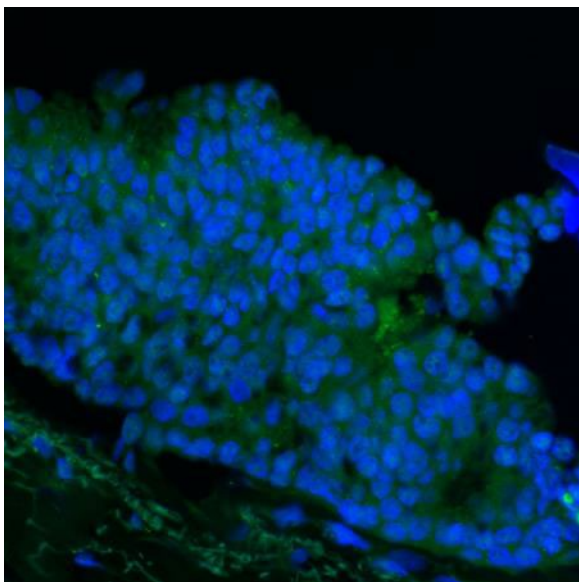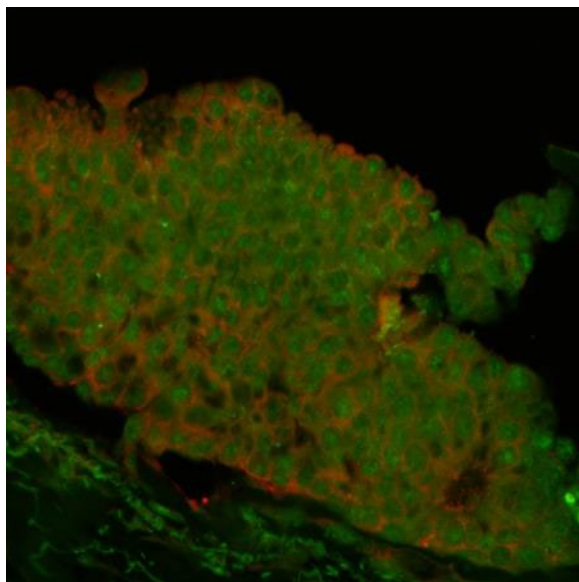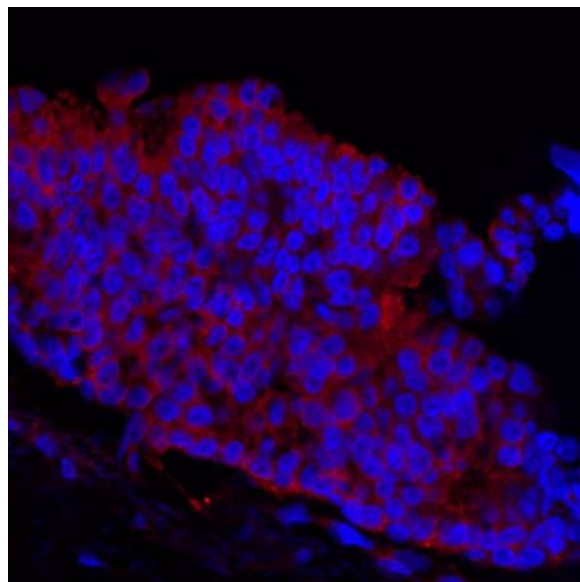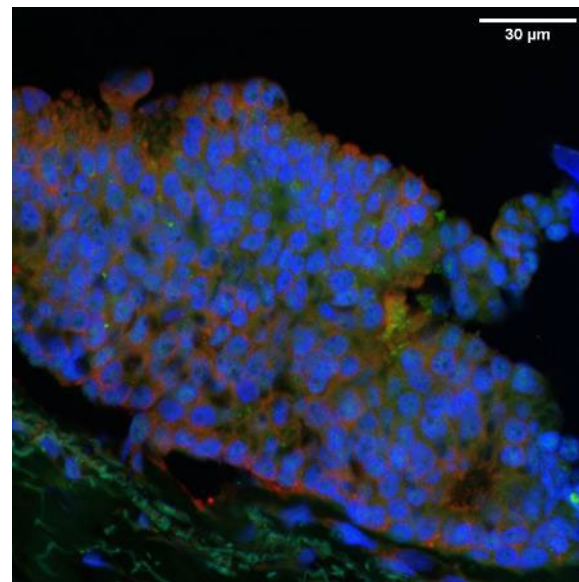

11086\_01

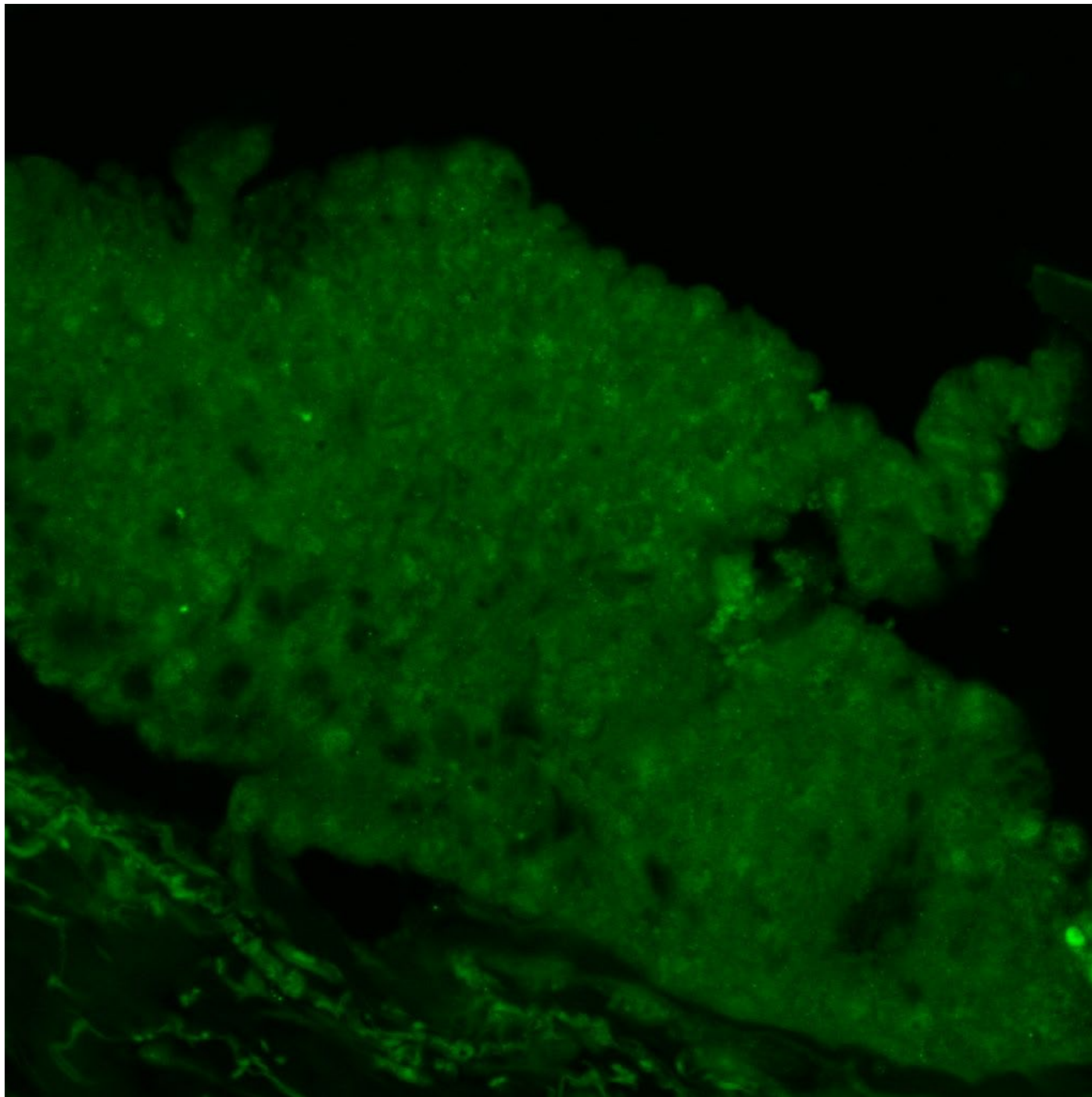

11086\_01

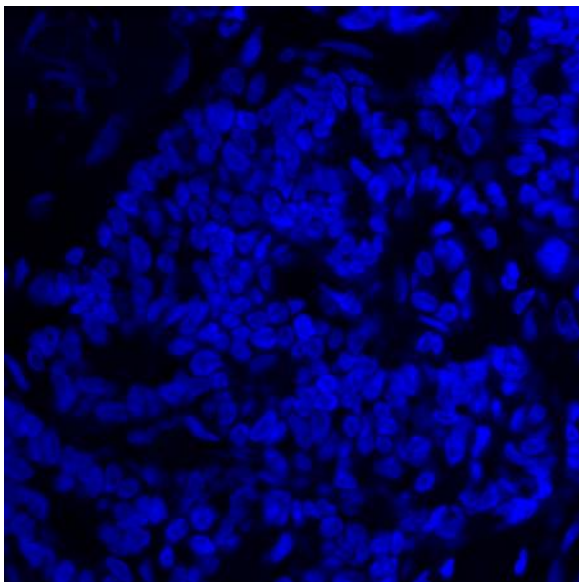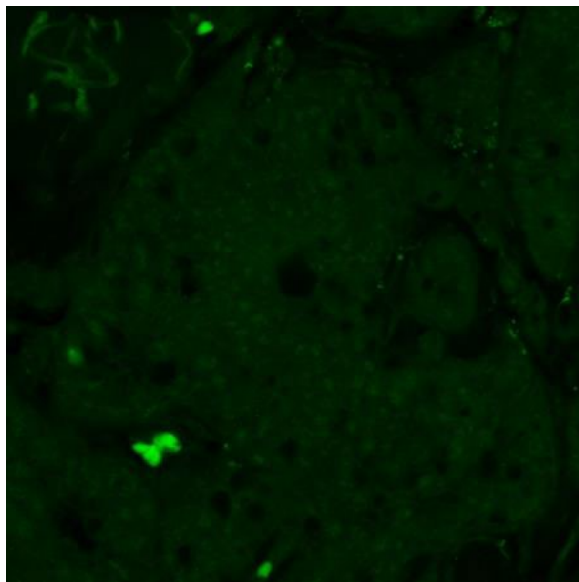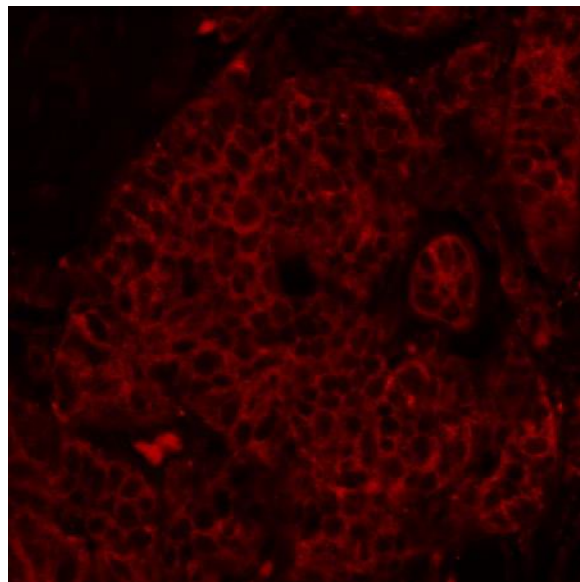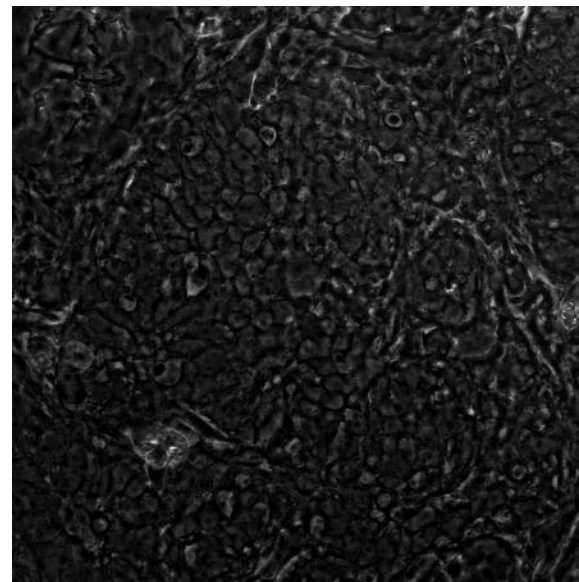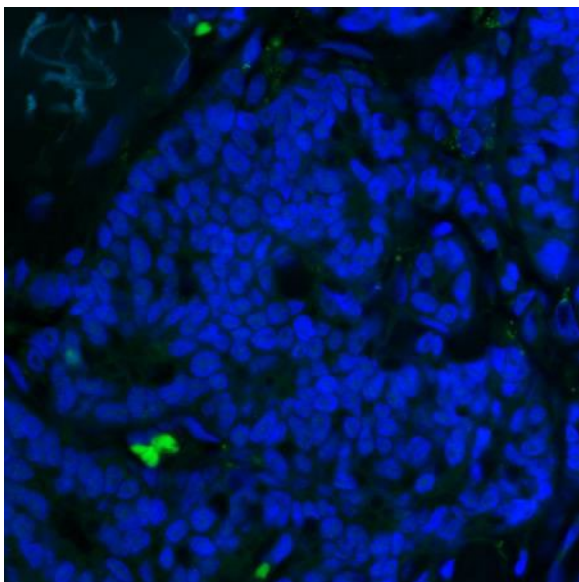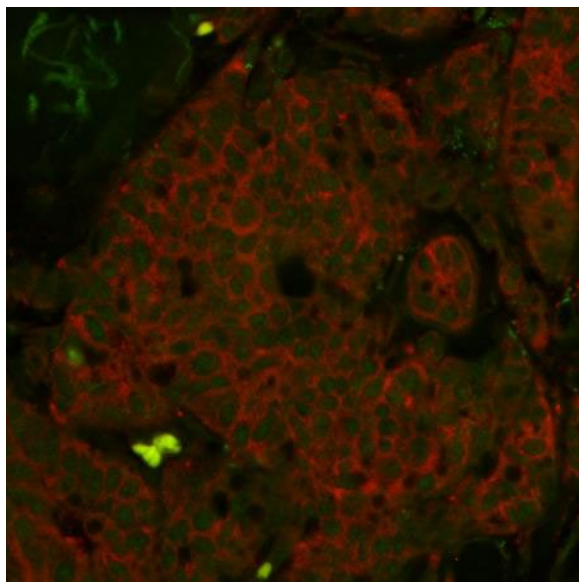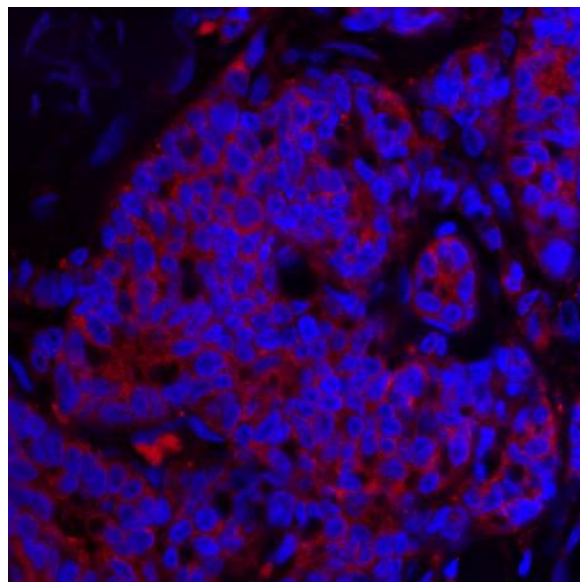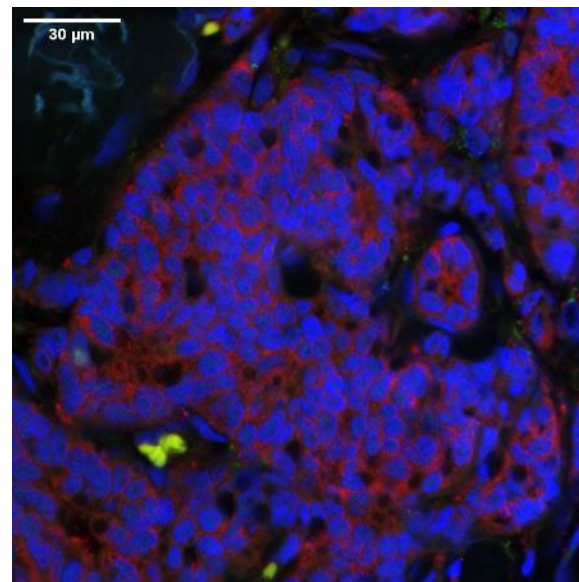

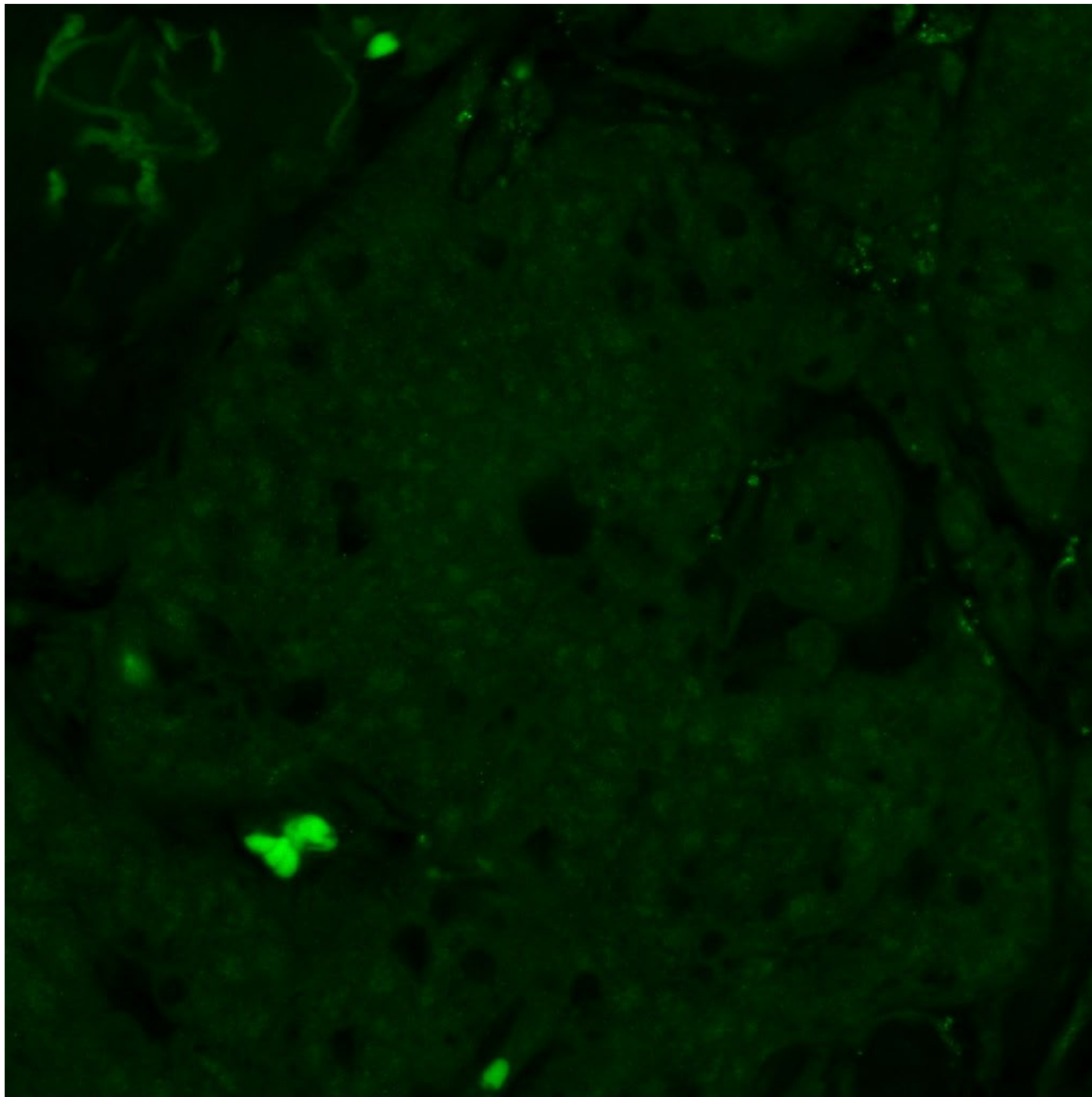

11086\_02

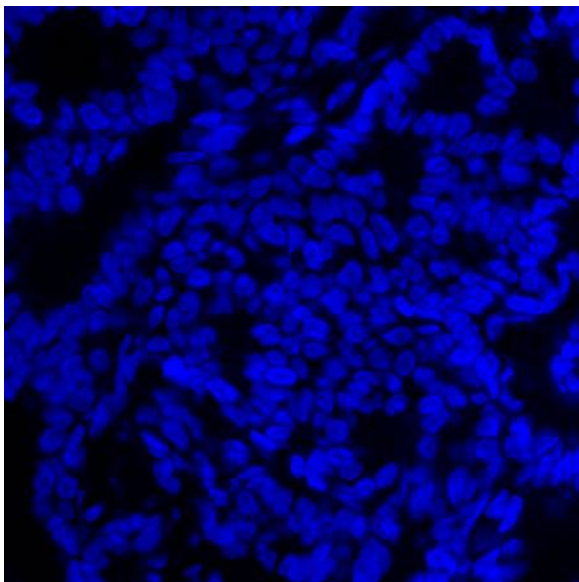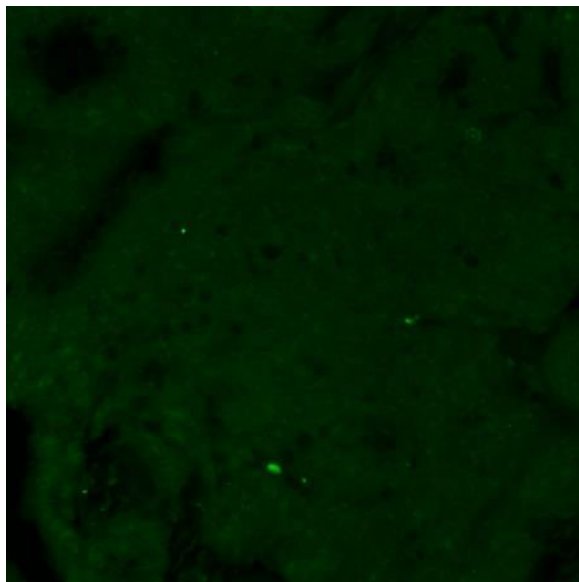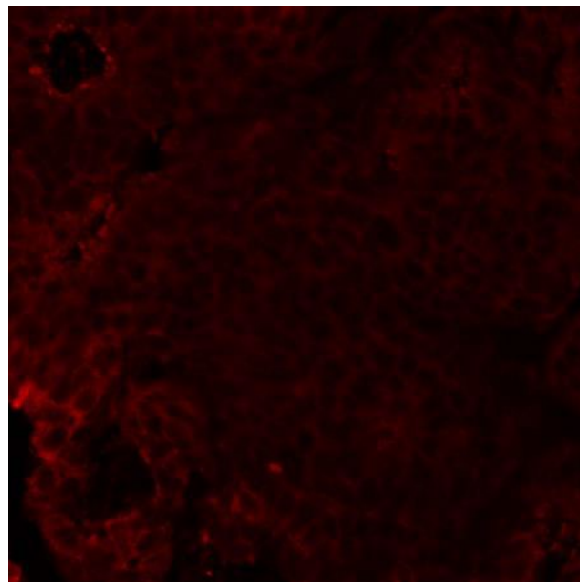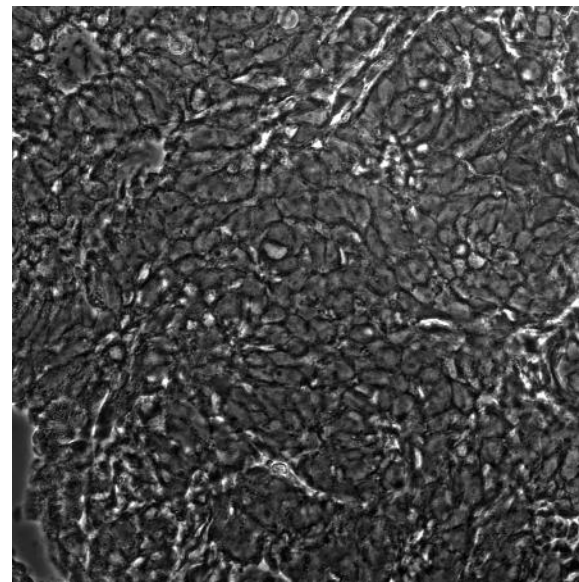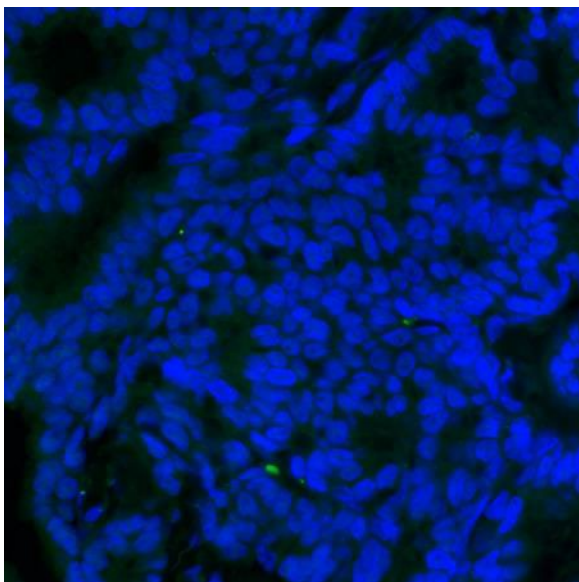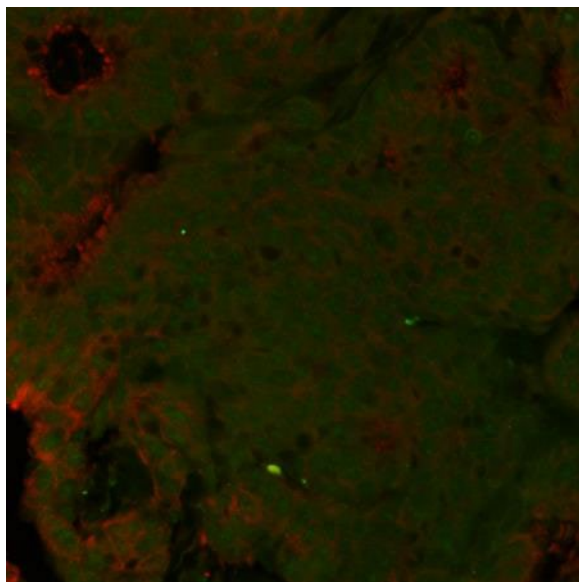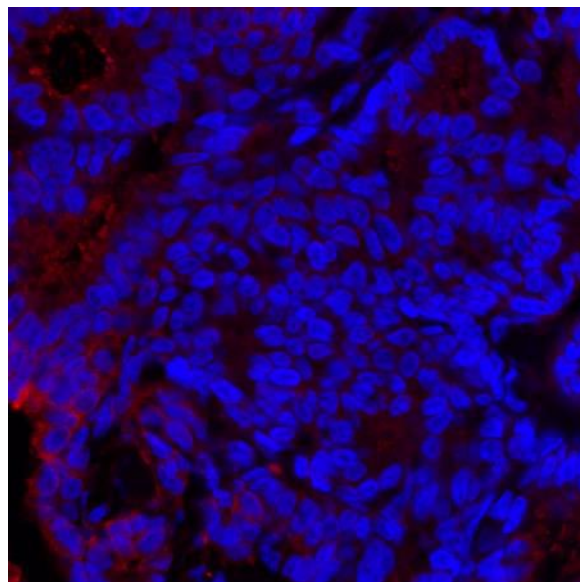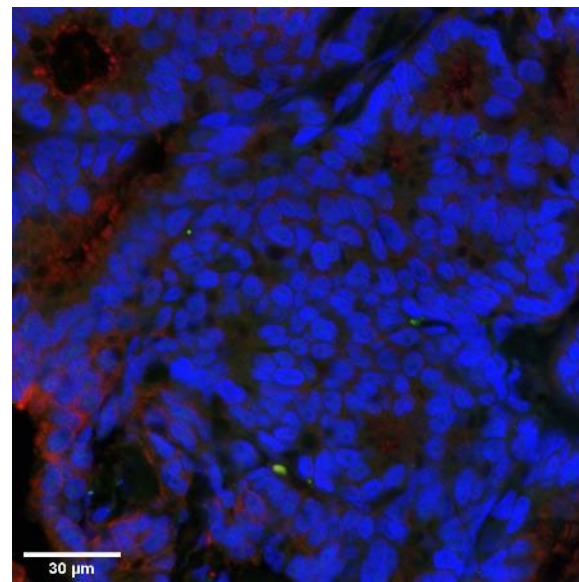

11086\_03

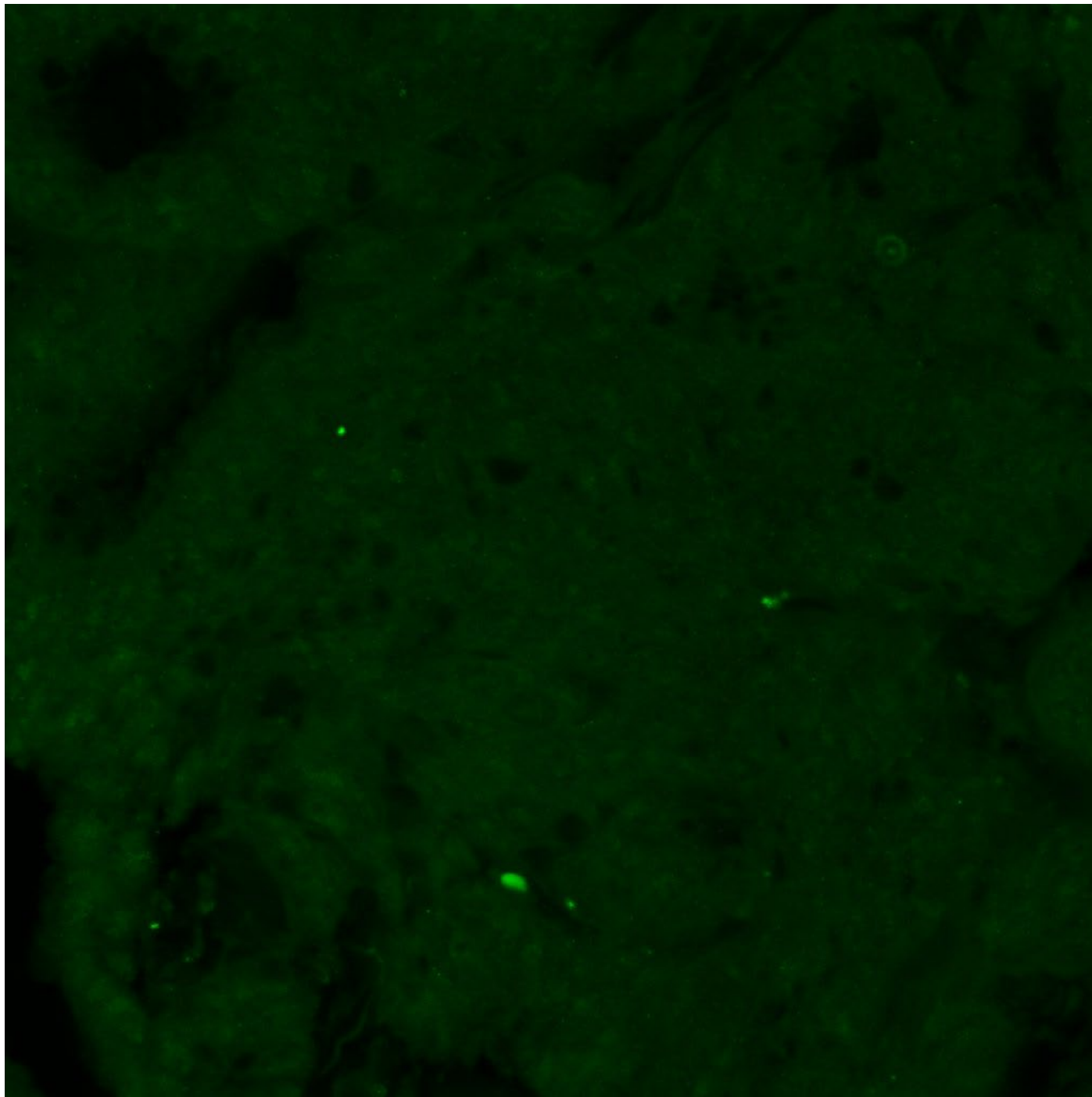

11086\_03

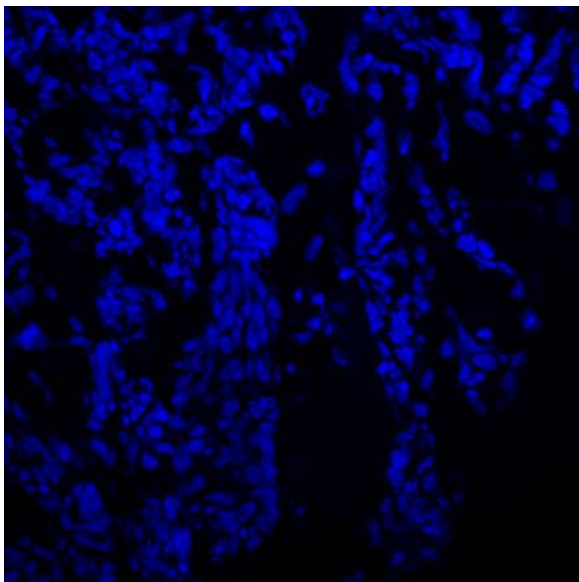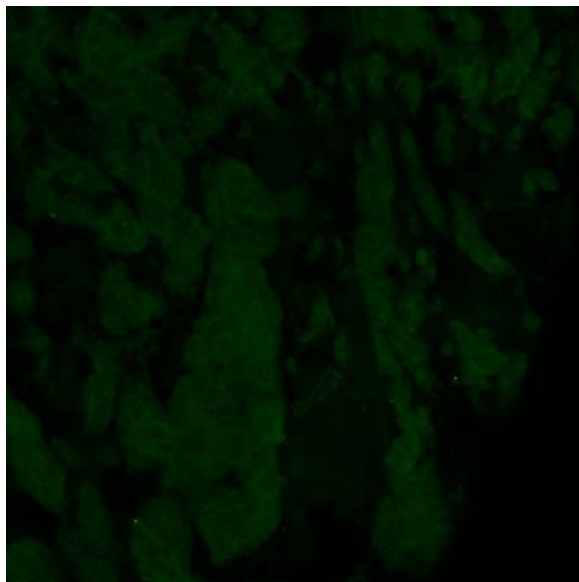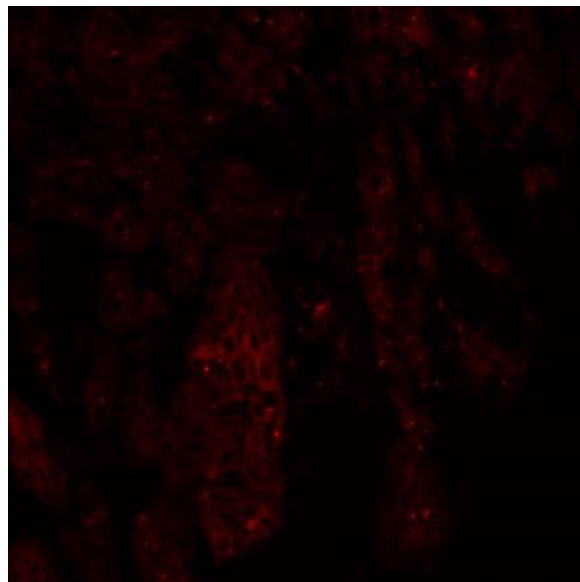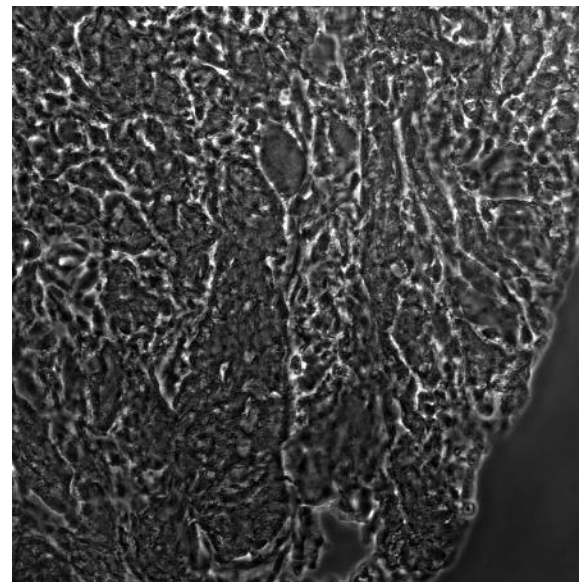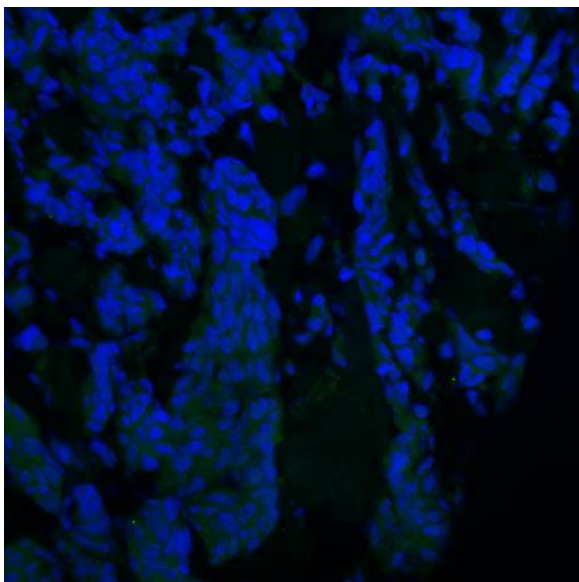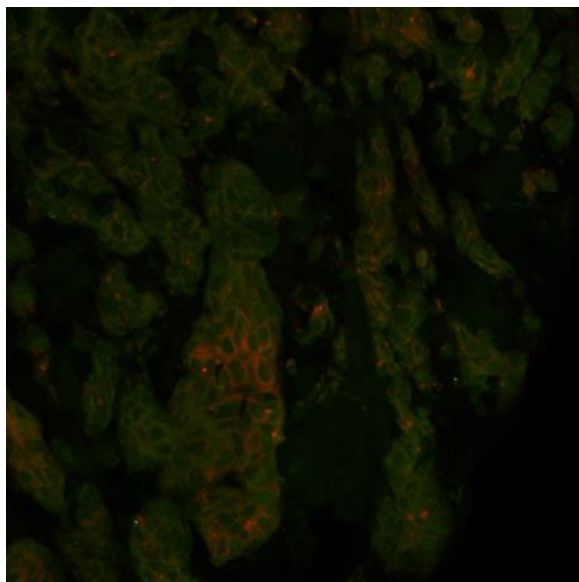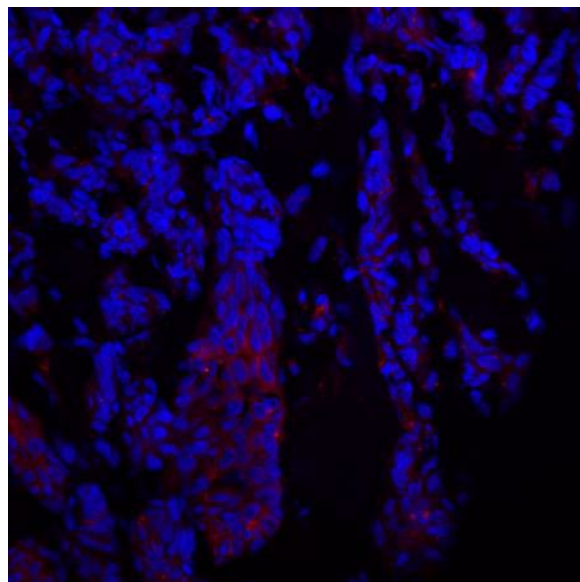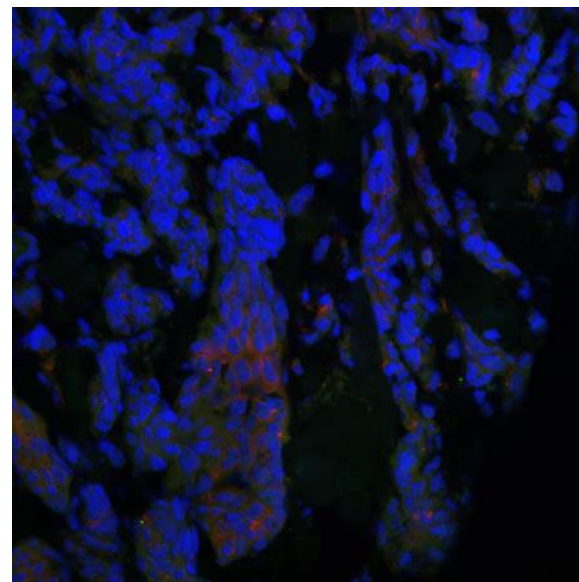

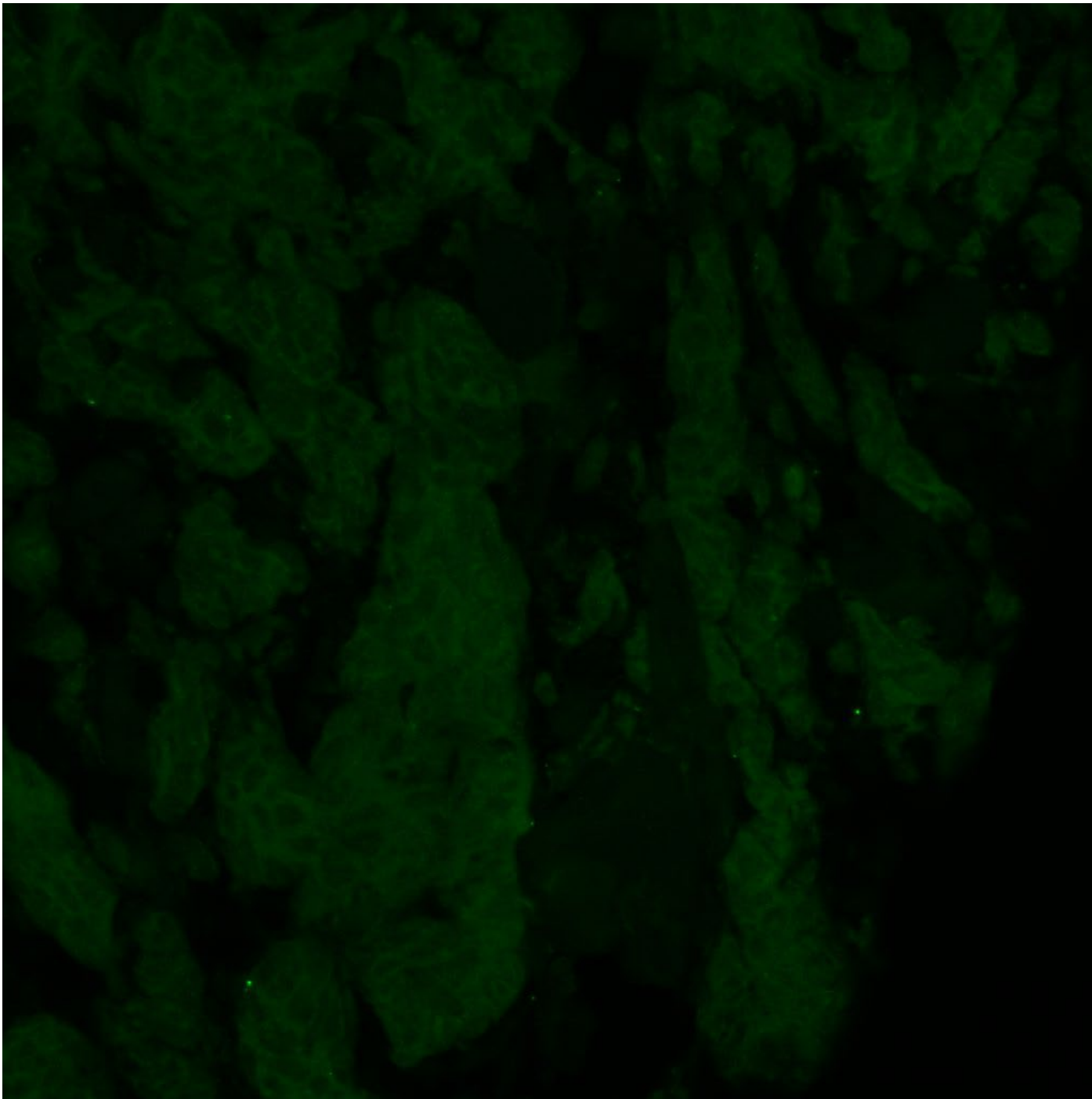

11086\_04

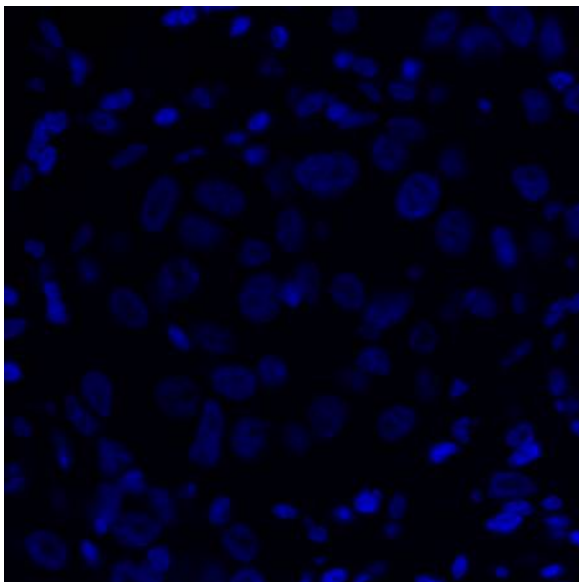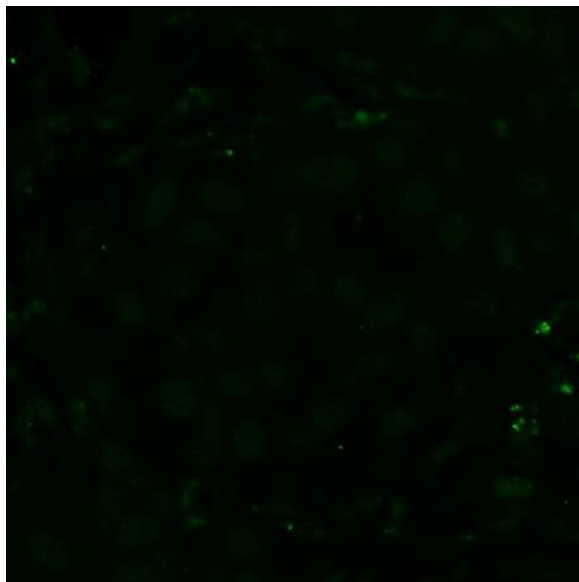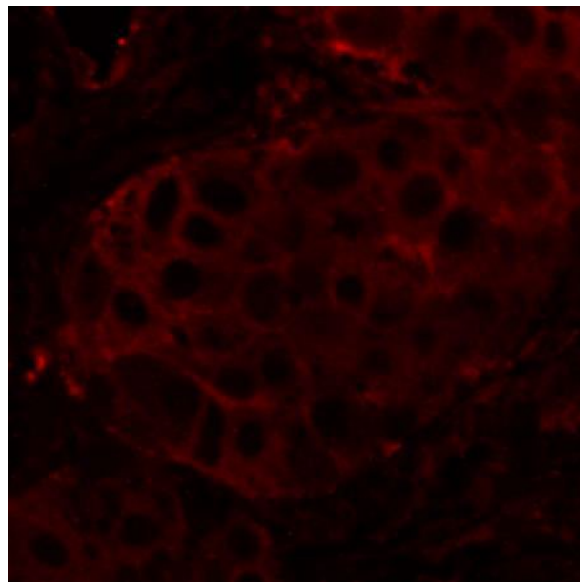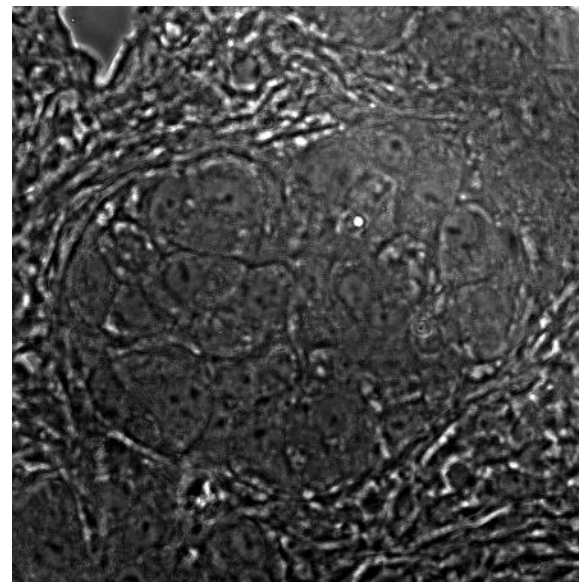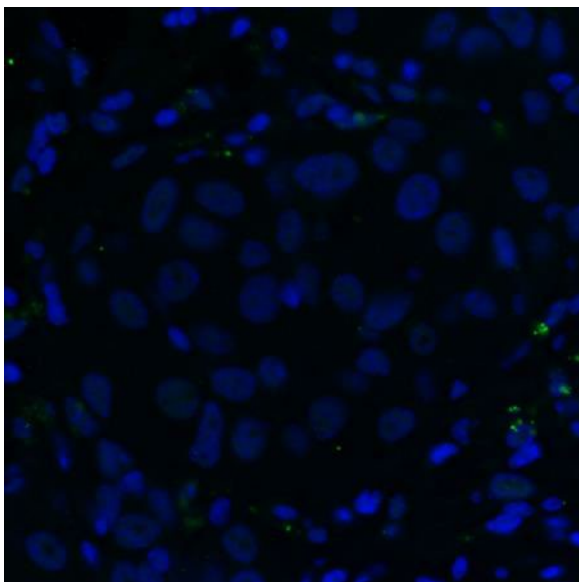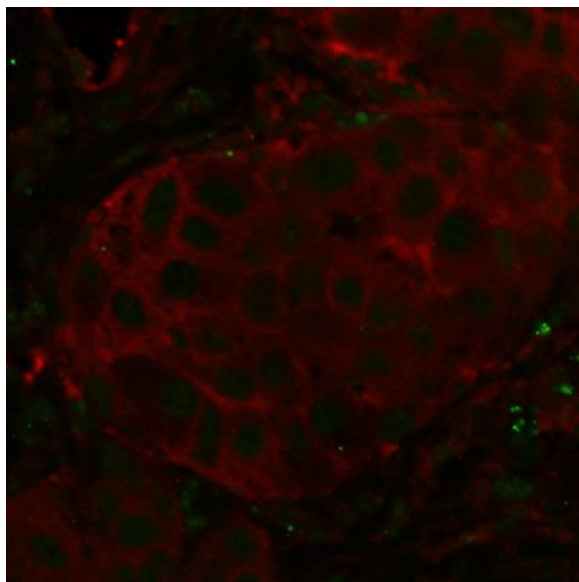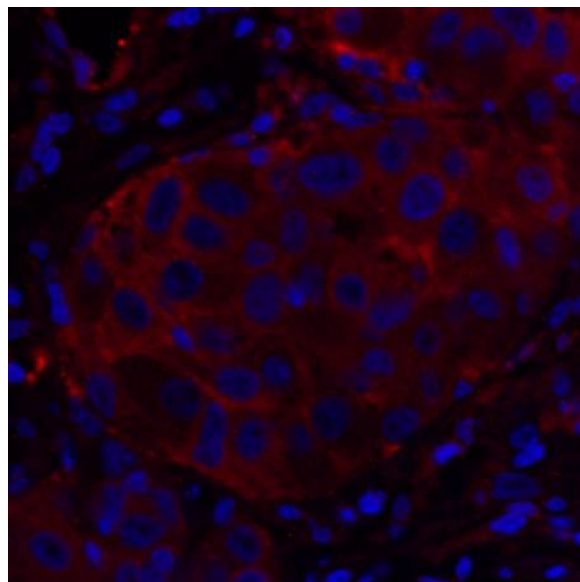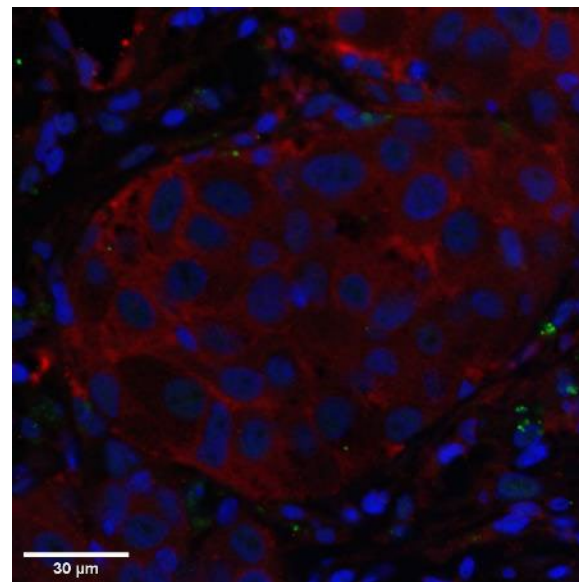

15542\_00

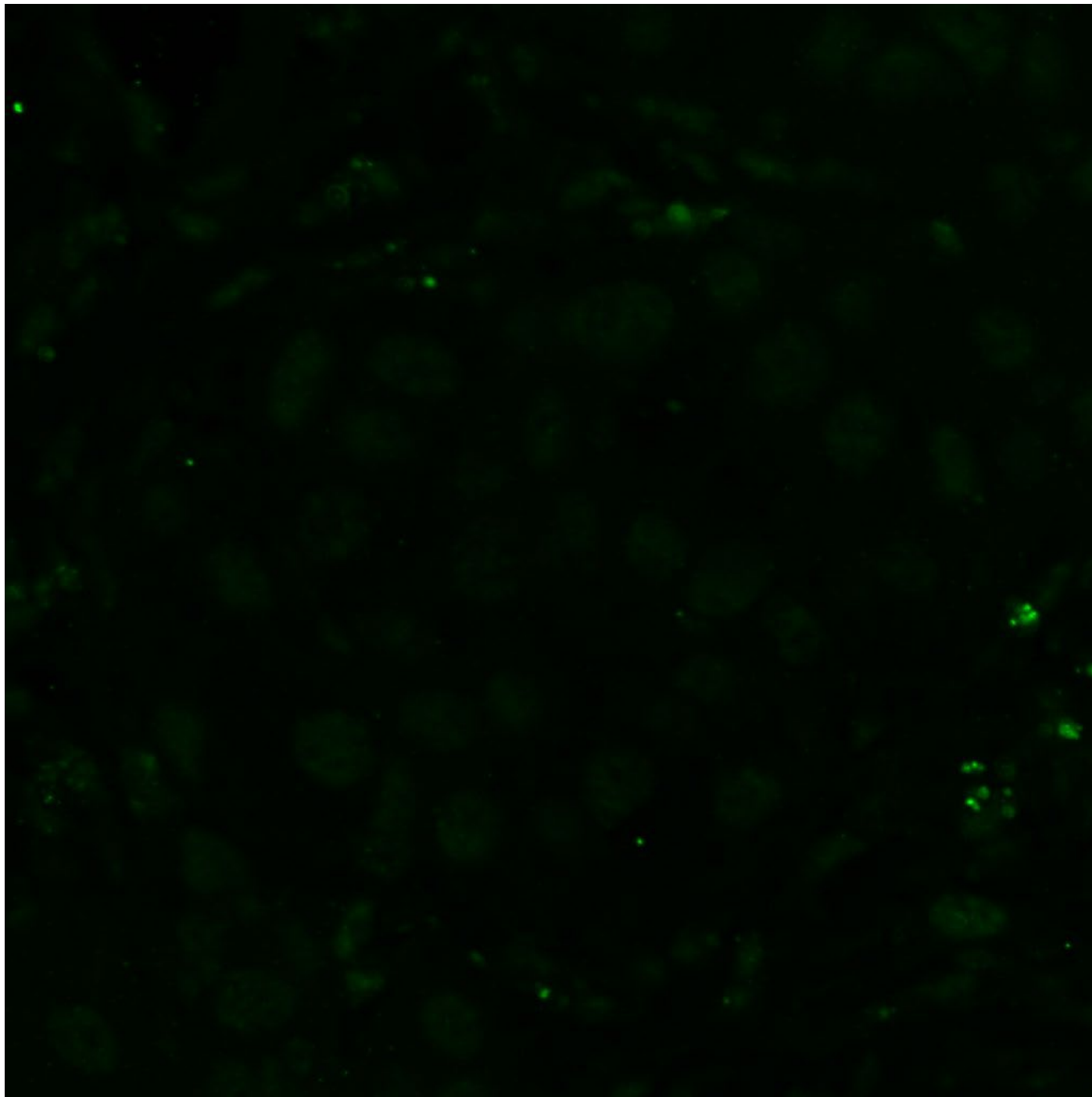

15542\_00

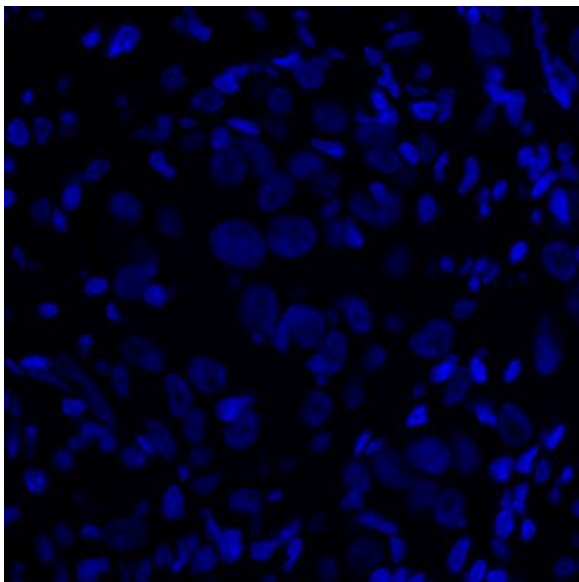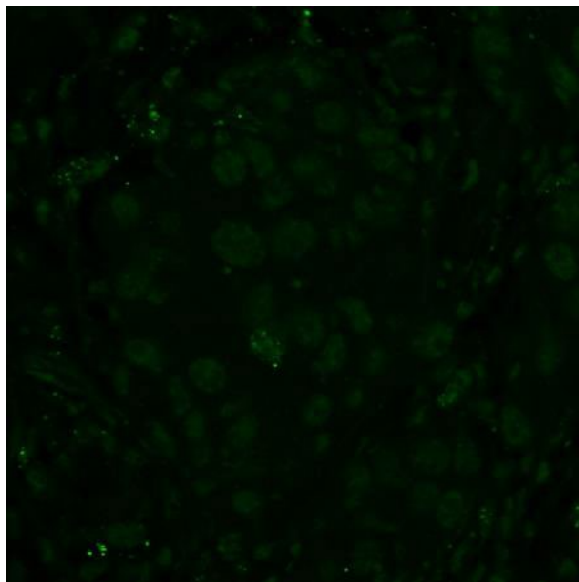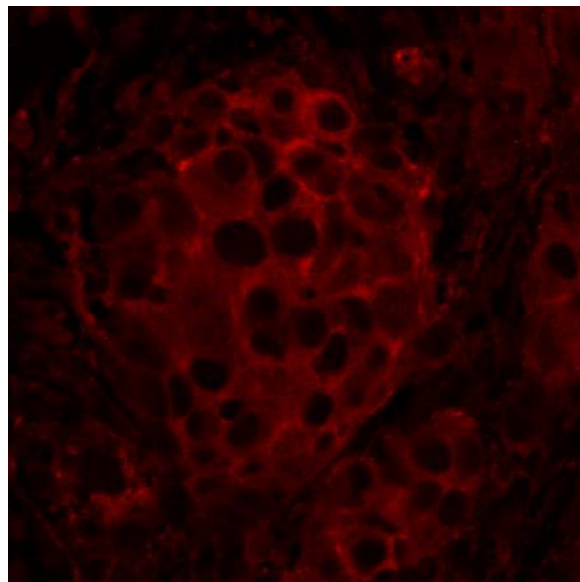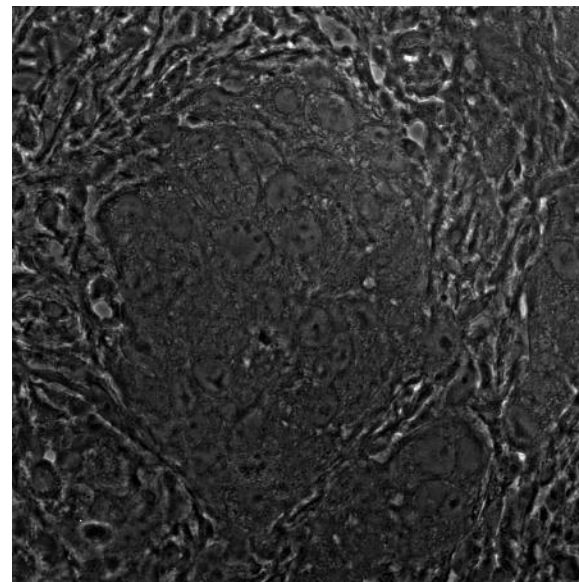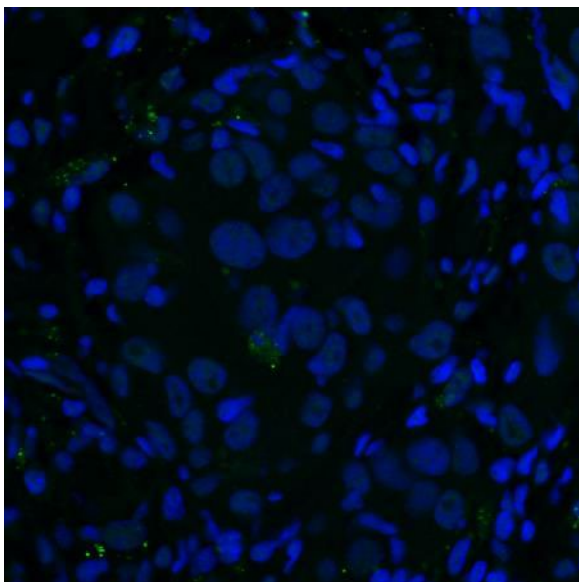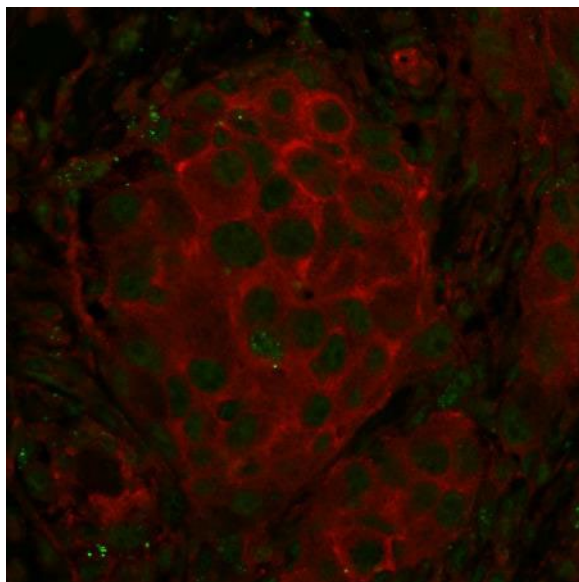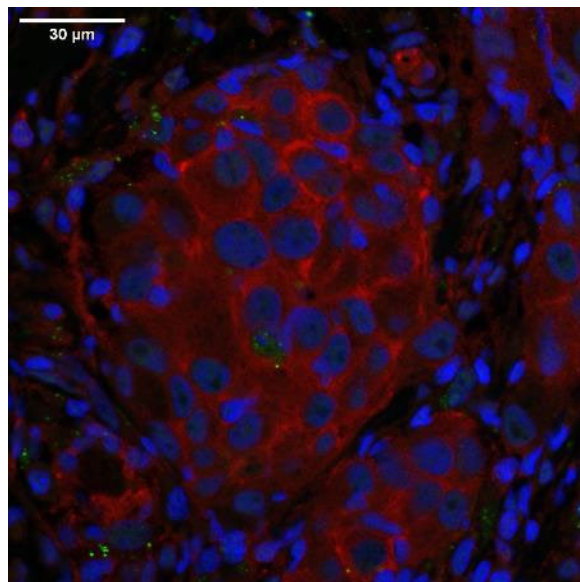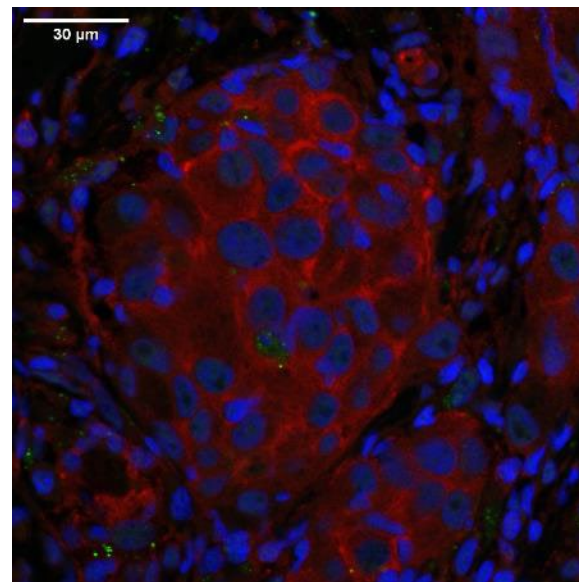

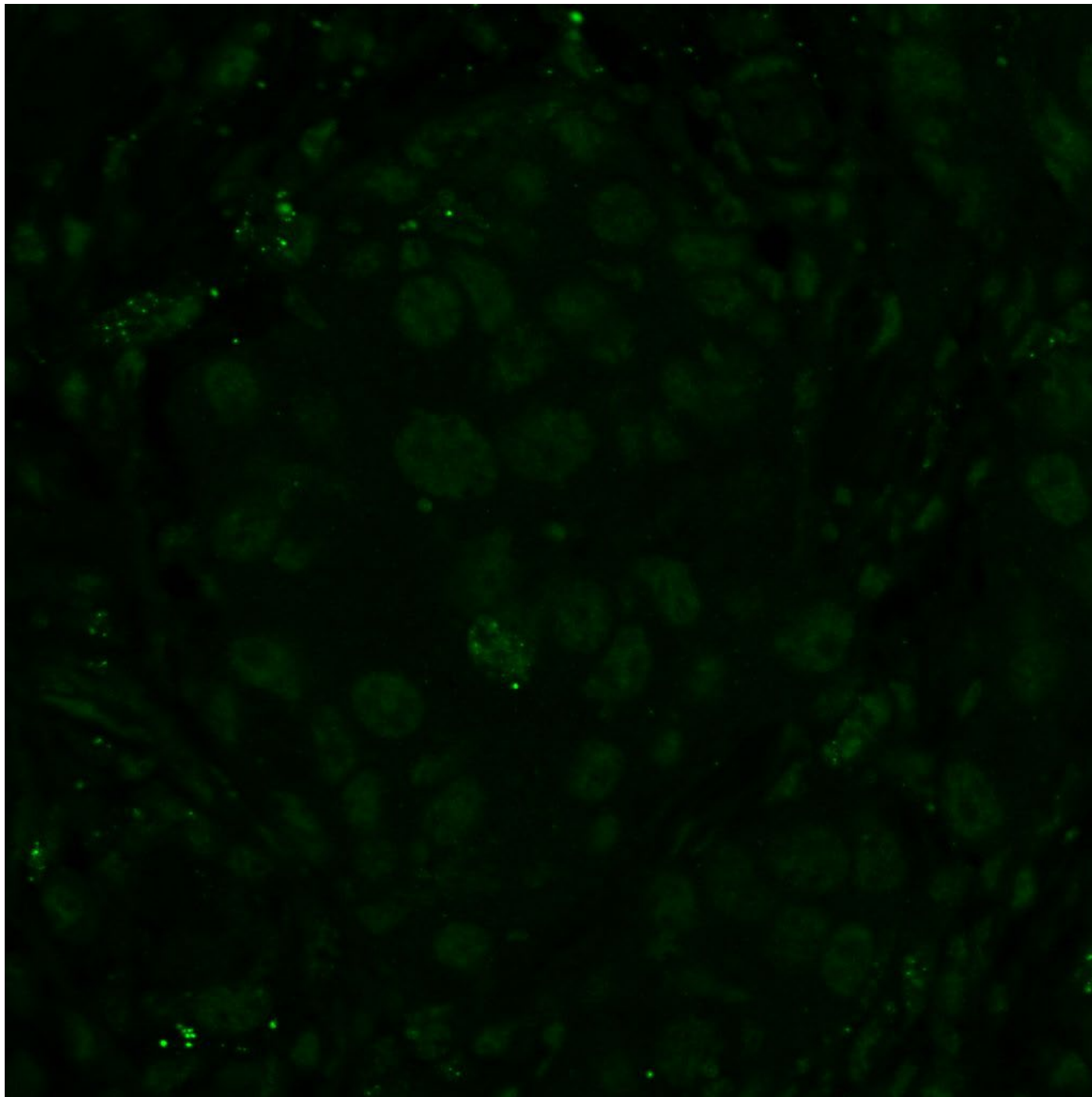

15542\_01

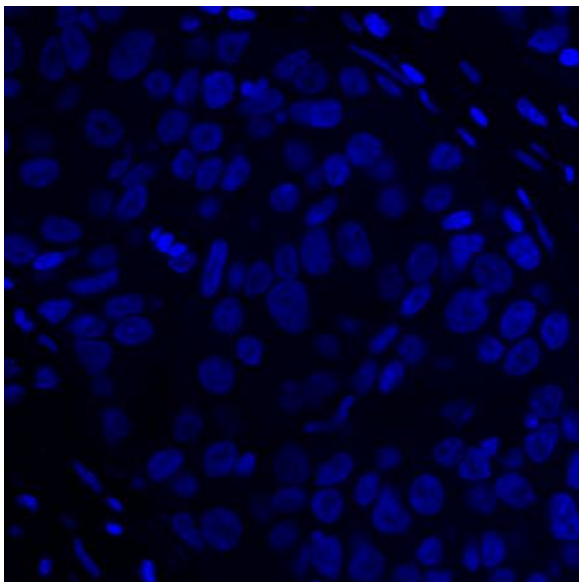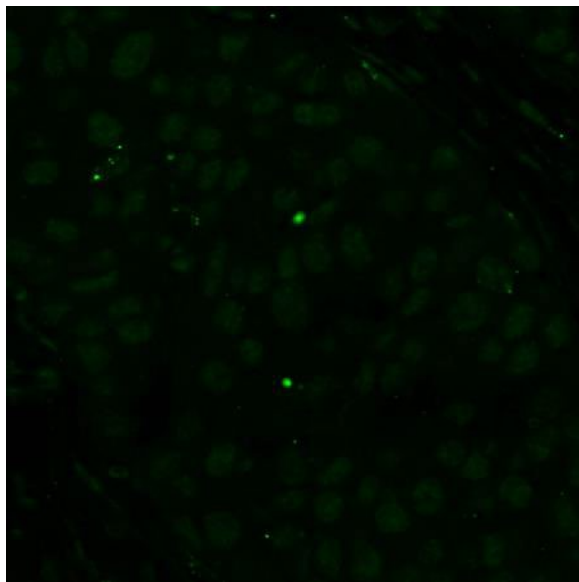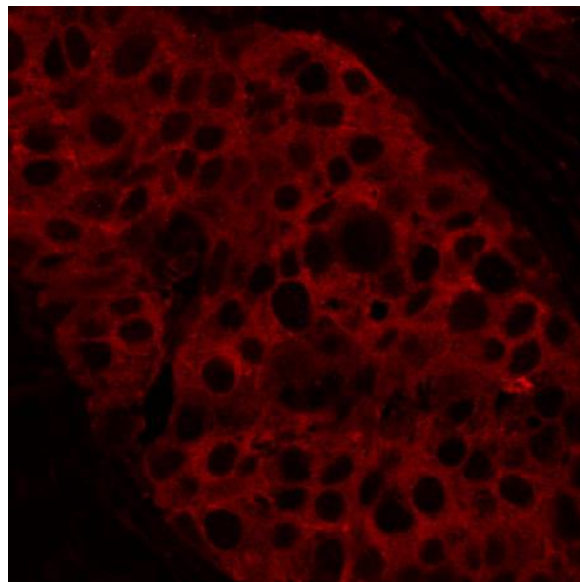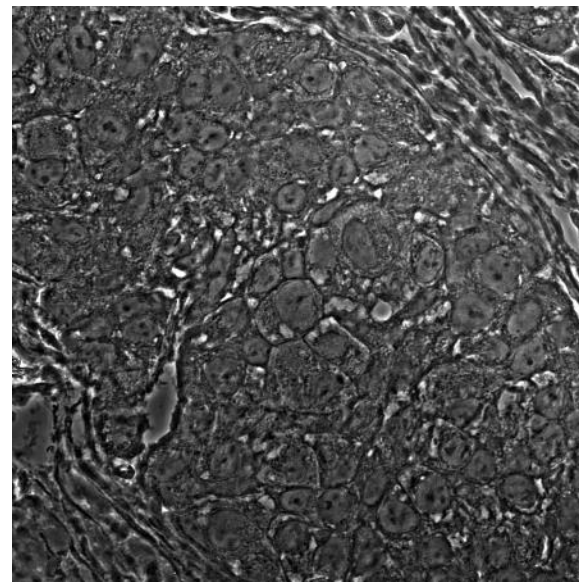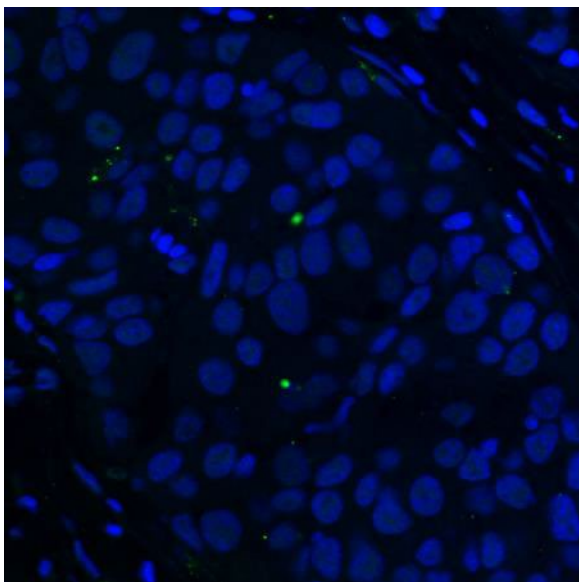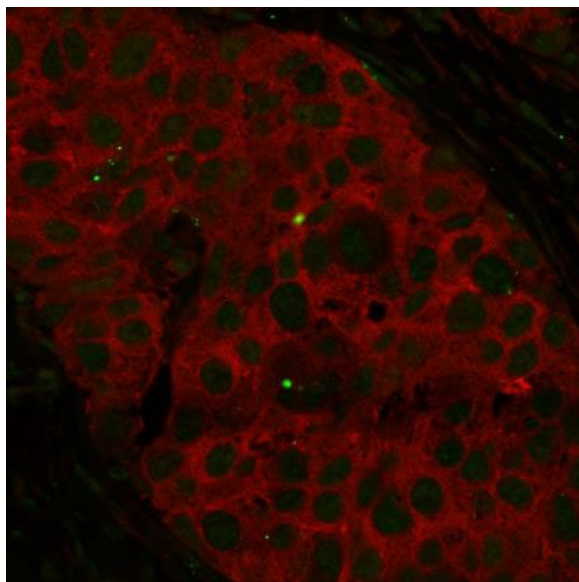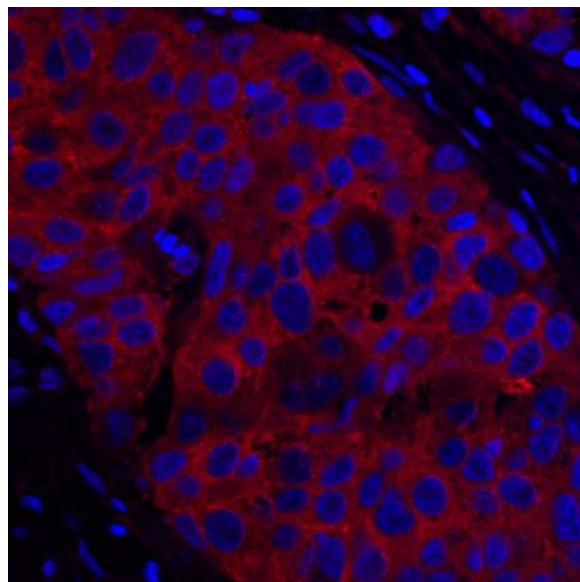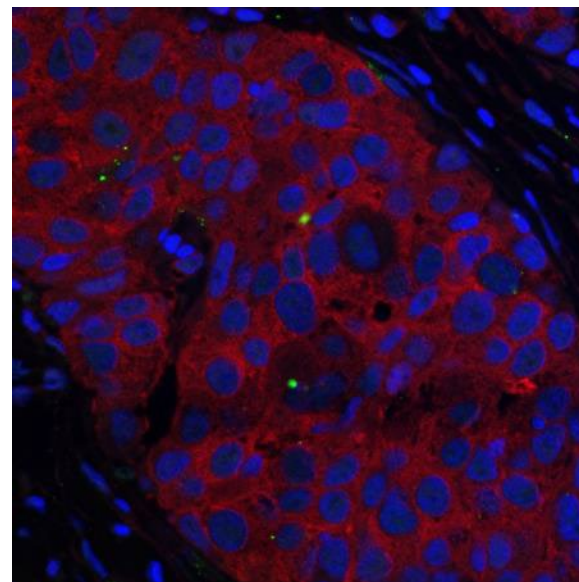

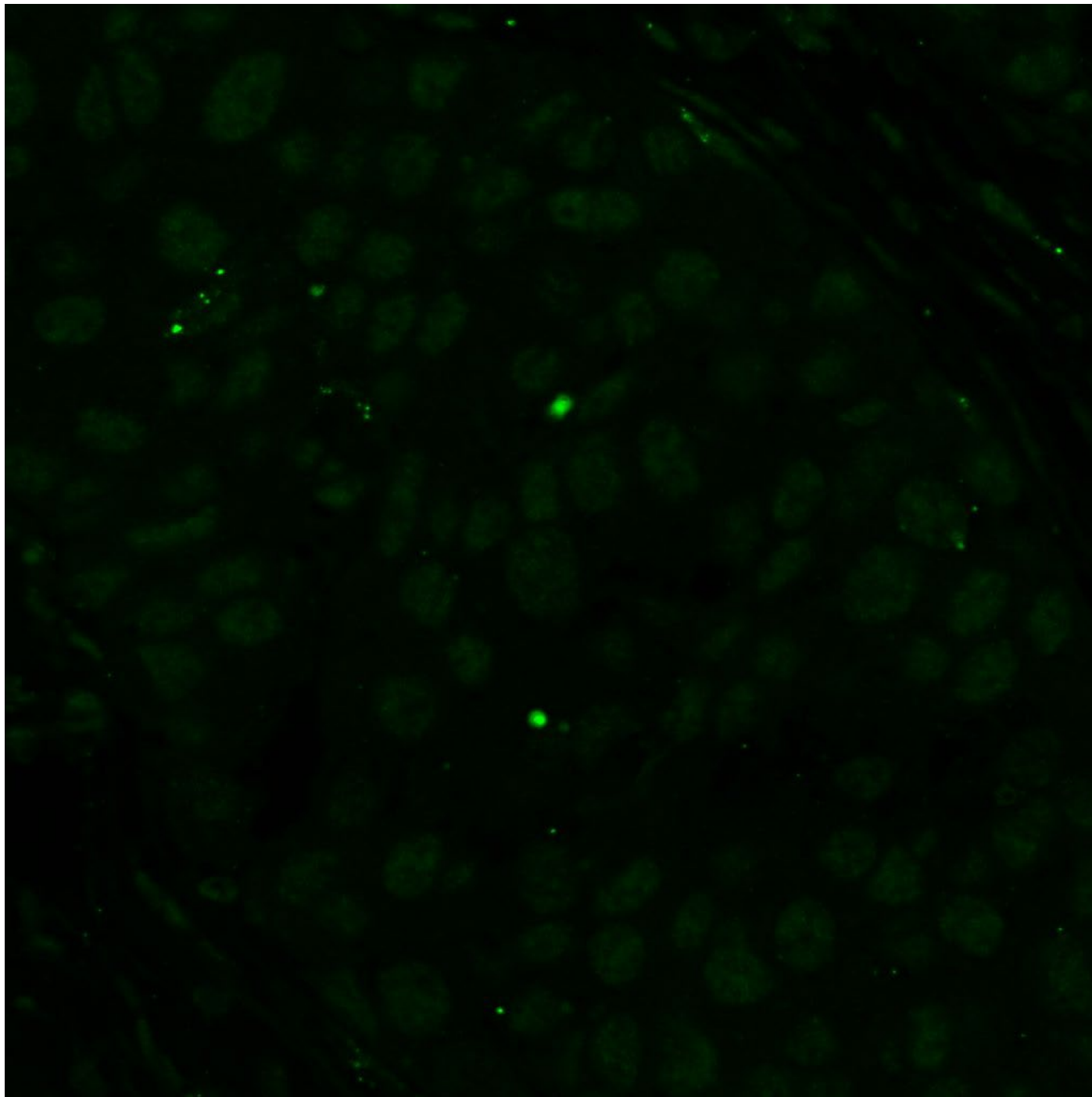

15542\_02

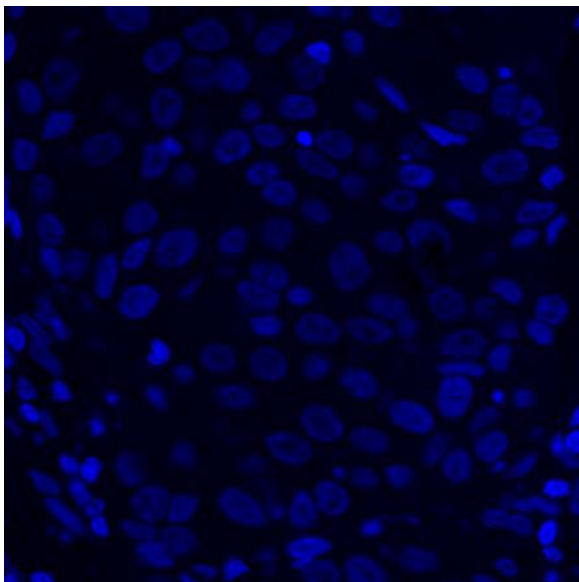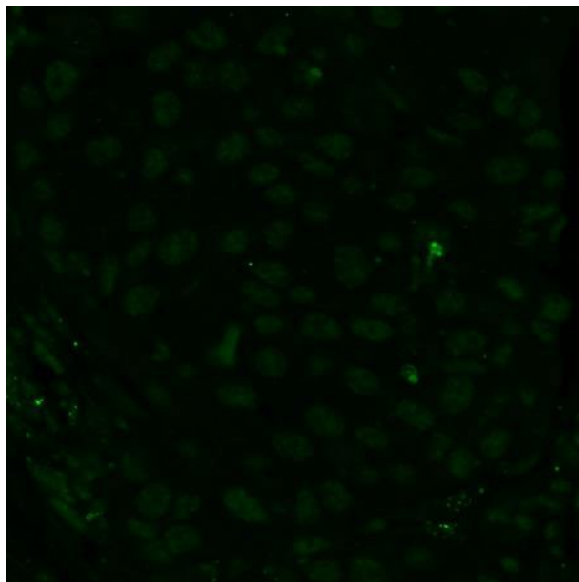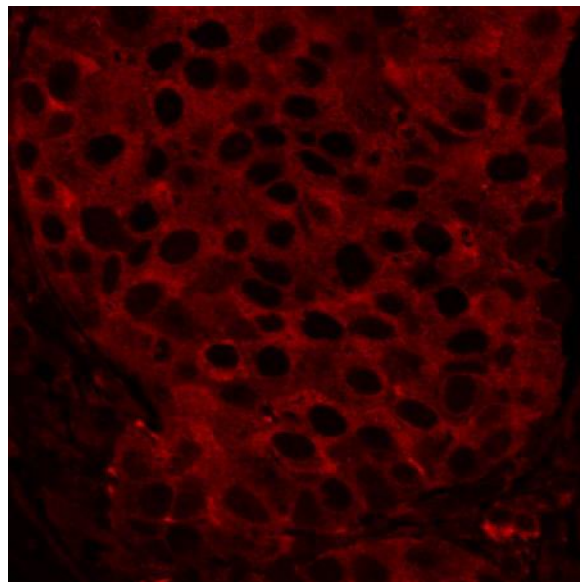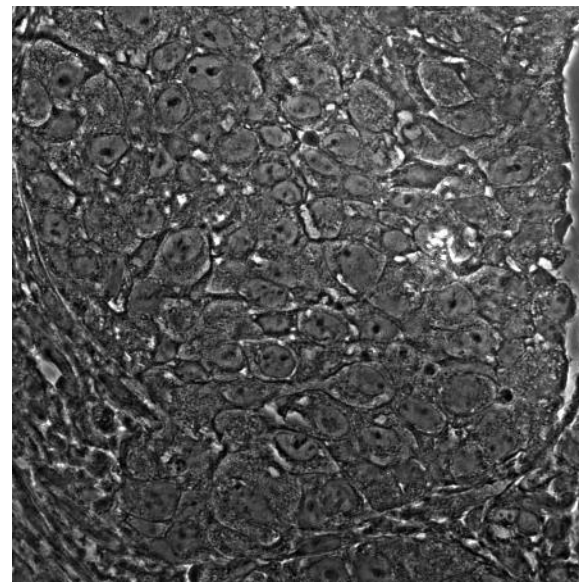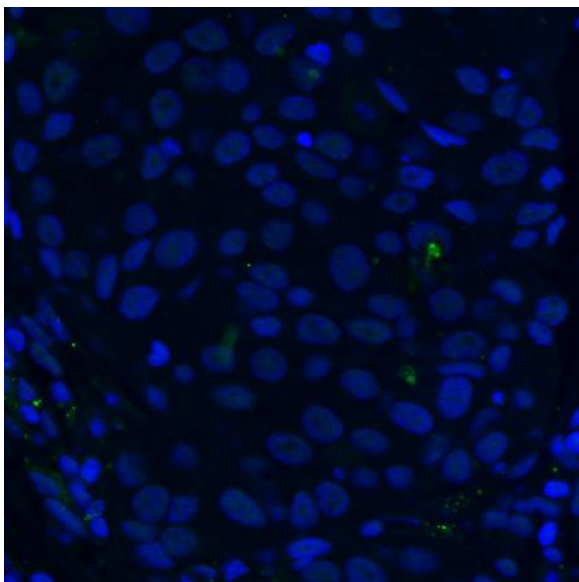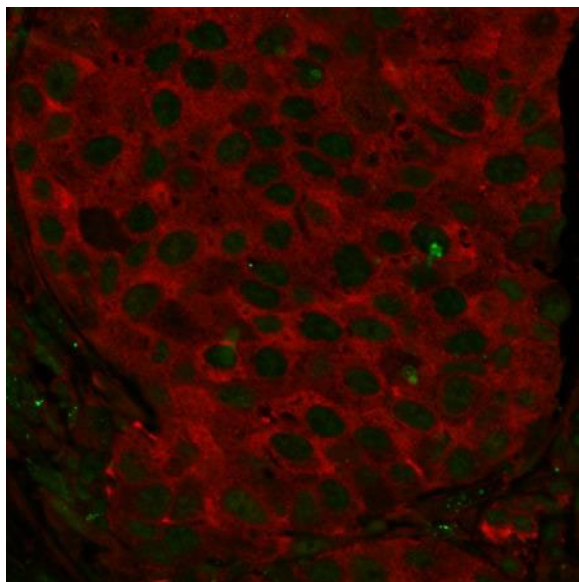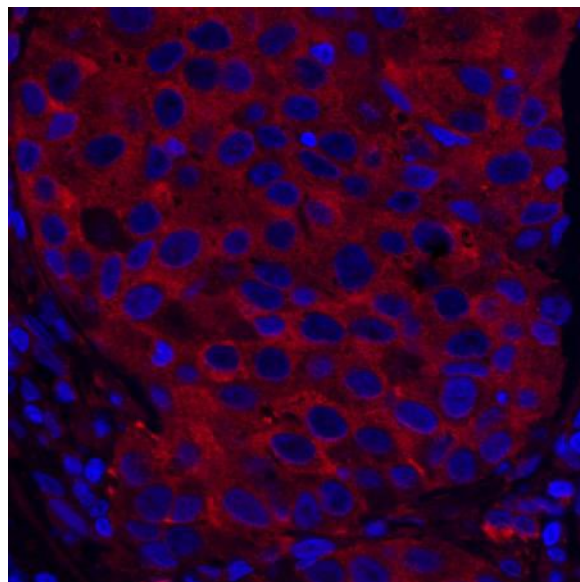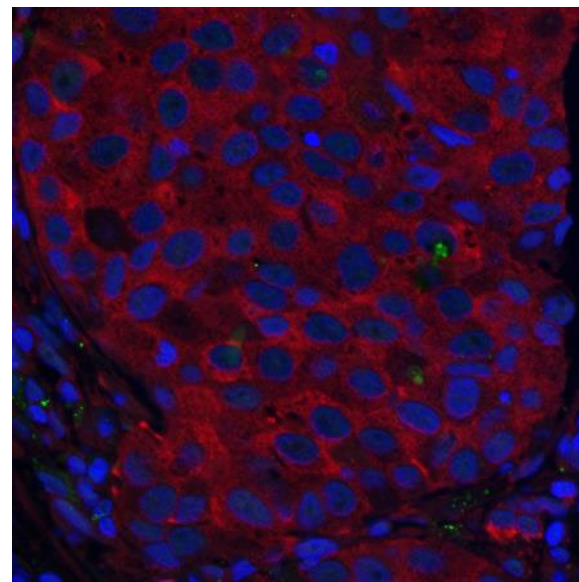

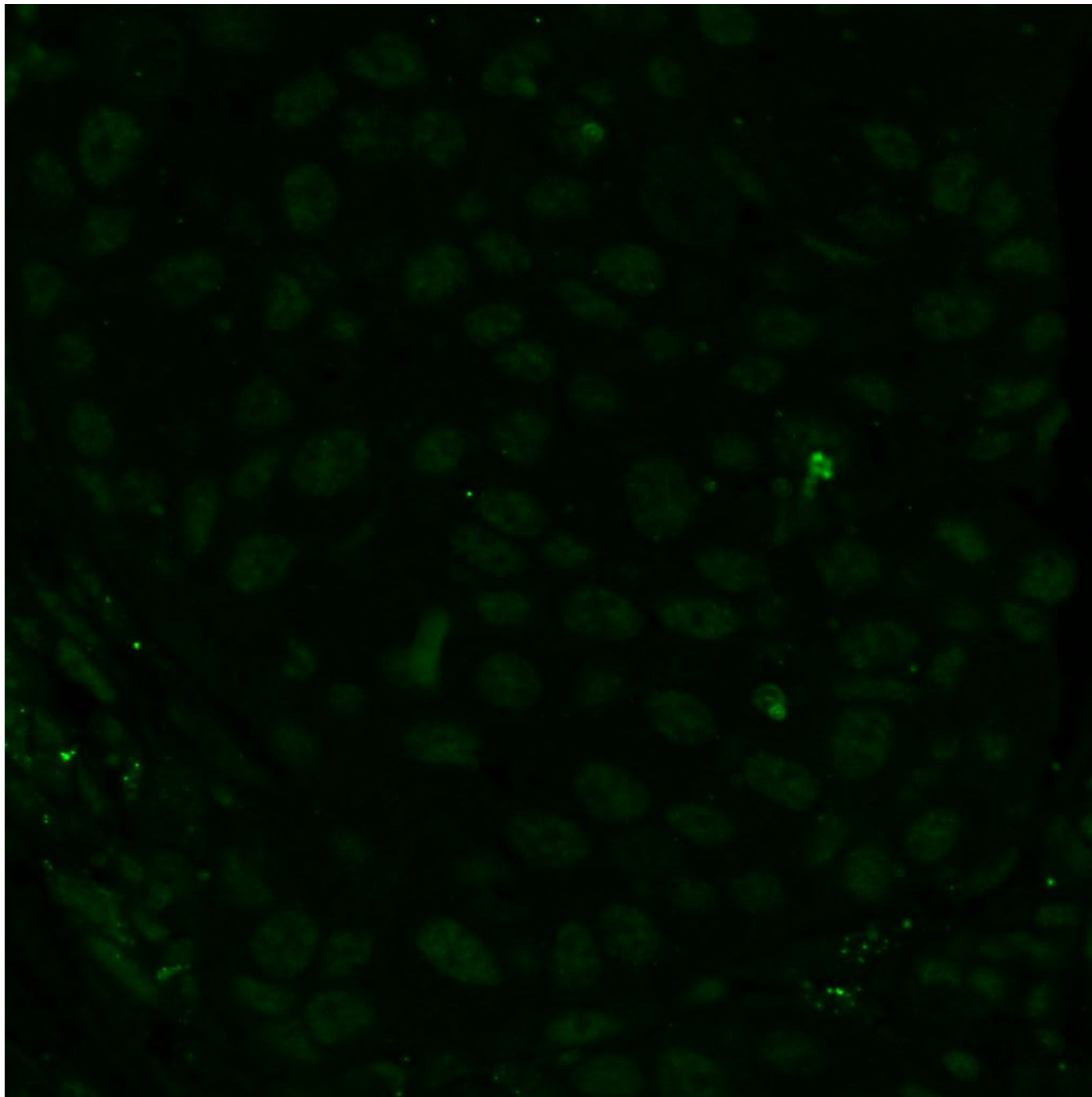

15542\_03

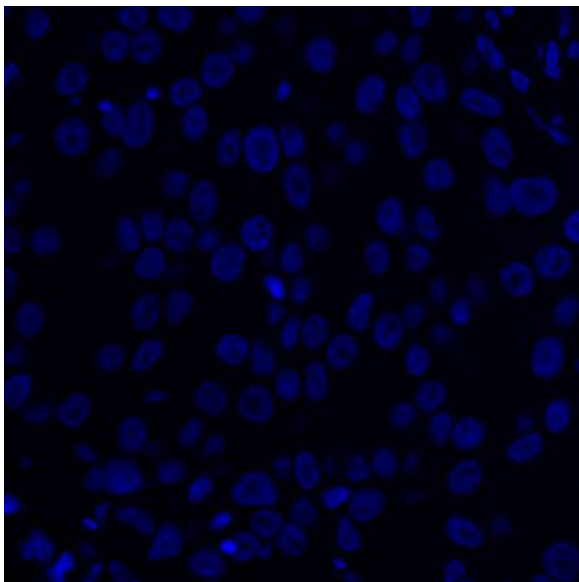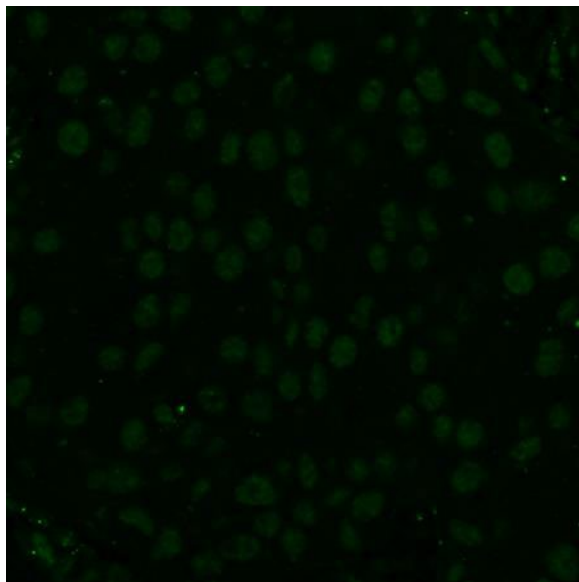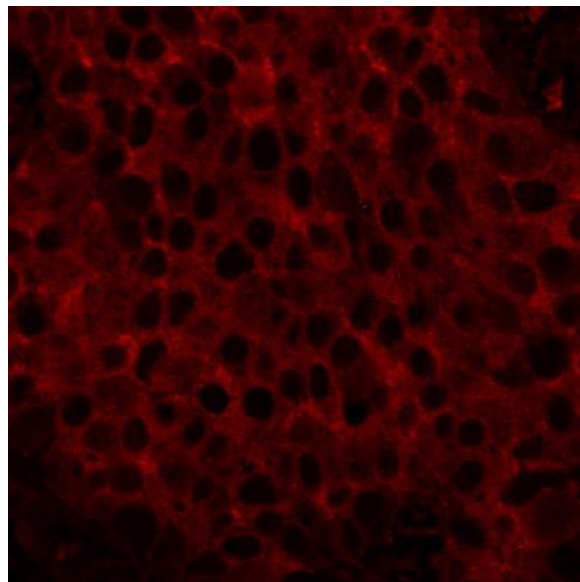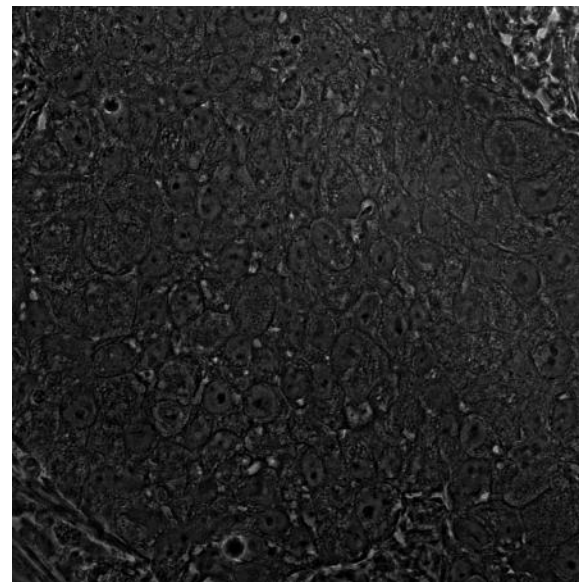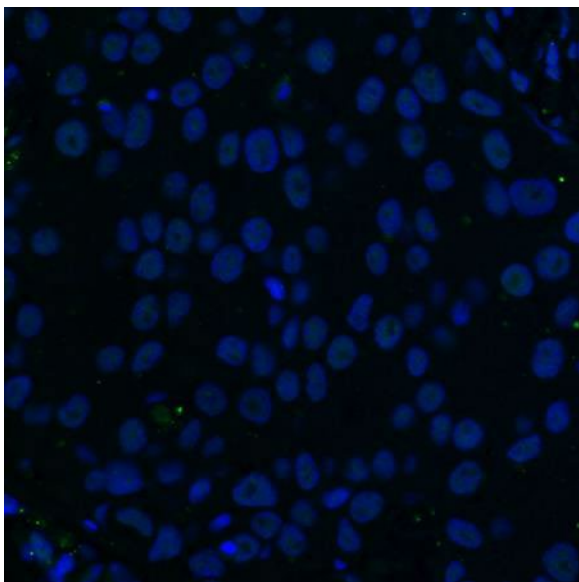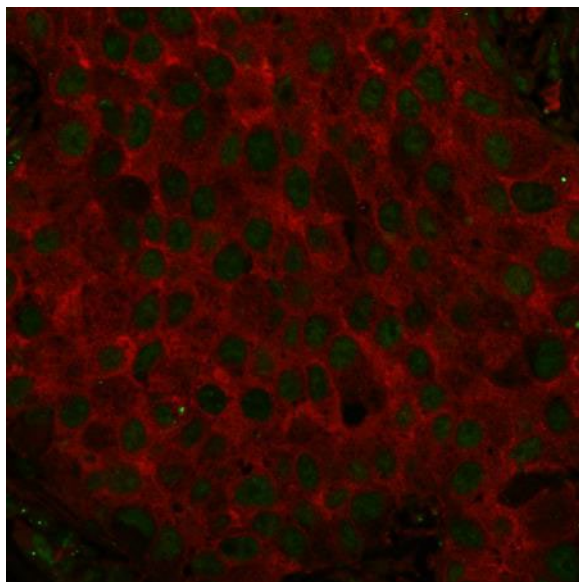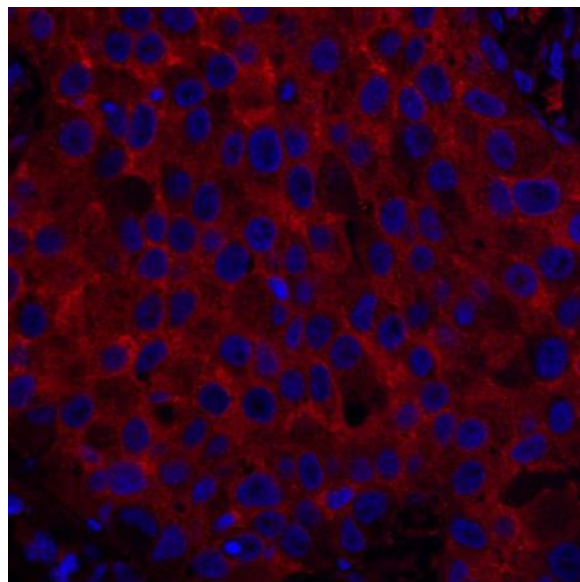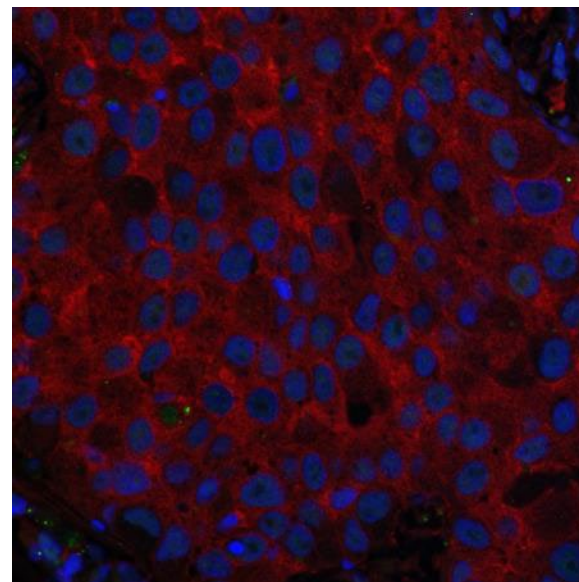

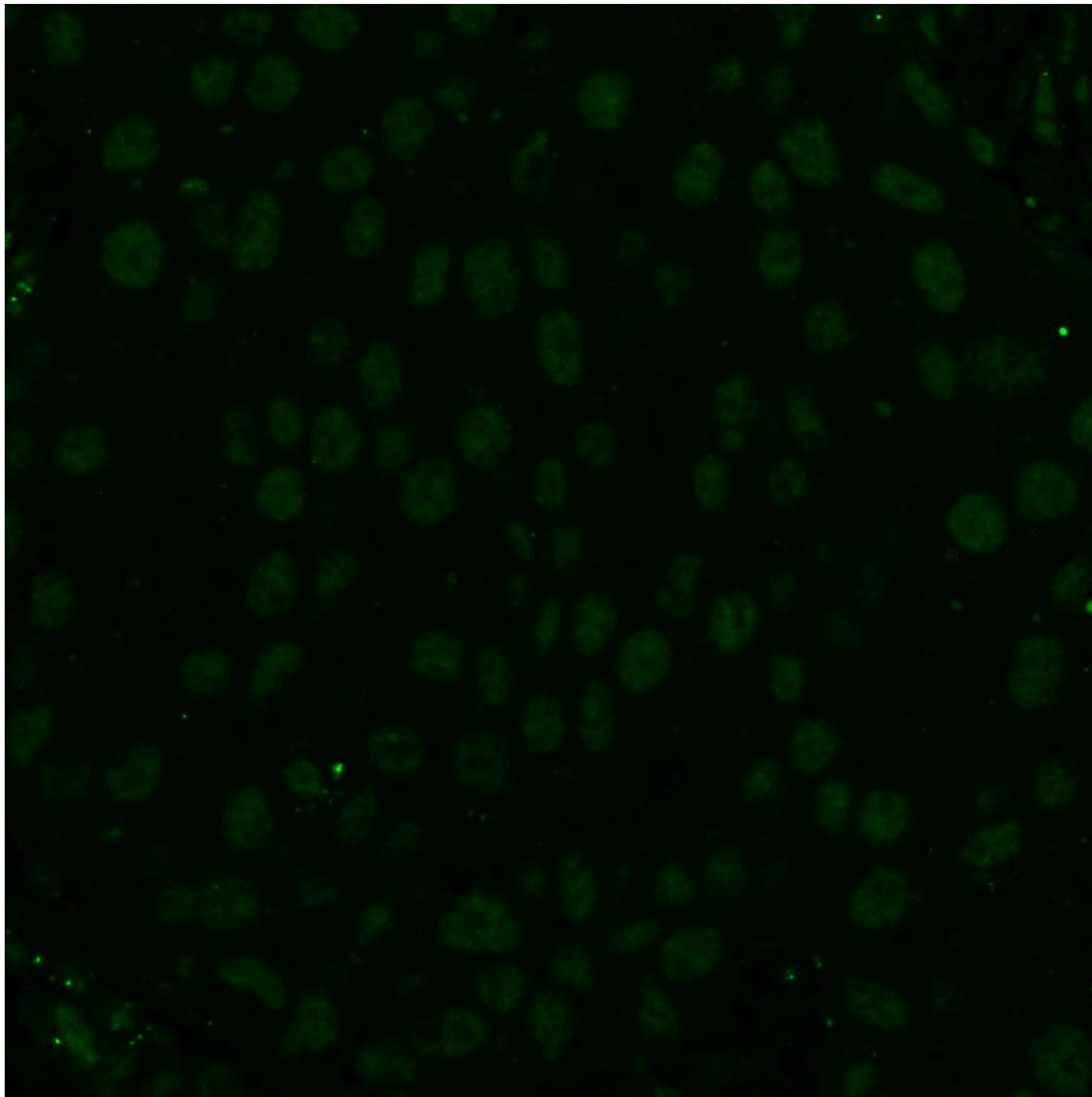

15542\_04

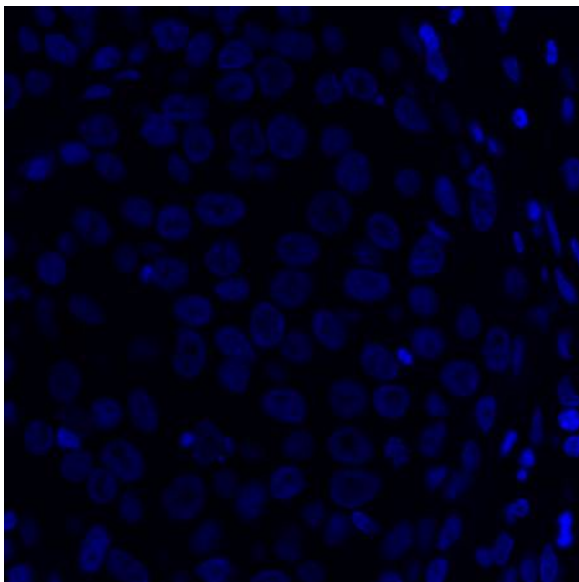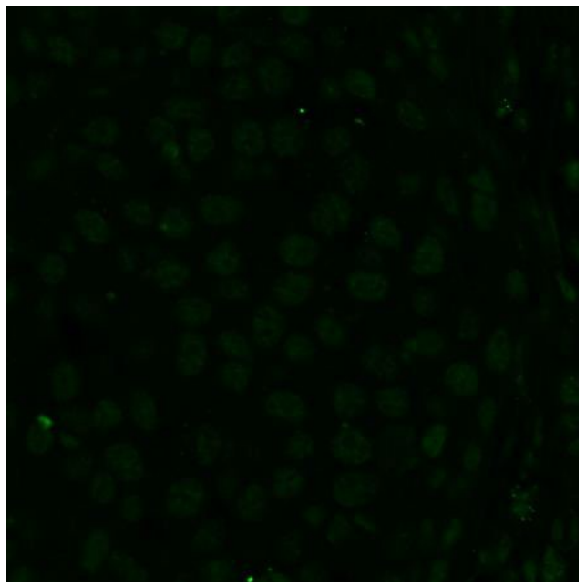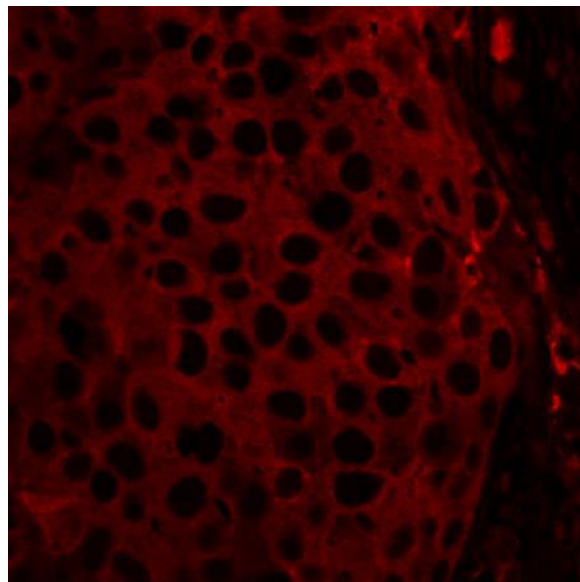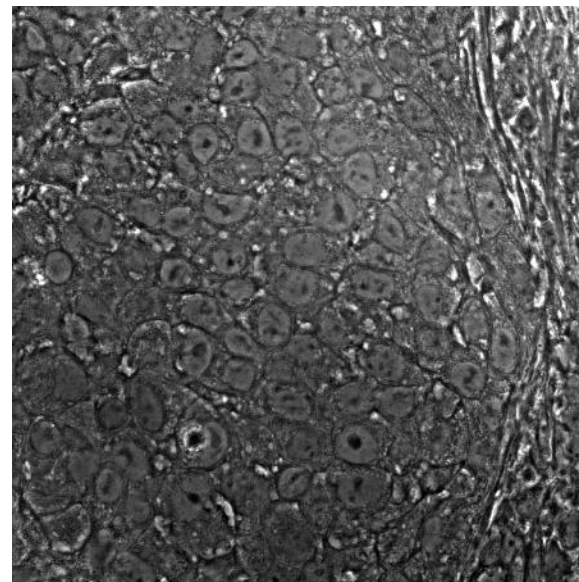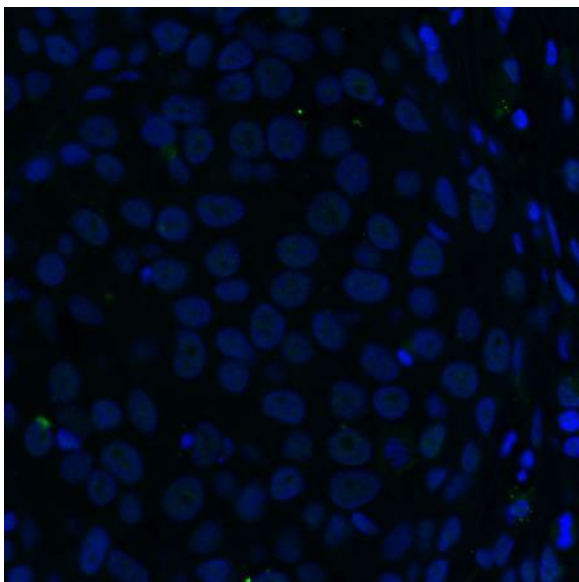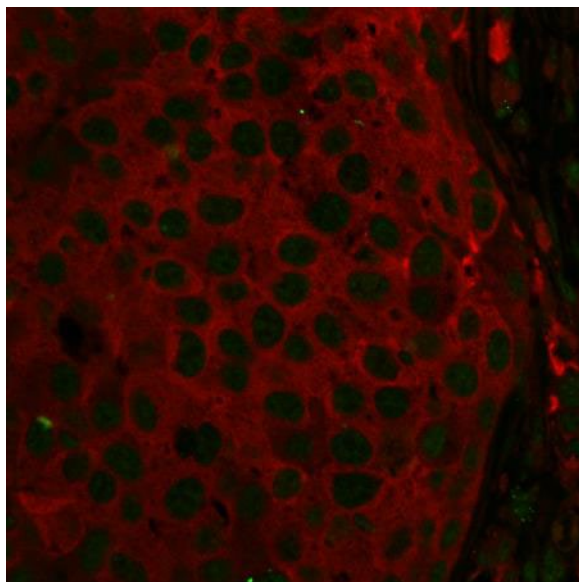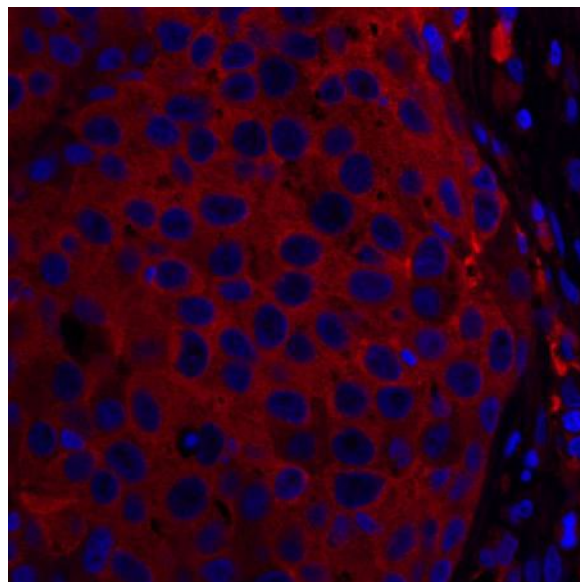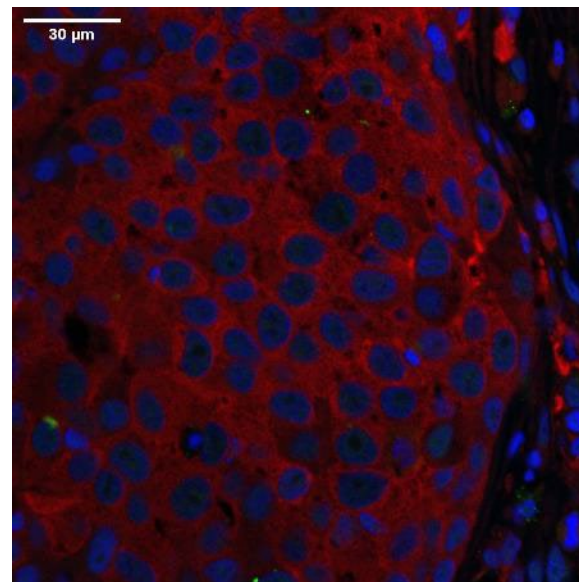

15542\_05

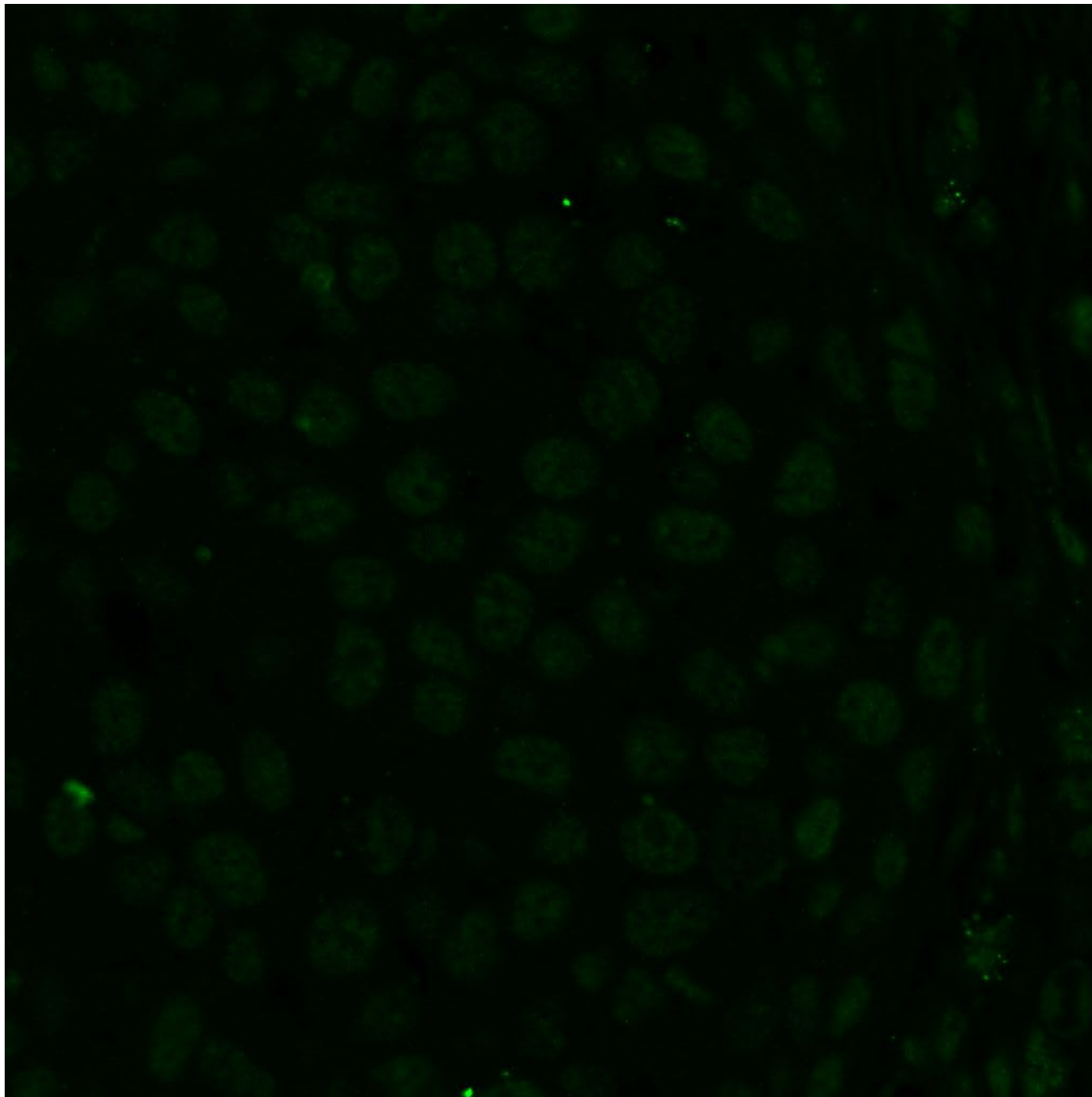

15542\_05

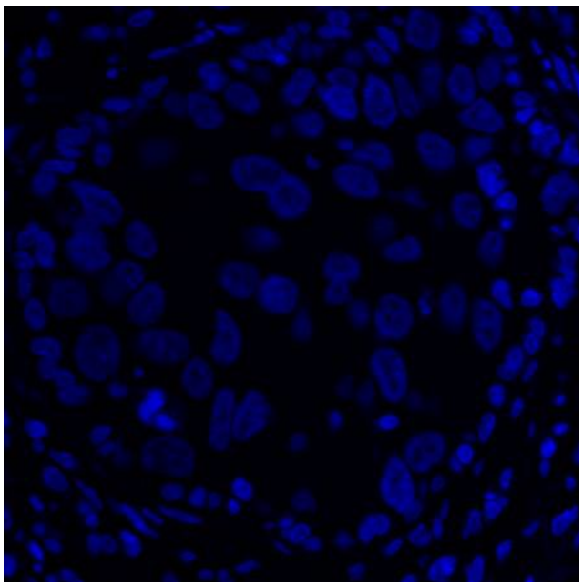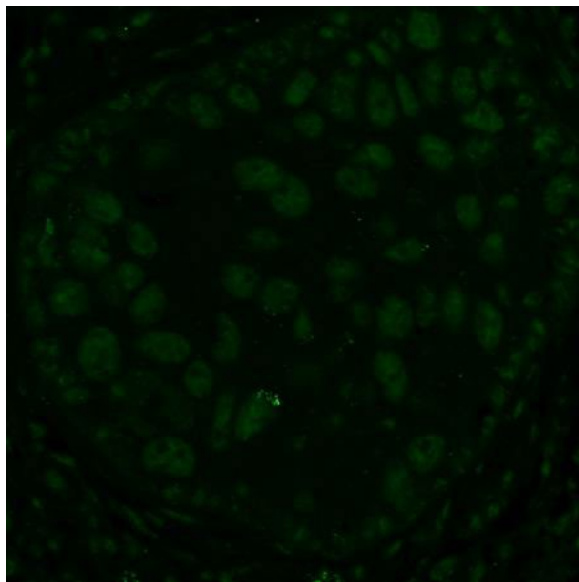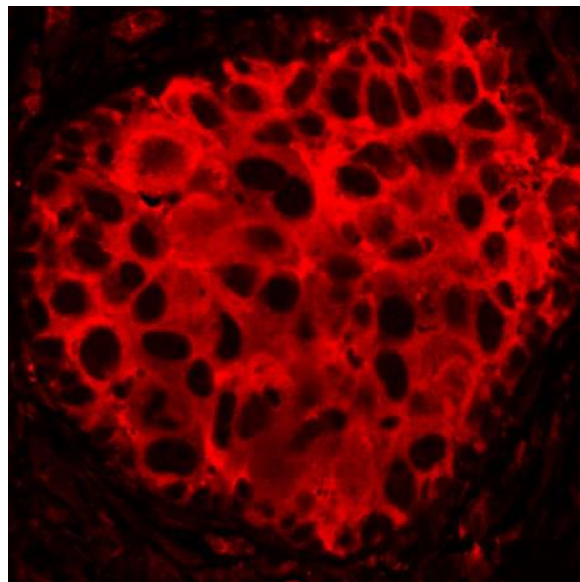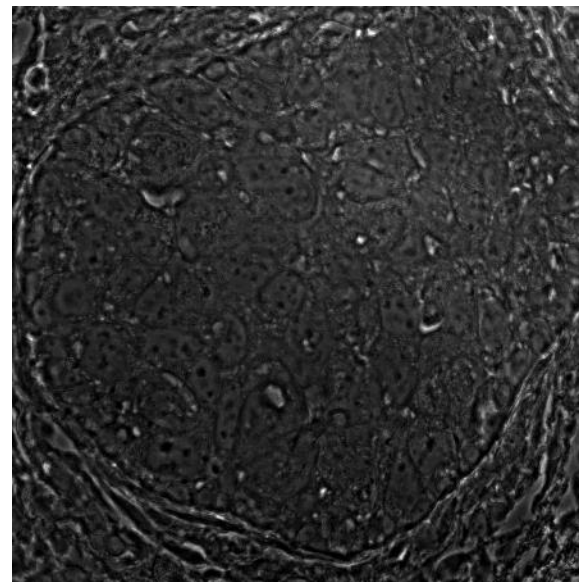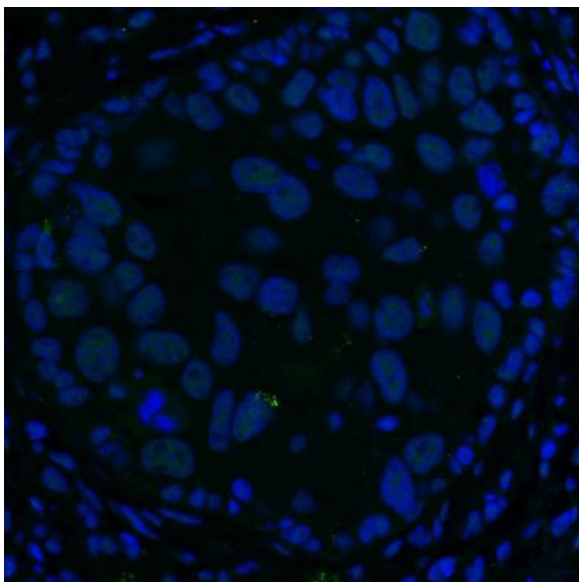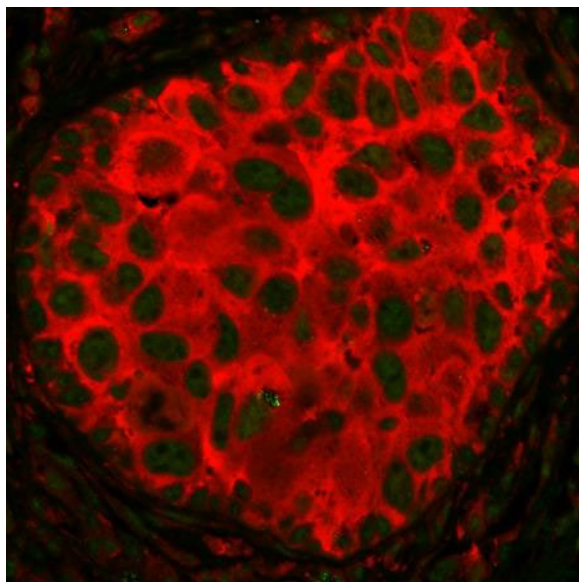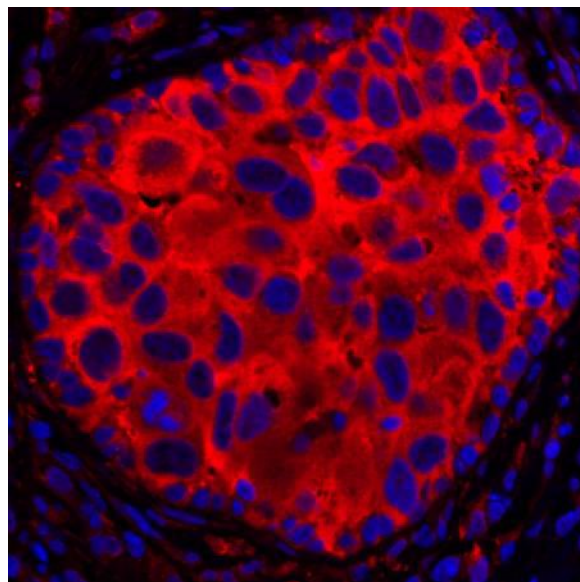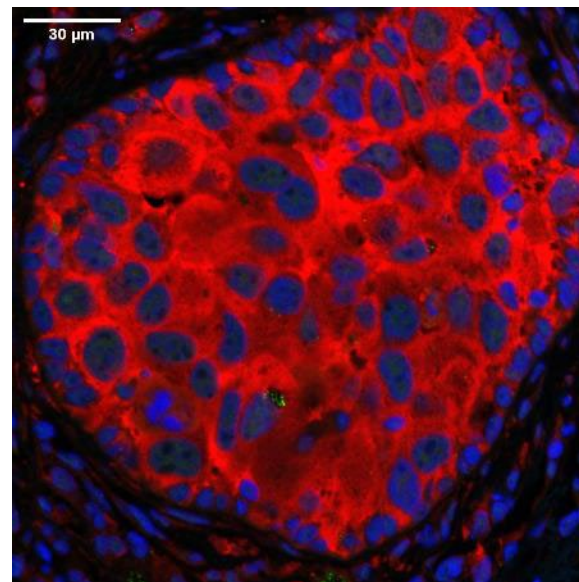

15542\_06

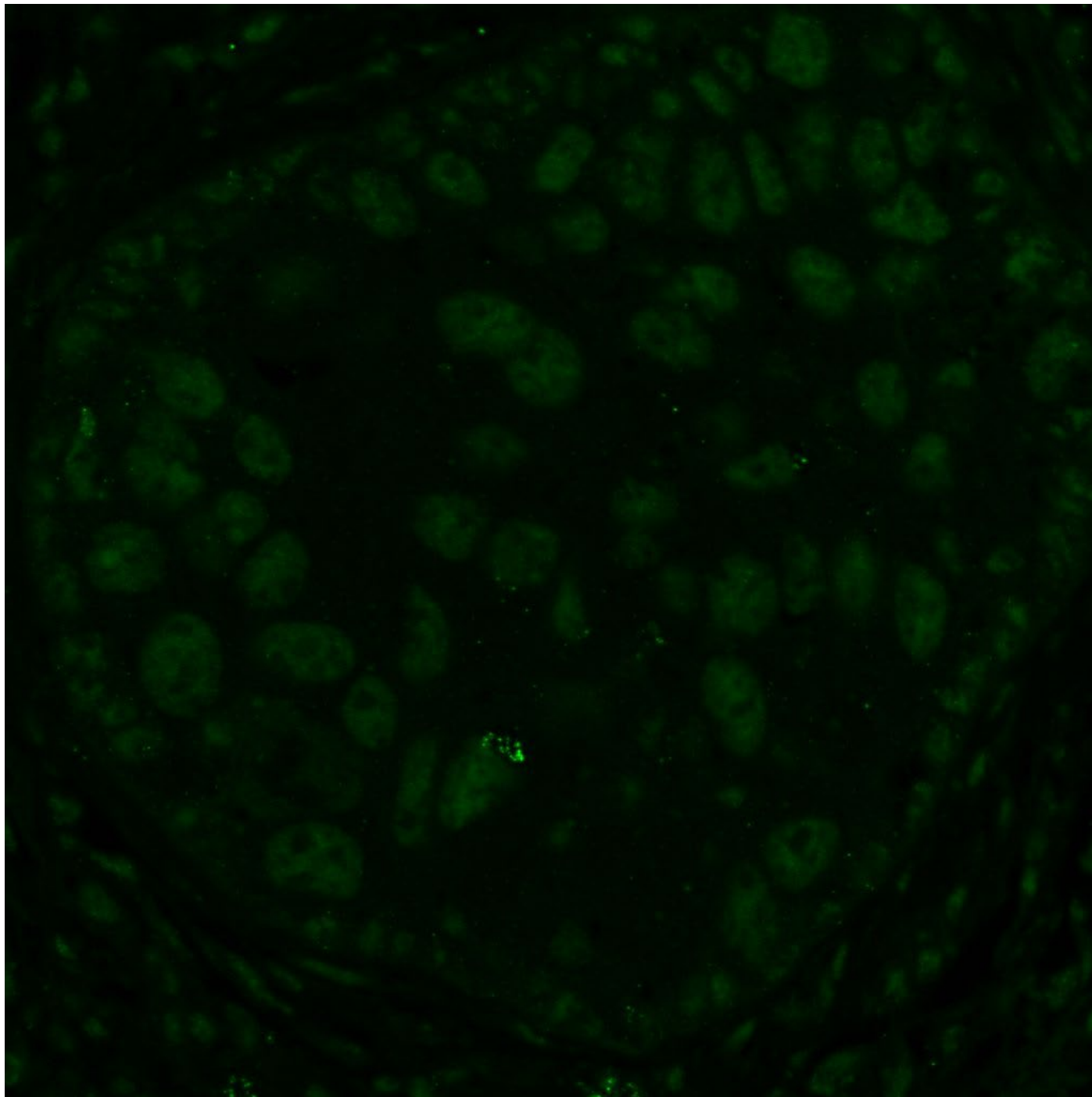

15542\_06

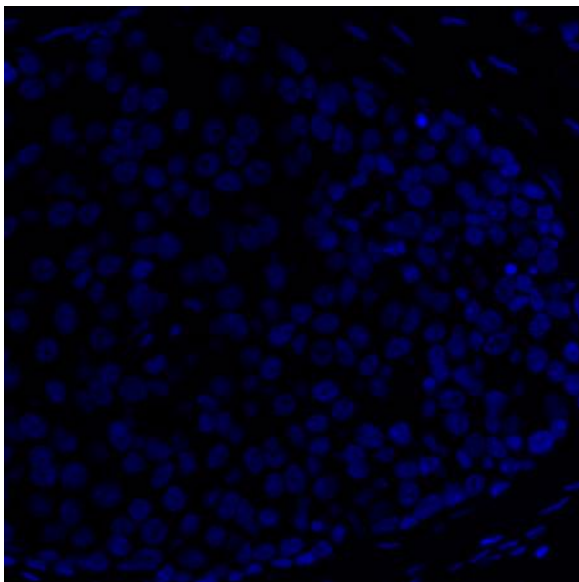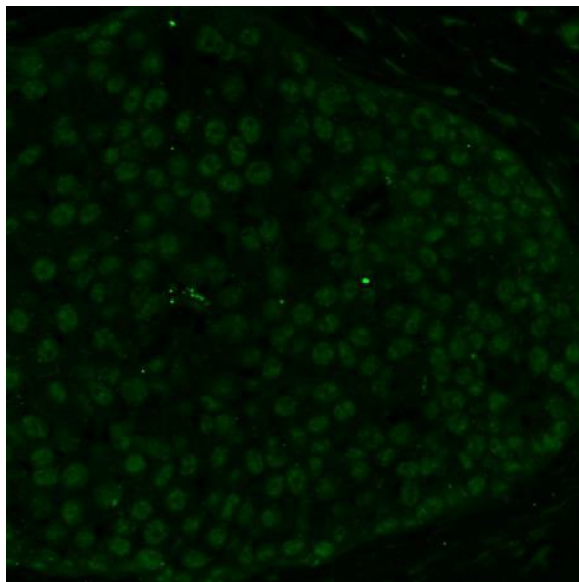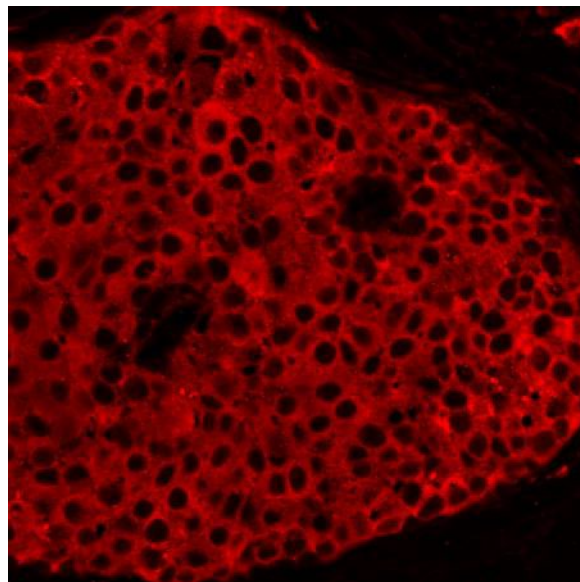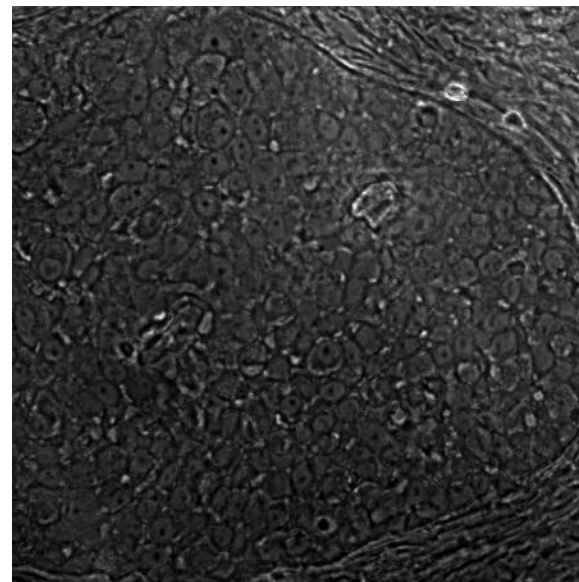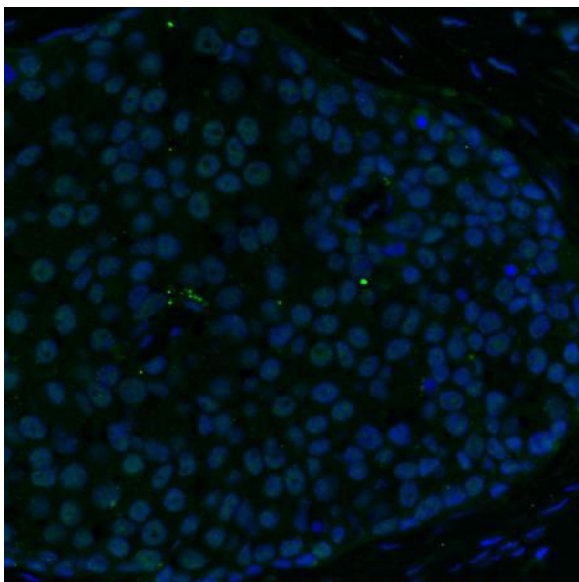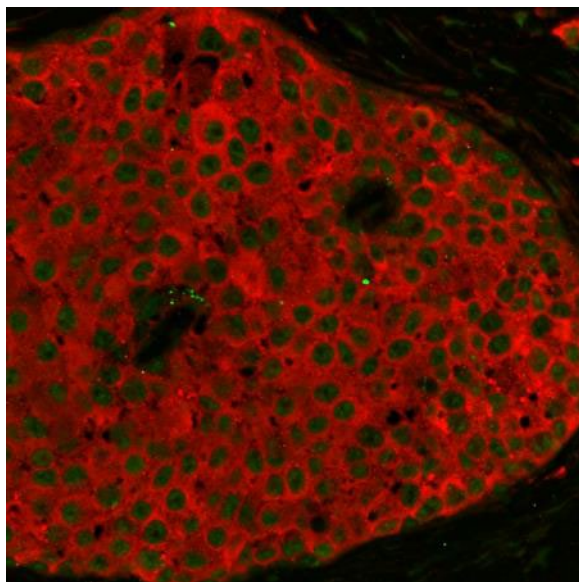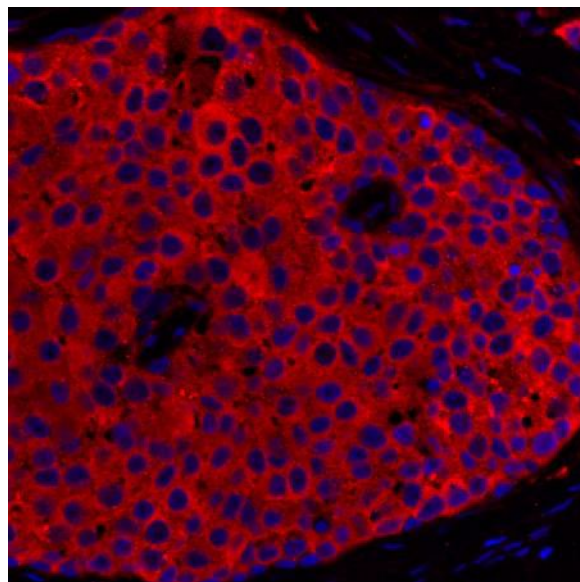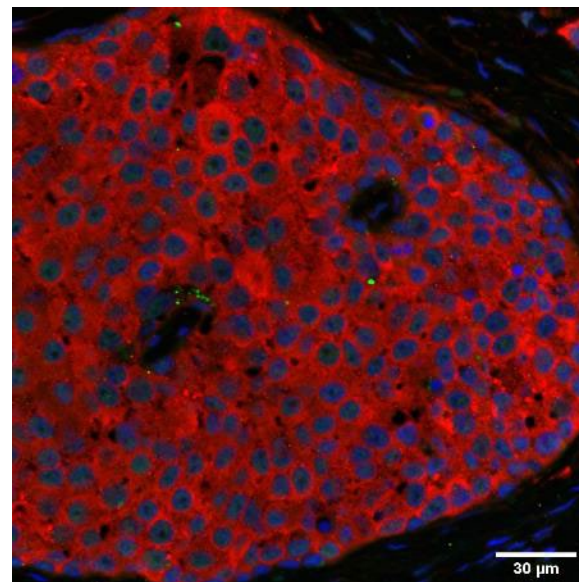

17490\_00

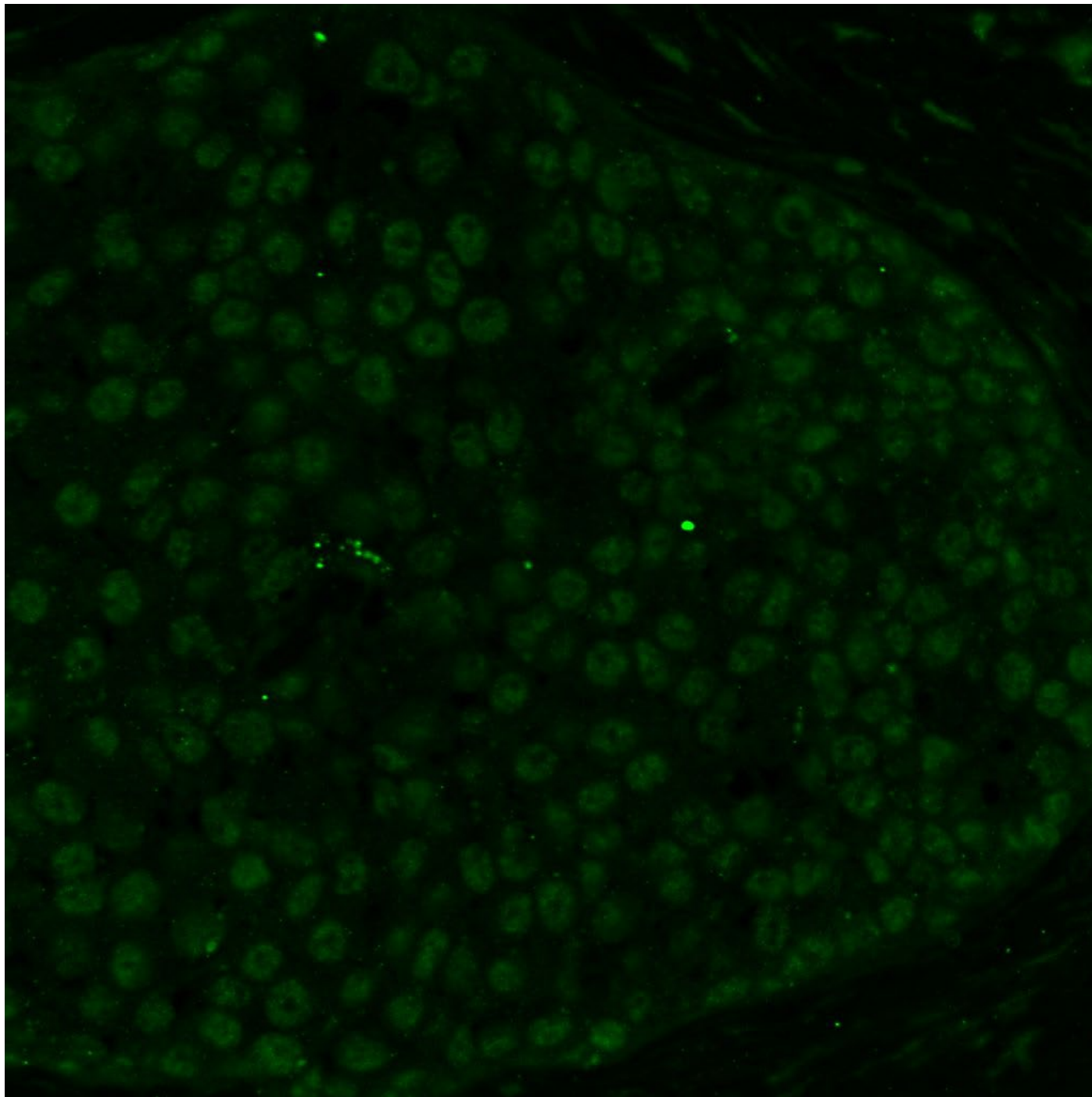

17490\_00

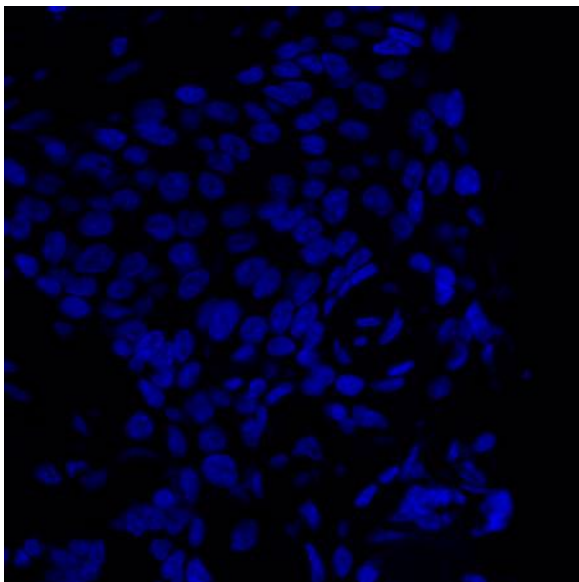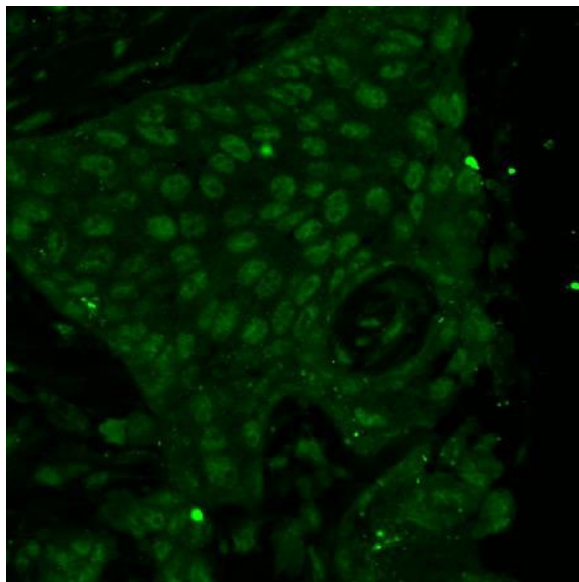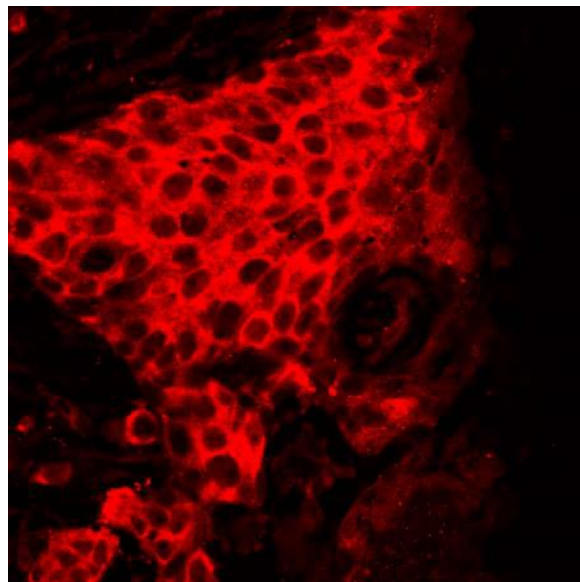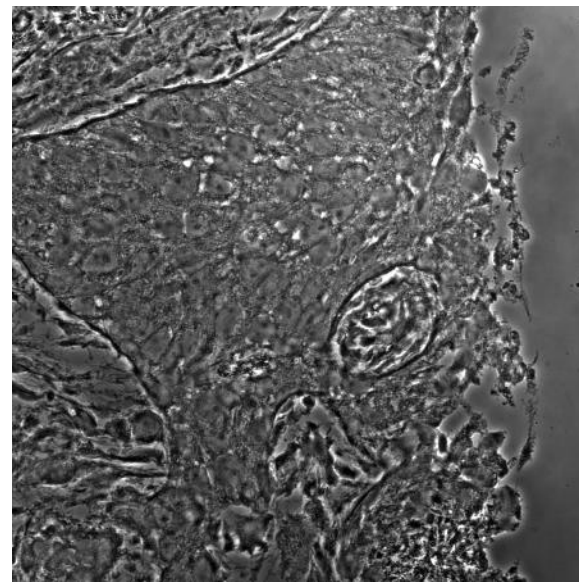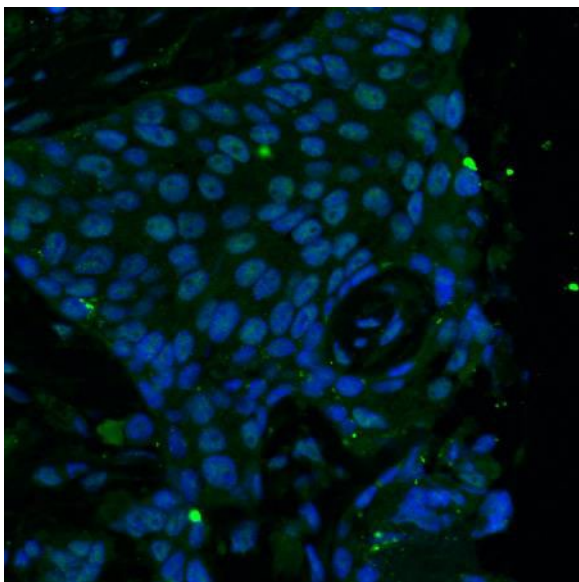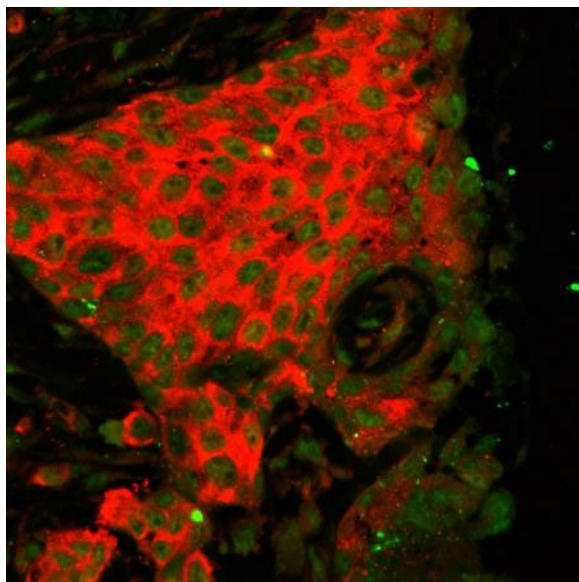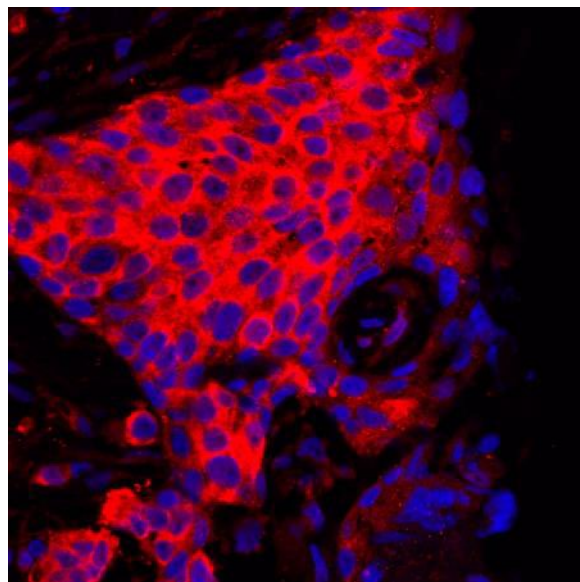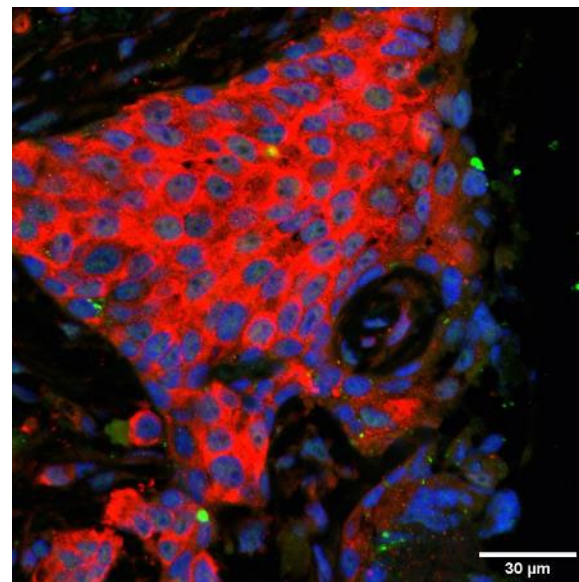

17490\_01

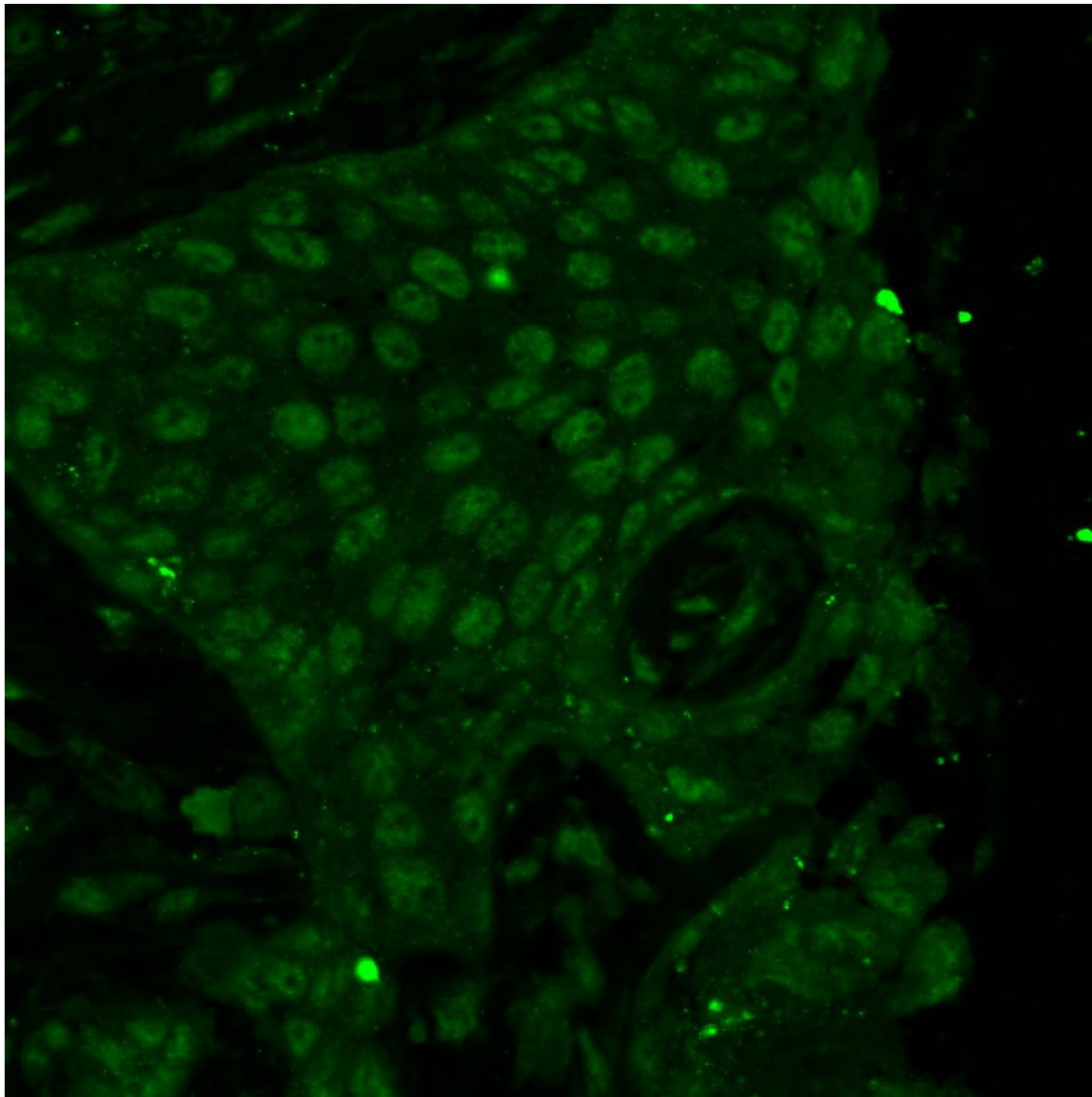

17490\_01

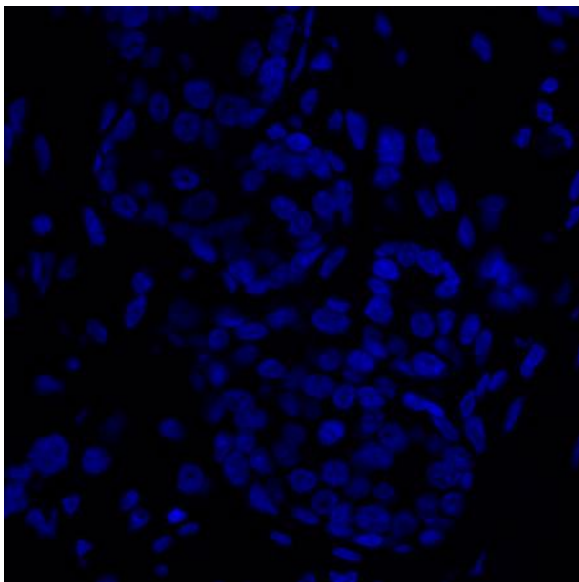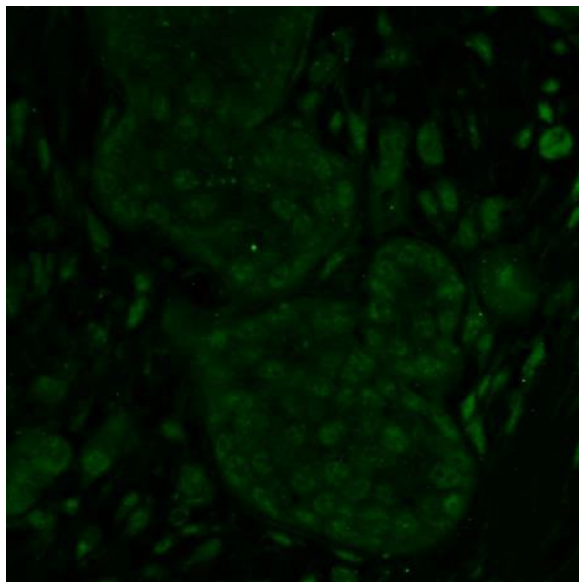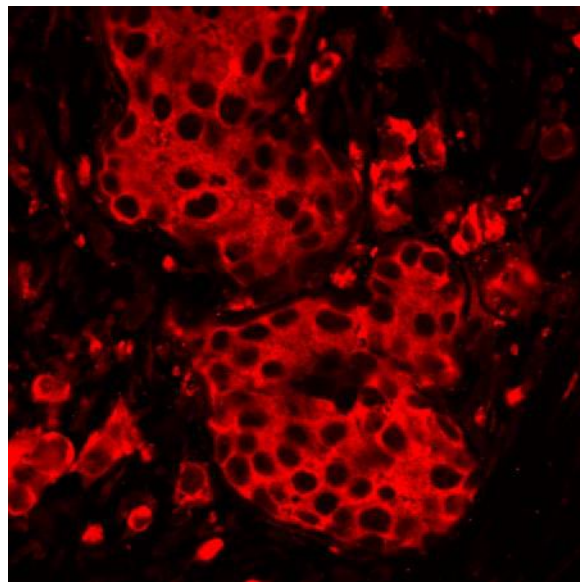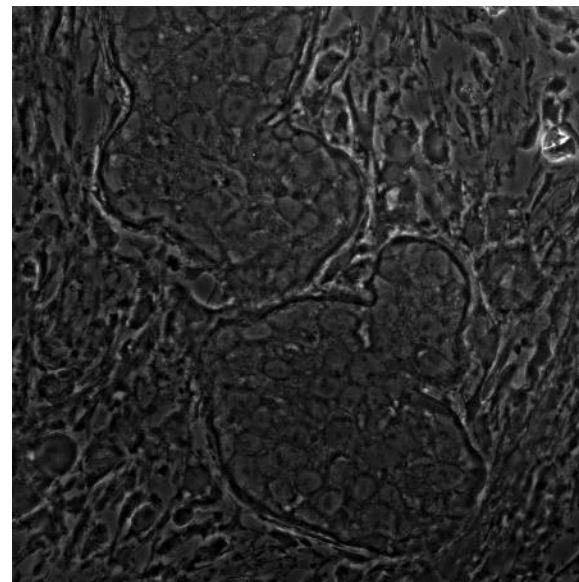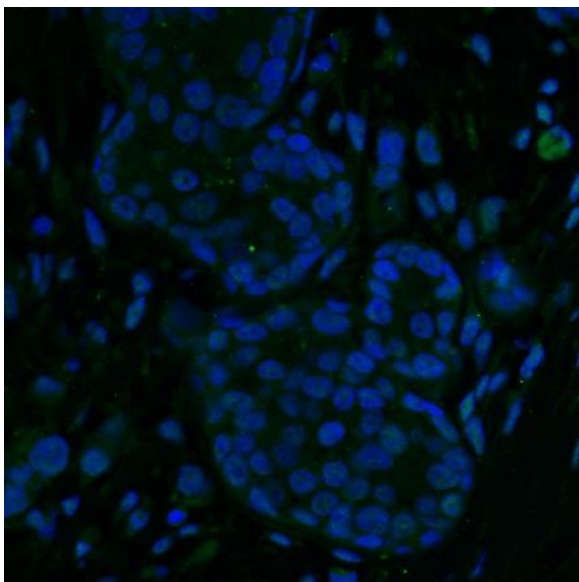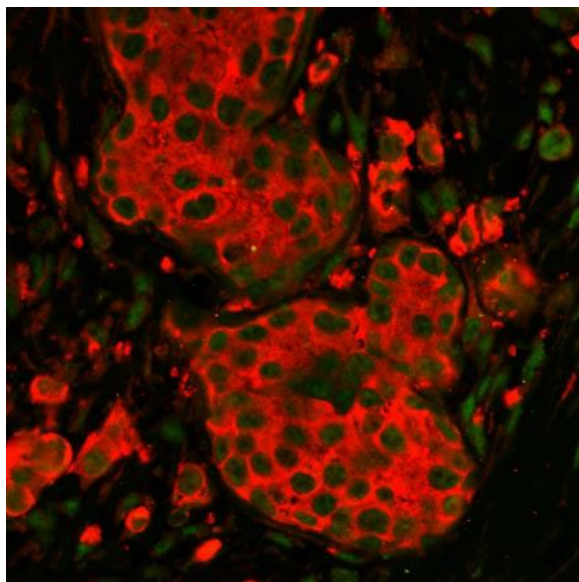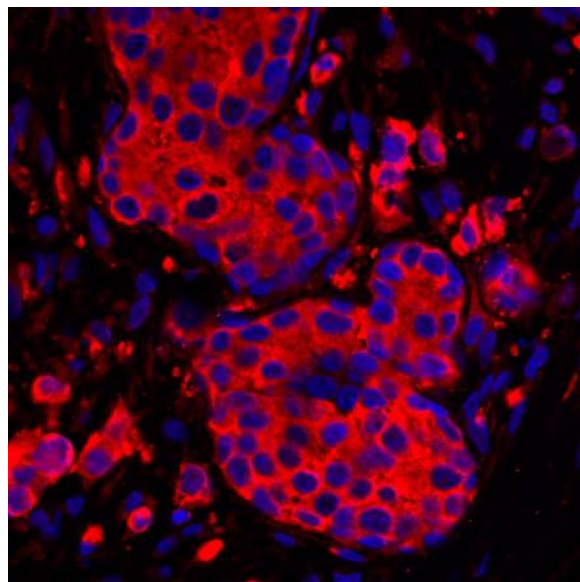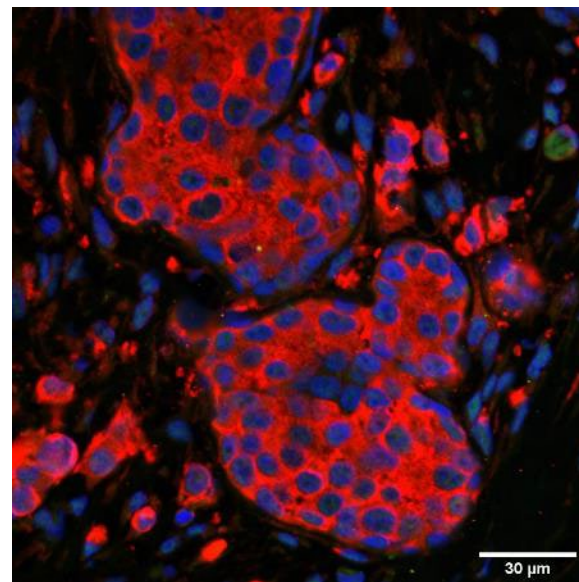

17490\_02

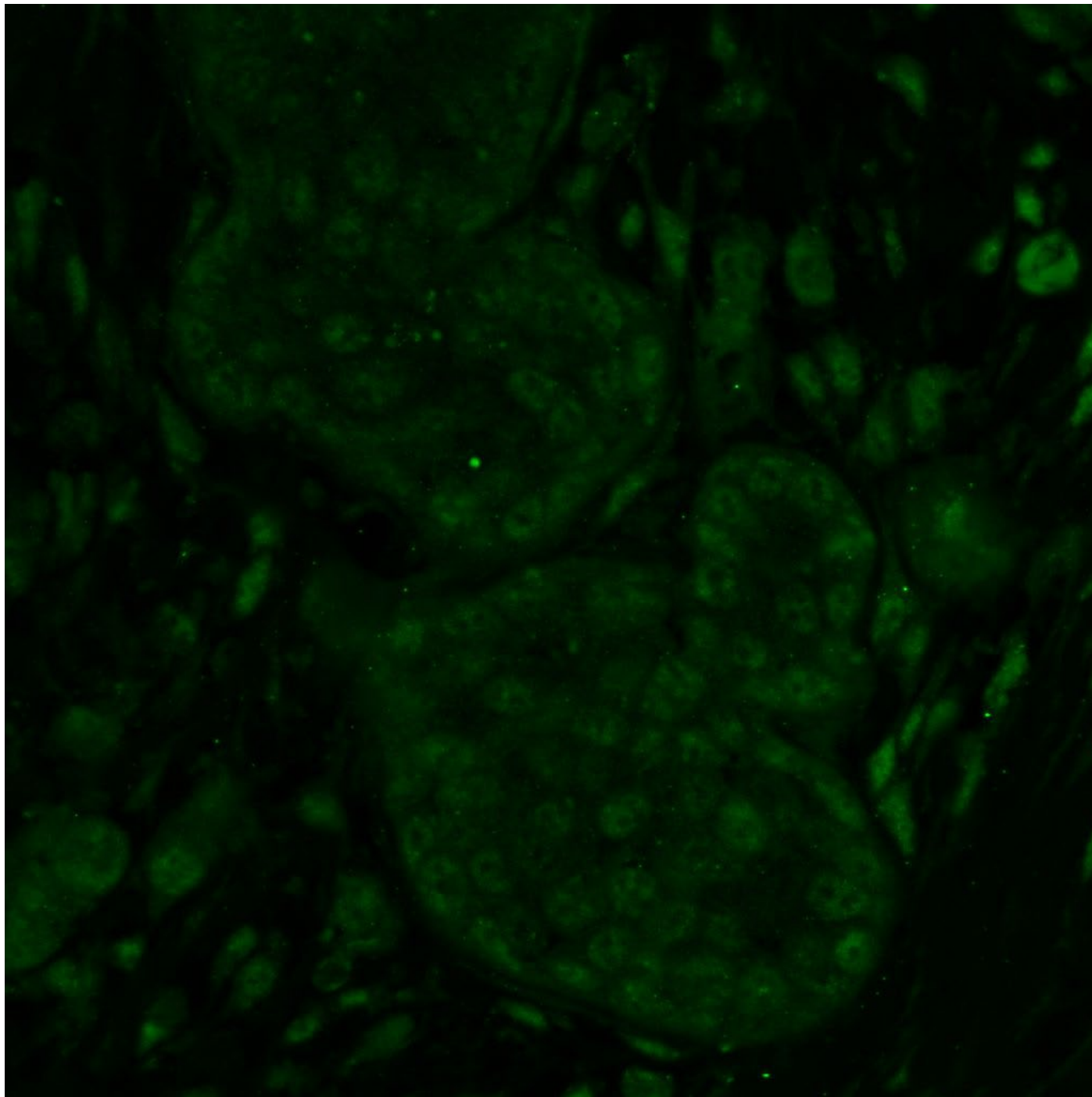

17490\_02

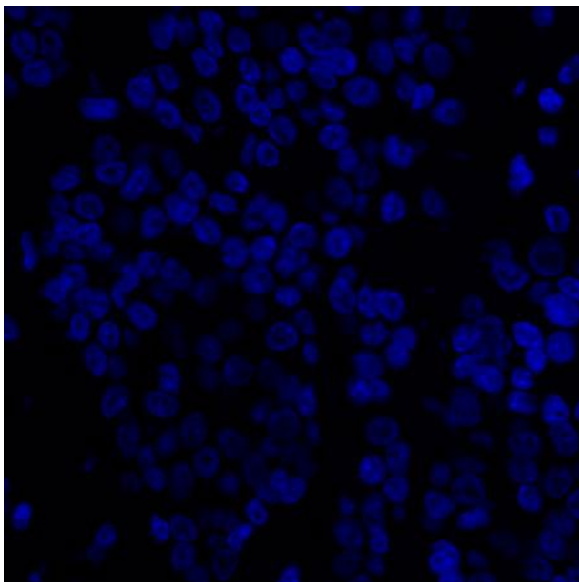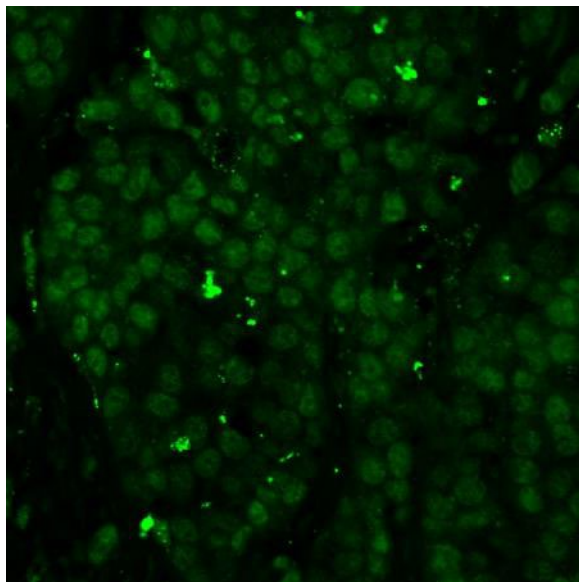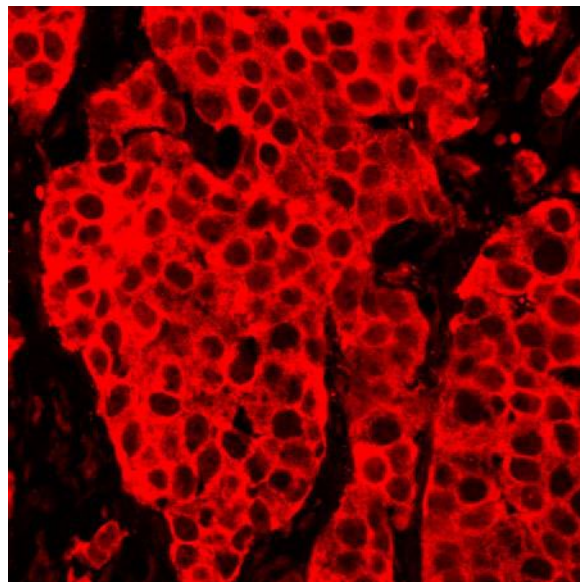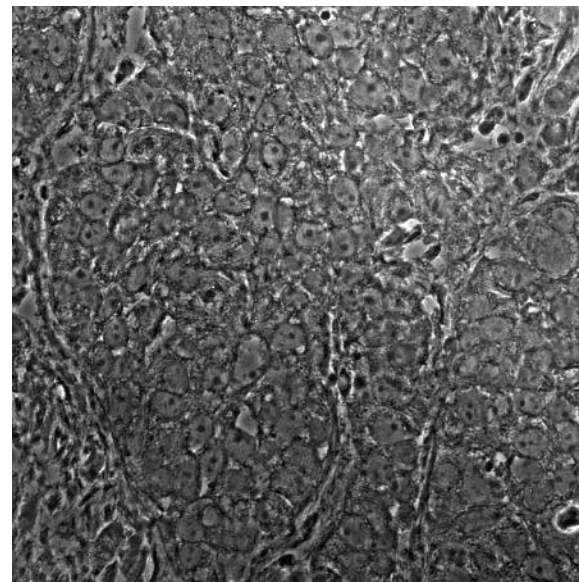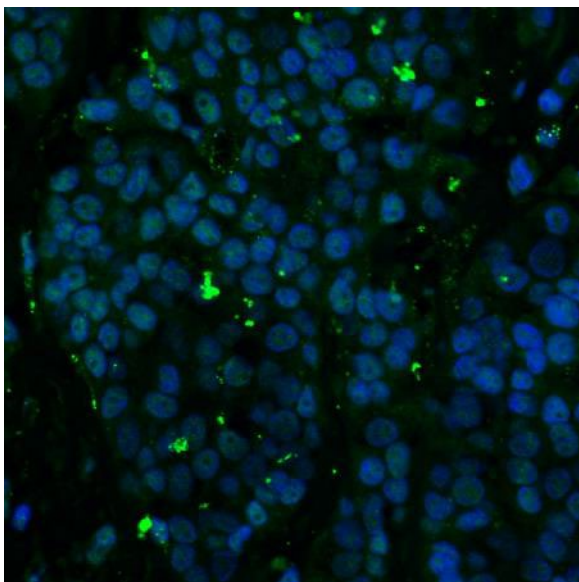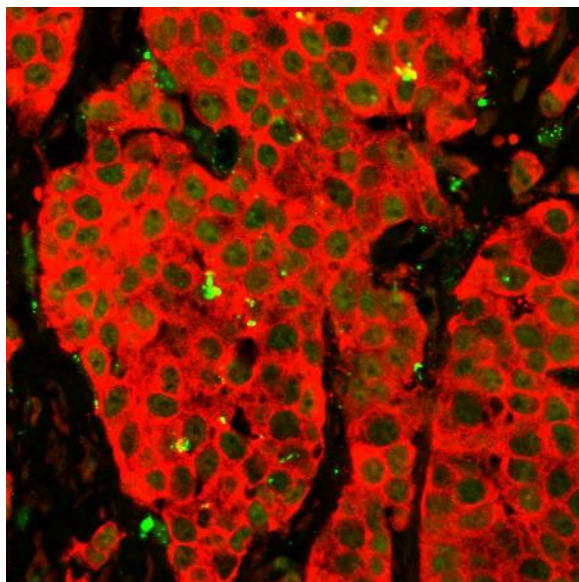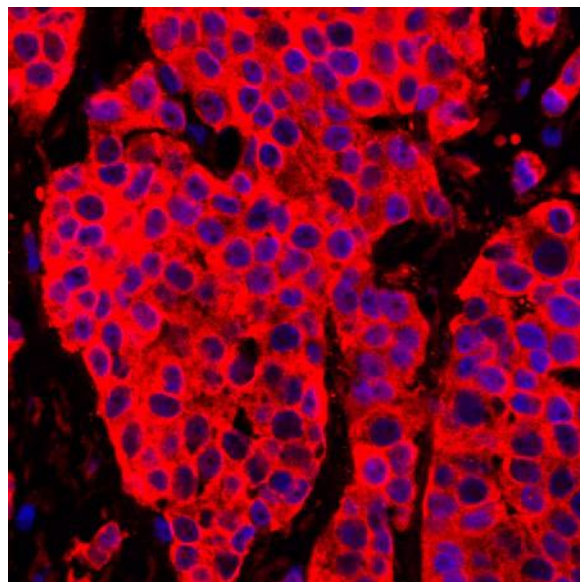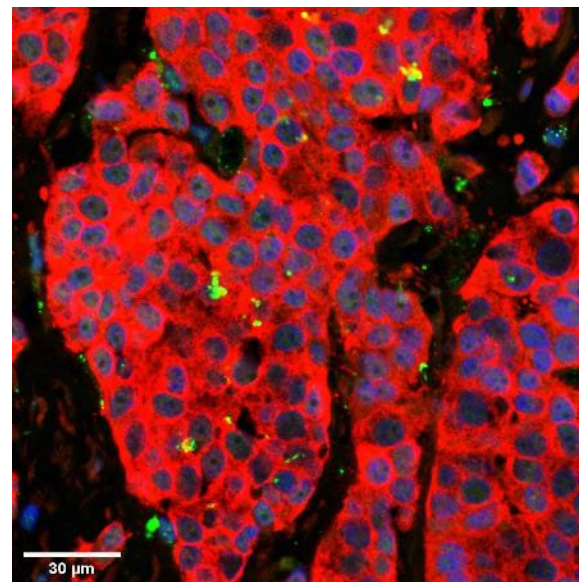

17490\_03

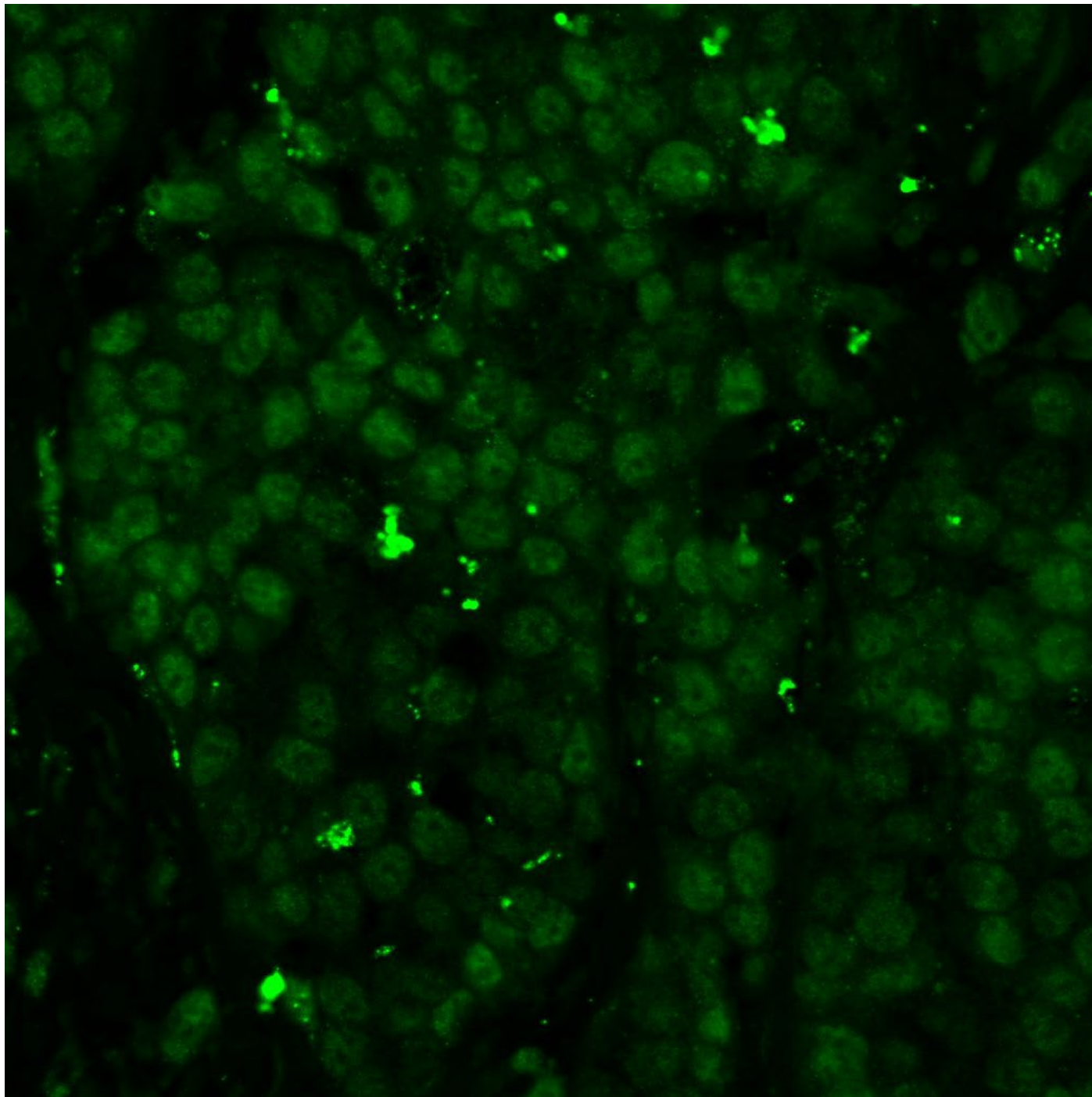

17490\_03

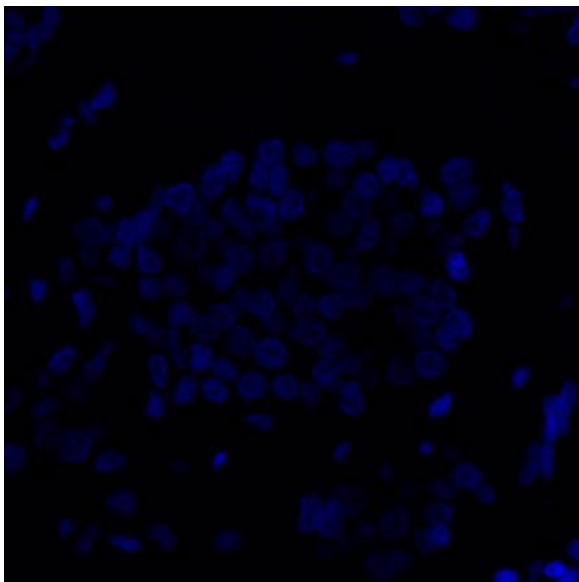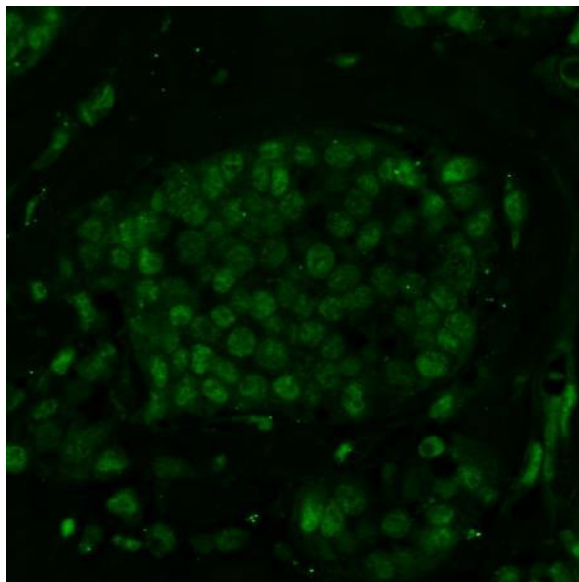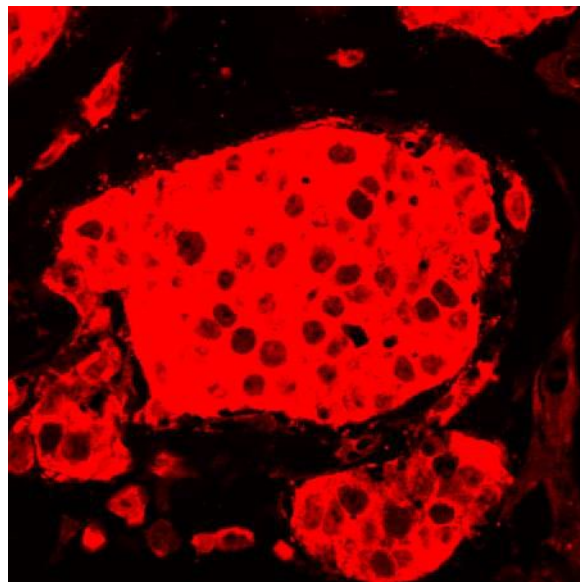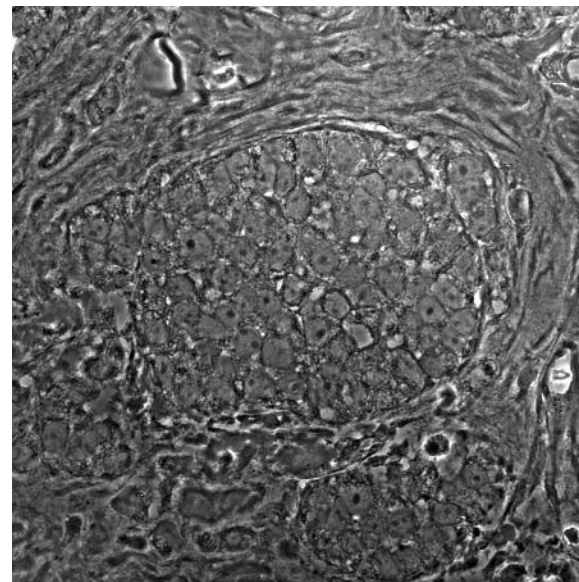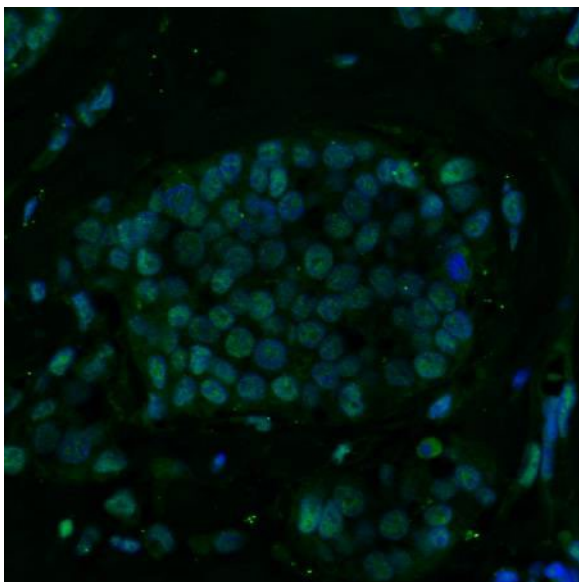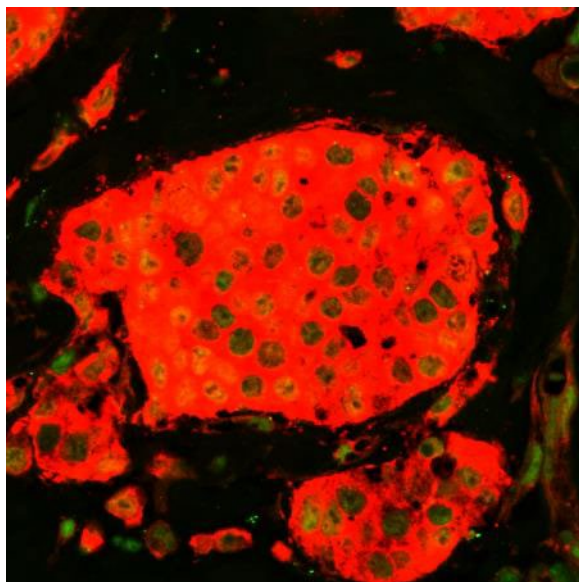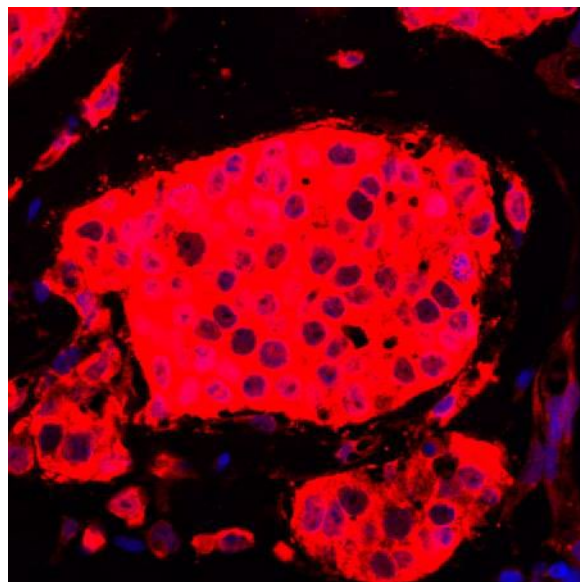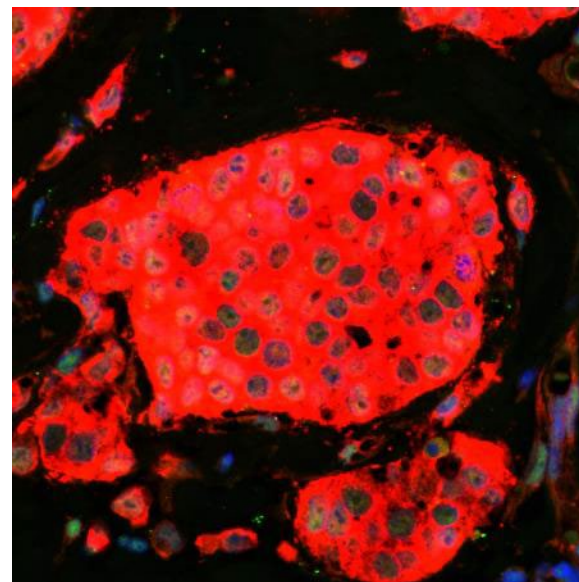

17490\_04

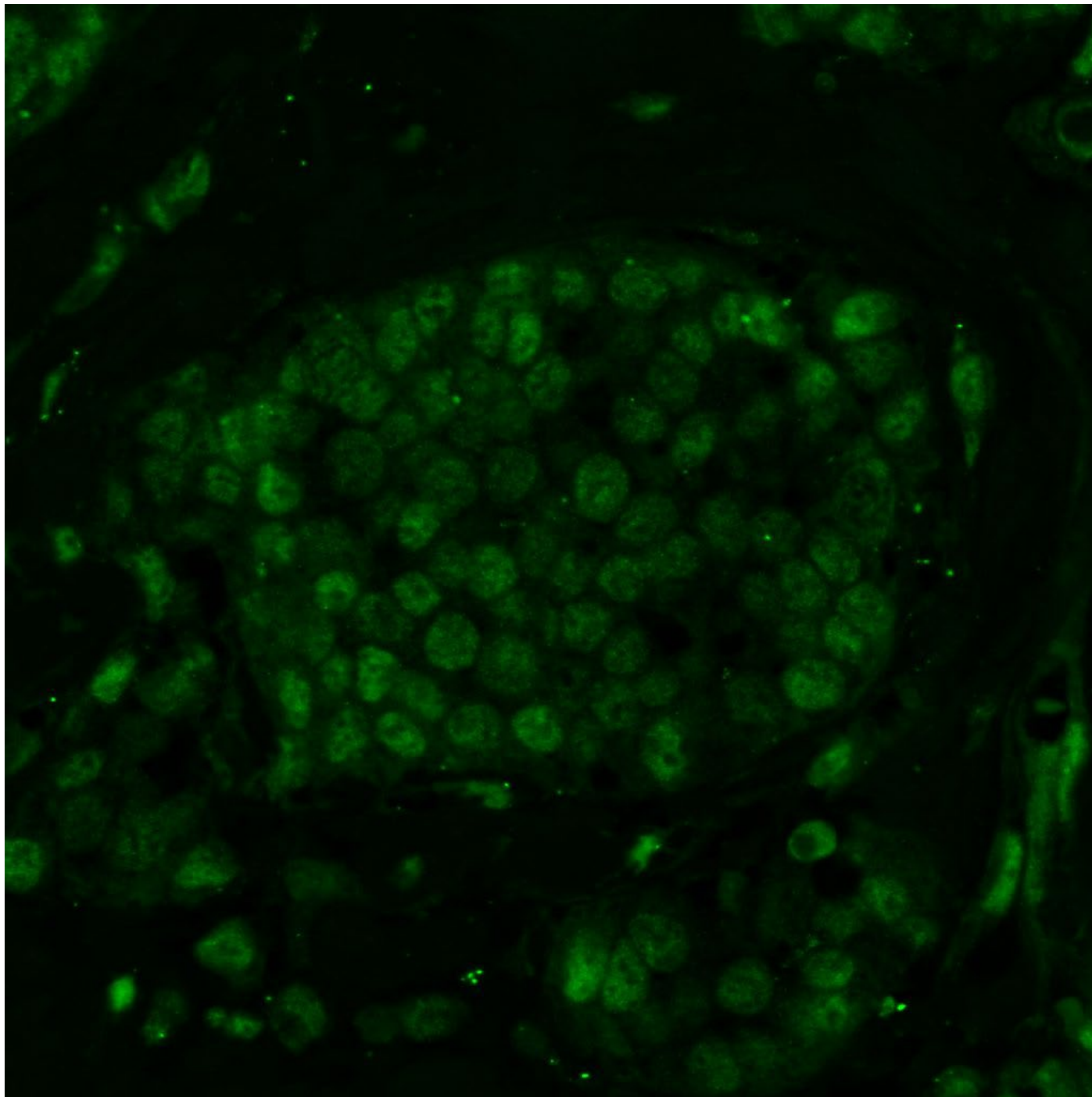

17490\_04

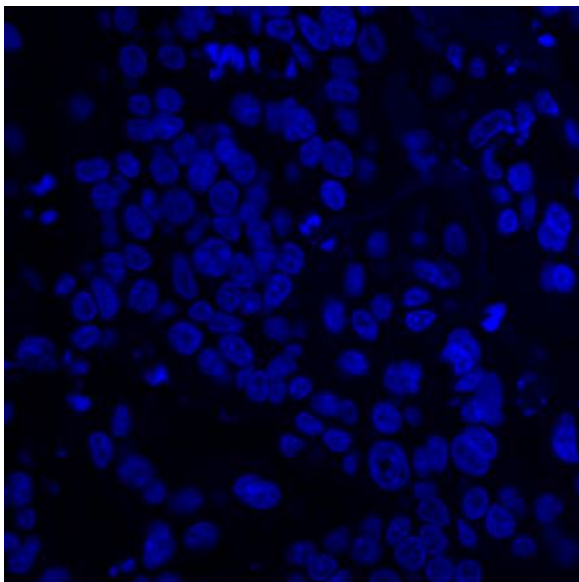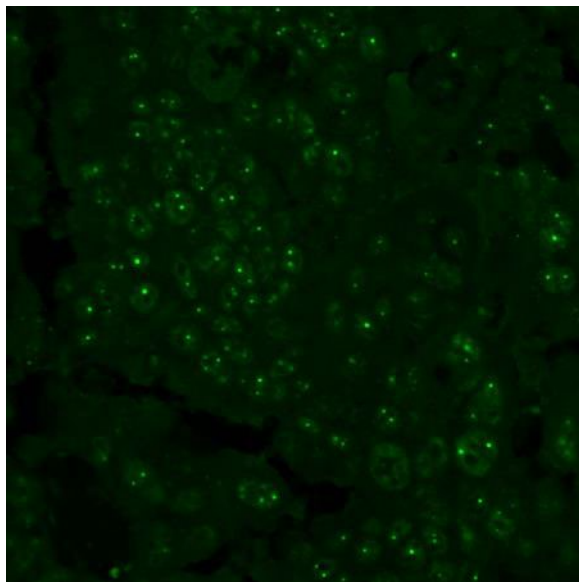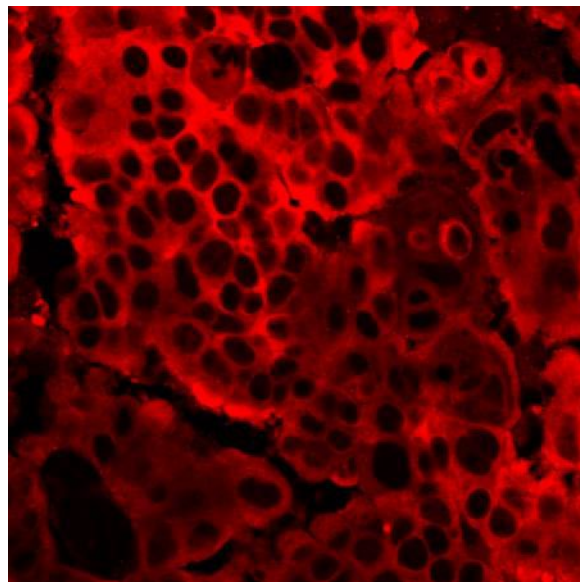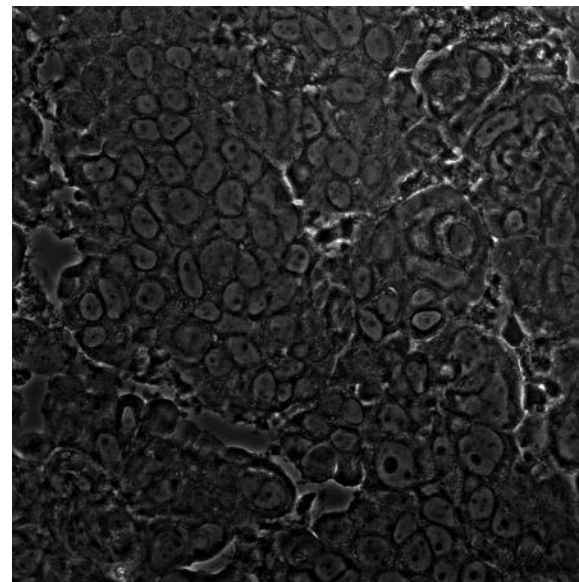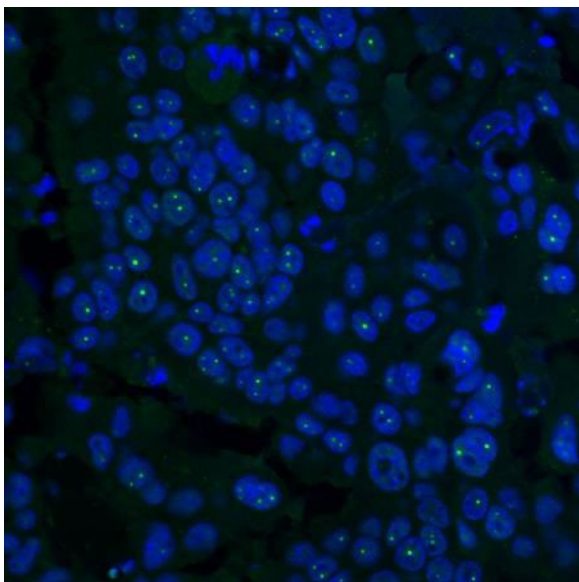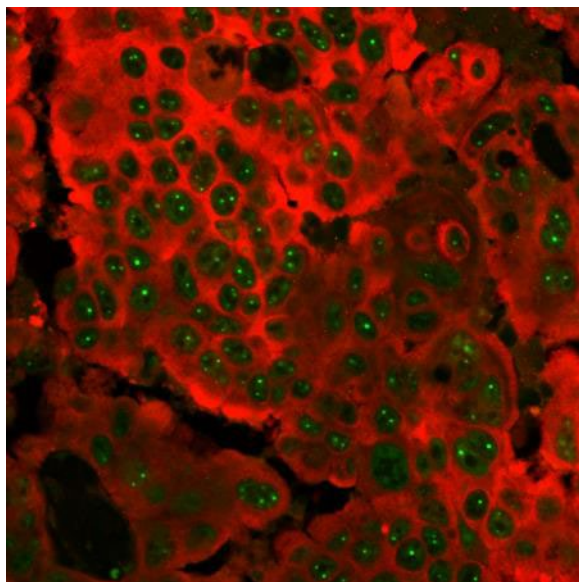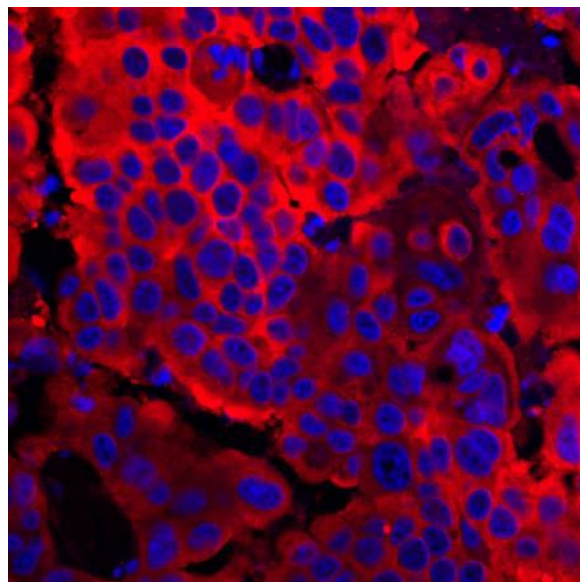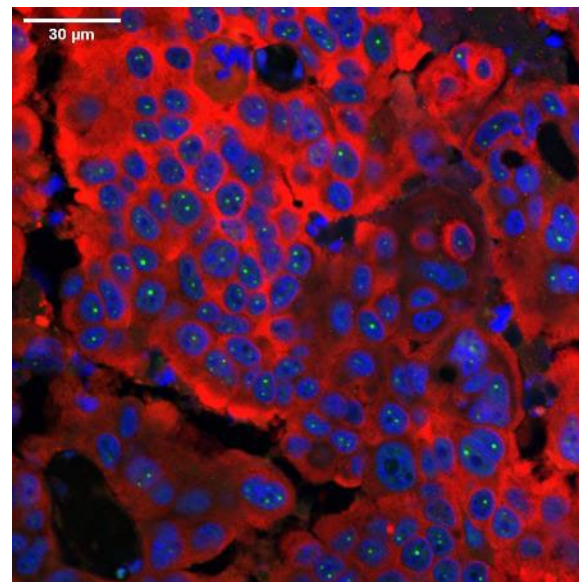

18440\_00

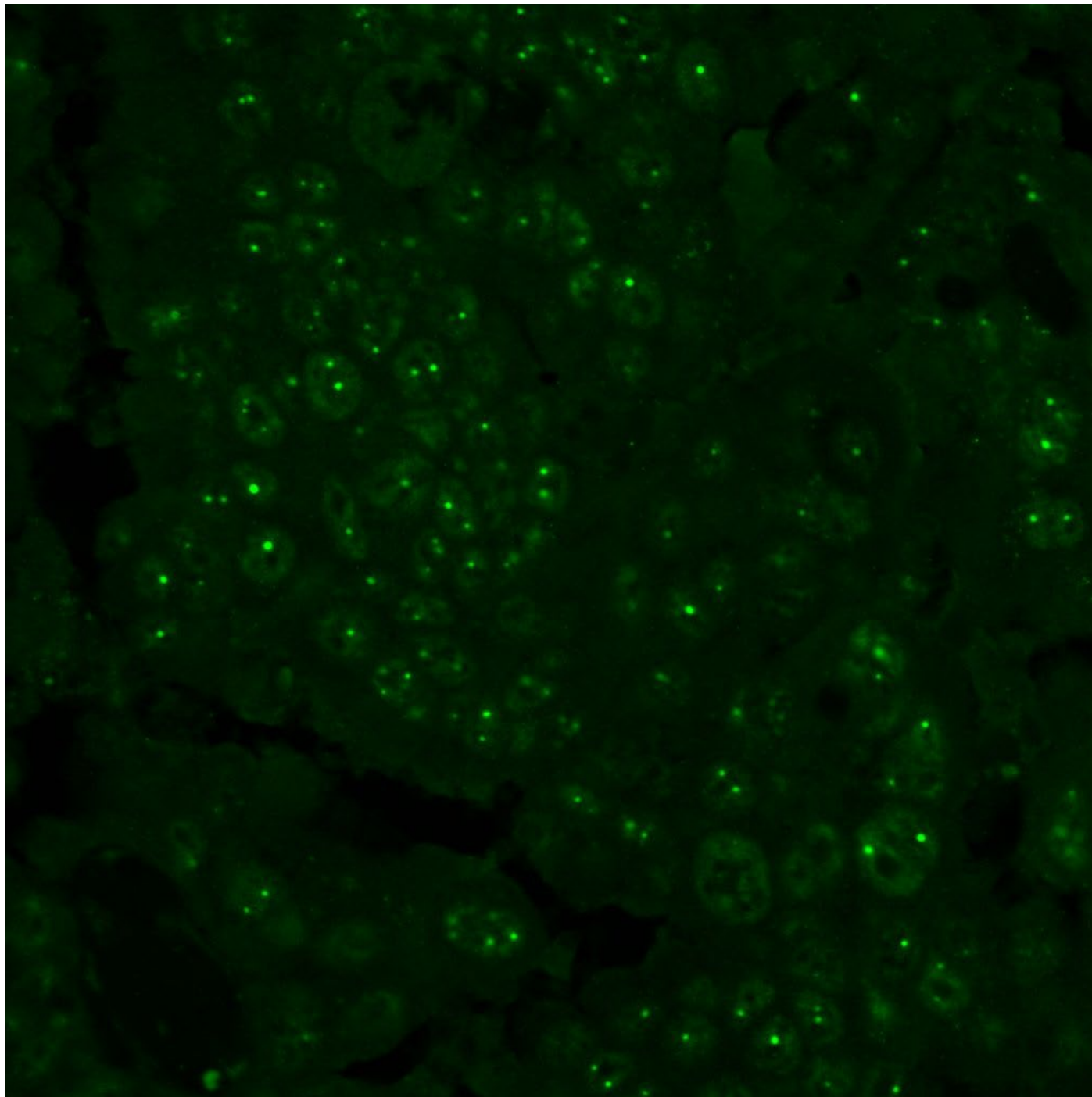

18440\_00

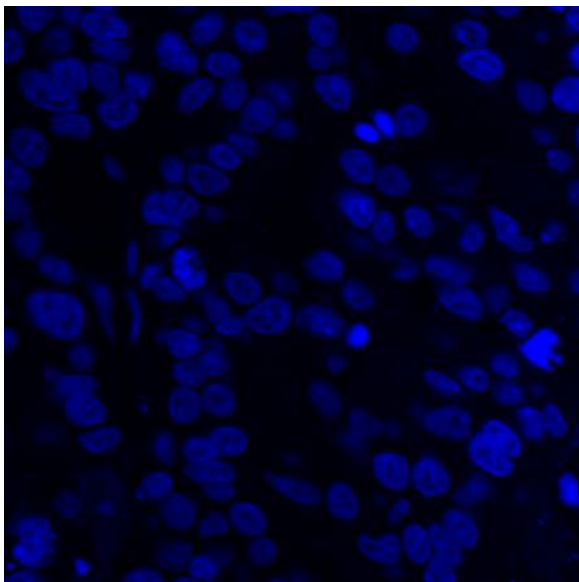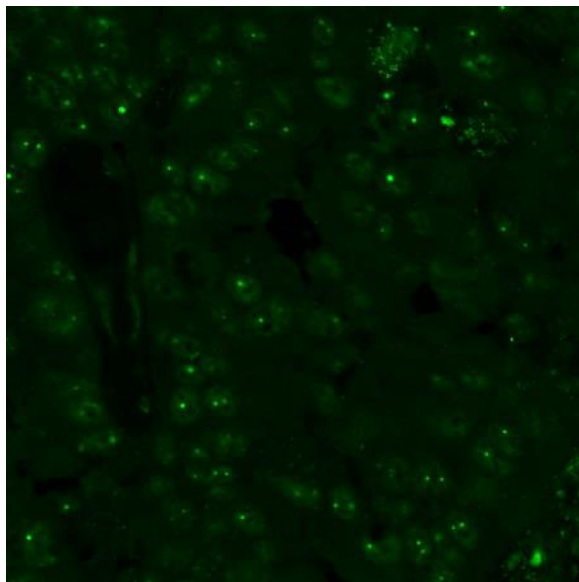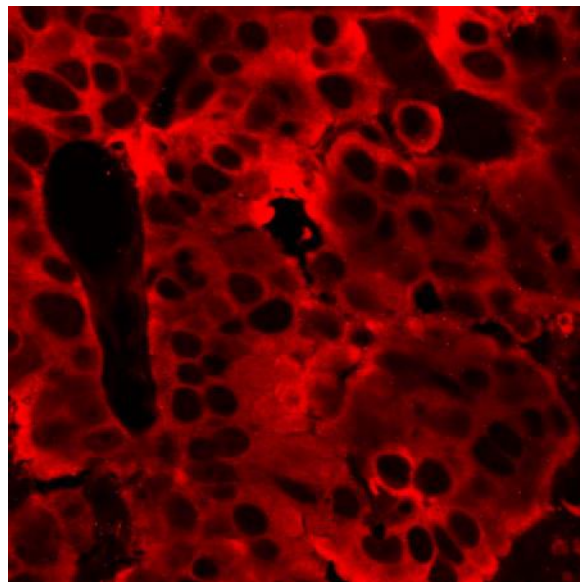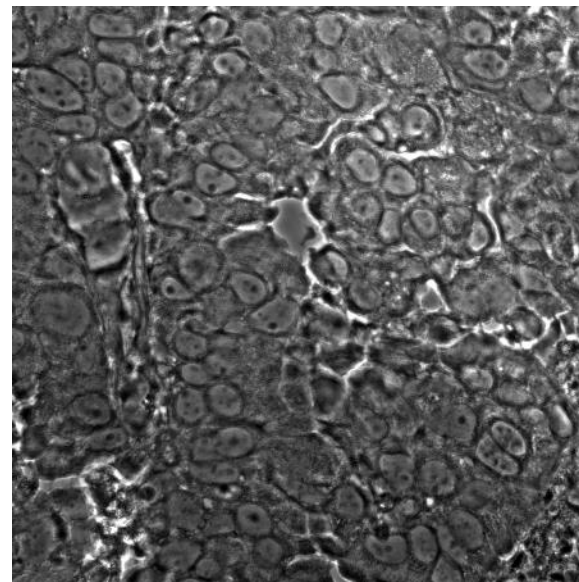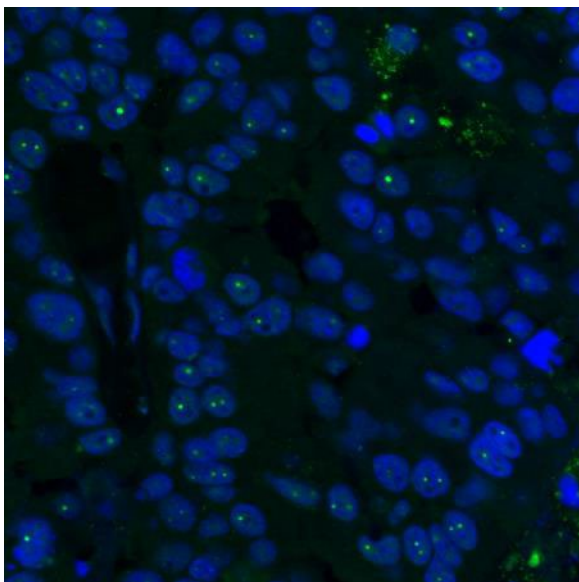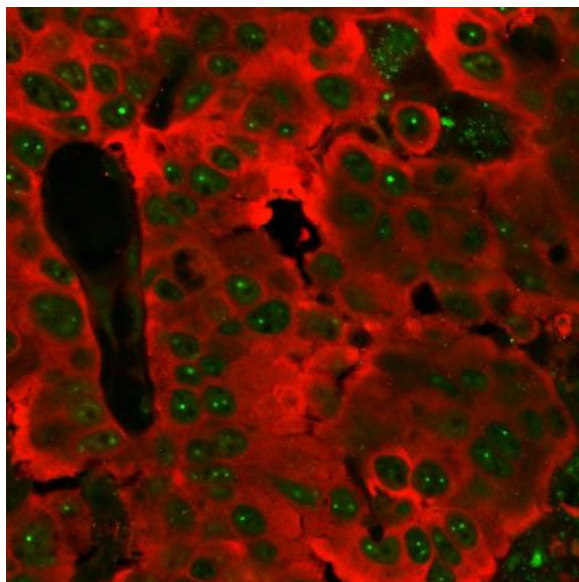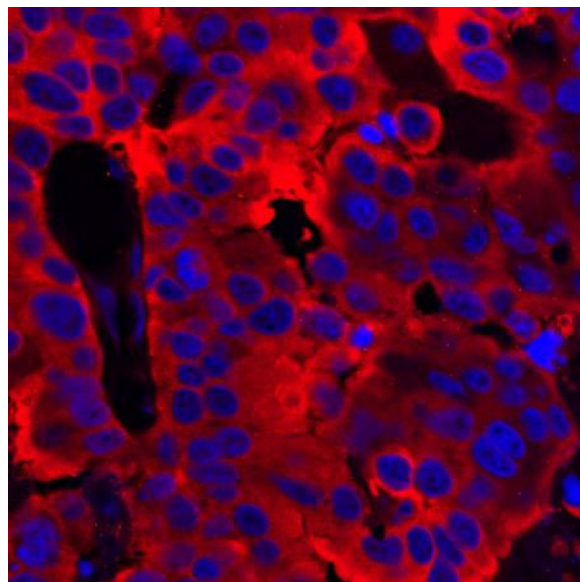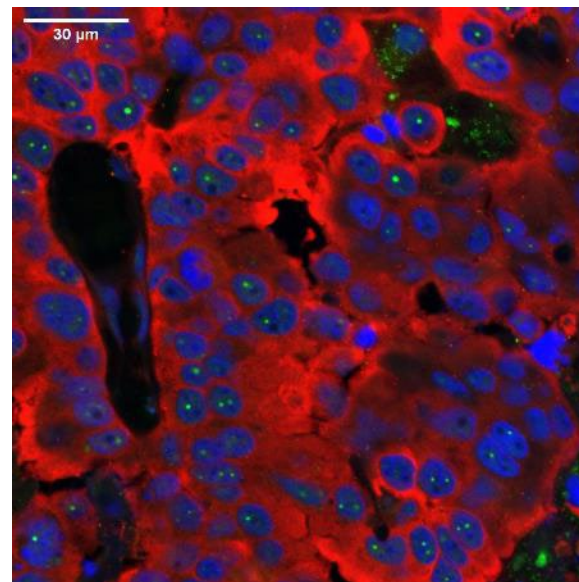

18440\_01

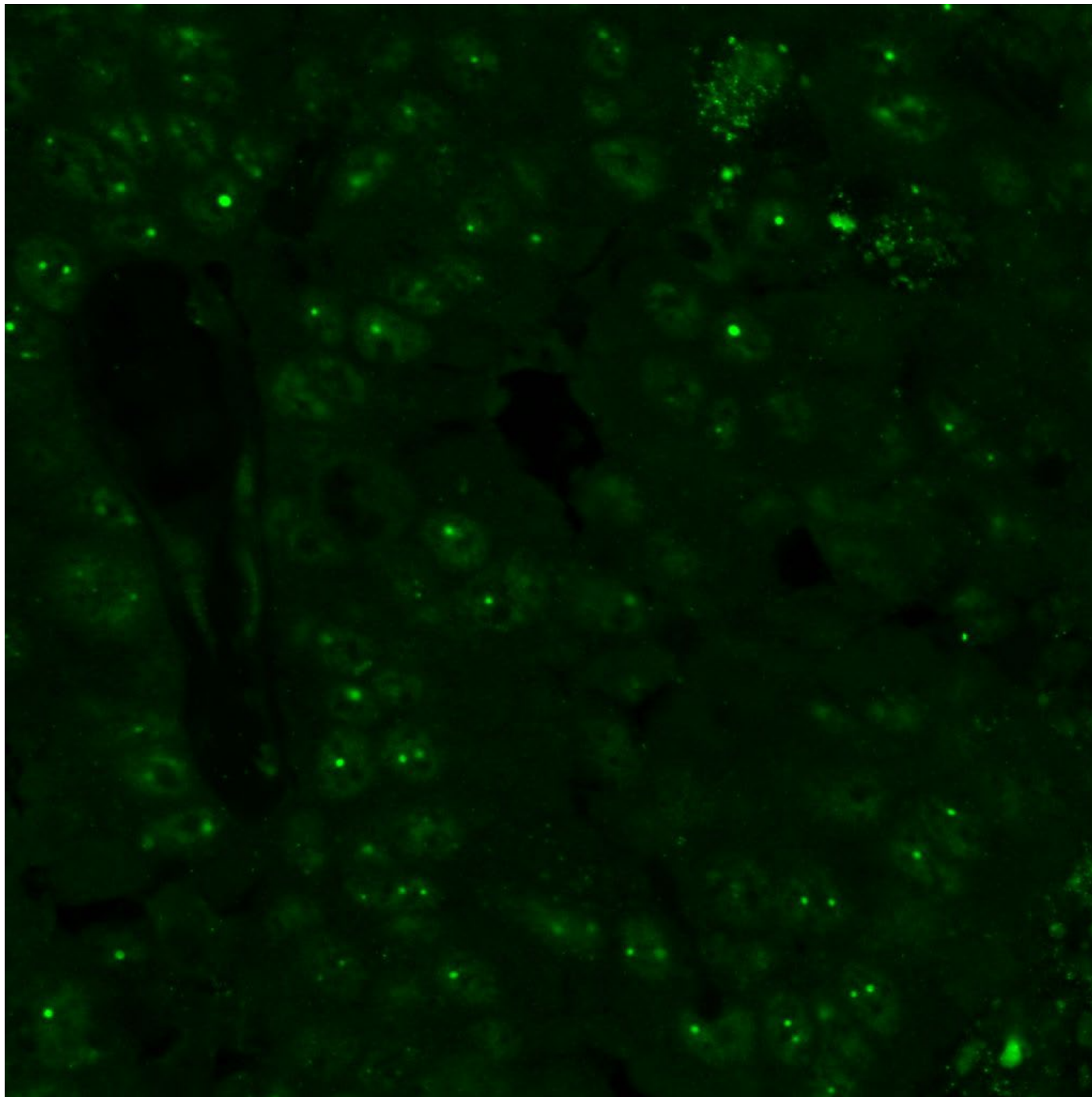

18440\_01

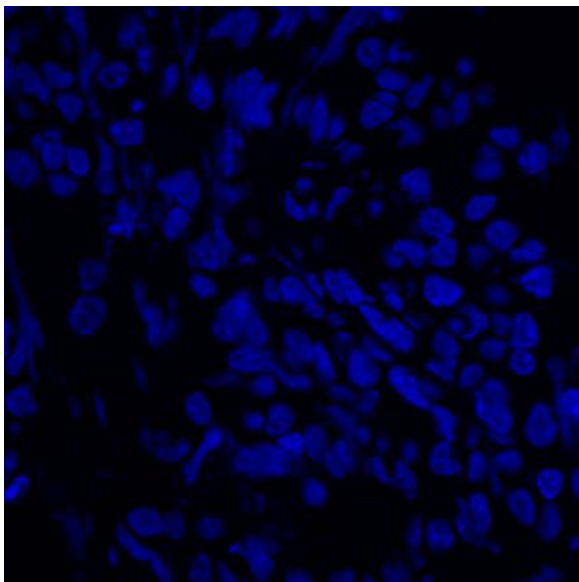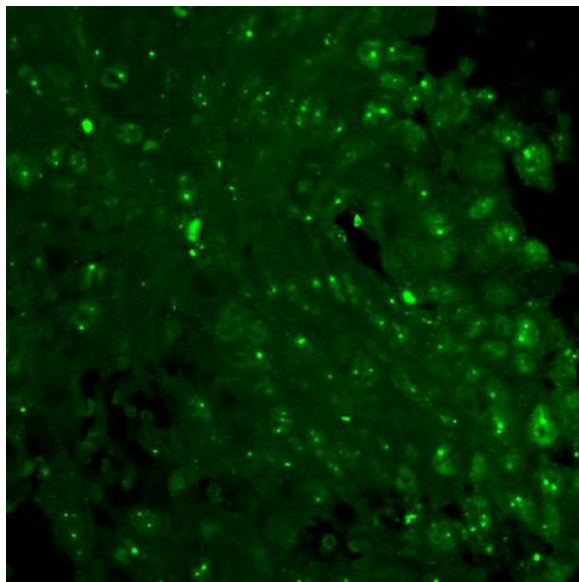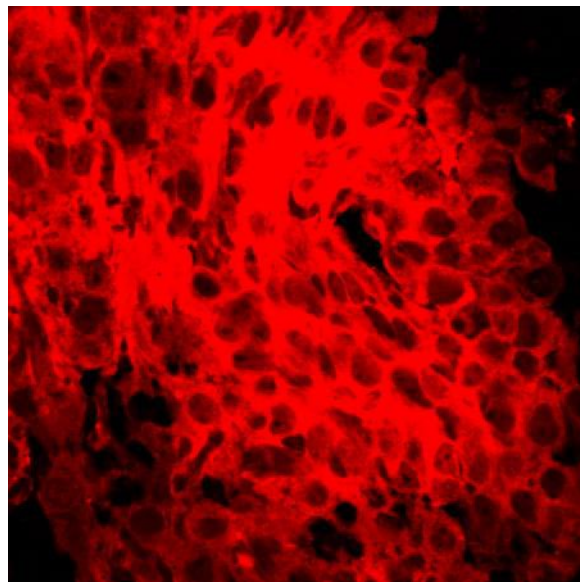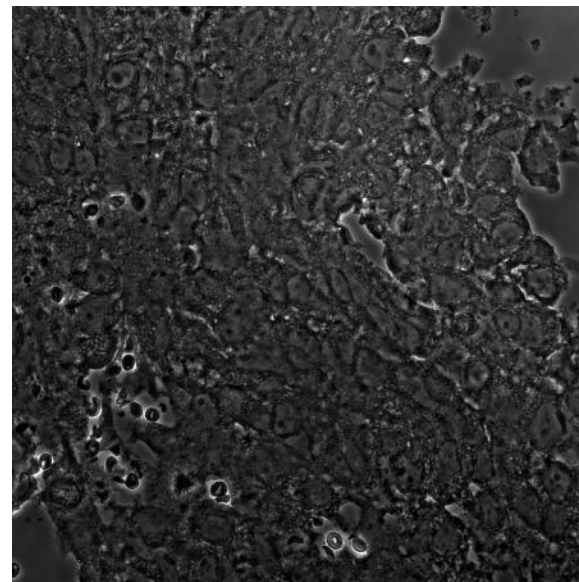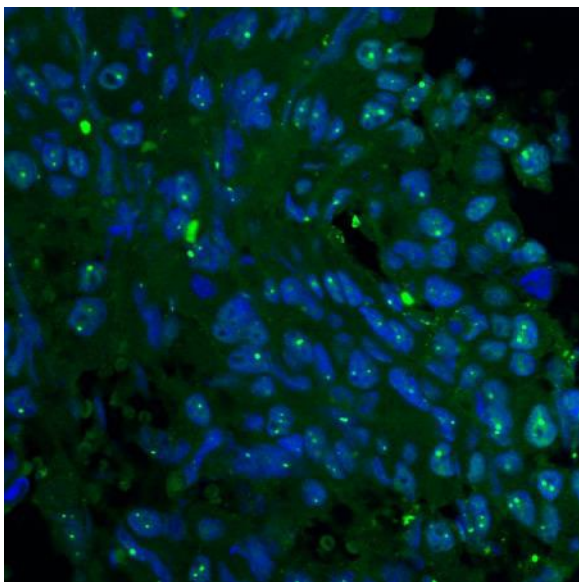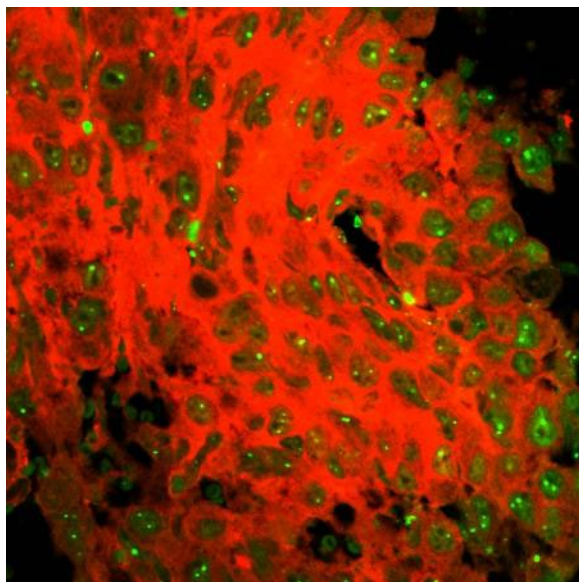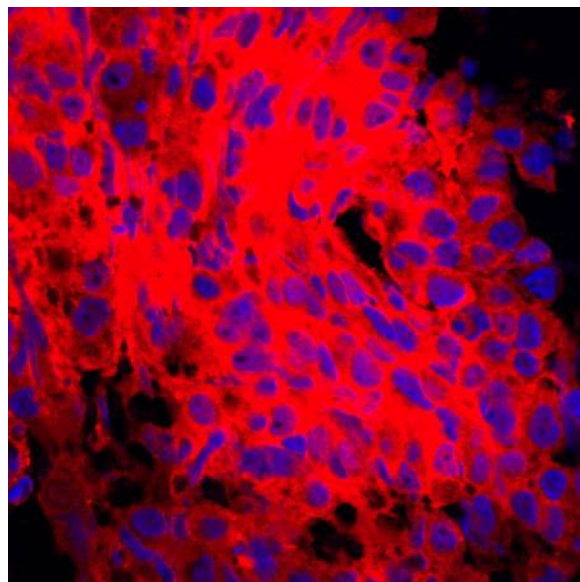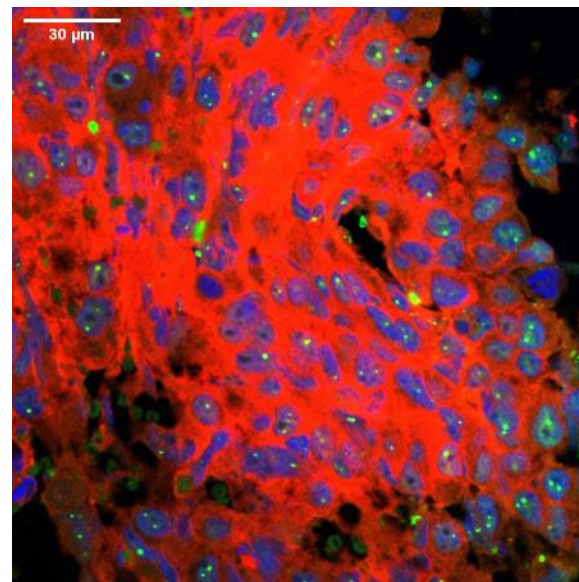

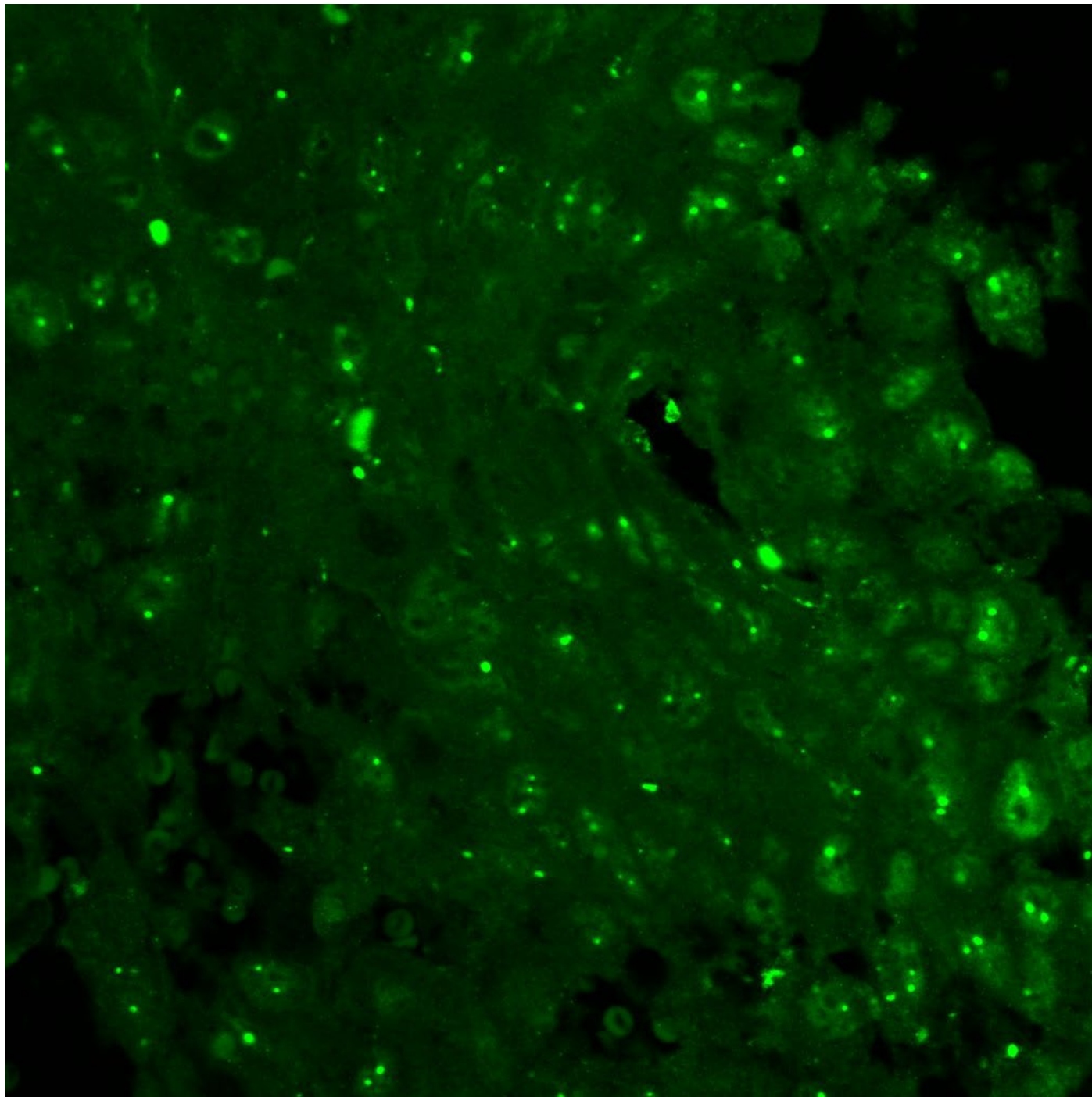

18440\_02

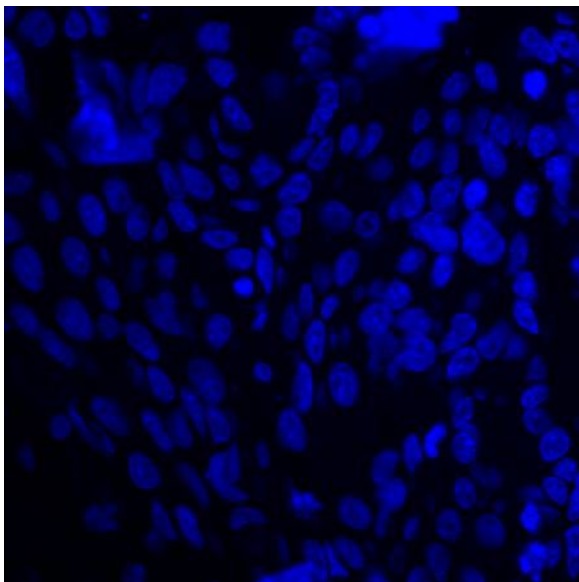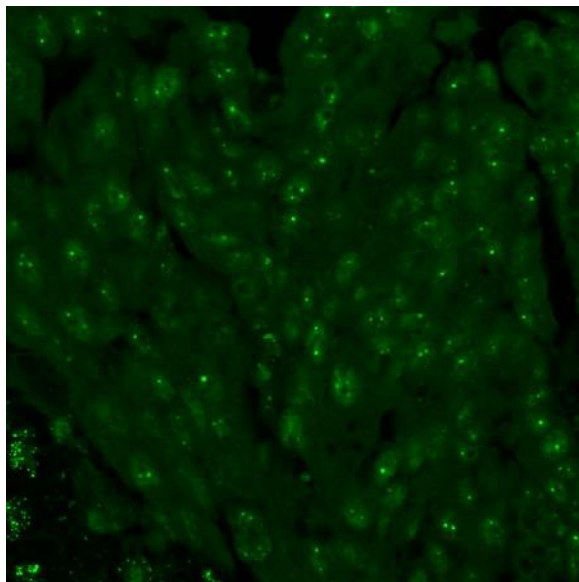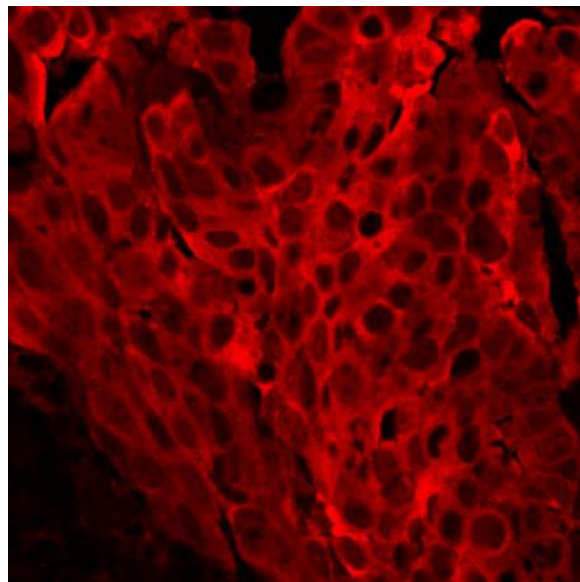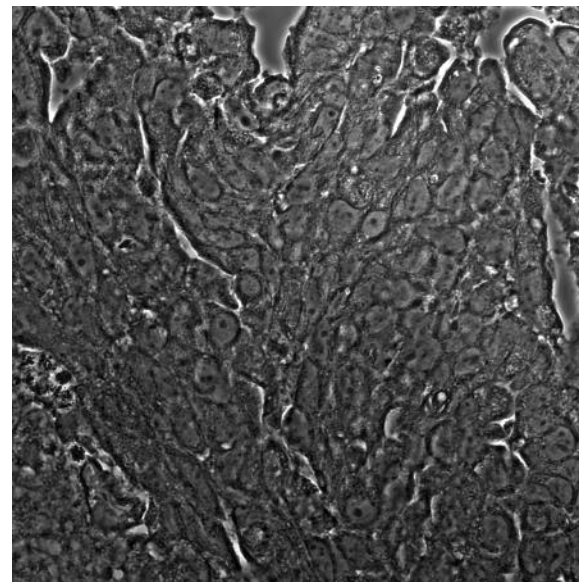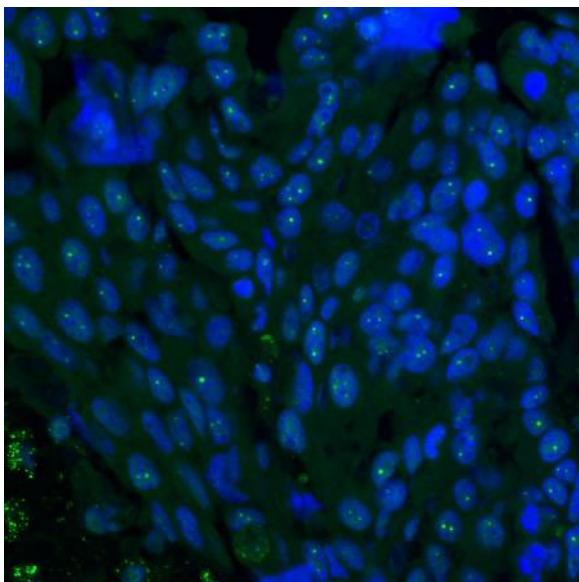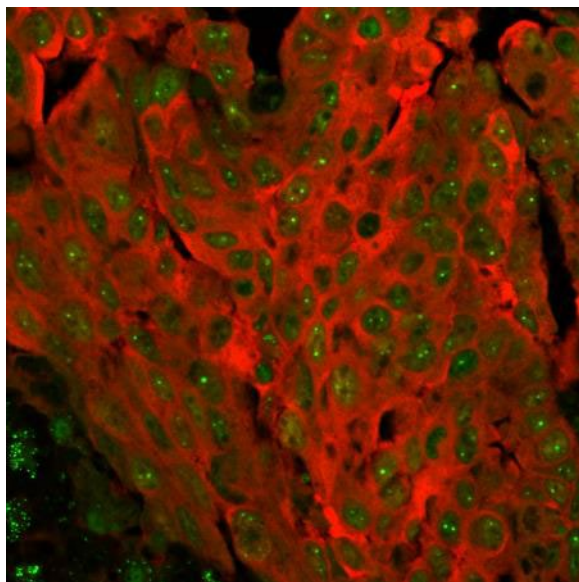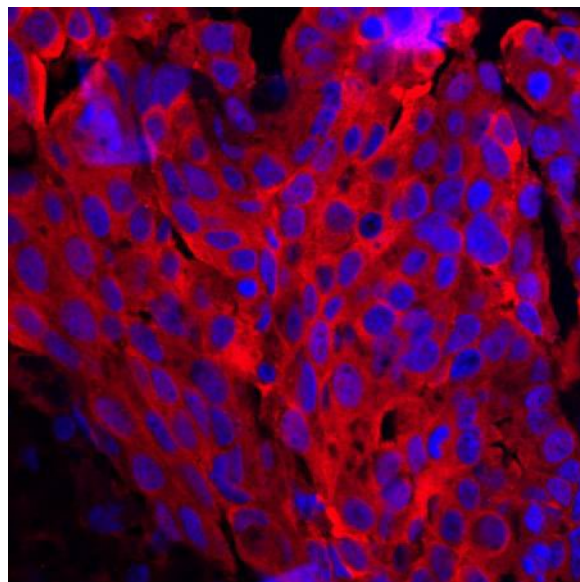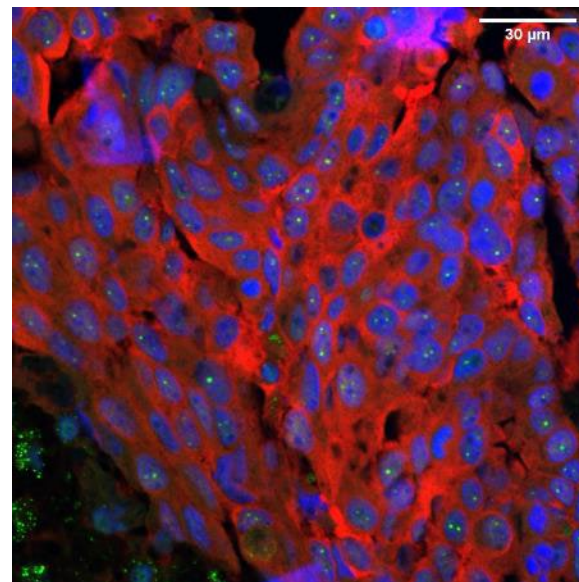

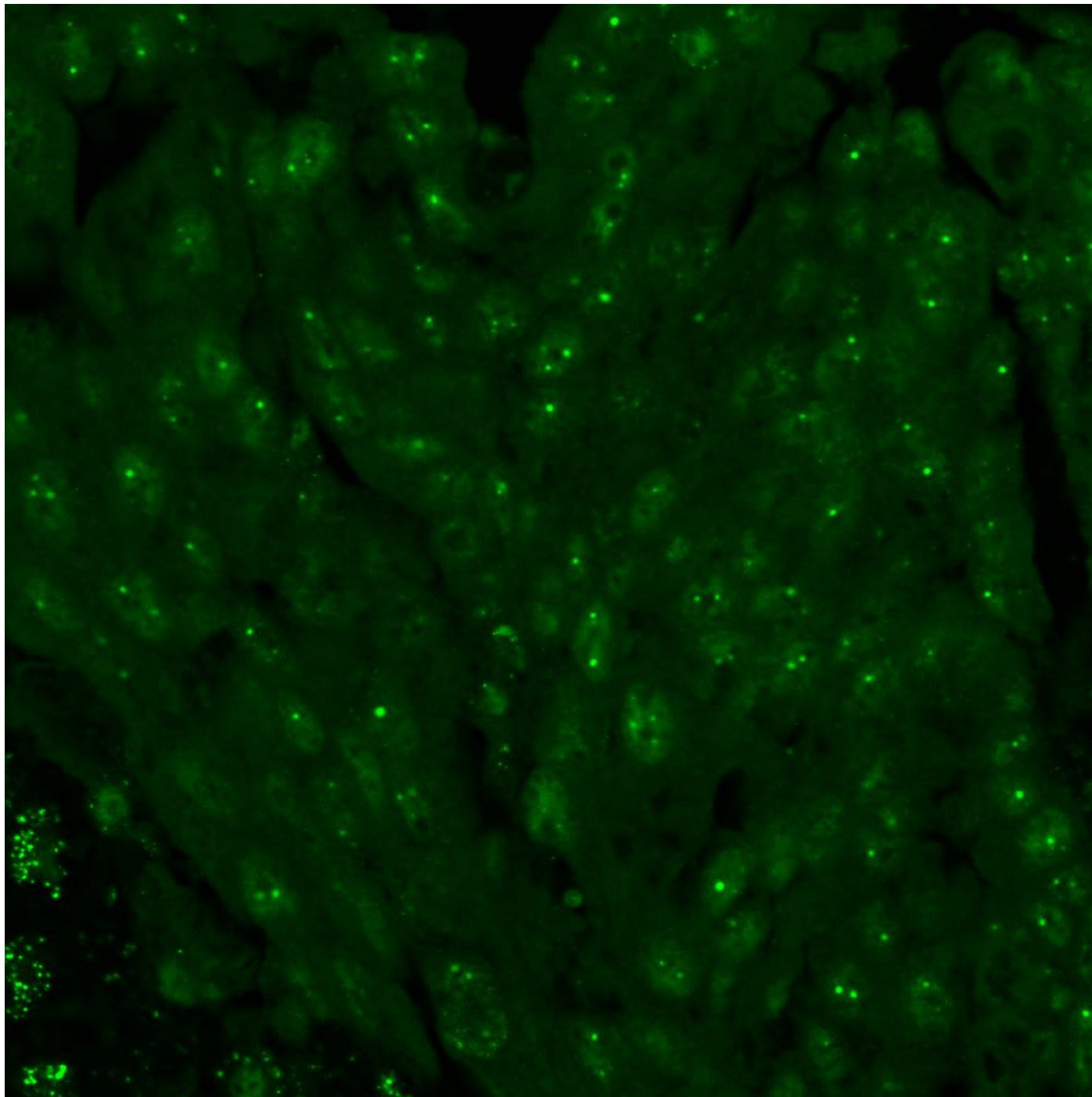

18440\_03

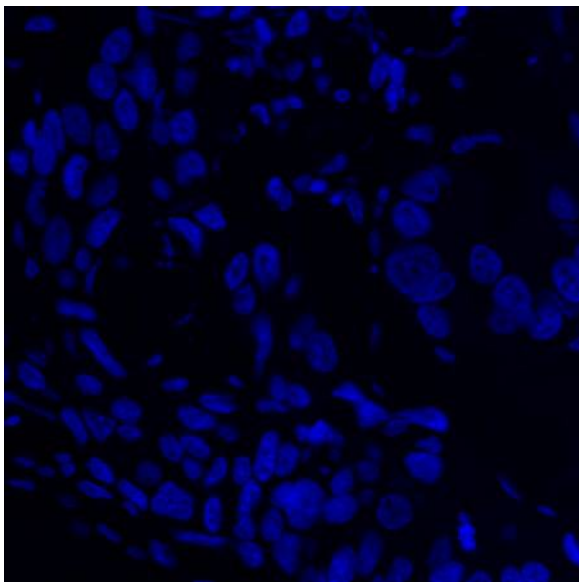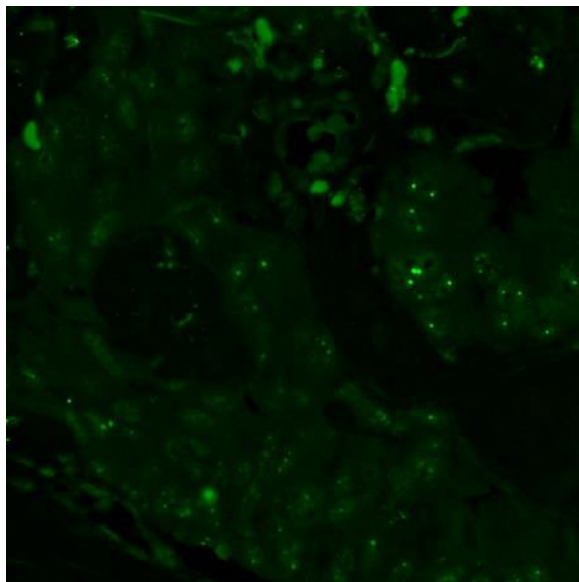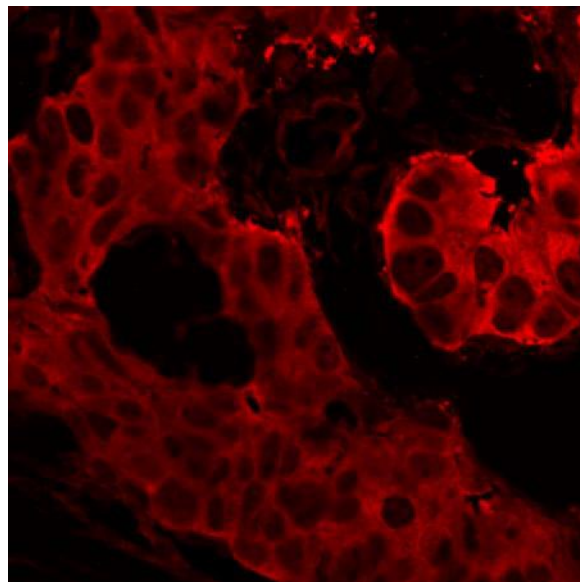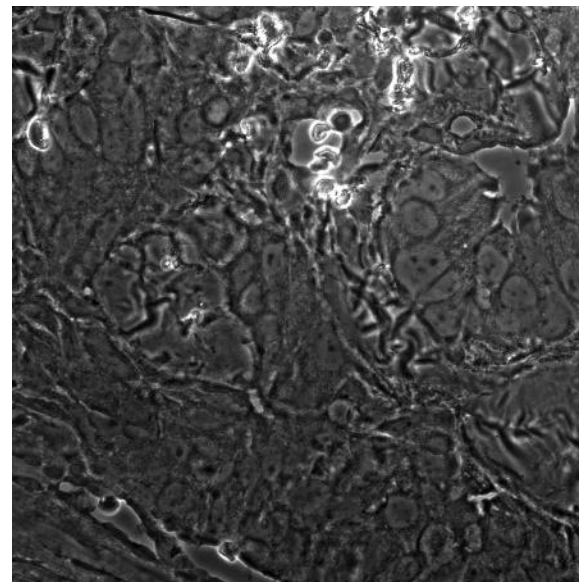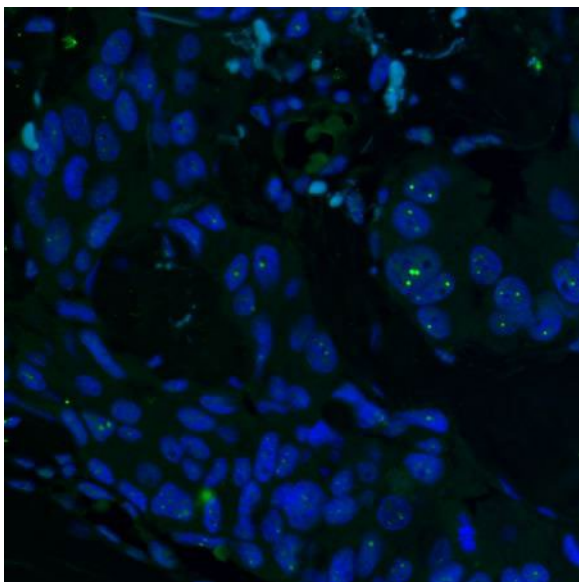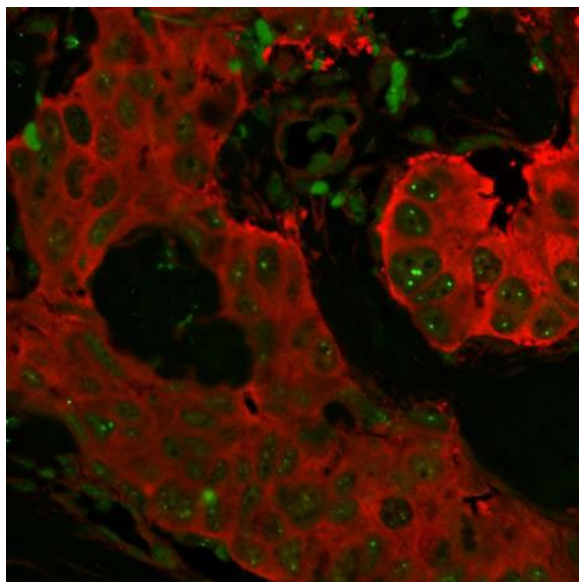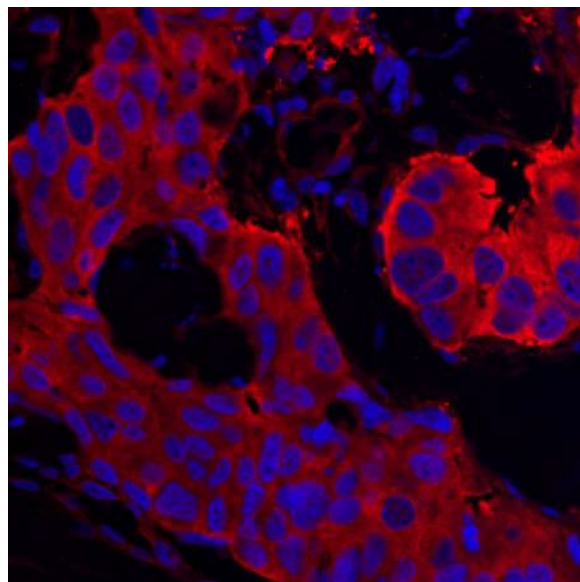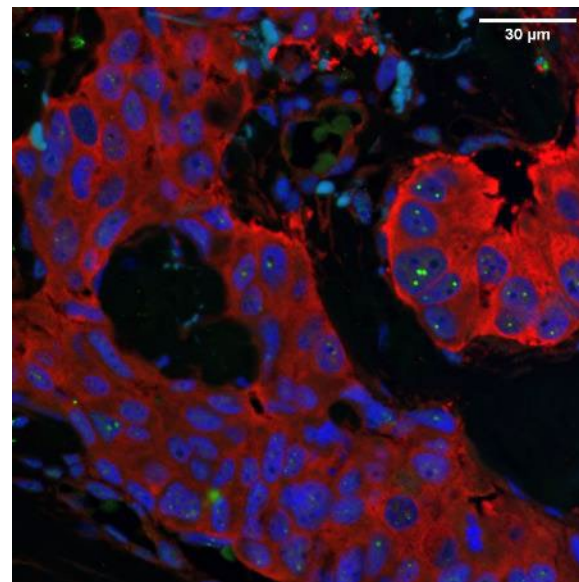

18440\_04

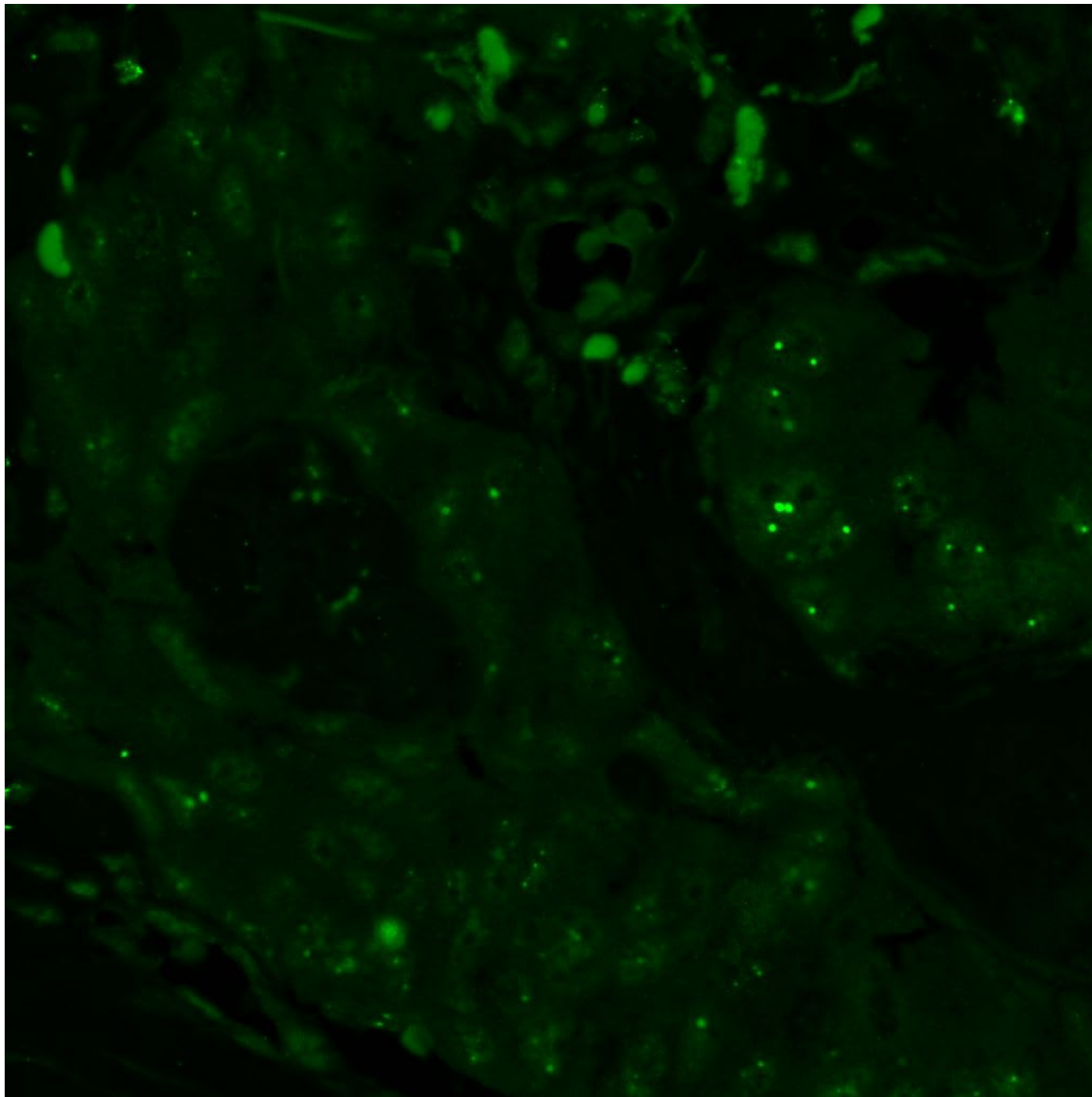

18440\_04

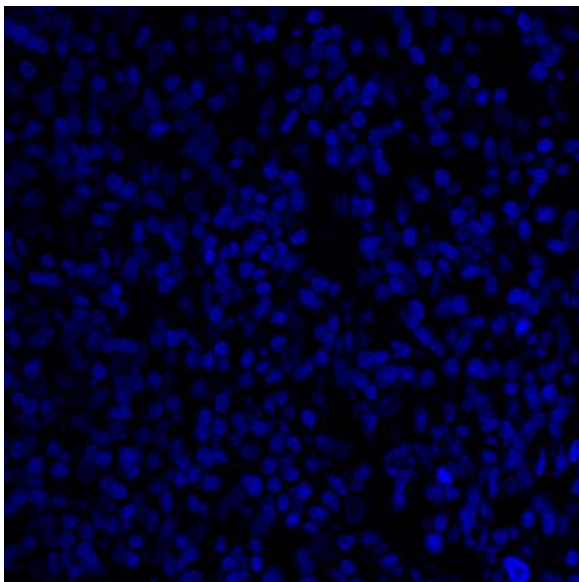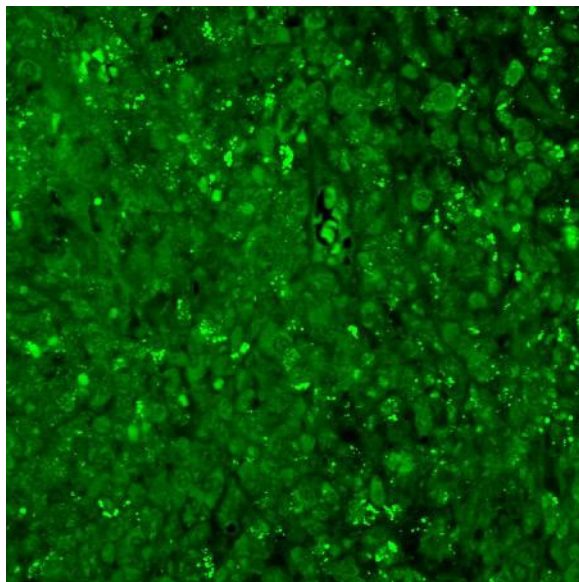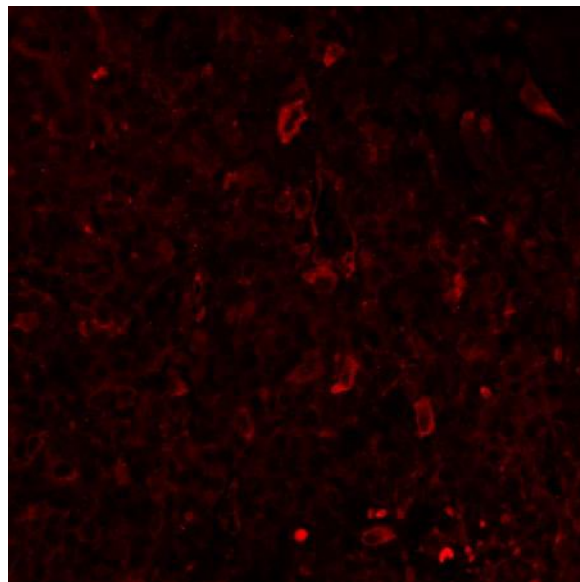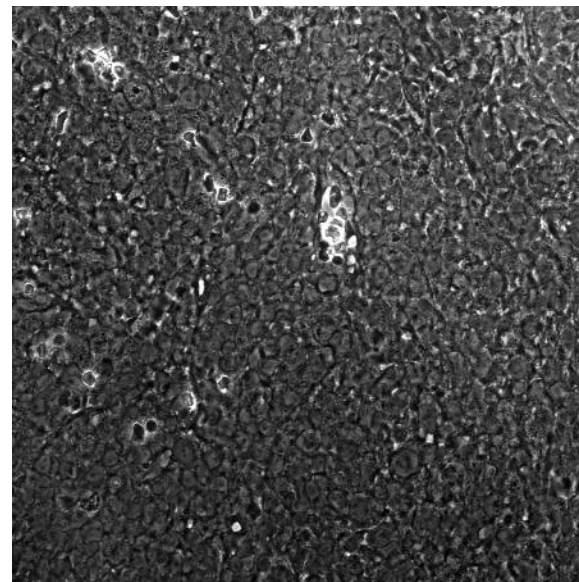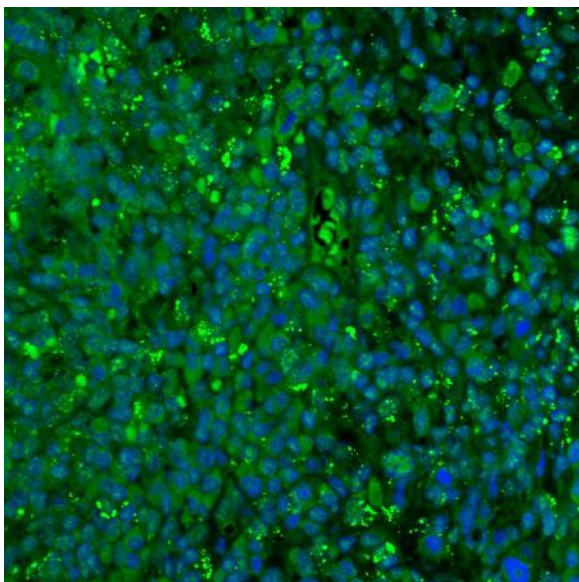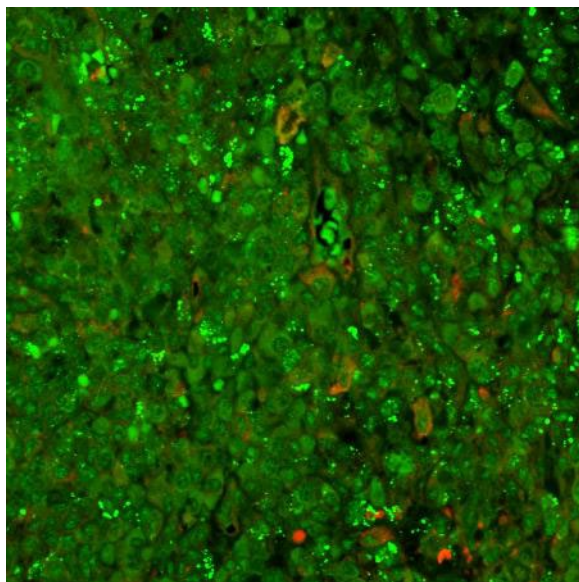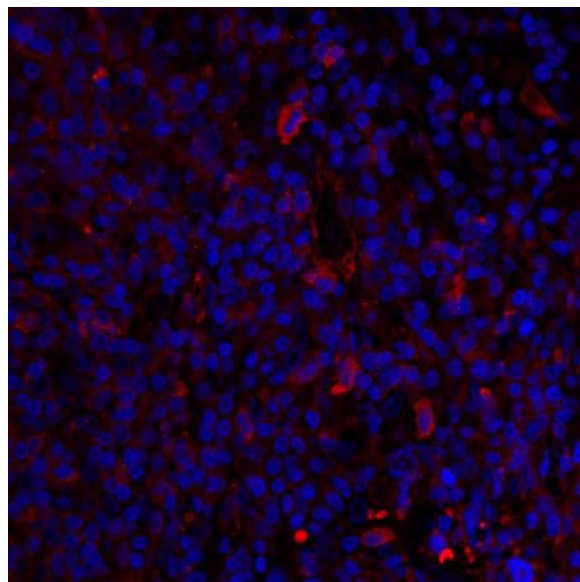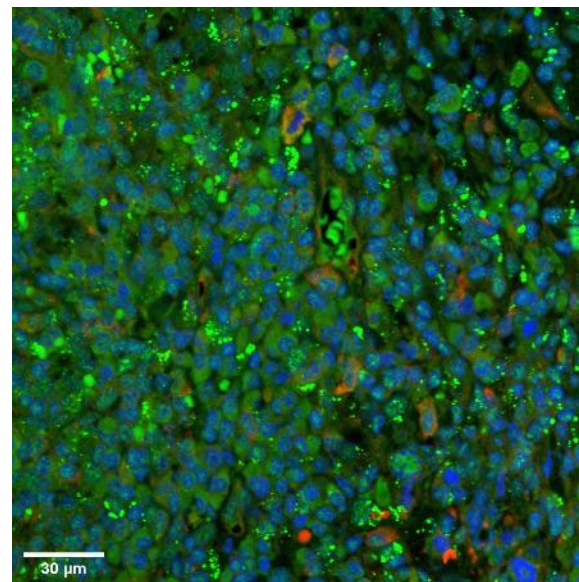

21192\_00

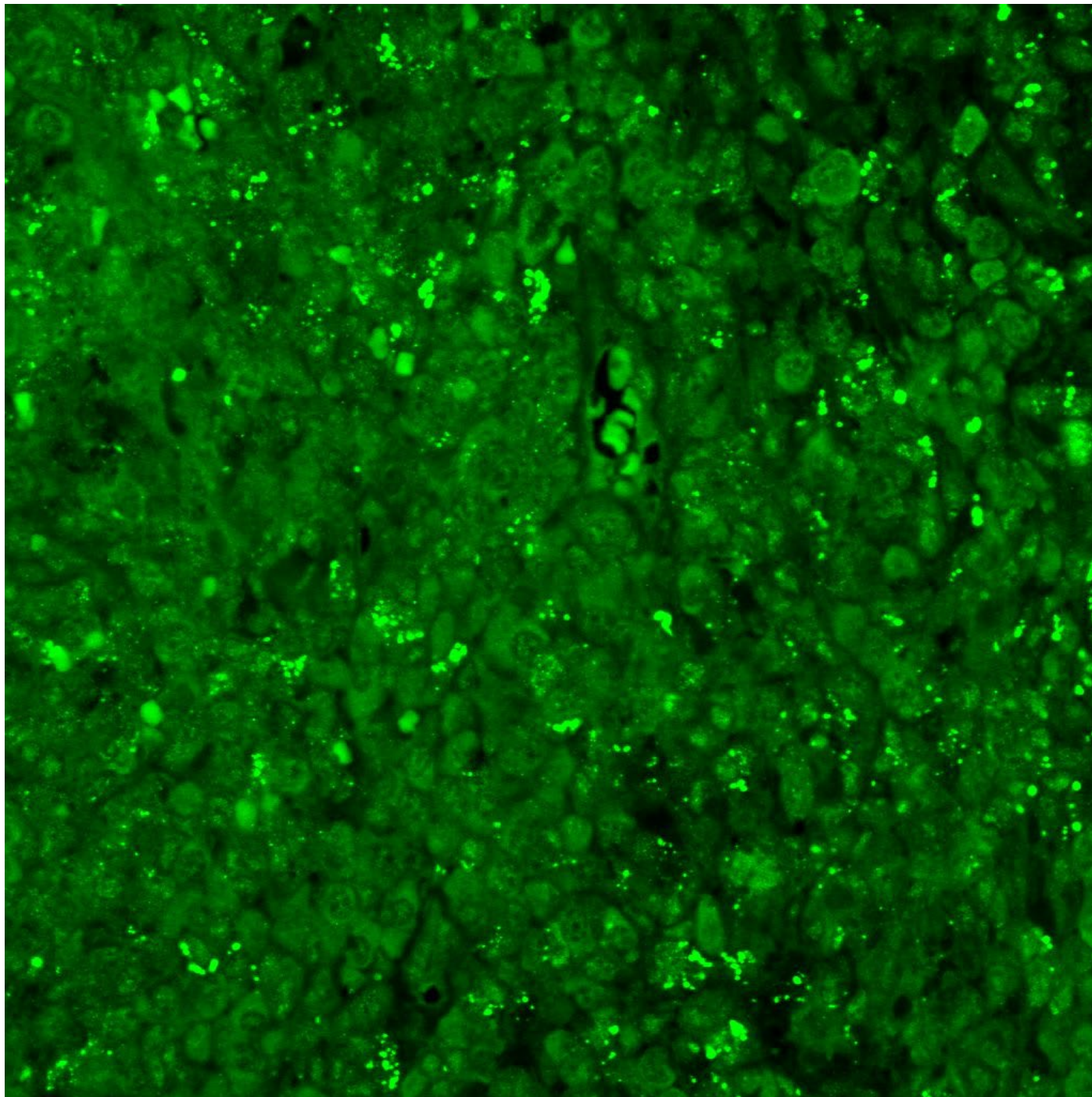

21192\_00

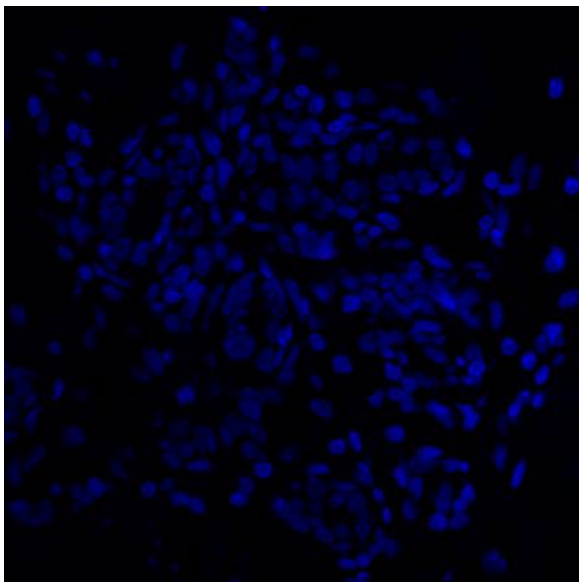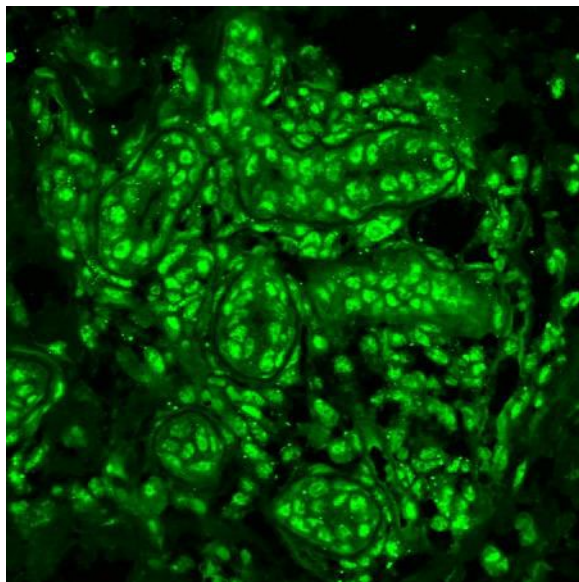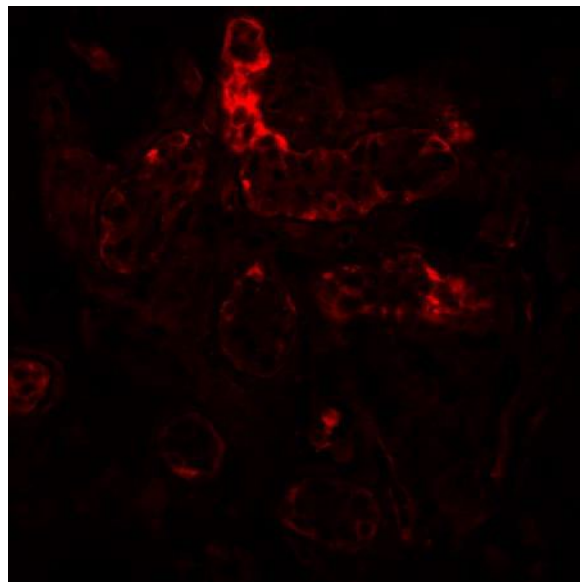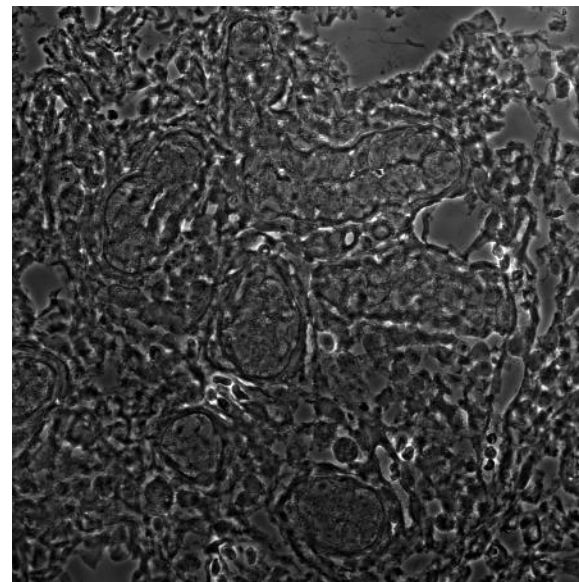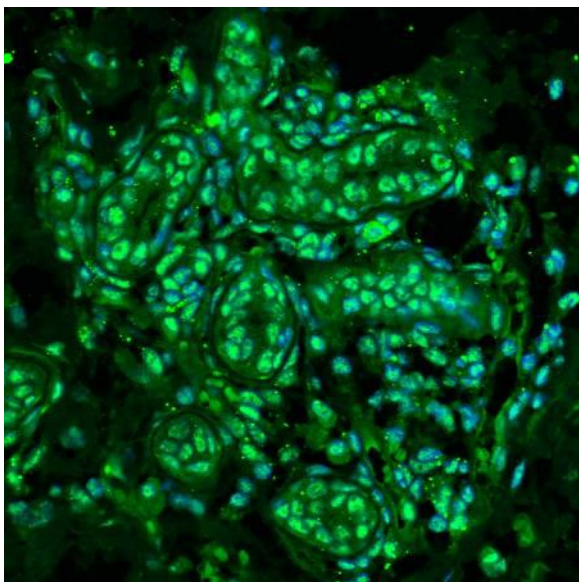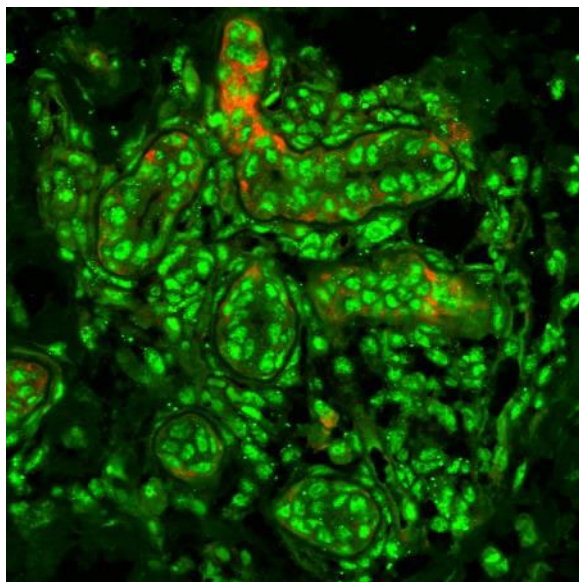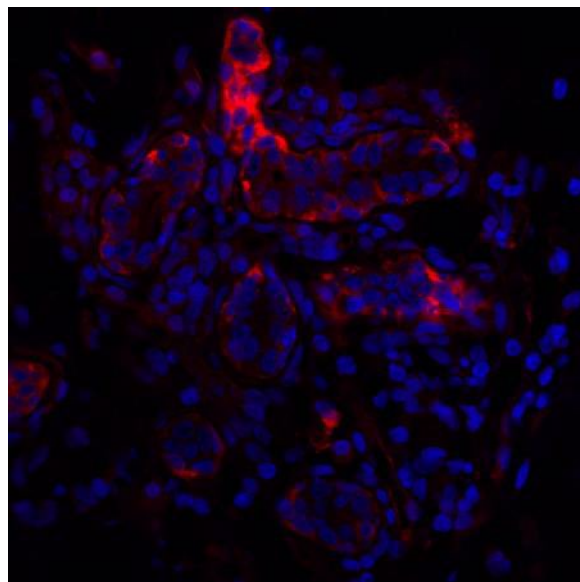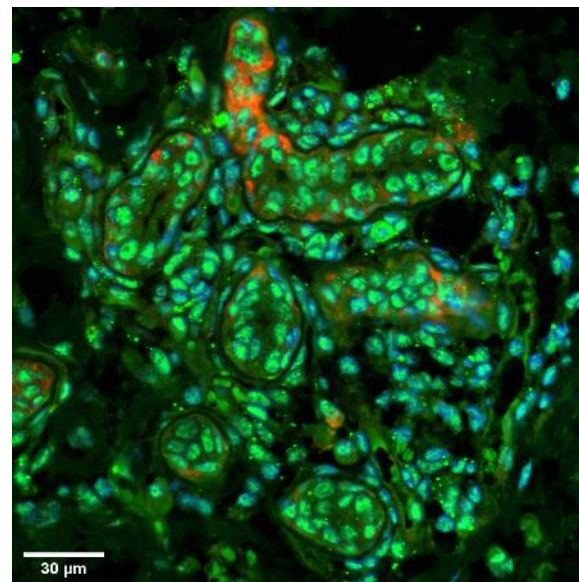

21192\_01

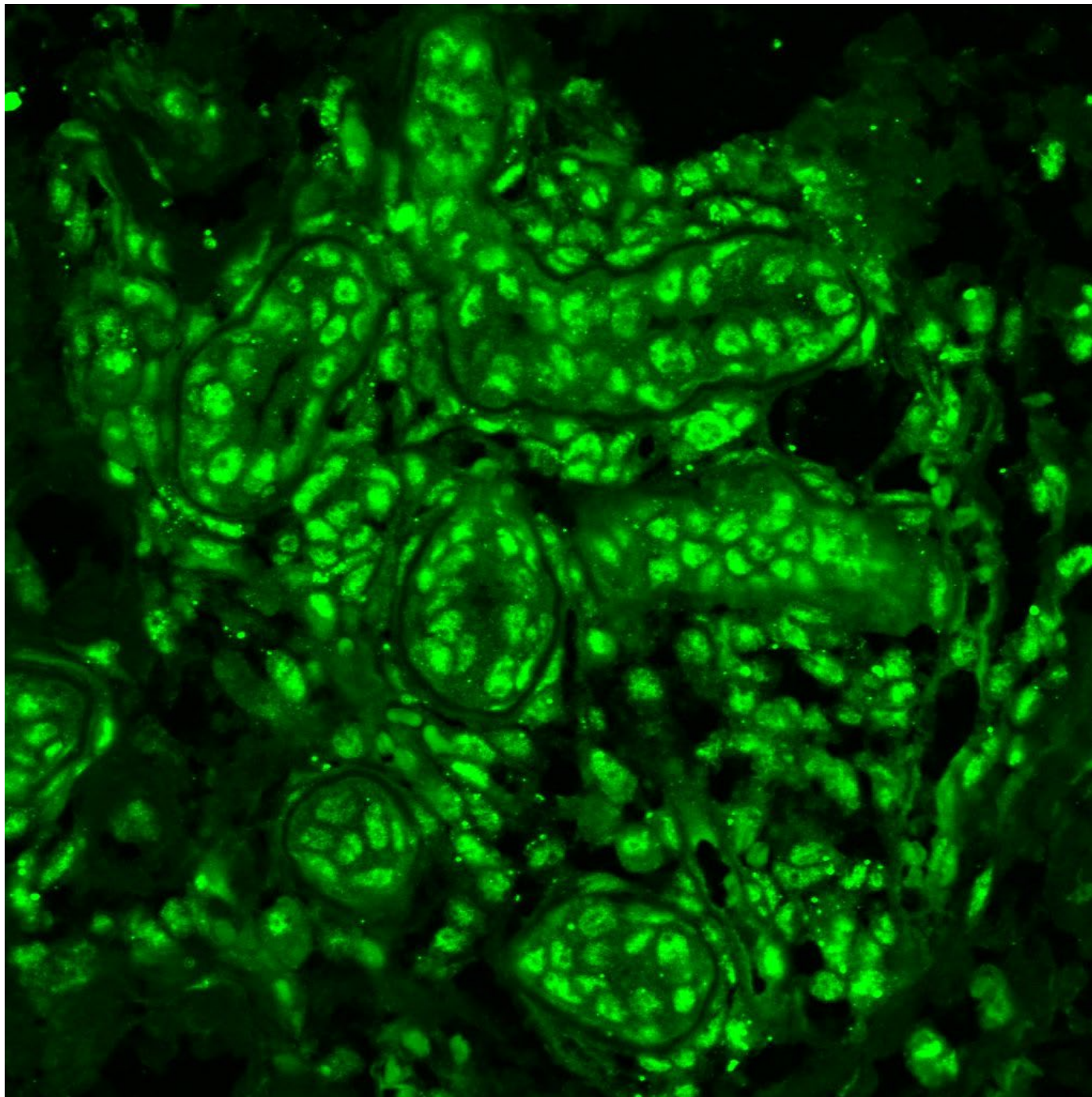

21192\_01

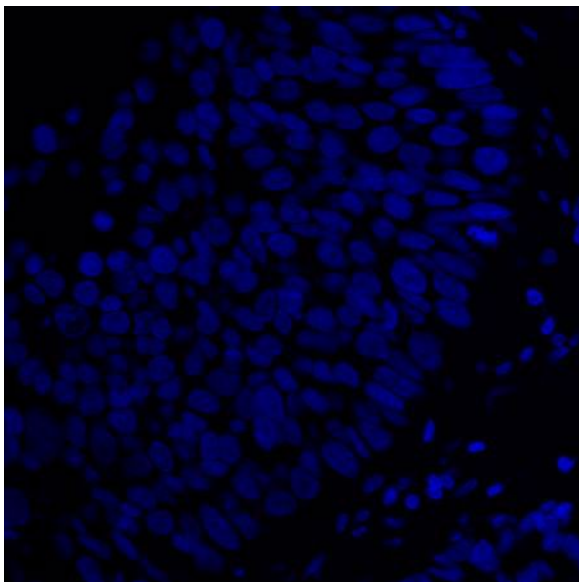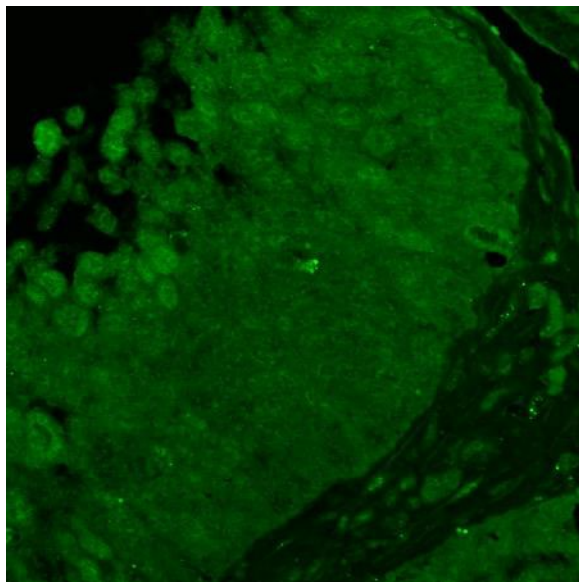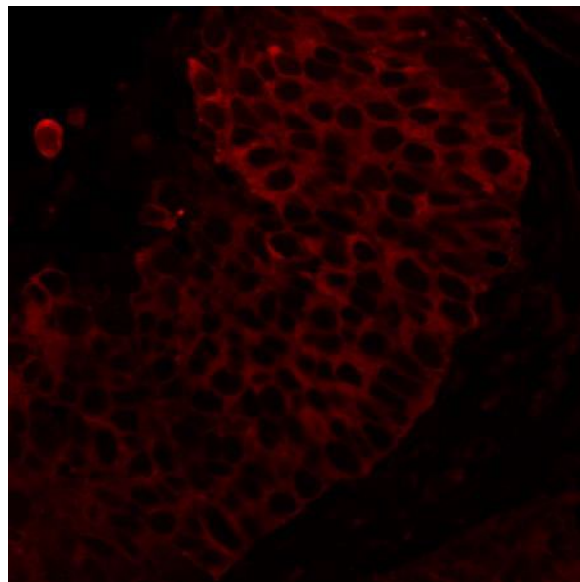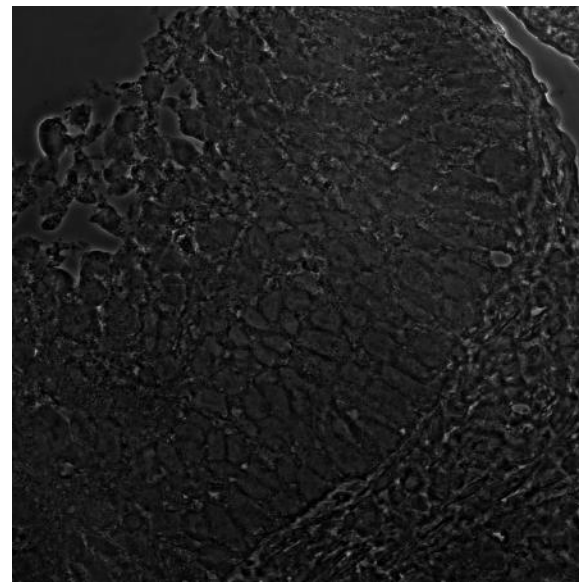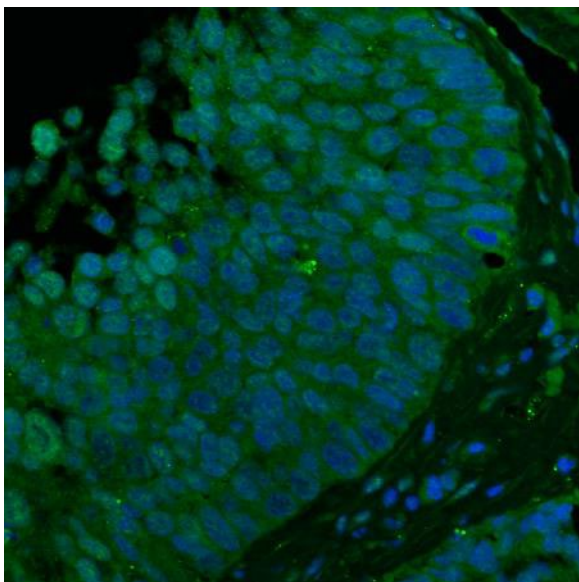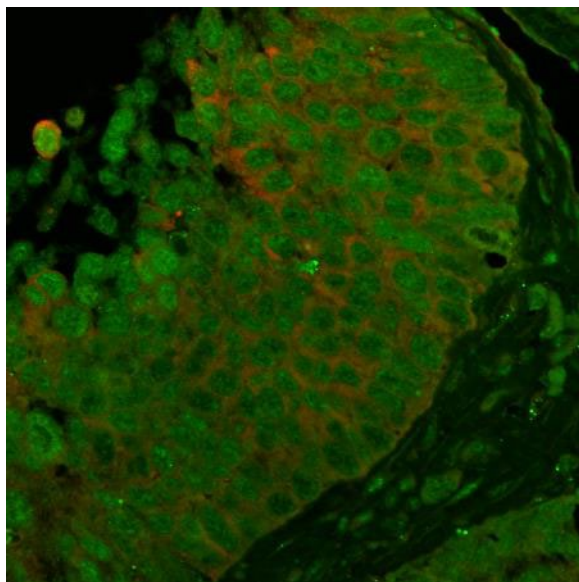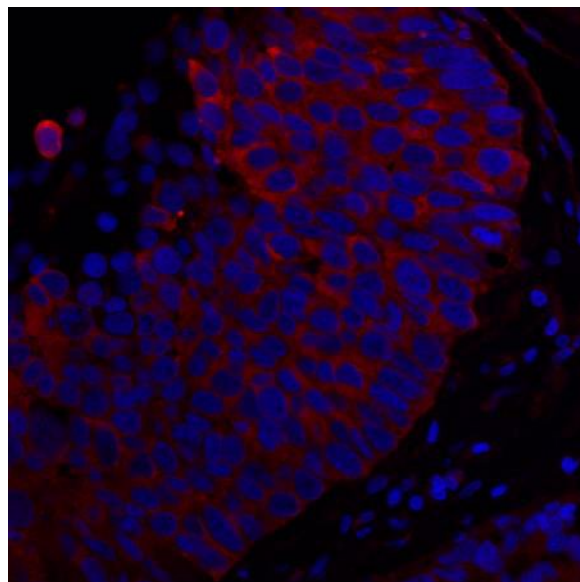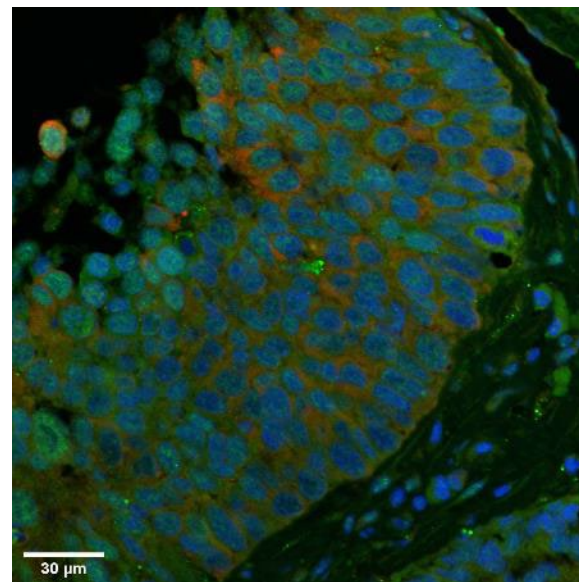

21192\_02

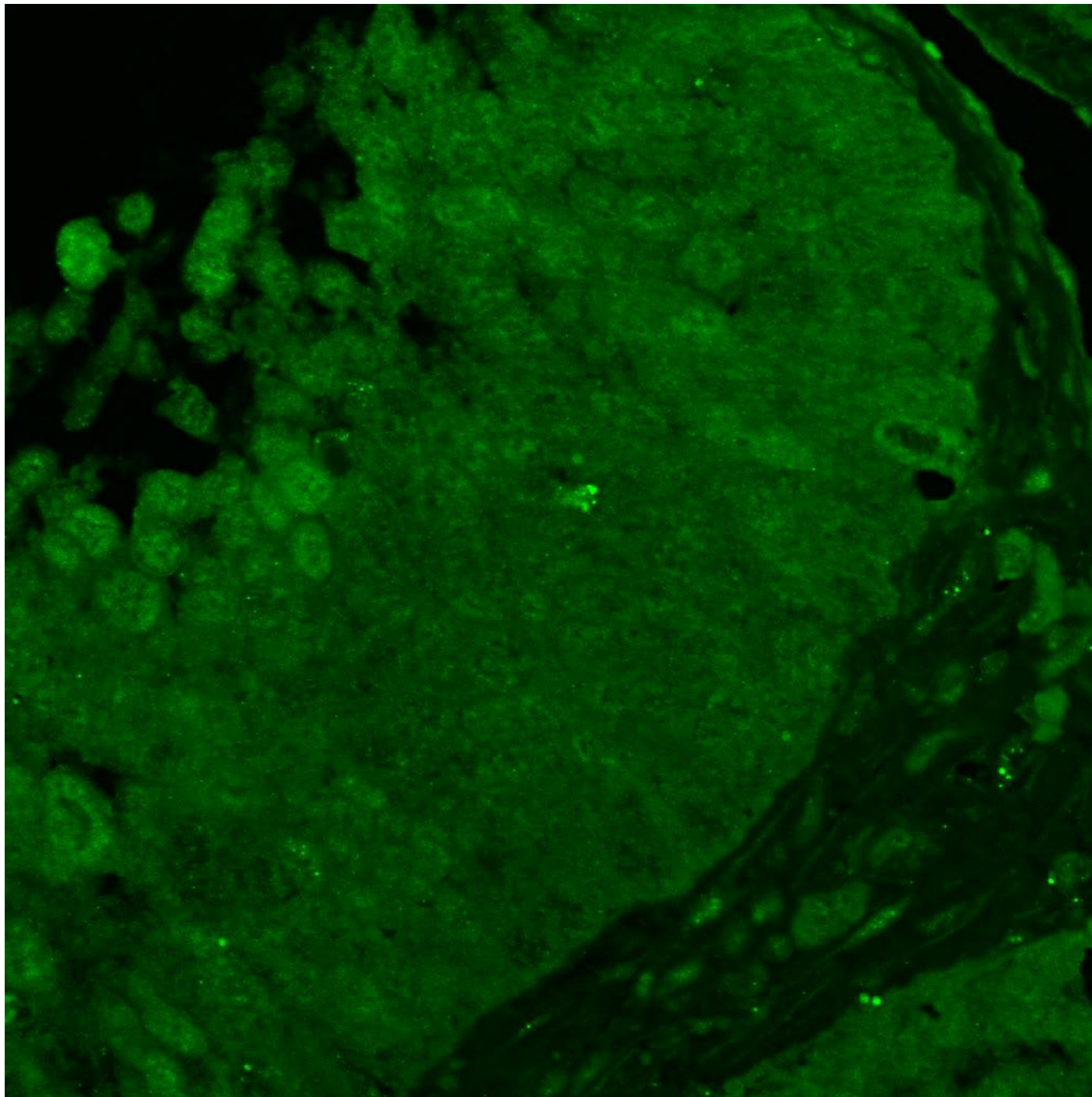

21192\_02

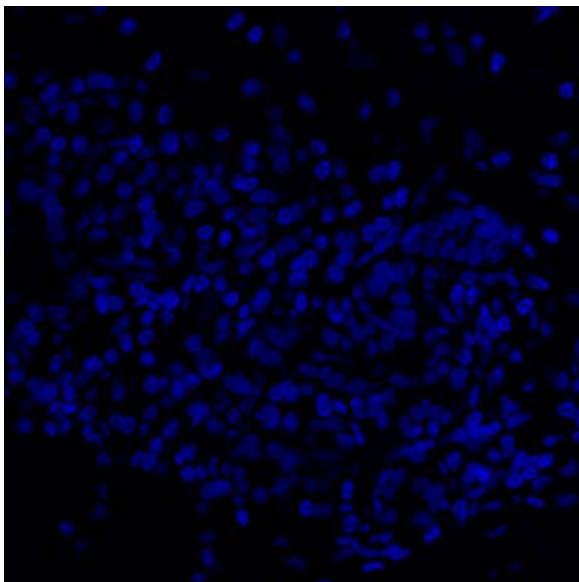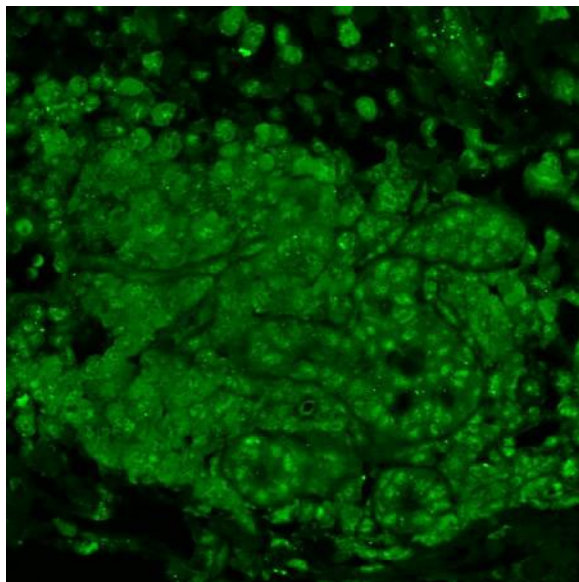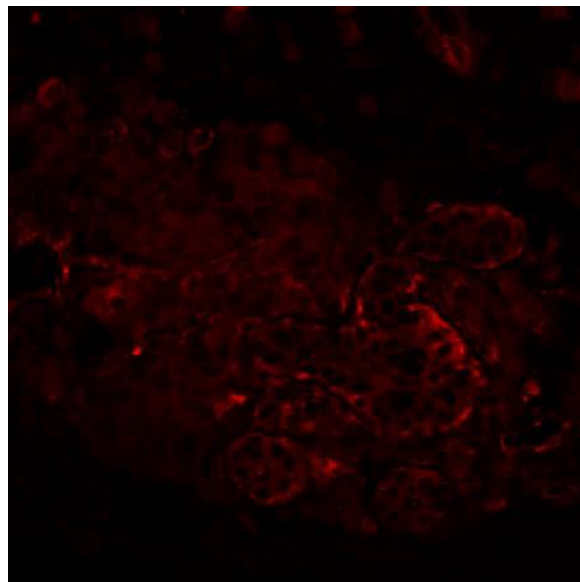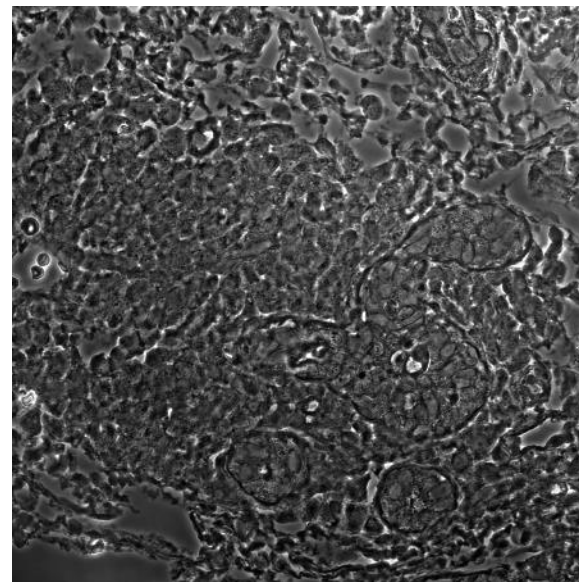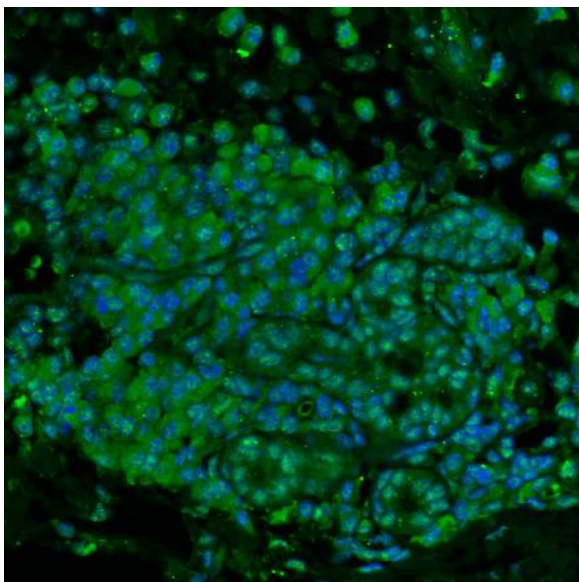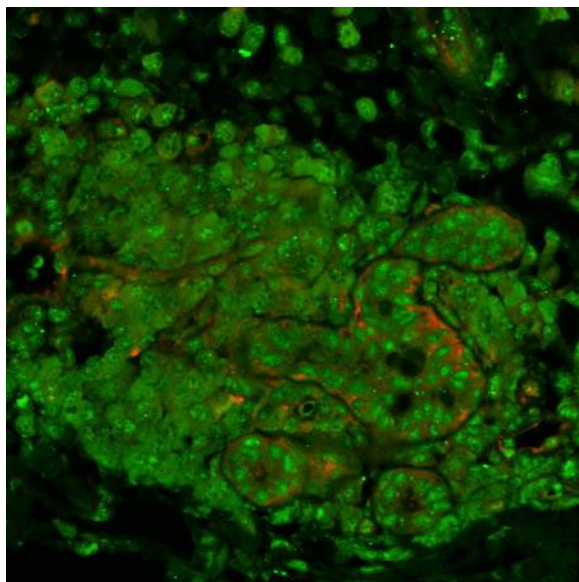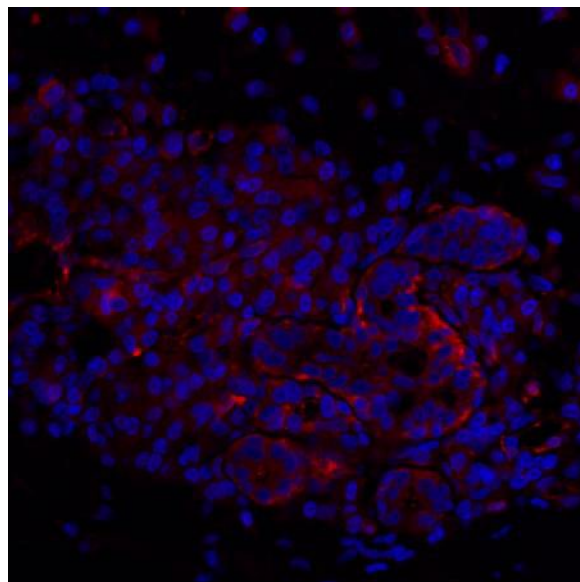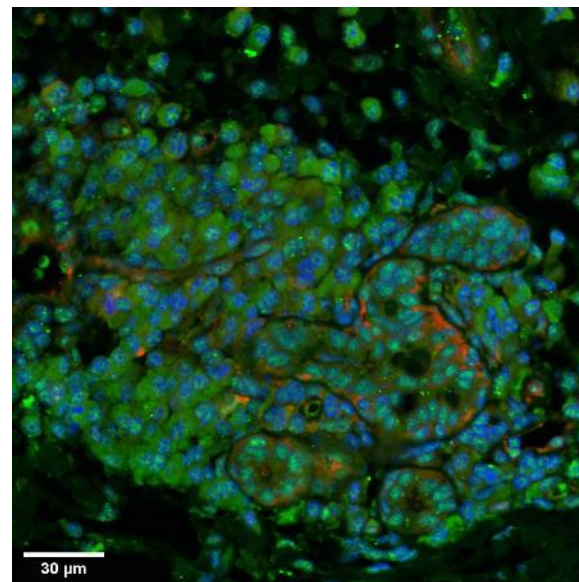

21192\_03

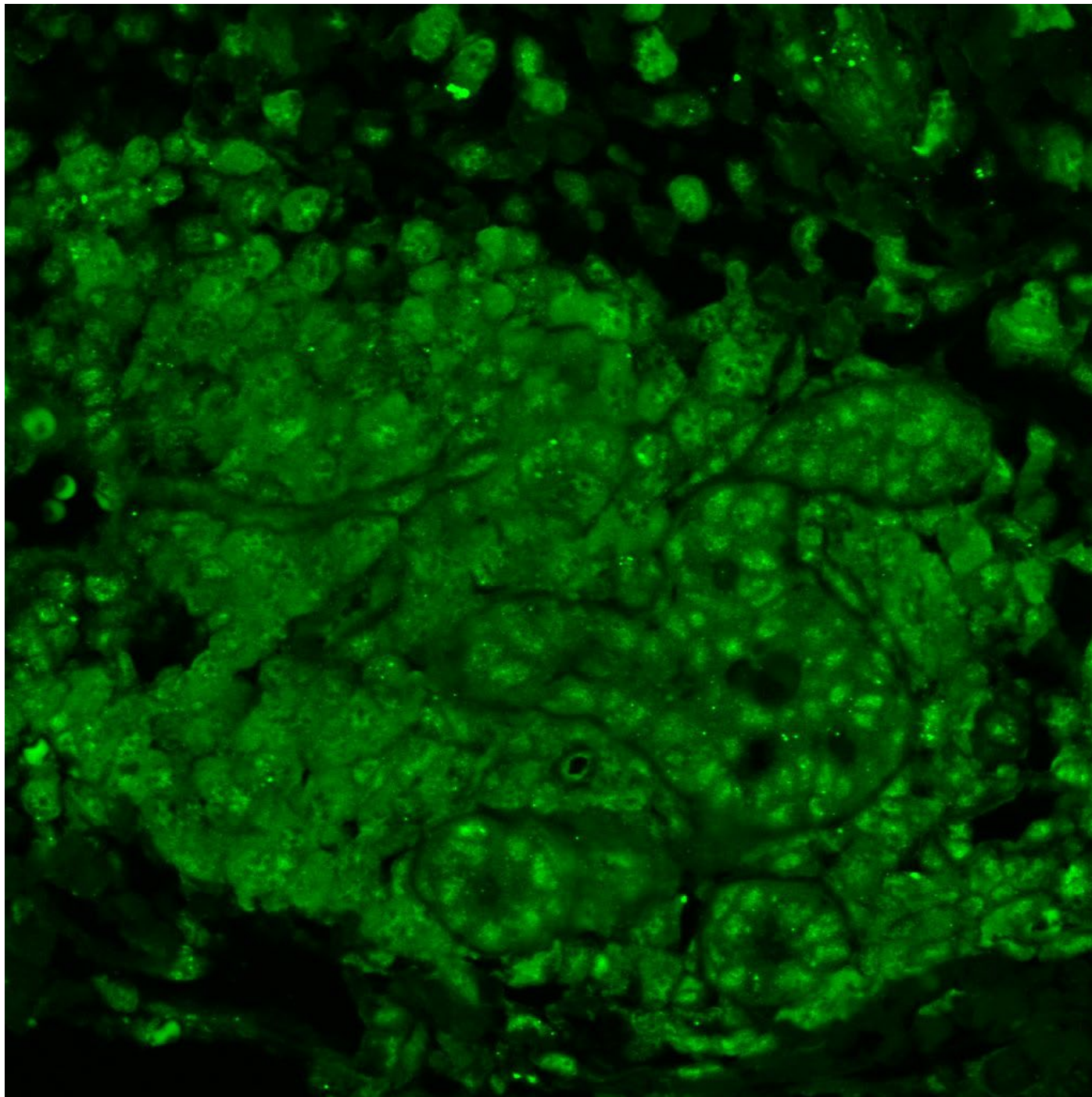

21192\_03

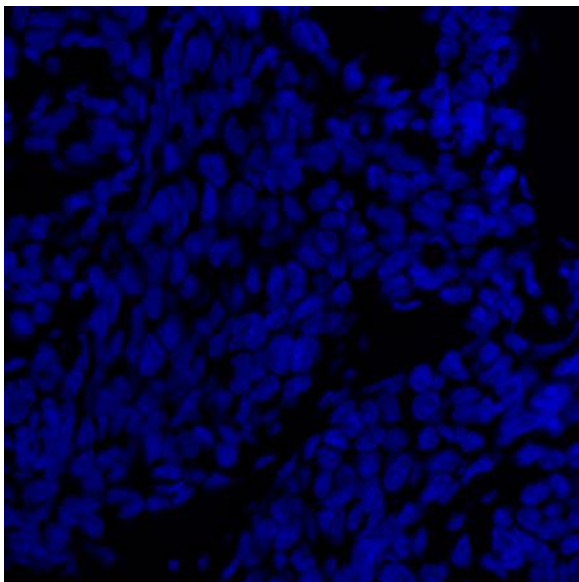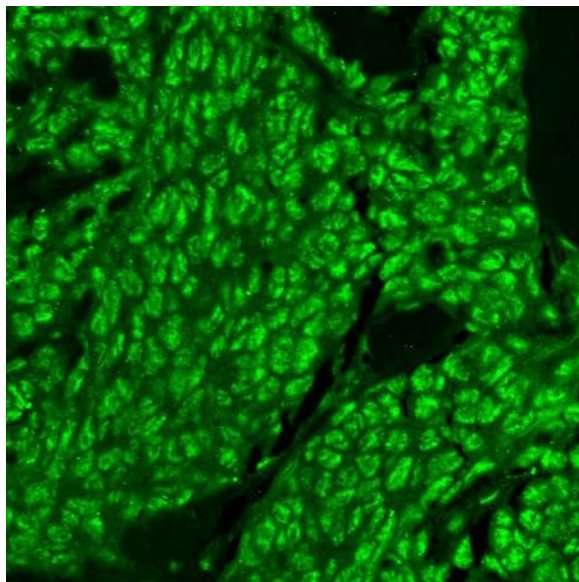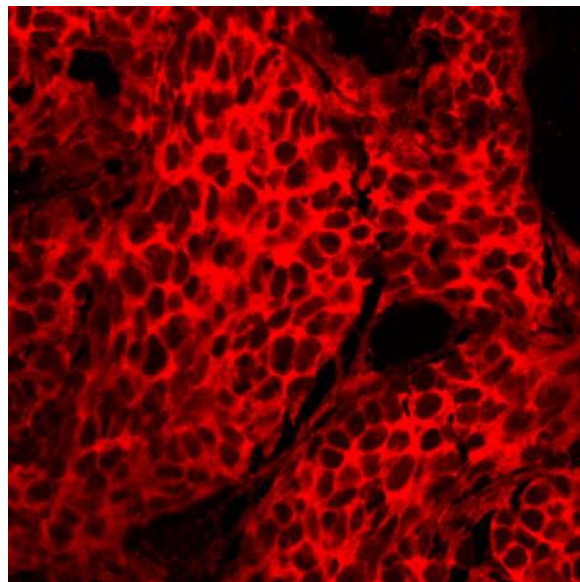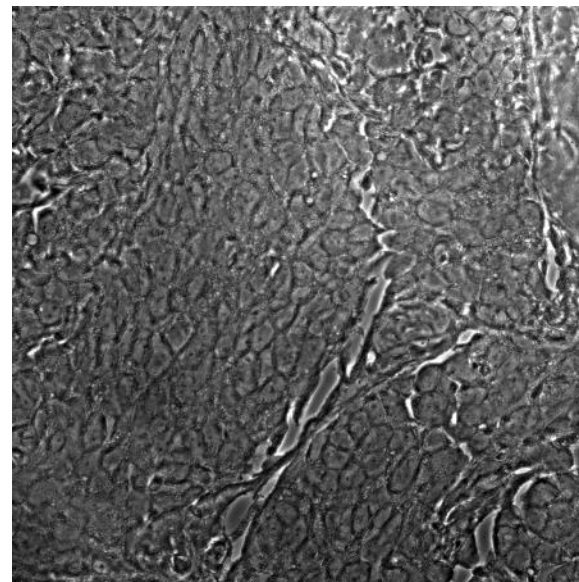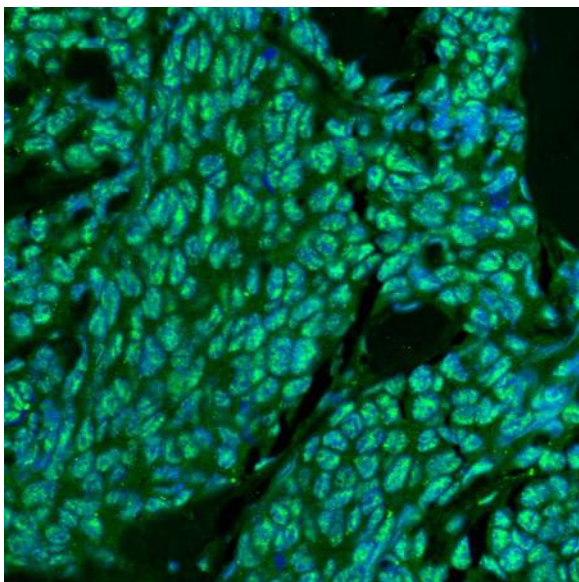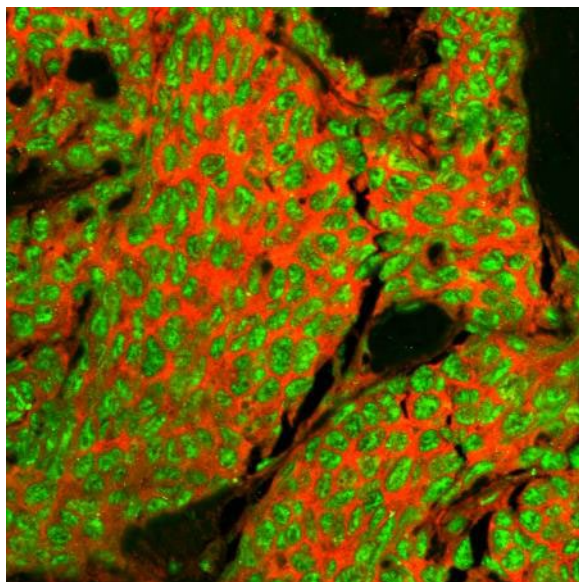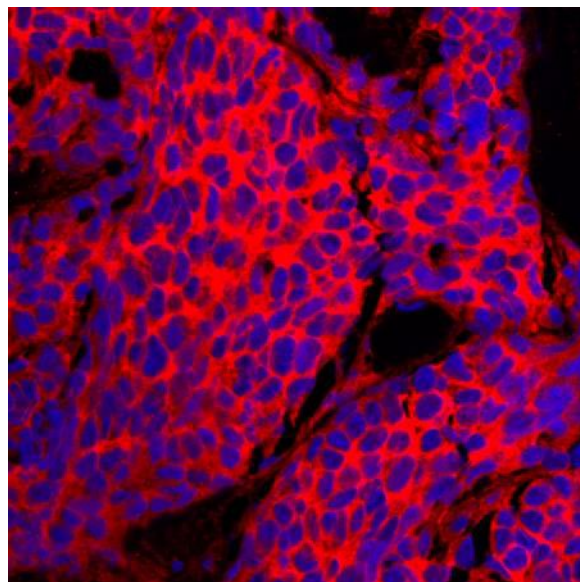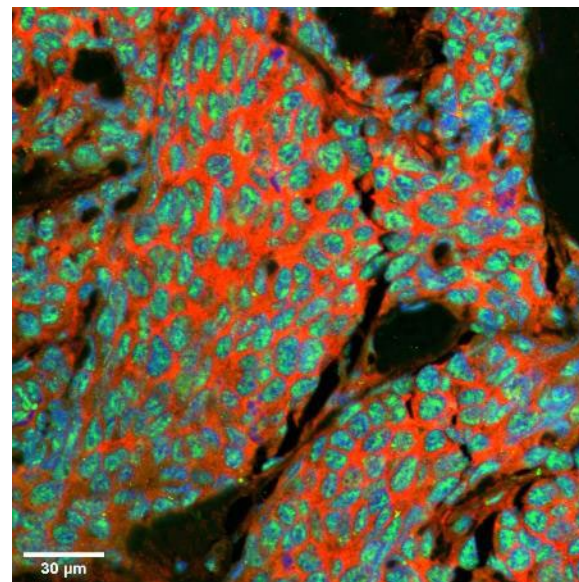

21825\_00

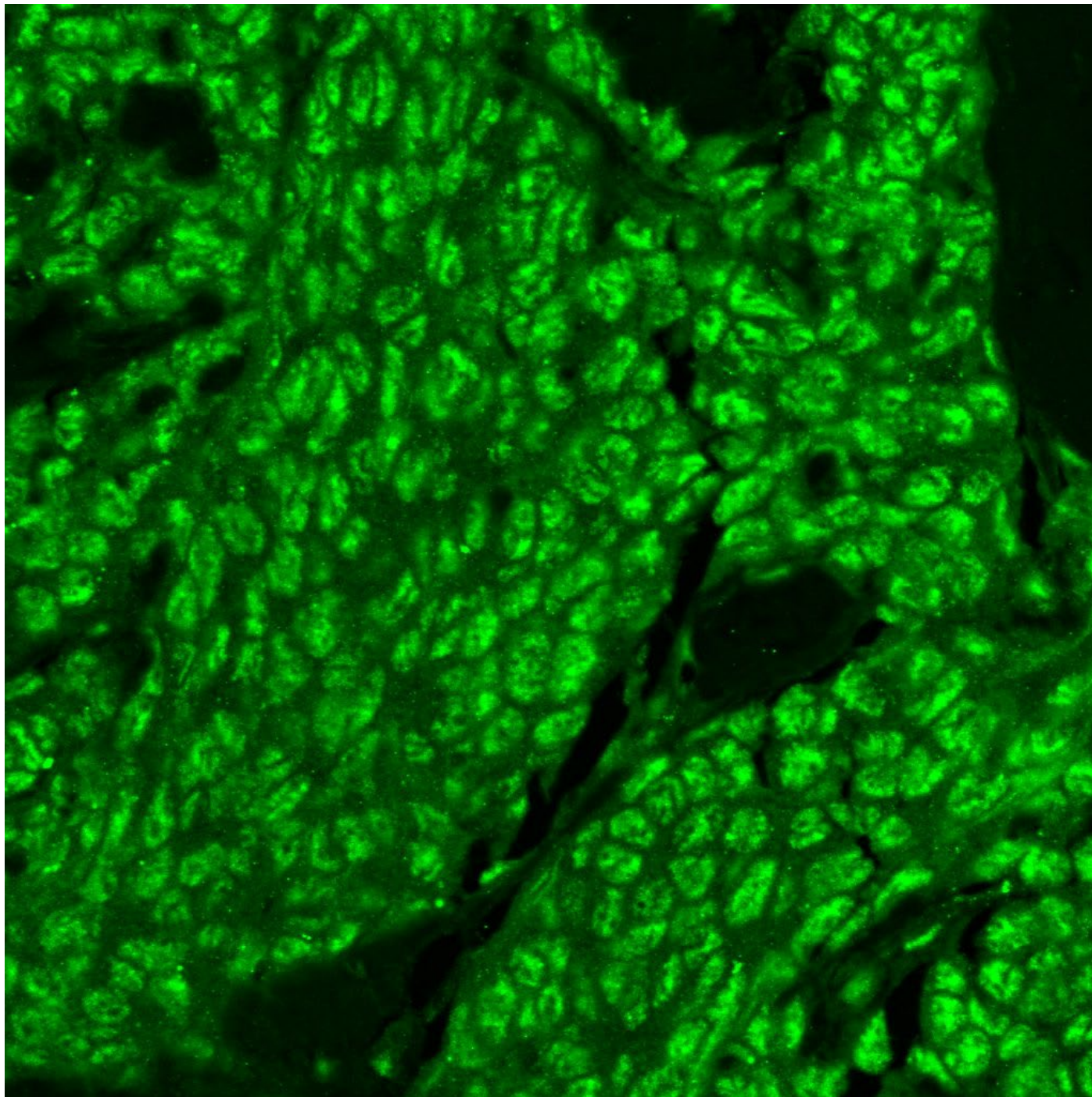

21825\_00

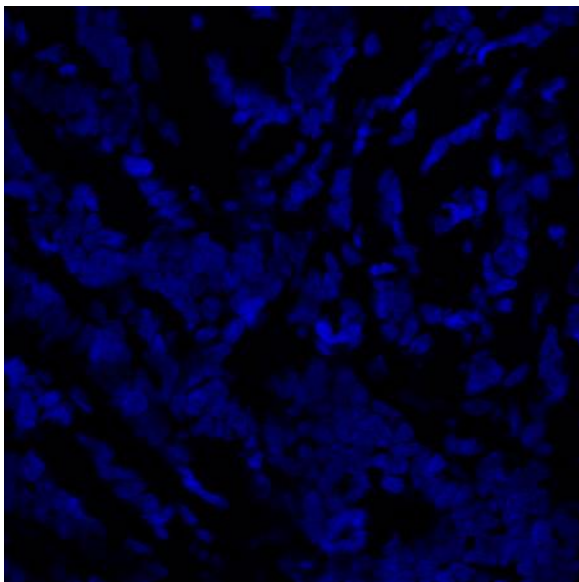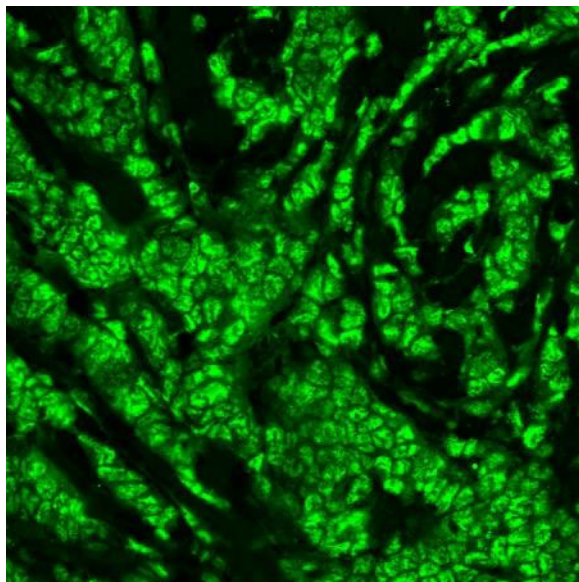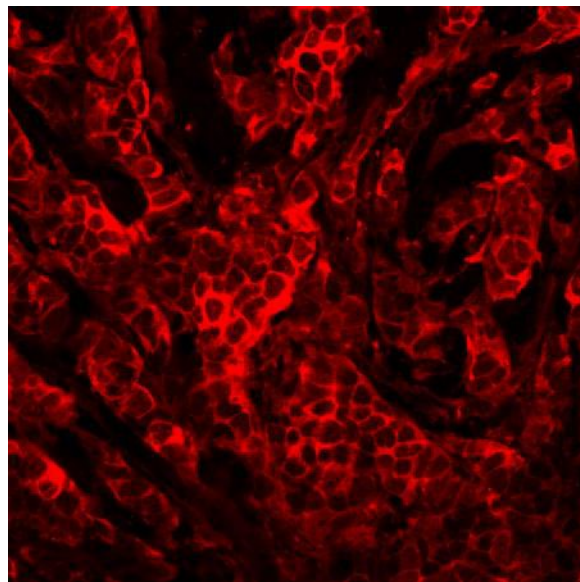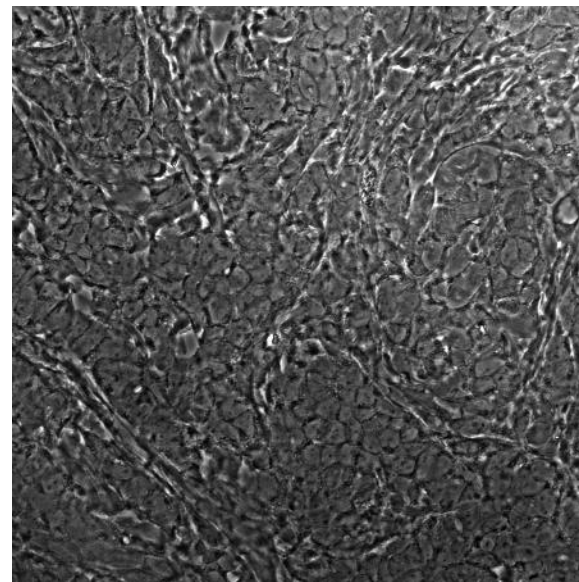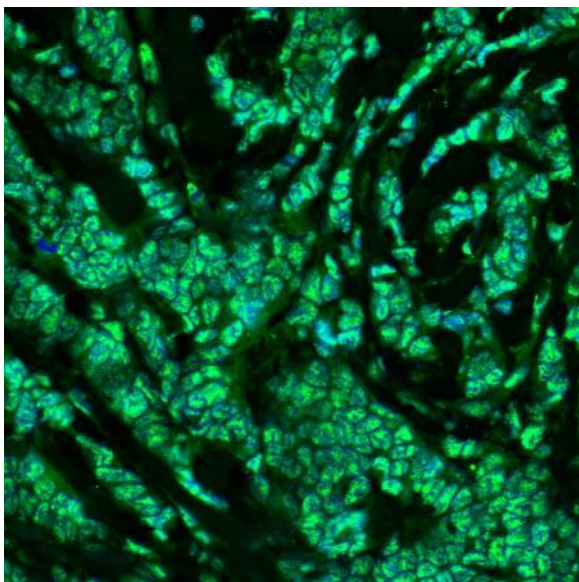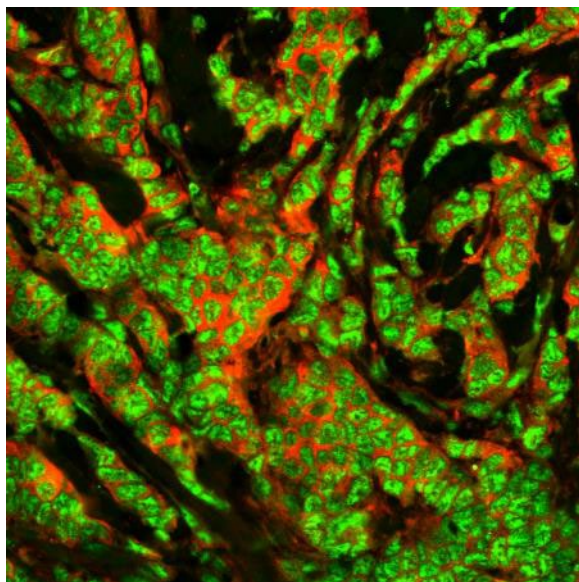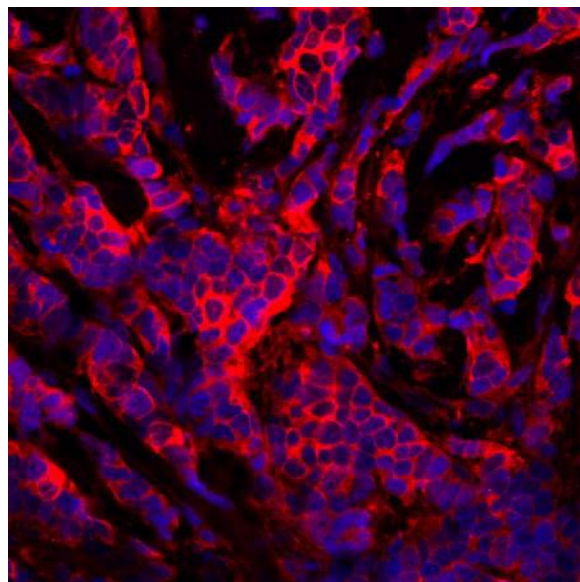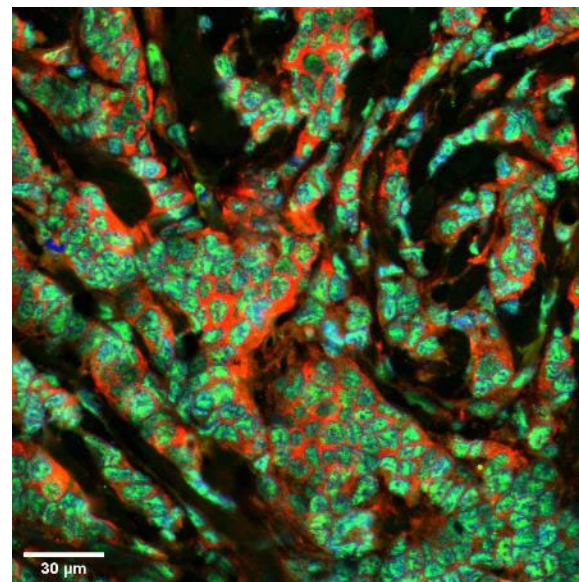

21825\_01

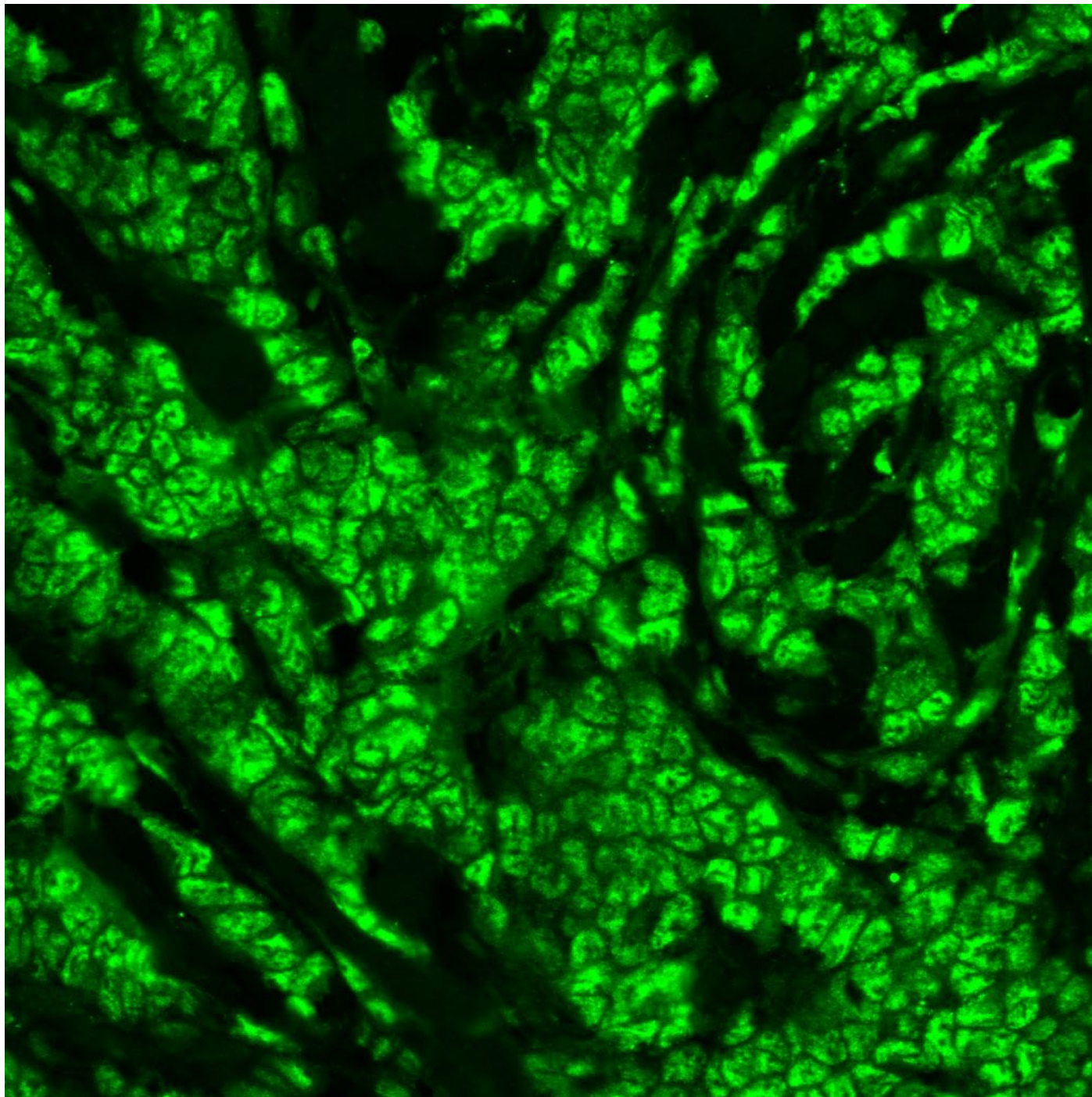

21825\_01

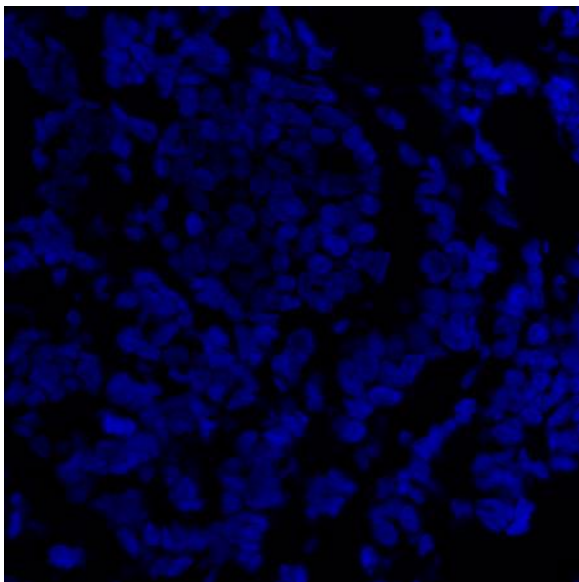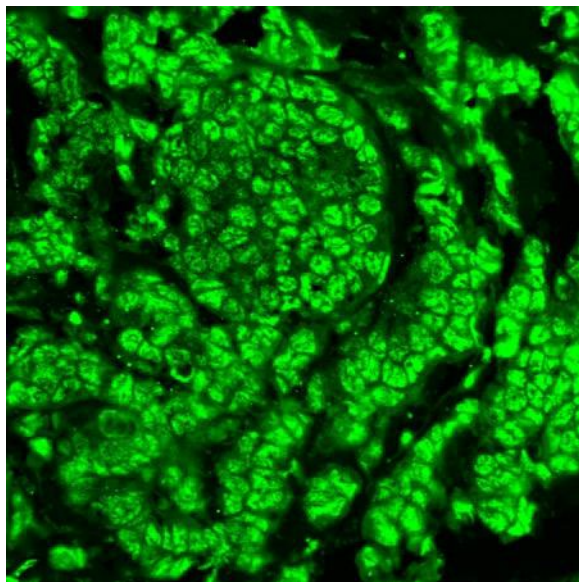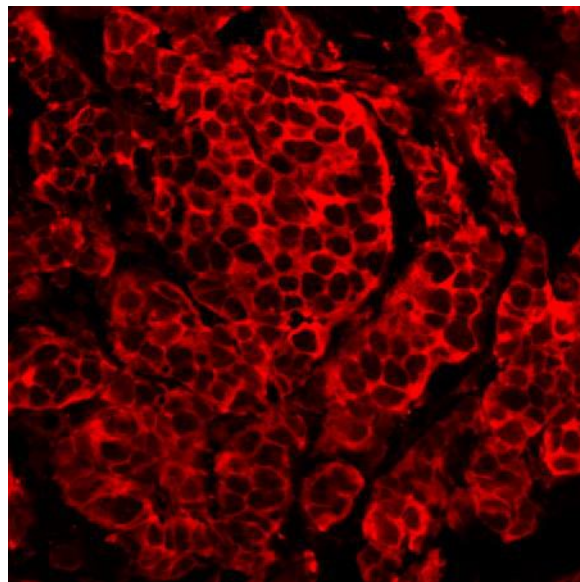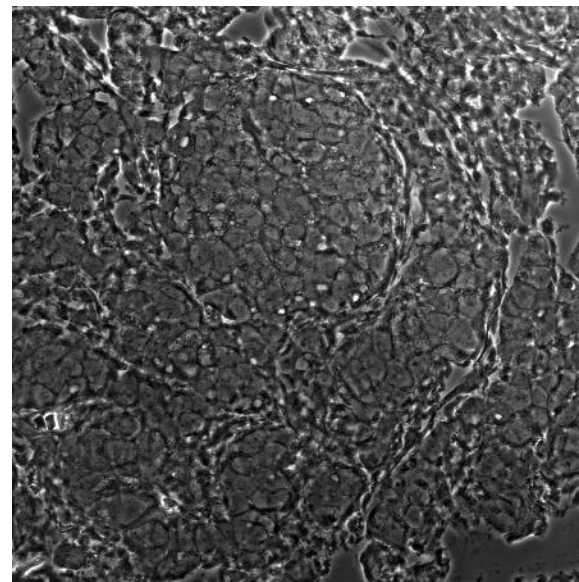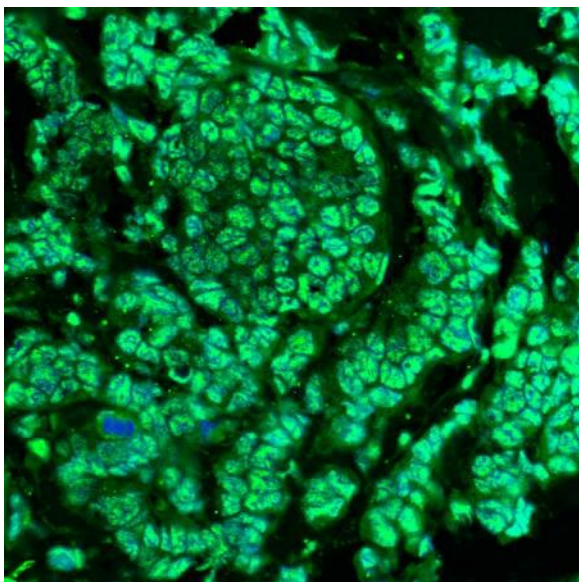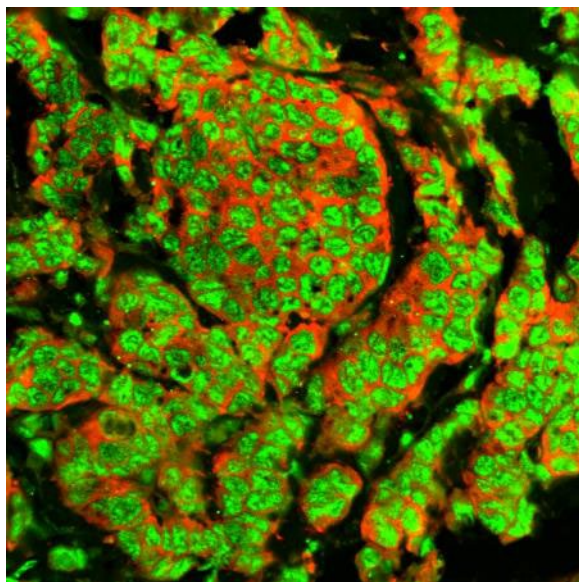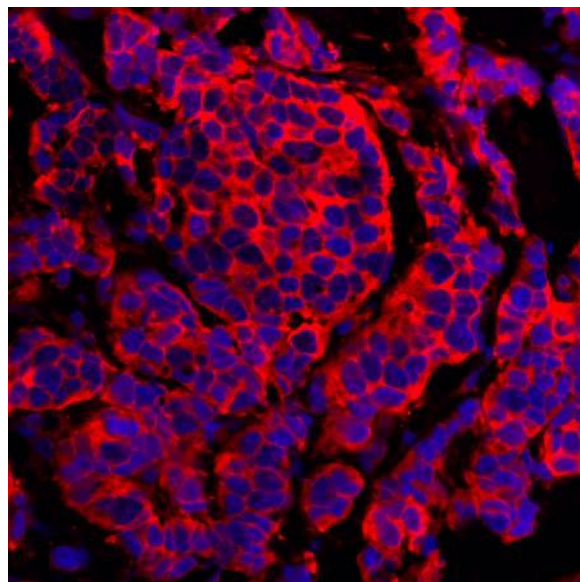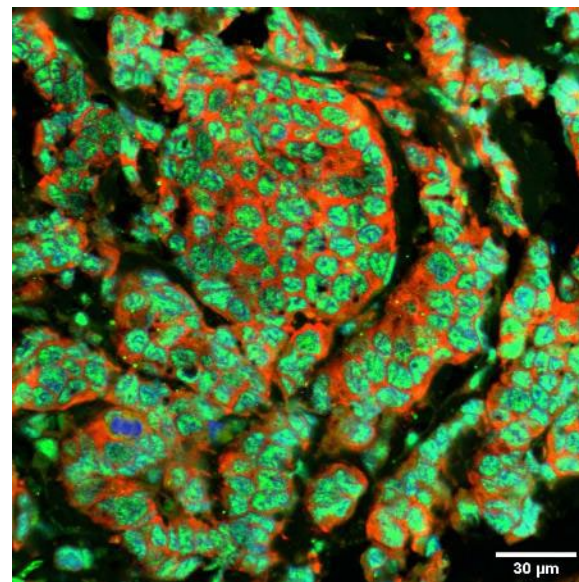

21825\_02

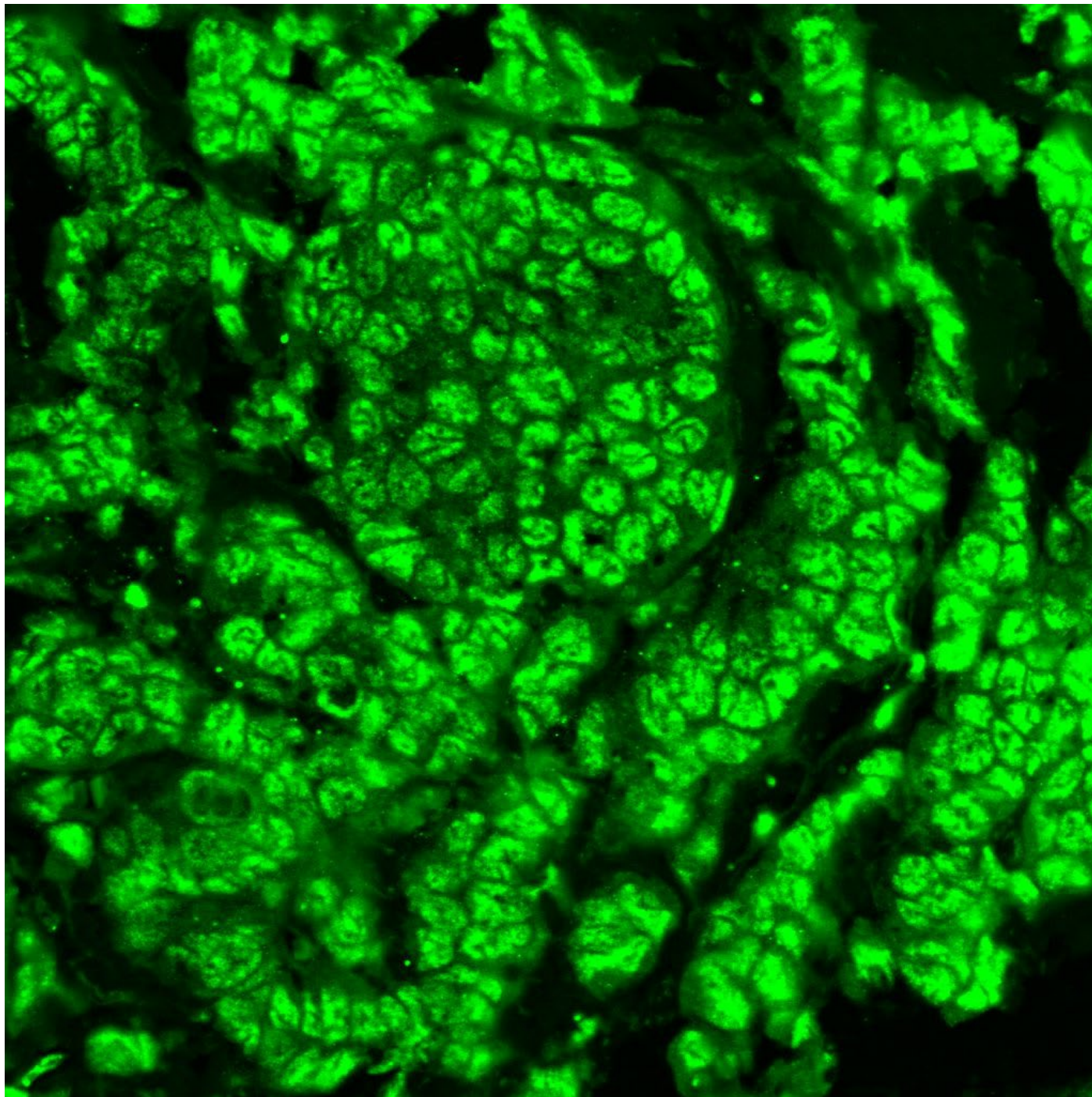

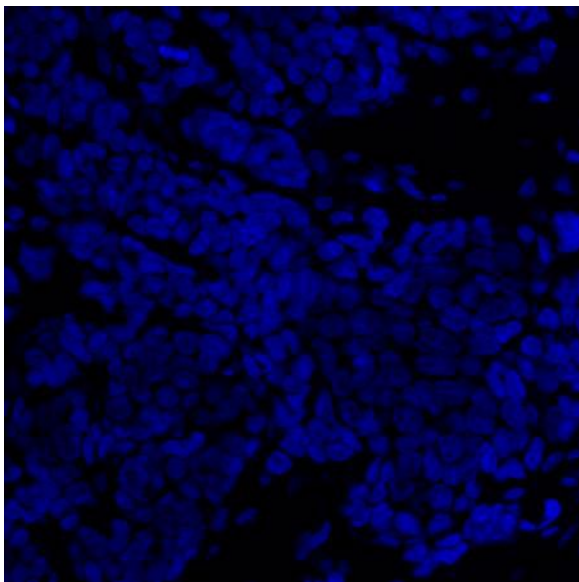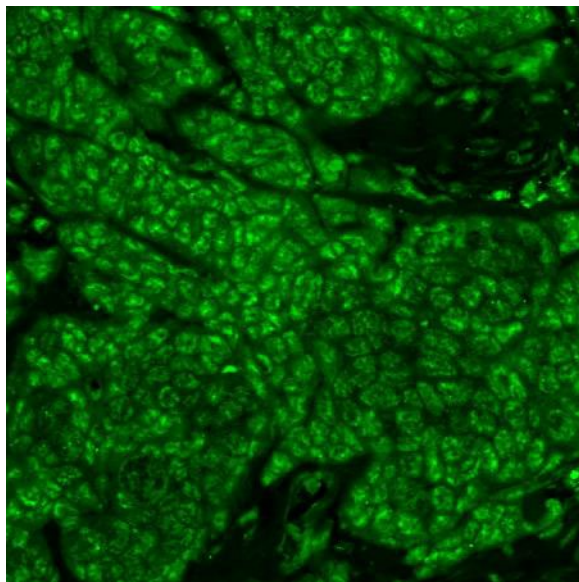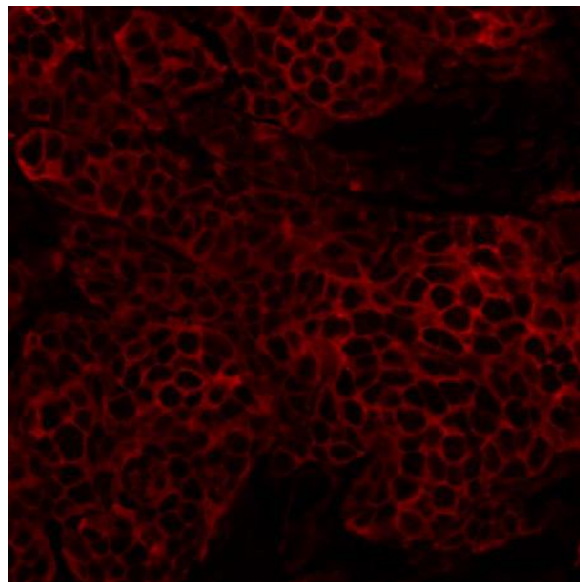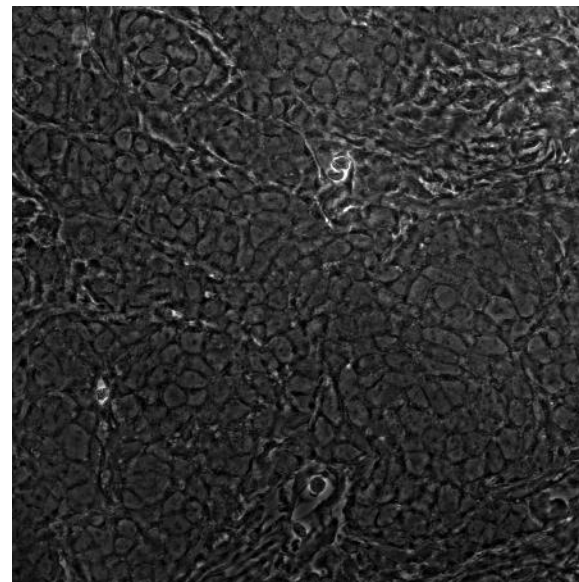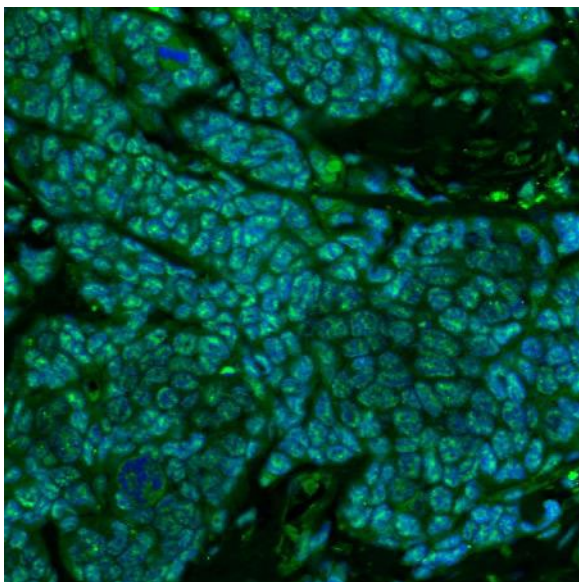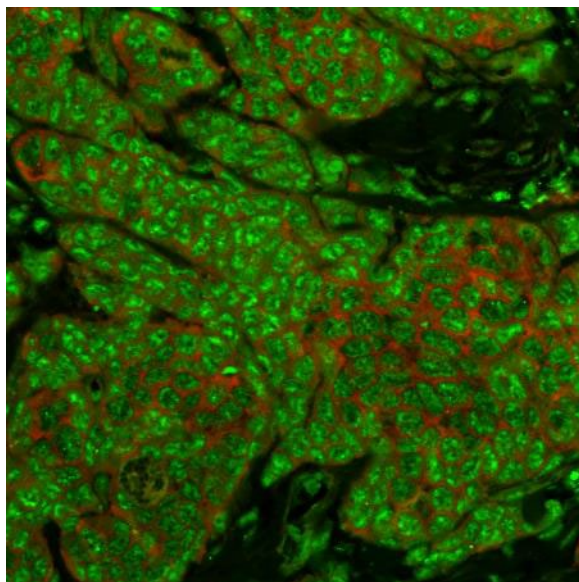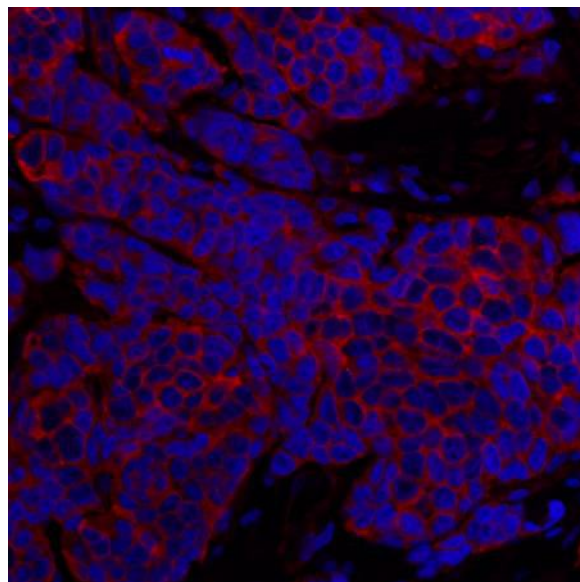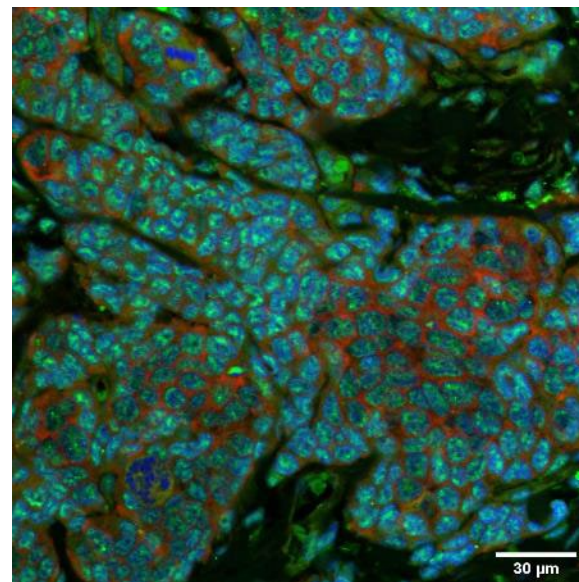

21825\_03

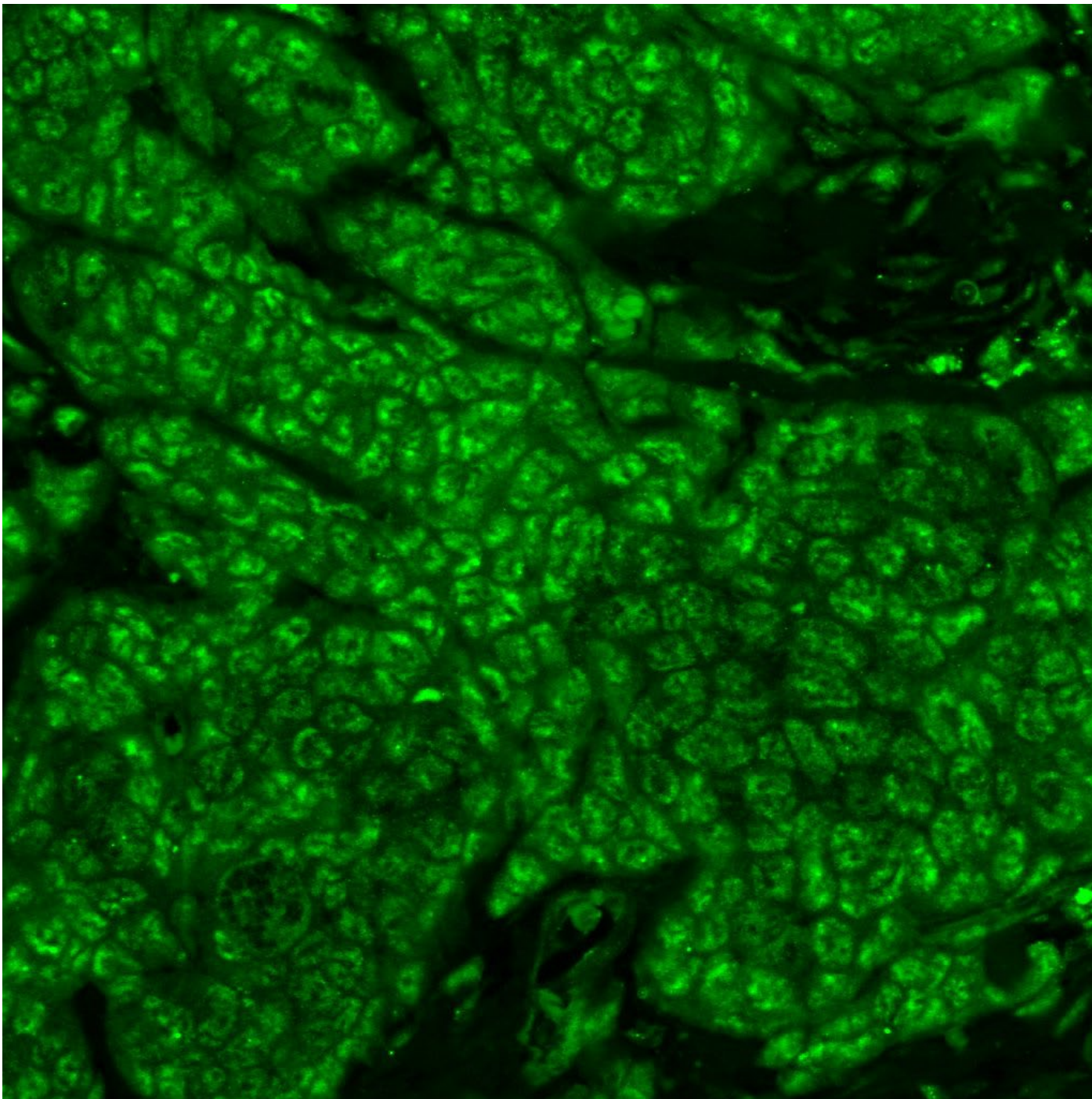

21825\_03

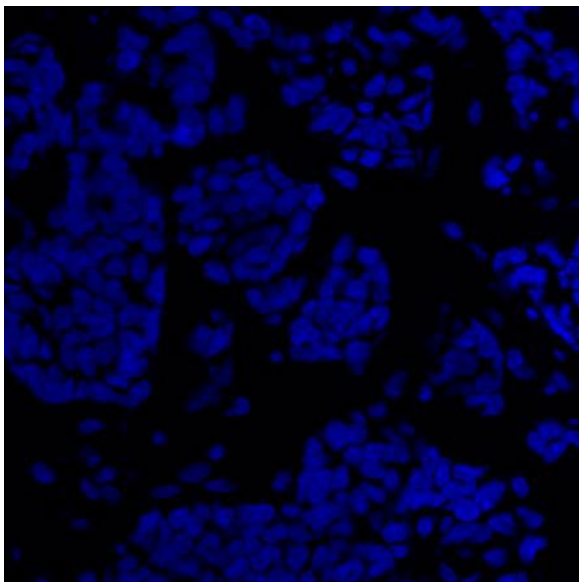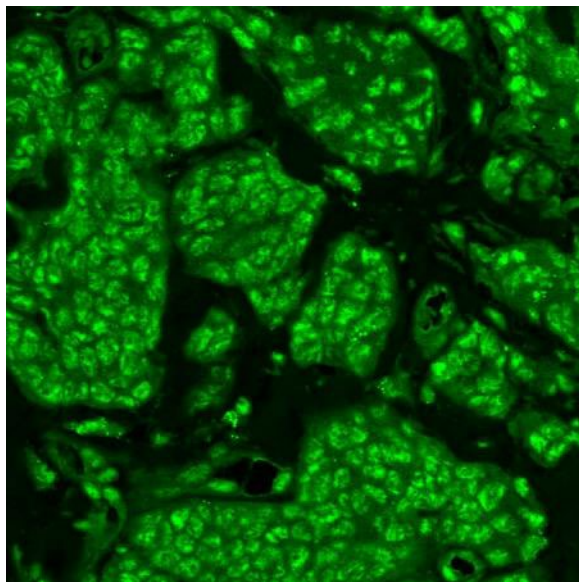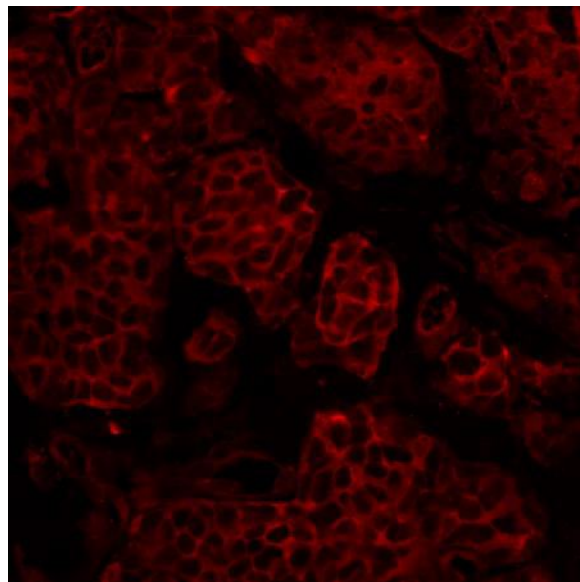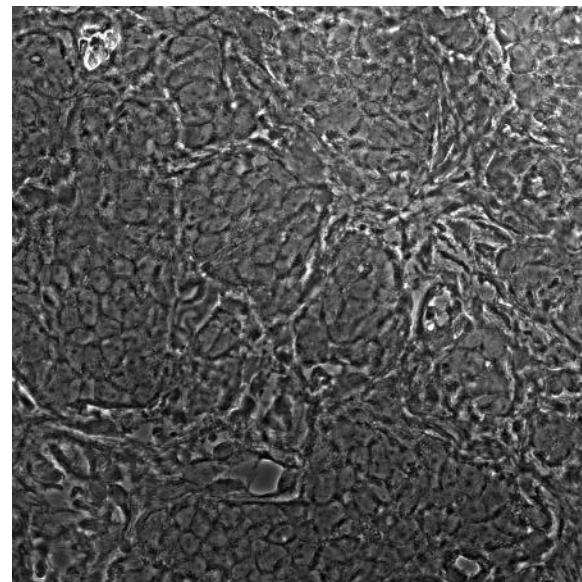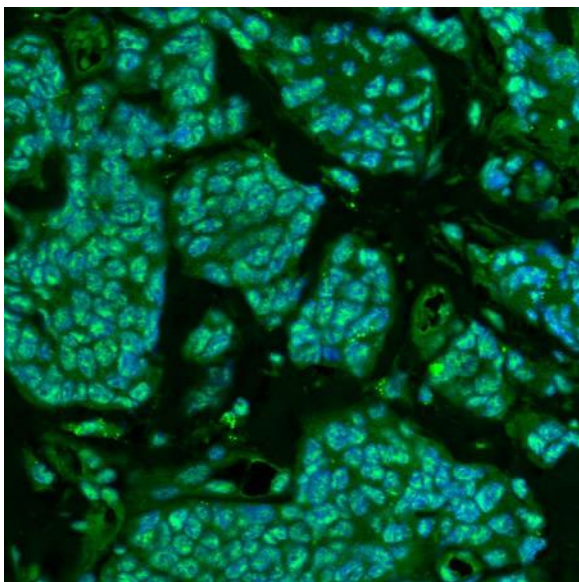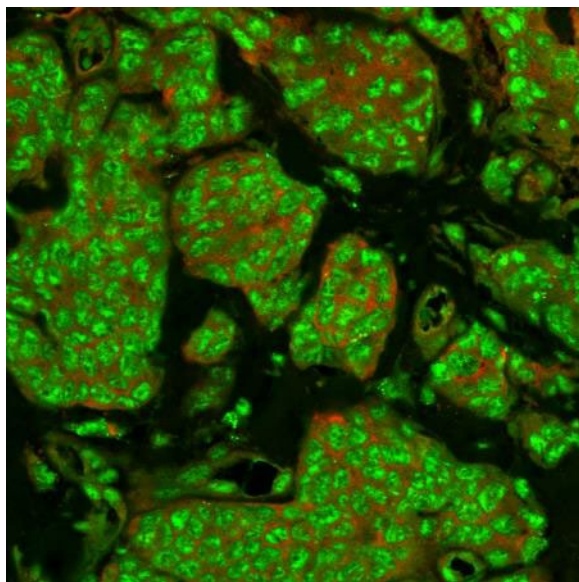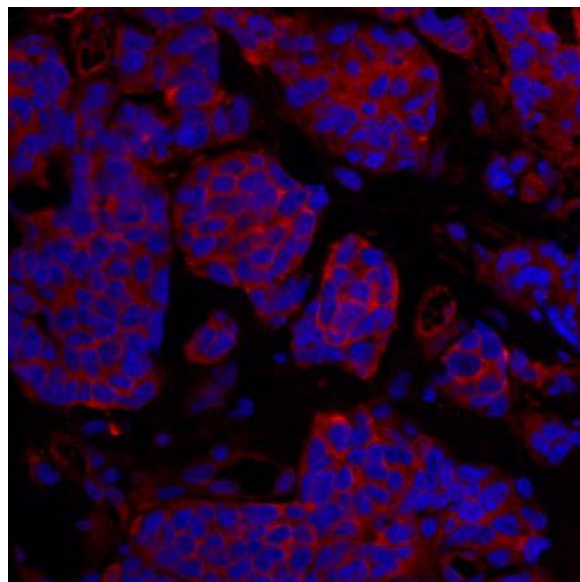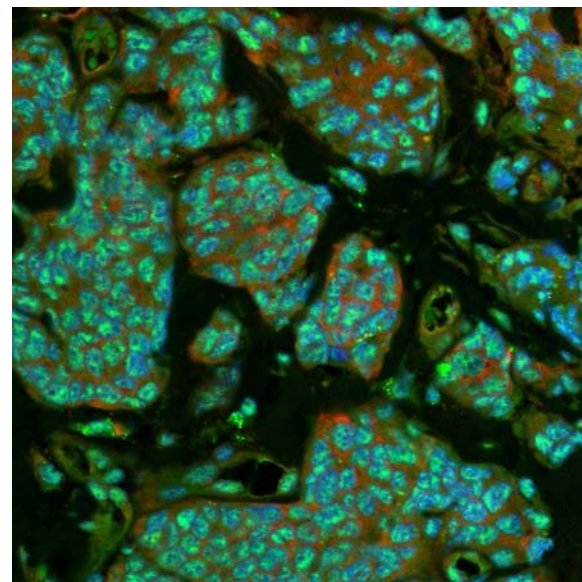

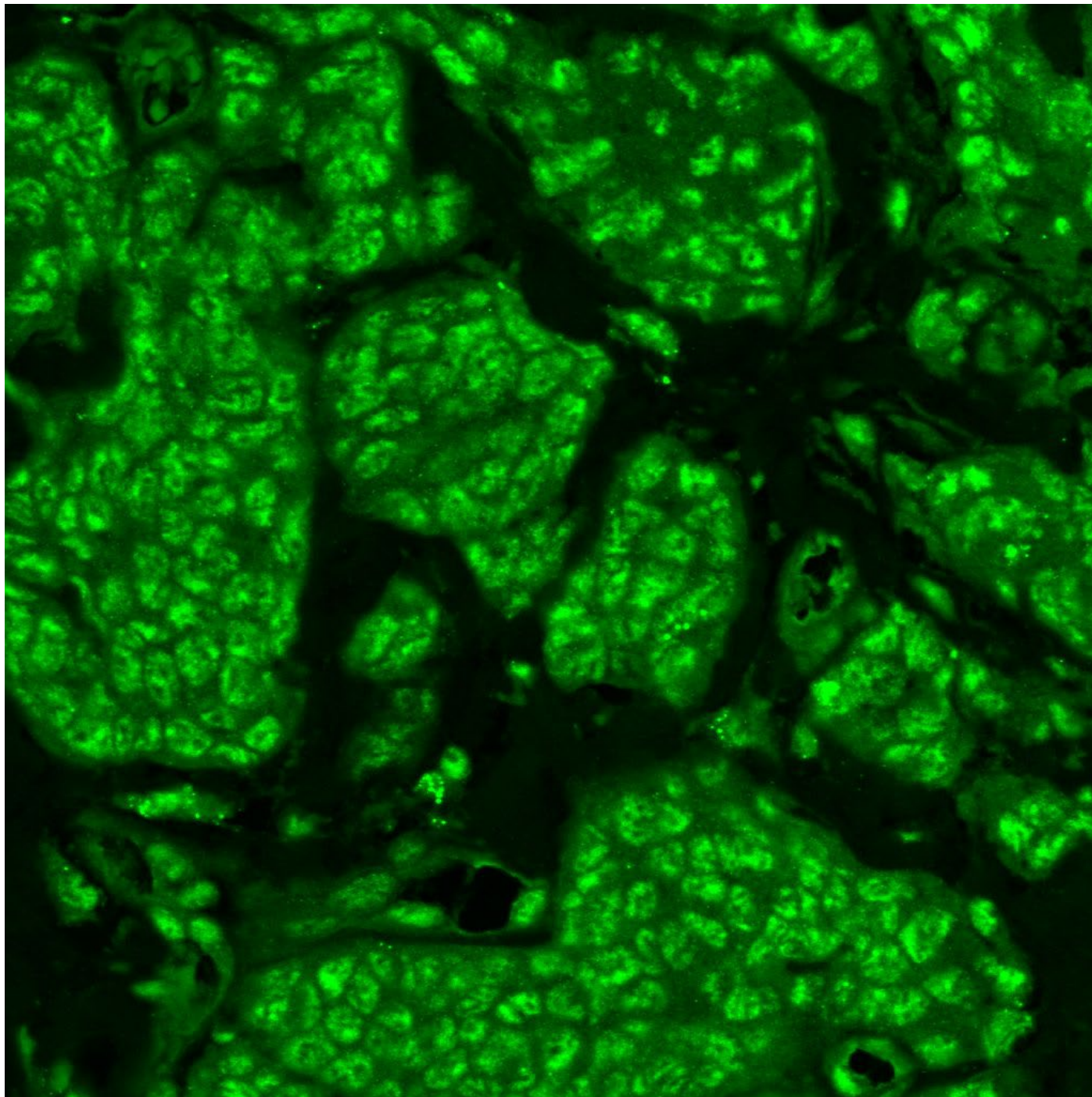

21825\_04

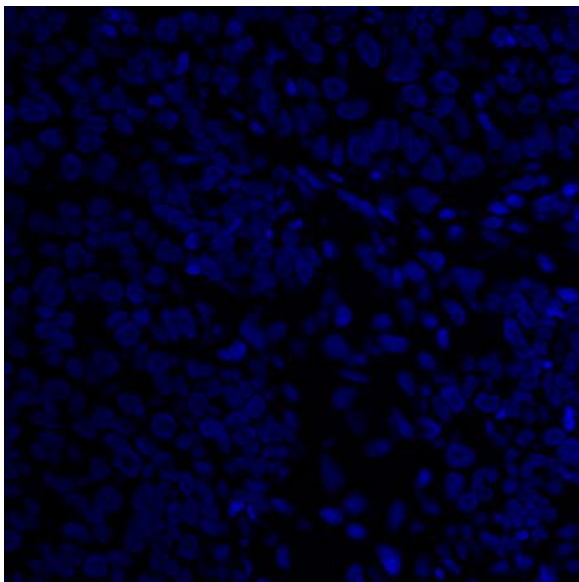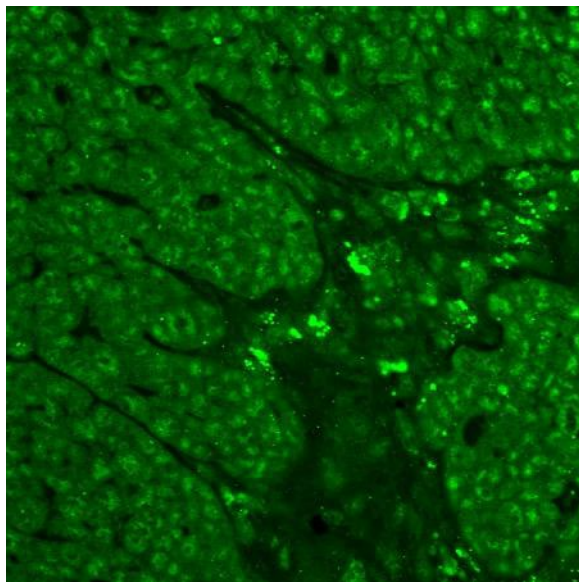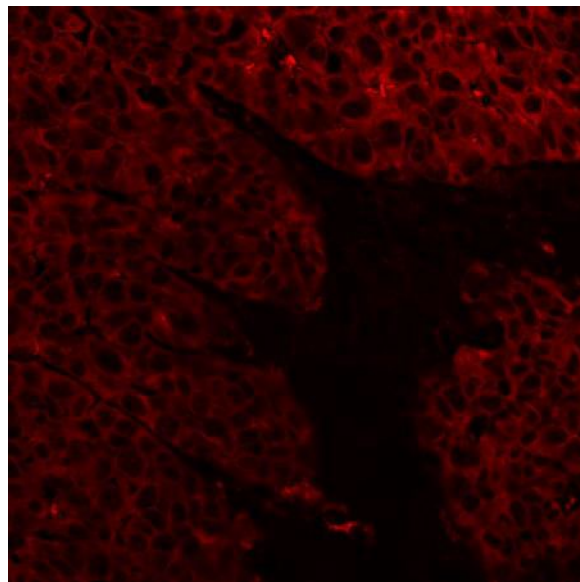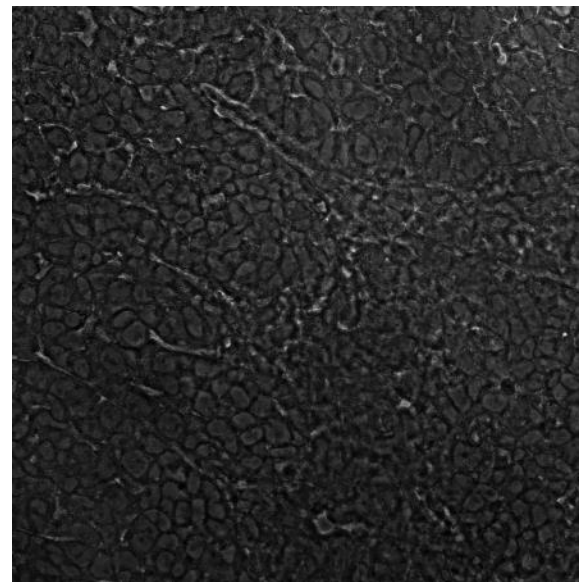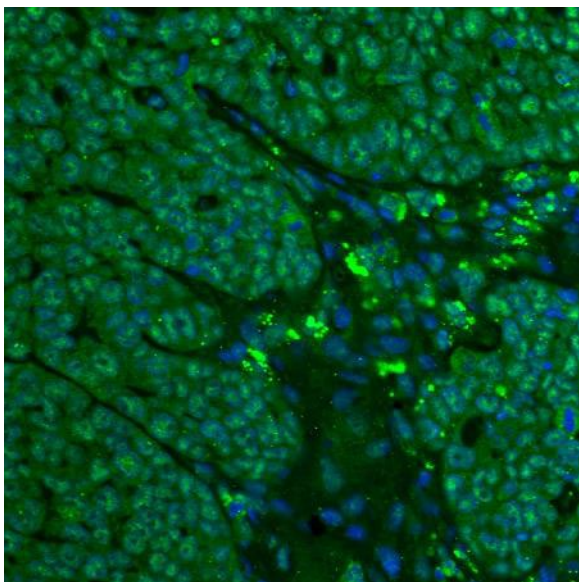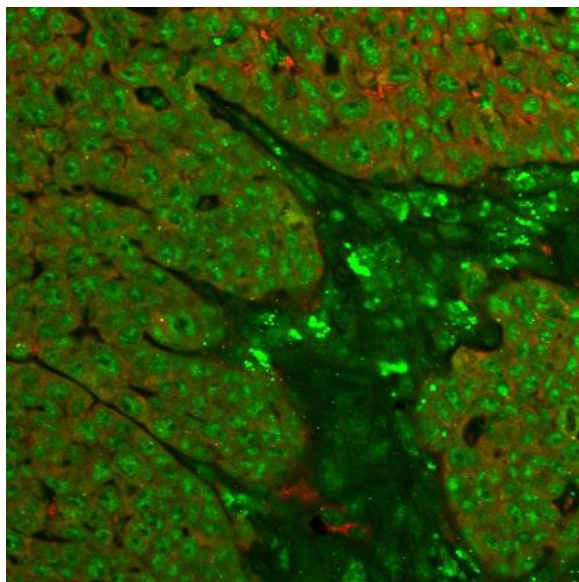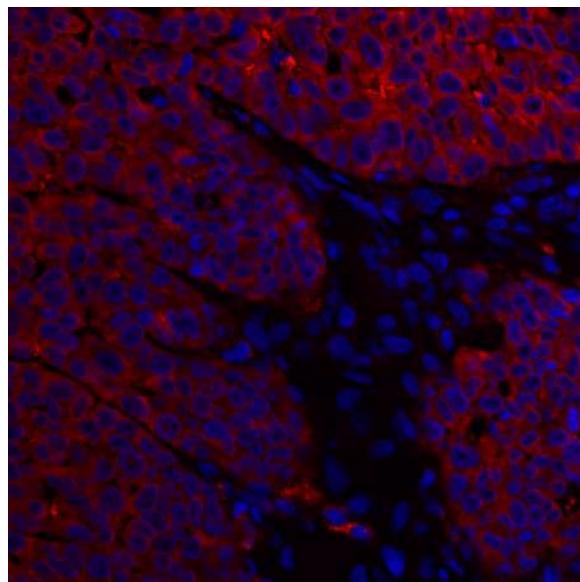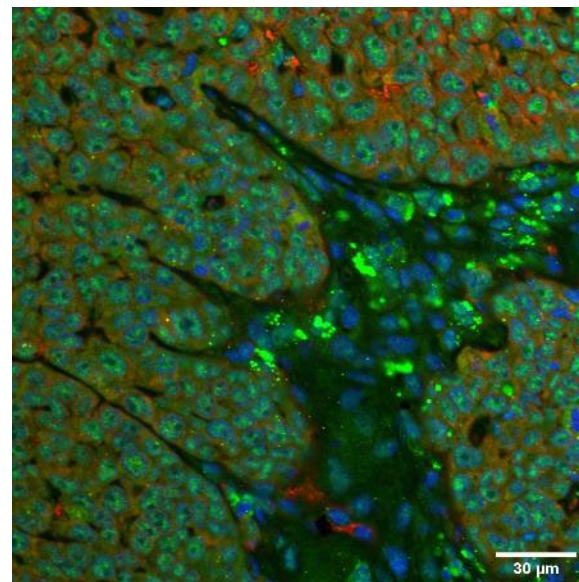

22316\_00

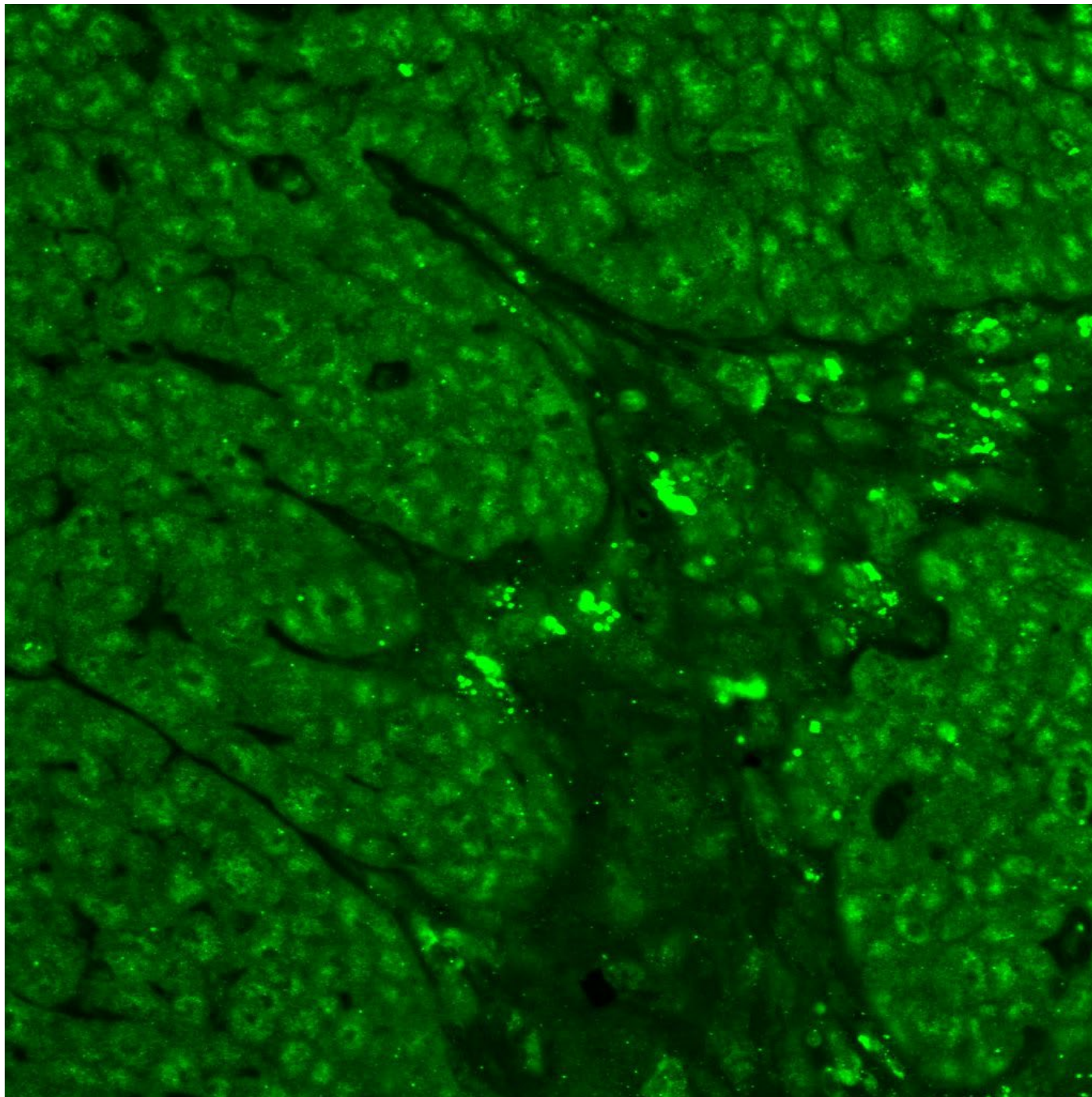

22316\_00

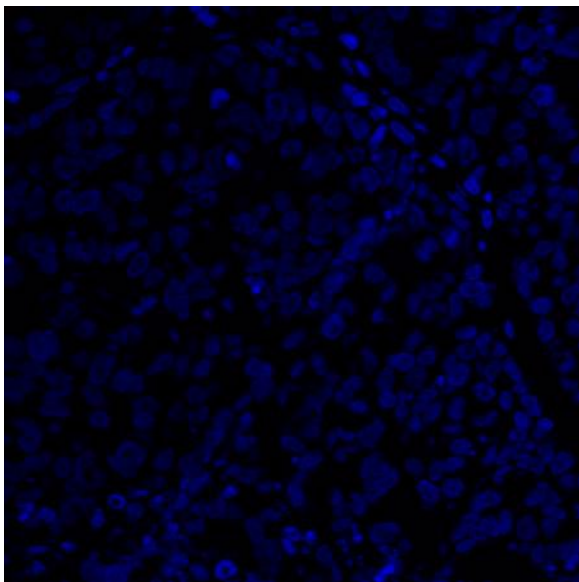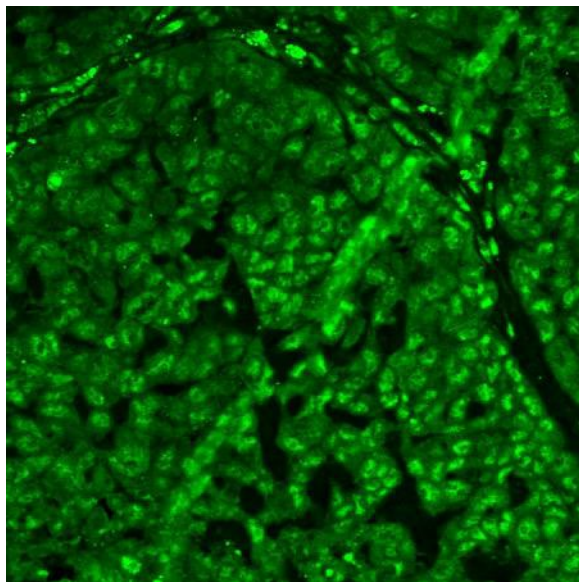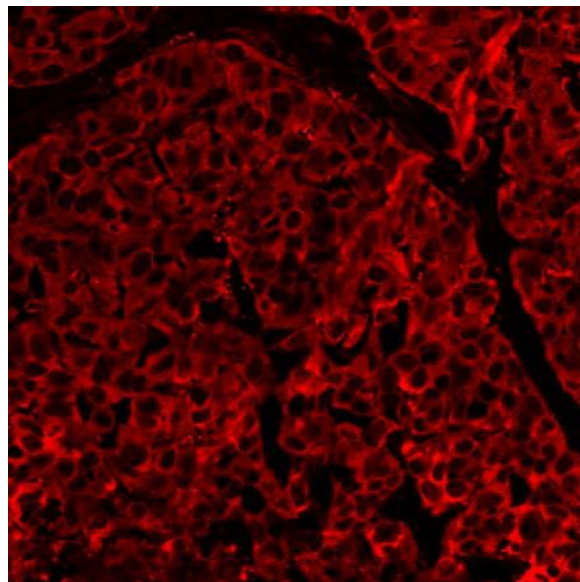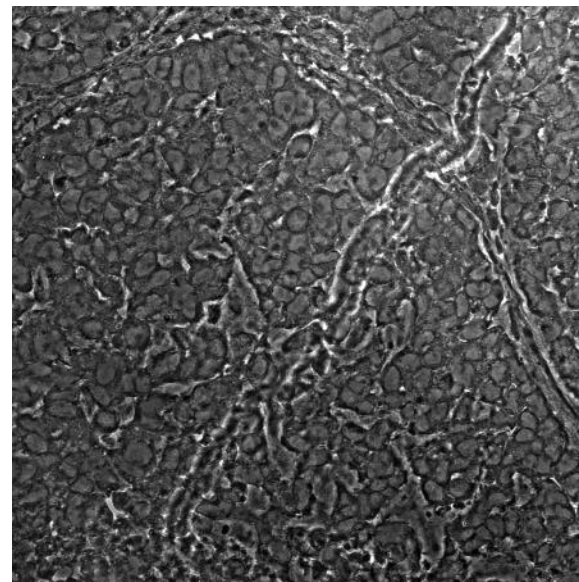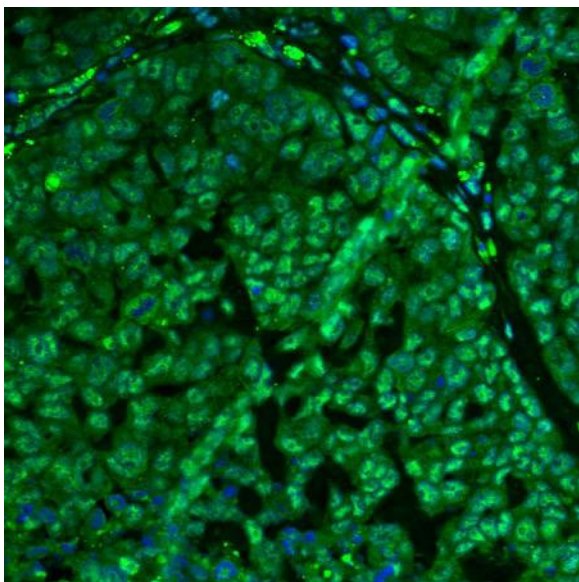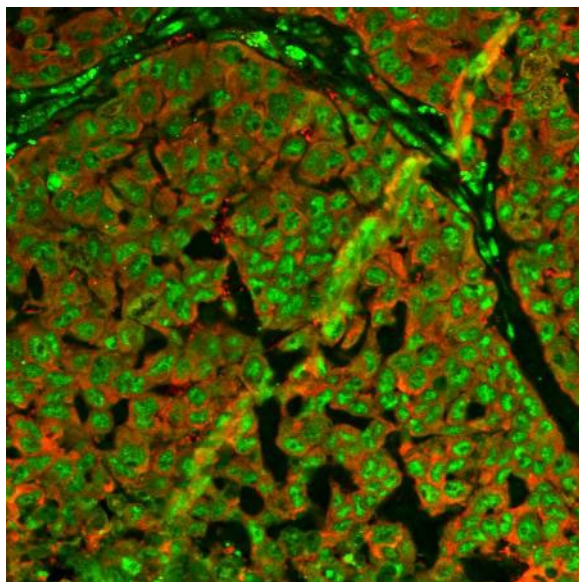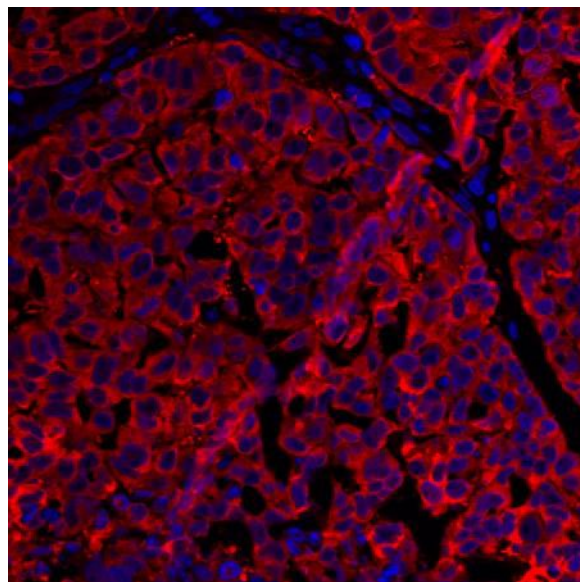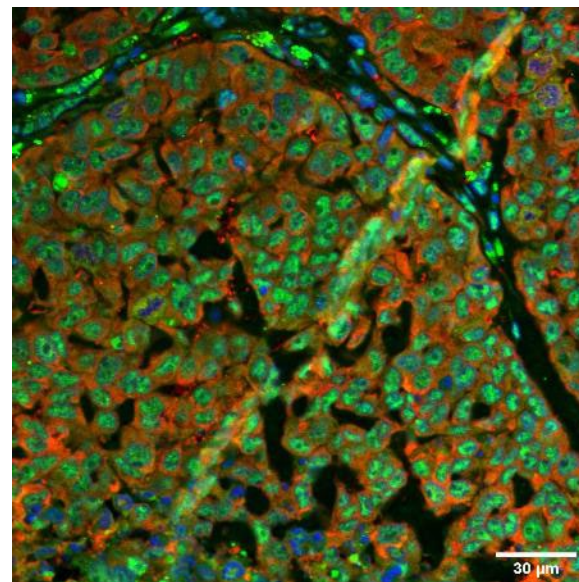

22316\_01

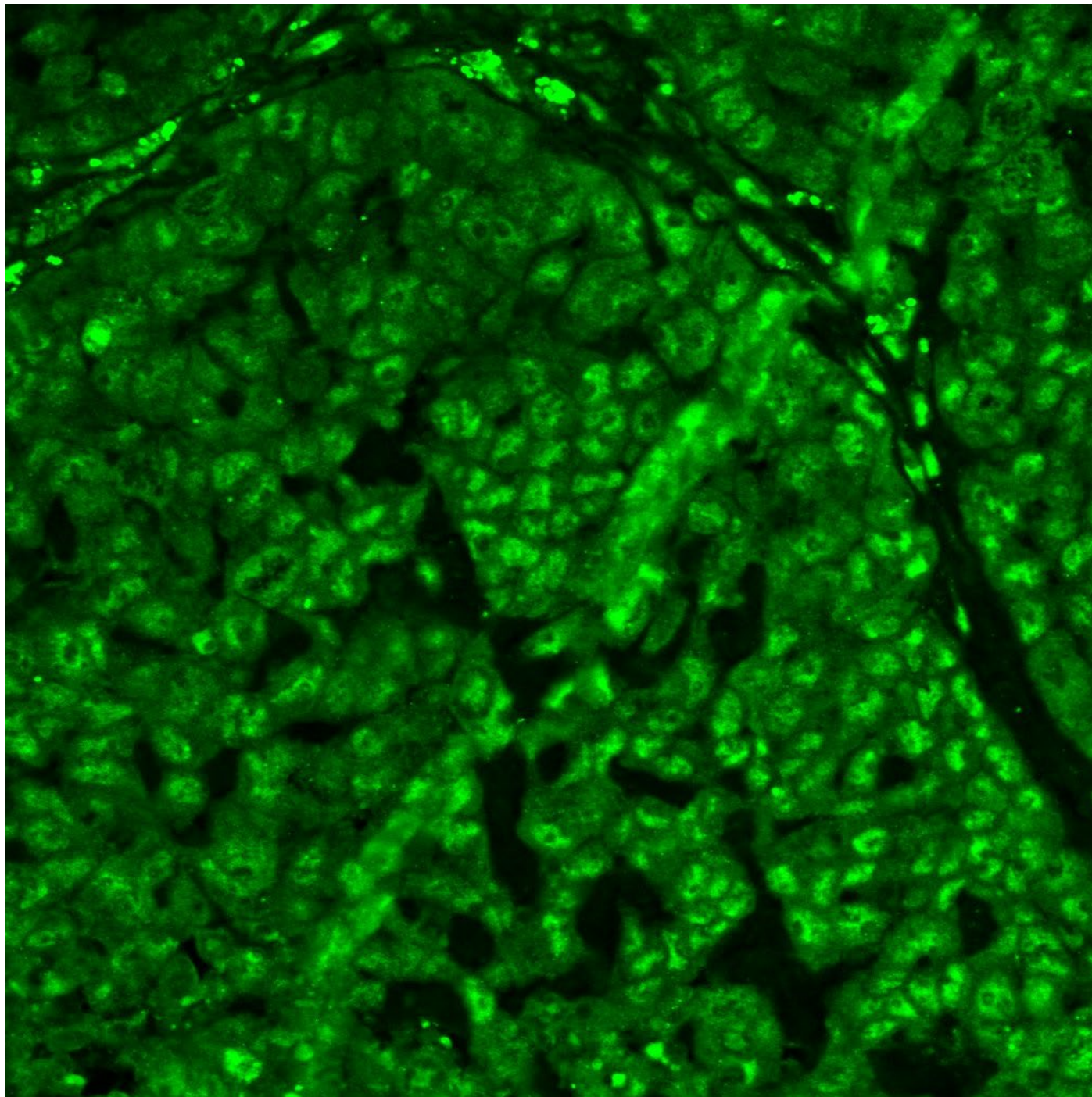

22316\_01

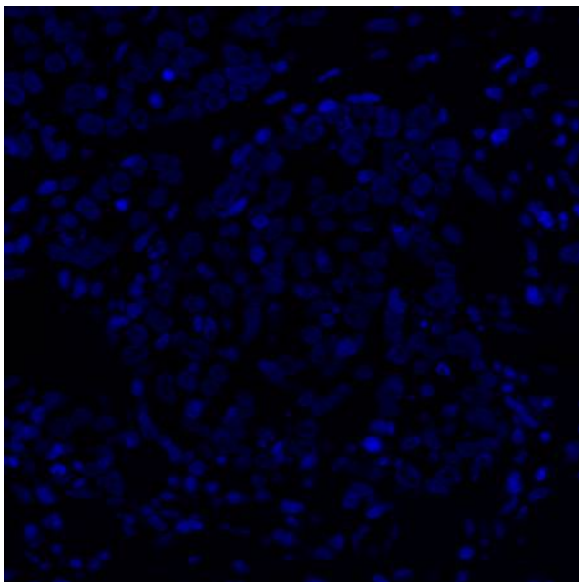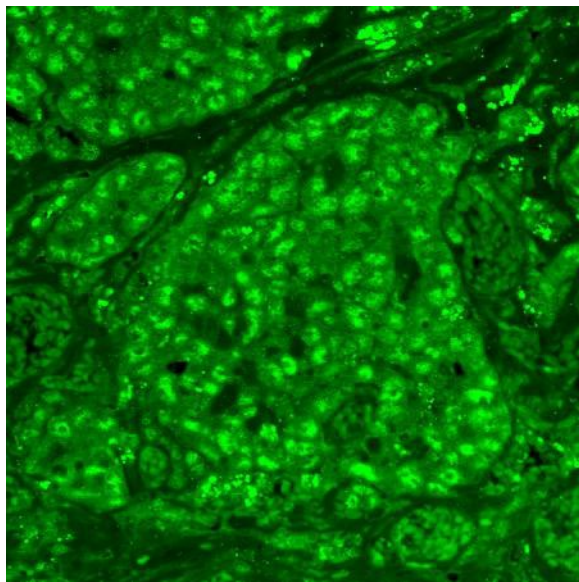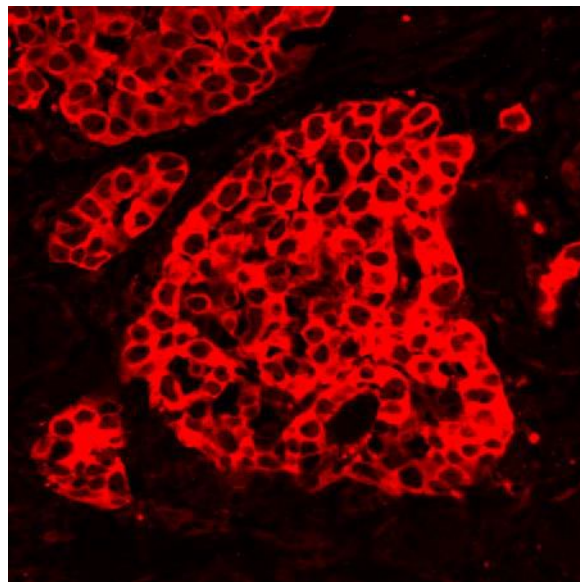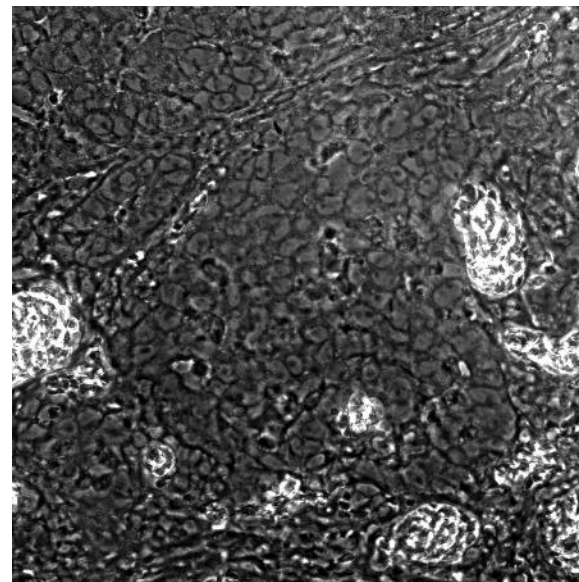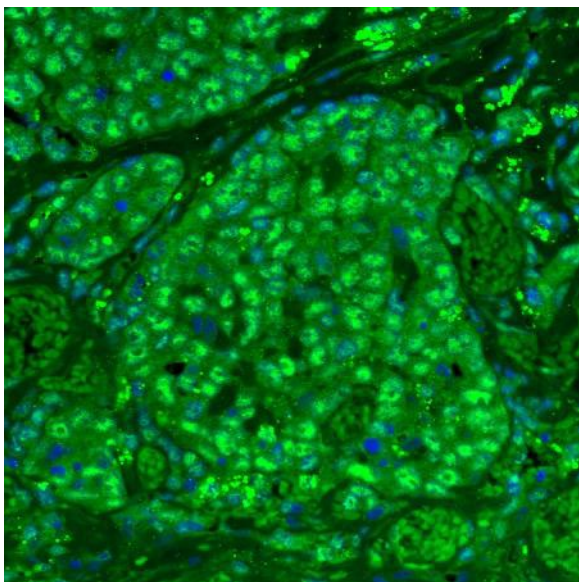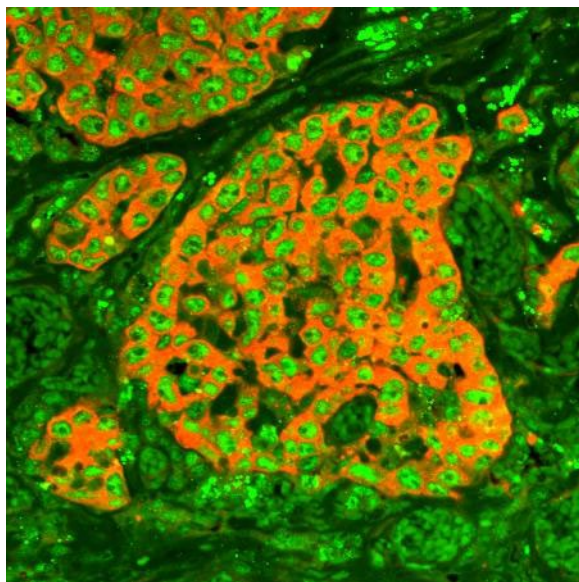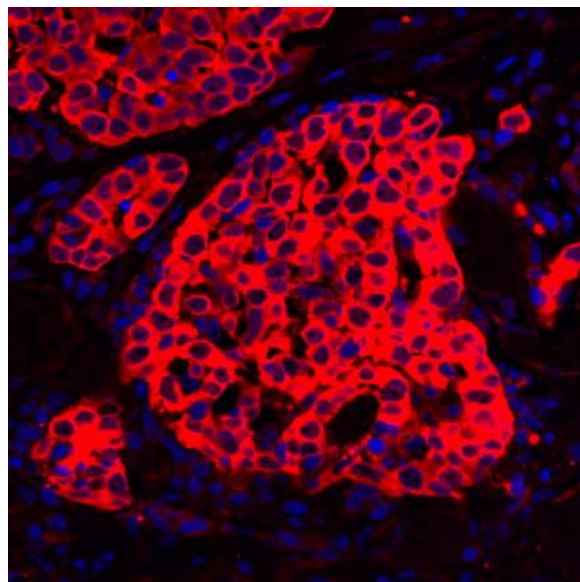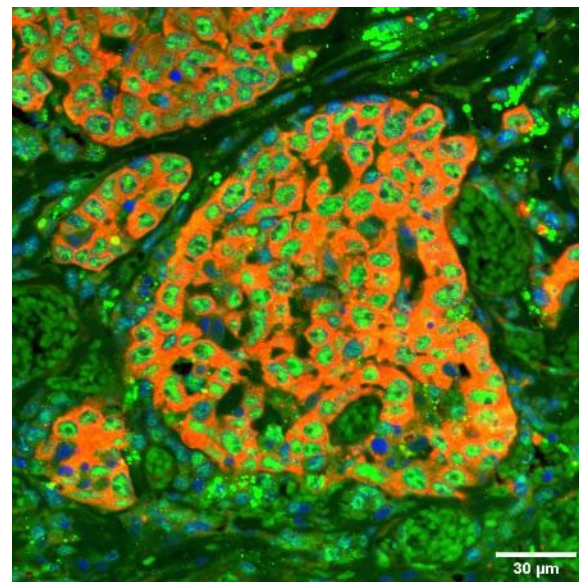

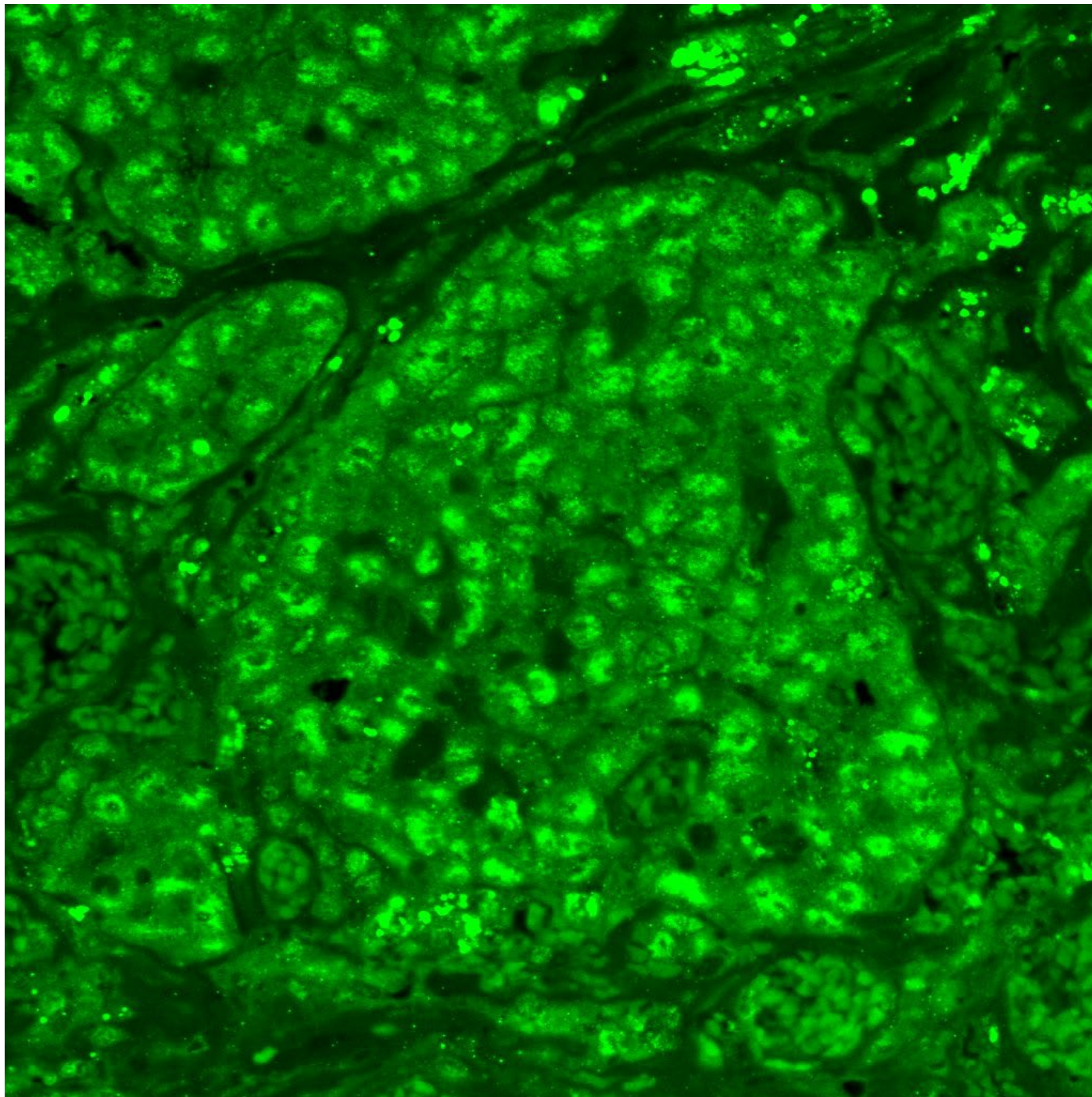

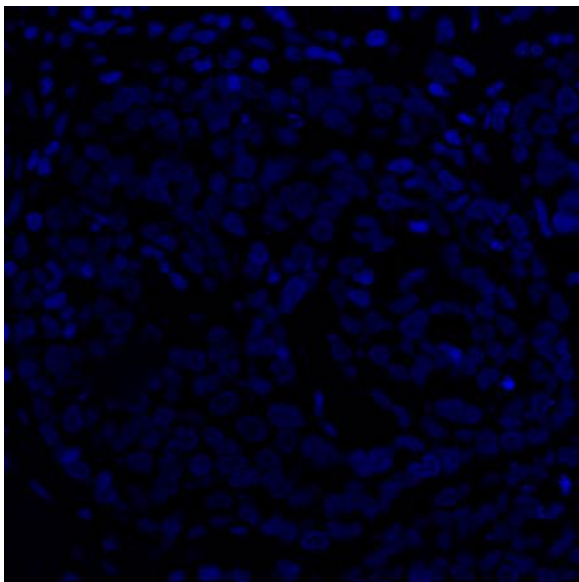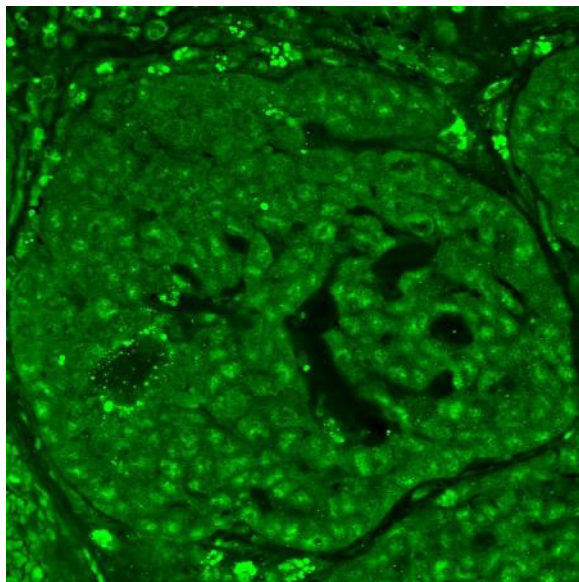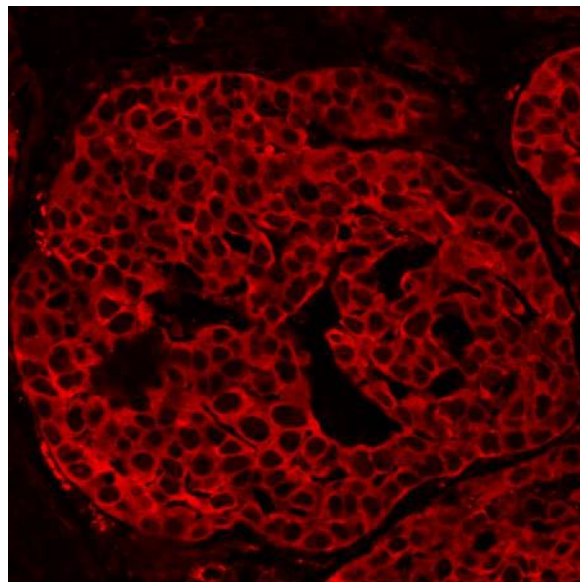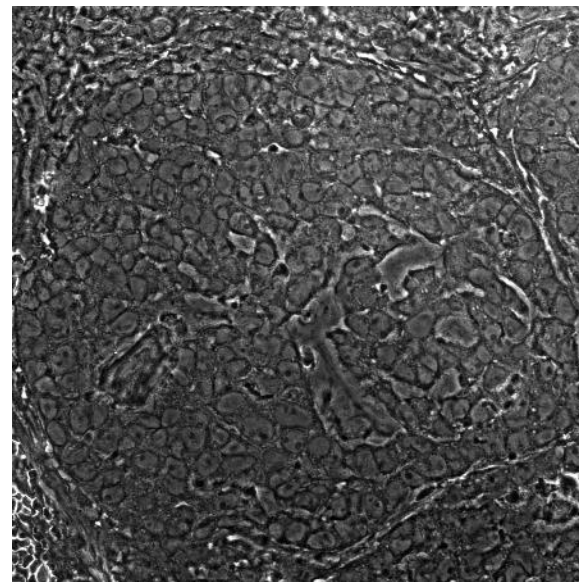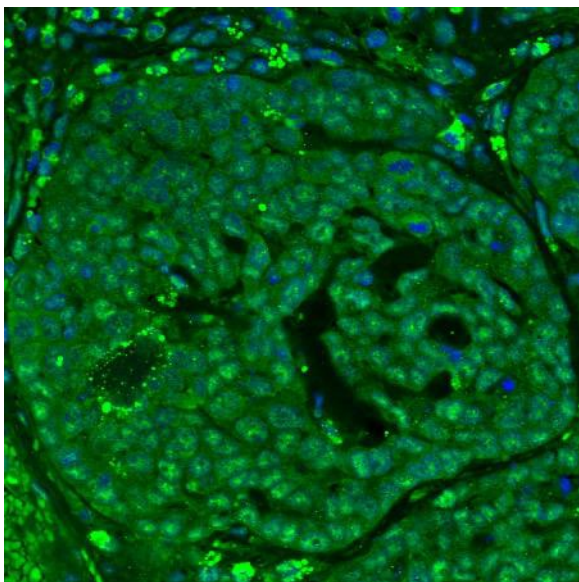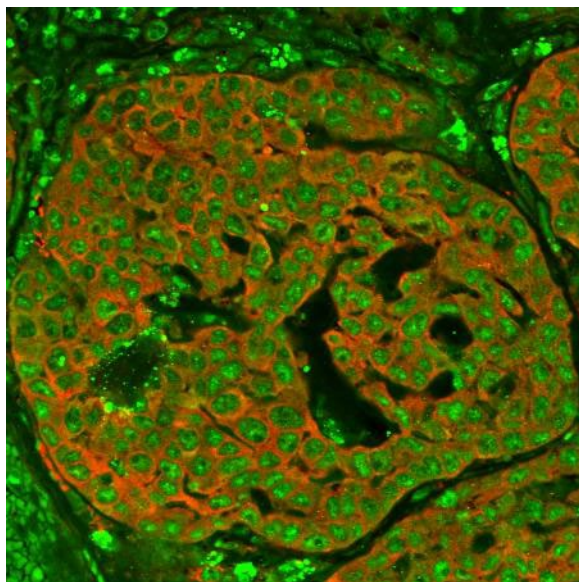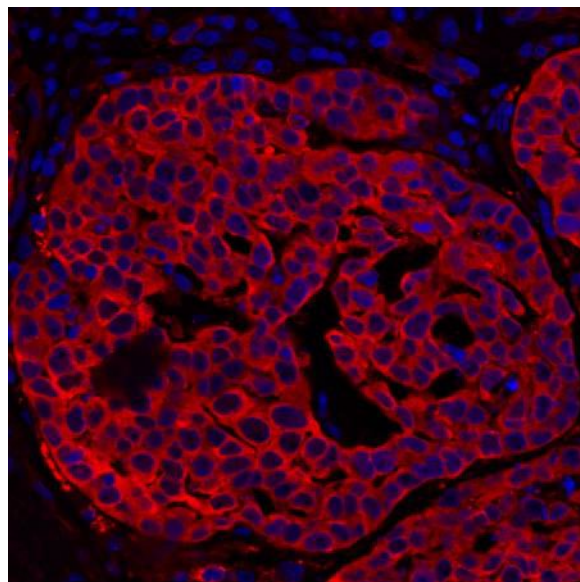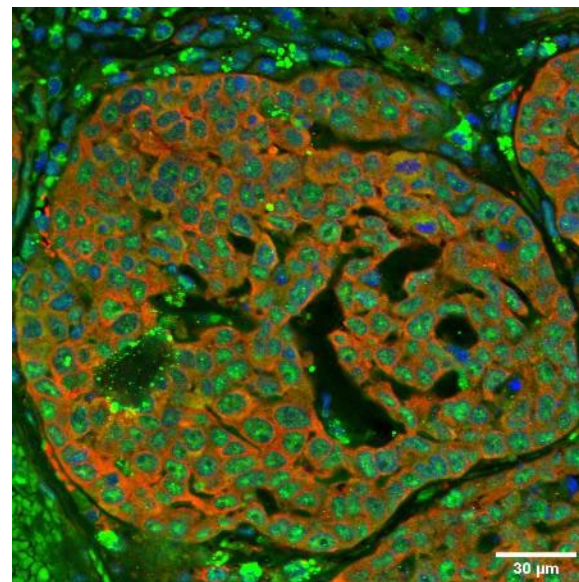

22316\_03

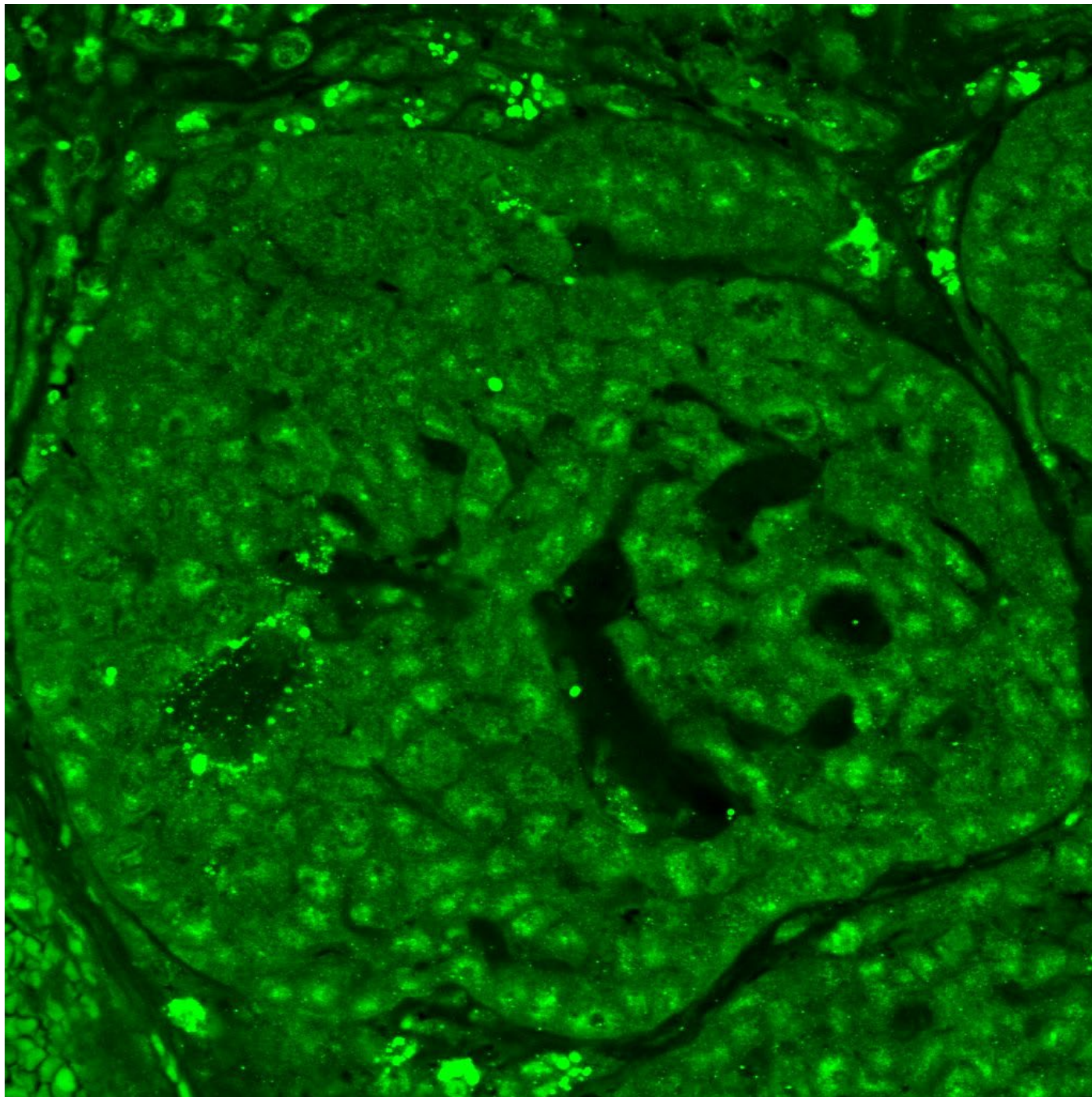

22316\_03

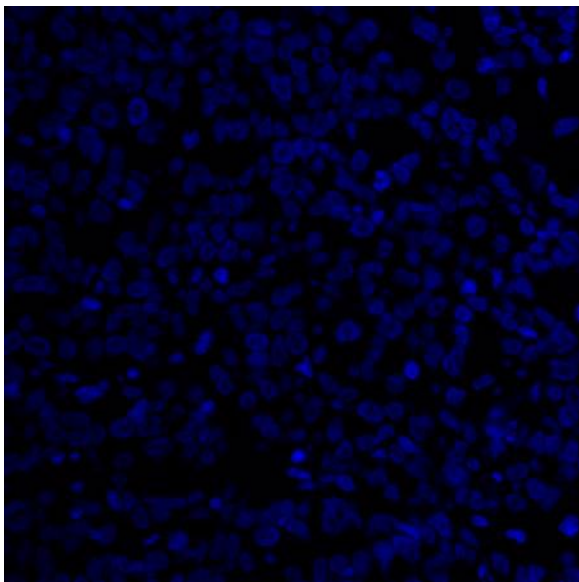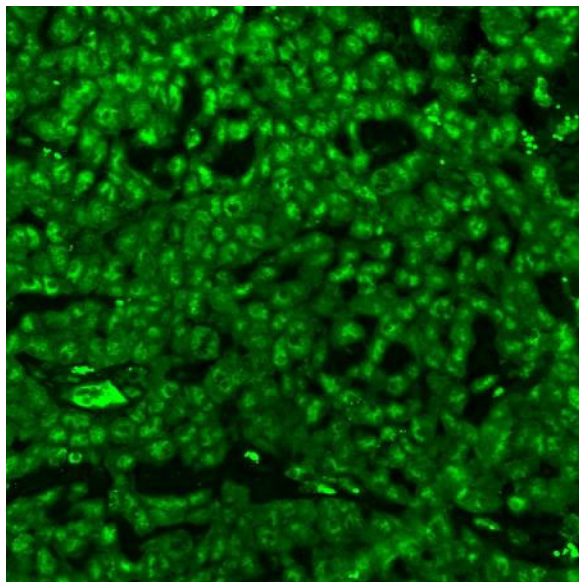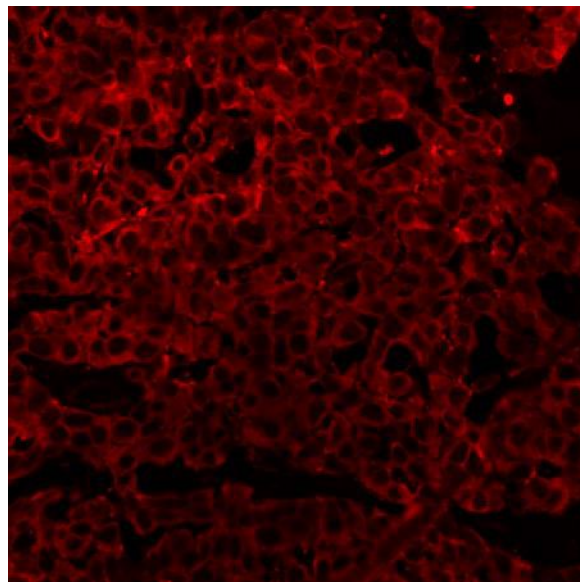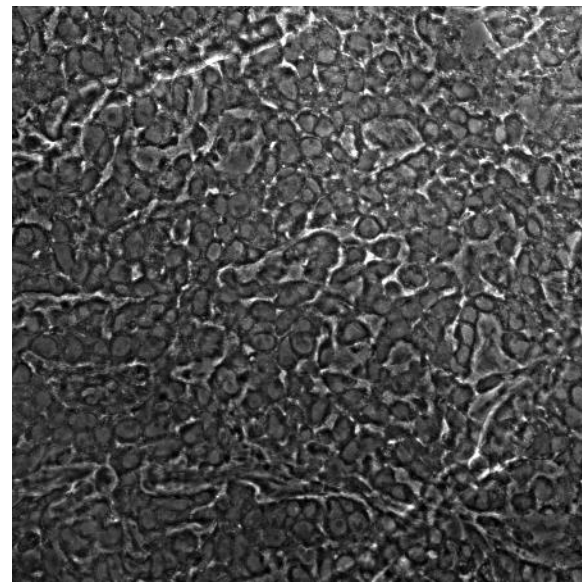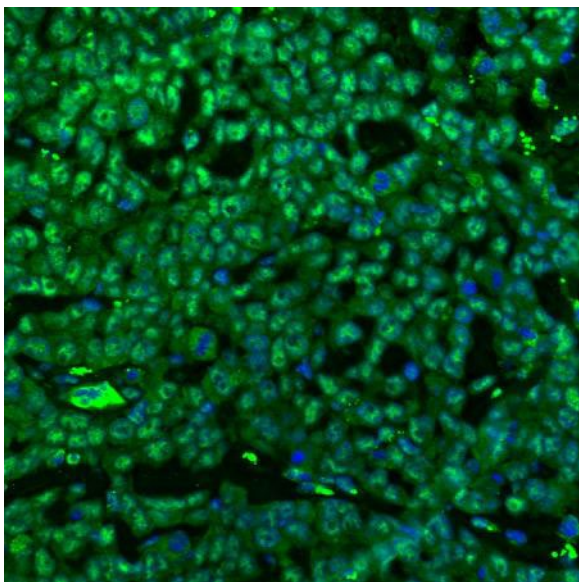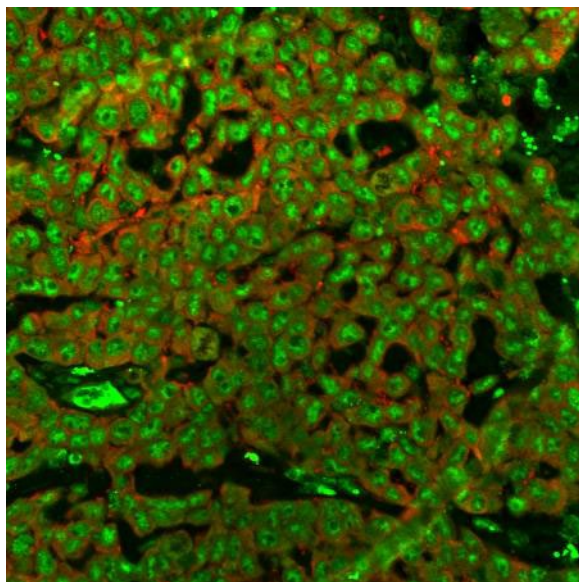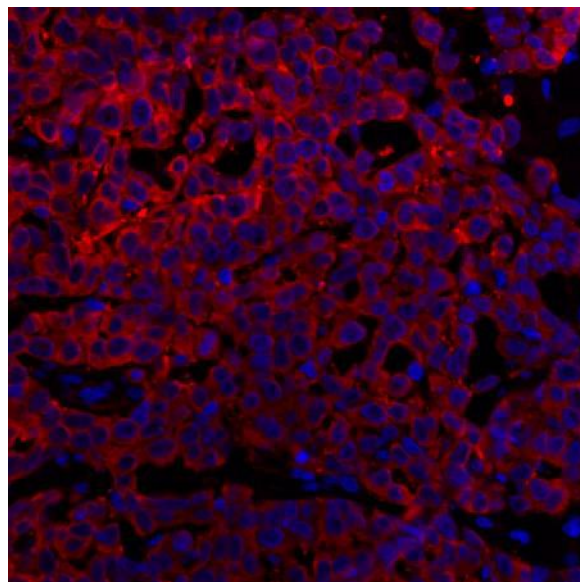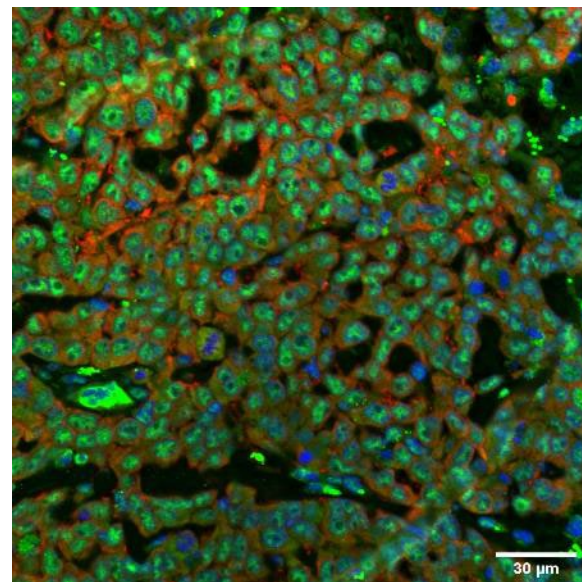

22316\_04

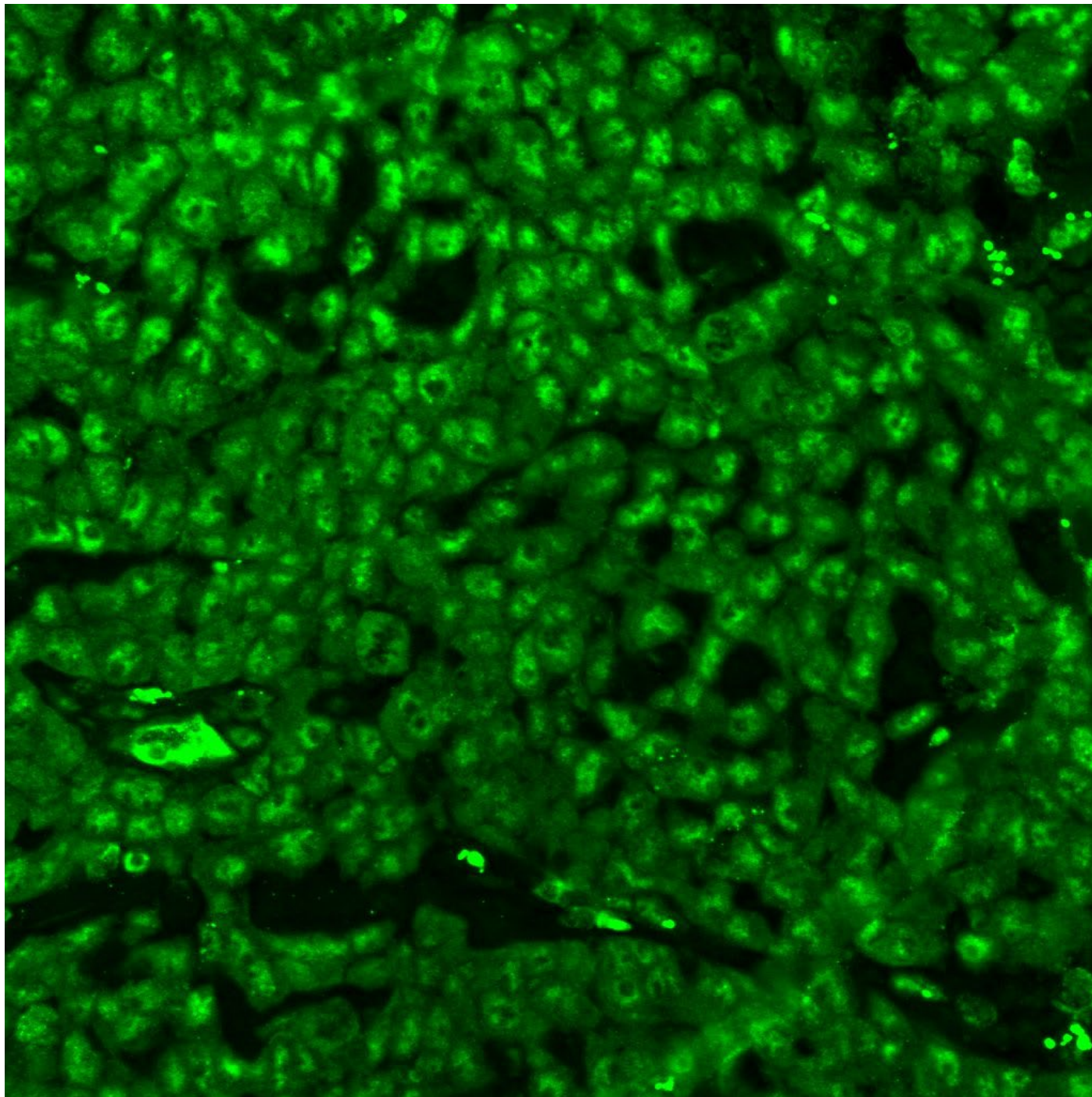

22316\_04

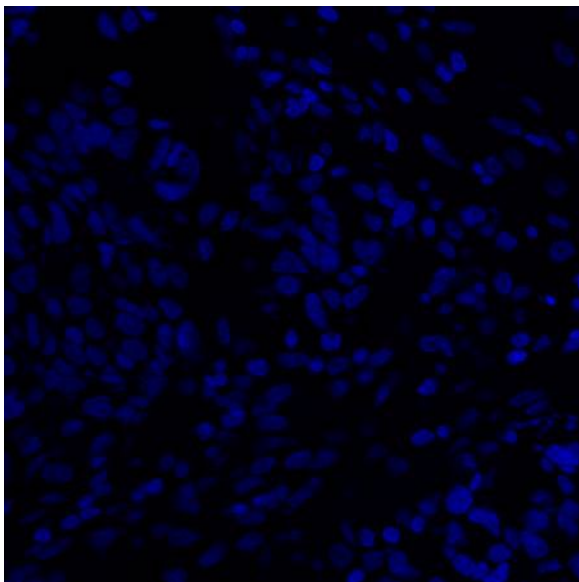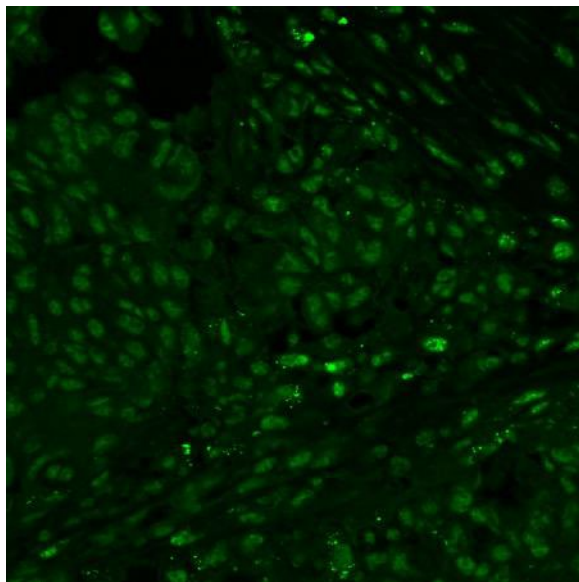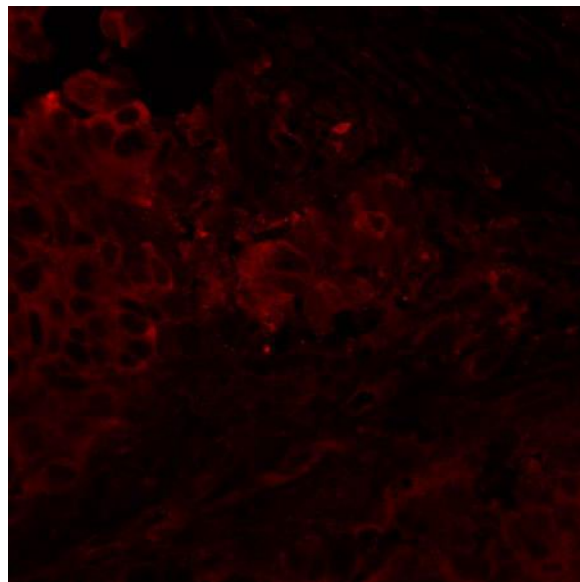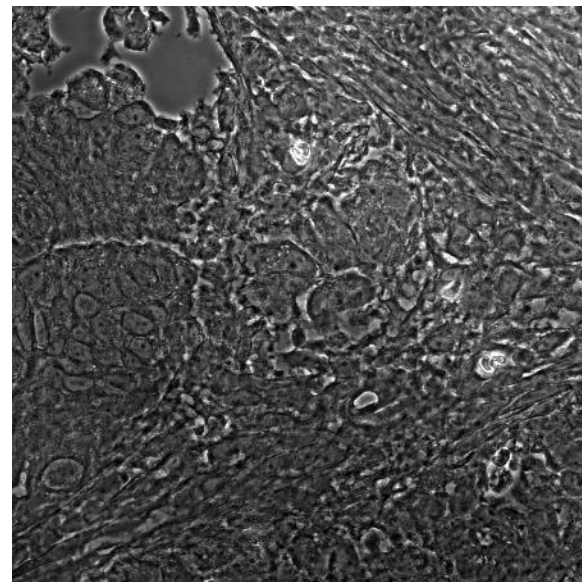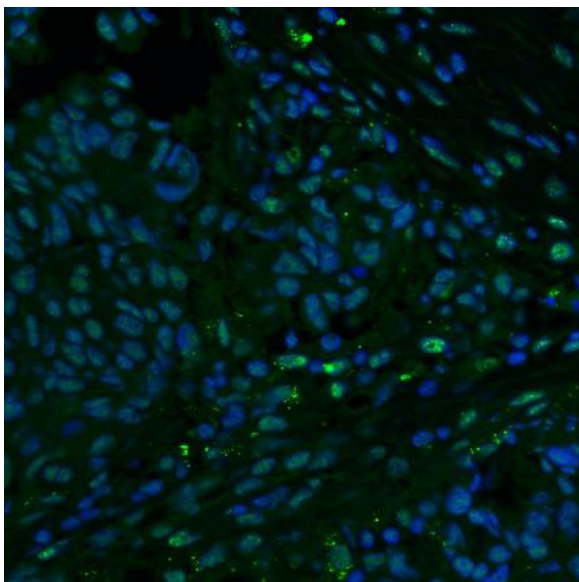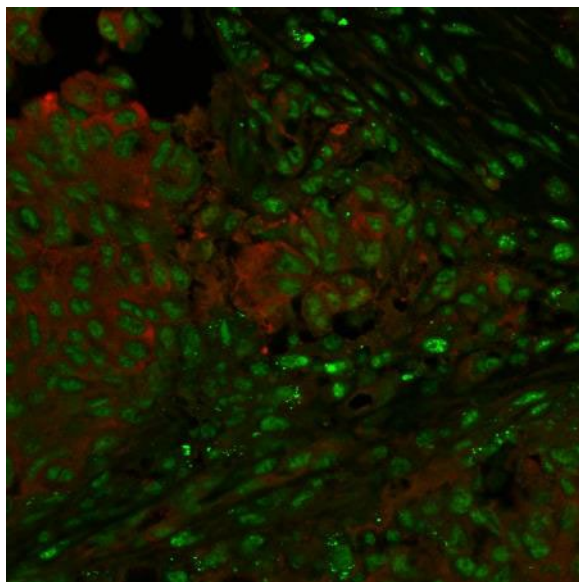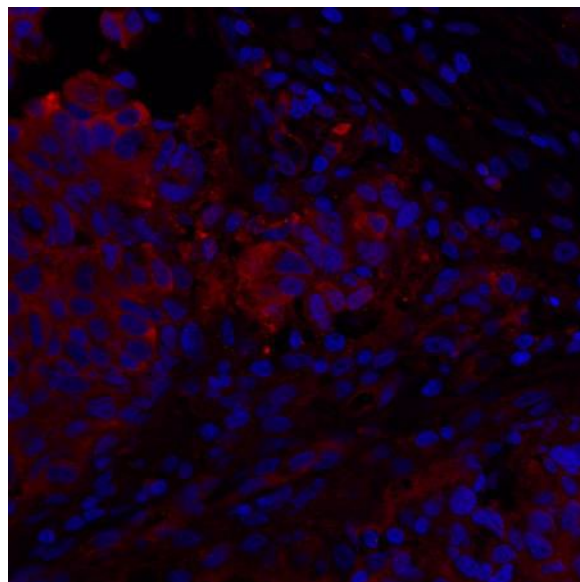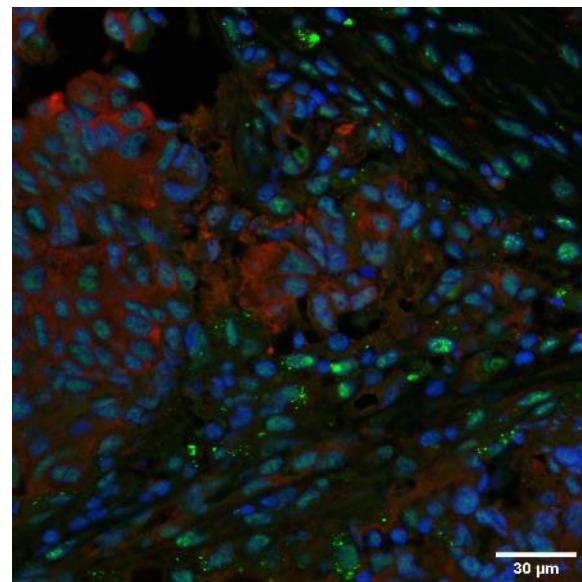

22376\_00

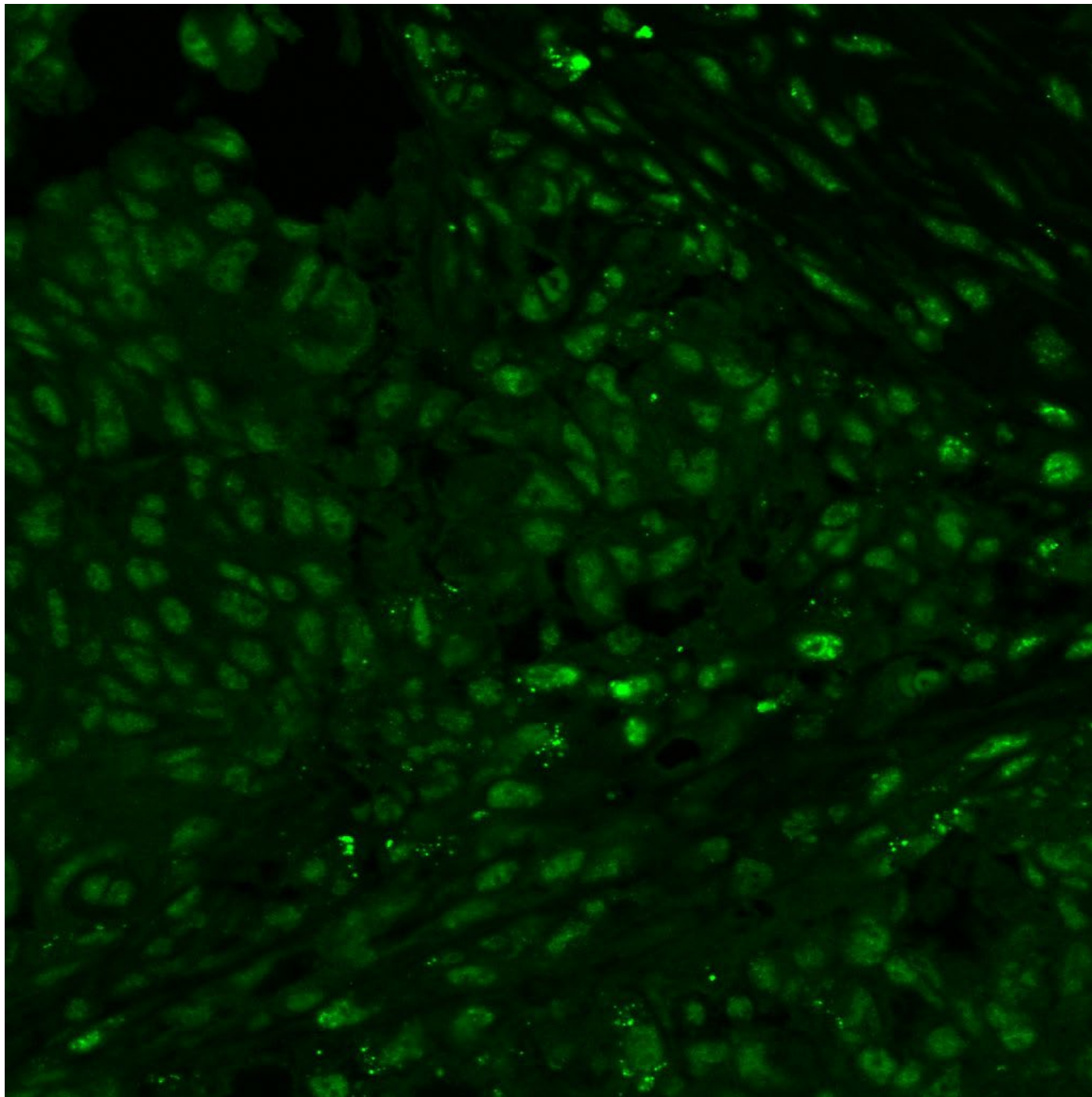

22376\_00

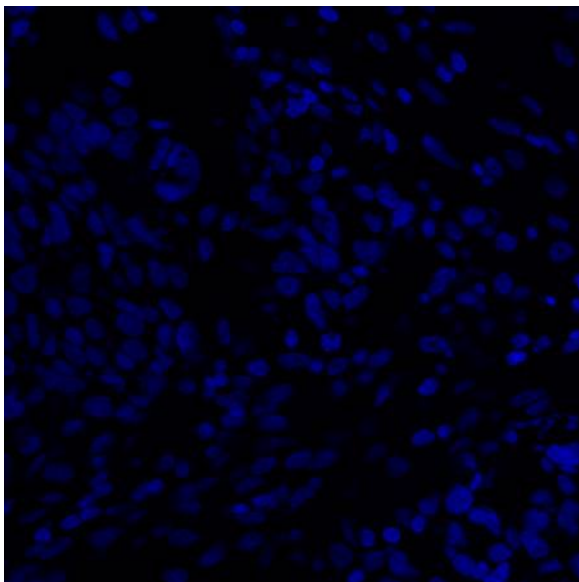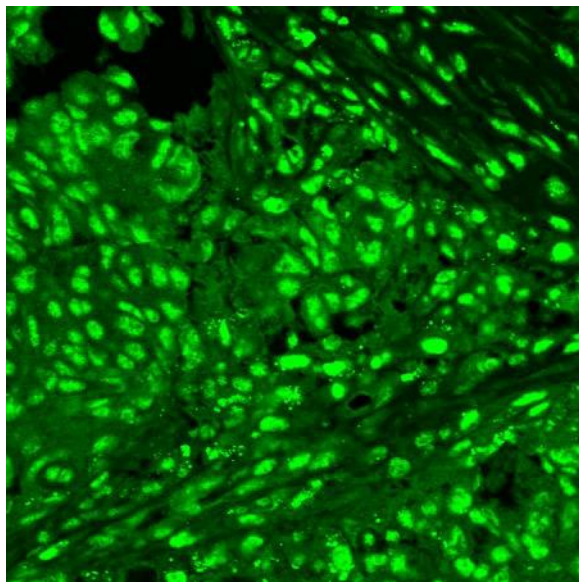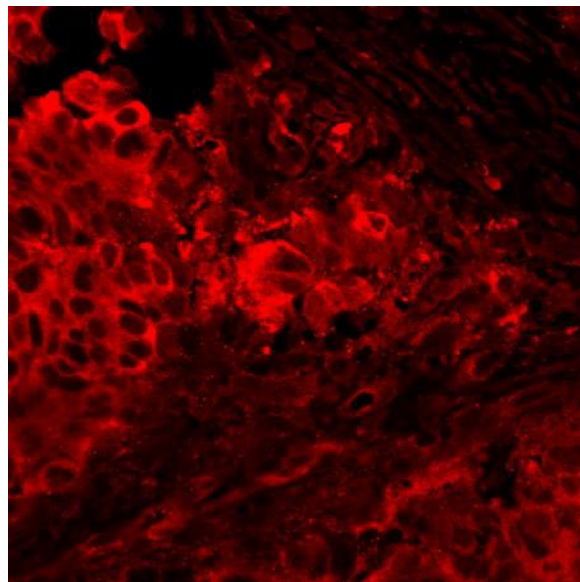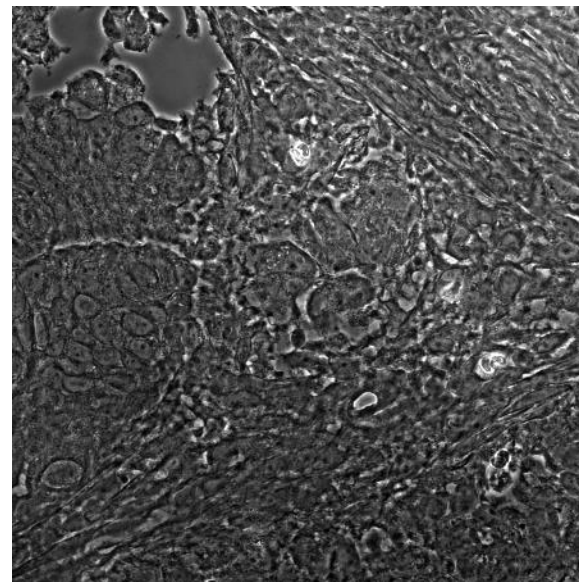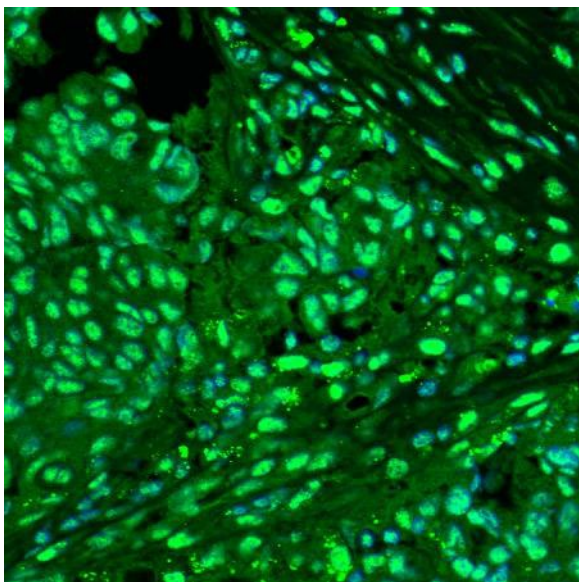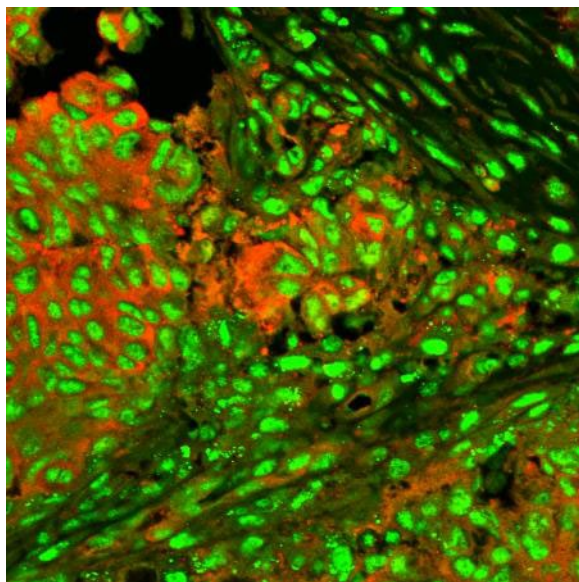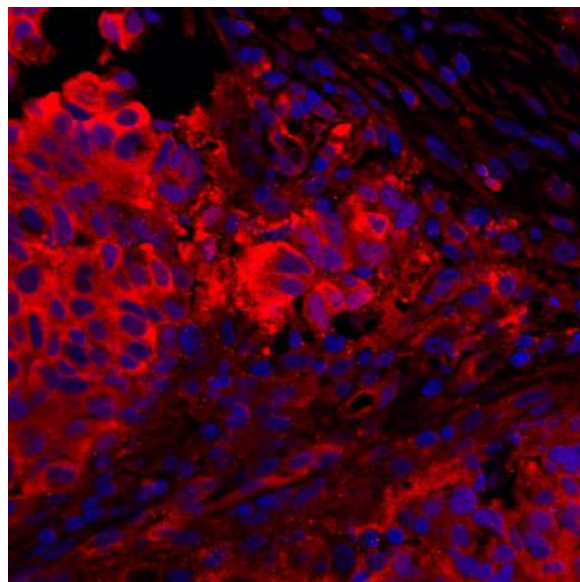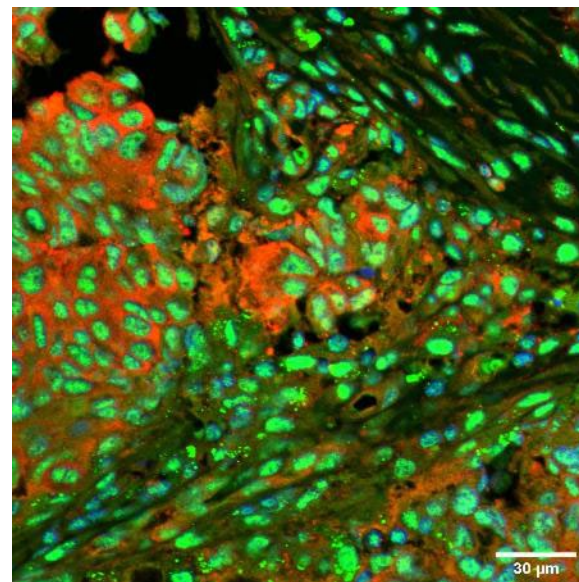

22376\_01

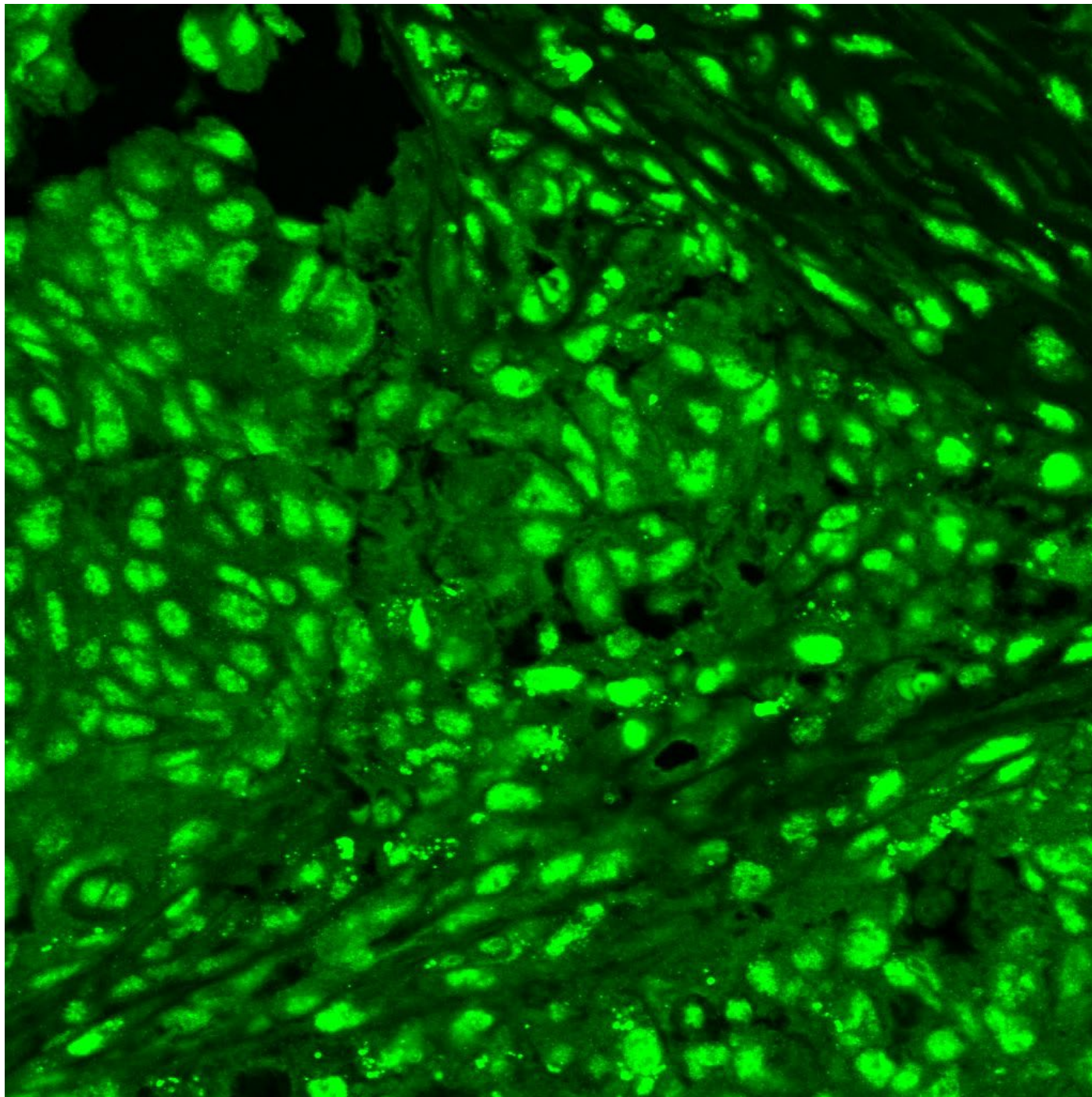

22376\_01

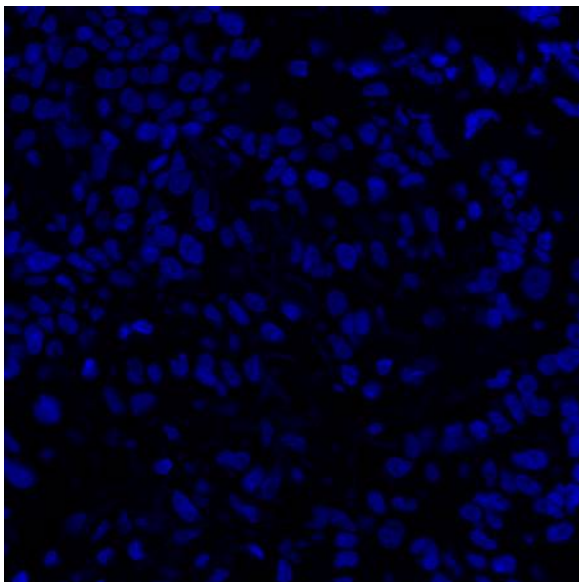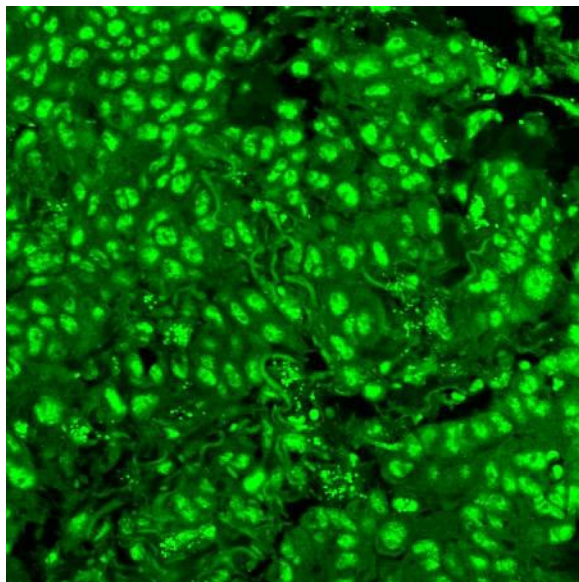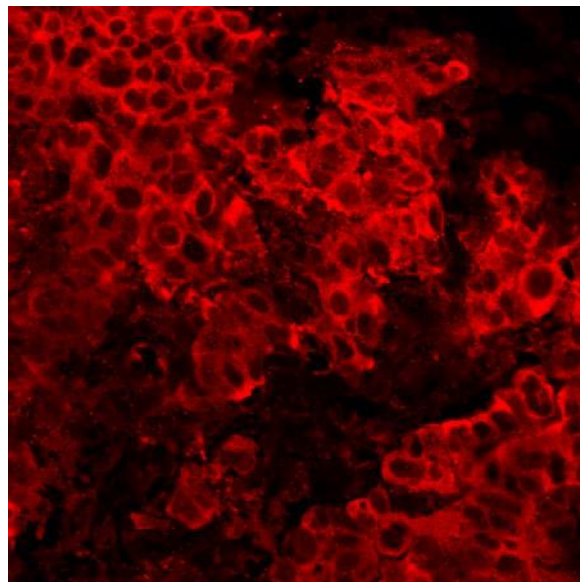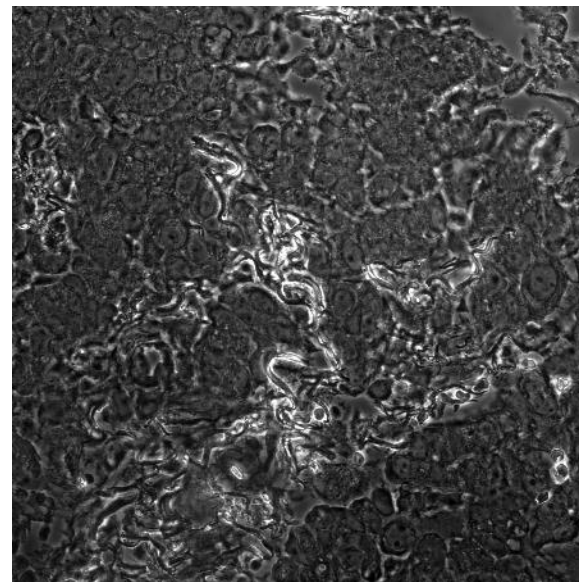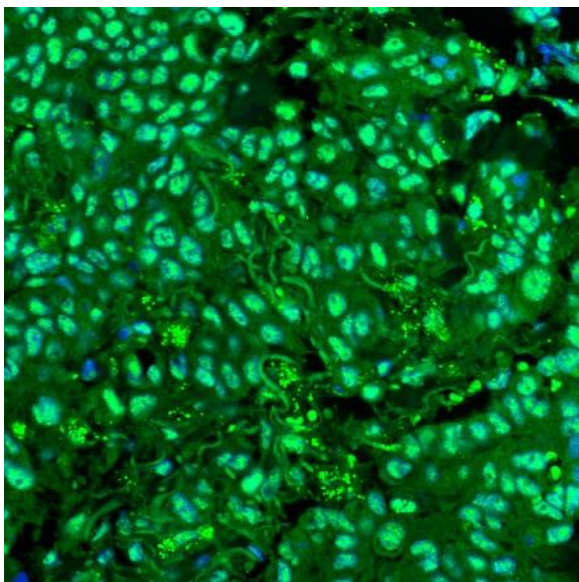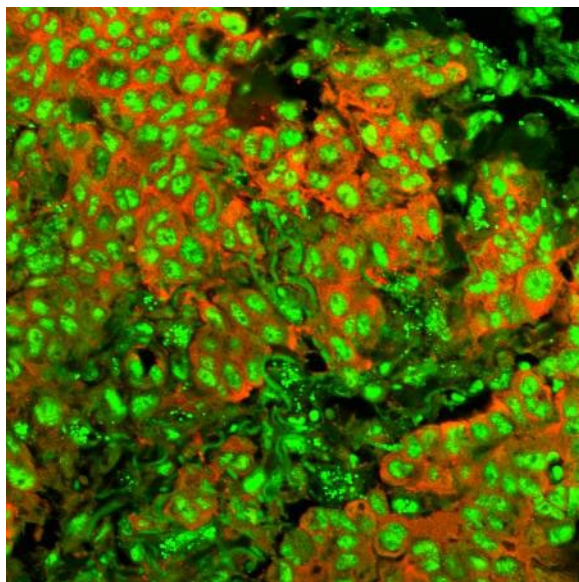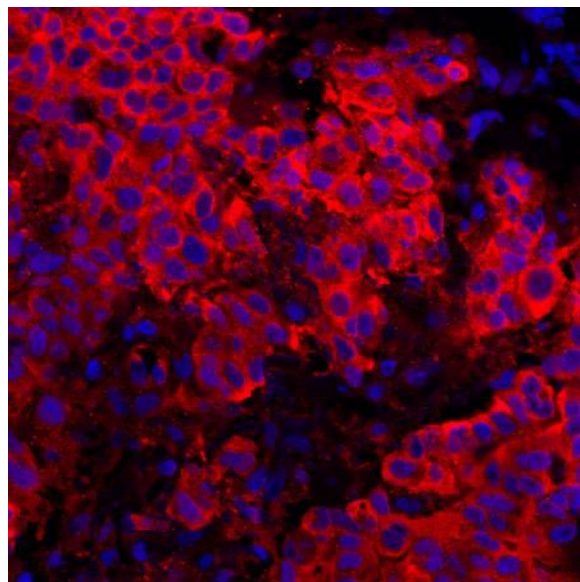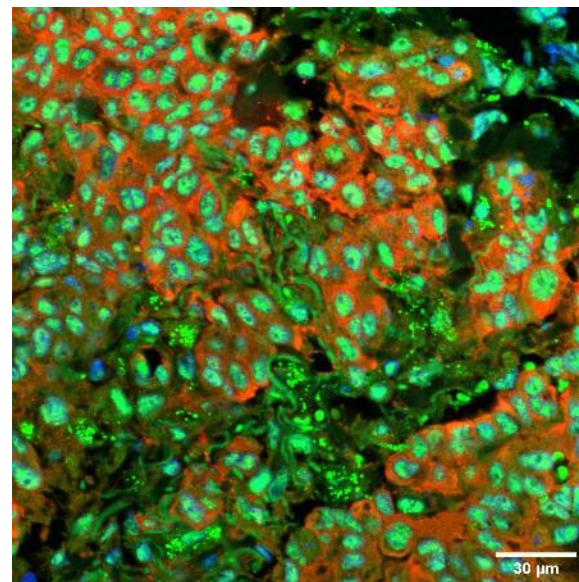

22376\_02

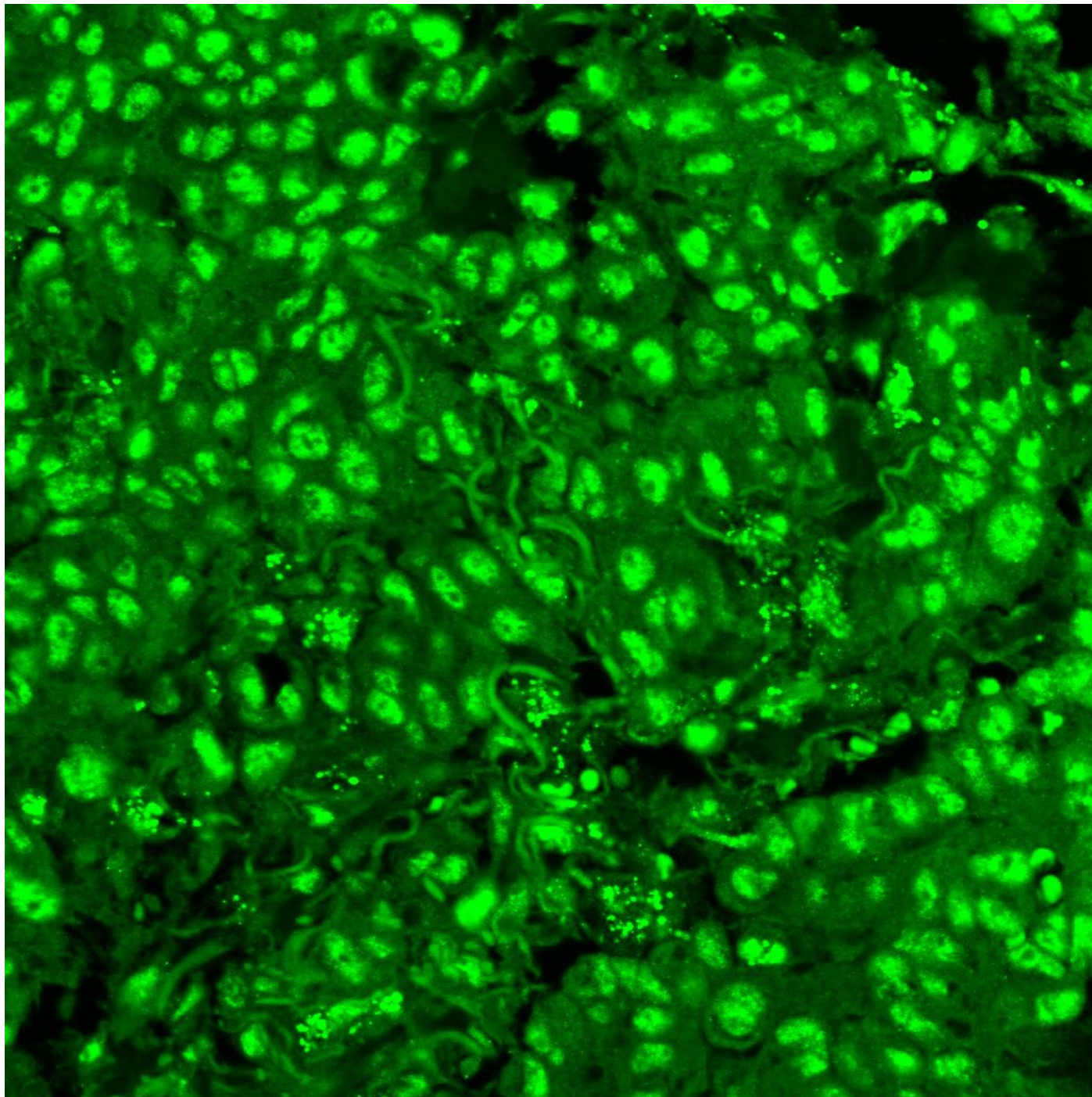

22376\_02

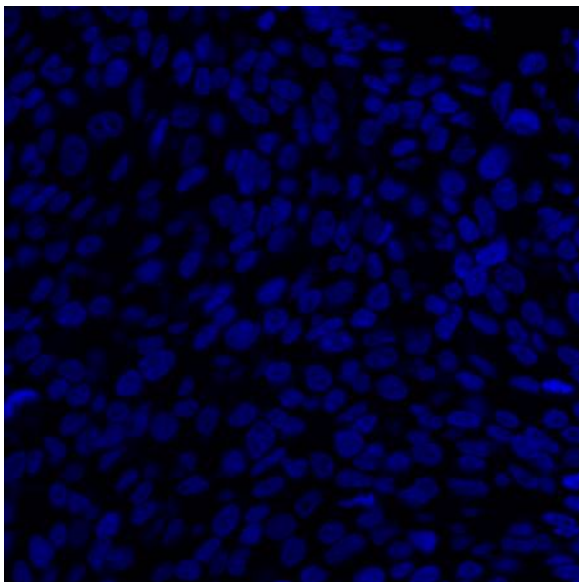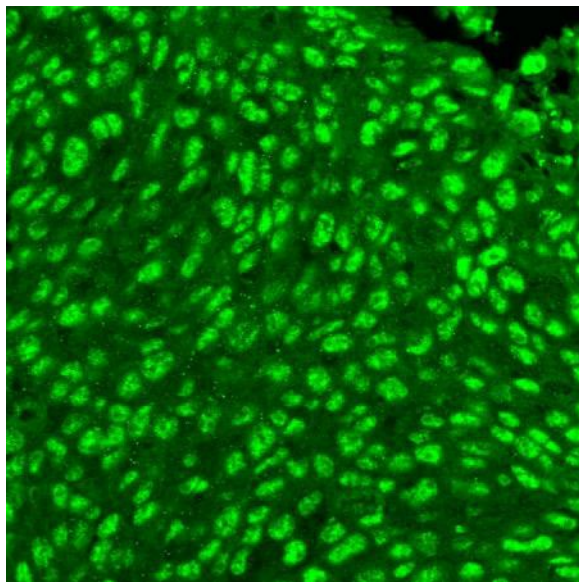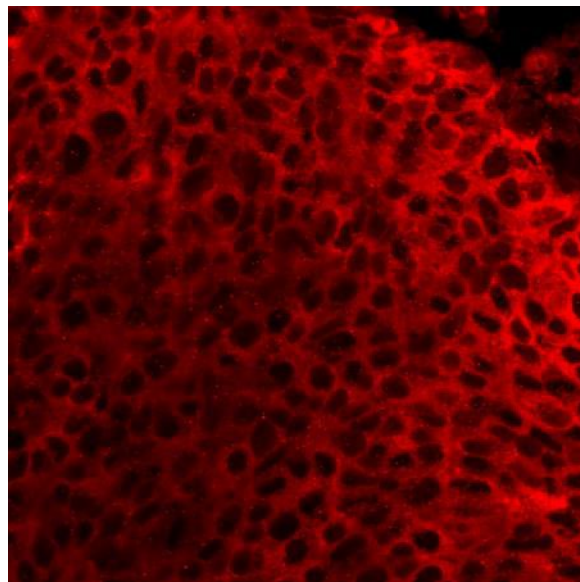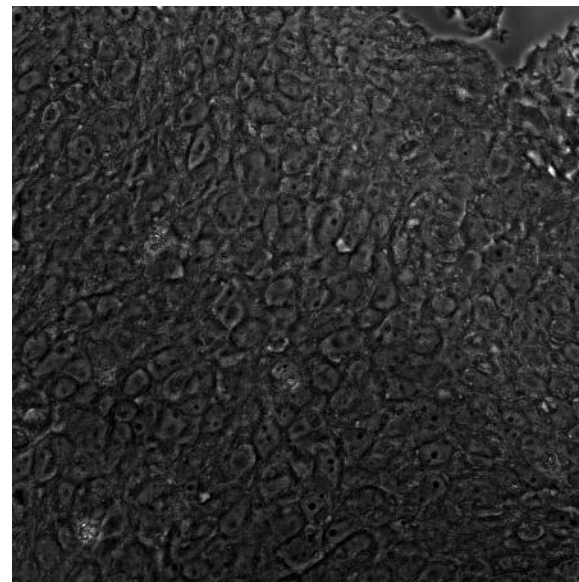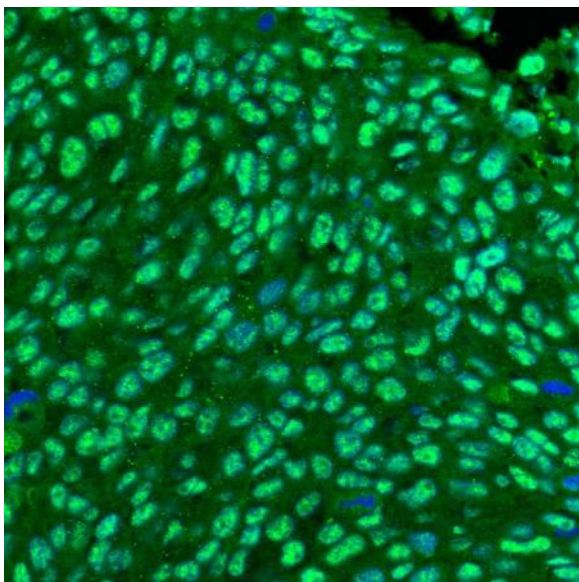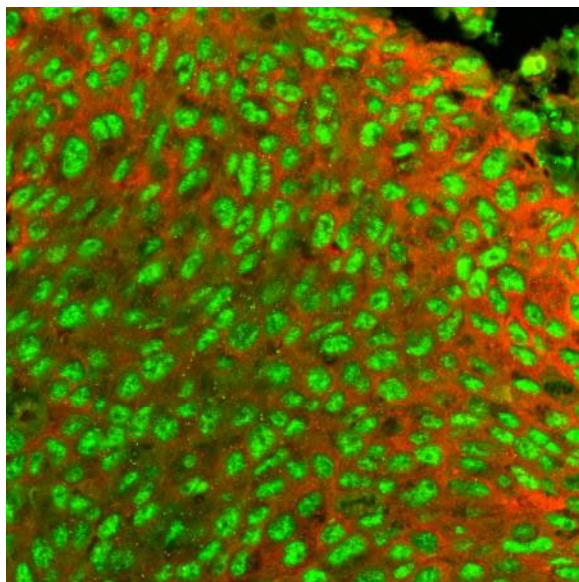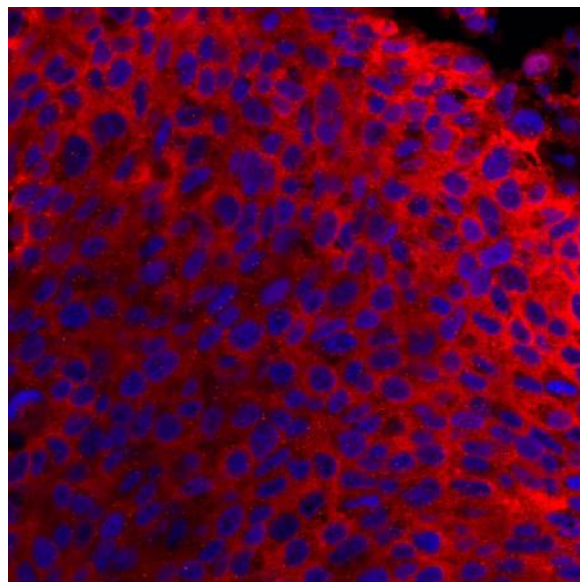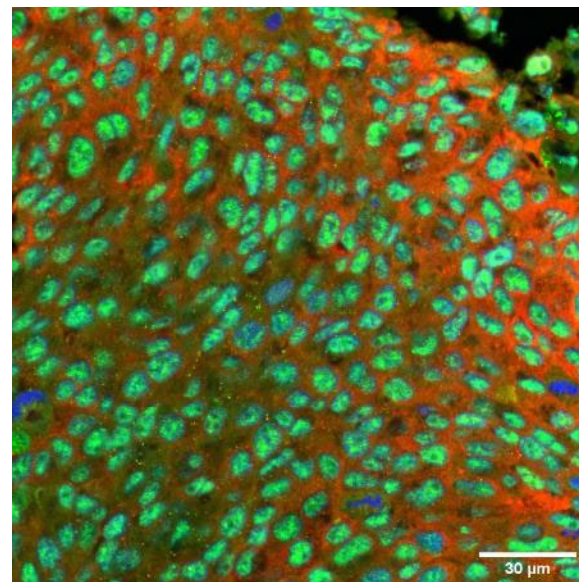

22376\_03

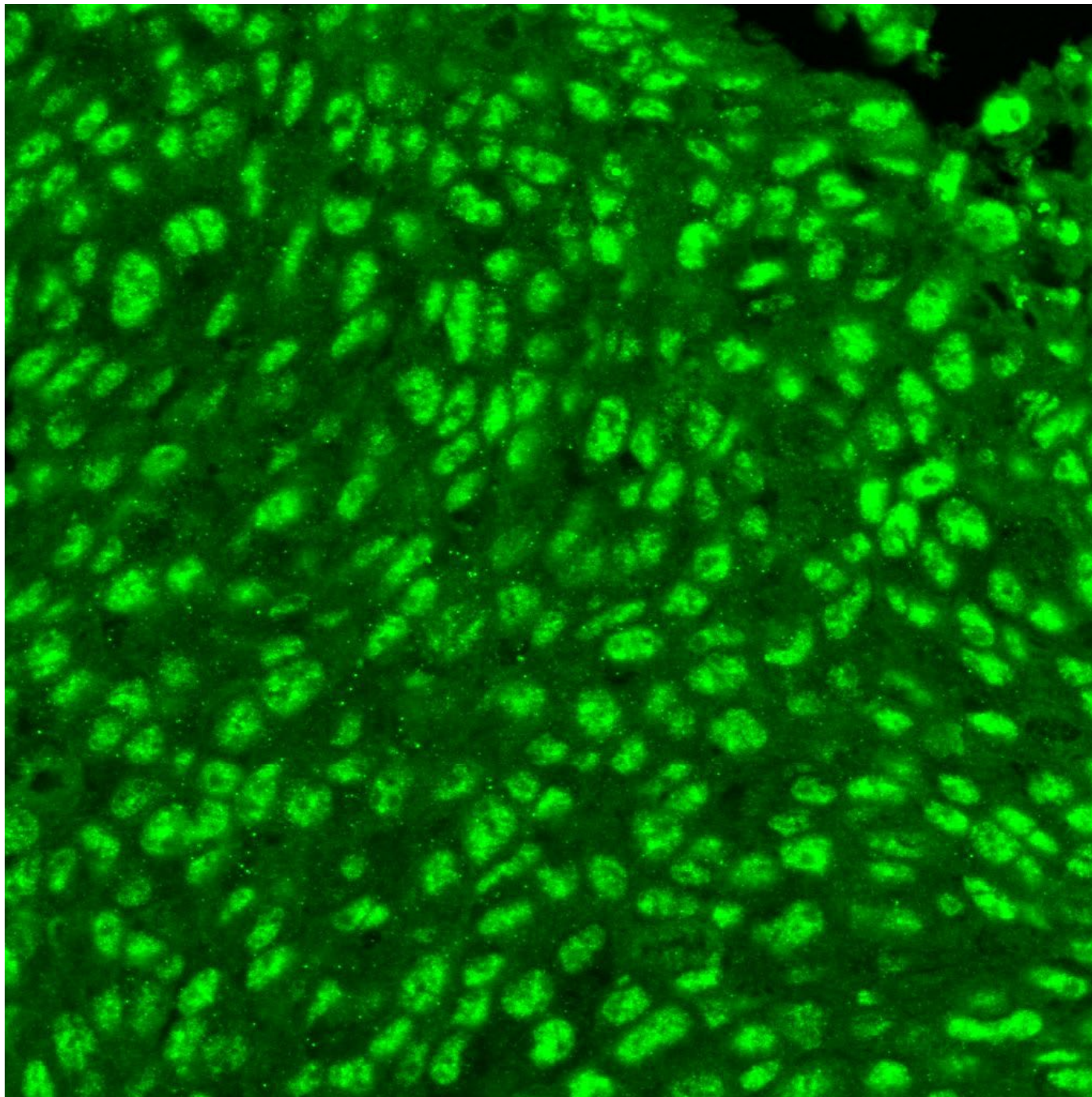

22376\_03

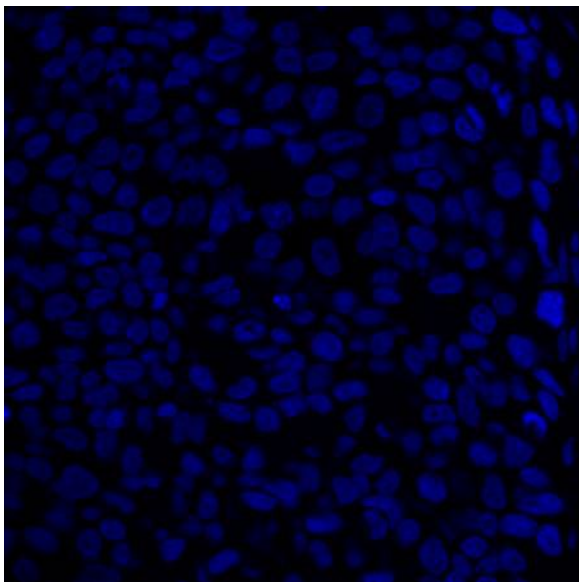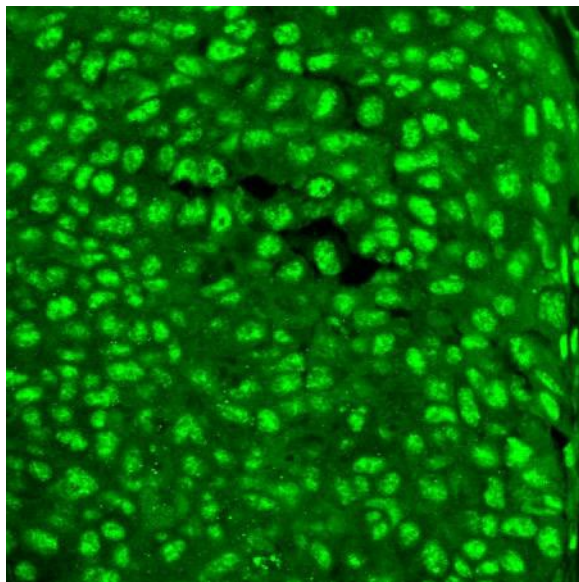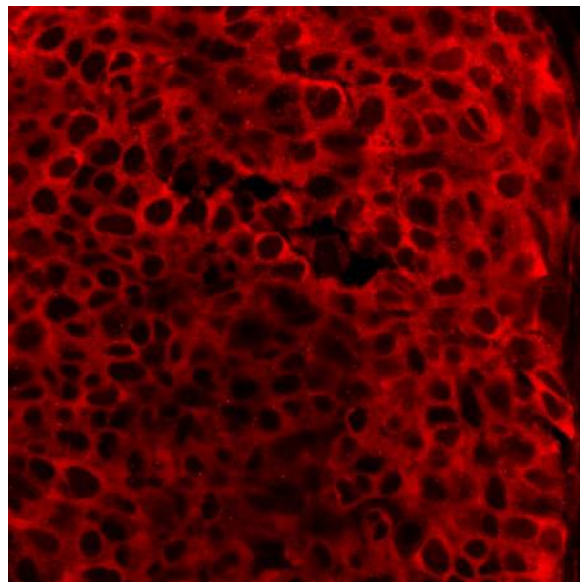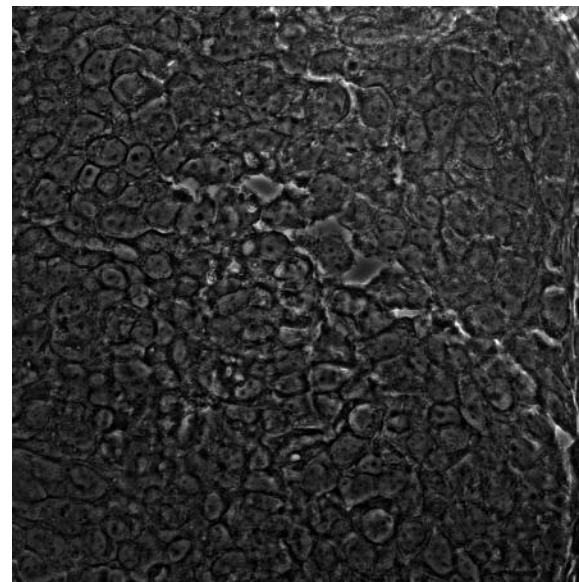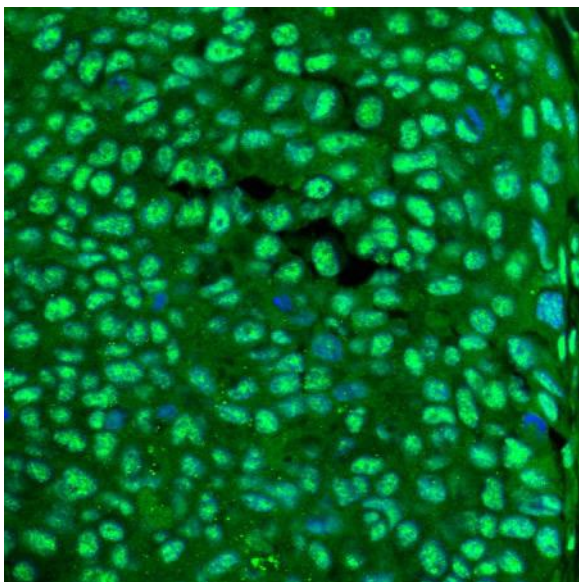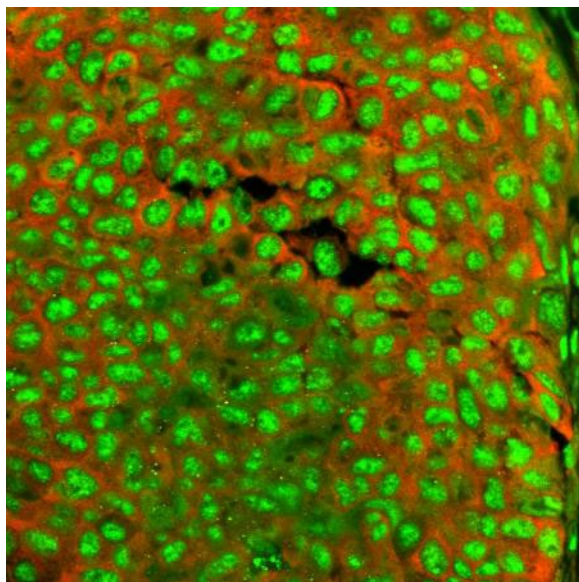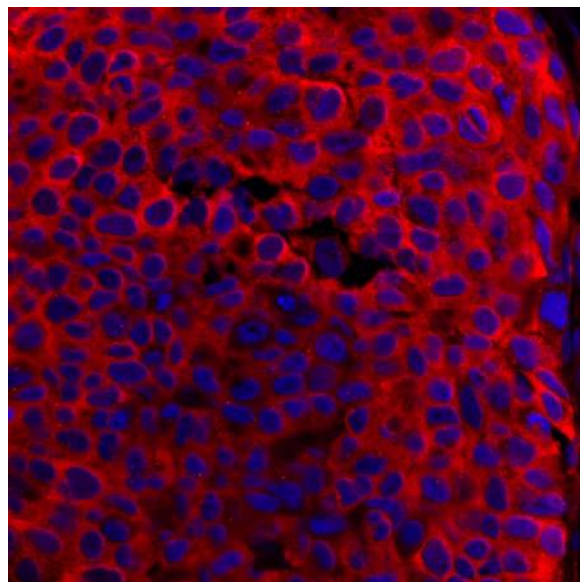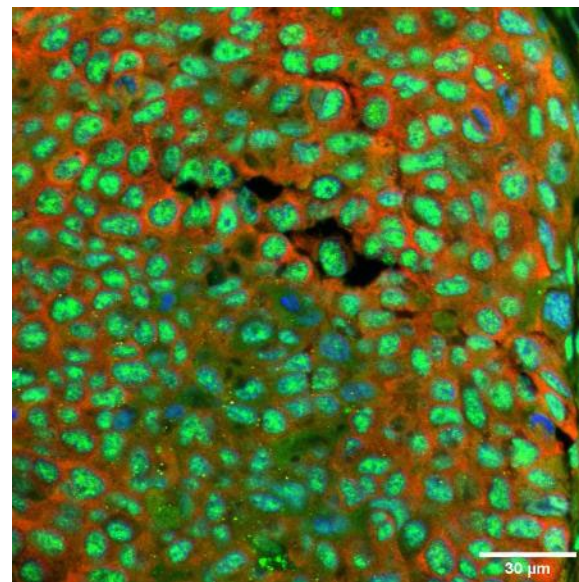

22376\_04

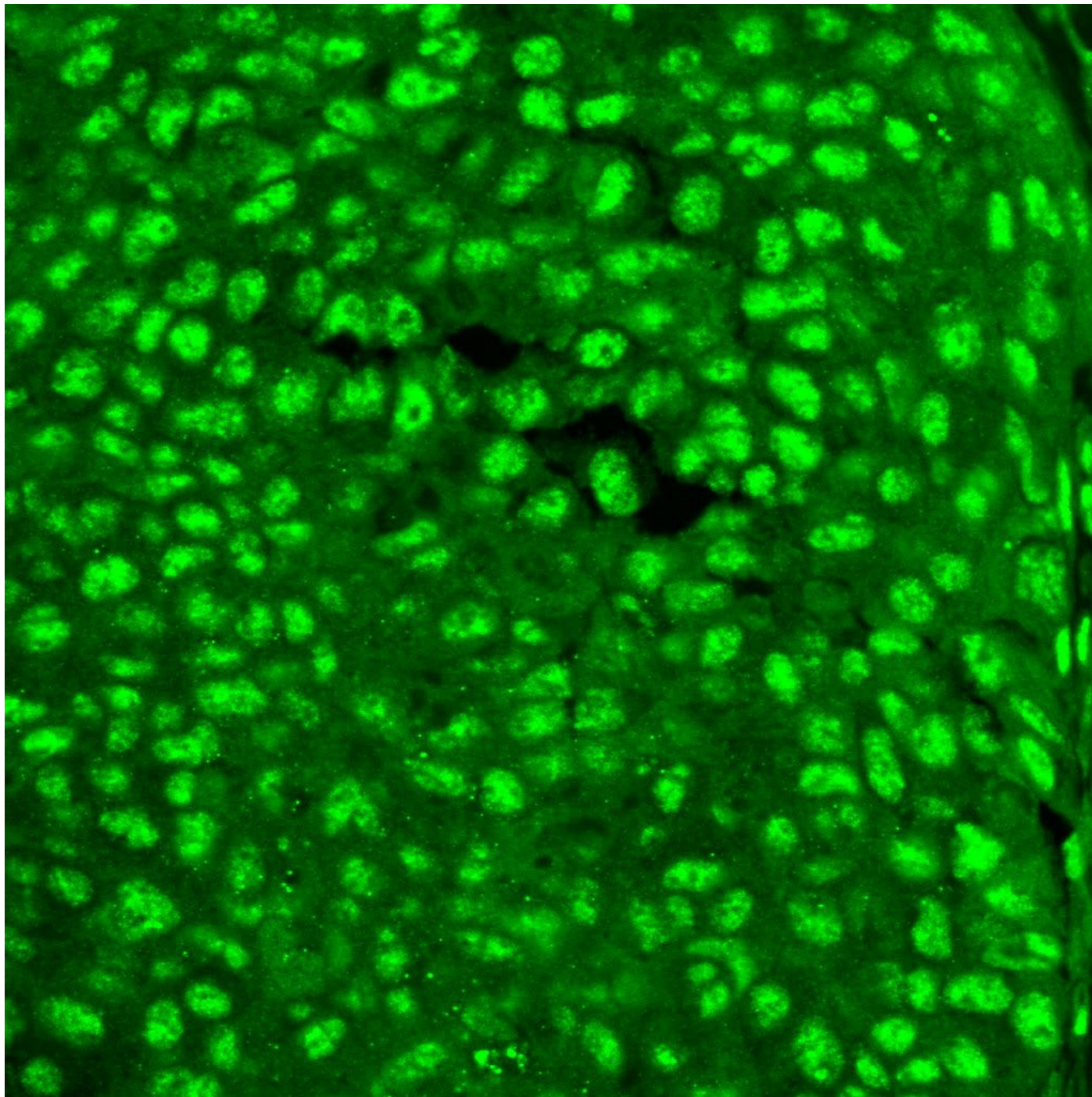

22376\_04

Supplement: Supplementary file 1 [file ijms-24-15869-s001.zip › Figure S3.pdf]
